# Supplementary material for: Angle between DNA linker and nucleosome core particle regulates array compaction revealed by individual-particle cryo-electron tomography
Source: Nat Commun. 2024 May 23;15:4395. doi: 10.1038/s41467-024-48305-1 (PMC11116431; doi:10.1038/s41467-024-48305-1)
Supplement: Supplementary file 1 — Supplementary Information [file 41467_2024_48305_MOESM1_ESM.pdf]

Supplementary Information for

Angle between DNA linker and nucleosome core particle regulates array compaction by individual-particle cryo-electron tomography

Meng Zhang[1,2,3], César Díaz-Celis[3,4], Jianfang Liu[1], Jinhui Tao[5], Paul D. Ashby[1], Carlos Bustamante[2,3,4,6,7,8,9,10*], Gang Ren[1,*]

[1] The Molecular Foundry, Lawrence Berkeley National Laboratory, Berkeley, USA
[2] Applied Science and Technology Graduate Group, University of California, Berkeley, USA
[3] California Institute for Quantitative Biosciences, University of California, Berkeley, USA
[4] Howard Hughes Medical Institute, University of California, Berkeley, USA
[5] Physical Sciences Division, Pacific Northwest National Laboratory, Richland, WA 99352
[6] Department of Chemistry, University of California, Berkeley, USA
[7] Department of Physics, University of California, Berkeley, USA
[8] Department of Molecular and Cell Biology, University of California, Berkeley, USA
[9] Molecular Biophysics and Integrative Bioimaging Division, Lawrence Berkeley National Laboratory, USA
[10] Kavli Energy Nanoscience Institute, University of California, Berkeley, USA

* Correspondence should be addressed to: G.R. (gren@lbl.gov) and C. B. (carlosb@berkeley.edu)

Supplementary Fig. 1. Development of nucleosome compaction and condensation 2
Supplementary Fig. 2. Morphology and IPET 3D map of mononucleosomes by NS EM 3-4
Supplementary Fig. 3. Validation of the cryo-ET 3D reconstruction resolution 5
Supplementary Fig. 4. Structural variety analyzed Hierarchical clustering 6
Supplementary Fig. 5. Cryo-ET structure of individual di-, tri-, tetranucleosome particles 7-8
Supplementary Fig. 6. Analysis of nucleosome array unwrapping dynamics 9
Supplementary Fig. 7. θ angles distribution and analysis of central dinucleosome unit 10
Supplementary Fig. 8. Distributions of the wrapping angle α and the bending angle β 11
Supplementary Fig. 9. Distribution of the intra-nucleosome NCP distances and dihedral angles 12
Supplementary Fig. 10. 3D structure of tetranucleosome in 50 mM Na^+ and 5 mM with H1 13
Supplementary Fig. 11. Morphology induced by H1 under various Na^+ 14
Supplementary Fig. 12. H1 inducing local networks and conformational change 15
Supplementary Fig. 13. Simulation of chromatin regulated by the experimental distributions 16
Supplementary Fig. 14-60. 3D reconstruction of mono-nucleosome in 5 mM Na^+ 17-63
Supplementary Fig. 61-93. 3D reconstruction of di-nucleosome in 5 mM Na^+ 64-96
Supplementary Fig. 94-138. 3D reconstruction of tri-nucleosome in 5 mM Na^+ 97-141
Supplementary Fig. 139-169. 3D reconstruction of tetra-nucleosome in 5 mM Na^+ 142-172
Supplementary Fig. 170-204. 3D reconstruction of tetra-nucleosome in 50 mM Na^+ 173-206
Supplementary Fig. 205-238. 3D reconstruction of tetra-nucleosome in 5 mM Na^+ with H1 ... 207-239

Supplementary Figures:

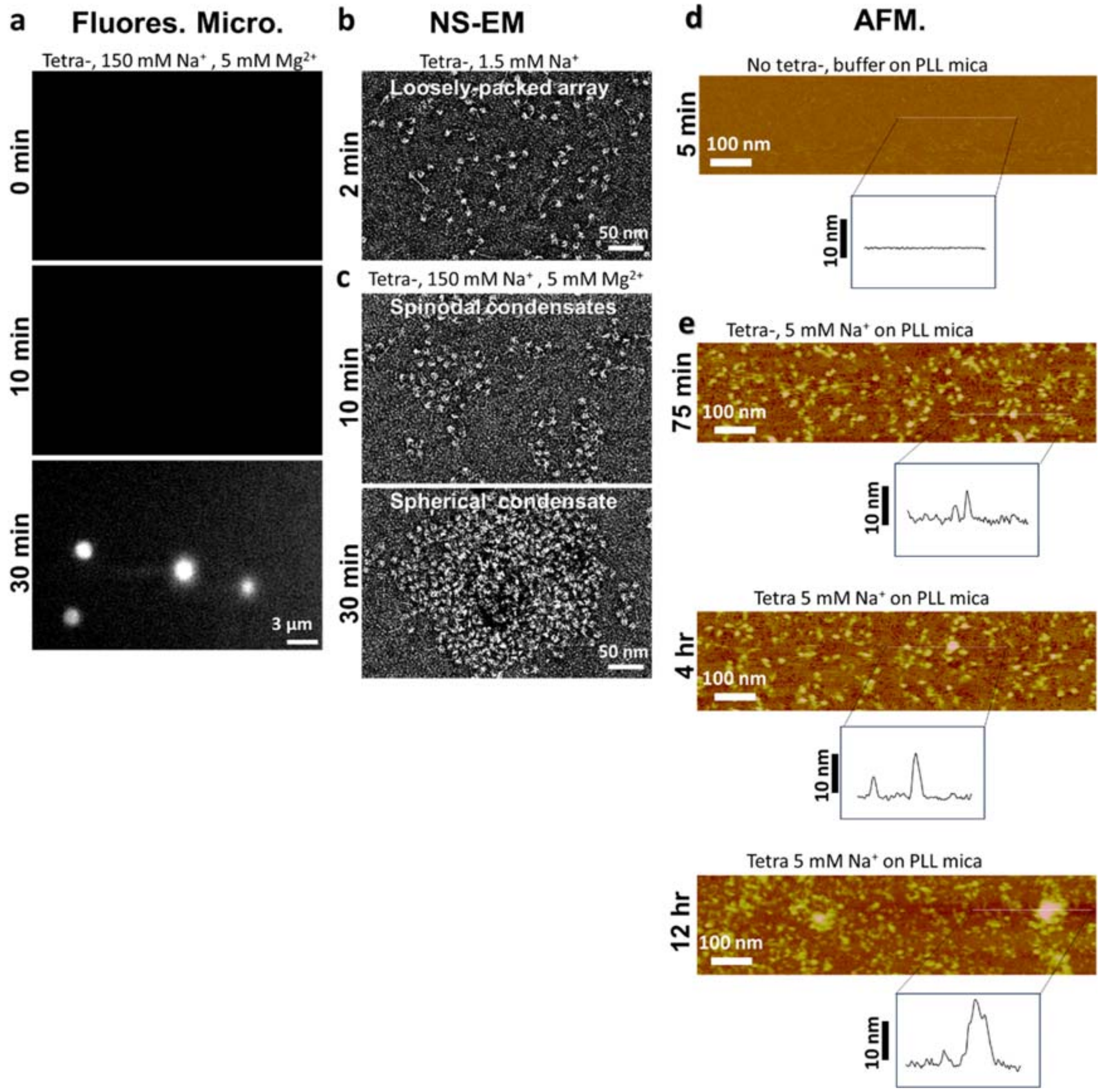

Supplementary Fig. 1: Development of nucleosomal compaction and condensation. a, Phase separation process of tetranucleosome in physiological salt conditions observed by fluorescence microscopy, with tetranucleosomes labeled with Cy3 fluorophore. **b,** NS-EM images show loosely-packed arrays at low-salt (1.5 mM Na^+) conditions, and **c,** inter-array interaction indicating spinodal and spherical condensates at 10 and 30 minutes, respectively, under the physiological salt concentration (150 mM Na^+, and 5 mM Mg^{2+}). **d,** AFM observation of spinodal condensate conformation. The control image at the top panel is from a sample without tetranucleosome, containing only HEPES buffer with 5 mM Na^+ after 5 minutes incubation. **e,** The remaining three images are from the samples containing the 30 nM tetranucleosome in 20 mM HEPES buffer with 5 mM Na^+ after 75 minutes, 4 hours, and 12 hours of incubation, respectively.

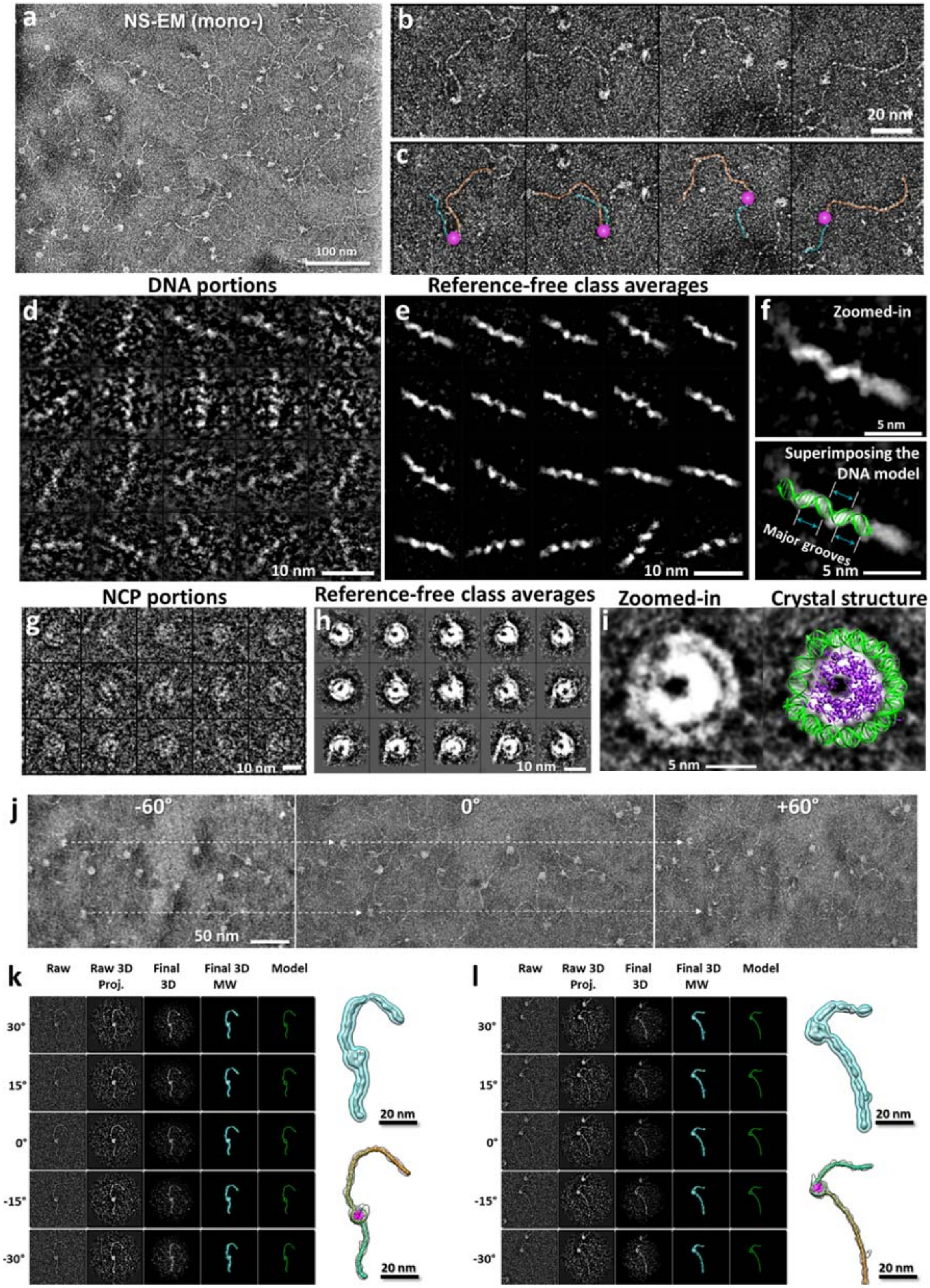

Supplementary Fig. 2: Morphology and IPET 3D map of mononucleosomes by NS EM. a, Survey NS-EM micrography of mononucleosomes particles assembled using histone octamers on a 447-bp DNA template with 200-bp and 100-bp entry and exit DNA arms in low salt condition (5 mM Na^{+}). **b,** Four representative particles with **c**, their overlapped carton showing particle morphology, where the core particles, entry and exit DNA arms are marked by pink, orange and cyan, respectively. **d**, Zoomed-in

images of DNA arms segments, and **e**, selected reference-free class averages, with **f**, one zoomed-in image of a representative class averages (top panel), super-imposed with a standard dsDNA model (bottom panel), where the major groove is indicated by cyan arrows. **g**, Representative images of NCPs portion and **h**, their reference-free class averages. **i**, Zoomed-in image of a representative averaged NCP particle (left), superimposed with the crystal structure 1AOI (right). **j**, Representative tilt-series images of mononucleosome sample acquired by NS-ET, linking targeted particles with dashed arrows. **k**,**l**, 3D reconstruction of two NS-ET mononucleosome particles. The IPET 3D reconstruction refinement steps are displayed in the left panels. The zoomed-in views of the final 3D-maps, superimposed with their flexible fitted model, are shown in the left panels. The histone, entry, and exit DNA arms are colored in pink, orange and cyan, respectively.

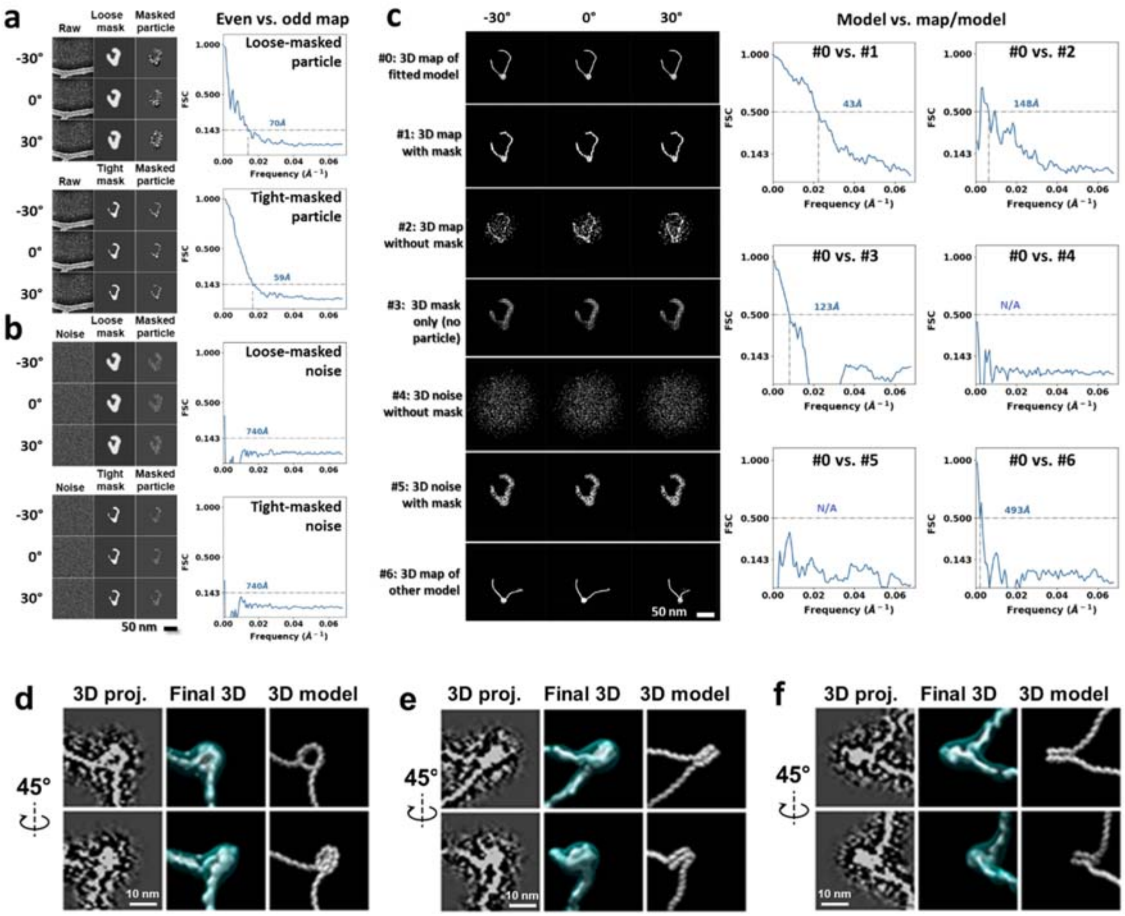

Supplementary Fig. 3: Validation of the cryo-ET 3D reconstruction resolution. a, Resolution comparison demonstrating that reconstructions of cryo-ET samples are not sensitive to the mask size used. Both loose (top panel) and tight (bottom panel) particle-shaped mask were applied to the same aligned images before 3D reconstructions and resolution estimation using their even and odd indices of tilt series. **b**, Resolution comparison showing that masks alone do not contribute to the high resolution. Particle-shaped masks were applied to simulated noise images for 3D reconstructions and resolution estimation. **c**, Resolution evaluation by comparing the FSCs between the fitted model density (#0) with different maps (#1-6), including the final 3D reconstruction after masking (#1), before masking (#2), the 3D map of the mask (#3), the 3D reconstruction from the noise images (#4), the 3D reconstruction of the masked noise images (#5), and the 3D density map of another particle fitted model (#6). Masks #1,3,5 used the same tight particle-shaped mask. **d,e,f,** Three representative particle showed from two directions (45°), displayed by the projection of the final 3D density map, two isososurphace contour maps, and fitting model. The observed spatial orientation of 2-nm dsNDA linker validates the reported 3D map resolution.

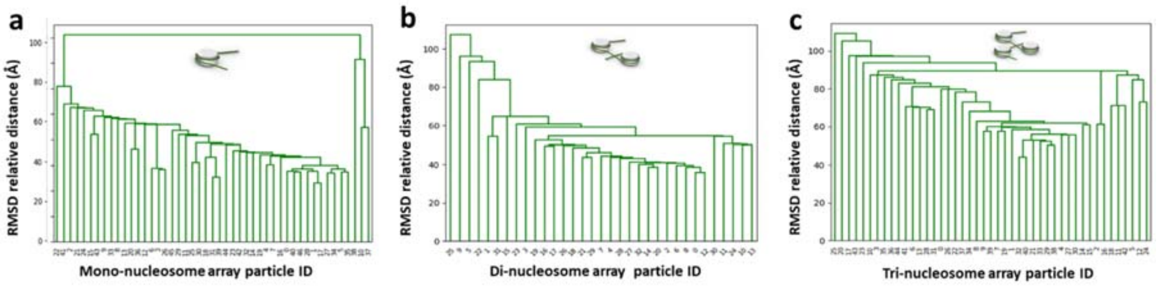

Supplementary Fig. 4: Structural variety analyzed Hierarchical clustering. A dendrogram from hierarchical clustering analysis of RMSD values, with structures on the X-axis and cluster distance on the Y-axis, showing **a**, mononuclesome, **b**, dinucleosome, and **c**, trinucleosomes.

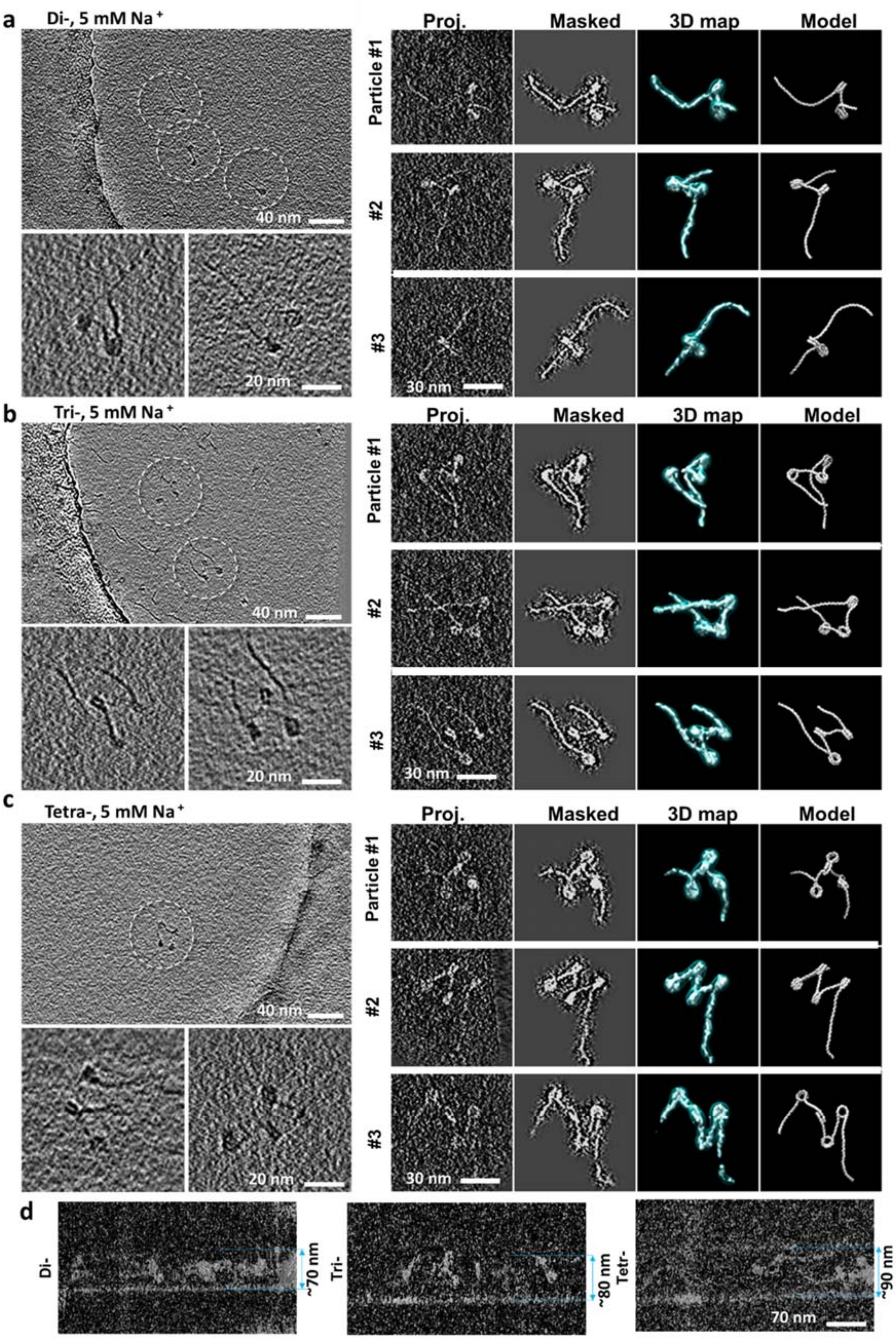

Supplementary Fig. 5: Cryo-ET 3D reconstructions of individual di-, tri-, tetranucleosome particles. a, Survey cryo-EM micrograph of a reconstituted dinucleosome array in 20 mM HEPES

buffer with 5 mM Na^+ (top left panel). Target particles are highlighted with dashed-line circles. Two representative z-dimensional slices (3 nm thickness) of dinucleosome particle reconstructions are shown in the bottom left panel. The 3D projection of the mask-free initial 3D reconstruction, the projection of the masked 3D reconstruction, the final 3D density map displayed at two contour levels, and the flexible fitted model of the targeted particle (column 1 through 4, respectively, right panel) are compared. **b**,**c**, The same representation scheme is applied to the trinucleosome (**b**) and tetranucleosome (**c**) array samples in 20 mM HEPES buffer with 5 mM Na^+. **d**, Three representative z-direction views of di-, tri-, tetranucleosome samples showing their particle distributions within the ice in thickness of ~70-90 nm.

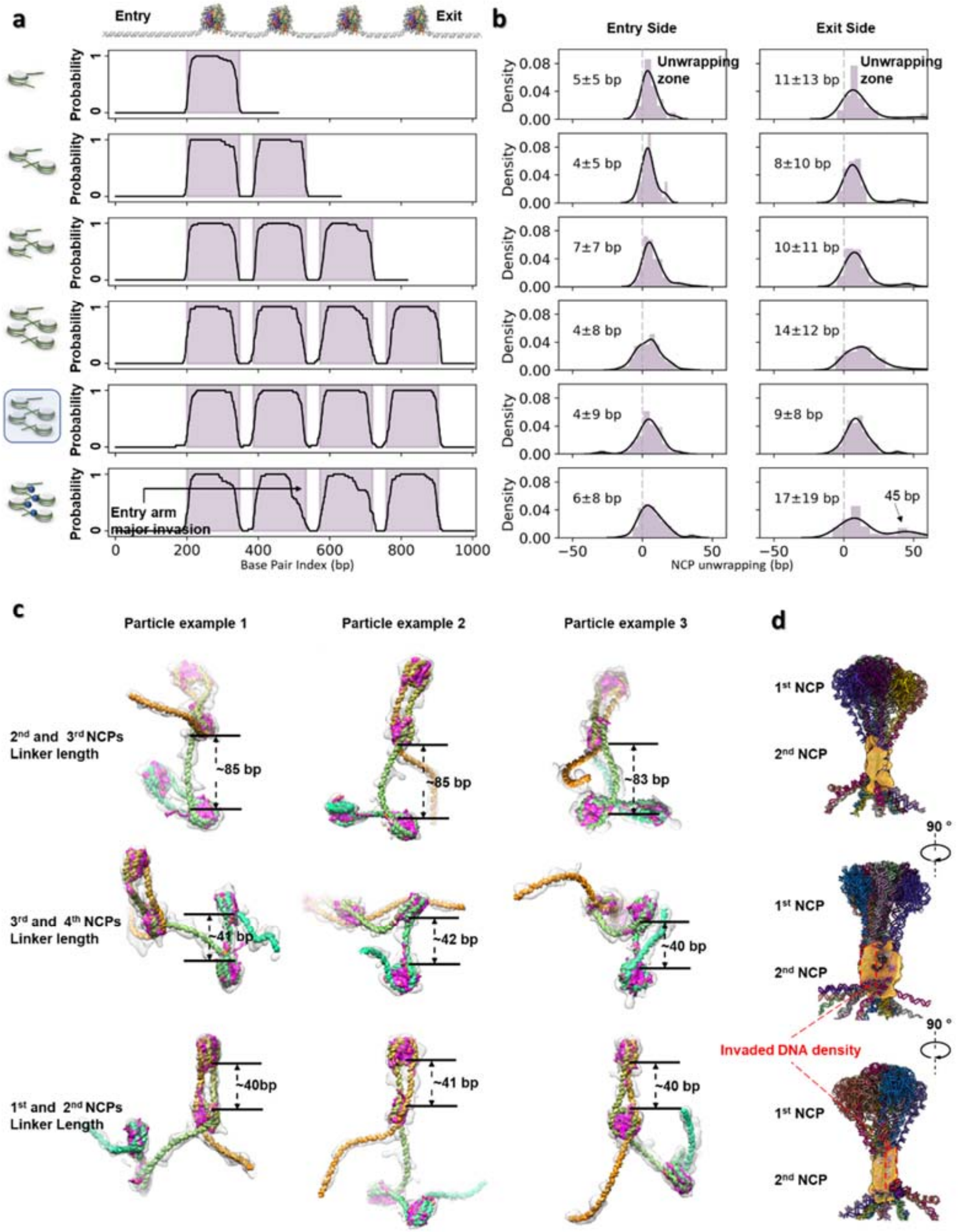

Supplementary Fig. 6: Analysis of nucleosome array unwrapping dynamics. **a**, Histone octamer positioning along the DNA template of mono-, di-, tri-, tetranucleosome array at 5 mM Na^+, tetranucleosome array at 50 mM Na^+, and tetranucleosome array at 5 mM Na^+ in the presence of H1 (row 1 through 6, respectively). The octamer positioning is presented as the probability of finding specific DNA base pairs in contact with the octamer surface (black line). The designed 601-regions on each DNA template are highlighted by filled magenta boxes. **b**, Histograms show the corresponding DNA unwrapping distributions on the entry (left panel) and exit (right panel) side of all NCPs in (**a**). The positive value zone represents the events of unwrapping, while the negative zone indicates fully wrapped arms extending along the surface of the NCP beyond the exit point. **c**, Three representative tetranucleosome particles at 5 mM Na^+ in the presence of H1 displaying a longer link length between second and third NCPs. **d,** Structure alignment based on the second NCP models (rainbow color) and averaging of their density maps (orange color). The invaded DNA density is highlighted in red dashed-line boxes.

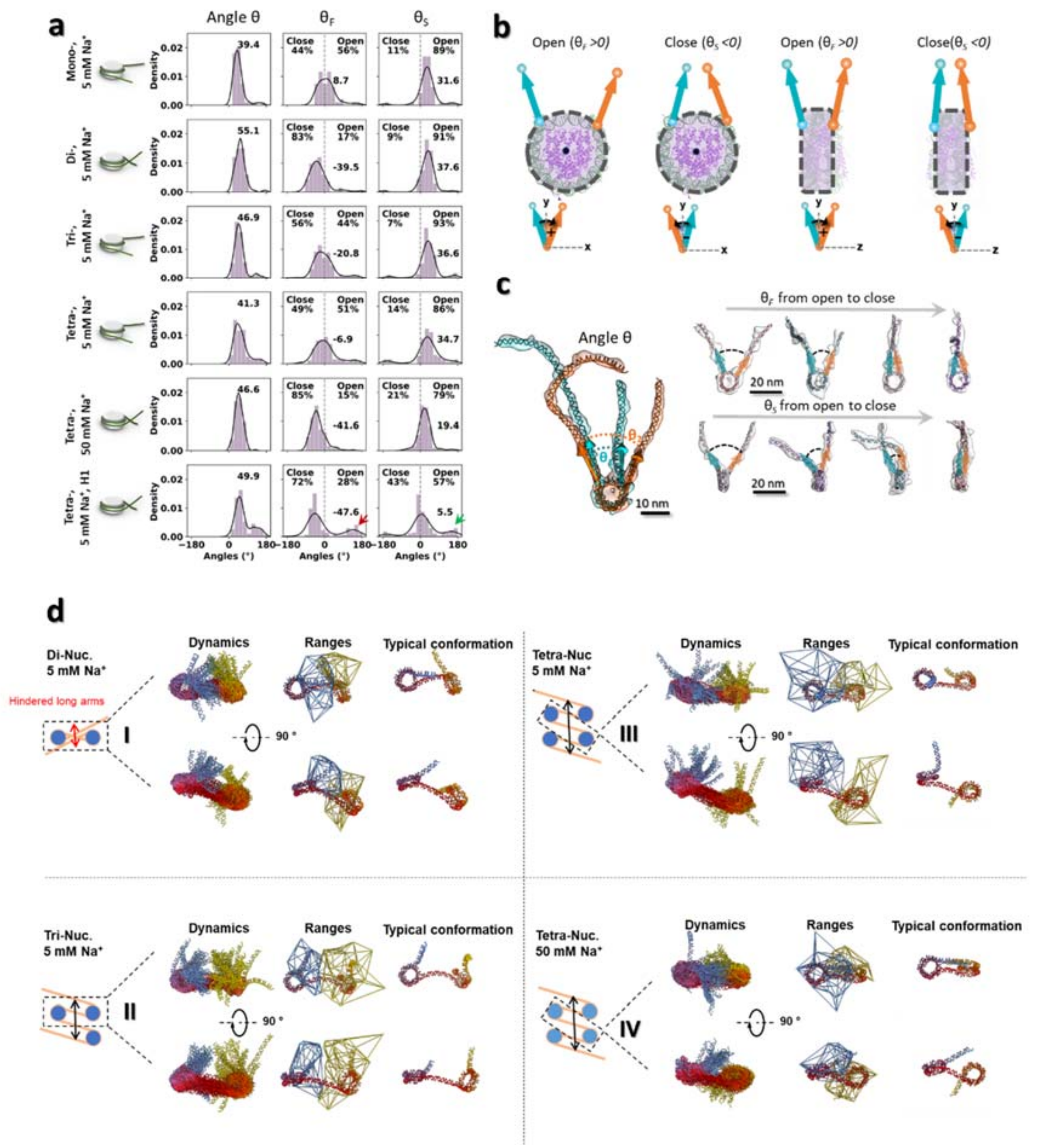

Supplementary Fig. 7: Distribution of the nucleosome array θ angles and analysis of array central dinucleosome unit morphology. a, Distribution of the $\boldsymbol{\theta}$ angle between two DNA linkers (first column) measured from all NCP units of different type of nucleosome arrays (schematic on left). The corresponding $\boldsymbol{\theta}_{\parallel}$ and $\boldsymbol{\theta}_{\perp}$ fraction are shown in second and third column, respectively. **b**, Definition of the open-closed arm/linker conformation of an NCP by the sign of its $\boldsymbol{\theta}_{\parallel}$ and $\boldsymbol{\theta}_{\perp}$ fraction. **c**, Representative 3D reconstructions showing the dynamic of the $\boldsymbol{\theta}$ angle (left) and its two fractions in orthogonal views (right) (planes parallel and perpendicular to the NCP discoidal plane, respectively). **d**, The central dinucleosome unit dynamics of di- (I), tri- (II), and tetranucleosome (III) array in 5 mM Na$^+$ and the latter in 50 mM Na$^+$ (IV). The central dinucleosome components (40-147-40-147-40, dashed-line box) of arrays within the same category are extracted and aligned by minimizing their NCPs RMSD (left schematics). The red arrow marks the close distance between two distal DNA arms in dinucleosome array in contrast to those spatially separated arms presented in other type of arrays (black arrows). The DNA linker/arm conformational dynamics, dynamic range, and their typical conformation of each array categories are shown in column one through three. The DNA are rainbow colored from blue, red to yellow along the DNA entry-exist direction.

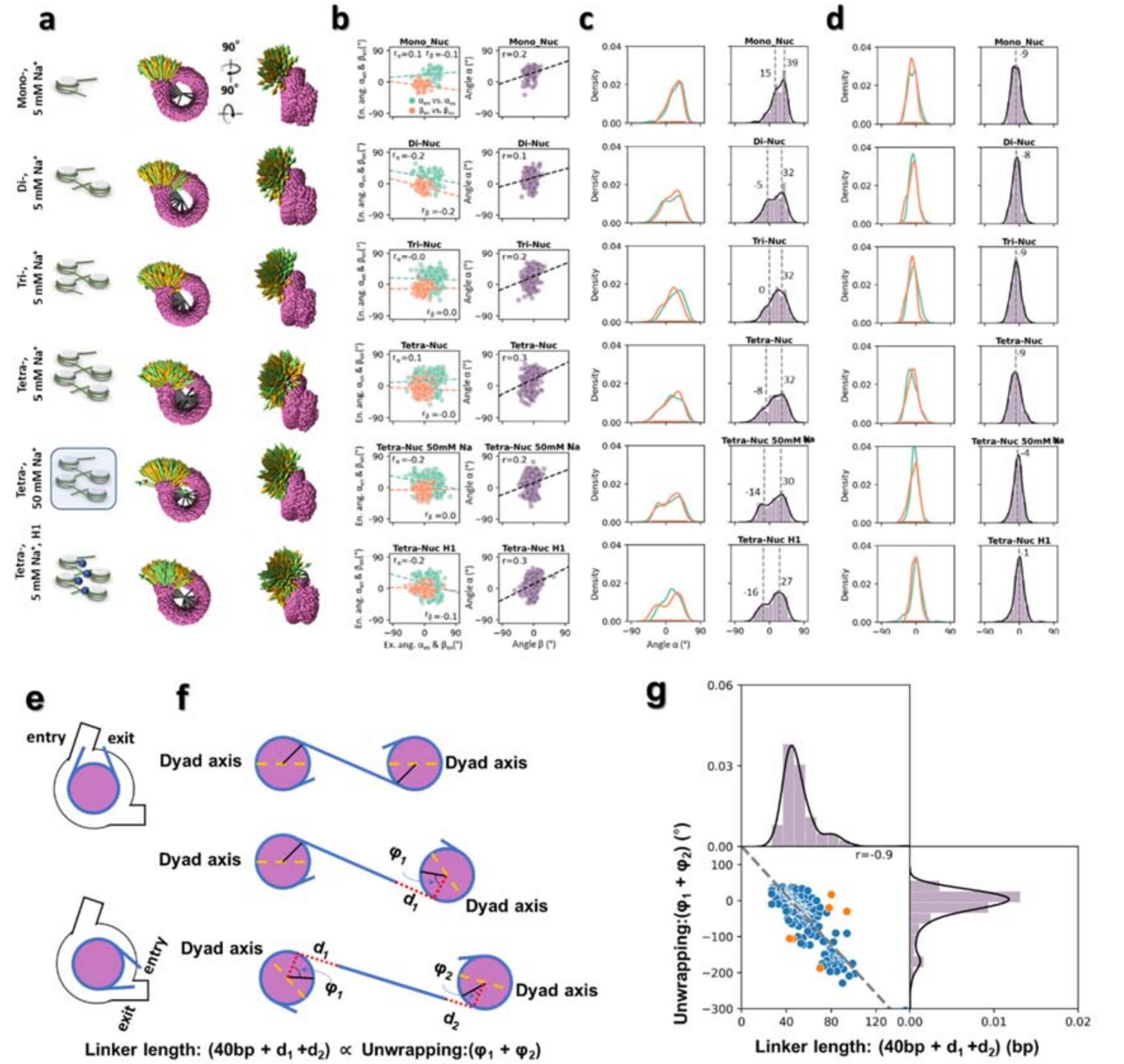

Supplementary Fig. 8: Distributions of the wrapping angle α and the bending angle β. a, Superimposed vectors of the entry (yellow) and exit (green) DNA linkers on a consensus nucleosome model (magenta) for each array categories (left schematics), shown in orthogonal views. **b**, The Pearson correlation analysis between the two wrapping angles, α_{en} vs. α_{ex} (cyan) and between the two bending angles, β_{en} vs. β_{ex} (orange) on the same NCPs within the corresponding array category (left column). The correlation analysis is also performed between the same side of wrapping angle and bending angle of NCPs (purple) within each category (right column). **c**, Distribution of α_{en} and α_{ex} shown in cyan and orange, respectively (left column) and their pooling (right column). **d**, Distribution of β_{en} and β_{ex} shown in cyan and orange, respectively (left column) and their pooling (right column). **e-g,** Evaluate nucleosome array model fitting by correlating unwrapping angle with linker length. **e**, Schematic of two possible NCP fitting orientations for an unwrapped NCP map. **f**, NCP unwrapping leads to longer linker length, proportional to degree of unwrapping (measured as φ angle compared to fully wrapped crystal structure). **g**, Correlation analysis between DNA linker length and NCP unwrapping angle from all NCP pairs, with orange points as fitting outliers.

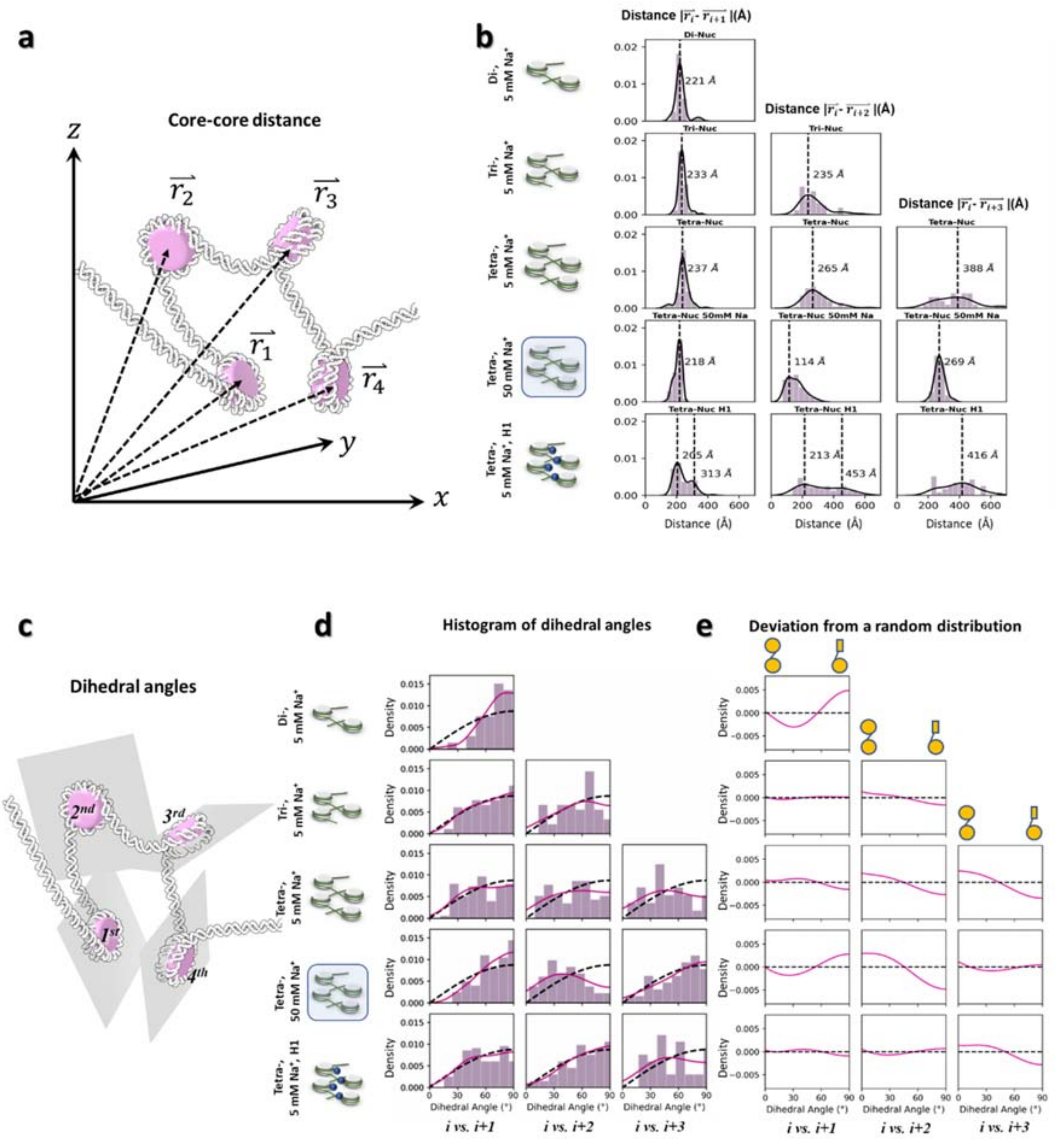

Supplementary Fig. 9: Distribution of intra-nucleosome array NCP distances and dihedral angles. **a**, Schematics of the vector, $\vec{r_i}$ starting from the origin and pointing to the center of mass of each NCP. **b**, Histograms of the measured distances between each pair of NCP centers within the array. **c**, Schematics defining the NCP central discoidal planes within the array. **d**, Histograms of the dihedral angle between each pair of NCP discoidal planes. The measured angle distributions (pink line) are compared with a sine function (black dashed-line, presenting the angle distribution of a randomly rotated plane against a fixed plane). **e**, The deviation of the dihedral angle distribution from the sine function indicates a preferred core-to-core conformational arrangement (schematics on top of each column).

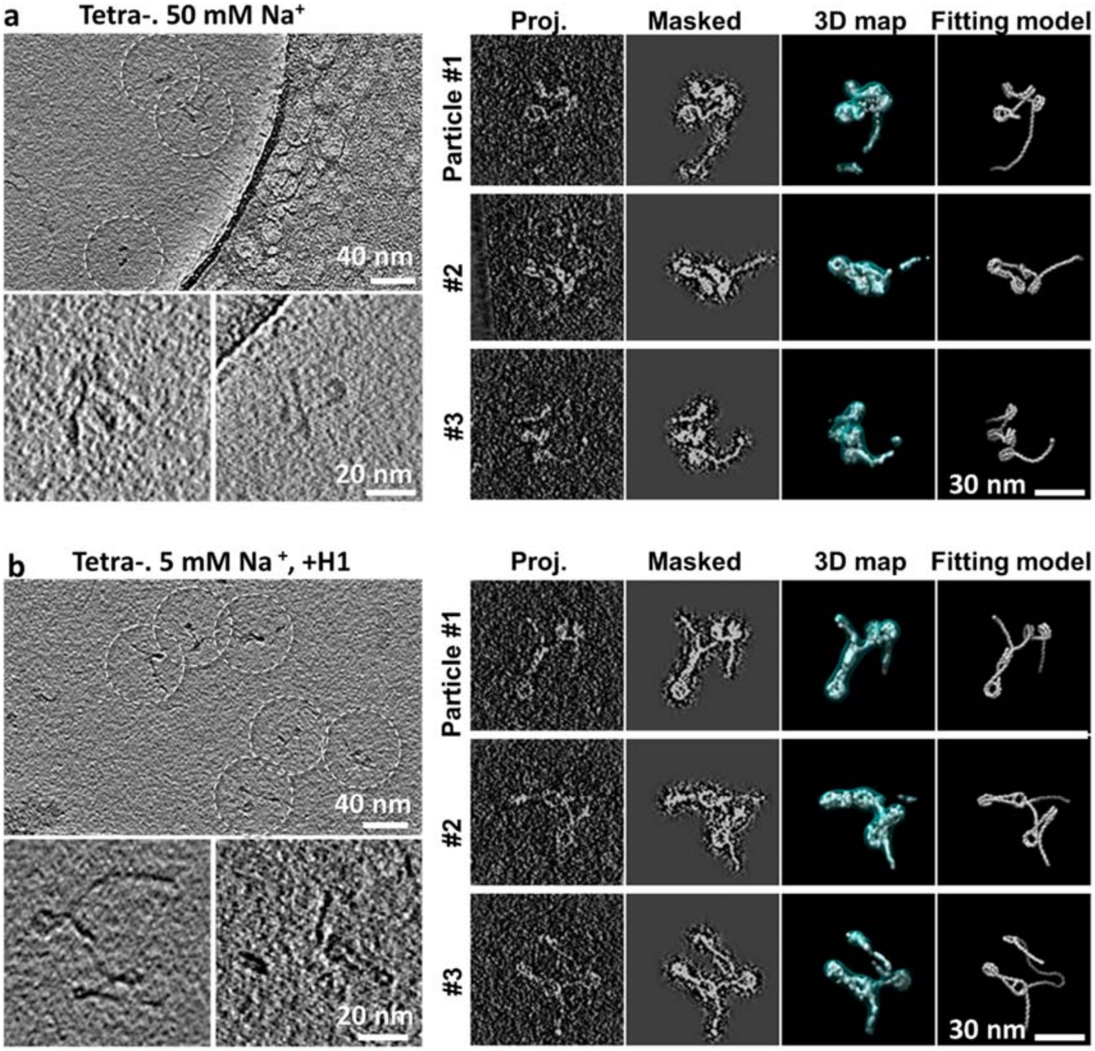

Supplementary Fig. 10: Cryo-ET 3D reconstructions of tetranucleosome particles in high-salt buffer and in the presence of H1. a, Survey micrograph of cryo-EM 3D mapof tetranucleosome array in 20 mM HEPES buffer with 50 mM Na^+ (top left panel). Target particles are highlighted by dashed-line circles. Two representative z-dimensional slices (3 nm thickness) of tetranucleosome particle reconstructions are shown in the bottom left panel. The 3D projection of the mask-free initial 3D reconstruction, the projection of the masked 3D reconstruction, the final 3D density map displayed at two contour levels, and the flexible fitted model of the targeted particle (column 1 through 4, respectively, right panel) are compared. **b**, The same representation scheme is applied to the tetranucleosome array samples in 20 mM HEPES buffer with 5 mM Na^+ in the presence of H1.

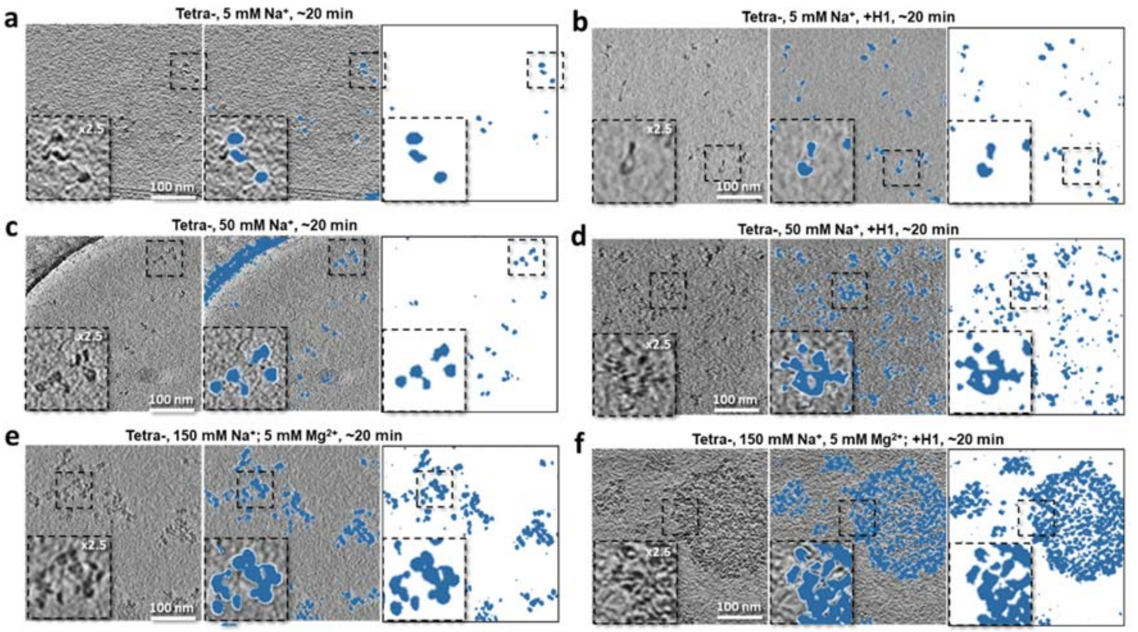

Supplementary Fig. 11: Morphology induced by the present of H1 under various Na$^+$ concentrations. **a**, The Cryo-ET image (left panel) of tetranucleosome in 5 mM Na$^+$ after 20 min incubation at room temperature. The high-density portions are highlighted in blue (left panel) and superimposed on the micrograph (middle panel). **b**, The same sample in present of H1 is compared. **c**, Cryo-ET image of the tetranucleosome in 50 mM Na$^+$ after 20 min incubation at room temperature, **d**, which is compared to same sample in the present of H1. **e**, The Cryo-ET image of the same sample in physiological salt concentration after 20 min of incubation at room temperature, **f**, which is compared to the same sample in the present of H1.

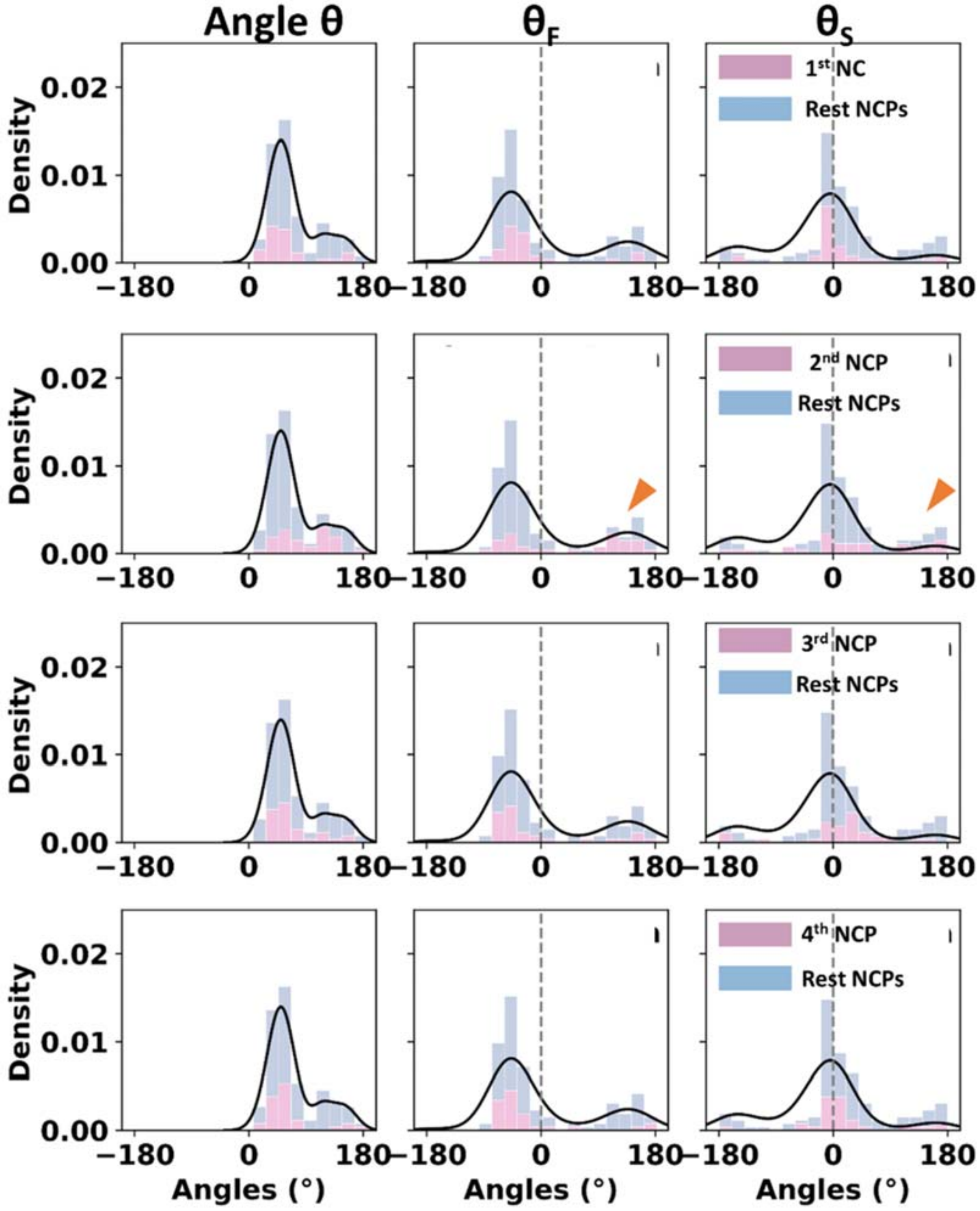

Supplementary Fig. 12: Linker histone H1 inducing local array networks and conformational change of 2nd NCP. Distribution of NCP $\boldsymbol{\theta}$ angles and its two perpendicular fractions measured from tetranucleosome array in the presence of H1 (column 1 through 3). The contribution of each NCP unit to the sub-population (minor peak of the distribution) is plotted individually in pink color (row 1 through 4). The orange arrows indicate that the minor peak is primary caused by the second NCP.

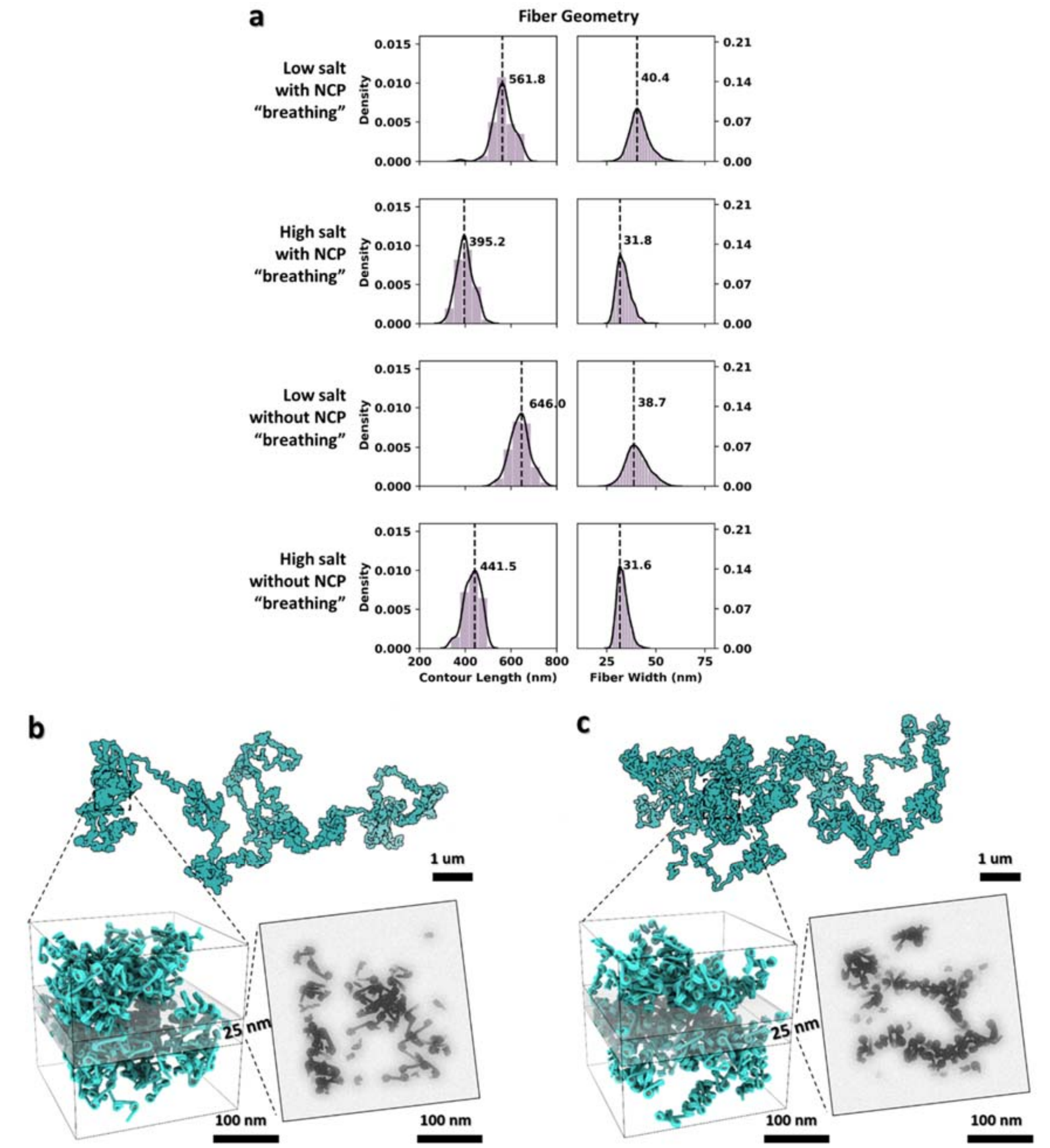

Supplementary Fig. 13: Simulation of the chromatin morphology regulated by the experimental distribution. a, Distribution of in-silico hectanucleosome array fiber length, width, and NCP density and the statistics of the experimentally measured array parameters. Histograms of the measured contour length (left columns) and width (right column) of the simulated nucleosome array fiber with 100 NCP units. 100 simulated array fibers are generated for each category (row 1 through 4) to calculate the statistics. **b,c**, Simulation of a mini-chromosome by threading 250,000 NCPs using the parameters measured from the tetranucleosome arrays in 5 mM and 50 mM Na^{+}, respectively. Representative 200-nm cubic volumes (middle panel) and their central slices with a thickness of 25 nm (right panel) were cropped from the high-density regions of each structure.

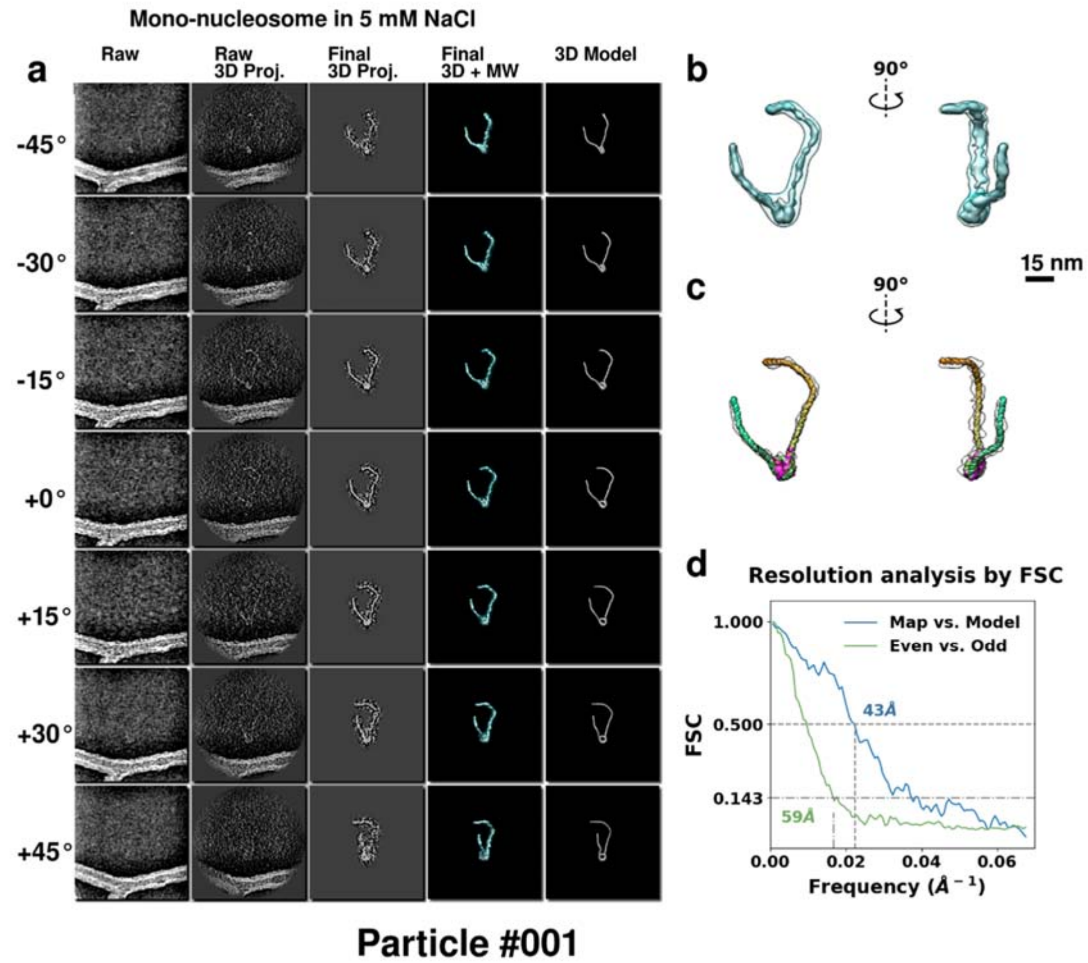

Supplementary Fig. 14: Cryo-ET 3D reconstruction of individual mono-nucleosome particle (index no. 1) in 5 mM NaCl. **a**, IPET 3D reconstruction of individual mono-nucleosome particles. The first column shows seven representative tilt images of an individual particle after CTF correction. Through alignment of the tilt images to a common center for 3D reconstruction via iterative refinement, the second and third columns display the 3D projections of the reconstruction before and after particle-shaped masking, respectively. The fourth column shows the final 3D reconstruction with missing wedge correction, and the fifth column presents the flexibly fitted model at the corresponding tilt angles. **b**, Zoomed-in view of the final 3D density map displayed in orthogonal views, shown at two contour levels. **c**, Superimposition of the high contour level map from (b) onto its flexibly fitted model. **d**, Resolution evaluation of the final 3D density map using two criteria: Fourier shell correlation (FSC) between two-half maps reconstructed from the even and odd index of the tilted series and FSC between the final 3D map and the fitted structure model. The resolution for the former and latter criteria is evaluated at frequencies of 0.5 and 0.143, respectively.

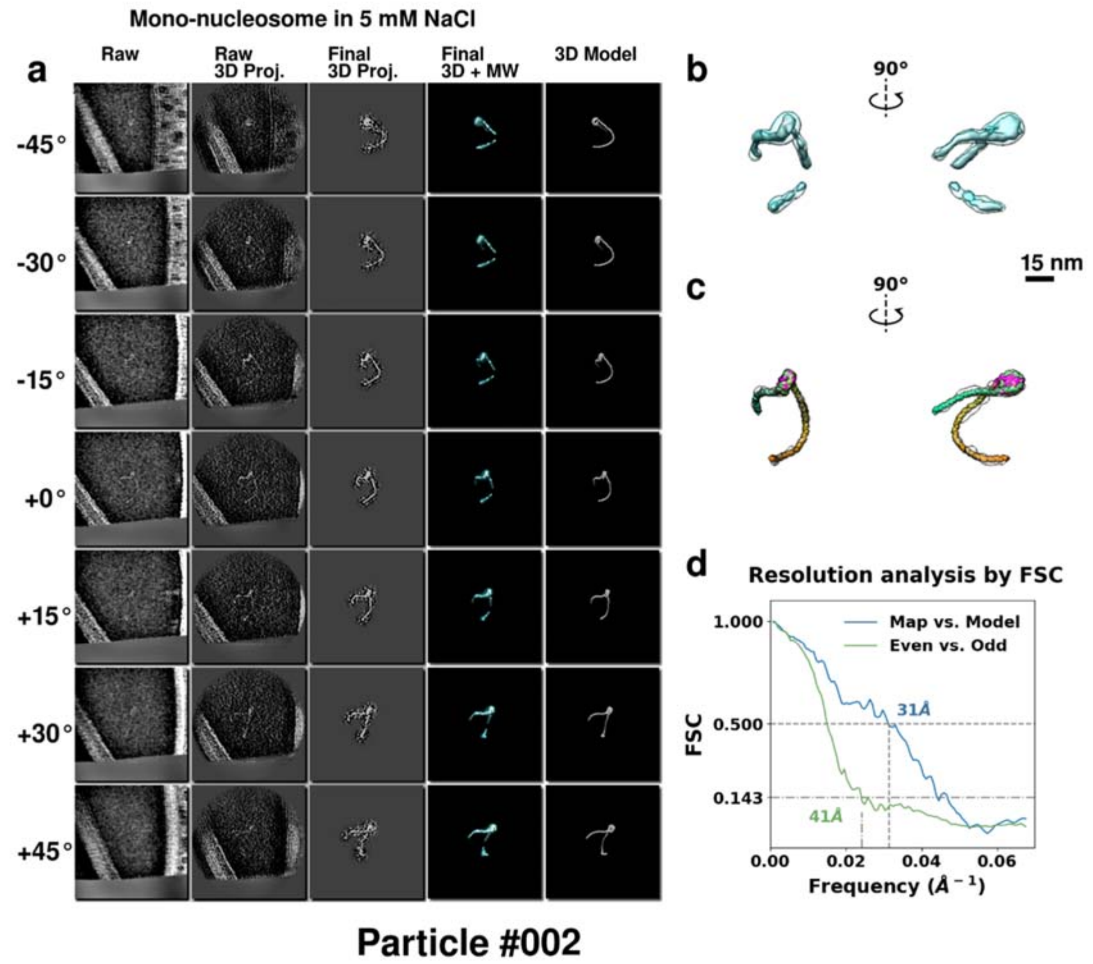

Supplementary Fig. 15. Cryo-ET 3D reconstruction of individual mono-nucleosome particle (index no. 2) in 5 mM NaCl. a, IPET 3D reconstruction of individual mono-nucleosome particles. The first column shows seven representative tilt images of an individual particle after CTF correction. Through alignment of the tilt images to a common center for 3D reconstruction via iterative refinement, the second and third columns display the 3D projections of the reconstruction before and after particle-shaped masking, respectively. The fourth column shows the final 3D reconstruction with missing wedge correction, and the fifth column presents the flexibly fitted model at the corresponding tilt angles. **b**, Zoomed-in view of the final 3D density map displayed in orthogonal views, shown at two contour levels. **c**, Superimposition of the high contour level map from (b) onto its flexibly fitted model. **d**, Resolution evaluation of the final 3D density map using two criteria: Fourier shell correlation (FSC) between two-half maps reconstructed from the even and odd index of the tilted series and FSC between the final 3D map and the fitted structure model. The resolution for the former and latter criteria is evaluated at frequencies of 0.5 and 0.143, respectively.

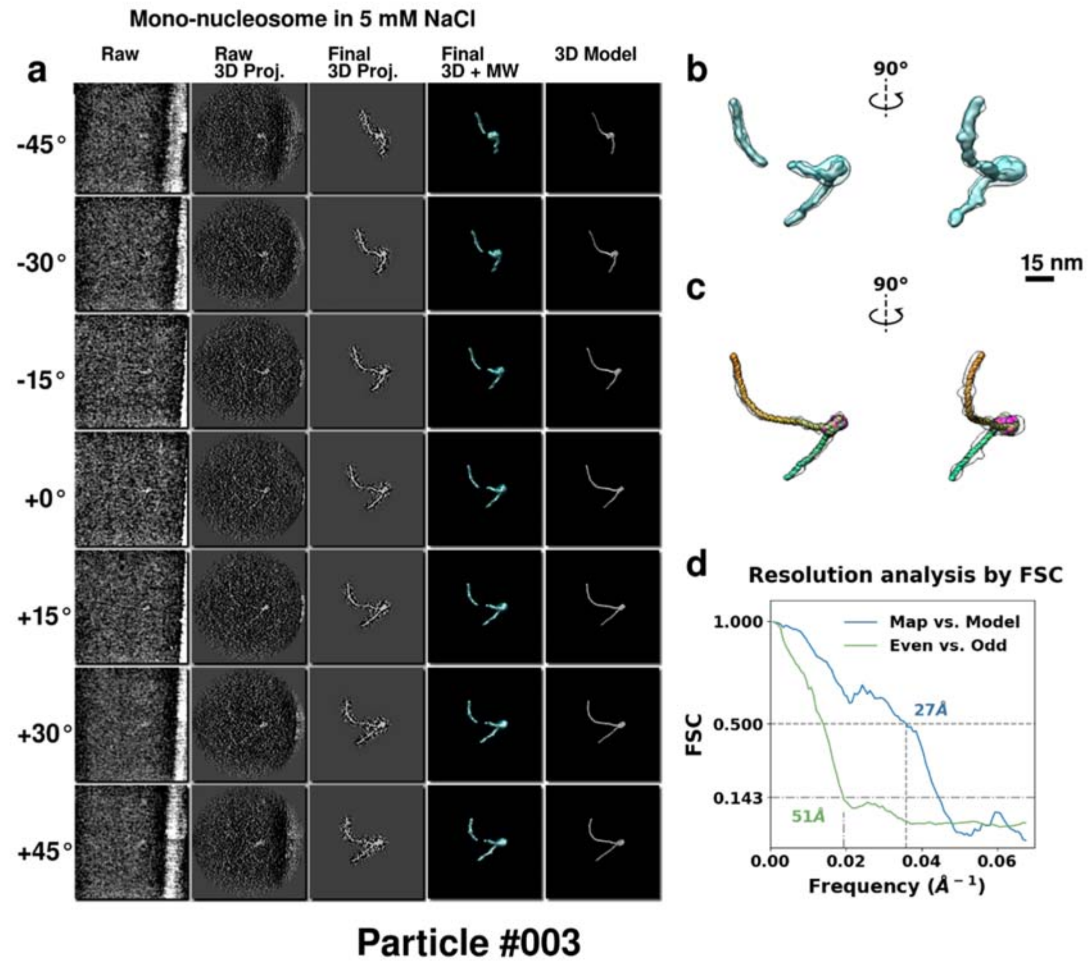

Supplementary Fig. 16. Cryo-ET 3D reconstruction of individual mono-nucleosome particle (index no. 3) in 5 mM NaCl. a, IPET 3D reconstruction of individual mono-nucleosome particles. The first column shows seven representative tilt images of an individual particle after CTF correction. Through alignment of the tilt images to a common center for 3D reconstruction via iterative refinement, the second and third columns display the 3D projections of the reconstruction before and after particle-shaped masking, respectively. The fourth column shows the final 3D reconstruction with missing wedge correction, and the fifth column presents the flexibly fitted model at the corresponding tilt angles. **b**, Zoomed-in view of the final 3D density map displayed in orthogonal views, shown at two contour levels. **c**, Superimposition of the high contour level map from (b) onto its flexibly fitted model. **d**, Resolution evaluation of the final 3D density map using two criteria: Fourier shell correlation (FSC) between two-half maps reconstructed from the even and odd index of the tilted series and FSC between the final 3D map and the fitted structure model. The resolution for the former and latter criteria is evaluated at frequencies of 0.5 and 0.143, respectively.

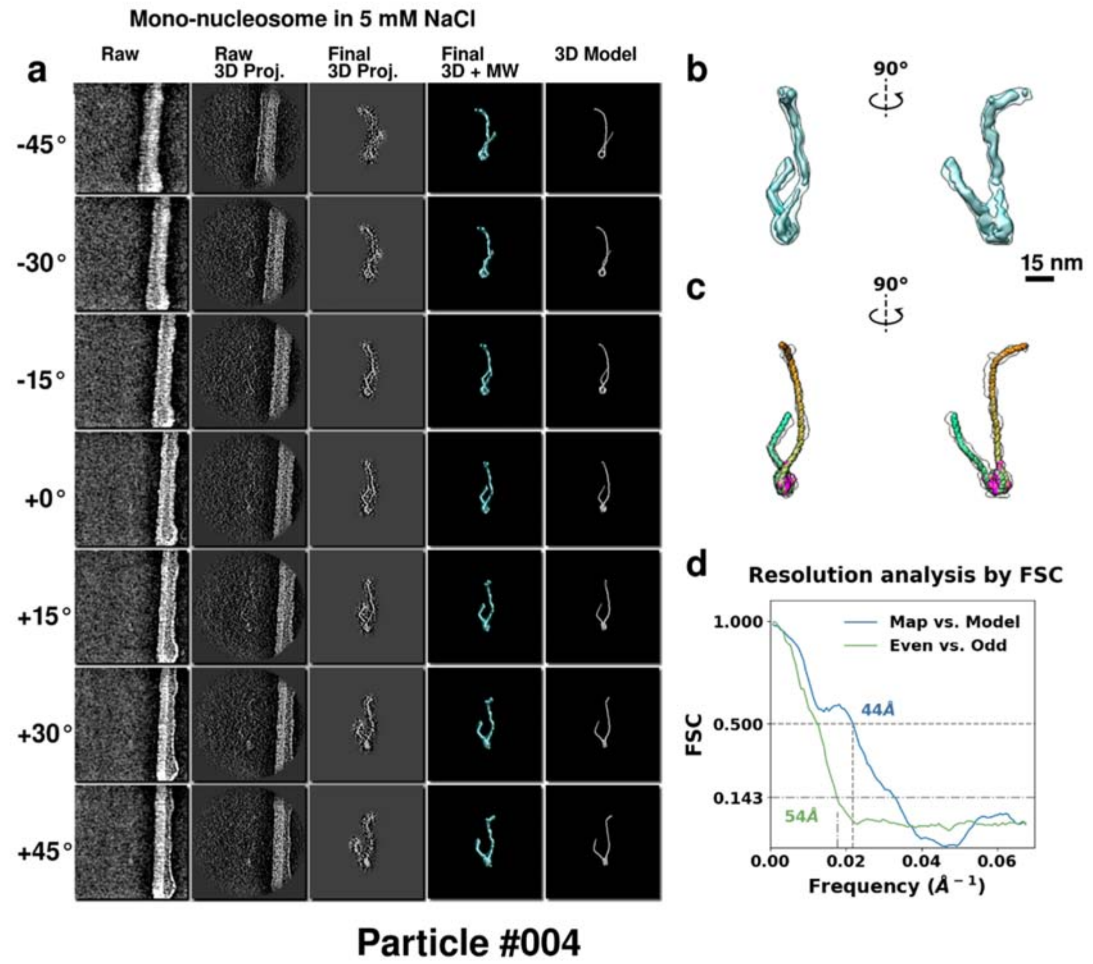

Supplementary Fig. 17. Cryo-ET 3D reconstruction of individual mono-nucleosome particle (index no. 4) in 5 mM NaCl. a, IPET 3D reconstruction of individual mono-nucleosome particles. The first column shows seven representative tilt images of an individual particle after CTF correction. Through alignment of the tilt images to a common center for 3D reconstruction via iterative refinement, the second and third columns display the 3D projections of the reconstruction before and after particle-shaped masking, respectively. The fourth column shows the final 3D reconstruction with missing wedge correction, and the fifth column presents the flexibly fitted model at the corresponding tilt angles. **b**, Zoomed-in view of the final 3D density map displayed in orthogonal views, shown at two contour levels. **c**, Superimposition of the high contour level map from (b) onto its flexibly fitted model. **d**, Resolution evaluation of the final 3D density map using two criteria: Fourier shell correlation (FSC) between two-half maps reconstructed from the even and odd index of the tilted series and FSC between the final 3D map and the fitted structure model. The resolution for the former and latter criteria is evaluated at frequencies of 0.5 and 0.143, respectively.

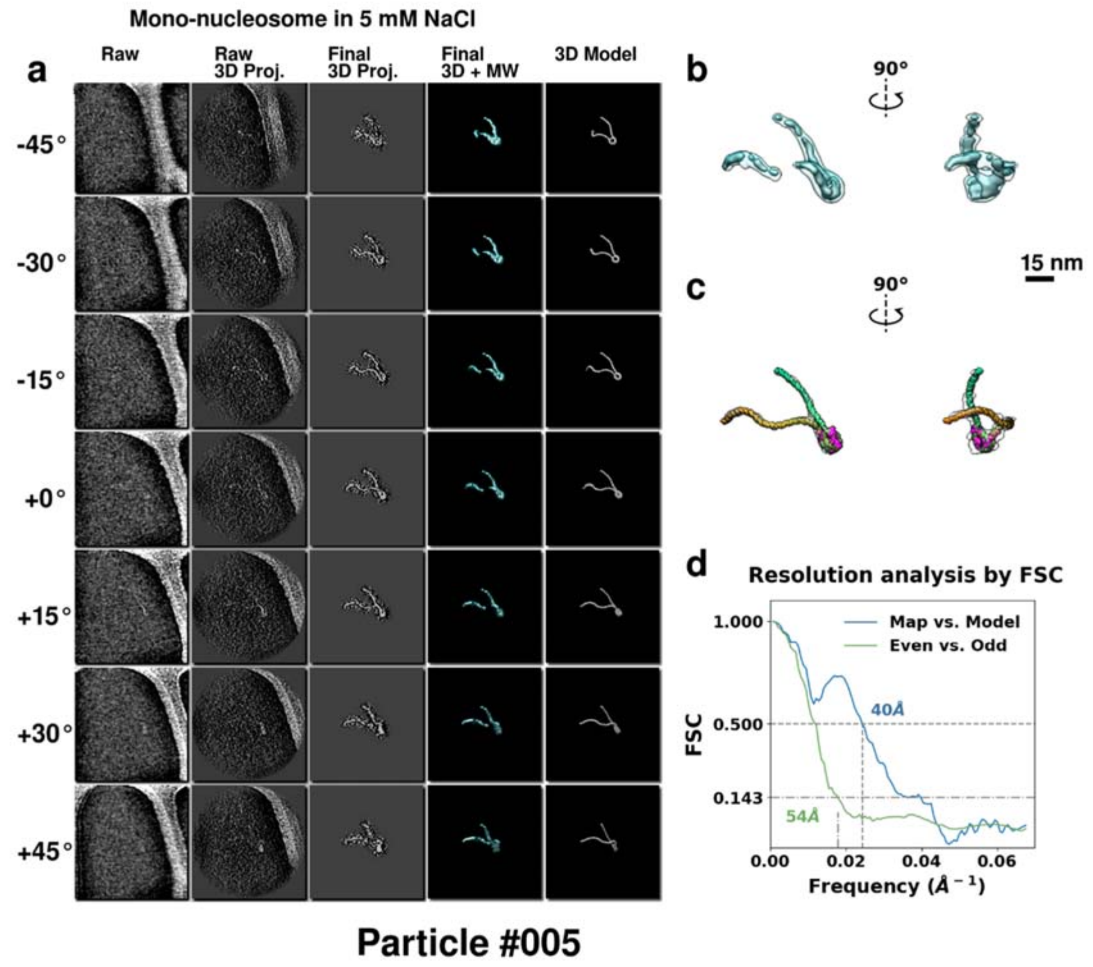

Supplementary Fig. 18. Cryo-ET 3D reconstruction of individual mono-nucleosome particle (index no. 5) in 5 mM NaCl. a, IPET 3D reconstruction of individual mono-nucleosome particles. The first column shows seven representative tilt images of an individual particle after CTF correction. Through alignment of the tilt images to a common center for 3D reconstruction via iterative refinement, the second and third columns display the 3D projections of the reconstruction before and after particle-shaped masking, respectively. The fourth column shows the final 3D reconstruction with missing wedge correction, and the fifth column presents the flexibly fitted model at the corresponding tilt angles. **b**, Zoomed-in view of the final 3D density map displayed in orthogonal views, shown at two contour levels. **c**, Superimposition of the high contour level map from (b) onto its flexibly fitted model. **d**, Resolution evaluation of the final 3D density map using two criteria: Fourier shell correlation (FSC) between two-half maps reconstructed from the even and odd index of the tilted series and FSC between the final 3D map and the fitted structure model. The resolution for the former and latter criteria is evaluated at frequencies of 0.5 and 0.143, respectively.

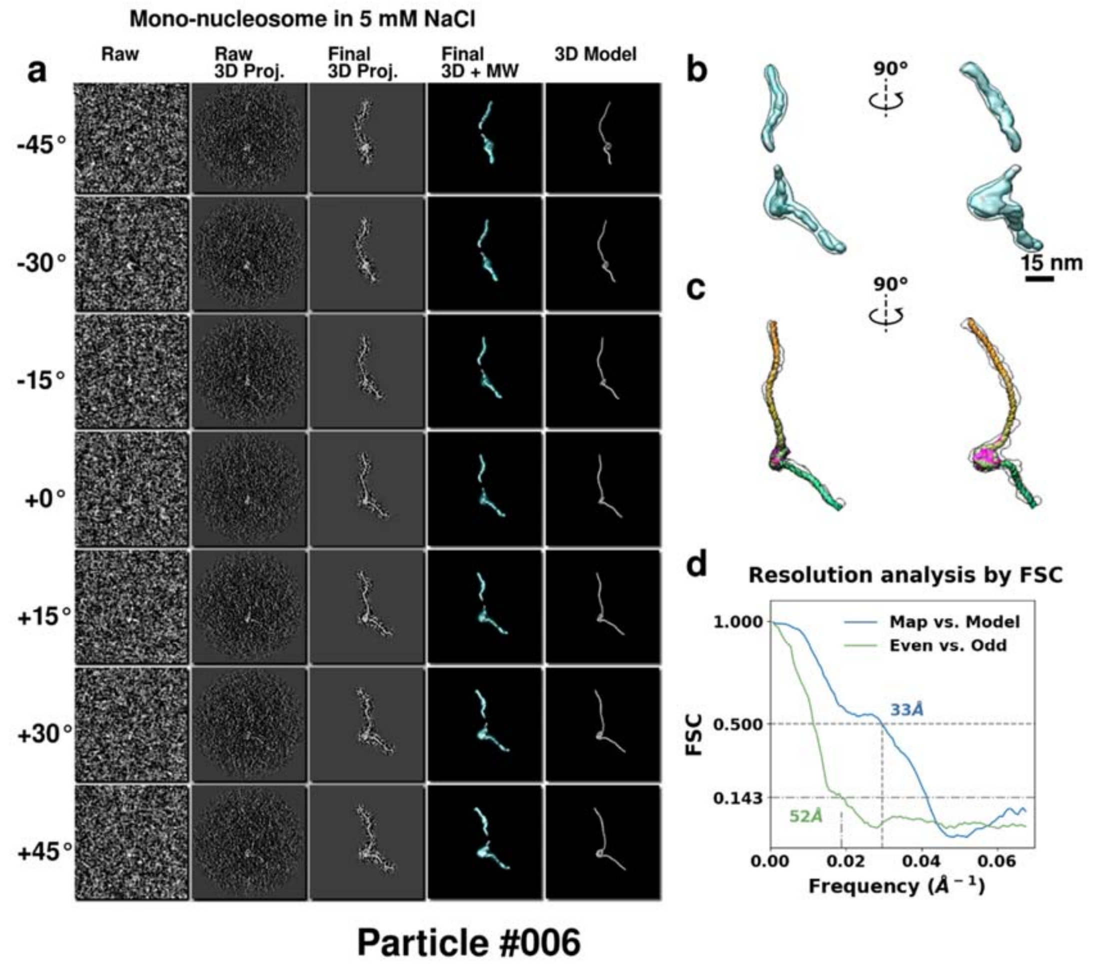

Supplementary Fig. 19. Cryo-ET 3D reconstruction of individual mono-nucleosome particle (index no. 6) in 5 mM NaCl. a, IPET 3D reconstruction of individual mono-nucleosome particles. The first column shows seven representative tilt images of an individual particle after CTF correction. Through alignment of the tilt images to a common center for 3D reconstruction via iterative refinement, the second and third columns display the 3D projections of the reconstruction before and after particle-shaped masking, respectively. The fourth column shows the final 3D reconstruction with missing wedge correction, and the fifth column presents the flexibly fitted model at the corresponding tilt angles. **b**, Zoomed-in view of the final 3D density map displayed in orthogonal views, shown at two contour levels. **c**, Superimposition of the high contour level map from (b) onto its flexibly fitted model. **d**, Resolution evaluation of the final 3D density map using two criteria: Fourier shell correlation (FSC) between two-half maps reconstructed from the even and odd index of the tilted series and FSC between the final 3D map and the fitted structure model. The resolution for the former and latter criteria is evaluated at frequencies of 0.5 and 0.143, respectively.

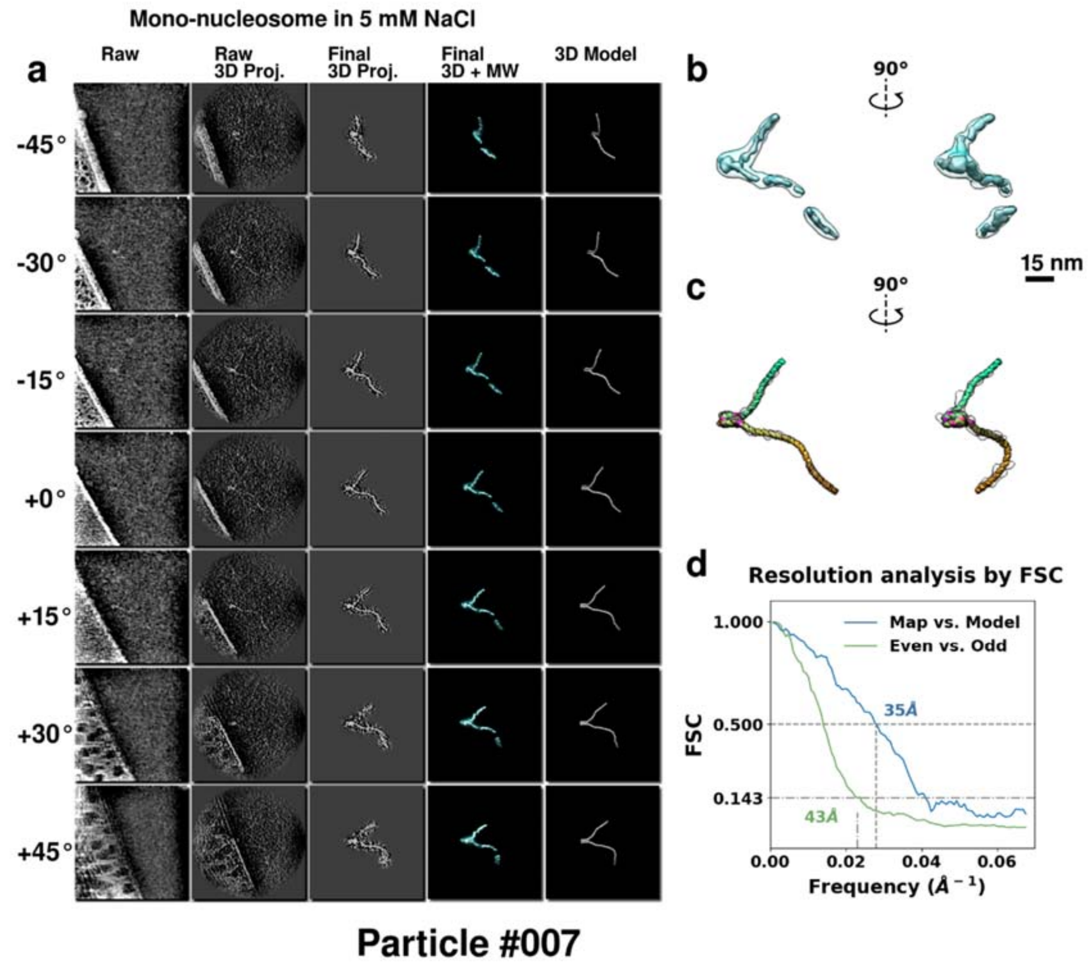

Supplementary Fig. 20. Cryo-ET 3D reconstruction of individual mono-nucleosome particle (index no. 7) in 5 mM NaCl. a, IPET 3D reconstruction of individual mono-nucleosome particles. The first column shows seven representative tilt images of an individual particle after CTF correction. Through alignment of the tilt images to a common center for 3D reconstruction via iterative refinement, the second and third columns display the 3D projections of the reconstruction before and after particle-shaped masking, respectively. The fourth column shows the final 3D reconstruction with missing wedge correction, and the fifth column presents the flexibly fitted model at the corresponding tilt angles. **b**, Zoomed-in view of the final 3D density map displayed in orthogonal views, shown at two contour levels. **c**, Superimposition of the high contour level map from (b) onto its flexibly fitted model. **d**, Resolution evaluation of the final 3D density map using two criteria: Fourier shell correlation (FSC) between two-half maps reconstructed from the even and odd index of the tilted series and FSC between the final 3D map and the fitted structure model. The resolution for the former and latter criteria is evaluated at frequencies of 0.5 and 0.143, respectively.

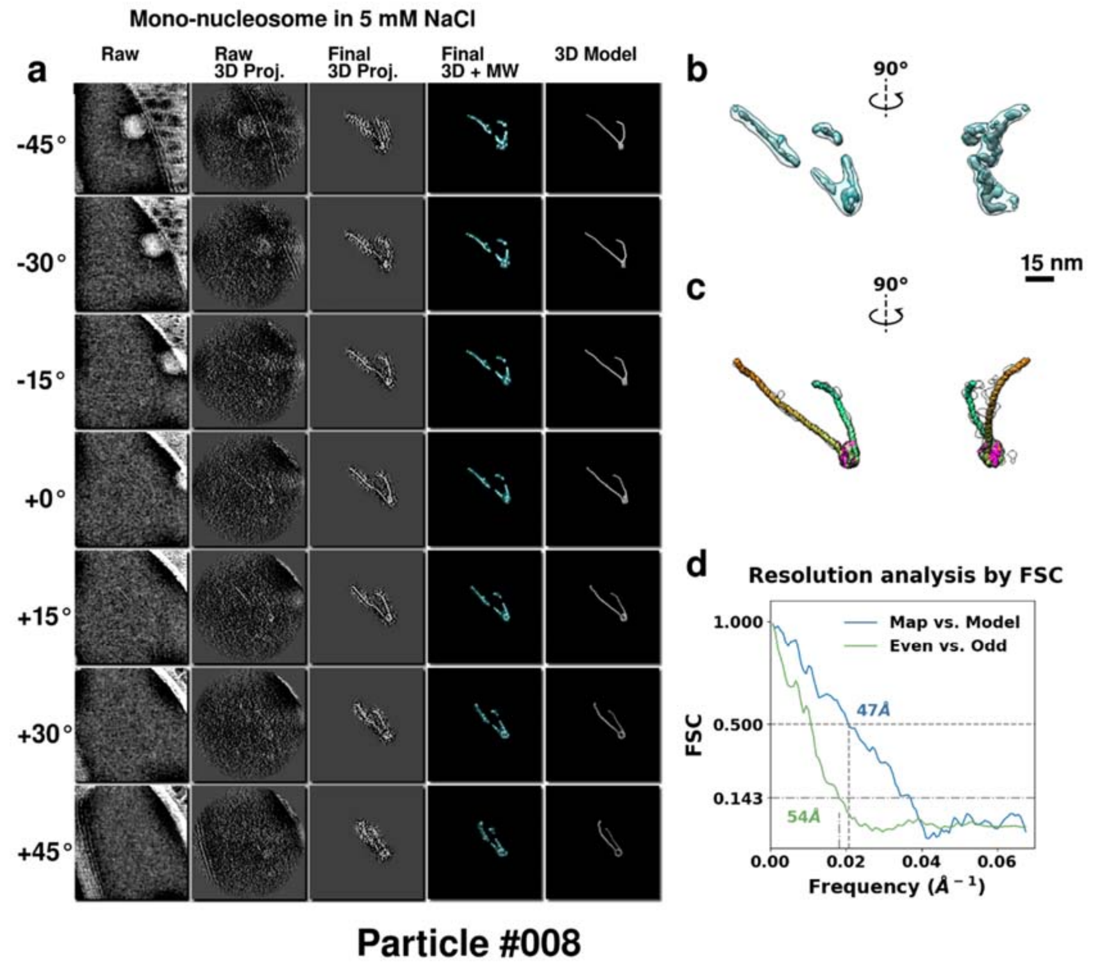

Supplementary Fig. 21. Cryo-ET 3D reconstruction of individual mono-nucleosome particle (index no. 8) in 5 mM NaCl. a, IPET 3D reconstruction of individual mono-nucleosome particles. The first column shows seven representative tilt images of an individual particle after CTF correction. Through alignment of the tilt images to a common center for 3D reconstruction via iterative refinement, the second and third columns display the 3D projections of the reconstruction before and after particle-shaped masking, respectively. The fourth column shows the final 3D reconstruction with missing wedge correction, and the fifth column presents the flexibly fitted model at the corresponding tilt angles. **b**, Zoomed-in view of the final 3D density map displayed in orthogonal views, shown at two contour levels. **c**, Superimposition of the high contour level map from (b) onto its flexibly fitted model. **d**, Resolution evaluation of the final 3D density map using two criteria: Fourier shell correlation (FSC) between two-half maps reconstructed from the even and odd index of the tilted series and FSC between the final 3D map and the fitted structure model. The resolution for the former and latter criteria is evaluated at frequencies of 0.5 and 0.143, respectively.

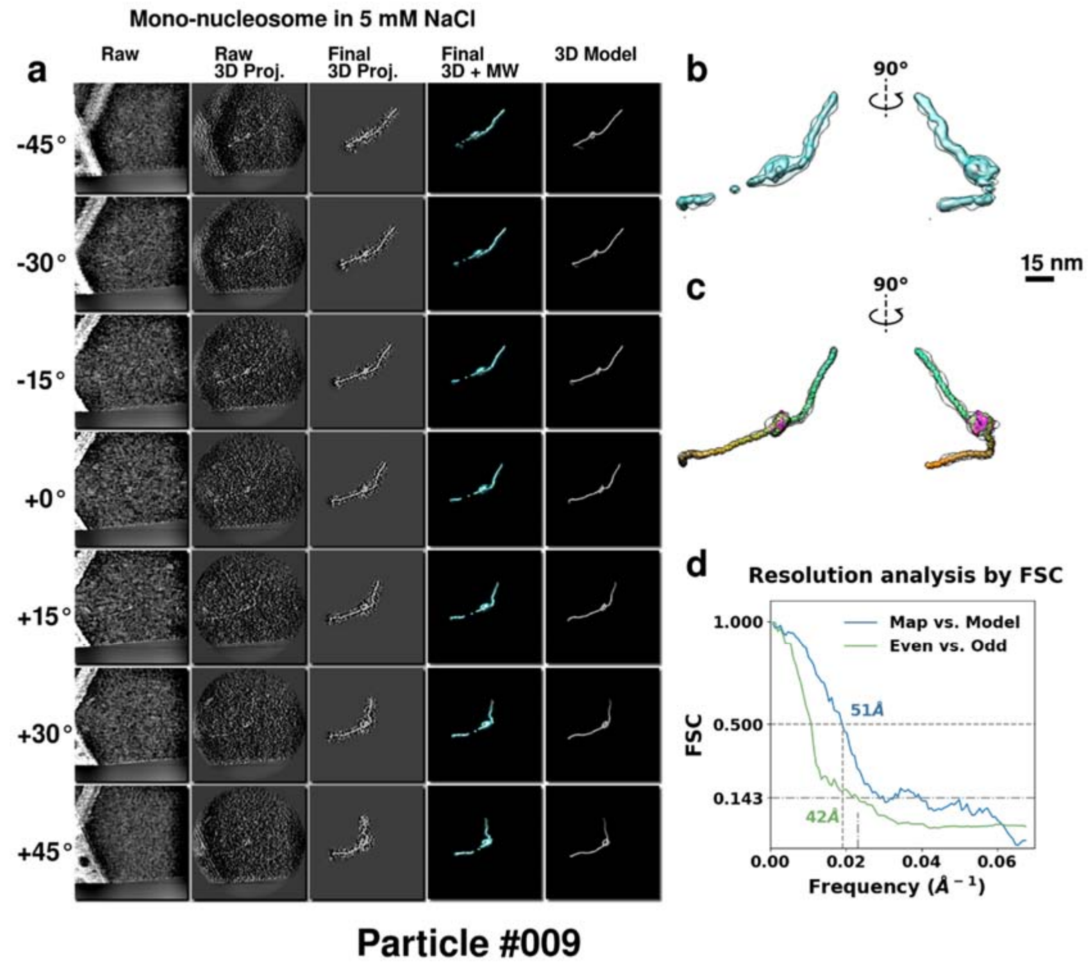

Supplementary Fig. 22. Cryo-ET 3D reconstruction of individual mono-nucleosome particle (index no. 9) in 5 mM NaCl. a, IPET 3D reconstruction of individual mono-nucleosome particles. The first column shows seven representative tilt images of an individual particle after CTF correction. Through alignment of the tilt images to a common center for 3D reconstruction via iterative refinement, the second and third columns display the 3D projections of the reconstruction before and after particle-shaped masking, respectively. The fourth column shows the final 3D reconstruction with missing wedge correction, and the fifth column presents the flexibly fitted model at the corresponding tilt angles. **b**, Zoomed-in view of the final 3D density map displayed in orthogonal views, shown at two contour levels. **c**, Superimposition of the high contour level map from (b) onto its flexibly fitted model. **d**, Resolution evaluation of the final 3D density map using two criteria: Fourier shell correlation (FSC) between two-half maps reconstructed from the even and odd index of the tilted series and FSC between the final 3D map and the fitted structure model. The resolution for the former and latter criteria is evaluated at frequencies of 0.5 and 0.143, respectively.

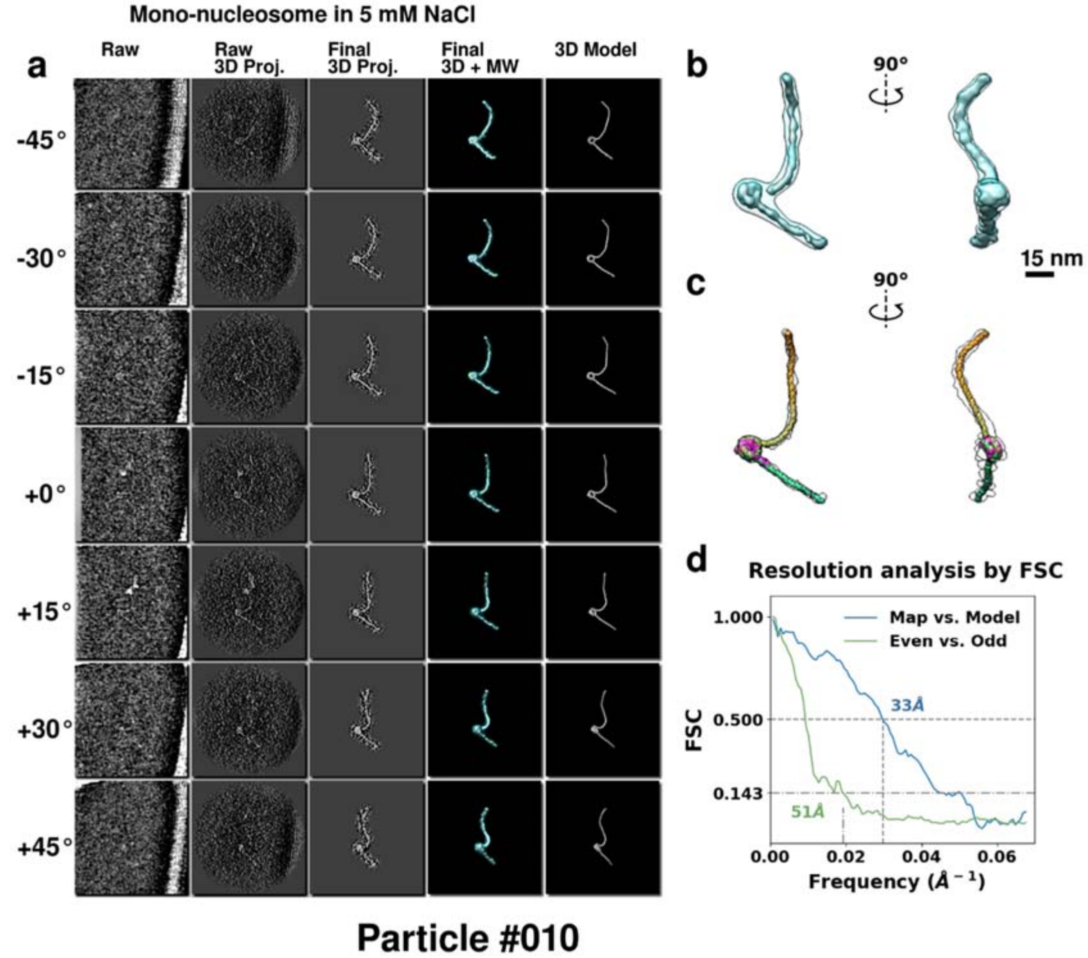

Supplementary Fig. 23. Cryo-ET 3D reconstruction of individual mono-nucleosome particle (index no. 10) in 5 mM NaCl. a, IPET 3D reconstruction of individual mono-nucleosome particles. The first column shows seven representative tilt images of an individual particle after CTF correction. Through alignment of the tilt images to a common center for 3D reconstruction via iterative refinement, the second and third columns display the 3D projections of the reconstruction before and after particle-shaped masking, respectively. The fourth column shows the final 3D reconstruction with missing wedge correction, and the fifth column presents the flexibly fitted model at the corresponding tilt angles. **b**, Zoomed-in view of the final 3D density map displayed in orthogonal views, shown at two contour levels. **c**, Superimposition of the high contour level map from (b) onto its flexibly fitted model. **d**, Resolution evaluation of the final 3D density map using two criteria: Fourier shell correlation (FSC) between two-half maps reconstructed from the even and odd index of the tilted series and FSC between the final 3D map and the fitted structure model. The resolution for the former and latter criteria is evaluated at frequencies of 0.5 and 0.143, respectively.

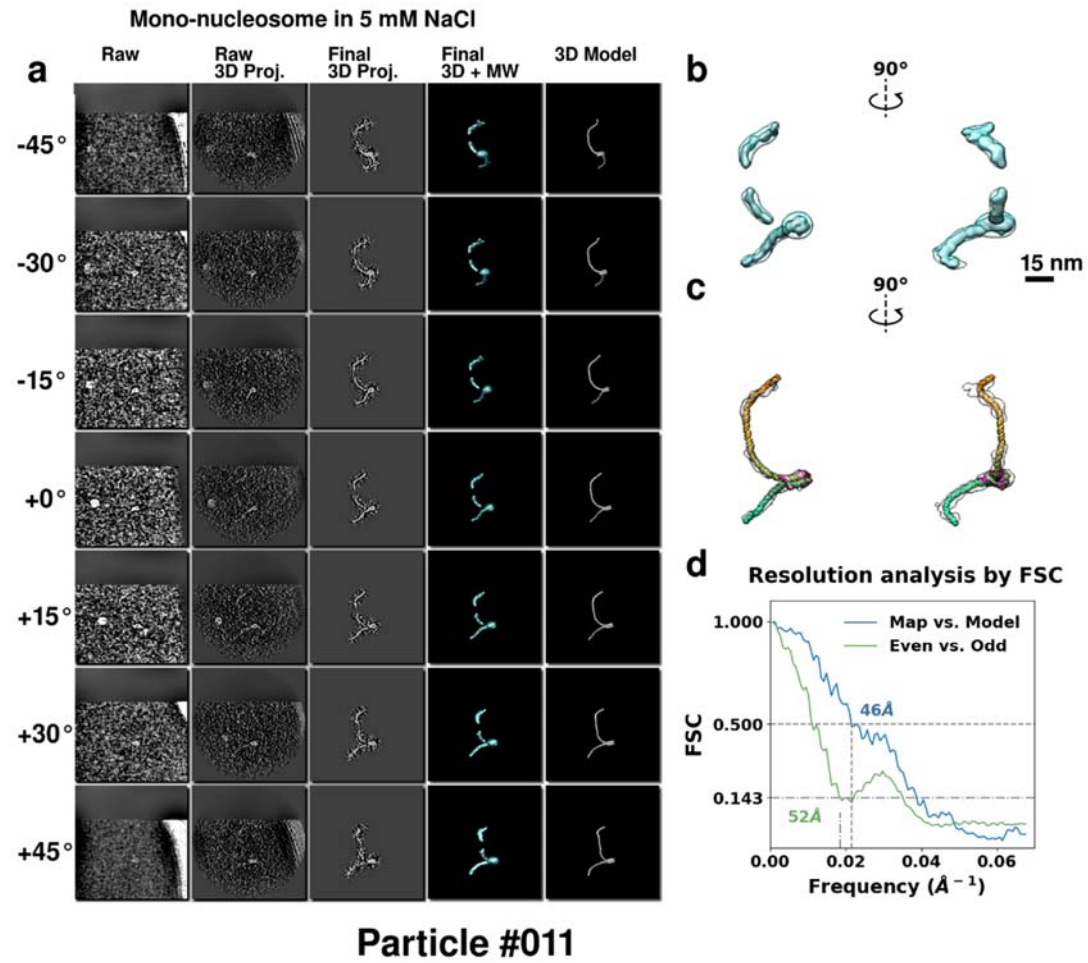

Supplementary Fig. 24. Cryo-ET 3D reconstruction of individual mono-nucleosome particle (index no. 11) in 5 mM NaCl. a, IPET 3D reconstruction of individual mono-nucleosome particles. The first column shows seven representative tilt images of an individual particle after CTF correction. Through alignment of the tilt images to a common center for 3D reconstruction via iterative refinement, the second and third columns display the 3D projections of the reconstruction before and after particle-shaped masking, respectively. The fourth column shows the final 3D reconstruction with missing wedge correction, and the fifth column presents the flexibly fitted model at the corresponding tilt angles. **b**, Zoomed-in view of the final 3D density map displayed in orthogonal views, shown at two contour levels. **c**, Superimposition of the high contour level map from (b) onto its flexibly fitted model. **d**, Resolution evaluation of the final 3D density map using two criteria: Fourier shell correlation (FSC) between two-half maps reconstructed from the even and odd index of the tilted series and FSC between the final 3D map and the fitted structure model. The resolution for the former and latter criteria is evaluated at frequencies of 0.5 and 0.143, respectively.

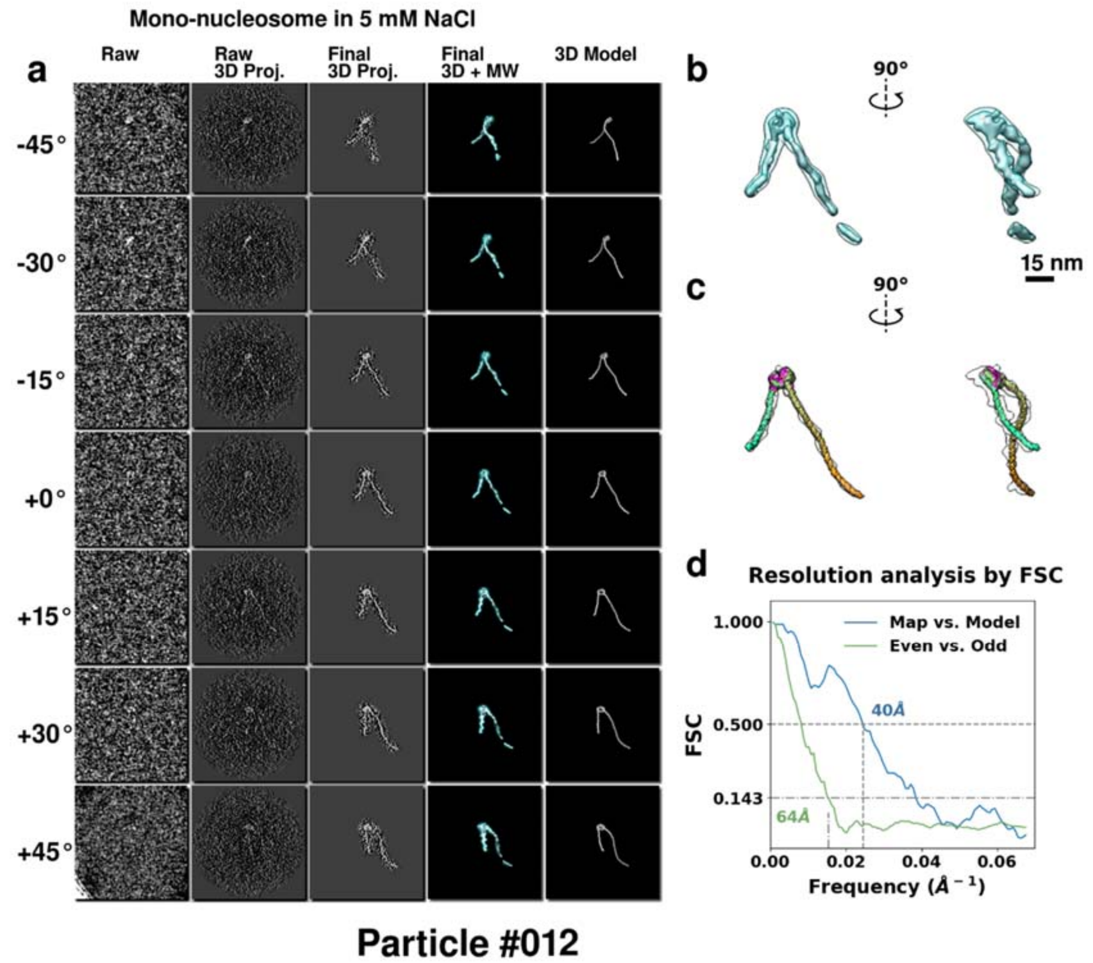

Supplementary Fig. 25. Cryo-ET 3D reconstruction of individual mono-nucleosome particle (index no. 12) in 5 mM NaCl. a, IPET 3D reconstruction of individual mono-nucleosome particles. The first column shows seven representative tilt images of an individual particle after CTF correction. Through alignment of the tilt images to a common center for 3D reconstruction via iterative refinement, the second and third columns display the 3D projections of the reconstruction before and after particle-shaped masking, respectively. The fourth column shows the final 3D reconstruction with missing wedge correction, and the fifth column presents the flexibly fitted model at the corresponding tilt angles. **b**, Zoomed-in view of the final 3D density map displayed in orthogonal views, shown at two contour levels. **c**, Superimposition of the high contour level map from (b) onto its flexibly fitted model. **d**, Resolution evaluation of the final 3D density map using two criteria: Fourier shell correlation (FSC) between two-half maps reconstructed from the even and odd index of the tilted series and FSC between the final 3D map and the fitted structure model. The resolution for the former and latter criteria is evaluated at frequencies of 0.5 and 0.143, respectively.

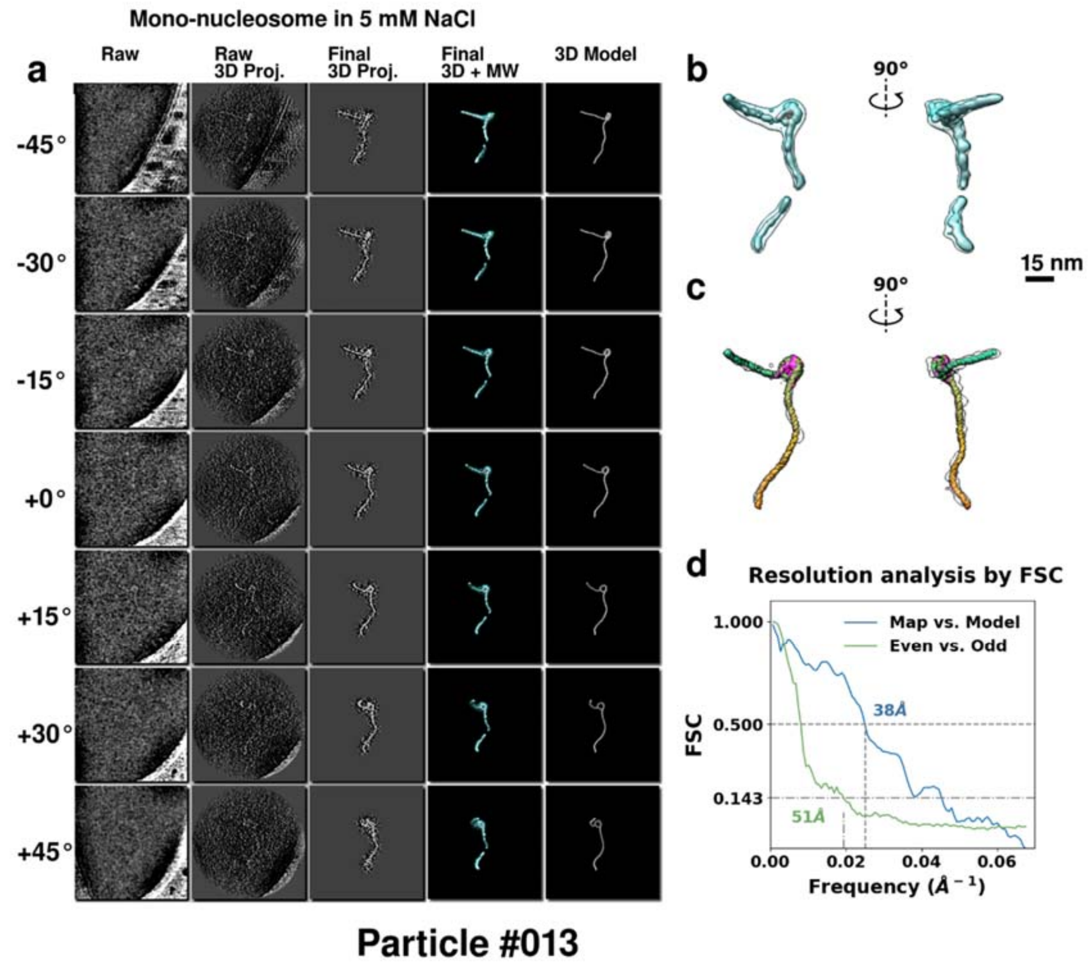

Supplementary Fig. 26. Cryo-ET 3D reconstruction of individual mono-nucleosome particle (index no. 13) in 5 mM NaCl. a, IPET 3D reconstruction of individual mono-nucleosome particles. The first column shows seven representative tilt images of an individual particle after CTF correction. Through alignment of the tilt images to a common center for 3D reconstruction via iterative refinement, the second and third columns display the 3D projections of the reconstruction before and after particle-shaped masking, respectively. The fourth column shows the final 3D reconstruction with missing wedge correction, and the fifth column presents the flexibly fitted model at the corresponding tilt angles. **b**, Zoomed-in view of the final 3D density map displayed in orthogonal views, shown at two contour levels. **c**, Superimposition of the high contour level map from (b) onto its flexibly fitted model. **d**, Resolution evaluation of the final 3D density map using two criteria: Fourier shell correlation (FSC) between two-half maps reconstructed from the even and odd index of the tilted series and FSC between the final 3D map and the fitted structure model. The resolution for the former and latter criteria is evaluated at frequencies of 0.5 and 0.143, respectively.

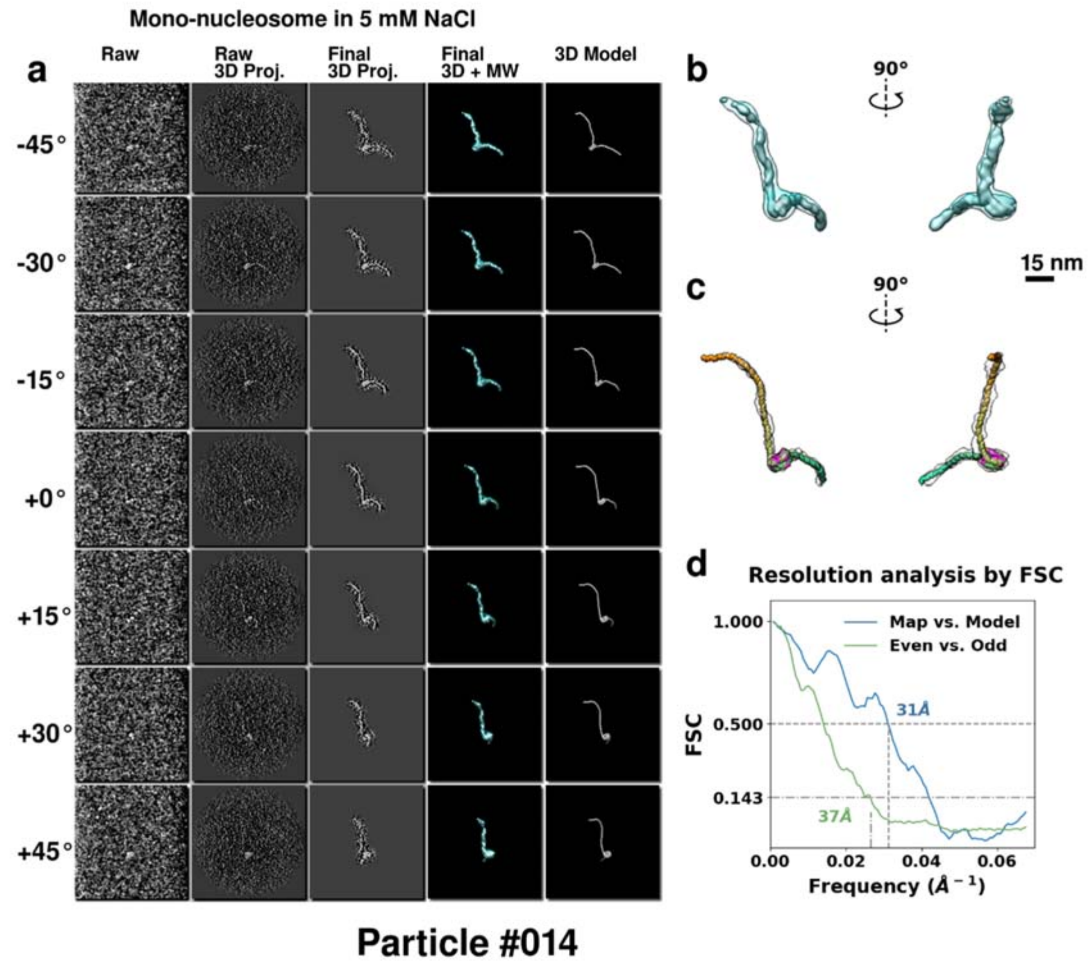

Supplementary Fig. 27. Cryo-ET 3D reconstruction of individual mono-nucleosome particle (index no. 14) in 5 mM NaCl. a, IPET 3D reconstruction of individual mono-nucleosome particles. The first column shows seven representative tilt images of an individual particle after CTF correction. Through alignment of the tilt images to a common center for 3D reconstruction via iterative refinement, the second and third columns display the 3D projections of the reconstruction before and after particle-shaped masking, respectively. The fourth column shows the final 3D reconstruction with missing wedge correction, and the fifth column presents the flexibly fitted model at the corresponding tilt angles. **b**, Zoomed-in view of the final 3D density map displayed in orthogonal views, shown at two contour levels. **c**, Superimposition of the high contour level map from (b) onto its flexibly fitted model. **d**, Resolution evaluation of the final 3D density map using two criteria: Fourier shell correlation (FSC) between two-half maps reconstructed from the even and odd index of the tilted series and FSC between the final 3D map and the fitted structure model. The resolution for the former and latter criteria is evaluated at frequencies of 0.5 and 0.143, respectively.

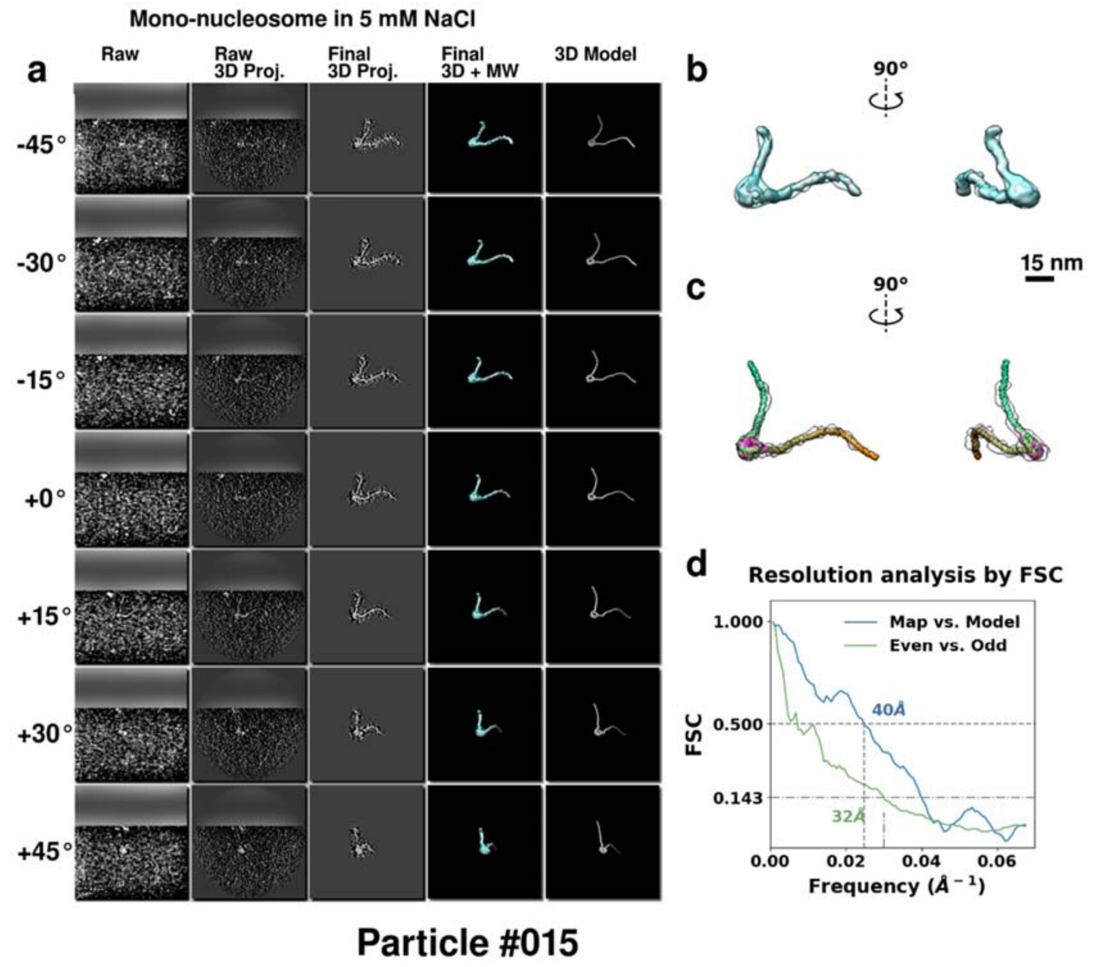

Supplementary Fig. 28. Cryo-ET 3D reconstruction of individual mono-nucleosome particle (index no. 15) in 5 mM NaCl. **a**, IPET 3D reconstruction of individual mono-nucleosome particles. The first column shows seven representative tilt images of an individual particle after CTF correction. Through alignment of the tilt images to a common center for 3D reconstruction via iterative refinement, the second and third columns display the 3D projections of the reconstruction before and after particle-shaped masking, respectively. The fourth column shows the final 3D reconstruction with missing wedge correction, and the fifth column presents the flexibly fitted model at the corresponding tilt angles. **b**, Zoomed-in view of the final 3D density map displayed in orthogonal views, shown at two contour levels. **c**, Superimposition of the high contour level map from (b) onto its flexibly fitted model. **d**, Resolution evaluation of the final 3D density map using two criteria: Fourier shell correlation (FSC) between two-half maps reconstructed from the even and odd index of the tilted series and FSC between the final 3D map and the fitted structure model. The resolution for the former and latter criteria is evaluated at frequencies of 0.5 and 0.143, respectively.

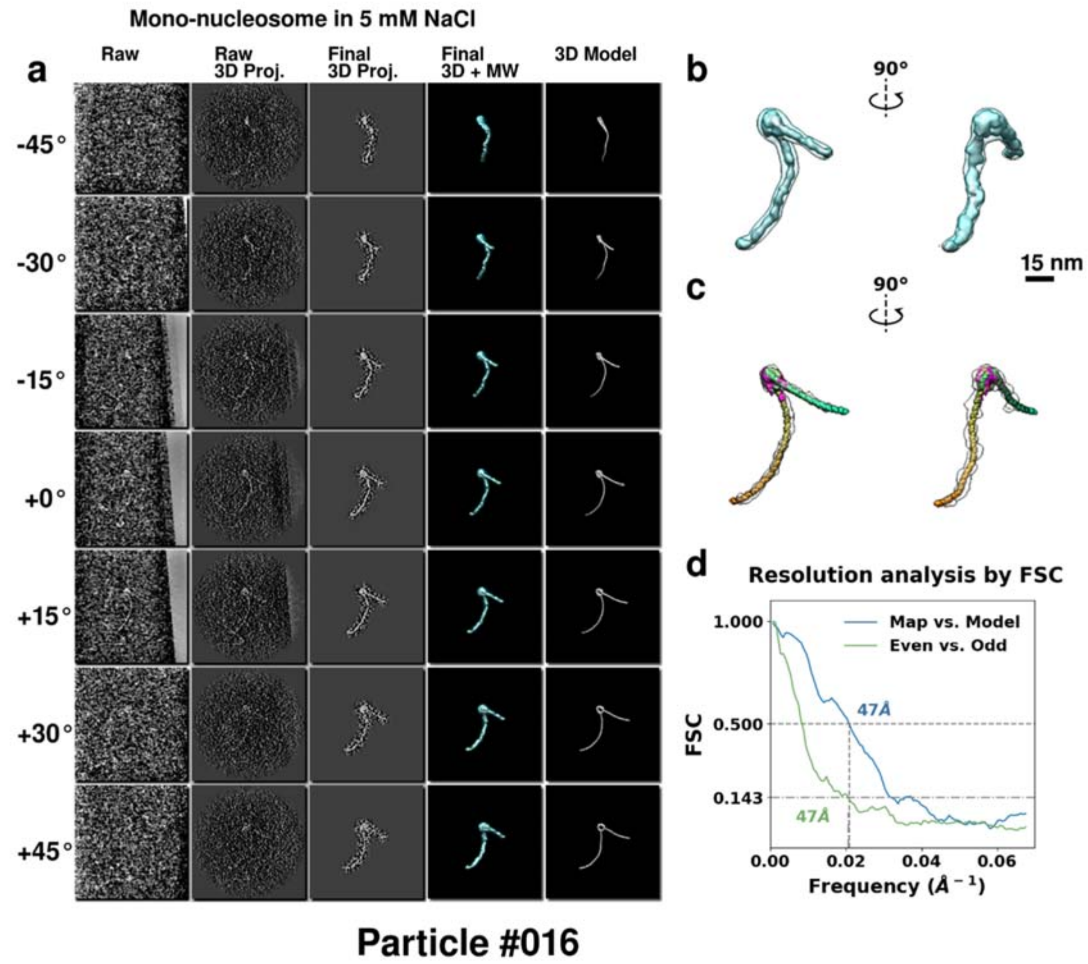

Supplementary Fig. 29. Cryo-ET 3D reconstruction of individual mono-nucleosome particle (index no. 16) in 5 mM NaCl. a, IPET 3D reconstruction of individual mono-nucleosome particles. The first column shows seven representative tilt images of an individual particle after CTF correction. Through alignment of the tilt images to a common center for 3D reconstruction via iterative refinement, the second and third columns display the 3D projections of the reconstruction before and after particle-shaped masking, respectively. The fourth column shows the final 3D reconstruction with missing wedge correction, and the fifth column presents the flexibly fitted model at the corresponding tilt angles. **b**, Zoomed-in view of the final 3D density map displayed in orthogonal views, shown at two contour levels. **c**, Superimposition of the high contour level map from (b) onto its flexibly fitted model. **d**, Resolution evaluation of the final 3D density map using two criteria: Fourier shell correlation (FSC) between two-half maps reconstructed from the even and odd index of the tilted series and FSC between the final 3D map and the fitted structure model. The resolution for the former and latter criteria is evaluated at frequencies of 0.5 and 0.143, respectively.

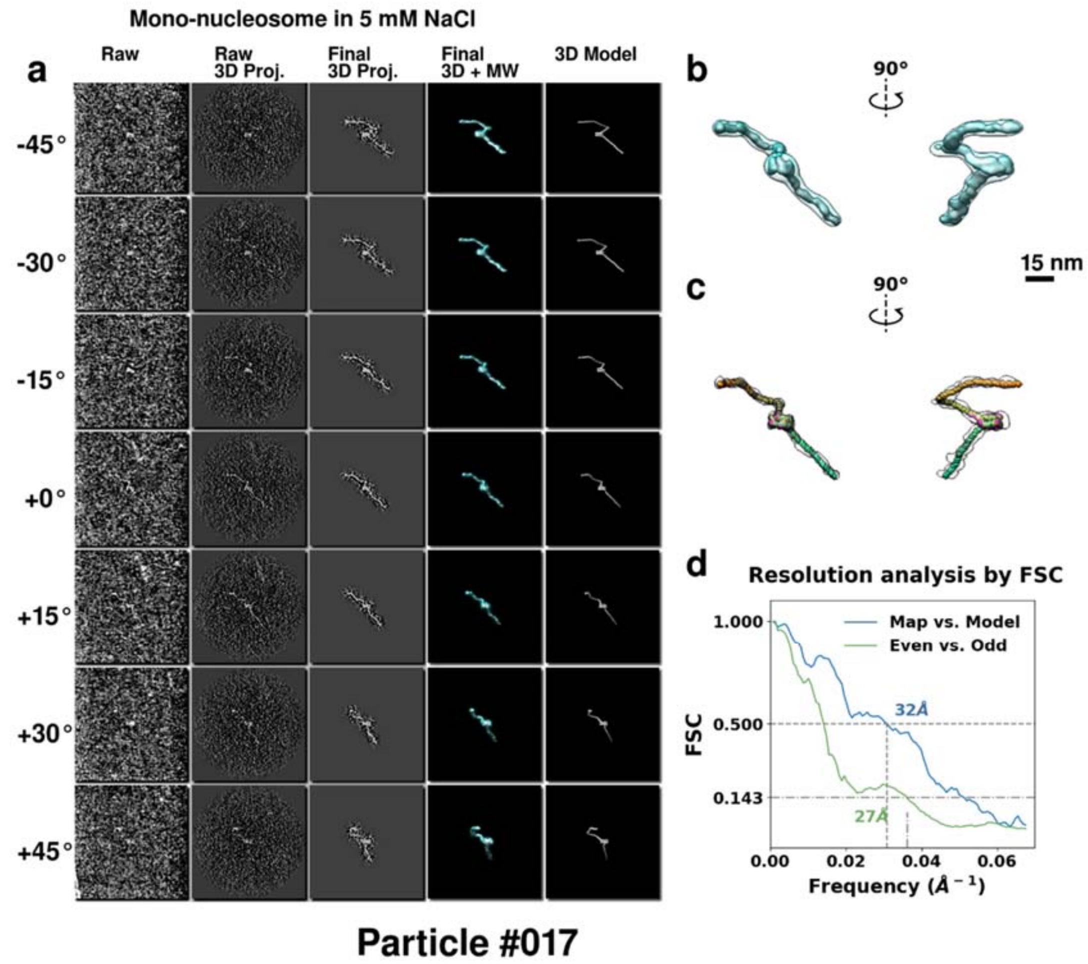

Supplementary Fig. 30. Cryo-ET 3D reconstruction of individual mono-nucleosome particle (index no. 17) in 5 mM NaCl. a, IPET 3D reconstruction of individual mono-nucleosome particles. The first column shows seven representative tilt images of an individual particle after CTF correction. Through alignment of the tilt images to a common center for 3D reconstruction via iterative refinement, the second and third columns display the 3D projections of the reconstruction before and after particle-shaped masking, respectively. The fourth column shows the final 3D reconstruction with missing wedge correction, and the fifth column presents the flexibly fitted model at the corresponding tilt angles. **b**, Zoomed-in view of the final 3D density map displayed in orthogonal views, shown at two contour levels. **c**, Superimposition of the high contour level map from (b) onto its flexibly fitted model. **d**, Resolution evaluation of the final 3D density map using two criteria: Fourier shell correlation (FSC) between two-half maps reconstructed from the even and odd index of the tilted series and FSC between the final 3D map and the fitted structure model. The resolution for the former and latter criteria is evaluated at frequencies of 0.5 and 0.143, respectively.

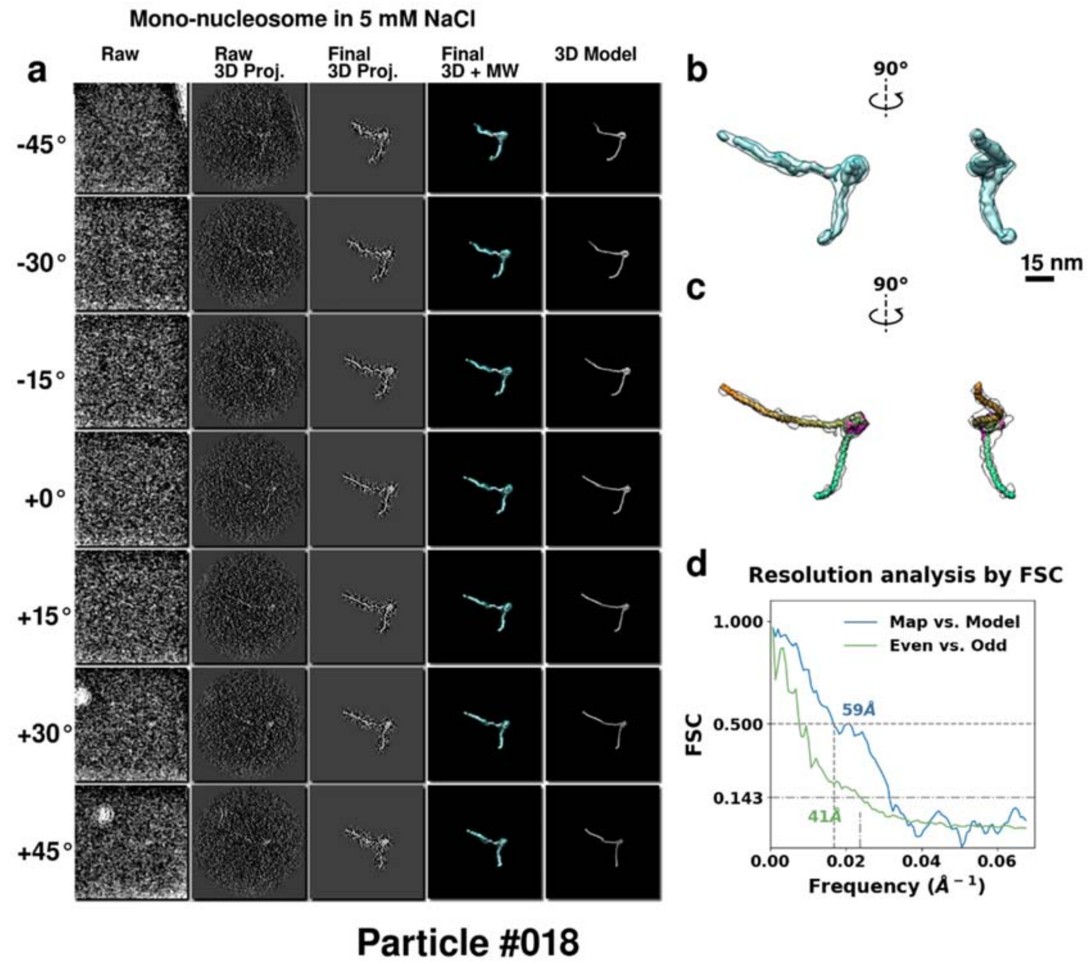

Supplementary Fig. 31. Cryo-ET 3D reconstruction of individual mono-nucleosome particle (index no. 18) in 5 mM NaCl. a, IPET 3D reconstruction of individual mono-nucleosome particles. The first column shows seven representative tilt images of an individual particle after CTF correction. Through alignment of the tilt images to a common center for 3D reconstruction via iterative refinement, the second and third columns display the 3D projections of the reconstruction before and after particle-shaped masking, respectively. The fourth column shows the final 3D reconstruction with missing wedge correction, and the fifth column presents the flexibly fitted model at the corresponding tilt angles. **b**, Zoomed-in view of the final 3D density map displayed in orthogonal views, shown at two contour levels. **c**, Superimposition of the high contour level map from (b) onto its flexibly fitted model. **d**, Resolution evaluation of the final 3D density map using two criteria: Fourier shell correlation (FSC) between two-half maps reconstructed from the even and odd index of the tilted series and FSC between the final 3D map and the fitted structure model. The resolution for the former and latter criteria is evaluated at frequencies of 0.5 and 0.143, respectively.

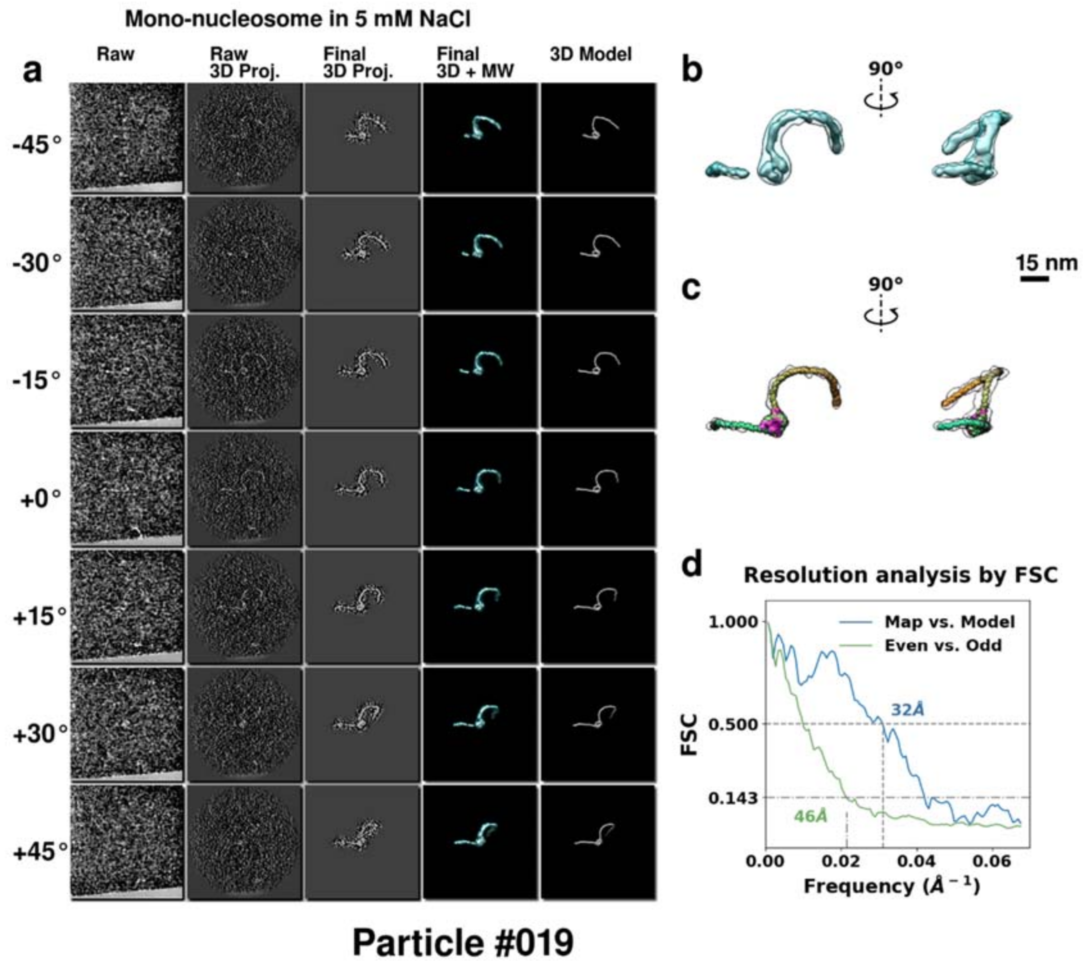

Supplementary Fig. 32. Cryo-ET 3D reconstruction of individual mono-nucleosome particle (index no. 19) in 5 mM NaCl. a, IPET 3D reconstruction of individual mono-nucleosome particles. The first column shows seven representative tilt images of an individual particle after CTF correction. Through alignment of the tilt images to a common center for 3D reconstruction via iterative refinement, the second and third columns display the 3D projections of the reconstruction before and after particle-shaped masking, respectively. The fourth column shows the final 3D reconstruction with missing wedge correction, and the fifth column presents the flexibly fitted model at the corresponding tilt angles. **b**, Zoomed-in view of the final 3D density map displayed in orthogonal views, shown at two contour levels. **c**, Superimposition of the high contour level map from (b) onto its flexibly fitted model. **d**, Resolution evaluation of the final 3D density map using two criteria: Fourier shell correlation (FSC) between two-half maps reconstructed from the even and odd index of the tilted series and FSC between the final 3D map and the fitted structure model. The resolution for the former and latter criteria is evaluated at frequencies of 0.5 and 0.143, respectively.

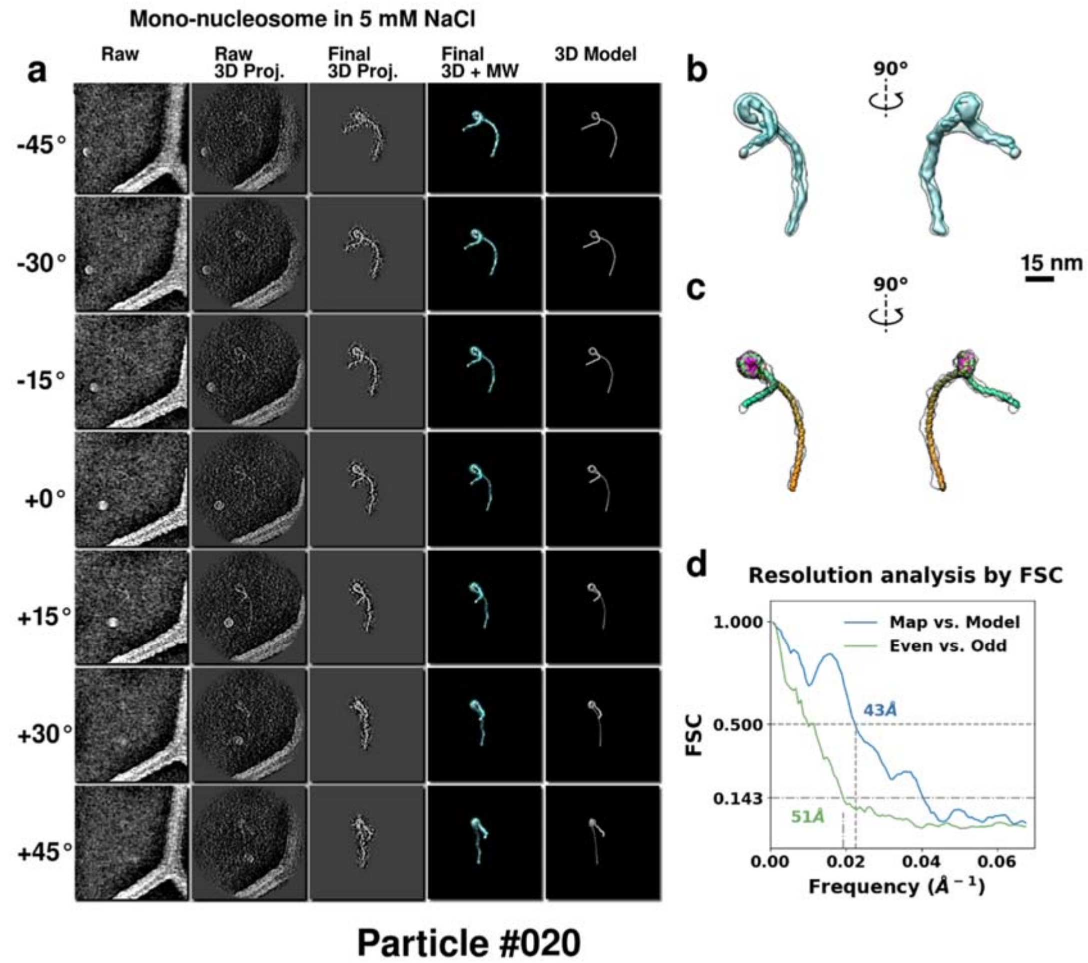

Supplementary Fig. 33. Cryo-ET 3D reconstruction of individual mono-nucleosome particle (index no. 20) in 5 mM NaCl. a, IPET 3D reconstruction of individual mono-nucleosome particles. The first column shows seven representative tilt images of an individual particle after CTF correction. Through alignment of the tilt images to a common center for 3D reconstruction via iterative refinement, the second and third columns display the 3D projections of the reconstruction before and after particle-shaped masking, respectively. The fourth column shows the final 3D reconstruction with missing wedge correction, and the fifth column presents the flexibly fitted model at the corresponding tilt angles. **b**, Zoomed-in view of the final 3D density map displayed in orthogonal views, shown at two contour levels. **c**, Superimposition of the high contour level map from (b) onto its flexibly fitted model. **d**, Resolution evaluation of the final 3D density map using two criteria: Fourier shell correlation (FSC) between two-half maps reconstructed from the even and odd index of the tilted series and FSC between the final 3D map and the fitted structure model. The resolution for the former and latter criteria is evaluated at frequencies of 0.5 and 0.143, respectively.

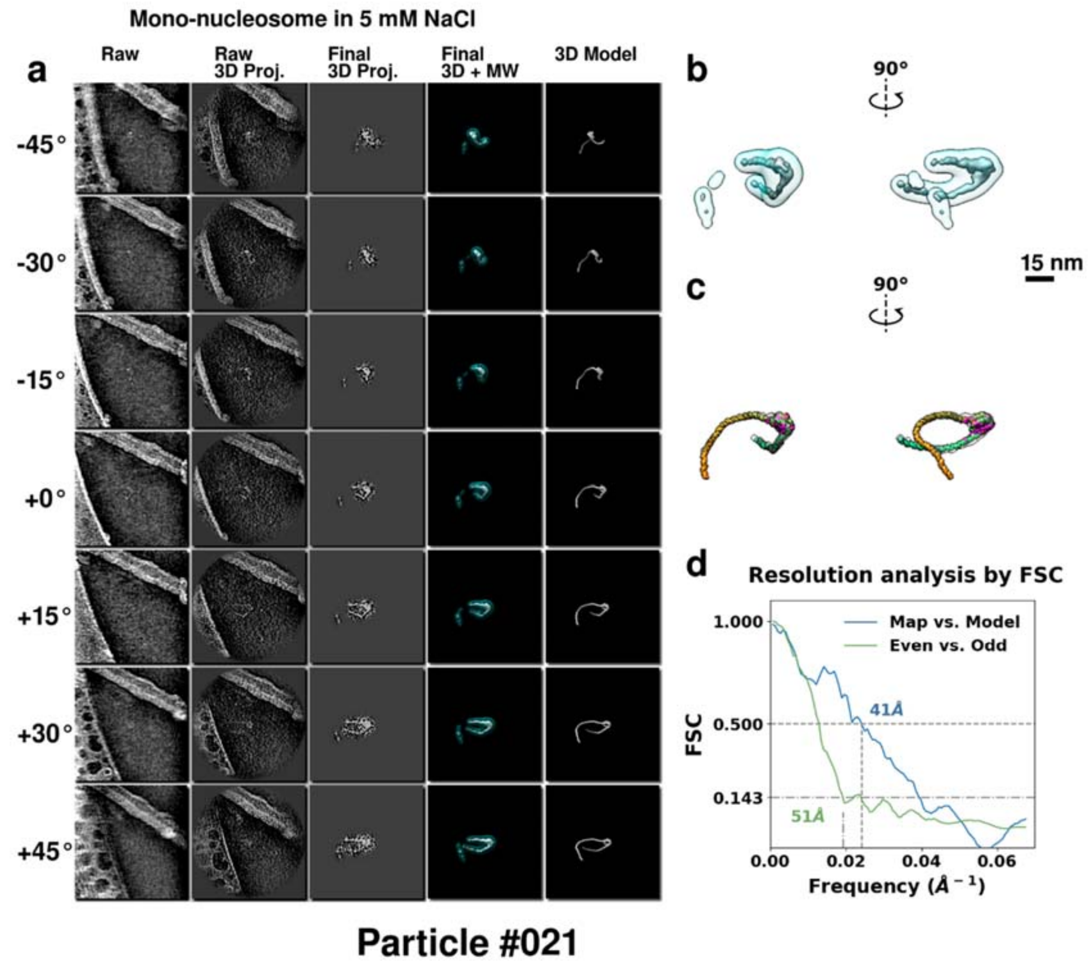

Supplementary Fig. 34. Cryo-ET 3D reconstruction of individual mono-nucleosome particle (index no. 21) in 5 mM NaCl. a, IPET 3D reconstruction of individual mono-nucleosome particles. The first column shows seven representative tilt images of an individual particle after CTF correction. Through alignment of the tilt images to a common center for 3D reconstruction via iterative refinement, the second and third columns display the 3D projections of the reconstruction before and after particle-shaped masking, respectively. The fourth column shows the final 3D reconstruction with missing wedge correction, and the fifth column presents the flexibly fitted model at the corresponding tilt angles. **b**, Zoomed-in view of the final 3D density map displayed in orthogonal views, shown at two contour levels. **c**, Superimposition of the high contour level map from (b) onto its flexibly fitted model. **d**, Resolution evaluation of the final 3D density map using two criteria: Fourier shell correlation (FSC) between two-half maps reconstructed from the even and odd index of the tilted series and FSC between the final 3D map and the fitted structure model. The resolution for the former and latter criteria is evaluated at frequencies of 0.5 and 0.143, respectively.

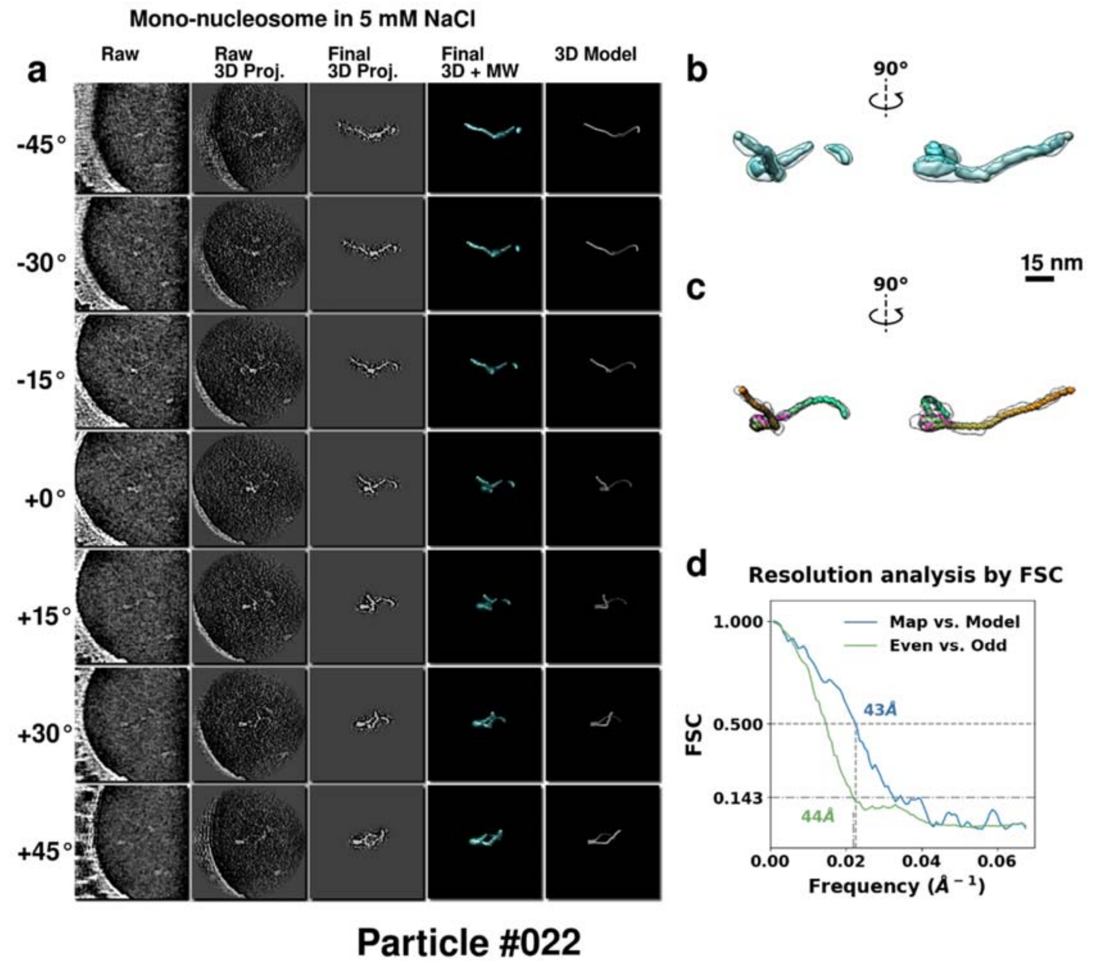

Supplementary Fig. 35. Cryo-ET 3D reconstruction of individual mono-nucleosome particle (index no. 22) in 5 mM NaCl. a, IPET 3D reconstruction of individual mono-nucleosome particles. The first column shows seven representative tilt images of an individual particle after CTF correction. Through alignment of the tilt images to a common center for 3D reconstruction via iterative refinement, the second and third columns display the 3D projections of the reconstruction before and after particle-shaped masking, respectively. The fourth column shows the final 3D reconstruction with missing wedge correction, and the fifth column presents the flexibly fitted model at the corresponding tilt angles. **b**, Zoomed-in view of the final 3D density map displayed in orthogonal views, shown at two contour levels. **c**, Superimposition of the high contour level map from (b) onto its flexibly fitted model. **d**, Resolution evaluation of the final 3D density map using two criteria: Fourier shell correlation (FSC) between two-half maps reconstructed from the even and odd index of the tilted series and FSC between the final 3D map and the fitted structure model. The resolution for the former and latter criteria is evaluated at frequencies of 0.5 and 0.143, respectively.

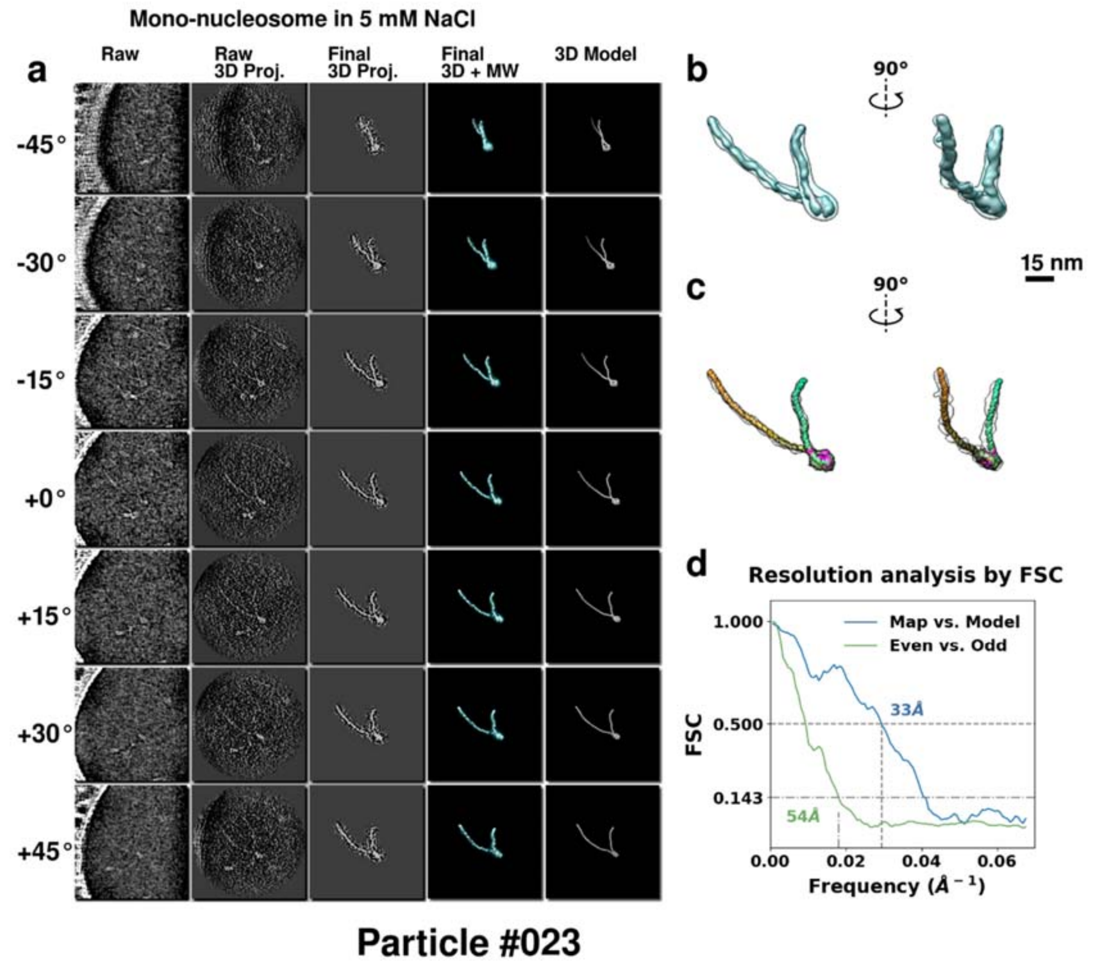

Supplementary Fig. 36. Cryo-ET 3D reconstruction of individual mono-nucleosome particle (index no. 23) in 5 mM NaCl. a, IPET 3D reconstruction of individual mono-nucleosome particles. The first column shows seven representative tilt images of an individual particle after CTF correction. Through alignment of the tilt images to a common center for 3D reconstruction via iterative refinement, the second and third columns display the 3D projections of the reconstruction before and after particle-shaped masking, respectively. The fourth column shows the final 3D reconstruction with missing wedge correction, and the fifth column presents the flexibly fitted model at the corresponding tilt angles. **b**, Zoomed-in view of the final 3D density map displayed in orthogonal views, shown at two contour levels. **c**, Superimposition of the high contour level map from (b) onto its flexibly fitted model. **d**, Resolution evaluation of the final 3D density map using two criteria: Fourier shell correlation (FSC) between two-half maps reconstructed from the even and odd index of the tilted series and FSC between the final 3D map and the fitted structure model. The resolution for the former and latter criteria is evaluated at frequencies of 0.5 and 0.143, respectively.

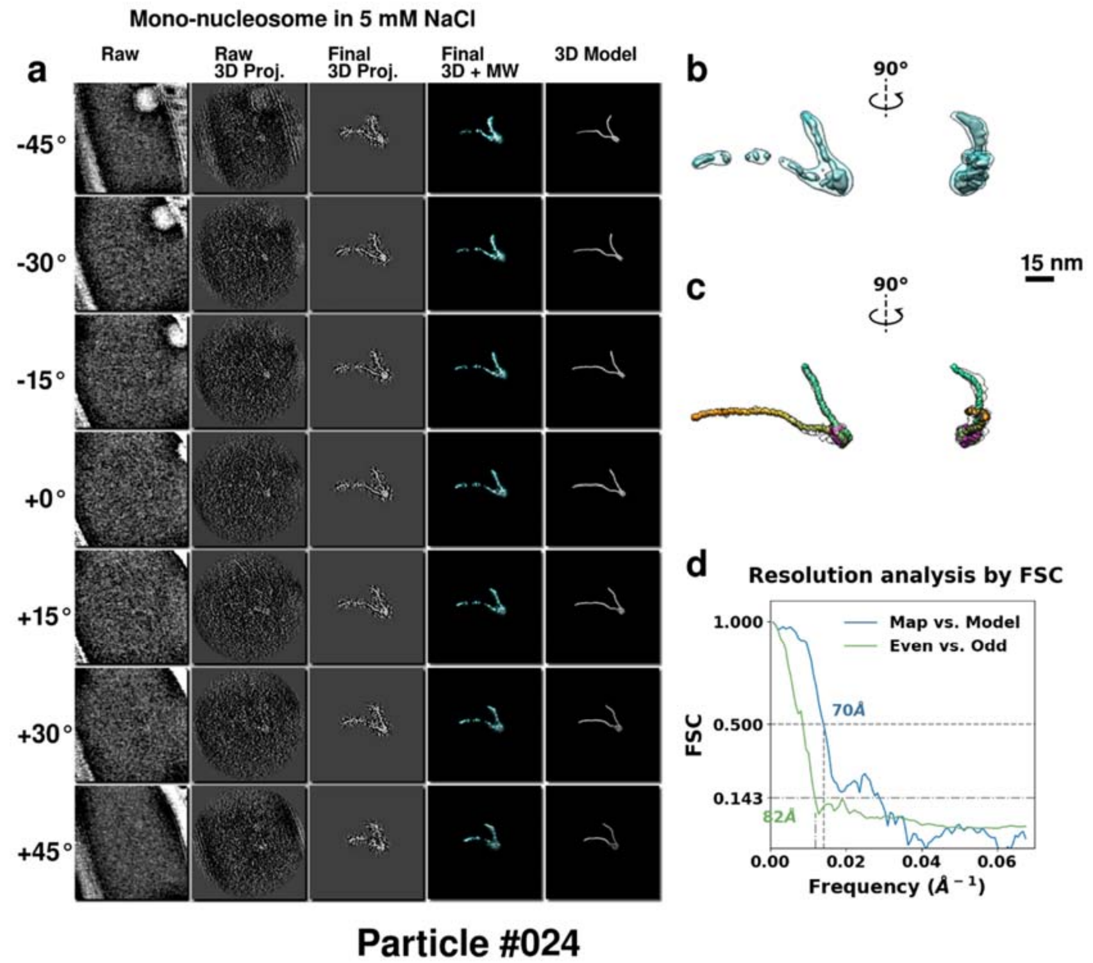

Supplementary Fig. 37. Cryo-ET 3D reconstruction of individual mono-nucleosome particle (index no. 24) in 5 mM NaCl. a, IPET 3D reconstruction of individual mono-nucleosome particles. The first column shows seven representative tilt images of an individual particle after CTF correction. Through alignment of the tilt images to a common center for 3D reconstruction via iterative refinement, the second and third columns display the 3D projections of the reconstruction before and after particle-shaped masking, respectively. The fourth column shows the final 3D reconstruction with missing wedge correction, and the fifth column presents the flexibly fitted model at the corresponding tilt angles. **b**, Zoomed-in view of the final 3D density map displayed in orthogonal views, shown at two contour levels. **c**, Superimposition of the high contour level map from (b) onto its flexibly fitted model. **d**, Resolution evaluation of the final 3D density map using two criteria: Fourier shell correlation (FSC) between two-half maps reconstructed from the even and odd index of the tilted series and FSC between the final 3D map and the fitted structure model. The resolution for the former and latter criteria is evaluated at frequencies of 0.5 and 0.143, respectively.

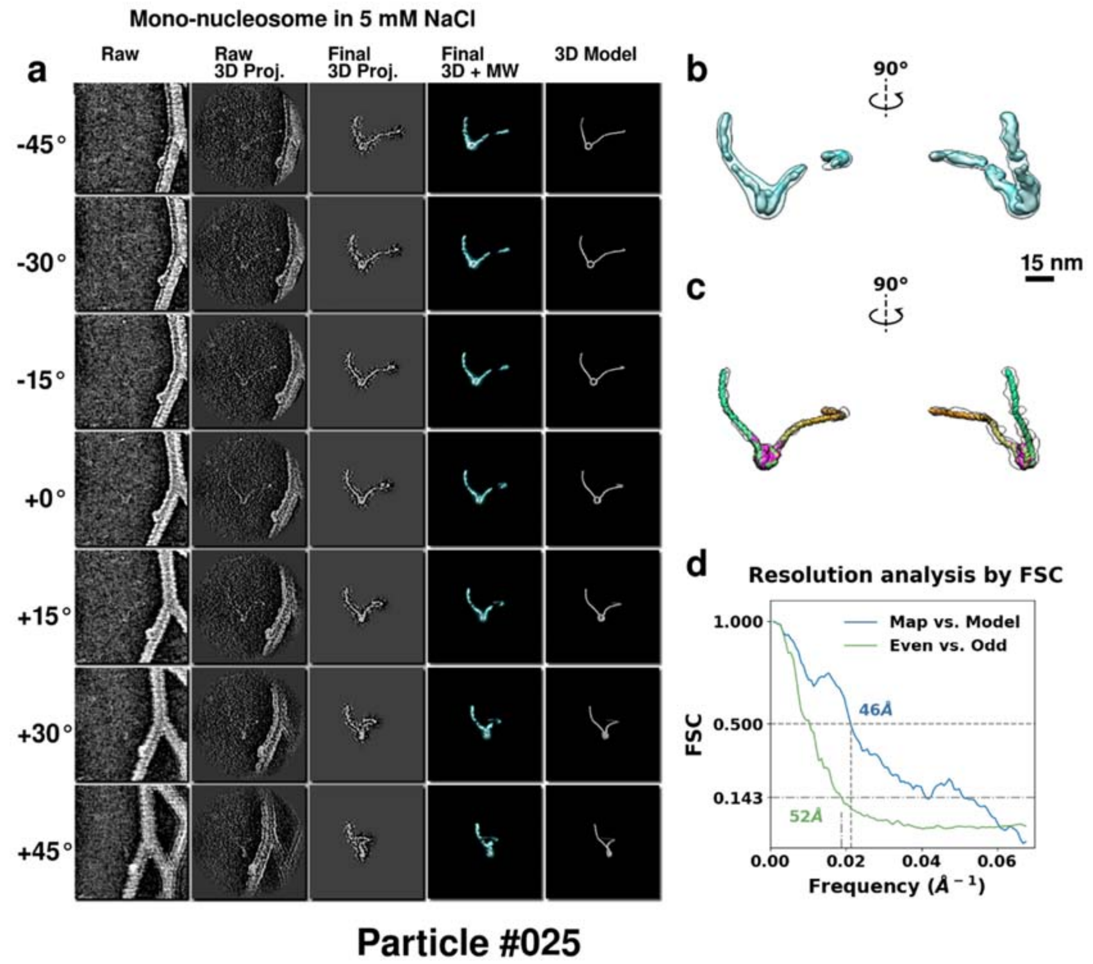

Supplementary Fig. 38. Cryo-ET 3D reconstruction of individual mono-nucleosome particle (index no. 25) in 5 mM NaCl. a, IPET 3D reconstruction of individual mono-nucleosome particles. The first column shows seven representative tilt images of an individual particle after CTF correction. Through alignment of the tilt images to a common center for 3D reconstruction via iterative refinement, the second and third columns display the 3D projections of the reconstruction before and after particle-shaped masking, respectively. The fourth column shows the final 3D reconstruction with missing wedge correction, and the fifth column presents the flexibly fitted model at the corresponding tilt angles. **b**, Zoomed-in view of the final 3D density map displayed in orthogonal views, shown at two contour levels. **c**, Superimposition of the high contour level map from (b) onto its flexibly fitted model. **d**, Resolution evaluation of the final 3D density map using two criteria: Fourier shell correlation (FSC) between two-half maps reconstructed from the even and odd index of the tilted series and FSC between the final 3D map and the fitted structure model. The resolution for the former and latter criteria is evaluated at frequencies of 0.5 and 0.143, respectively.

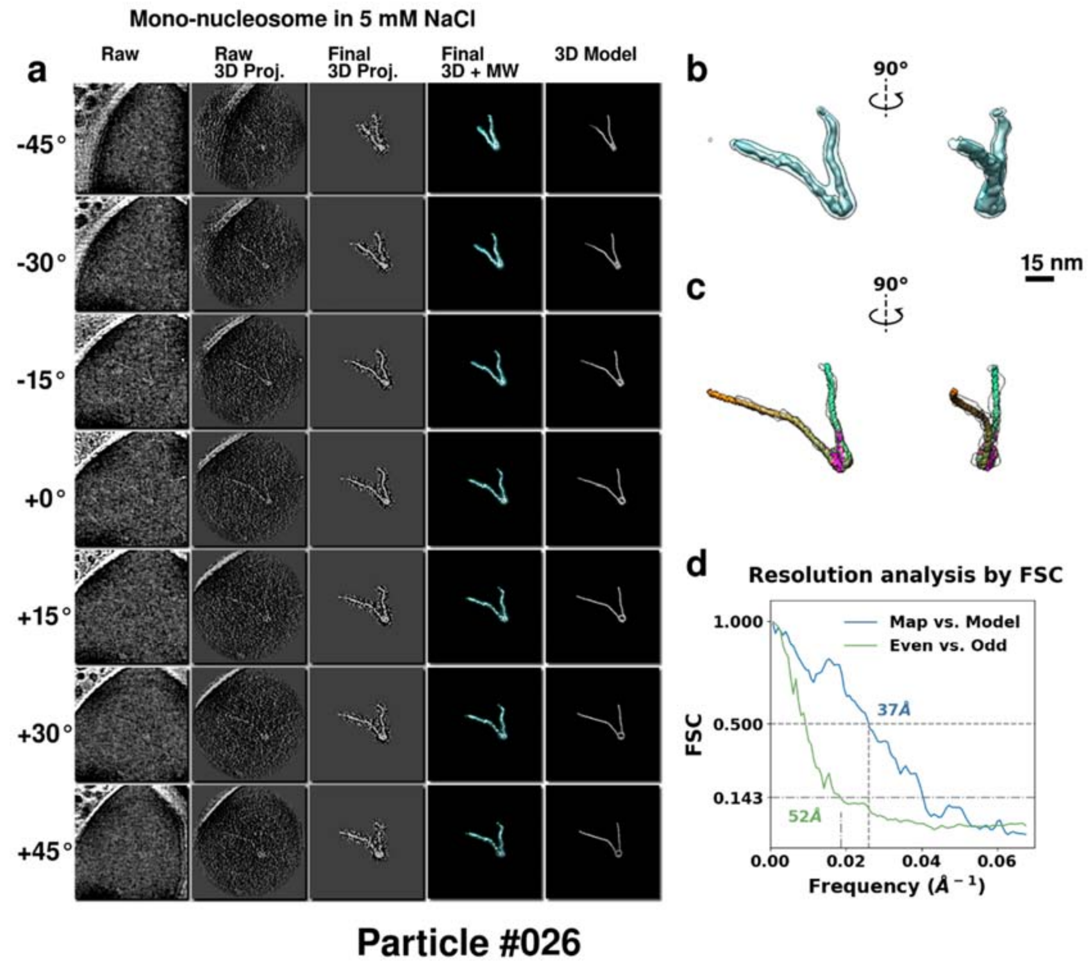

Supplementary Fig. 39. Cryo-ET 3D reconstruction of individual mono-nucleosome particle (index no. 26) in 5 mM NaCl. a, IPET 3D reconstruction of individual mono-nucleosome particles. The first column shows seven representative tilt images of an individual particle after CTF correction. Through alignment of the tilt images to a common center for 3D reconstruction via iterative refinement, the second and third columns display the 3D projections of the reconstruction before and after particle-shaped masking, respectively. The fourth column shows the final 3D reconstruction with missing wedge correction, and the fifth column presents the flexibly fitted model at the corresponding tilt angles. **b**, Zoomed-in view of the final 3D density map displayed in orthogonal views, shown at two contour levels. **c**, Superimposition of the high contour level map from (b) onto its flexibly fitted model. **d**, Resolution evaluation of the final 3D density map using two criteria: Fourier shell correlation (FSC) between two-half maps reconstructed from the even and odd index of the tilted series and FSC between the final 3D map and the fitted structure model. The resolution for the former and latter criteria is evaluated at frequencies of 0.5 and 0.143, respectively.

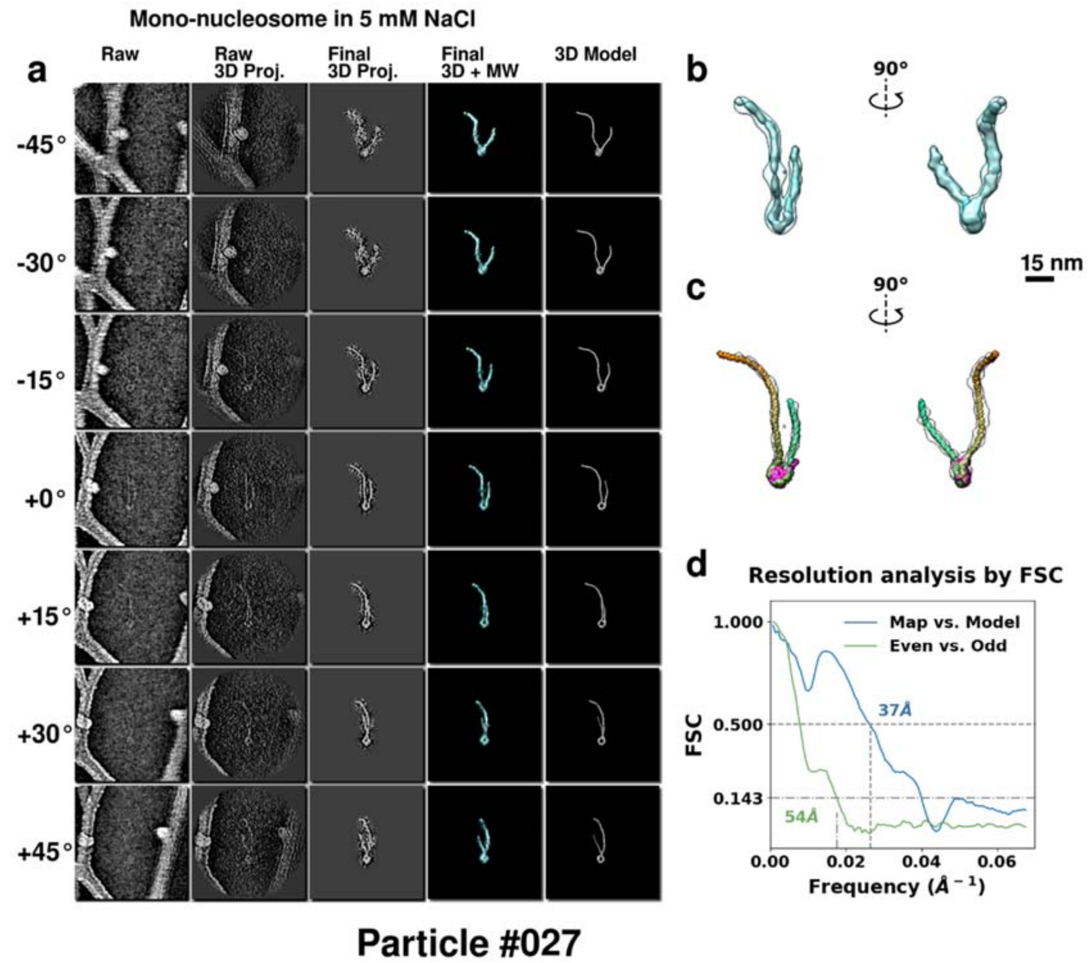

Supplementary Fig. 40. Cryo-ET 3D reconstruction of individual mono-nucleosome particle (index no. 27) in 5 mM NaCl. a, IPET 3D reconstruction of individual mono-nucleosome particles. The first column shows seven representative tilt images of an individual particle after CTF correction. Through alignment of the tilt images to a common center for 3D reconstruction via iterative refinement, the second and third columns display the 3D projections of the reconstruction before and after particle-shaped masking, respectively. The fourth column shows the final 3D reconstruction with missing wedge correction, and the fifth column presents the flexibly fitted model at the corresponding tilt angles. **b**, Zoomed-in view of the final 3D density map displayed in orthogonal views, shown at two contour levels. **c**, Superimposition of the high contour level map from (b) onto its flexibly fitted model. **d**, Resolution evaluation of the final 3D density map using two criteria: Fourier shell correlation (FSC) between two-half maps reconstructed from the even and odd index of the tilted series and FSC between the final 3D map and the fitted structure model. The resolution for the former and latter criteria is evaluated at frequencies of 0.5 and 0.143, respectively.

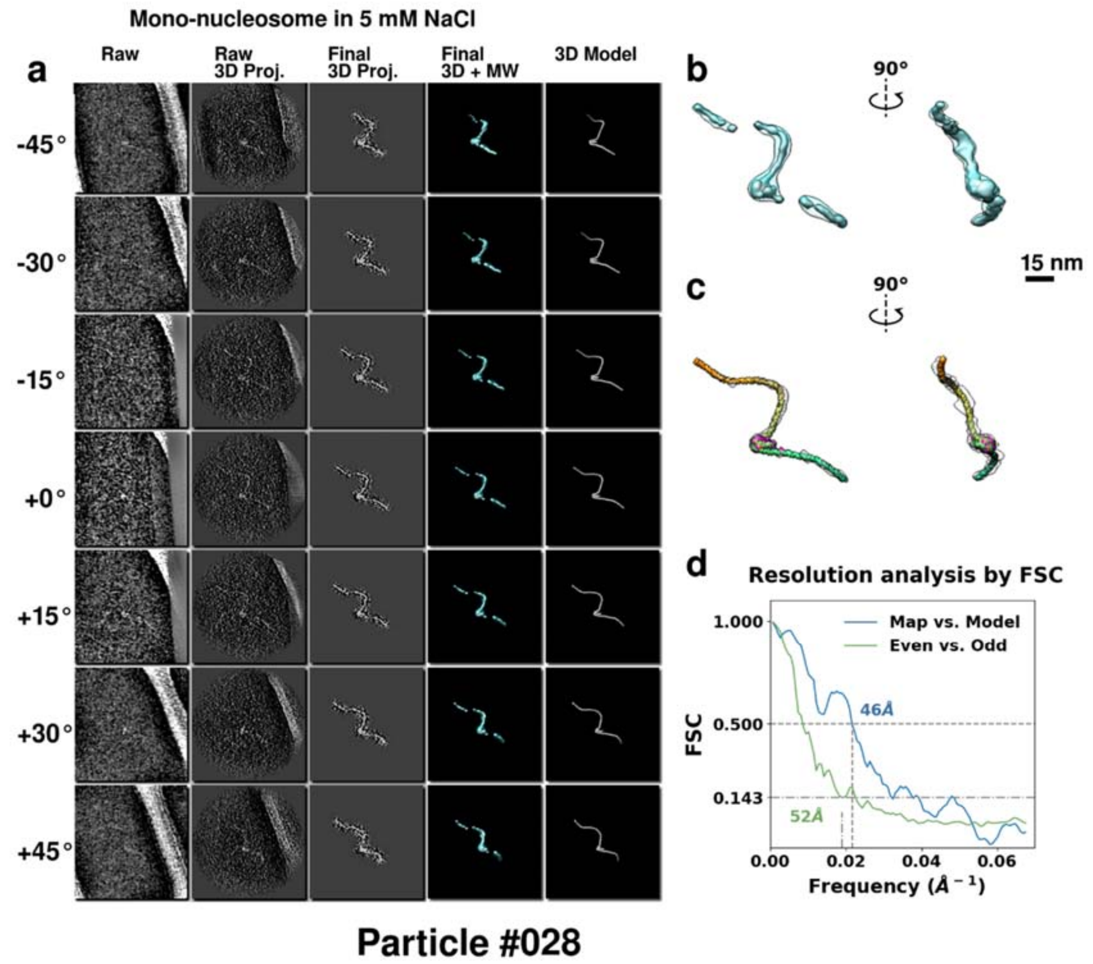

Supplementary Fig. 41. Cryo-ET 3D reconstruction of individual mono-nucleosome particle (index no. 28) in 5 mM NaCl. a, IPET 3D reconstruction of individual mono-nucleosome particles. The first column shows seven representative tilt images of an individual particle after CTF correction. Through alignment of the tilt images to a common center for 3D reconstruction via iterative refinement, the second and third columns display the 3D projections of the reconstruction before and after particle-shaped masking, respectively. The fourth column shows the final 3D reconstruction with missing wedge correction, and the fifth column presents the flexibly fitted model at the corresponding tilt angles. **b**, Zoomed-in view of the final 3D density map displayed in orthogonal views, shown at two contour levels. **c**, Superimposition of the high contour level map from (b) onto its flexibly fitted model. **d**, Resolution evaluation of the final 3D density map using two criteria: Fourier shell correlation (FSC) between two-half maps reconstructed from the even and odd index of the tilted series and FSC between the final 3D map and the fitted structure model. The resolution for the former and latter criteria is evaluated at frequencies of 0.5 and 0.143, respectively.

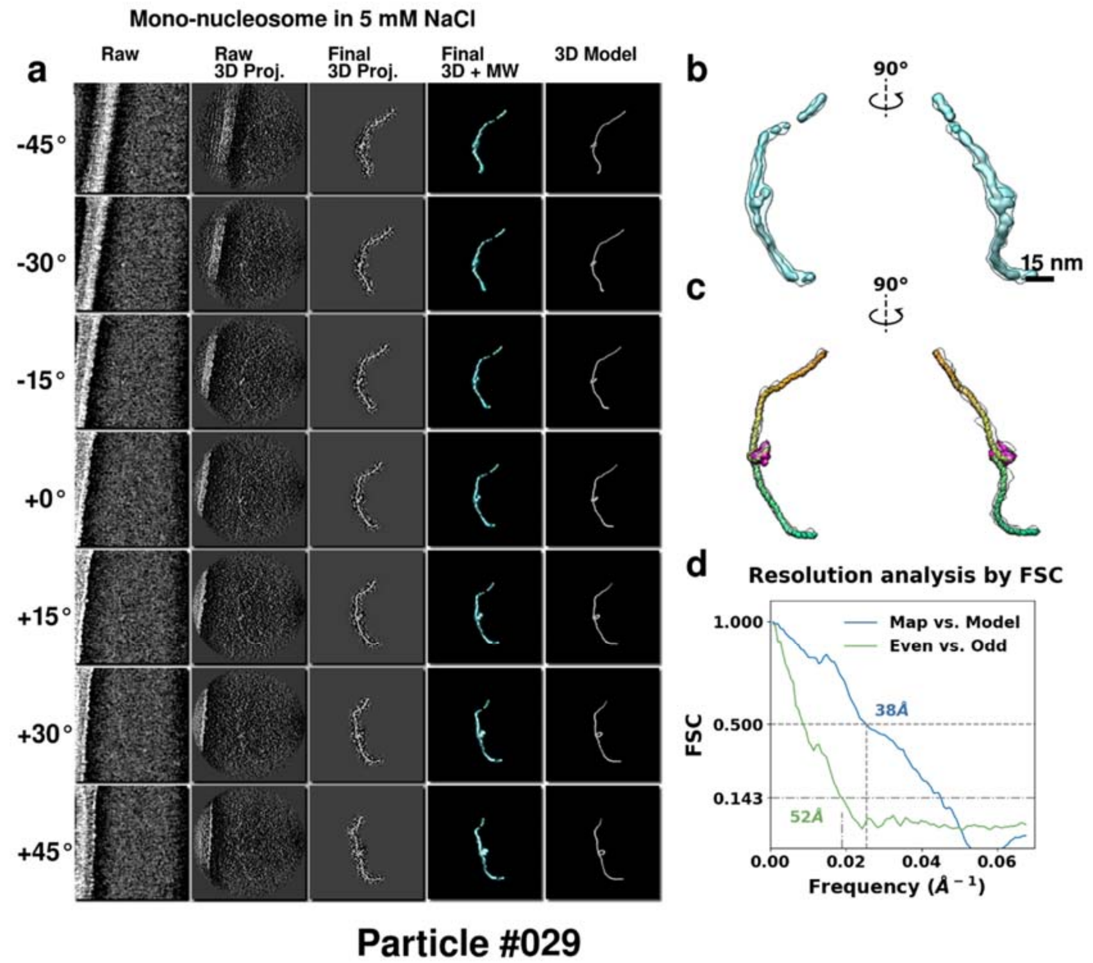

Supplementary Fig. 42. Cryo-ET 3D reconstruction of individual mono-nucleosome particle (index no. 29) in 5 mM NaCl. a, IPET 3D reconstruction of individual mono-nucleosome particles. The first column shows seven representative tilt images of an individual particle after CTF correction. Through alignment of the tilt images to a common center for 3D reconstruction via iterative refinement, the second and third columns display the 3D projections of the reconstruction before and after particle-shaped masking, respectively. The fourth column shows the final 3D reconstruction with missing wedge correction, and the fifth column presents the flexibly fitted model at the corresponding tilt angles. **b**, Zoomed-in view of the final 3D density map displayed in orthogonal views, shown at two contour levels. **c**, Superimposition of the high contour level map from (b) onto its flexibly fitted model. **d**, Resolution evaluation of the final 3D density map using two criteria: Fourier shell correlation (FSC) between two-half maps reconstructed from the even and odd index of the tilted series and FSC between the final 3D map and the fitted structure model. The resolution for the former and latter criteria is evaluated at frequencies of 0.5 and 0.143, respectively.

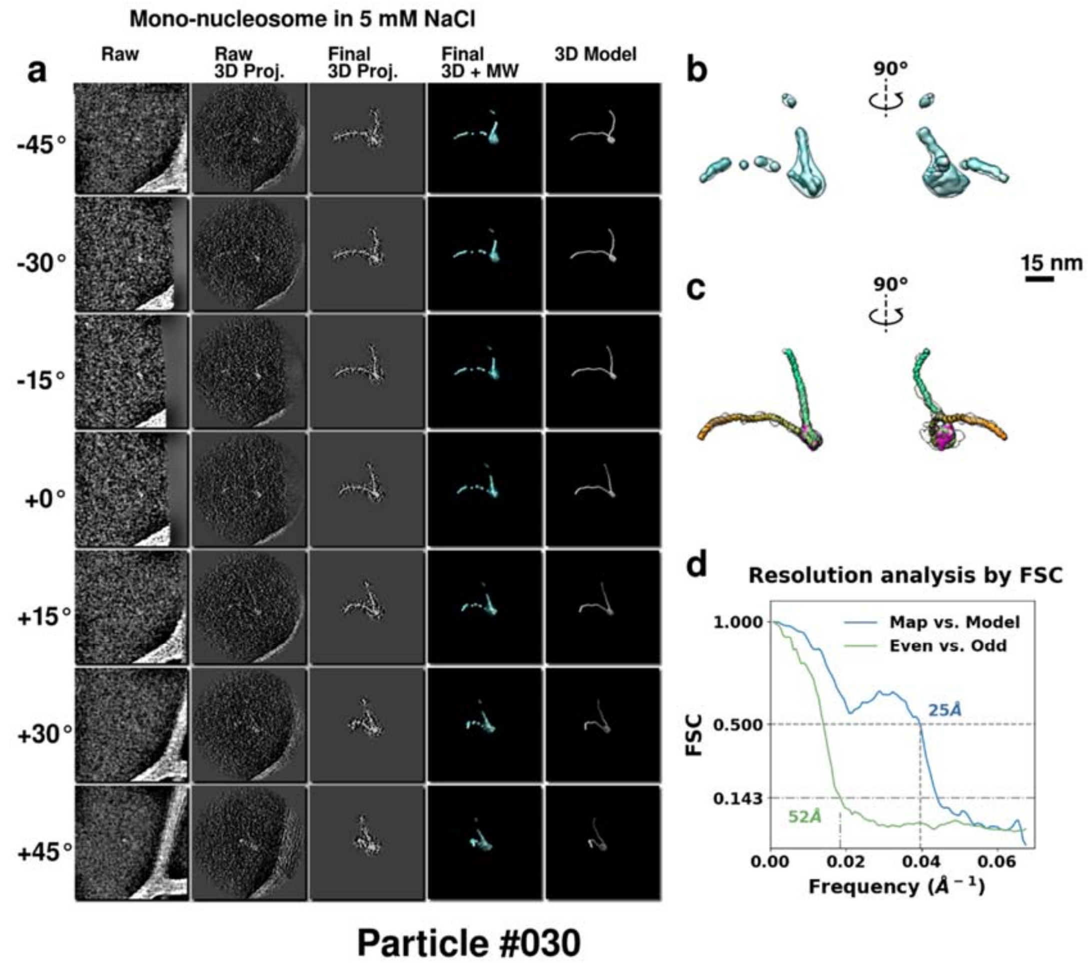

Supplementary Fig. 43. Cryo-ET 3D reconstruction of individual mono-nucleosome particle (index no. 30) in 5 mM NaCl. a, IPET 3D reconstruction of individual mono-nucleosome particles. The first column shows seven representative tilt images of an individual particle after CTF correction. Through alignment of the tilt images to a common center for 3D reconstruction via iterative refinement, the second and third columns display the 3D projections of the reconstruction before and after particle-shaped masking, respectively. The fourth column shows the final 3D reconstruction with missing wedge correction, and the fifth column presents the flexibly fitted model at the corresponding tilt angles. **b**, Zoomed-in view of the final 3D density map displayed in orthogonal views, shown at two contour levels. **c**, Superimposition of the high contour level map from (b) onto its flexibly fitted model. **d**, Resolution evaluation of the final 3D density map using two criteria: Fourier shell correlation (FSC) between two-half maps reconstructed from the even and odd index of the tilted series and FSC between the final 3D map and the fitted structure model. The resolution for the former and latter criteria is evaluated at frequencies of 0.5 and 0.143, respectively.

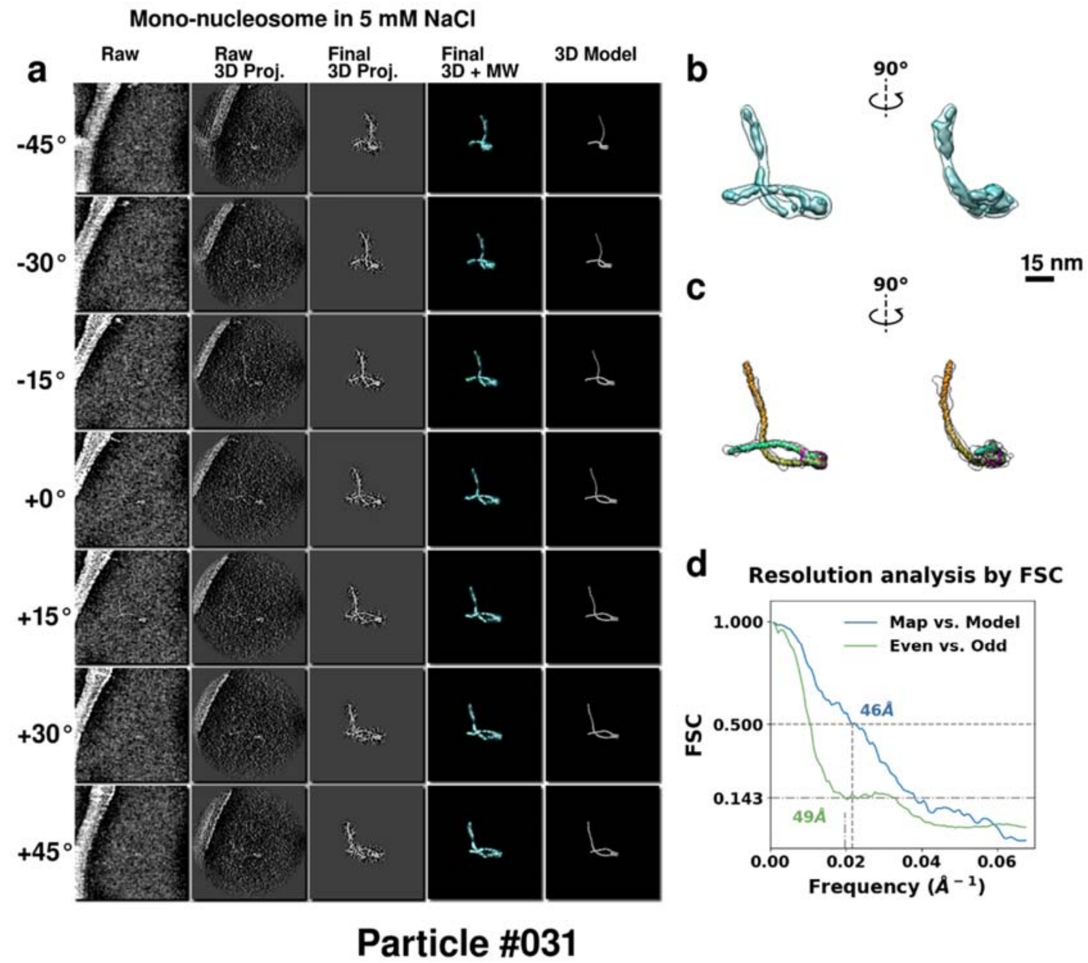

Supplementary Fig. 44. Cryo-ET 3D reconstruction of individual mono-nucleosome particle (index no. 31) in 5 mM NaCl. a, IPET 3D reconstruction of individual mono-nucleosome particles. The first column shows seven representative tilt images of an individual particle after CTF correction. Through alignment of the tilt images to a common center for 3D reconstruction via iterative refinement, the second and third columns display the 3D projections of the reconstruction before and after particle-shaped masking, respectively. The fourth column shows the final 3D reconstruction with missing wedge correction, and the fifth column presents the flexibly fitted model at the corresponding tilt angles. **b**, Zoomed-in view of the final 3D density map displayed in orthogonal views, shown at two contour levels. **c**, Superimposition of the high contour level map from (b) onto its flexibly fitted model. **d**, Resolution evaluation of the final 3D density map using two criteria: Fourier shell correlation (FSC) between two-half maps reconstructed from the even and odd index of the tilted series and FSC between the final 3D map and the fitted structure model. The resolution for the former and latter criteria is evaluated at frequencies of 0.5 and 0.143, respectively.

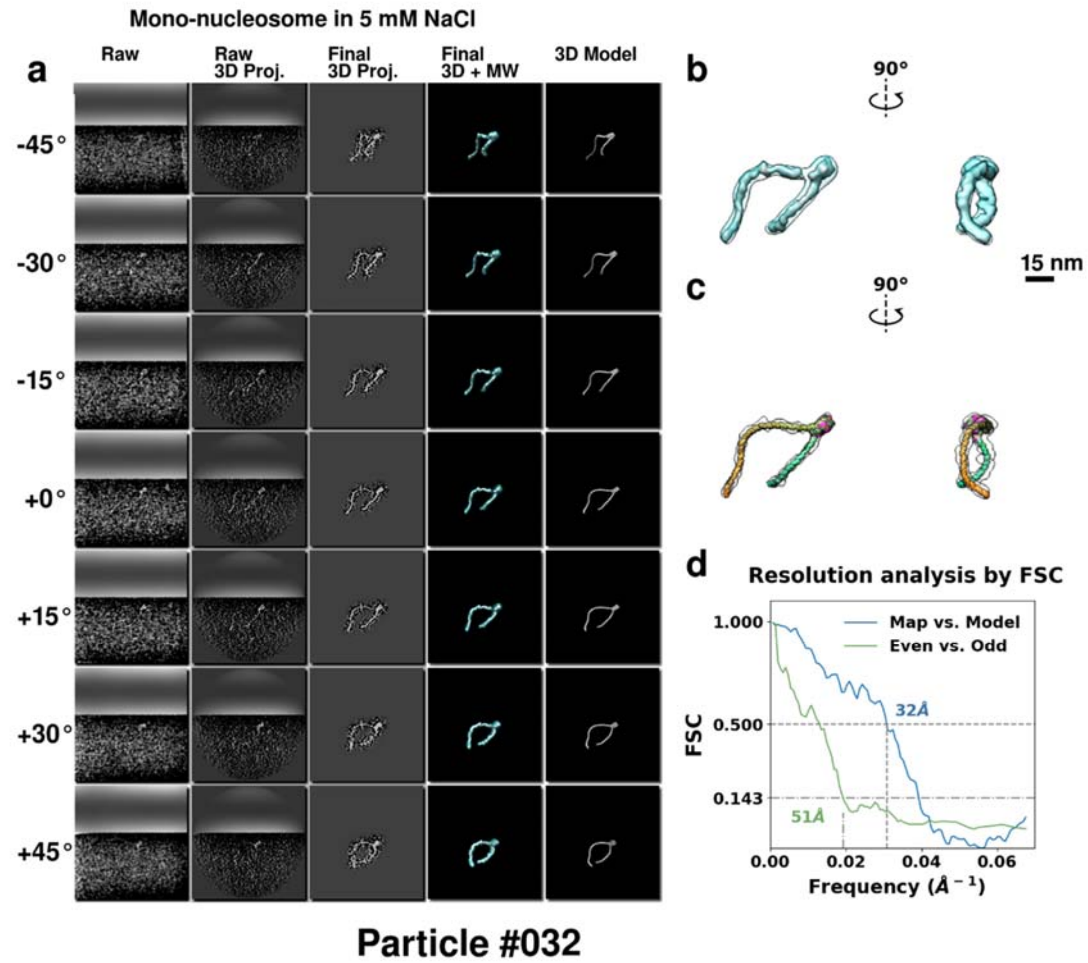

Supplementary Fig. 45. Cryo-ET 3D reconstruction of individual mono-nucleosome particle (index no. 32) in 5 mM NaCl. a, IPET 3D reconstruction of individual mono-nucleosome particles. The first column shows seven representative tilt images of an individual particle after CTF correction. Through alignment of the tilt images to a common center for 3D reconstruction via iterative refinement, the second and third columns display the 3D projections of the reconstruction before and after particle-shaped masking, respectively. The fourth column shows the final 3D reconstruction with missing wedge correction, and the fifth column presents the flexibly fitted model at the corresponding tilt angles. **b**, Zoomed-in view of the final 3D density map displayed in orthogonal views, shown at two contour levels. **c**, Superimposition of the high contour level map from (b) onto its flexibly fitted model. **d**, Resolution evaluation of the final 3D density map using two criteria: Fourier shell correlation (FSC) between two-half maps reconstructed from the even and odd index of the tilted series and FSC between the final 3D map and the fitted structure model. The resolution for the former and latter criteria is evaluated at frequencies of 0.5 and 0.143, respectively.

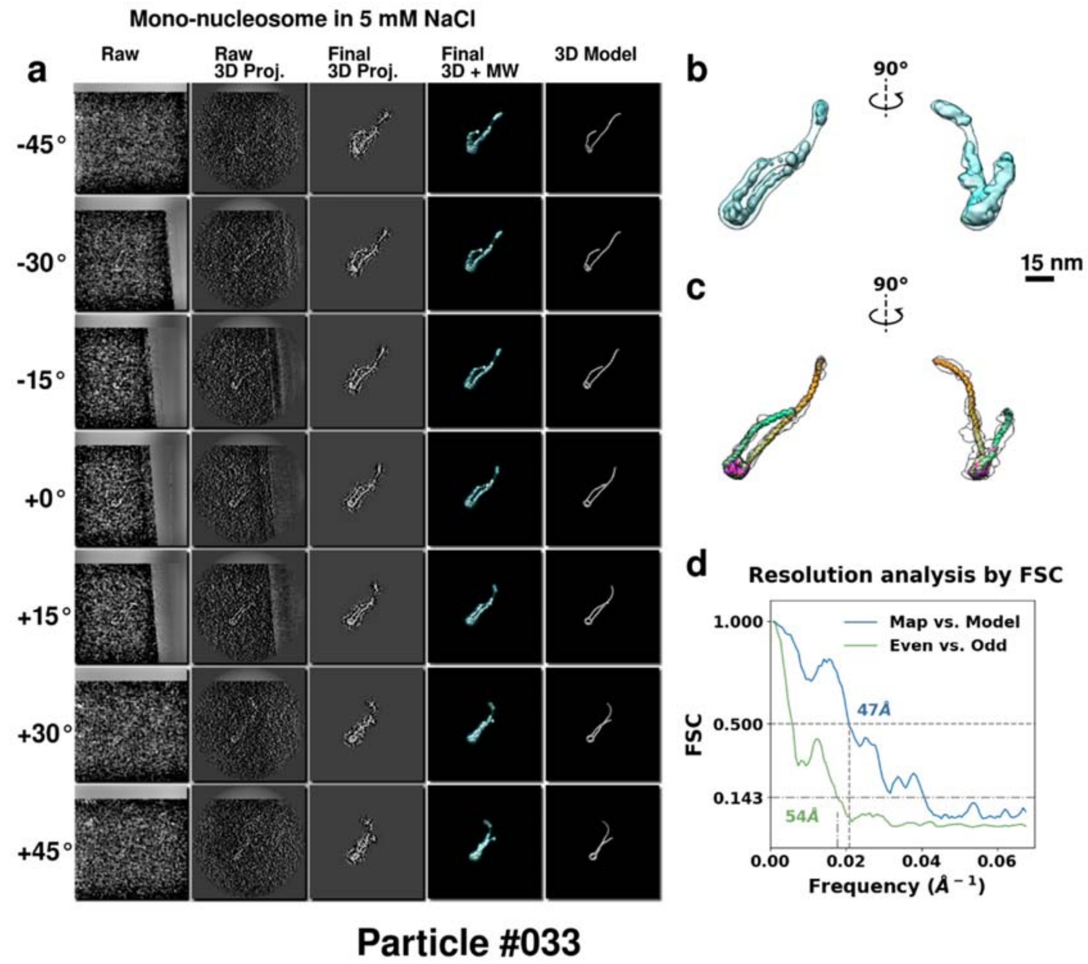

Supplementary Fig. 46. Cryo-ET 3D reconstruction of individual mono-nucleosome particle (index no. 33) in 5 mM NaCl. a, IPET 3D reconstruction of individual mono-nucleosome particles. The first column shows seven representative tilt images of an individual particle after CTF correction. Through alignment of the tilt images to a common center for 3D reconstruction via iterative refinement, the second and third columns display the 3D projections of the reconstruction before and after particle-shaped masking, respectively. The fourth column shows the final 3D reconstruction with missing wedge correction, and the fifth column presents the flexibly fitted model at the corresponding tilt angles. **b**, Zoomed-in view of the final 3D density map displayed in orthogonal views, shown at two contour levels. **c**, Superimposition of the high contour level map from (b) onto its flexibly fitted model. **d**, Resolution evaluation of the final 3D density map using two criteria: Fourier shell correlation (FSC) between two-half maps reconstructed from the even and odd index of the tilted series and FSC between the final 3D map and the fitted structure model. The resolution for the former and latter criteria is evaluated at frequencies of 0.5 and 0.143, respectively.

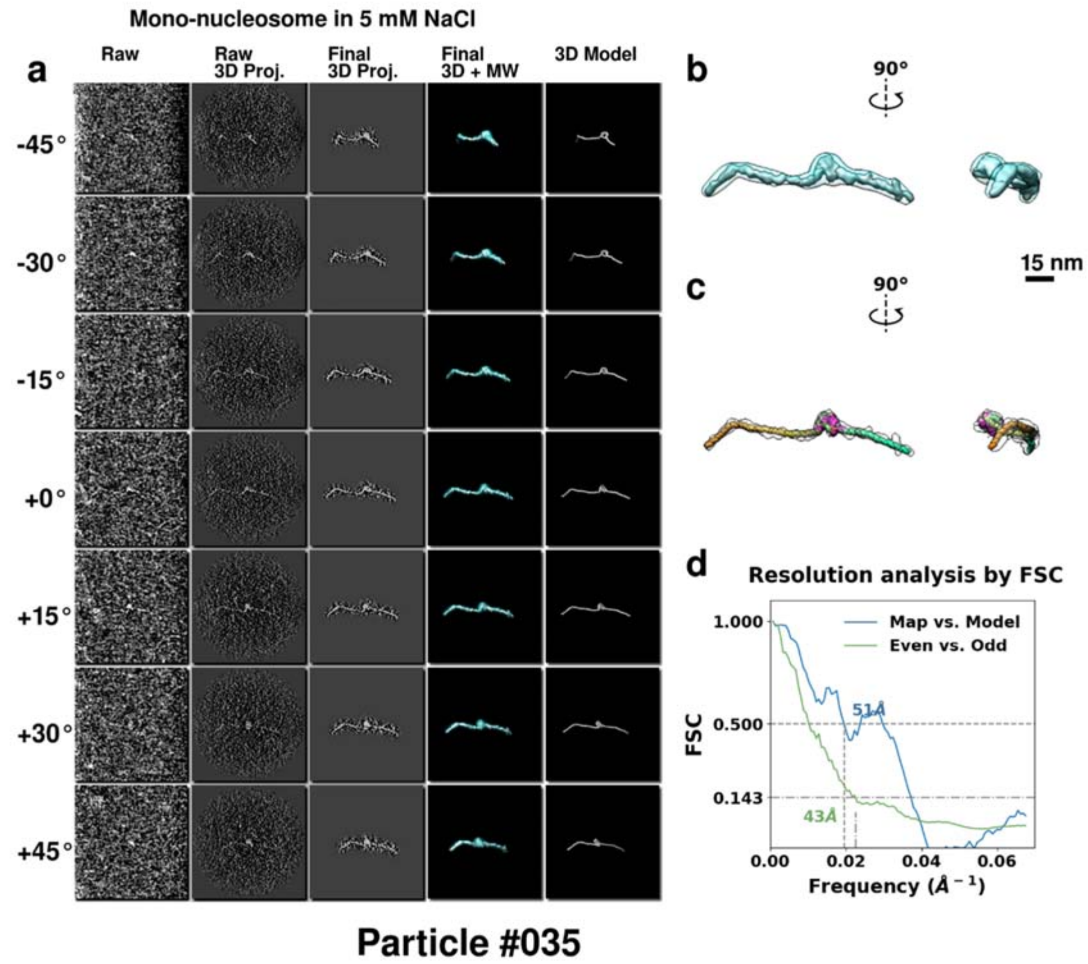

Supplementary Fig. 47. Cryo-ET 3D reconstruction of individual mono-nucleosome particle (index no. 34) in 5 mM NaCl. a, IPET 3D reconstruction of individual mono-nucleosome particles. The first column shows seven representative tilt images of an individual particle after CTF correction. Through alignment of the tilt images to a common center for 3D reconstruction via iterative refinement, the second and third columns display the 3D projections of the reconstruction before and after particle-shaped masking, respectively. The fourth column shows the final 3D reconstruction with missing wedge correction, and the fifth column presents the flexibly fitted model at the corresponding tilt angles. **b**, Zoomed-in view of the final 3D density map displayed in orthogonal views, shown at two contour levels. **c**, Superimposition of the high contour level map from (b) onto its flexibly fitted model. **d**, Resolution evaluation of the final 3D density map using two criteria: Fourier shell correlation (FSC) between two-half maps reconstructed from the even and odd index of the tilted series and FSC between the final 3D map and the fitted structure model. The resolution for the former and latter criteria is evaluated at frequencies of 0.5 and 0.143, respectively.

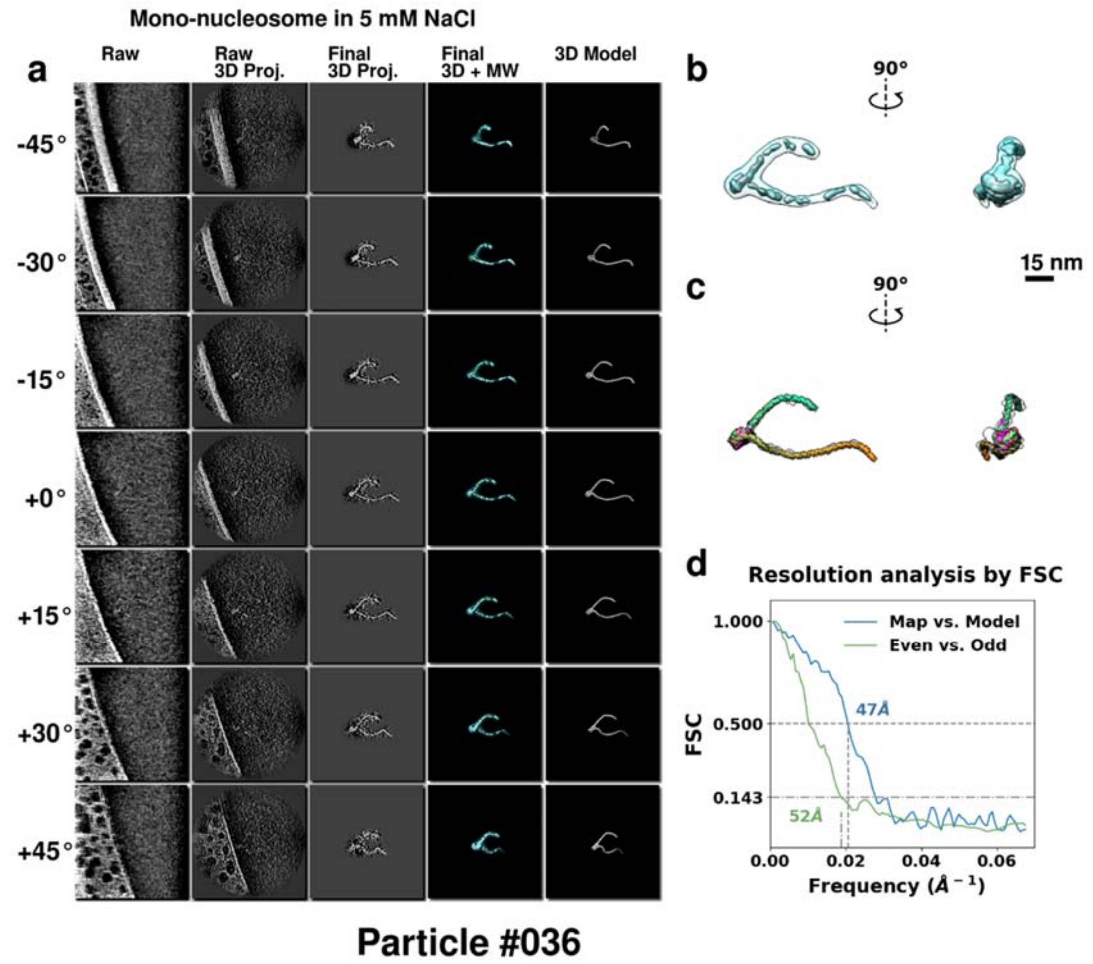

Supplementary Fig. 48. Cryo-ET 3D reconstruction of individual mono-nucleosome particle (index no. 35) in 5 mM NaCl. a, IPET 3D reconstruction of individual mono-nucleosome particles. The first column shows seven representative tilt images of an individual particle after CTF correction. Through alignment of the tilt images to a common center for 3D reconstruction via iterative refinement, the second and third columns display the 3D projections of the reconstruction before and after particle-shaped masking, respectively. The fourth column shows the final 3D reconstruction with missing wedge correction, and the fifth column presents the flexibly fitted model at the corresponding tilt angles. **b**, Zoomed-in view of the final 3D density map displayed in orthogonal views, shown at two contour levels. **c**, Superimposition of the high contour level map from (b) onto its flexibly fitted model. **d**, Resolution evaluation of the final 3D density map using two criteria: Fourier shell correlation (FSC) between two-half maps reconstructed from the even and odd index of the tilted series and FSC between the final 3D map and the fitted structure model. The resolution for the former and latter criteria is evaluated at frequencies of 0.5 and 0.143, respectively.

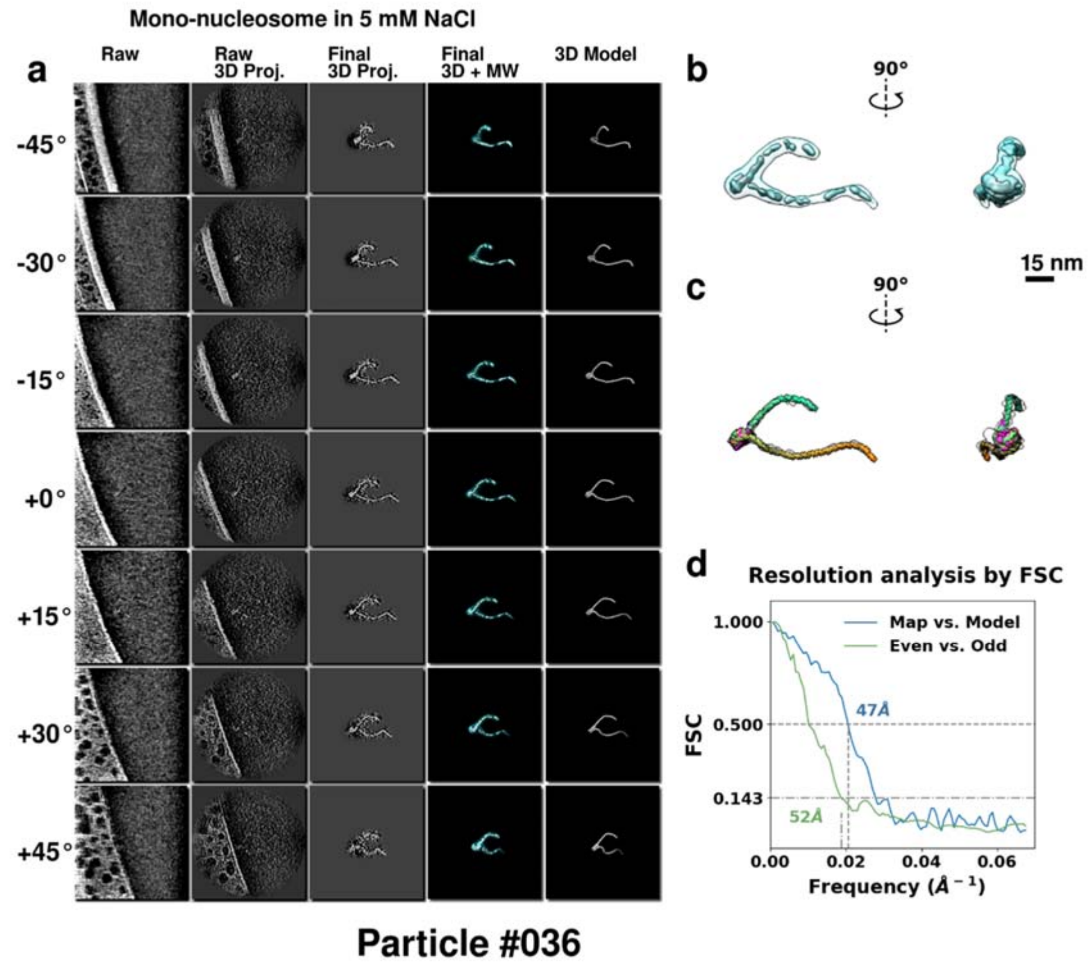

Supplementary Fig. 49. Cryo-ET 3D reconstruction of individual mono-nucleosome particle (index no. 36) in 5 mM NaCl. a, IPET 3D reconstruction of individual mono-nucleosome particles. The first column shows seven representative tilt images of an individual particle after CTF correction. Through alignment of the tilt images to a common center for 3D reconstruction via iterative refinement, the second and third columns display the 3D projections of the reconstruction before and after particle-shaped masking, respectively. The fourth column shows the final 3D reconstruction with missing wedge correction, and the fifth column presents the flexibly fitted model at the corresponding tilt angles. **b**, Zoomed-in view of the final 3D density map displayed in orthogonal views, shown at two contour levels. **c**, Superimposition of the high contour level map from (b) onto its flexibly fitted model. **d**, Resolution evaluation of the final 3D density map using two criteria: Fourier shell correlation (FSC) between two-half maps reconstructed from the even and odd index of the tilted series and FSC between the final 3D map and the fitted structure model. The resolution for the former and latter criteria is evaluated at frequencies of 0.5 and 0.143, respectively.

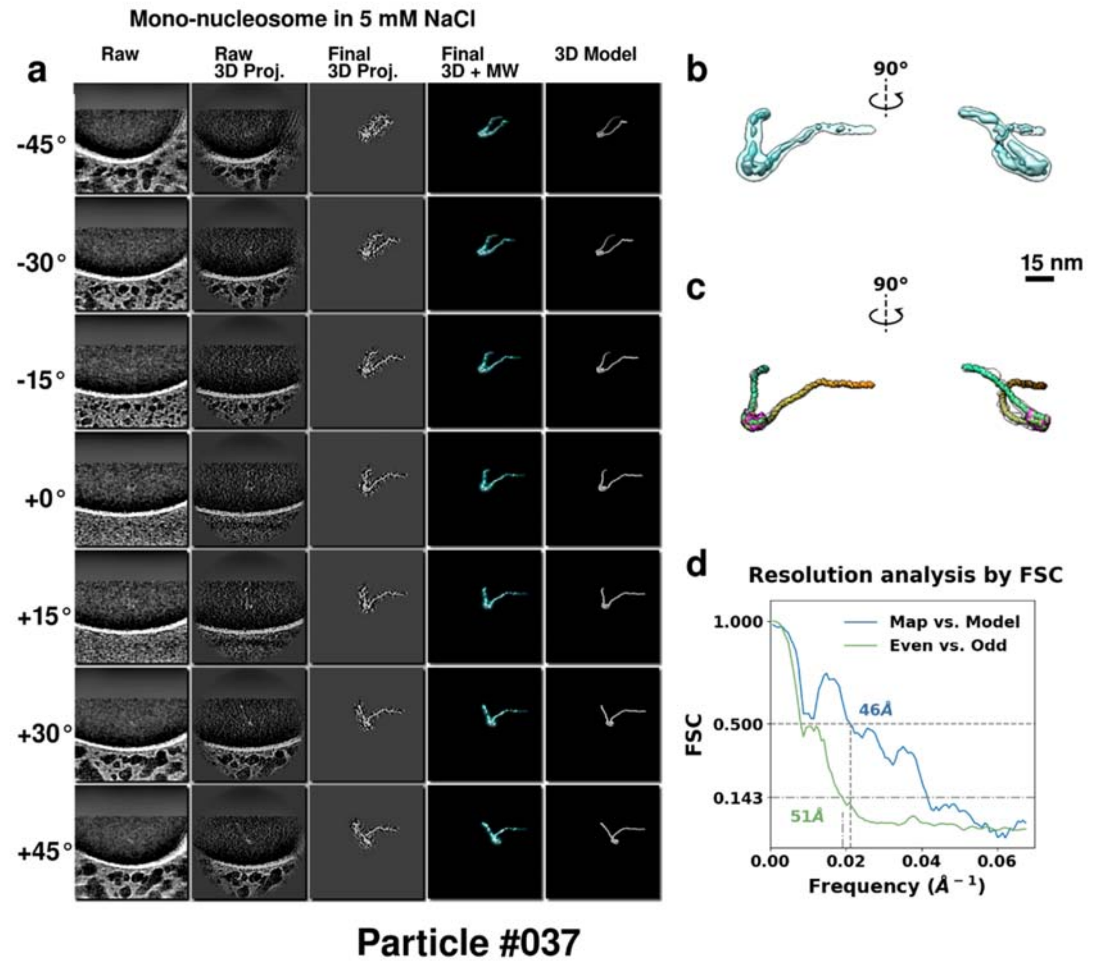

Supplementary Fig. 50. Cryo-ET 3D reconstruction of individual mono-nucleosome particle (index no. 37) in 5 mM NaCl. a, IPET 3D reconstruction of individual mono-nucleosome particles. The first column shows seven representative tilt images of an individual particle after CTF correction. Through alignment of the tilt images to a common center for 3D reconstruction via iterative refinement, the second and third columns display the 3D projections of the reconstruction before and after particle-shaped masking, respectively. The fourth column shows the final 3D reconstruction with missing wedge correction, and the fifth column presents the flexibly fitted model at the corresponding tilt angles. **b**, Zoomed-in view of the final 3D density map displayed in orthogonal views, shown at two contour levels. **c**, Superimposition of the high contour level map from (b) onto its flexibly fitted model. **d**, Resolution evaluation of the final 3D density map using two criteria: Fourier shell correlation (FSC) between two-half maps reconstructed from the even and odd index of the tilted series and FSC between the final 3D map and the fitted structure model. The resolution for the former and latter criteria is evaluated at frequencies of 0.5 and 0.143, respectively.

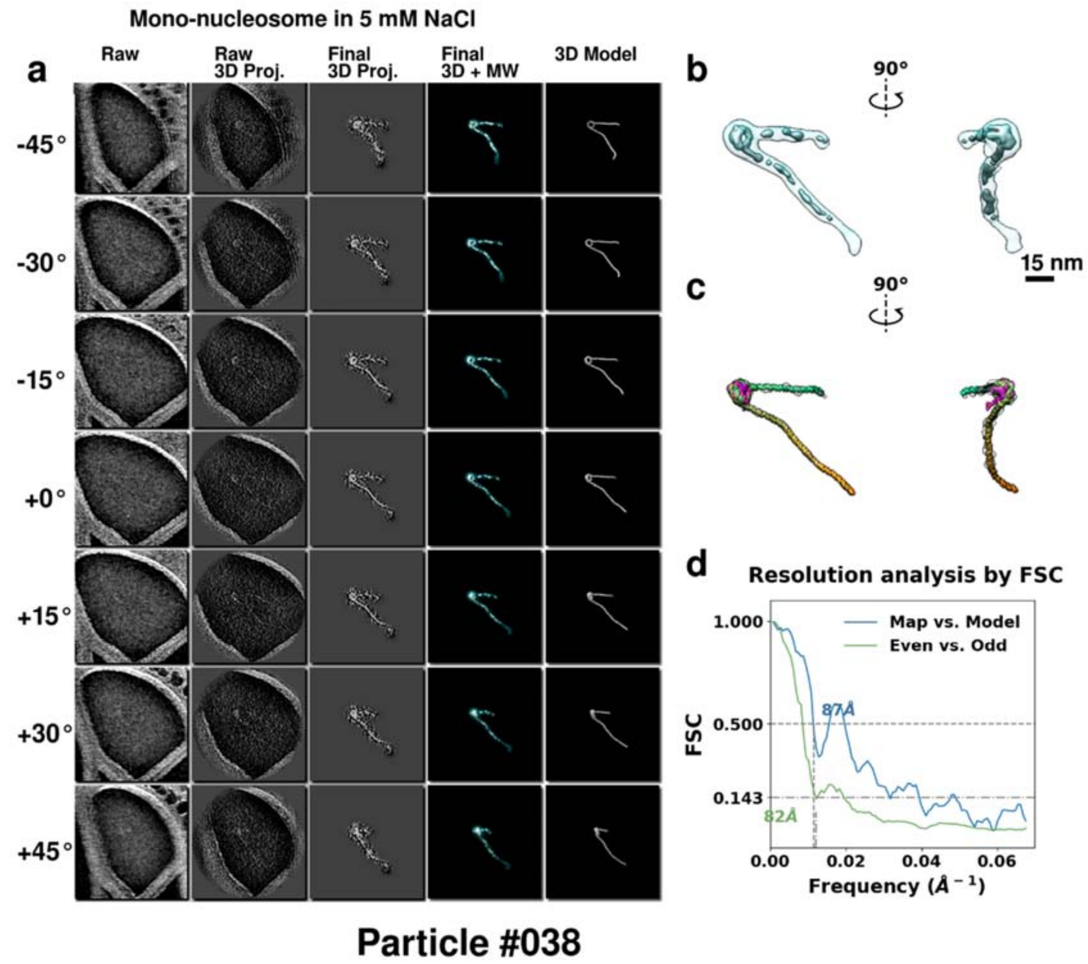

Supplementary Fig. 51. Cryo-ET 3D reconstruction of individual mono-nucleosome particle (index no. 38) in 5 mM NaCl. a, IPET 3D reconstruction of individual mono-nucleosome particles. The first column shows seven representative tilt images of an individual particle after CTF correction. Through alignment of the tilt images to a common center for 3D reconstruction via iterative refinement, the second and third columns display the 3D projections of the reconstruction before and after particle-shaped masking, respectively. The fourth column shows the final 3D reconstruction with missing wedge correction, and the fifth column presents the flexibly fitted model at the corresponding tilt angles. **b**, Zoomed-in view of the final 3D density map displayed in orthogonal views, shown at two contour levels. **c**, Superimposition of the high contour level map from (b) onto its flexibly fitted model. **d**, Resolution evaluation of the final 3D density map using two criteria: Fourier shell correlation (FSC) between two-half maps reconstructed from the even and odd index of the tilted series and FSC between the final 3D map and the fitted structure model. The resolution for the former and latter criteria is evaluated at frequencies of 0.5 and 0.143, respectively.

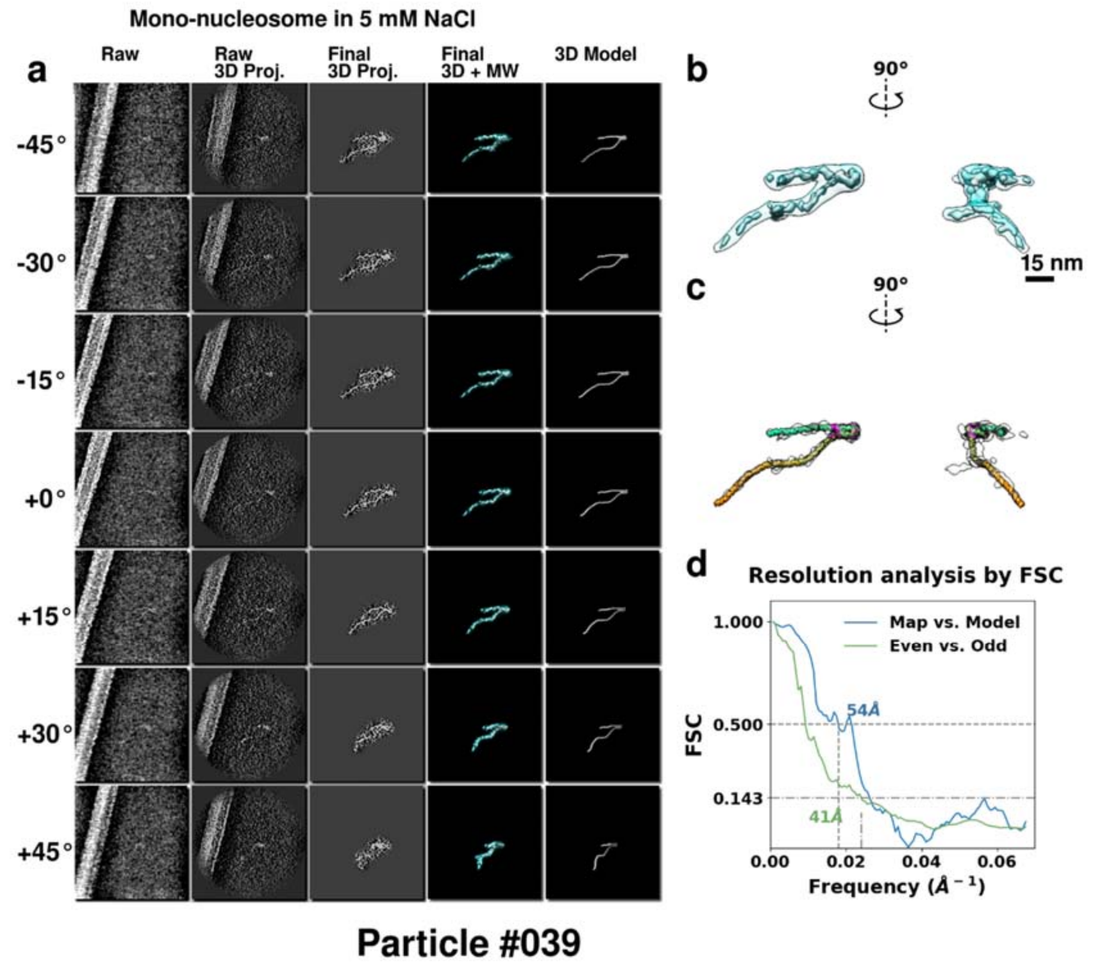

Supplementary Fig. 52. Cryo-ET 3D reconstruction of individual mono-nucleosome particle (index no. 39) in 5 mM NaCl. a, IPET 3D reconstruction of individual mono-nucleosome particles. The first column shows seven representative tilt images of an individual particle after CTF correction. Through alignment of the tilt images to a common center for 3D reconstruction via iterative refinement, the second and third columns display the 3D projections of the reconstruction before and after particle-shaped masking, respectively. The fourth column shows the final 3D reconstruction with missing wedge correction, and the fifth column presents the flexibly fitted model at the corresponding tilt angles. **b**, Zoomed-in view of the final 3D density map displayed in orthogonal views, shown at two contour levels. **c**, Superimposition of the high contour level map from (b) onto its flexibly fitted model. **d**, Resolution evaluation of the final 3D density map using two criteria: Fourier shell correlation (FSC) between two-half maps reconstructed from the even and odd index of the tilted series and FSC between the final 3D map and the fitted structure model. The resolution for the former and latter criteria is evaluated at frequencies of 0.5 and 0.143, respectively.

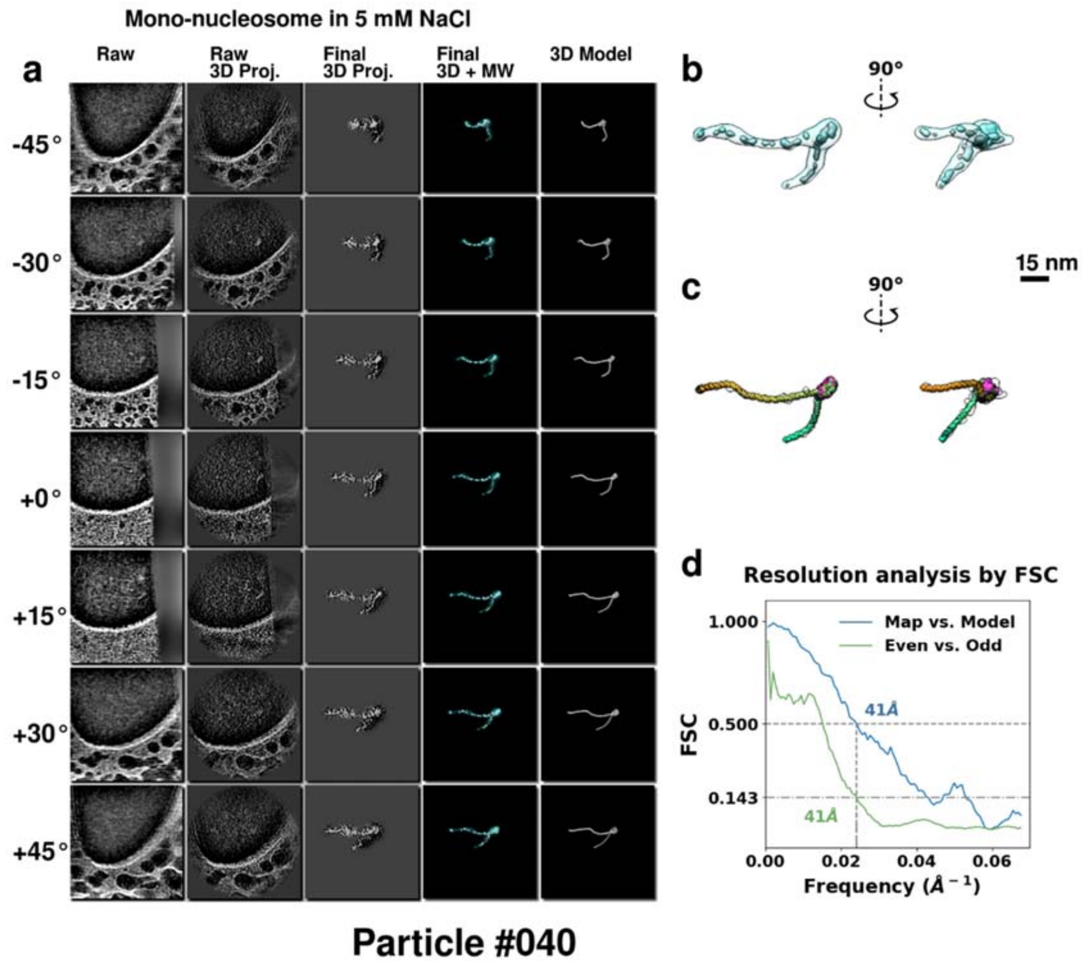

Supplementary Fig. 53. Cryo-ET 3D reconstruction of individual mono-nucleosome particle (index no. 40) in 5 mM NaCl. a, IPET 3D reconstruction of individual mono-nucleosome particles. The first column shows seven representative tilt images of an individual particle after CTF correction. Through alignment of the tilt images to a common center for 3D reconstruction via iterative refinement, the second and third columns display the 3D projections of the reconstruction before and after particle-shaped masking, respectively. The fourth column shows the final 3D reconstruction with missing wedge correction, and the fifth column presents the flexibly fitted model at the corresponding tilt angles. **b**, Zoomed-in view of the final 3D density map displayed in orthogonal views, shown at two contour levels. **c**, Superimposition of the high contour level map from (b) onto its flexibly fitted model. **d**, Resolution evaluation of the final 3D density map using two criteria: Fourier shell correlation (FSC) between two-half maps reconstructed from the even and odd index of the tilted series and FSC between the final 3D map and the fitted structure model. The resolution for the former and latter criteria is evaluated at frequencies of 0.5 and 0.143, respectively.

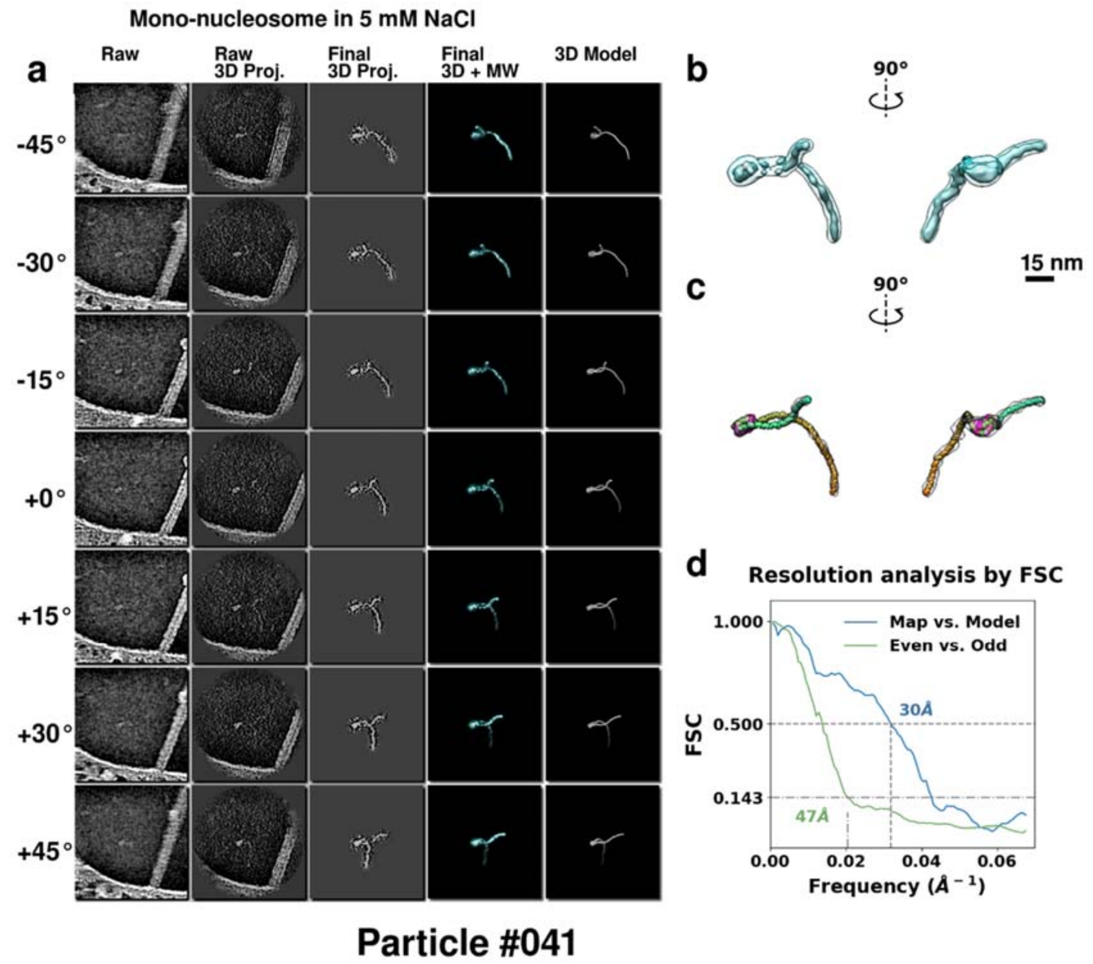

Supplementary Fig. 54. Cryo-ET 3D reconstruction of individual mono-nucleosome particle (index no. 41) in 5 mM NaCl. a, IPET 3D reconstruction of individual mono-nucleosome particles. The first column shows seven representative tilt images of an individual particle after CTF correction. Through alignment of the tilt images to a common center for 3D reconstruction via iterative refinement, the second and third columns display the 3D projections of the reconstruction before and after particle-shaped masking, respectively. The fourth column shows the final 3D reconstruction with missing wedge correction, and the fifth column presents the flexibly fitted model at the corresponding tilt angles. **b**, Zoomed-in view of the final 3D density map displayed in orthogonal views, shown at two contour levels. **c**, Superimposition of the high contour level map from (b) onto its flexibly fitted model. **d**, Resolution evaluation of the final 3D density map using two criteria: Fourier shell correlation (FSC) between two-half maps reconstructed from the even and odd index of the tilted series and FSC between the final 3D map and the fitted structure model. The resolution for the former and latter criteria is evaluated at frequencies of 0.5 and 0.143, respectively.

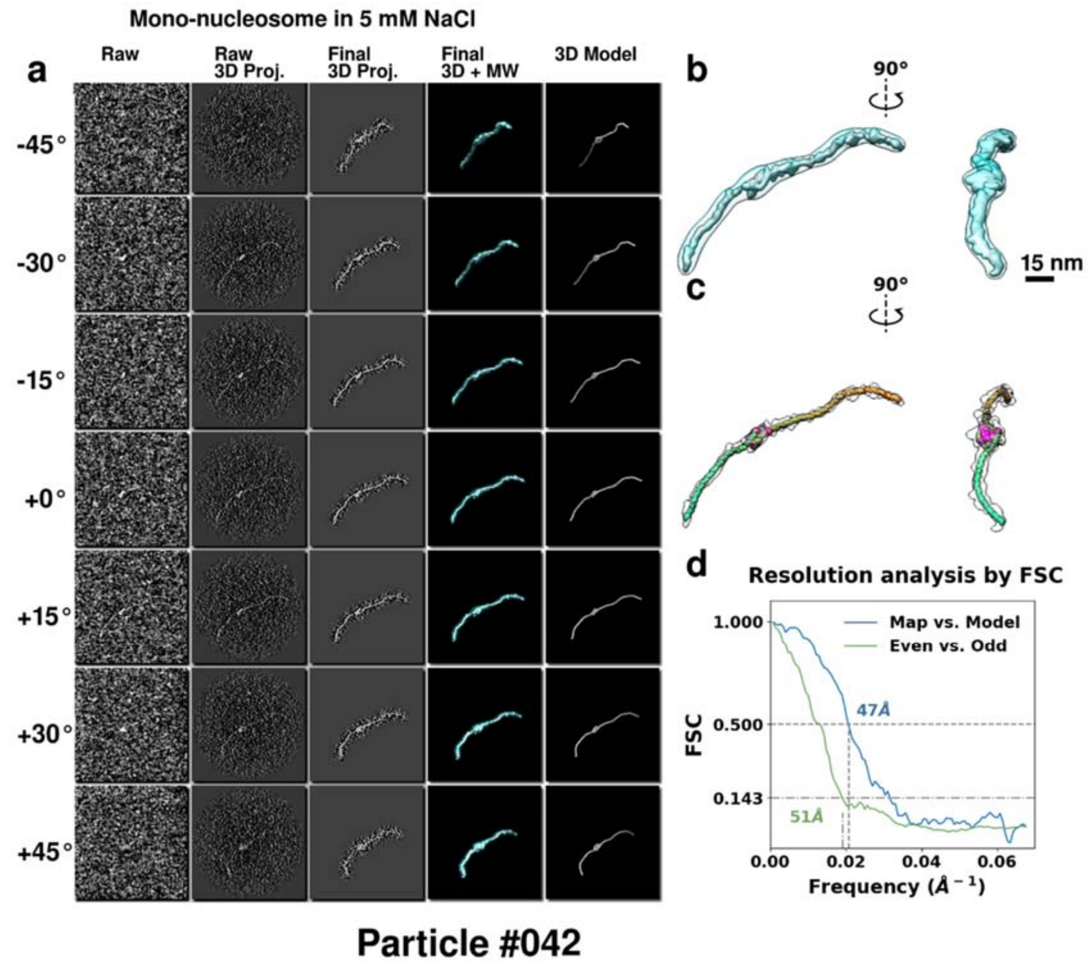

Supplementary Fig. 55. Cryo-ET 3D reconstruction of individual mono-nucleosome particle (index no. 42) in 5 mM NaCl. a, IPET 3D reconstruction of individual mono-nucleosome particles. The first column shows seven representative tilt images of an individual particle after CTF correction. Through alignment of the tilt images to a common center for 3D reconstruction via iterative refinement, the second and third columns display the 3D projections of the reconstruction before and after particle-shaped masking, respectively. The fourth column shows the final 3D reconstruction with missing wedge correction, and the fifth column presents the flexibly fitted model at the corresponding tilt angles. **b**, Zoomed-in view of the final 3D density map displayed in orthogonal views, shown at two contour levels. **c**, Superimposition of the high contour level map from (b) onto its flexibly fitted model. **d**, Resolution evaluation of the final 3D density map using two criteria: Fourier shell correlation (FSC) between two-half maps reconstructed from the even and odd index of the tilted series and FSC between the final 3D map and the fitted structure model. The resolution for the former and latter criteria is evaluated at frequencies of 0.5 and 0.143, respectively.

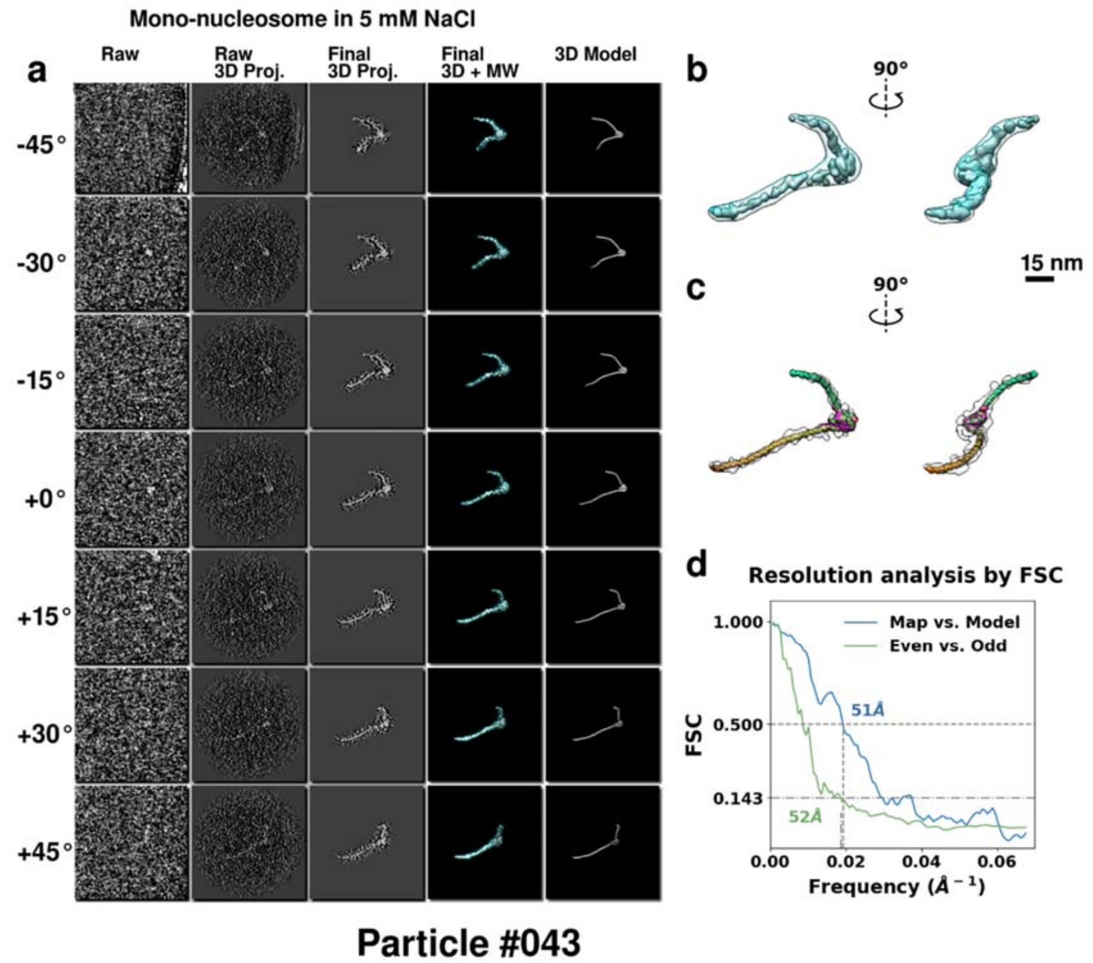

Supplementary Fig. 56. Cryo-ET 3D reconstruction of individual mono-nucleosome particle (index no. 43) in 5 mM NaCl. a, IPET 3D reconstruction of individual mono-nucleosome particles. The first column shows seven representative tilt images of an individual particle after CTF correction. Through alignment of the tilt images to a common center for 3D reconstruction via iterative refinement, the second and third columns display the 3D projections of the reconstruction before and after particle-shaped masking, respectively. The fourth column shows the final 3D reconstruction with missing wedge correction, and the fifth column presents the flexibly fitted model at the corresponding tilt angles. **b**, Zoomed-in view of the final 3D density map displayed in orthogonal views, shown at two contour levels. **c**, Superimposition of the high contour level map from (b) onto its flexibly fitted model. **d**, Resolution evaluation of the final 3D density map using two criteria: Fourier shell correlation (FSC) between two-half maps reconstructed from the even and odd index of the tilted series and FSC between the final 3D map and the fitted structure model. The resolution for the former and latter criteria is evaluated at frequencies of 0.5 and 0.143, respectively.

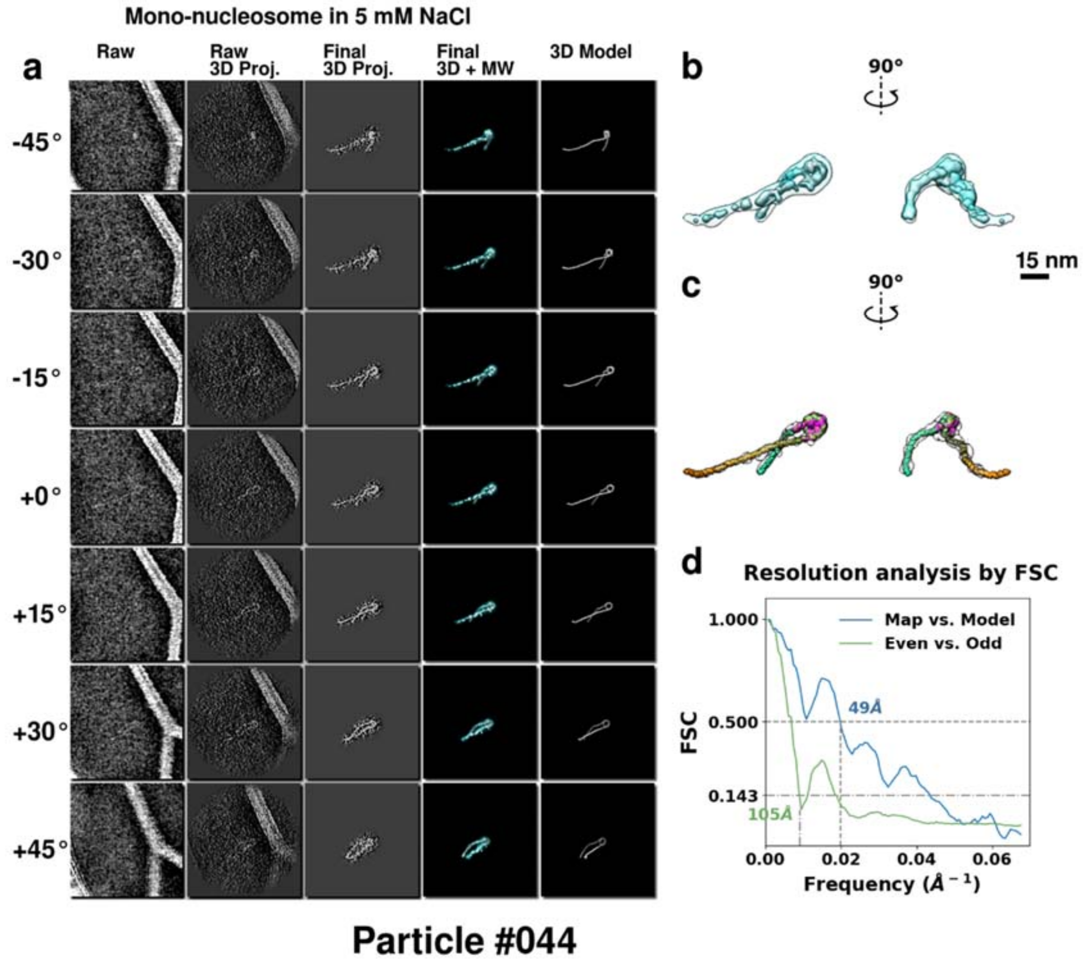

Supplementary Fig. 57. Cryo-ET 3D reconstruction of individual mono-nucleosome particle (index no. 44) in 5 mM NaCl. a, IPET 3D reconstruction of individual mono-nucleosome particles. The first column shows seven representative tilt images of an individual particle after CTF correction. Through alignment of the tilt images to a common center for 3D reconstruction via iterative refinement, the second and third columns display the 3D projections of the reconstruction before and after particle-shaped masking, respectively. The fourth column shows the final 3D reconstruction with missing wedge correction, and the fifth column presents the flexibly fitted model at the corresponding tilt angles. **b**, Zoomed-in view of the final 3D density map displayed in orthogonal views, shown at two contour levels. **c**, Superimposition of the high contour level map from (b) onto its flexibly fitted model. **d**, Resolution evaluation of the final 3D density map using two criteria: Fourier shell correlation (FSC) between two-half maps reconstructed from the even and odd index of the tilted series and FSC between the final 3D map and the fitted structure model. The resolution for the former and latter criteria is evaluated at frequencies of 0.5 and 0.143, respectively.

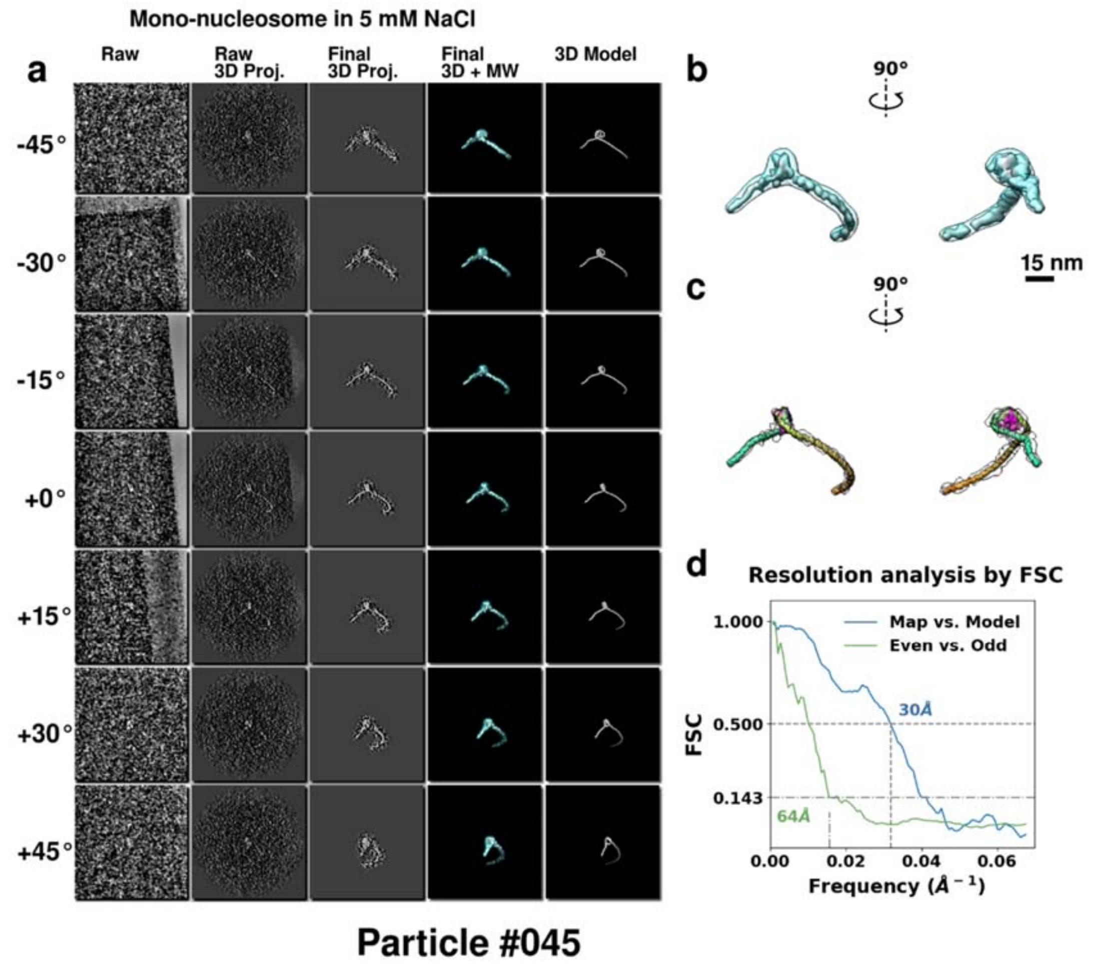

Supplementary Fig. 58. Cryo-ET 3D reconstruction of individual mono-nucleosome particle (index no. 45) in 5 mM NaCl. a, IPET 3D reconstruction of individual mono-nucleosome particles. The first column shows seven representative tilt images of an individual particle after CTF correction. Through alignment of the tilt images to a common center for 3D reconstruction via iterative refinement, the second and third columns display the 3D projections of the reconstruction before and after particle-shaped masking, respectively. The fourth column shows the final 3D reconstruction with missing wedge correction, and the fifth column presents the flexibly fitted model at the corresponding tilt angles. **b**, Zoomed-in view of the final 3D density map displayed in orthogonal views, shown at two contour levels. **c**, Superimposition of the high contour level map from (b) onto its flexibly fitted model. **d**, Resolution evaluation of the final 3D density map using two criteria: Fourier shell correlation (FSC) between two-half maps reconstructed from the even and odd index of the tilted series and FSC between the final 3D map and the fitted structure model. The resolution for the former and latter criteria is evaluated at frequencies of 0.5 and 0.143, respectively.

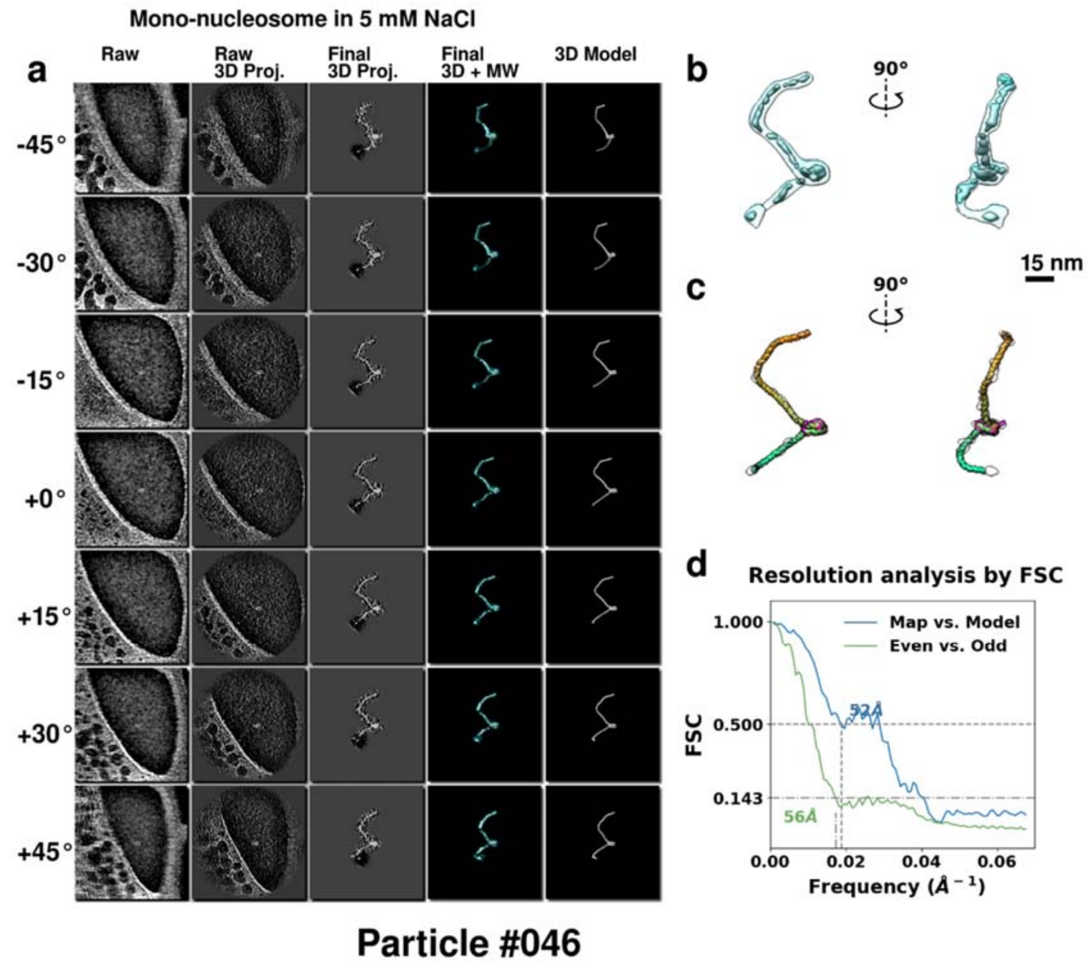

Supplementary Fig. 59. Cryo-ET 3D reconstruction of individual mono-nucleosome particle (index no. 46) in 5 mM NaCl. a, IPET 3D reconstruction of individual mono-nucleosome particles. The first column shows seven representative tilt images of an individual particle after CTF correction. Through alignment of the tilt images to a common center for 3D reconstruction via iterative refinement, the second and third columns display the 3D projections of the reconstruction before and after particle-shaped masking, respectively. The fourth column shows the final 3D reconstruction with missing wedge correction, and the fifth column presents the flexibly fitted model at the corresponding tilt angles. **b**, Zoomed-in view of the final 3D density map displayed in orthogonal views, shown at two contour levels. **c**, Superimposition of the high contour level map from (b) onto its flexibly fitted model. **d**, Resolution evaluation of the final 3D density map using two criteria: Fourier shell correlation (FSC) between two-half maps reconstructed from the even and odd index of the tilted series and FSC between the final 3D map and the fitted structure model. The resolution for the former and latter criteria is evaluated at frequencies of 0.5 and 0.143, respectively.

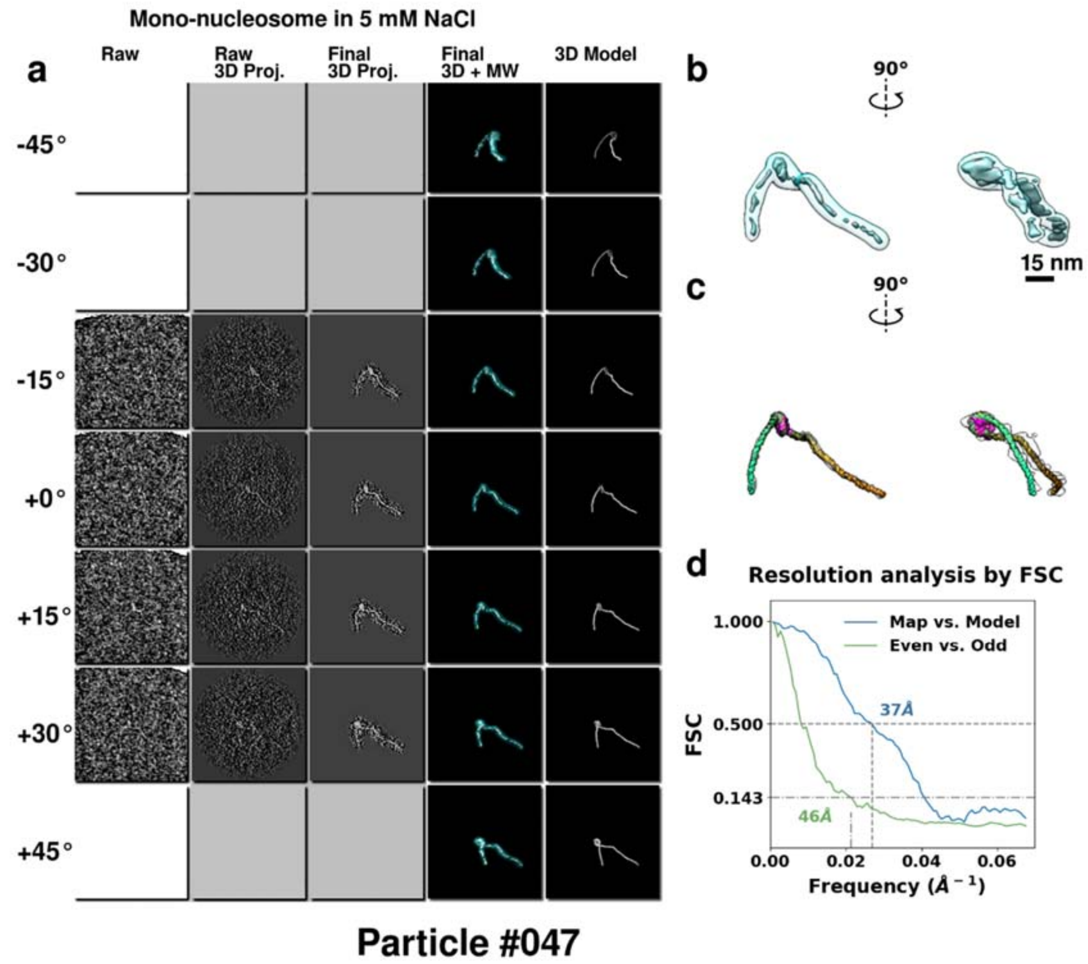

Supplementary Fig. 60. Cryo-ET 3D reconstruction of individual mono-nucleosome particle (index no. 47) in 5 mM NaCl. a, IPET 3D reconstruction of individual mono-nucleosome particles. The first column shows seven representative tilt images of an individual particle after CTF correction. Through alignment of the tilt images to a common center for 3D reconstruction via iterative refinement, the second and third columns display the 3D projections of the reconstruction before and after particle-shaped masking, respectively. The fourth column shows the final 3D reconstruction with missing wedge correction, and the fifth column presents the flexibly fitted model at the corresponding tilt angles. **b**, Zoomed-in view of the final 3D density map displayed in orthogonal views, shown at two contour levels. **c**, Superimposition of the high contour level map from (b) onto its flexibly fitted model. **d**, Resolution evaluation of the final 3D density map using two criteria: Fourier shell correlation (FSC) between two-half maps reconstructed from the even and odd index of the tilted series and FSC between the final 3D map and the fitted structure model. The resolution for the former and latter criteria is evaluated at frequencies of 0.5 and 0.143, respectively.

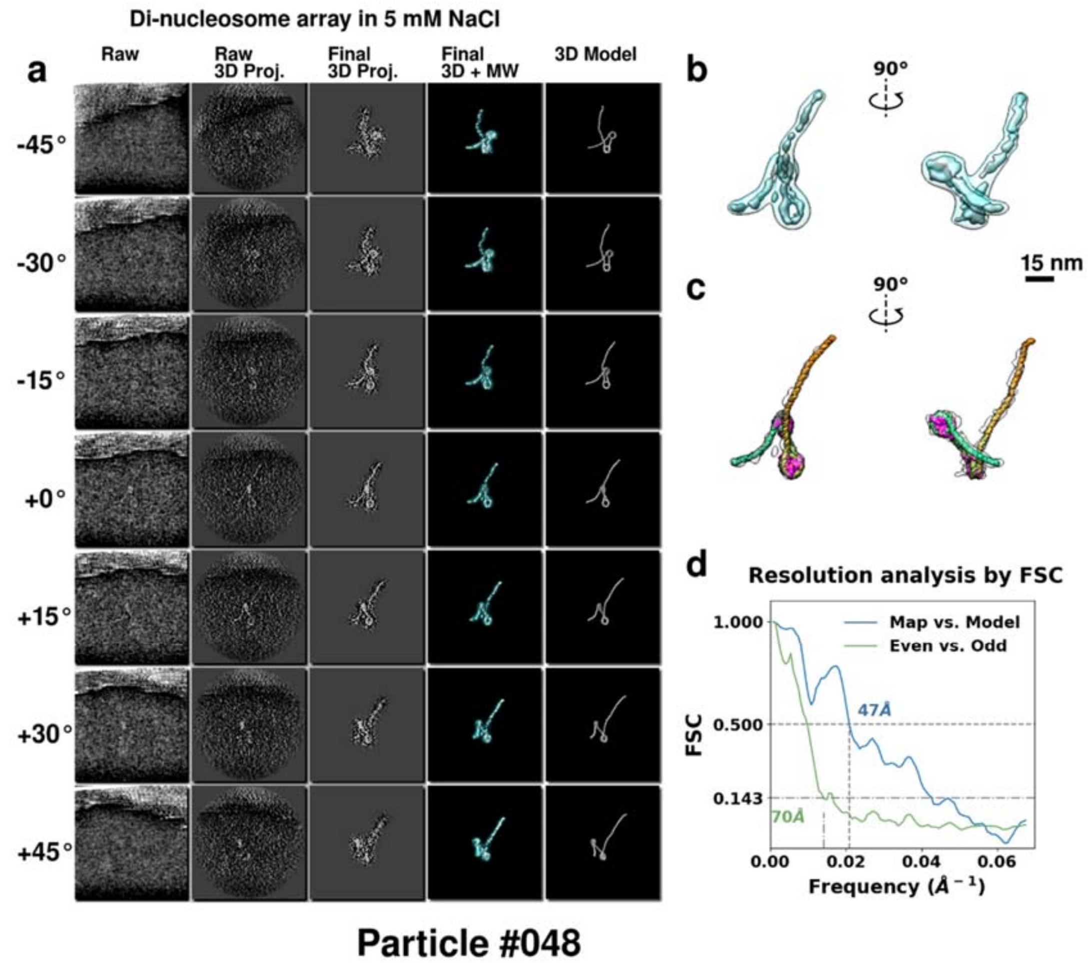

Supplementary Fig. 61. Cryo-ET 3D reconstruction of individual di-nucleosome particle (index no. 48) in 5 mM NaCl. a, IPET 3D reconstruction of individual di-nucleosome particles. The first column shows seven representative tilt images of an individual particle after CTF correction. Through alignment of the tilt images to a common center for 3D reconstruction via iterative refinement, the second and third columns display the 3D projections of the reconstruction before and after particle-shaped masking, respectively. The fourth column shows the final 3D reconstruction with missing wedge correction, and the fifth column presents the flexibly fitted model at the corresponding tilt angles. **b**, Zoomed-in view of the final 3D density map displayed in orthogonal views, shown at two contour levels. **c**, Superimposition of the high contour level map from (b) onto its flexibly fitted model. **d**, Resolution evaluation of the final 3D density map using two criteria: Fourier shell correlation (FSC) between two-half maps reconstructed from the even and odd index of the tilted series and FSC between the final 3D map and the fitted structure model. The resolution for the former and latter criteria is evaluated at frequencies of 0.5 and 0.143, respectively.

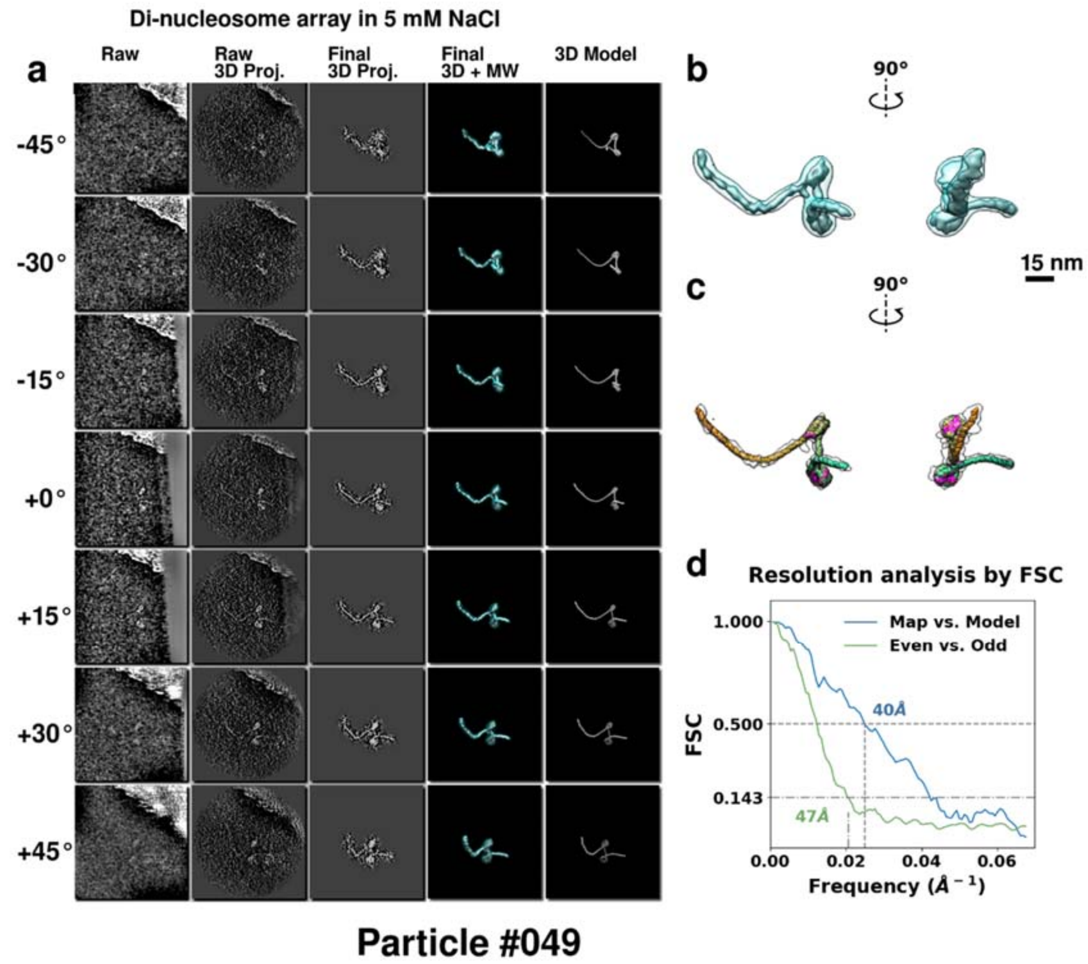

Supplementary Fig. 62. Cryo-ET 3D reconstruction of individual di-nucleosome particle (index no. 49) in 5 mM NaCl. a, IPET 3D reconstruction of individual di-nucleosome particles. The first column shows seven representative tilt images of an individual particle after CTF correction. Through alignment of the tilt images to a common center for 3D reconstruction via iterative refinement, the second and third columns display the 3D projections of the reconstruction before and after particle-shaped masking, respectively. The fourth column shows the final 3D reconstruction with missing wedge correction, and the fifth column presents the flexibly fitted model at the corresponding tilt angles. **b**, Zoomed-in view of the final 3D density map displayed in orthogonal views, shown at two contour levels. **c**, Superimposition of the high contour level map from (b) onto its flexibly fitted model. **d**, Resolution evaluation of the final 3D density map using two criteria: Fourier shell correlation (FSC) between two-half maps reconstructed from the even and odd index of the tilted series and FSC between the final 3D map and the fitted structure model. The resolution for the former and latter criteria is evaluated at frequencies of 0.5 and 0.143, respectively.

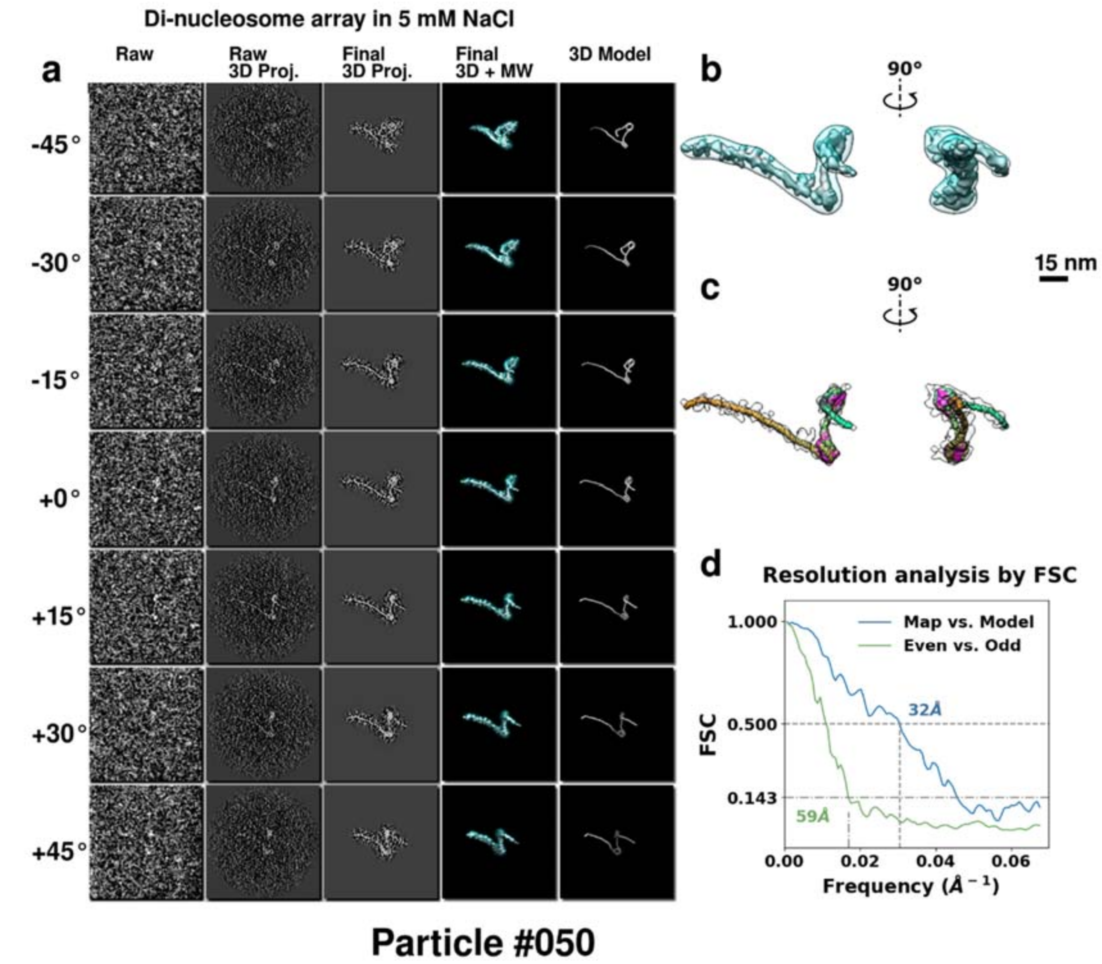

Supplementary Fig. 63. Cryo-ET 3D reconstruction of individual di-nucleosome particle (index no. 50) in 5 mM NaCl. a, IPET 3D reconstruction of individual di-nucleosome particles. The first column shows seven representative tilt images of an individual particle after CTF correction. Through alignment of the tilt images to a common center for 3D reconstruction via iterative refinement, the second and third columns display the 3D projections of the reconstruction before and after particle-shaped masking, respectively. The fourth column shows the final 3D reconstruction with missing wedge correction, and the fifth column presents the flexibly fitted model at the corresponding tilt angles. **b**, Zoomed-in view of the final 3D density map displayed in orthogonal views, shown at two contour levels. **c**, Superimposition of the high contour level map from (b) onto its flexibly fitted model. **d**, Resolution evaluation of the final 3D density map using two criteria: Fourier shell correlation (FSC) between two-half maps reconstructed from the even and odd index of the tilted series and FSC between the final 3D map and the fitted structure model. The resolution for the former and latter criteria is evaluated at frequencies of 0.5 and 0.143, respectively.

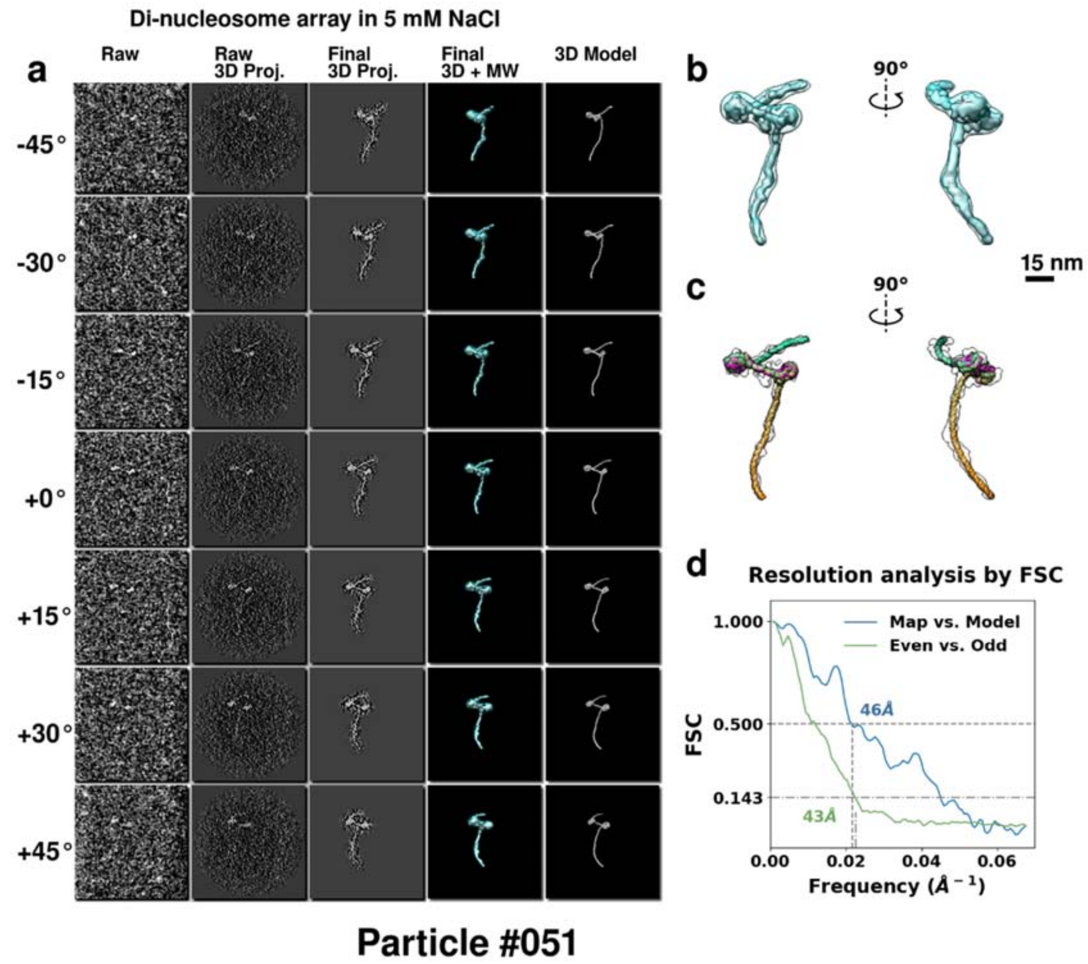

Supplementary Fig. 64. Cryo-ET 3D reconstruction of individual di-nucleosome particle (index no. 51) in 5 mM NaCl. a, IPET 3D reconstruction of individual di-nucleosome particles. The first column shows seven representative tilt images of an individual particle after CTF correction. Through alignment of the tilt images to a common center for 3D reconstruction via iterative refinement, the second and third columns display the 3D projections of the reconstruction before and after particle-shaped masking, respectively. The fourth column shows the final 3D reconstruction with missing wedge correction, and the fifth column presents the flexibly fitted model at the corresponding tilt angles. **b**, Zoomed-in view of the final 3D density map displayed in orthogonal views, shown at two contour levels. **c**, Superimposition of the high contour level map from (b) onto its flexibly fitted model. **d**, Resolution evaluation of the final 3D density map using two criteria: Fourier shell correlation (FSC) between two-half maps reconstructed from the even and odd index of the tilted series and FSC between the final 3D map and the fitted structure model. The resolution for the former and latter criteria is evaluated at frequencies of 0.5 and 0.143, respectively.

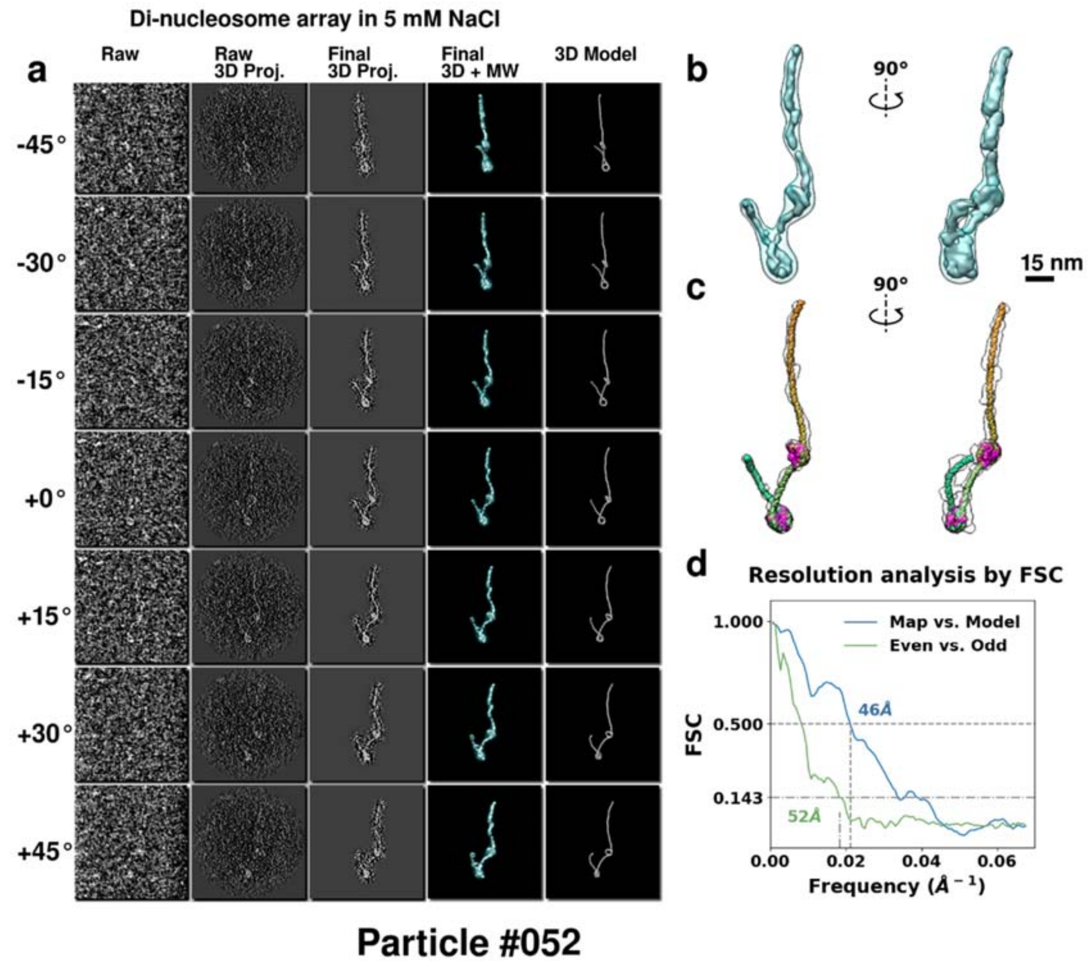

Supplementary Fig. 65. Cryo-ET 3D reconstruction of individual di-nucleosome particle (index no. 52) in 5 mM NaCl. **a**, IPET 3D reconstruction of individual di-nucleosome particles. The first column shows seven representative tilt images of an individual particle after CTF correction. Through alignment of the tilt images to a common center for 3D reconstruction via iterative refinement, the second and third columns display the 3D projections of the reconstruction before and after particle-shaped masking, respectively. The fourth column shows the final 3D reconstruction with missing wedge correction, and the fifth column presents the flexibly fitted model at the corresponding tilt angles. **b**, Zoomed-in view of the final 3D density map displayed in orthogonal views, shown at two contour levels. **c**, Superimposition of the high contour level map from (b) onto its flexibly fitted model. **d**, Resolution evaluation of the final 3D density map using two criteria: Fourier shell correlation (FSC) between two-half maps reconstructed from the even and odd index of the tilted series and FSC between the final 3D map and the fitted structure model. The resolution for the former and latter criteria is evaluated at frequencies of 0.5 and 0.143, respectively.

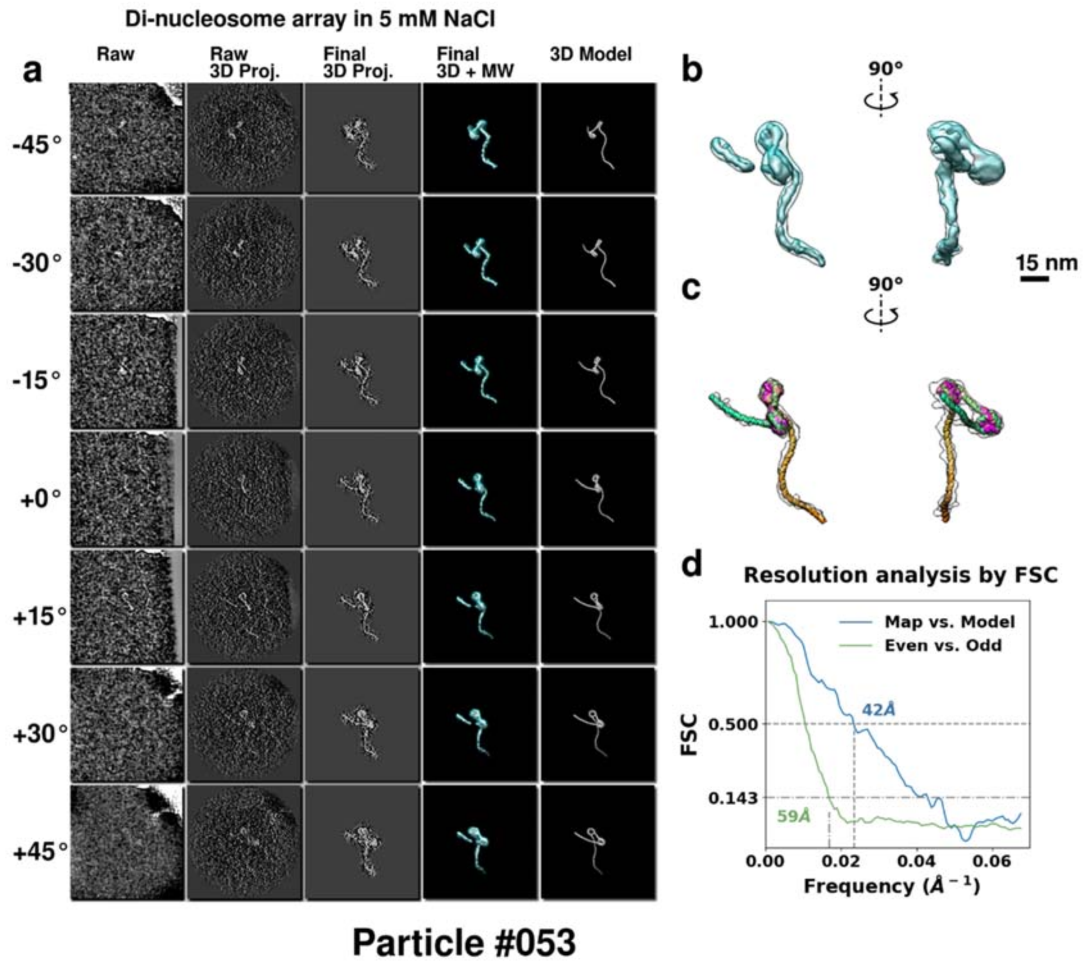

Supplementary Fig. 66. Cryo-ET 3D reconstruction of individual di-nucleosome particle (index no. 53) in 5 mM NaCl. a, IPET 3D reconstruction of individual di-nucleosome particles. The first column shows seven representative tilt images of an individual particle after CTF correction. Through alignment of the tilt images to a common center for 3D reconstruction via iterative refinement, the second and third columns display the 3D projections of the reconstruction before and after particle-shaped masking, respectively. The fourth column shows the final 3D reconstruction with missing wedge correction, and the fifth column presents the flexibly fitted model at the corresponding tilt angles. **b**, Zoomed-in view of the final 3D density map displayed in orthogonal views, shown at two contour levels. **c**, Superimposition of the high contour level map from (b) onto its flexibly fitted model. **d**, Resolution evaluation of the final 3D density map using two criteria: Fourier shell correlation (FSC) between two-half maps reconstructed from the even and odd index of the tilted series and FSC between the final 3D map and the fitted structure model. The resolution for the former and latter criteria is evaluated at frequencies of 0.5 and 0.143, respectively.

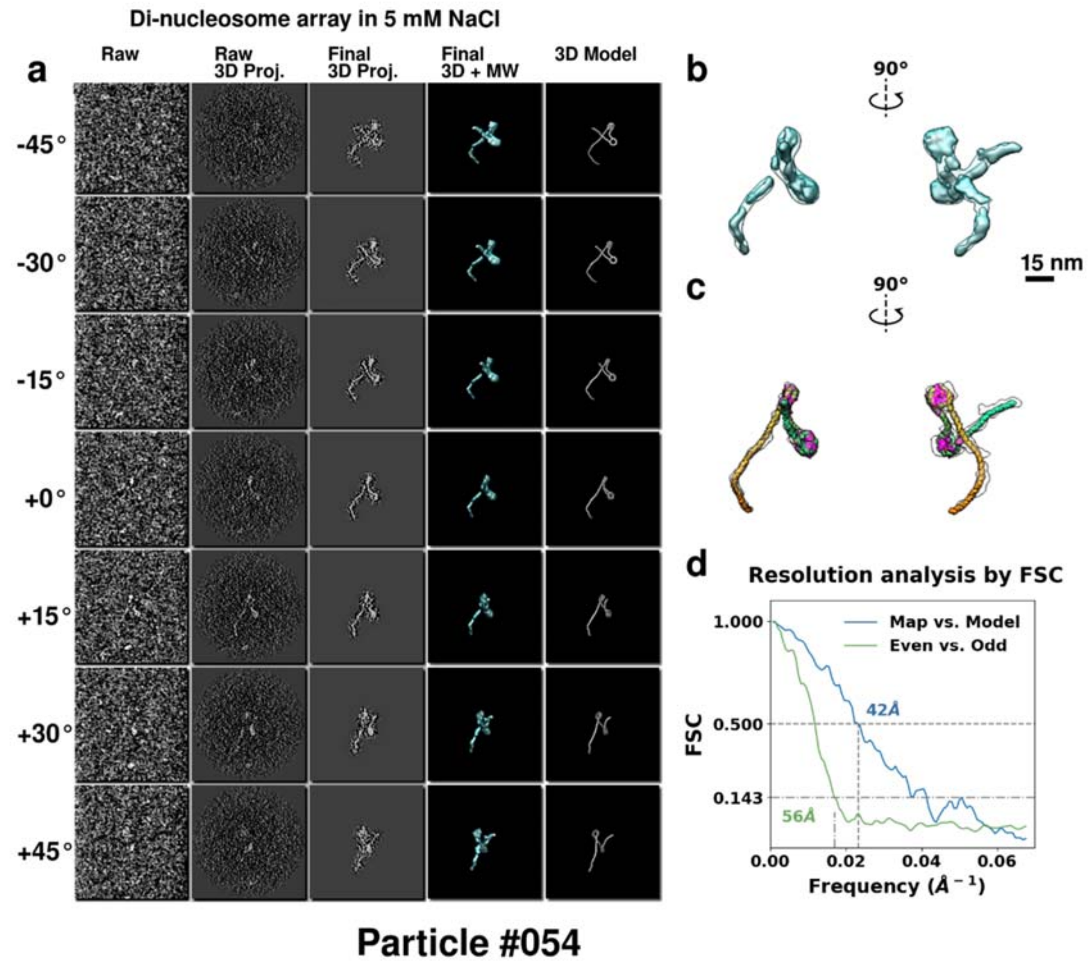

Supplementary Fig. 67. Cryo-ET 3D reconstruction of individual di-nucleosome particle (index no. 54) in 5 mM NaCl. a, IPET 3D reconstruction of individual di-nucleosome particles. The first column shows seven representative tilt images of an individual particle after CTF correction. Through alignment of the tilt images to a common center for 3D reconstruction via iterative refinement, the second and third columns display the 3D projections of the reconstruction before and after particle-shaped masking, respectively. The fourth column shows the final 3D reconstruction with missing wedge correction, and the fifth column presents the flexibly fitted model at the corresponding tilt angles. **b**, Zoomed-in view of the final 3D density map displayed in orthogonal views, shown at two contour levels. **c**, Superimposition of the high contour level map from (b) onto its flexibly fitted model. **d**, Resolution evaluation of the final 3D density map using two criteria: Fourier shell correlation (FSC) between two-half maps reconstructed from the even and odd index of the tilted series and FSC between the final 3D map and the fitted structure model. The resolution for the former and latter criteria is evaluated at frequencies of 0.5 and 0.143, respectively.

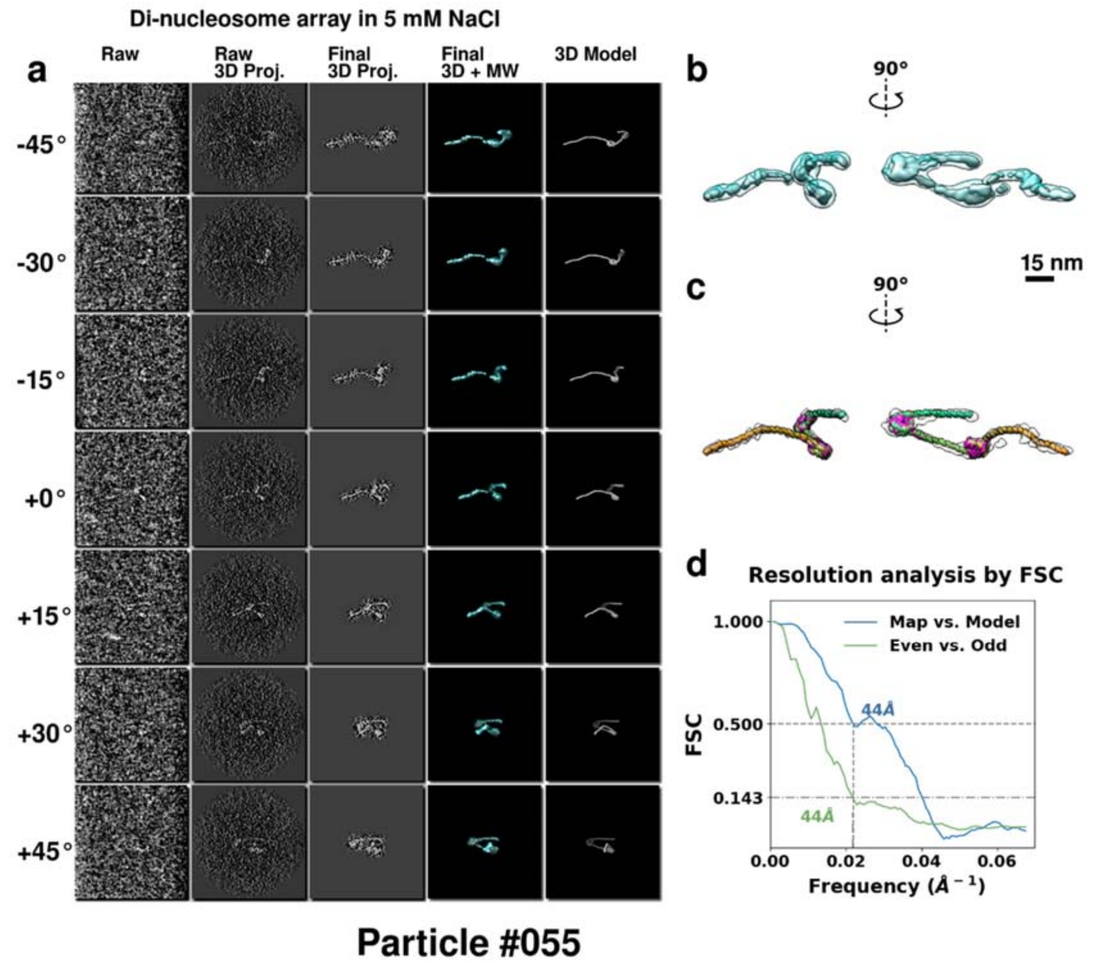

Supplementary Fig. 68. Cryo-ET 3D reconstruction of individual di-nucleosome particle (index no. 55) in 5 mM NaCl. a, IPET 3D reconstruction of individual di-nucleosome particles. The first column shows seven representative tilt images of an individual particle after CTF correction. Through alignment of the tilt images to a common center for 3D reconstruction via iterative refinement, the second and third columns display the 3D projections of the reconstruction before and after particle-shaped masking, respectively. The fourth column shows the final 3D reconstruction with missing wedge correction, and the fifth column presents the flexibly fitted model at the corresponding tilt angles. **b**, Zoomed-in view of the final 3D density map displayed in orthogonal views, shown at two contour levels. **c**, Superimposition of the high contour level map from (b) onto its flexibly fitted model. **d**, Resolution evaluation of the final 3D density map using two criteria: Fourier shell correlation (FSC) between two-half maps reconstructed from the even and odd index of the tilted series and FSC between the final 3D map and the fitted structure model. The resolution for the former and latter criteria is evaluated at frequencies of 0.5 and 0.143, respectively.

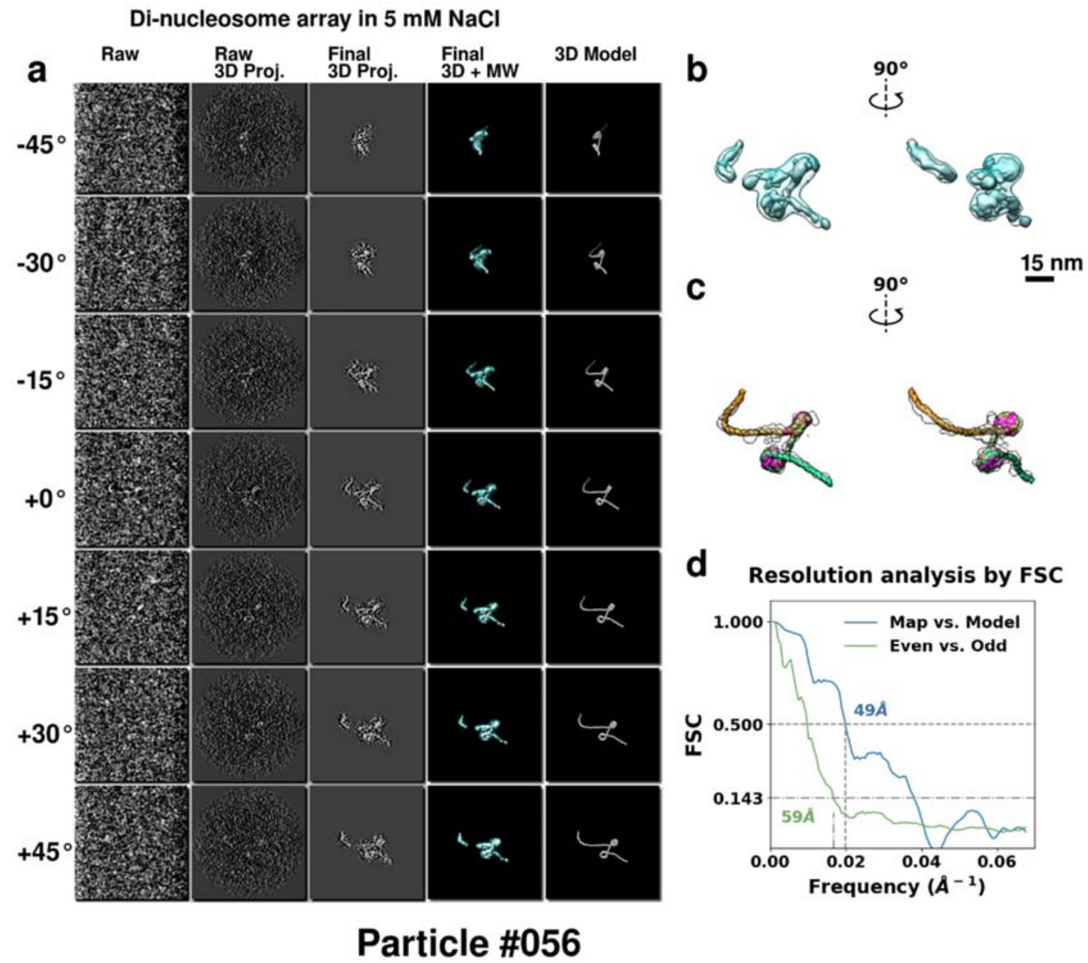

Supplementary Fig. 69. Cryo-ET 3D reconstruction of individual di-nucleosome particle (index no. 56) in 5 mM NaCl. a, IPET 3D reconstruction of individual di-nucleosome particles. The first column shows seven representative tilt images of an individual particle after CTF correction. Through alignment of the tilt images to a common center for 3D reconstruction via iterative refinement, the second and third columns display the 3D projections of the reconstruction before and after particle-shaped masking, respectively. The fourth column shows the final 3D reconstruction with missing wedge correction, and the fifth column presents the flexibly fitted model at the corresponding tilt angles. **b**, Zoomed-in view of the final 3D density map displayed in orthogonal views, shown at two contour levels. **c**, Superimposition of the high contour level map from (b) onto its flexibly fitted model. **d**, Resolution evaluation of the final 3D density map using two criteria: Fourier shell correlation (FSC) between two-half maps reconstructed from the even and odd index of the tilted series and FSC between the final 3D map and the fitted structure model. The resolution for the former and latter criteria is evaluated at frequencies of 0.5 and 0.143, respectively.

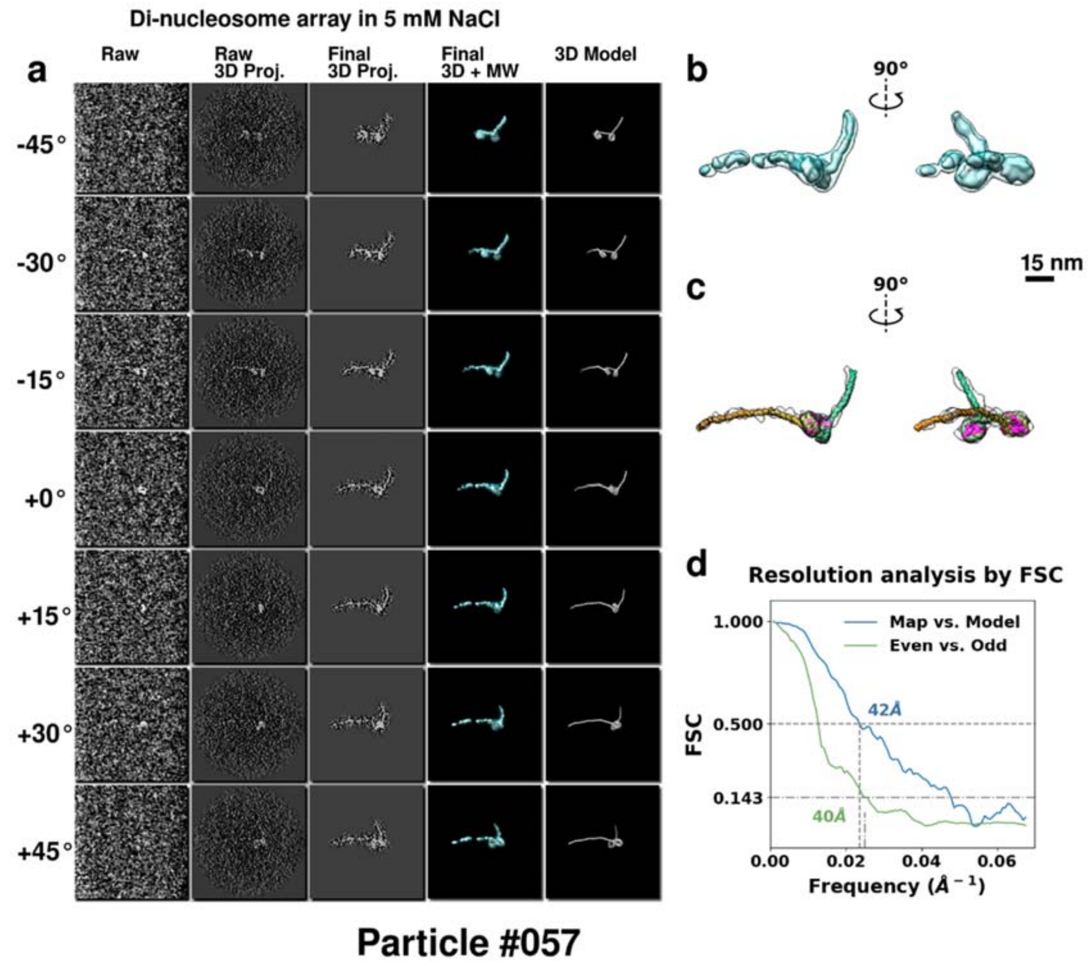

Supplementary Fig. 70. Cryo-ET 3D reconstruction of individual di-nucleosome particle (index no. 57) in 5 mM NaCl. a, IPET 3D reconstruction of individual di-nucleosome particles. The first column shows seven representative tilt images of an individual particle after CTF correction. Through alignment of the tilt images to a common center for 3D reconstruction via iterative refinement, the second and third columns display the 3D projections of the reconstruction before and after particle-shaped masking, respectively. The fourth column shows the final 3D reconstruction with missing wedge correction, and the fifth column presents the flexibly fitted model at the corresponding tilt angles. **b**, Zoomed-in view of the final 3D density map displayed in orthogonal views, shown at two contour levels. **c**, Superimposition of the high contour level map from (b) onto its flexibly fitted model. **d**, Resolution evaluation of the final 3D density map using two criteria: Fourier shell correlation (FSC) between two-half maps reconstructed from the even and odd index of the tilted series and FSC between the final 3D map and the fitted structure model. The resolution for the former and latter criteria is evaluated at frequencies of 0.5 and 0.143, respectively.

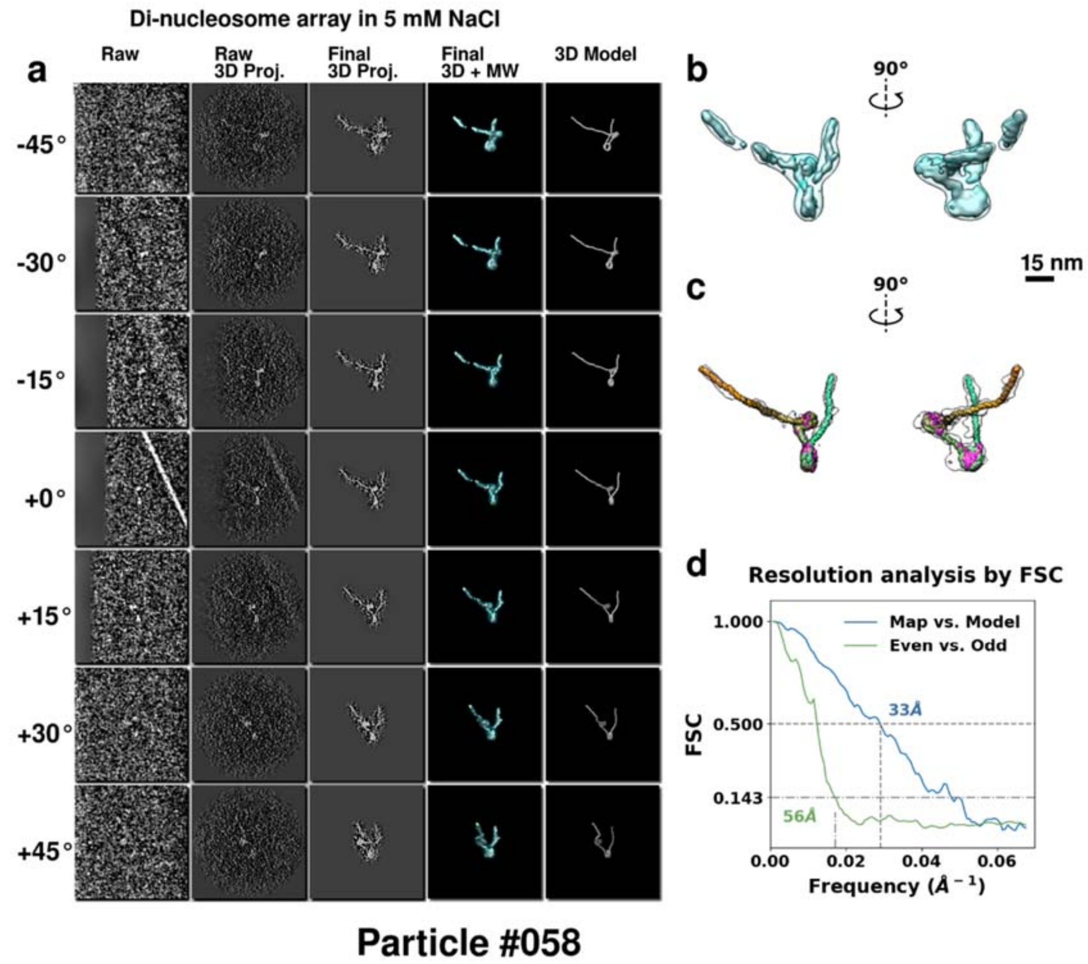

Supplementary Fig. 71. Cryo-ET 3D reconstruction of individual di-nucleosome particle (index no. 58) in 5 mM NaCl. **a**, IPET 3D reconstruction of individual di-nucleosome particles. The first column shows seven representative tilt images of an individual particle after CTF correction. Through alignment of the tilt images to a common center for 3D reconstruction via iterative refinement, the second and third columns display the 3D projections of the reconstruction before and after particle-shaped masking, respectively. The fourth column shows the final 3D reconstruction with missing wedge correction, and the fifth column presents the flexibly fitted model at the corresponding tilt angles. **b**, Zoomed-in view of the final 3D density map displayed in orthogonal views, shown at two contour levels. **c**, Superimposition of the high contour level map from (b) onto its flexibly fitted model. **d**, Resolution evaluation of the final 3D density map using two criteria: Fourier shell correlation (FSC) between two-half maps reconstructed from the even and odd index of the tilted series and FSC between the final 3D map and the fitted structure model. The resolution for the former and latter criteria is evaluated at frequencies of 0.5 and 0.143, respectively.

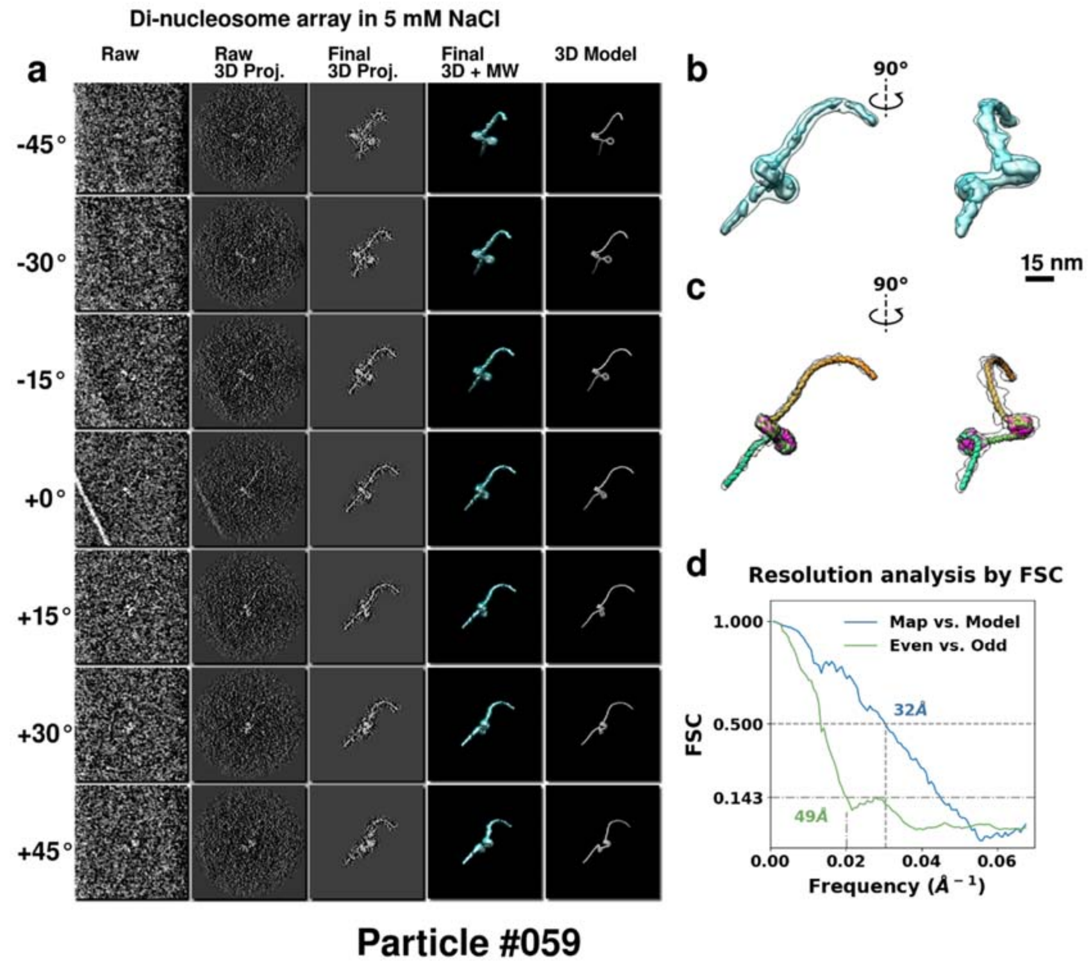

Supplementary Fig. 72. Cryo-ET 3D reconstruction of individual di-nucleosome particle (index no. 59) in 5 mM NaCl. a, IPET 3D reconstruction of individual di-nucleosome particles. The first column shows seven representative tilt images of an individual particle after CTF correction. Through alignment of the tilt images to a common center for 3D reconstruction via iterative refinement, the second and third columns display the 3D projections of the reconstruction before and after particle-shaped masking, respectively. The fourth column shows the final 3D reconstruction with missing wedge correction, and the fifth column presents the flexibly fitted model at the corresponding tilt angles. **b**, Zoomed-in view of the final 3D density map displayed in orthogonal views, shown at two contour levels. **c**, Superimposition of the high contour level map from (b) onto its flexibly fitted model. **d**, Resolution evaluation of the final 3D density map using two criteria: Fourier shell correlation (FSC) between two-half maps reconstructed from the even and odd index of the tilted series and FSC between the final 3D map and the fitted structure model. The resolution for the former and latter criteria is evaluated at frequencies of 0.5 and 0.143, respectively.

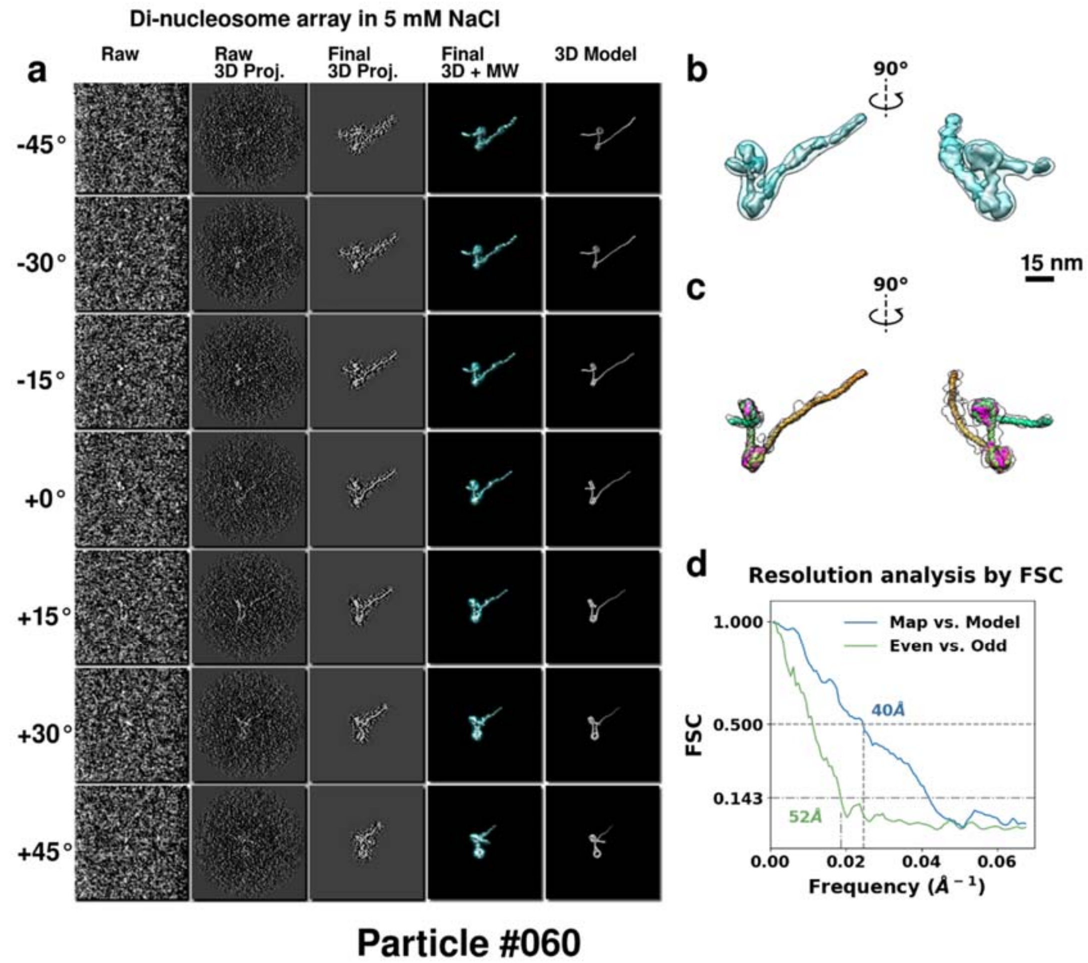

Supplementary Fig. 73. Cryo-ET 3D reconstruction of individual di-nucleosome particle (index no. 60) in 5 mM NaCl. a, IPET 3D reconstruction of individual di-nucleosome particles. The first column shows seven representative tilt images of an individual particle after CTF correction. Through alignment of the tilt images to a common center for 3D reconstruction via iterative refinement, the second and third columns display the 3D projections of the reconstruction before and after particle-shaped masking, respectively. The fourth column shows the final 3D reconstruction with missing wedge correction, and the fifth column presents the flexibly fitted model at the corresponding tilt angles. **b**, Zoomed-in view of the final 3D density map displayed in orthogonal views, shown at two contour levels. **c**, Superimposition of the high contour level map from (b) onto its flexibly fitted model. **d**, Resolution evaluation of the final 3D density map using two criteria: Fourier shell correlation (FSC) between two-half maps reconstructed from the even and odd index of the tilted series and FSC between the final 3D map and the fitted structure model. The resolution for the former and latter criteria is evaluated at frequencies of 0.5 and 0.143, respectively.

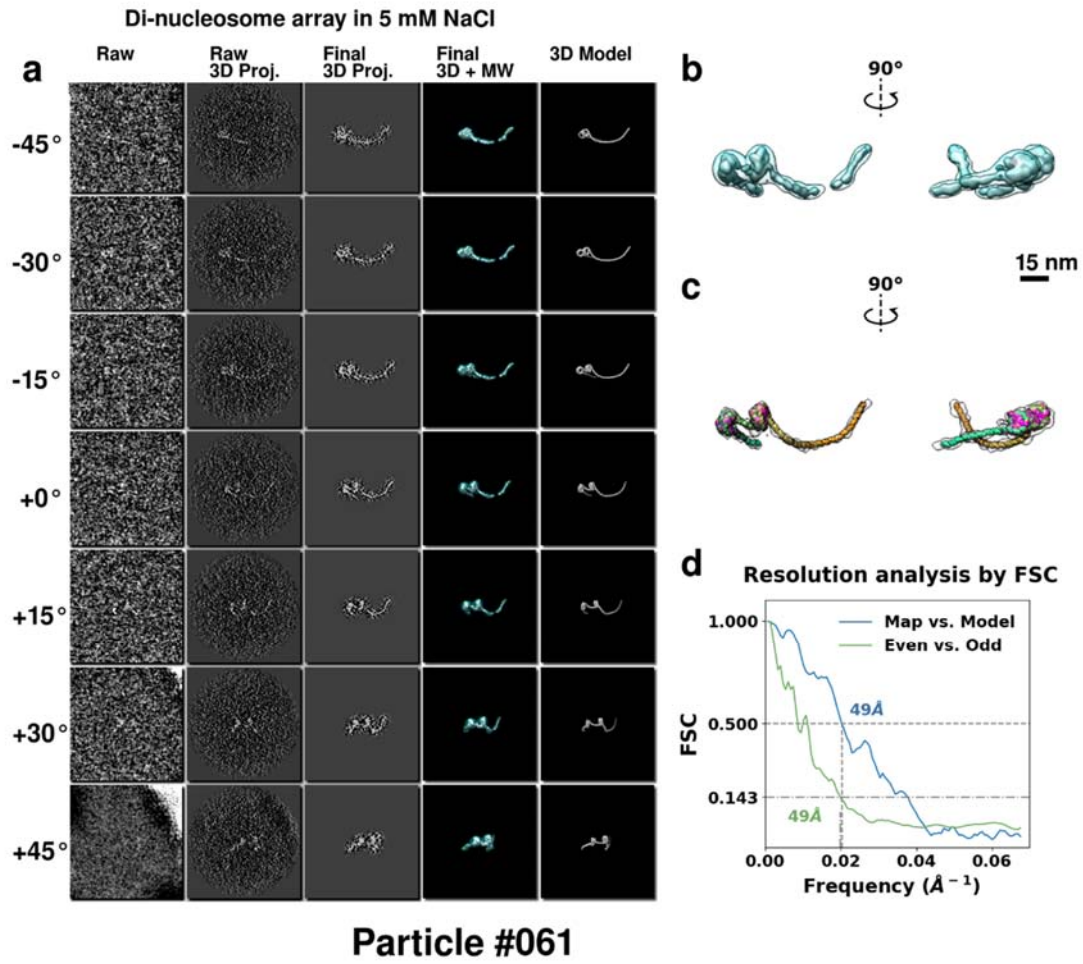

Supplementary Fig. 74. Cryo-ET 3D reconstruction of individual di-nucleosome particle (index no. 61) in 5 mM NaCl. a, IPET 3D reconstruction of individual di-nucleosome particles. The first column shows seven representative tilt images of an individual particle after CTF correction. Through alignment of the tilt images to a common center for 3D reconstruction via iterative refinement, the second and third columns display the 3D projections of the reconstruction before and after particle-shaped masking, respectively. The fourth column shows the final 3D reconstruction with missing wedge correction, and the fifth column presents the flexibly fitted model at the corresponding tilt angles. **b**, Zoomed-in view of the final 3D density map displayed in orthogonal views, shown at two contour levels. **c**, Superimposition of the high contour level map from (b) onto its flexibly fitted model. **d**, Resolution evaluation of the final 3D density map using two criteria: Fourier shell correlation (FSC) between two-half maps reconstructed from the even and odd index of the tilted series and FSC between the final 3D map and the fitted structure model. The resolution for the former and latter criteria is evaluated at frequencies of 0.5 and 0.143, respectively.

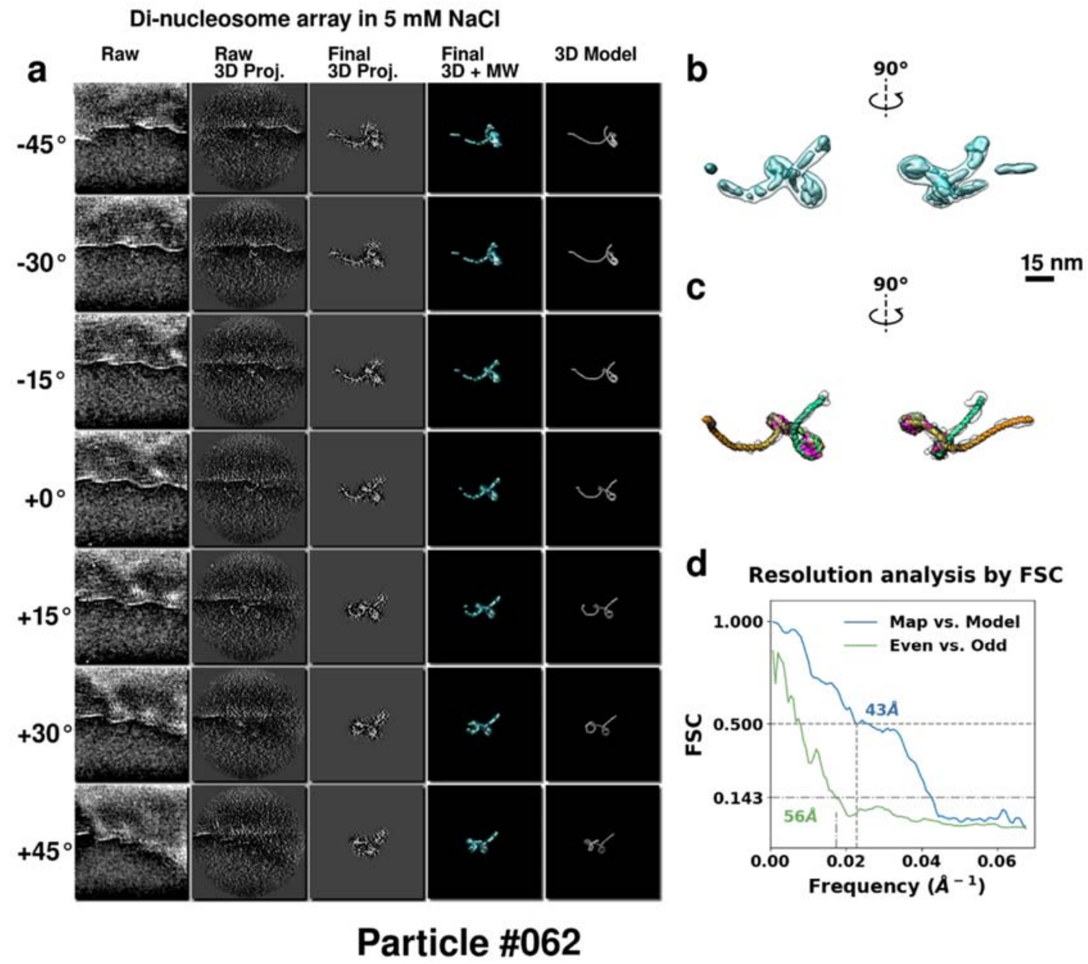

Supplementary Fig. 75. Cryo-ET 3D reconstruction of individual di-nucleosome particle (index no. 62) in 5 mM NaCl. a, IPET 3D reconstruction of individual di-nucleosome particles. The first column shows seven representative tilt images of an individual particle after CTF correction. Through alignment of the tilt images to a common center for 3D reconstruction via iterative refinement, the second and third columns display the 3D projections of the reconstruction before and after particle-shaped masking, respectively. The fourth column shows the final 3D reconstruction with missing wedge correction, and the fifth column presents the flexibly fitted model at the corresponding tilt angles. **b**, Zoomed-in view of the final 3D density map displayed in orthogonal views, shown at two contour levels. **c**, Superimposition of the high contour level map from (b) onto its flexibly fitted model. **d**, Resolution evaluation of the final 3D density map using two criteria: Fourier shell correlation (FSC) between two-half maps reconstructed from the even and odd index of the tilted series and FSC between the final 3D map and the fitted structure model. The resolution for the former and latter criteria is evaluated at frequencies of 0.5 and 0.143, respectively.

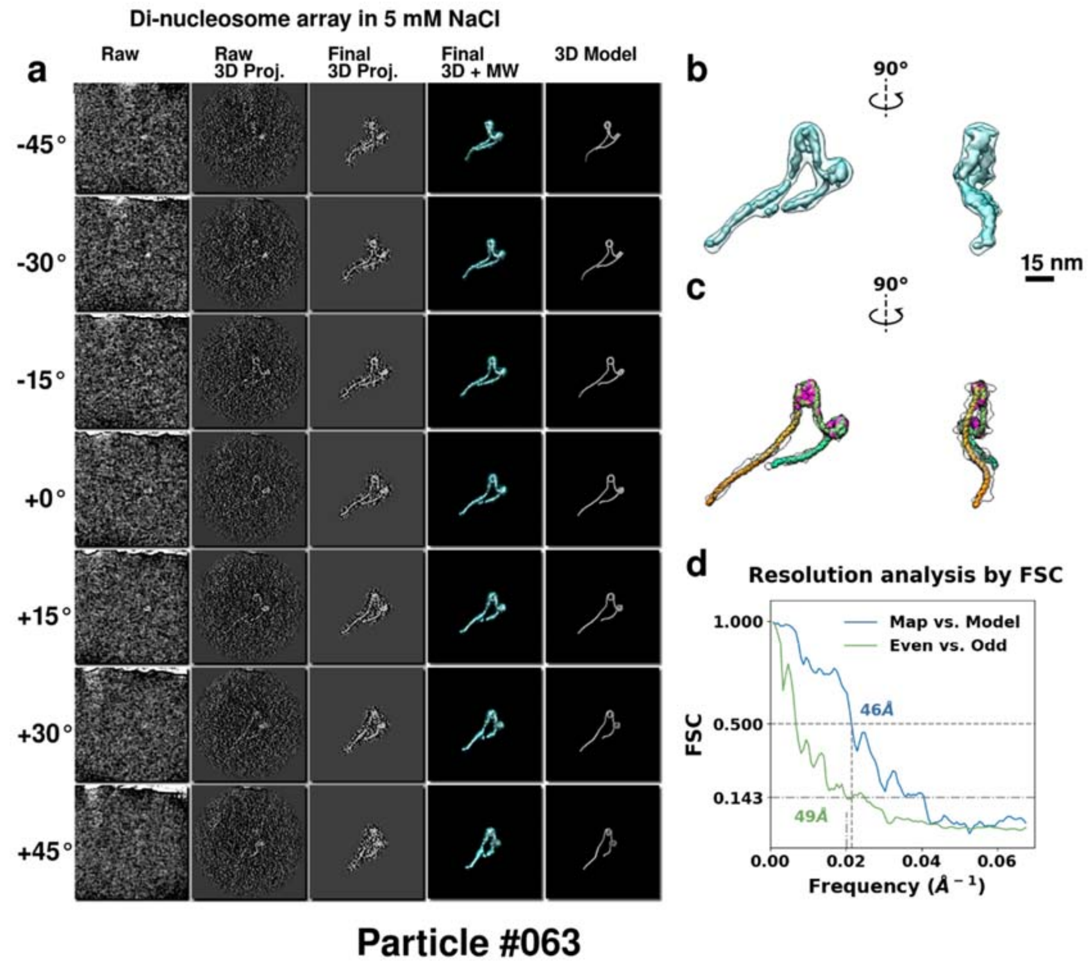

Supplementary Fig. 76. Cryo-ET 3D reconstruction of individual di-nucleosome particle (index no. 63) in 5 mM NaCl. a, IPET 3D reconstruction of individual di-nucleosome particles. The first column shows seven representative tilt images of an individual particle after CTF correction. Through alignment of the tilt images to a common center for 3D reconstruction via iterative refinement, the second and third columns display the 3D projections of the reconstruction before and after particle-shaped masking, respectively. The fourth column shows the final 3D reconstruction with missing wedge correction, and the fifth column presents the flexibly fitted model at the corresponding tilt angles. **b**, Zoomed-in view of the final 3D density map displayed in orthogonal views, shown at two contour levels. **c**, Superimposition of the high contour level map from (b) onto its flexibly fitted model. **d**, Resolution evaluation of the final 3D density map using two criteria: Fourier shell correlation (FSC) between two-half maps reconstructed from the even and odd index of the tilted series and FSC between the final 3D map and the fitted structure model. The resolution for the former and latter criteria is evaluated at frequencies of 0.5 and 0.143, respectively.

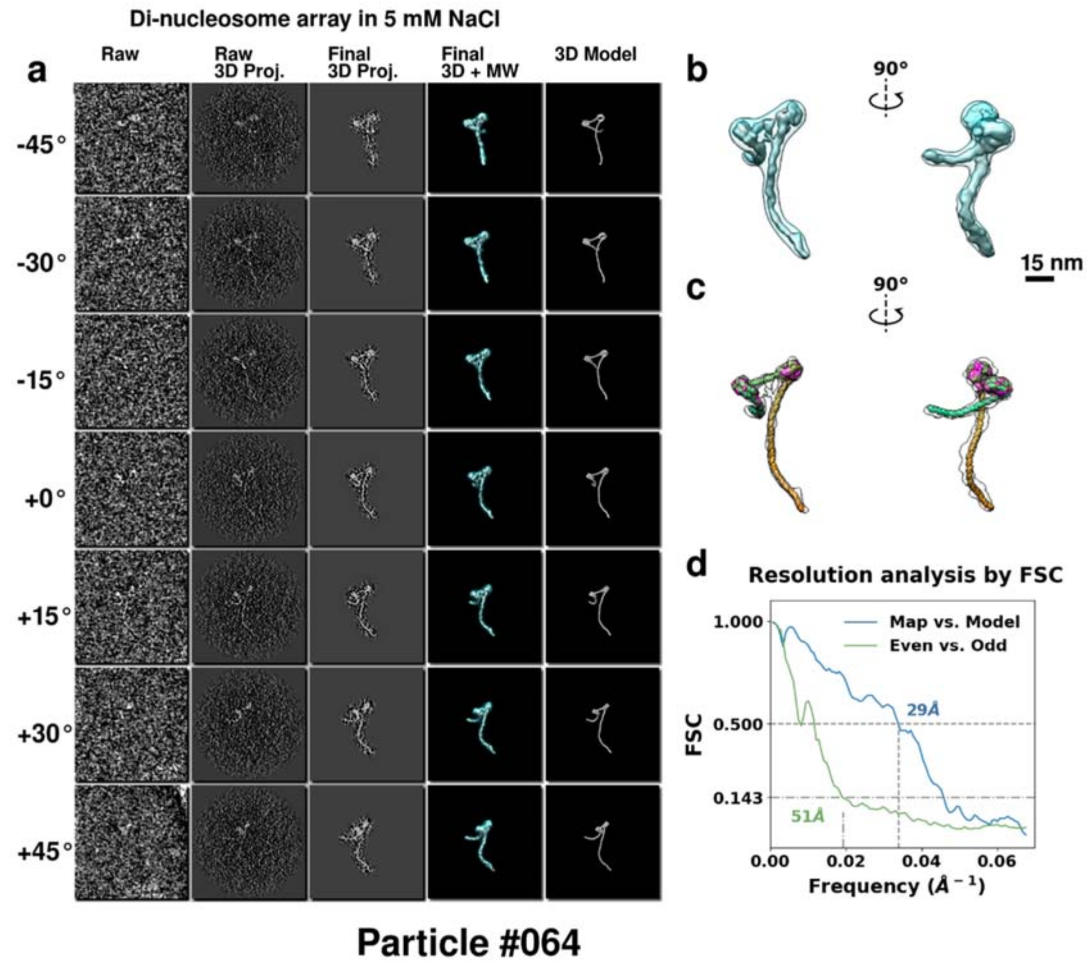

Supplementary Fig. 77. Cryo-ET 3D reconstruction of individual di-nucleosome particle (index no. 64) in 5 mM NaCl. a, IPET 3D reconstruction of individual di-nucleosome particles. The first column shows seven representative tilt images of an individual particle after CTF correction. Through alignment of the tilt images to a common center for 3D reconstruction via iterative refinement, the second and third columns display the 3D projections of the reconstruction before and after particle-shaped masking, respectively. The fourth column shows the final 3D reconstruction with missing wedge correction, and the fifth column presents the flexibly fitted model at the corresponding tilt angles. **b**, Zoomed-in view of the final 3D density map displayed in orthogonal views, shown at two contour levels. **c**, Superimposition of the high contour level map from (b) onto its flexibly fitted model. **d**, Resolution evaluation of the final 3D density map using two criteria: Fourier shell correlation (FSC) between two-half maps reconstructed from the even and odd index of the tilted series and FSC between the final 3D map and the fitted structure model. The resolution for the former and latter criteria is evaluated at frequencies of 0.5 and 0.143, respectively.

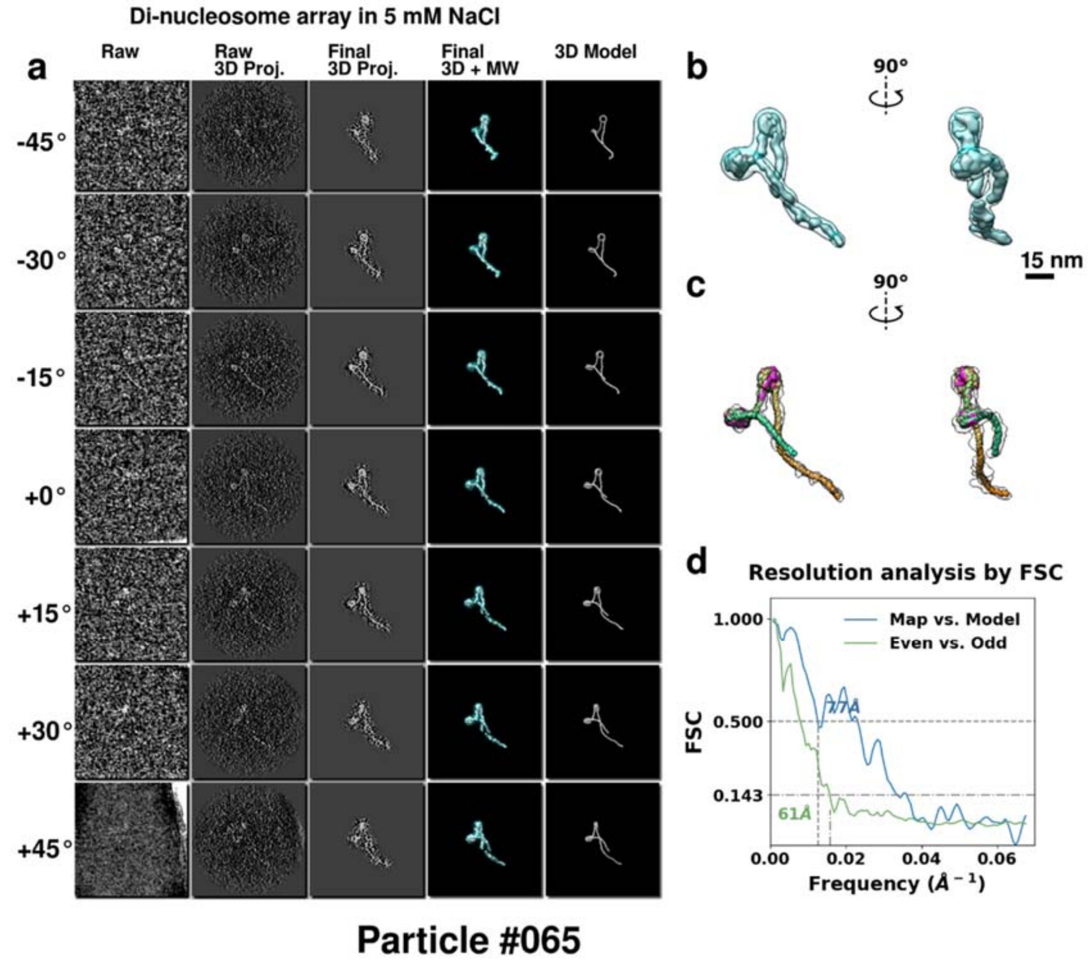

Supplementary Fig. 78. Cryo-ET 3D reconstruction of individual di-nucleosome particle (index no. 65) in 5 mM NaCl. a, IPET 3D reconstruction of individual di-nucleosome particles. The first column shows seven representative tilt images of an individual particle after CTF correction. Through alignment of the tilt images to a common center for 3D reconstruction via iterative refinement, the second and third columns display the 3D projections of the reconstruction before and after particle-shaped masking, respectively. The fourth column shows the final 3D reconstruction with missing wedge correction, and the fifth column presents the flexibly fitted model at the corresponding tilt angles. **b**, Zoomed-in view of the final 3D density map displayed in orthogonal views, shown at two contour levels. **c**, Superimposition of the high contour level map from (b) onto its flexibly fitted model. **d**, Resolution evaluation of the final 3D density map using two criteria: Fourier shell correlation (FSC) between two-half maps reconstructed from the even and odd index of the tilted series and FSC between the final 3D map and the fitted structure model. The resolution for the former and latter criteria is evaluated at frequencies of 0.5 and 0.143, respectively.

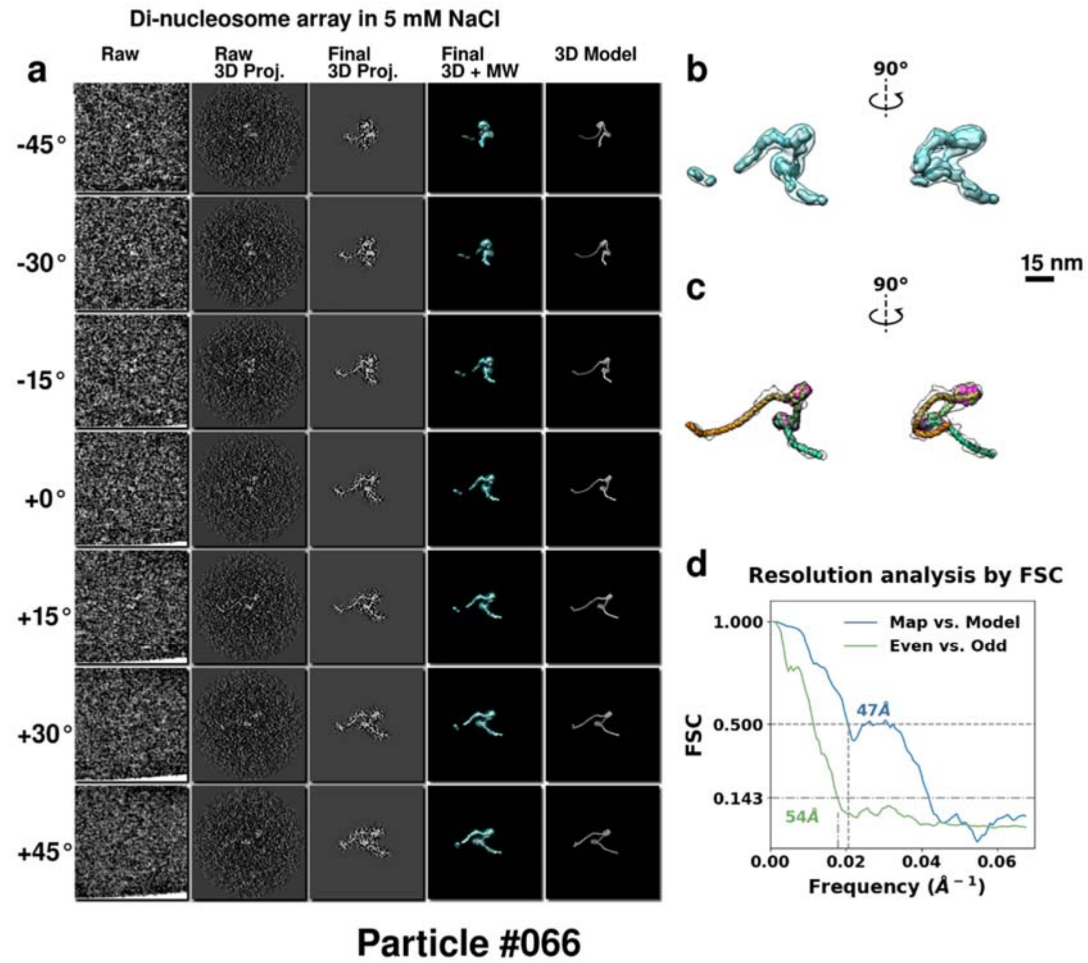

Supplementary Fig. 79. Cryo-ET 3D reconstruction of individual di-nucleosome particle (index no. 66) in 5 mM NaCl. a, IPET 3D reconstruction of individual di-nucleosome particles. The first column shows seven representative tilt images of an individual particle after CTF correction. Through alignment of the tilt images to a common center for 3D reconstruction via iterative refinement, the second and third columns display the 3D projections of the reconstruction before and after particle-shaped masking, respectively. The fourth column shows the final 3D reconstruction with missing wedge correction, and the fifth column presents the flexibly fitted model at the corresponding tilt angles. **b**, Zoomed-in view of the final 3D density map displayed in orthogonal views, shown at two contour levels. **c**, Superimposition of the high contour level map from (b) onto its flexibly fitted model. **d**, Resolution evaluation of the final 3D density map using two criteria: Fourier shell correlation (FSC) between two-half maps reconstructed from the even and odd index of the tilted series and FSC between the final 3D map and the fitted structure model. The resolution for the former and latter criteria is evaluated at frequencies of 0.5 and 0.143, respectively.

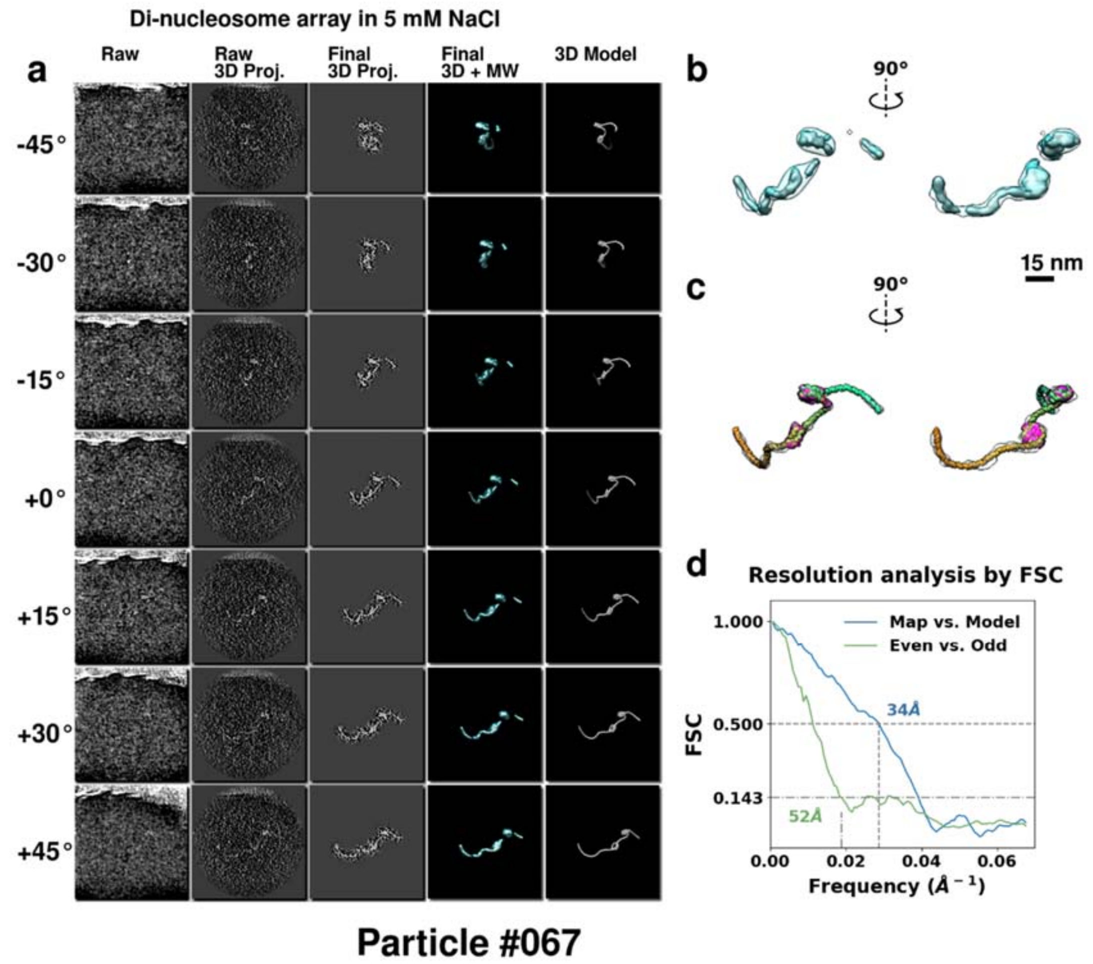

Supplementary Fig. 80. Cryo-ET 3D reconstruction of individual di-nucleosome particle (index no. 67) in 5 mM NaCl. a, IPET 3D reconstruction of individual di-nucleosome particles. The first column shows seven representative tilt images of an individual particle after CTF correction. Through alignment of the tilt images to a common center for 3D reconstruction via iterative refinement, the second and third columns display the 3D projections of the reconstruction before and after particle-shaped masking, respectively. The fourth column shows the final 3D reconstruction with missing wedge correction, and the fifth column presents the flexibly fitted model at the corresponding tilt angles. **b**, Zoomed-in view of the final 3D density map displayed in orthogonal views, shown at two contour levels. **c**, Superimposition of the high contour level map from (b) onto its flexibly fitted model. **d**, Resolution evaluation of the final 3D density map using two criteria: Fourier shell correlation (FSC) between two-half maps reconstructed from the even and odd index of the tilted series and FSC between the final 3D map and the fitted structure model. The resolution for the former and latter criteria is evaluated at frequencies of 0.5 and 0.143, respectively.

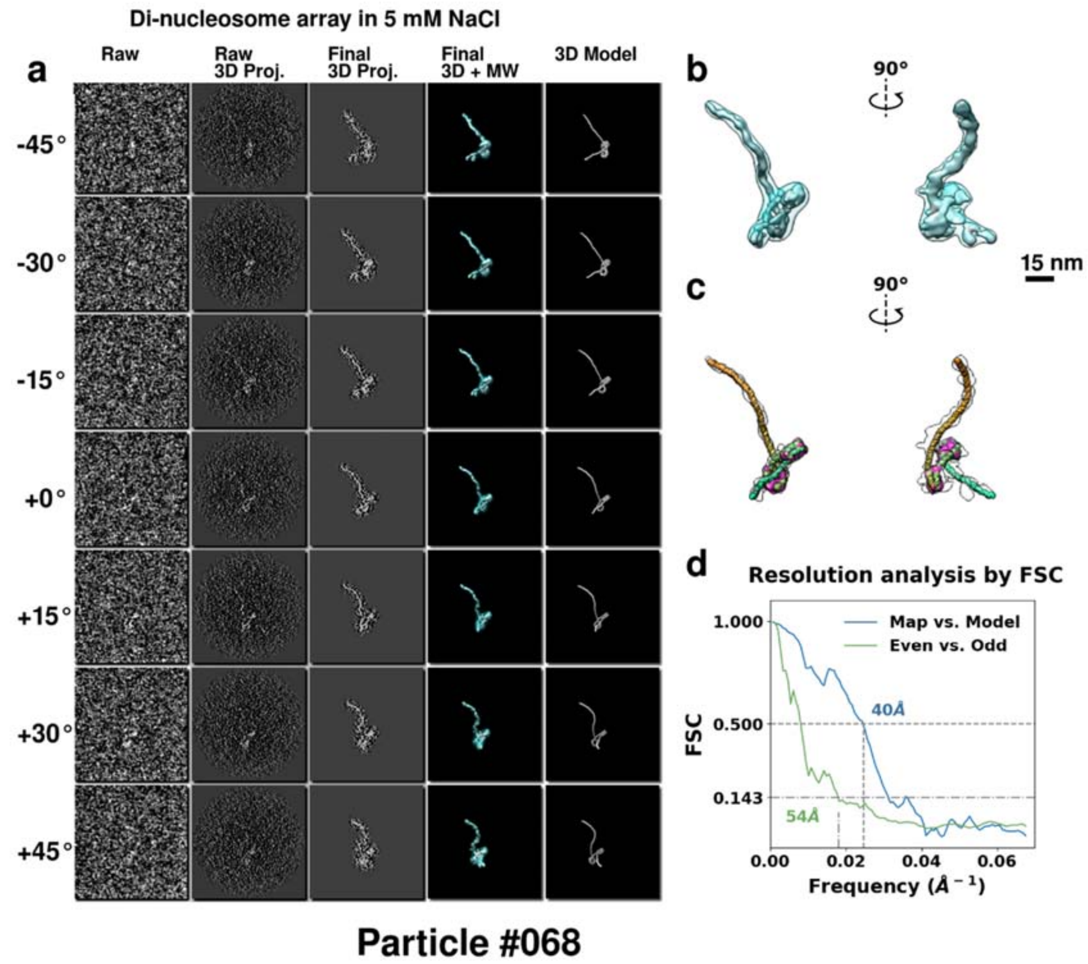

Supplementary Fig. 81. Cryo-ET 3D reconstruction of individual di-nucleosome particle (index no. 68) in 5 mM NaCl. a, IPET 3D reconstruction of individual di-nucleosome particles. The first column shows seven representative tilt images of an individual particle after CTF correction. Through alignment of the tilt images to a common center for 3D reconstruction via iterative refinement, the second and third columns display the 3D projections of the reconstruction before and after particle-shaped masking, respectively. The fourth column shows the final 3D reconstruction with missing wedge correction, and the fifth column presents the flexibly fitted model at the corresponding tilt angles. **b**, Zoomed-in view of the final 3D density map displayed in orthogonal views, shown at two contour levels. **c**, Superimposition of the high contour level map from (b) onto its flexibly fitted model. **d**, Resolution evaluation of the final 3D density map using two criteria: Fourier shell correlation (FSC) between two-half maps reconstructed from the even and odd index of the tilted series and FSC between the final 3D map and the fitted structure model. The resolution for the former and latter criteria is evaluated at frequencies of 0.5 and 0.143, respectively.

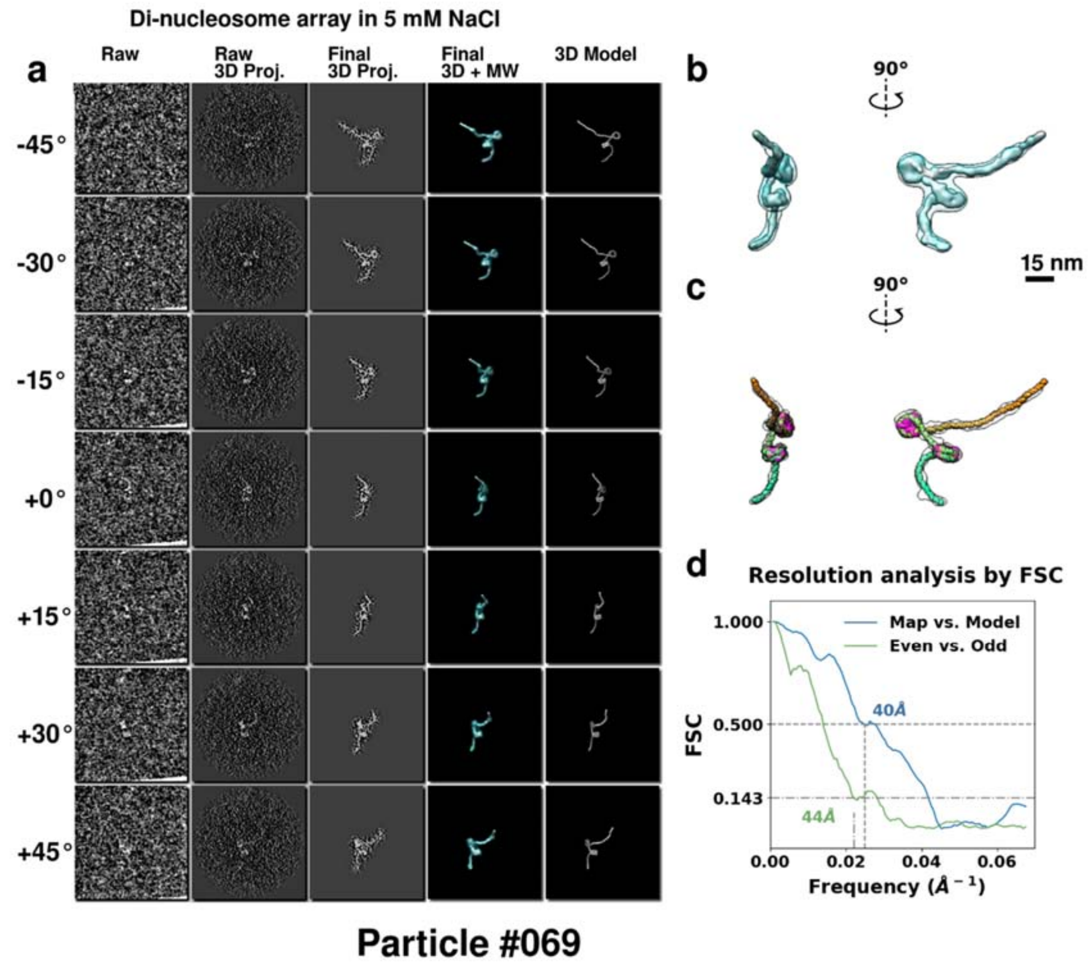

Supplementary Fig. 82. Cryo-ET 3D reconstruction of individual di-nucleosome particle (index no. 69) in 5 mM NaCl. a, IPET 3D reconstruction of individual di-nucleosome particles. The first column shows seven representative tilt images of an individual particle after CTF correction. Through alignment of the tilt images to a common center for 3D reconstruction via iterative refinement, the second and third columns display the 3D projections of the reconstruction before and after particle-shaped masking, respectively. The fourth column shows the final 3D reconstruction with missing wedge correction, and the fifth column presents the flexibly fitted model at the corresponding tilt angles. **b**, Zoomed-in view of the final 3D density map displayed in orthogonal views, shown at two contour levels. **c**, Superimposition of the high contour level map from (b) onto its flexibly fitted model. **d**, Resolution evaluation of the final 3D density map using two criteria: Fourier shell correlation (FSC) between two-half maps reconstructed from the even and odd index of the tilted series and FSC between the final 3D map and the fitted structure model. The resolution for the former and latter criteria is evaluated at frequencies of 0.5 and 0.143, respectively.

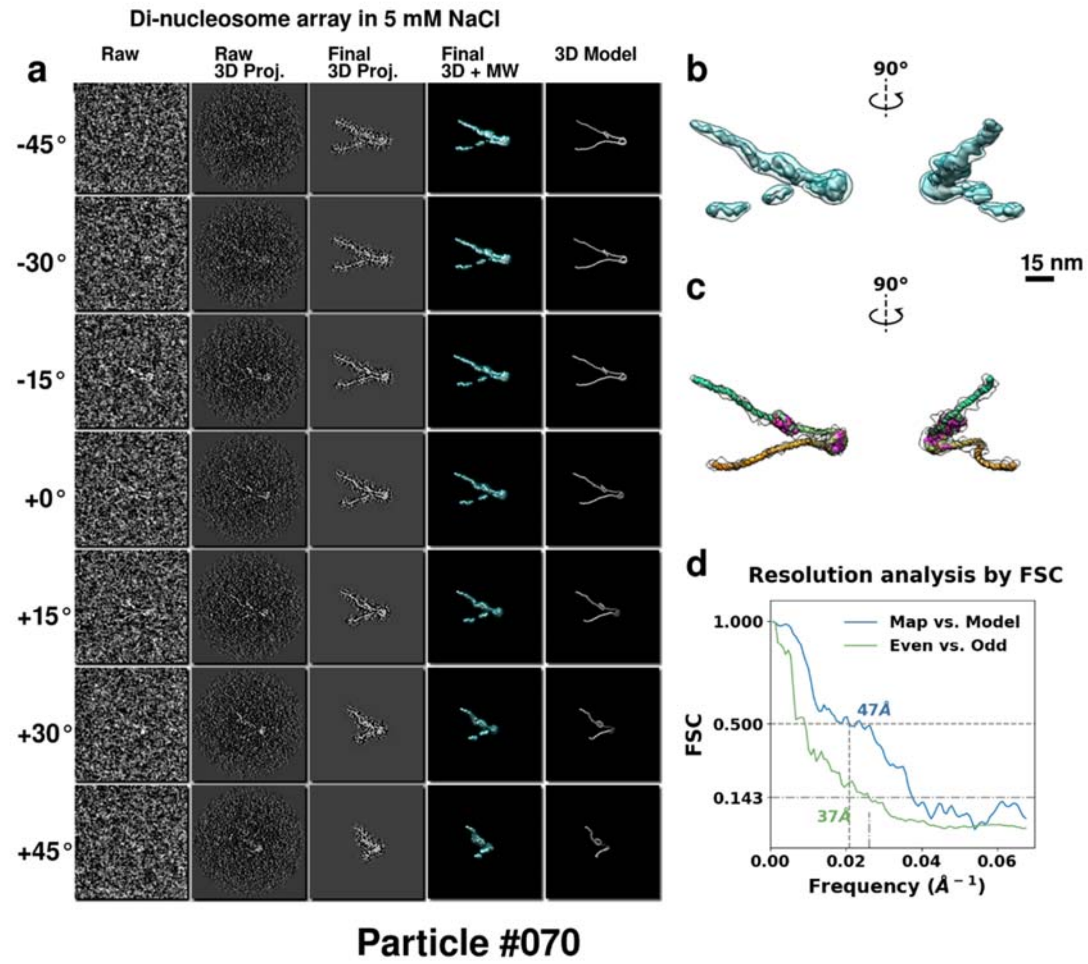

Supplementary Fig. 83. Cryo-ET 3D reconstruction of individual di-nucleosome particle (index no. 70) in 5 mM NaCl. **a**, IPET 3D reconstruction of individual di-nucleosome particles. The first column shows seven representative tilt images of an individual particle after CTF correction. Through alignment of the tilt images to a common center for 3D reconstruction via iterative refinement, the second and third columns display the 3D projections of the reconstruction before and after particle-shaped masking, respectively. The fourth column shows the final 3D reconstruction with missing wedge correction, and the fifth column presents the flexibly fitted model at the corresponding tilt angles. **b**, Zoomed-in view of the final 3D density map displayed in orthogonal views, shown at two contour levels. **c**, Superimposition of the high contour level map from (b) onto its flexibly fitted model. **d**, Resolution evaluation of the final 3D density map using two criteria: Fourier shell correlation (FSC) between two-half maps reconstructed from the even and odd index of the tilted series and FSC between the final 3D map and the fitted structure model. The resolution for the former and latter criteria is evaluated at frequencies of 0.5 and 0.143, respectively.

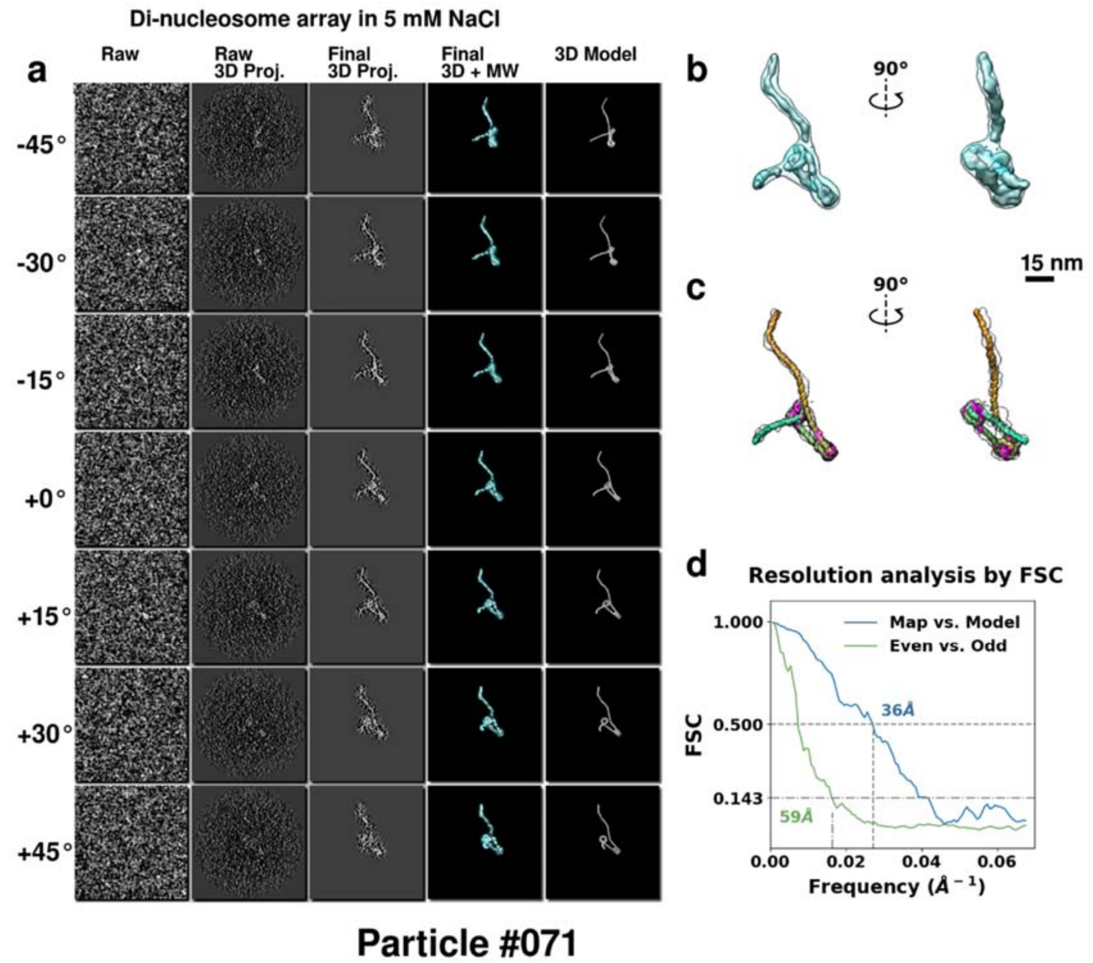

Supplementary Fig. 84. Cryo-ET 3D reconstruction of individual di-nucleosome particle (index no. 71) in 5 mM NaCl. a, IPET 3D reconstruction of individual di-nucleosome particles. The first column shows seven representative tilt images of an individual particle after CTF correction. Through alignment of the tilt images to a common center for 3D reconstruction via iterative refinement, the second and third columns display the 3D projections of the reconstruction before and after particle-shaped masking, respectively. The fourth column shows the final 3D reconstruction with missing wedge correction, and the fifth column presents the flexibly fitted model at the corresponding tilt angles. **b**, Zoomed-in view of the final 3D density map displayed in orthogonal views, shown at two contour levels. **c**, Superimposition of the high contour level map from (b) onto its flexibly fitted model. **d**, Resolution evaluation of the final 3D density map using two criteria: Fourier shell correlation (FSC) between two-half maps reconstructed from the even and odd index of the tilted series and FSC between the final 3D map and the fitted structure model. The resolution for the former and latter criteria is evaluated at frequencies of 0.5 and 0.143, respectively.

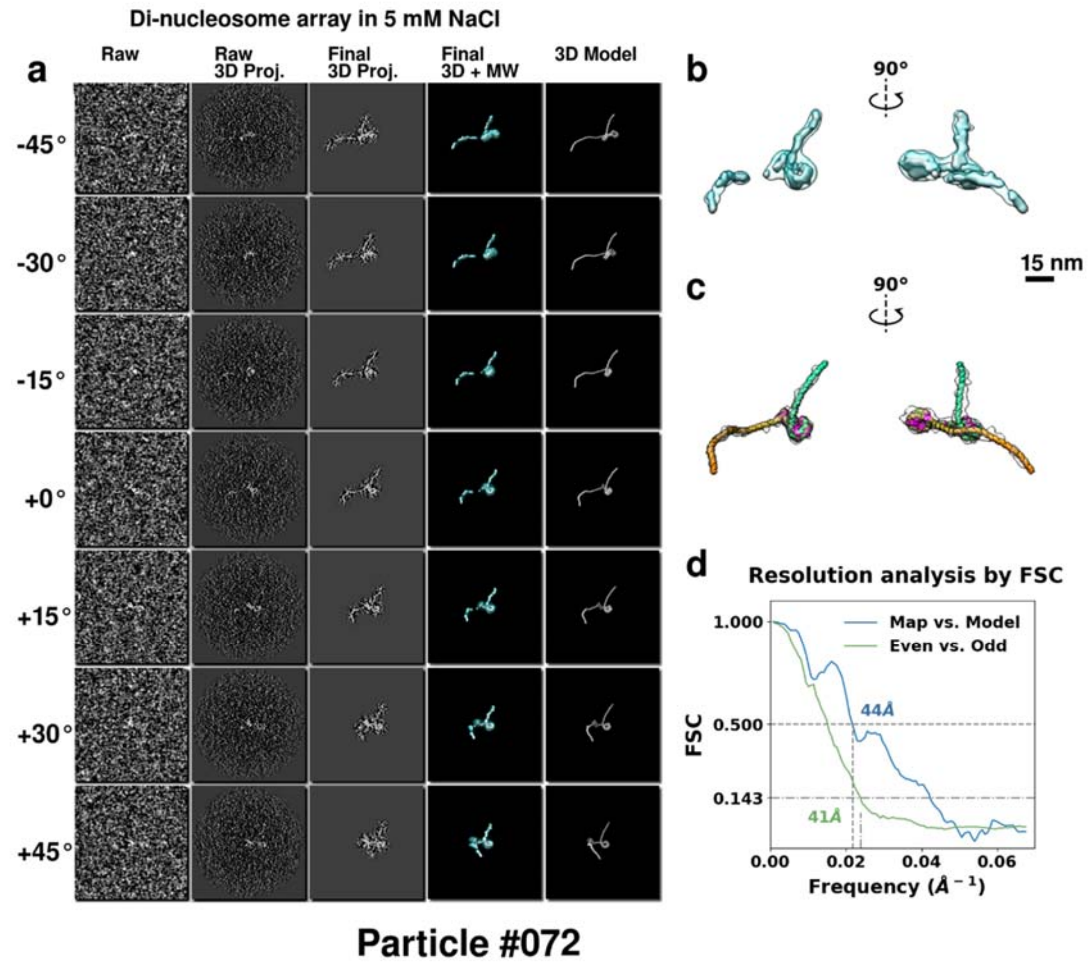

Supplementary Fig. 85. Cryo-ET 3D reconstruction of individual di-nucleosome particle (index no. 72) in 5 mM NaCl. a, IPET 3D reconstruction of individual di-nucleosome particles. The first column shows seven representative tilt images of an individual particle after CTF correction. Through alignment of the tilt images to a common center for 3D reconstruction via iterative refinement, the second and third columns display the 3D projections of the reconstruction before and after particle-shaped masking, respectively. The fourth column shows the final 3D reconstruction with missing wedge correction, and the fifth column presents the flexibly fitted model at the corresponding tilt angles. **b**, Zoomed-in view of the final 3D density map displayed in orthogonal views, shown at two contour levels. **c**, Superimposition of the high contour level map from (b) onto its flexibly fitted model. **d**, Resolution evaluation of the final 3D density map using two criteria: Fourier shell correlation (FSC) between two-half maps reconstructed from the even and odd index of the tilted series and FSC between the final 3D map and the fitted structure model. The resolution for the former and latter criteria is evaluated at frequencies of 0.5 and 0.143, respectively.

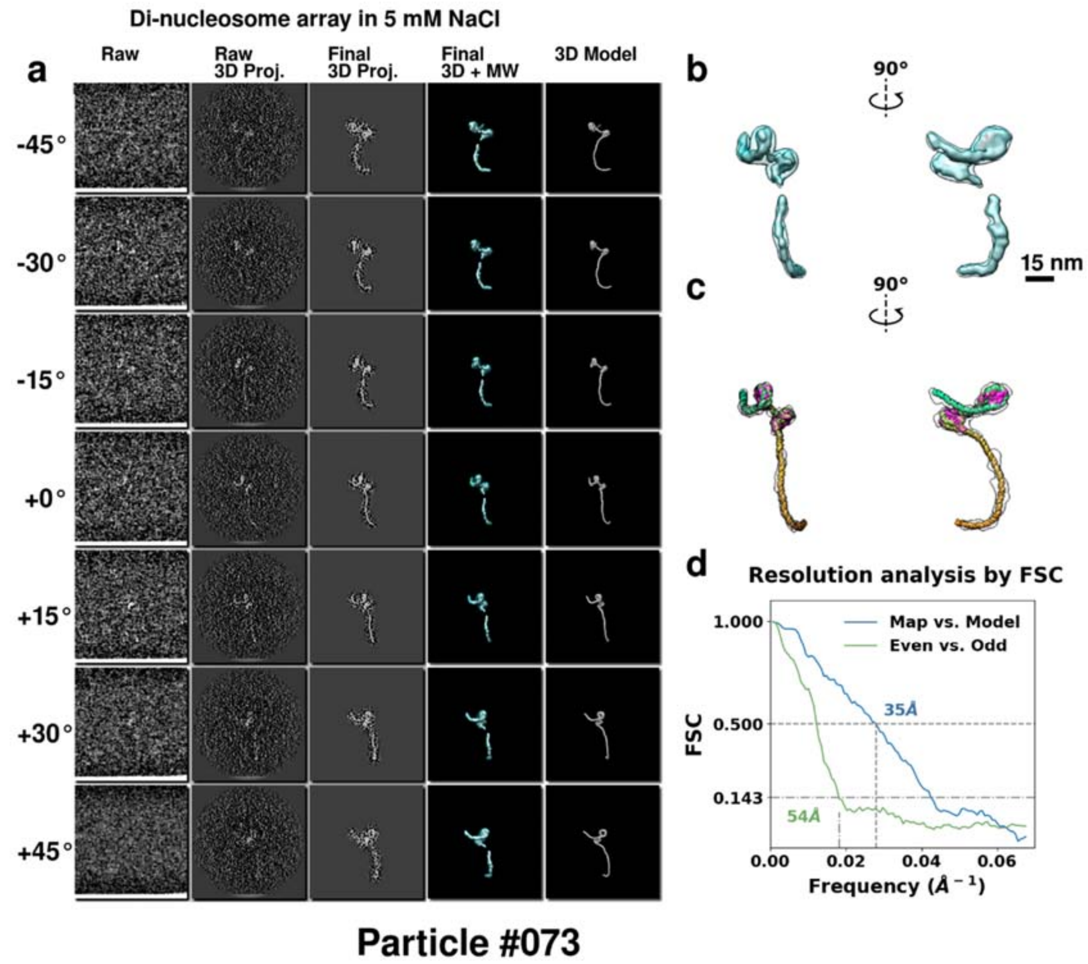

Supplementary Fig. 86. Cryo-ET 3D reconstruction of individual di-nucleosome particle (index no. 73) in 5 mM NaCl. a, IPET 3D reconstruction of individual di-nucleosome particles. The first column shows seven representative tilt images of an individual particle after CTF correction. Through alignment of the tilt images to a common center for 3D reconstruction via iterative refinement, the second and third columns display the 3D projections of the reconstruction before and after particle-shaped masking, respectively. The fourth column shows the final 3D reconstruction with missing wedge correction, and the fifth column presents the flexibly fitted model at the corresponding tilt angles. **b**, Zoomed-in view of the final 3D density map displayed in orthogonal views, shown at two contour levels. **c**, Superimposition of the high contour level map from (b) onto its flexibly fitted model. **d**, Resolution evaluation of the final 3D density map using two criteria: Fourier shell correlation (FSC) between two-half maps reconstructed from the even and odd index of the tilted series and FSC between the final 3D map and the fitted structure model. The resolution for the former and latter criteria is evaluated at frequencies of 0.5 and 0.143, respectively.

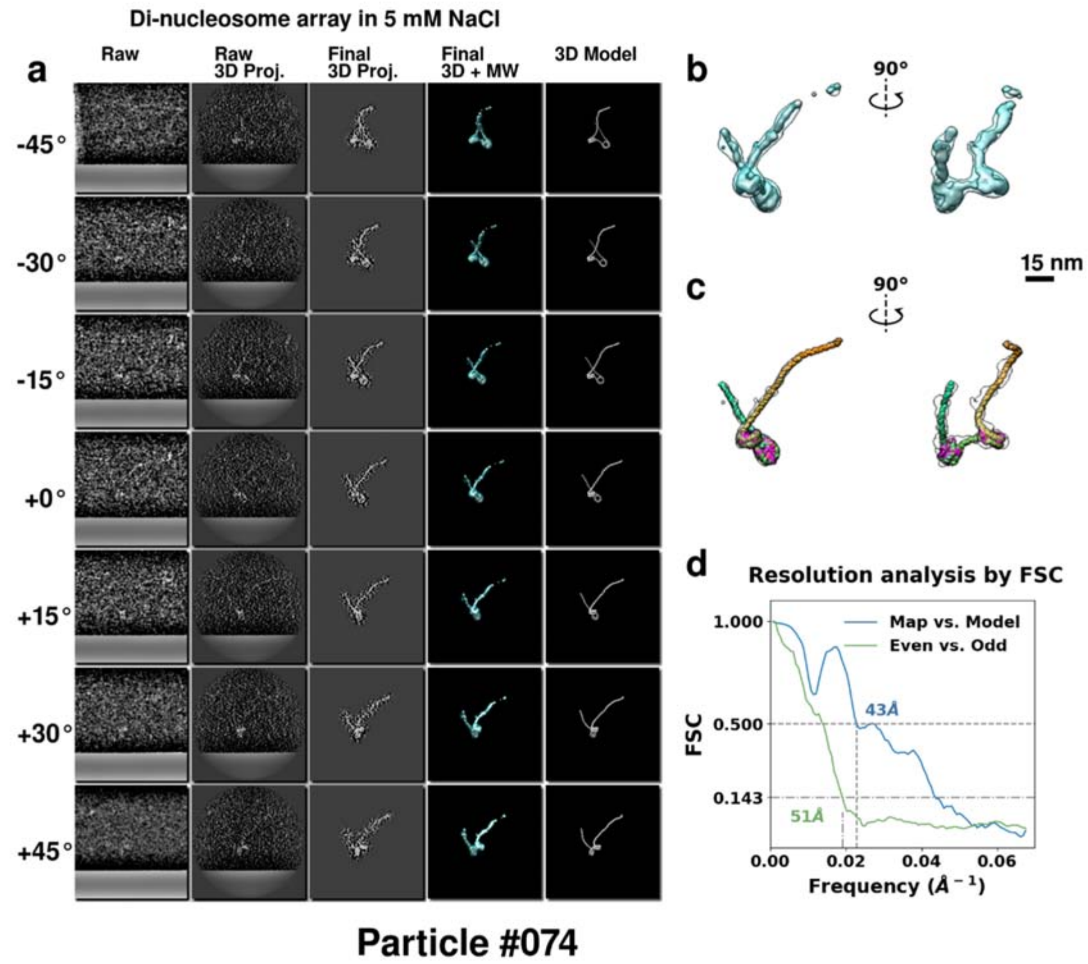

Supplementary Fig. 87. Cryo-ET 3D reconstruction of individual di-nucleosome particle (index no. 74) in 5 mM NaCl. a, IPET 3D reconstruction of individual di-nucleosome particles. The first column shows seven representative tilt images of an individual particle after CTF correction. Through alignment of the tilt images to a common center for 3D reconstruction via iterative refinement, the second and third columns display the 3D projections of the reconstruction before and after particle-shaped masking, respectively. The fourth column shows the final 3D reconstruction with missing wedge correction, and the fifth column presents the flexibly fitted model at the corresponding tilt angles. **b**, Zoomed-in view of the final 3D density map displayed in orthogonal views, shown at two contour levels. **c**, Superimposition of the high contour level map from (b) onto its flexibly fitted model. **d**, Resolution evaluation of the final 3D density map using two criteria: Fourier shell correlation (FSC) between two-half maps reconstructed from the even and odd index of the tilted series and FSC between the final 3D map and the fitted structure model. The resolution for the former and latter criteria is evaluated at frequencies of 0.5 and 0.143, respectively.

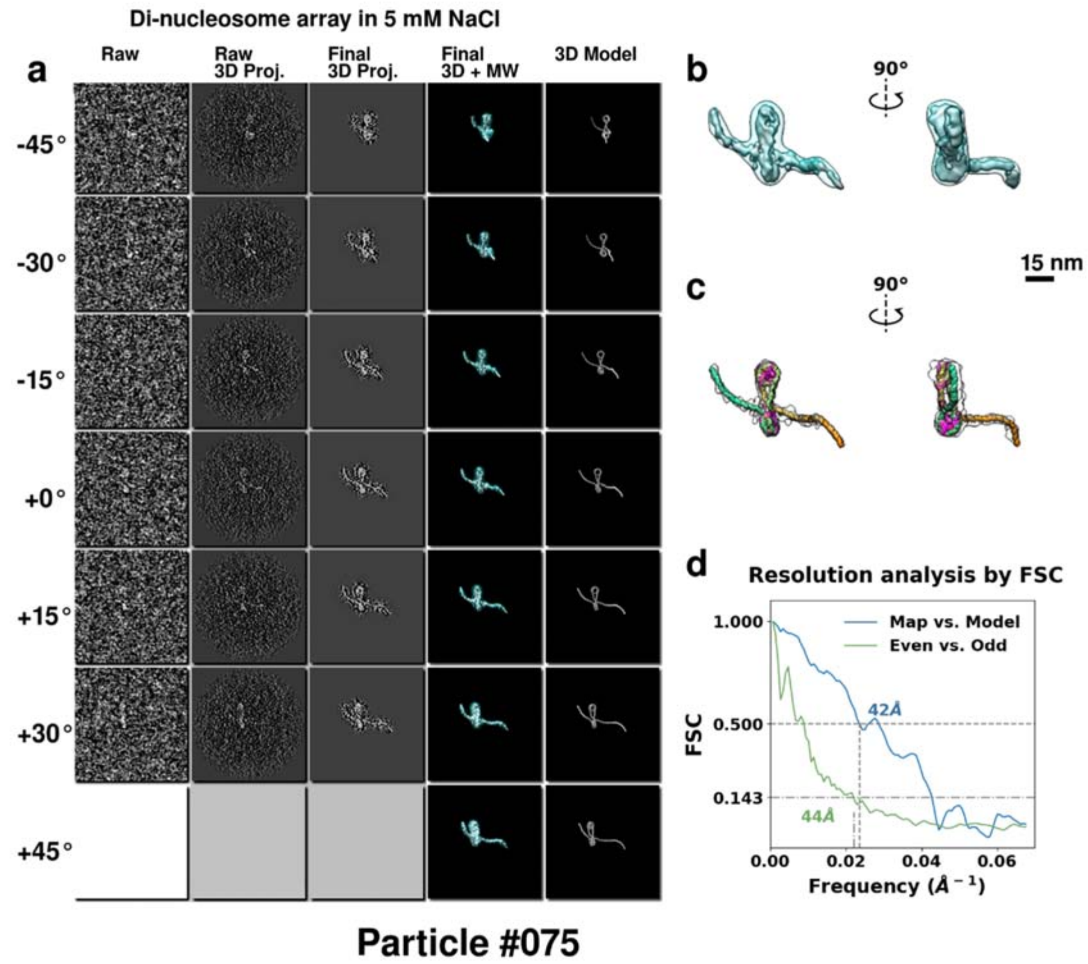

Supplementary Fig. 88. Cryo-ET 3D reconstruction of individual di-nucleosome particle (index no. 75) in 5 mM NaCl. a, IPET 3D reconstruction of individual di-nucleosome particles. The first column shows seven representative tilt images of an individual particle after CTF correction. Through alignment of the tilt images to a common center for 3D reconstruction via iterative refinement, the second and third columns display the 3D projections of the reconstruction before and after particle-shaped masking, respectively. The fourth column shows the final 3D reconstruction with missing wedge correction, and the fifth column presents the flexibly fitted model at the corresponding tilt angles. **b**, Zoomed-in view of the final 3D density map displayed in orthogonal views, shown at two contour levels. **c**, Superimposition of the high contour level map from (b) onto its flexibly fitted model. **d**, Resolution evaluation of the final 3D density map using two criteria: Fourier shell correlation (FSC) between two-half maps reconstructed from the even and odd index of the tilted series and FSC between the final 3D map and the fitted structure model. The resolution for the former and latter criteria is evaluated at frequencies of 0.5 and 0.143, respectively.

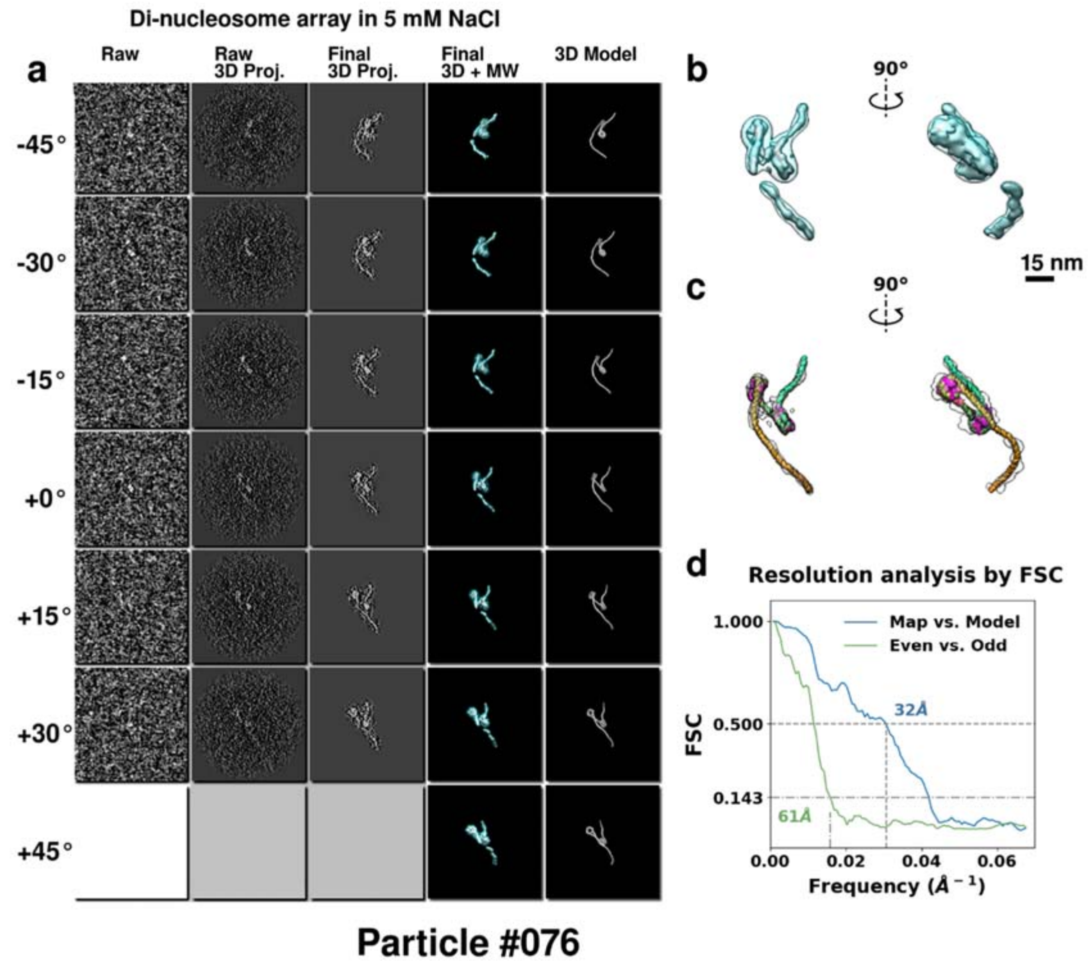

Supplementary Fig. 89. Cryo-ET 3D reconstruction of individual di-nucleosome particle (index no. 76) in 5 mM NaCl. a, IPET 3D reconstruction of individual di-nucleosome particles. The first column shows seven representative tilt images of an individual particle after CTF correction. Through alignment of the tilt images to a common center for 3D reconstruction via iterative refinement, the second and third columns display the 3D projections of the reconstruction before and after particle-shaped masking, respectively. The fourth column shows the final 3D reconstruction with missing wedge correction, and the fifth column presents the flexibly fitted model at the corresponding tilt angles. **b**, Zoomed-in view of the final 3D density map displayed in orthogonal views, shown at two contour levels. **c**, Superimposition of the high contour level map from (b) onto its flexibly fitted model. **d**, Resolution evaluation of the final 3D density map using two criteria: Fourier shell correlation (FSC) between two-half maps reconstructed from the even and odd index of the tilted series and FSC between the final 3D map and the fitted structure model. The resolution for the former and latter criteria is evaluated at frequencies of 0.5 and 0.143, respectively.

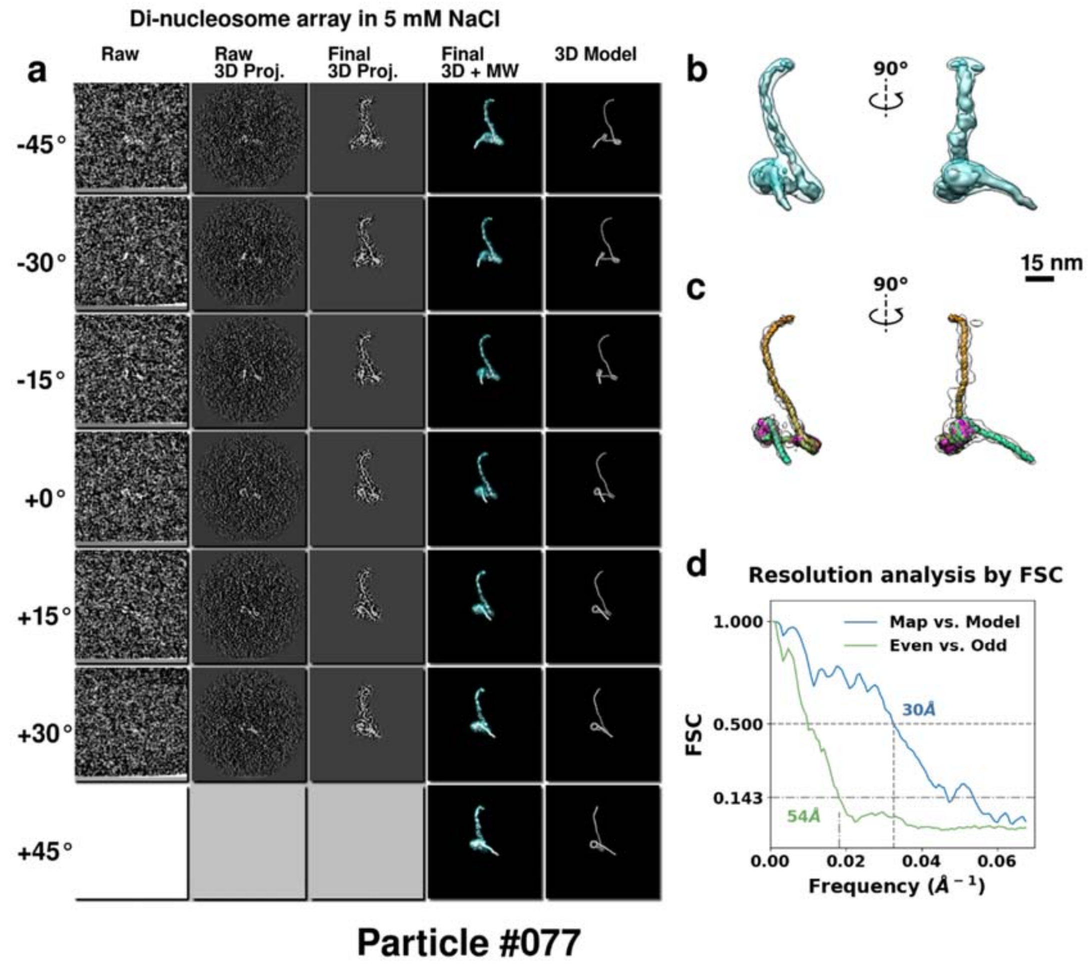

Supplementary Fig. 90. Cryo-ET 3D reconstruction of individual di-nucleosome particle (index no. 77) in 5 mM NaCl. a, IPET 3D reconstruction of individual di-nucleosome particles. The first column shows seven representative tilt images of an individual particle after CTF correction. Through alignment of the tilt images to a common center for 3D reconstruction via iterative refinement, the second and third columns display the 3D projections of the reconstruction before and after particle-shaped masking, respectively. The fourth column shows the final 3D reconstruction with missing wedge correction, and the fifth column presents the flexibly fitted model at the corresponding tilt angles. **b**, Zoomed-in view of the final 3D density map displayed in orthogonal views, shown at two contour levels. **c**, Superimposition of the high contour level map from (b) onto its flexibly fitted model. **d**, Resolution evaluation of the final 3D density map using two criteria: Fourier shell correlation (FSC) between two-half maps reconstructed from the even and odd index of the tilted series and FSC between the final 3D map and the fitted structure model. The resolution for the former and latter criteria is evaluated at frequencies of 0.5 and 0.143, respectively.

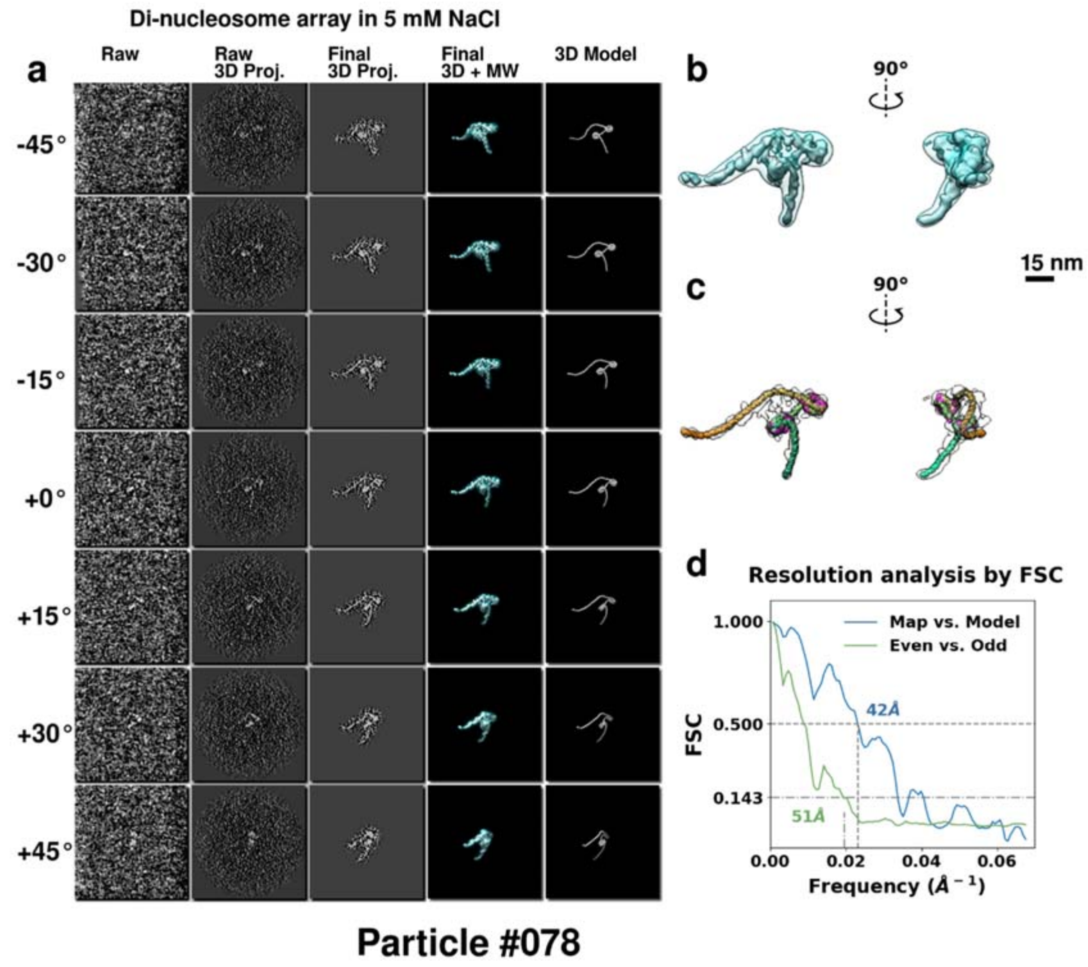

Supplementary Fig. 91. Cryo-ET 3D reconstruction of individual di-nucleosome particle (index no. 78) in 5 mM NaCl. a, IPET 3D reconstruction of individual di-nucleosome particles. The first column shows seven representative tilt images of an individual particle after CTF correction. Through alignment of the tilt images to a common center for 3D reconstruction via iterative refinement, the second and third columns display the 3D projections of the reconstruction before and after particle-shaped masking, respectively. The fourth column shows the final 3D reconstruction with missing wedge correction, and the fifth column presents the flexibly fitted model at the corresponding tilt angles. **b**, Zoomed-in view of the final 3D density map displayed in orthogonal views, shown at two contour levels. **c**, Superimposition of the high contour level map from (b) onto its flexibly fitted model. **d**, Resolution evaluation of the final 3D density map using two criteria: Fourier shell correlation (FSC) between two-half maps reconstructed from the even and odd index of the tilted series and FSC between the final 3D map and the fitted structure model. The resolution for the former and latter criteria is evaluated at frequencies of 0.5 and 0.143, respectively.

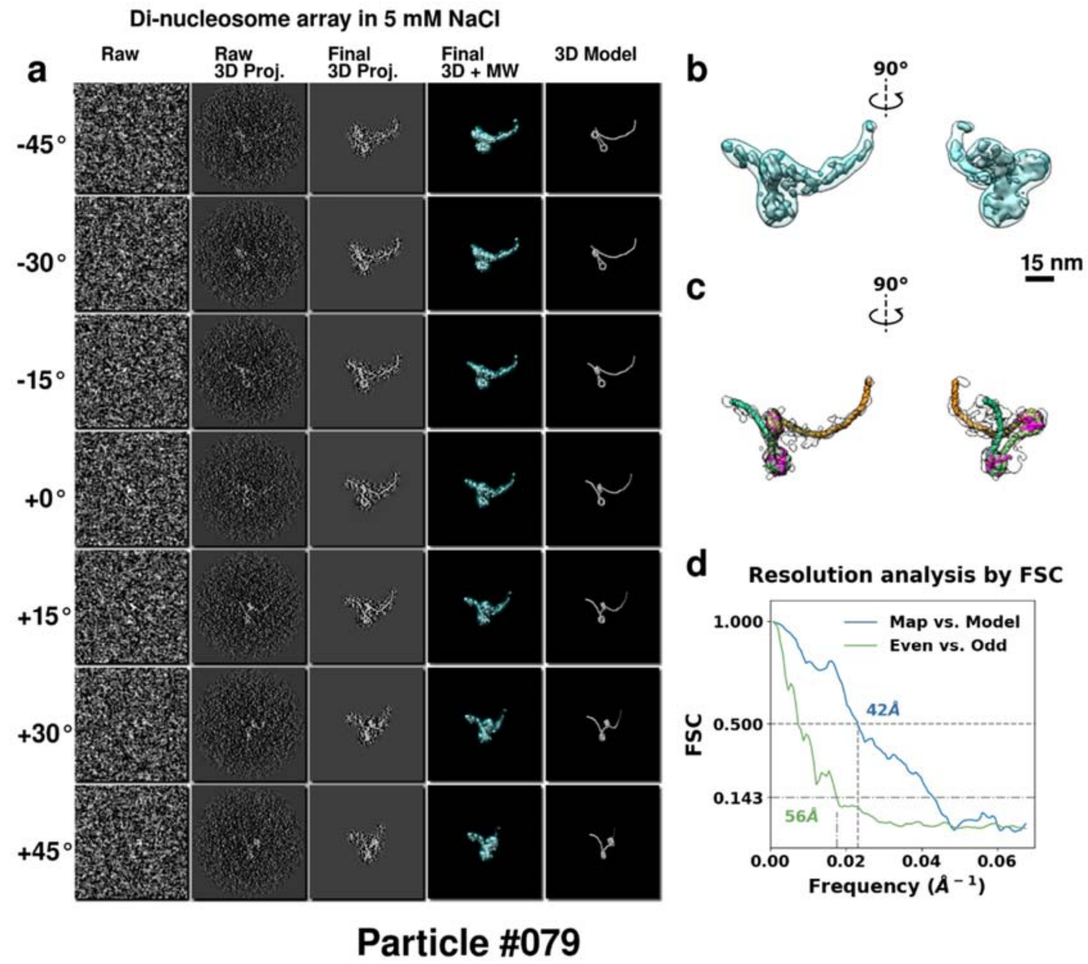

Supplementary Fig. 92. Cryo-ET 3D reconstruction of individual di-nucleosome particle (index no. 79) in 5 mM NaCl. a, IPET 3D reconstruction of individual di-nucleosome particles. The first column shows seven representative tilt images of an individual particle after CTF correction. Through alignment of the tilt images to a common center for 3D reconstruction via iterative refinement, the second and third columns display the 3D projections of the reconstruction before and after particle-shaped masking, respectively. The fourth column shows the final 3D reconstruction with missing wedge correction, and the fifth column presents the flexibly fitted model at the corresponding tilt angles. **b**, Zoomed-in view of the final 3D density map displayed in orthogonal views, shown at two contour levels. **c**, Superimposition of the high contour level map from (b) onto its flexibly fitted model. **d**, Resolution evaluation of the final 3D density map using two criteria: Fourier shell correlation (FSC) between two-half maps reconstructed from the even and odd index of the tilted series and FSC between the final 3D map and the fitted structure model. The resolution for the former and latter criteria is evaluated at frequencies of 0.5 and 0.143, respectively.

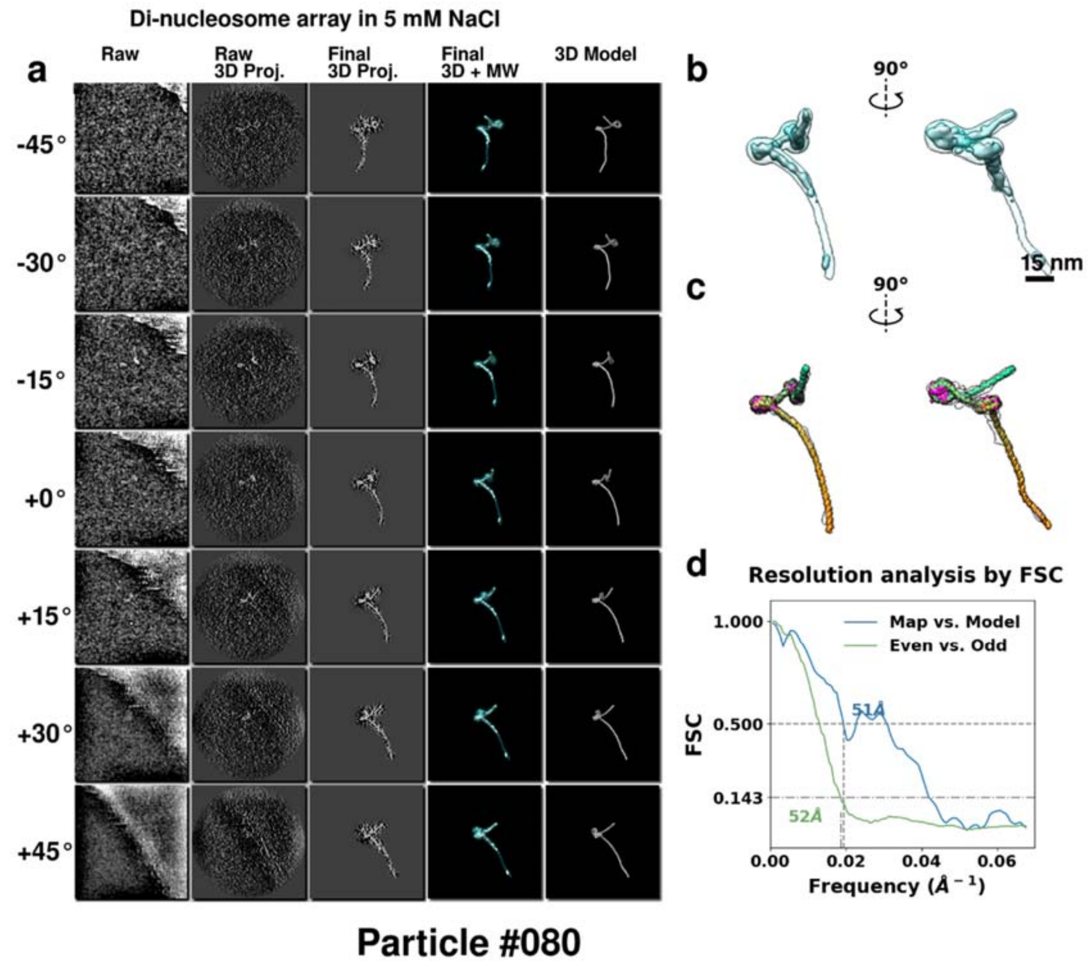

Supplementary Fig. 93. Cryo-ET 3D reconstruction of individual di-nucleosome particle (index no. 80) in 5 mM NaCl. a, IPET 3D reconstruction of individual di-nucleosome particles. The first column shows seven representative tilt images of an individual particle after CTF correction. Through alignment of the tilt images to a common center for 3D reconstruction via iterative refinement, the second and third columns display the 3D projections of the reconstruction before and after particle-shaped masking, respectively. The fourth column shows the final 3D reconstruction with missing wedge correction, and the fifth column presents the flexibly fitted model at the corresponding tilt angles. **b**, Zoomed-in view of the final 3D density map displayed in orthogonal views, shown at two contour levels. **c**, Superimposition of the high contour level map from (b) onto its flexibly fitted model. **d**, Resolution evaluation of the final 3D density map using two criteria: Fourier shell correlation (FSC) between two-half maps reconstructed from the even and odd index of the tilted series and FSC between the final 3D map and the fitted structure model. The resolution for the former and latter criteria is evaluated at frequencies of 0.5 and 0.143, respectively.

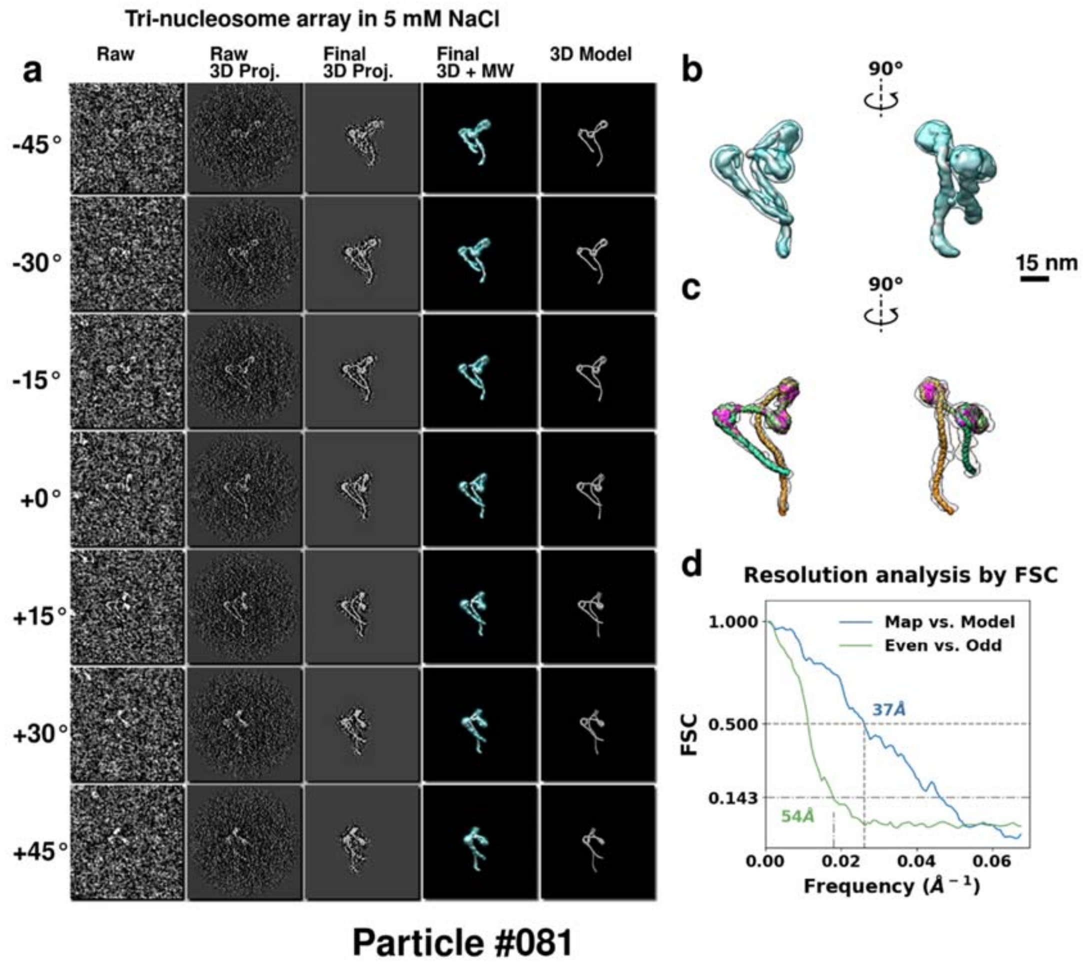

Supplementary Fig. 94. Cryo-ET 3D reconstruction of individual tri-nucleosome particle (index no. 81) in 5 mM NaCl. a, IPET 3D reconstruction of individual tri-nucleosome particles. The first column shows seven representative tilt images of an individual particle after CTF correction. Through alignment of the tilt images to a common center for 3D reconstruction via iterative refinement, the second and third columns display the 3D projections of the reconstruction before and after particle-shaped masking, respectively. The fourth column shows the final 3D reconstruction with missing wedge correction, and the fifth column presents the flexibly fitted model at the corresponding tilt angles. **b**, Zoomed-in view of the final 3D density map displayed in orthogonal views, shown at two contour levels. **c**, Superimposition of the high contour level map from (b) onto its flexibly fitted model. **d**, Resolution evaluation of the final 3D density map using two criteria: Fourier shell correlation (FSC) between two-half maps reconstructed from the even and odd index of the tilted series and FSC between the final 3D map and the fitted structure model. The resolution for the former and latter criteria is evaluated at frequencies of 0.5 and 0.143, respectively.

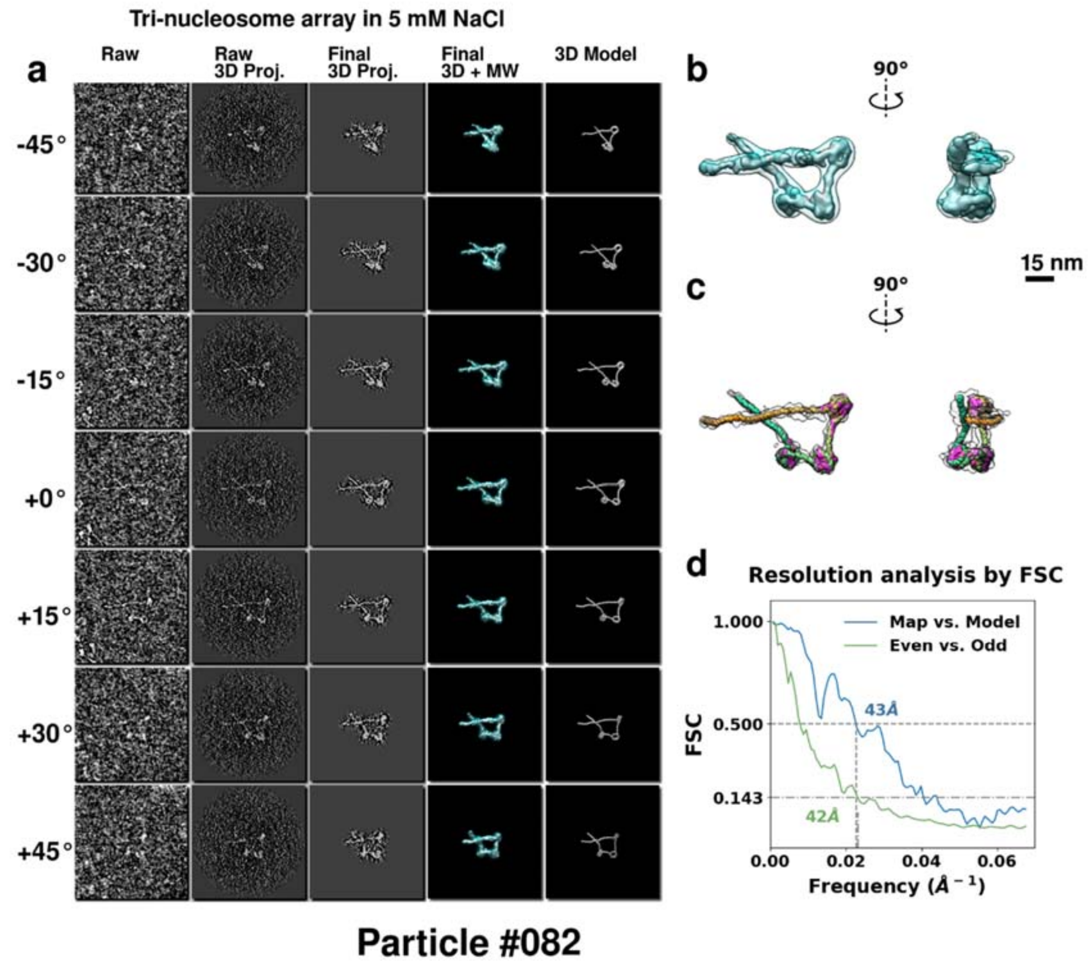

Supplementary Fig. 95. Cryo-ET 3D reconstruction of individual tri-nucleosome particle (index no. 82) in 5 mM NaCl. a, IPET 3D reconstruction of individual tri-nucleosome particles. The first column shows seven representative tilt images of an individual particle after CTF correction. Through alignment of the tilt images to a common center for 3D reconstruction via iterative refinement, the second and third columns display the 3D projections of the reconstruction before and after particle-shaped masking, respectively. The fourth column shows the final 3D reconstruction with missing wedge correction, and the fifth column presents the flexibly fitted model at the corresponding tilt angles. **b**, Zoomed-in view of the final 3D density map displayed in orthogonal views, shown at two contour levels. **c**, Superimposition of the high contour level map from (b) onto its flexibly fitted model. **d**, Resolution evaluation of the final 3D density map using two criteria: Fourier shell correlation (FSC) between two-half maps reconstructed from the even and odd index of the tilted series and FSC between the final 3D map and the fitted structure model. The resolution for the former and latter criteria is evaluated at frequencies of 0.5 and 0.143, respectively.

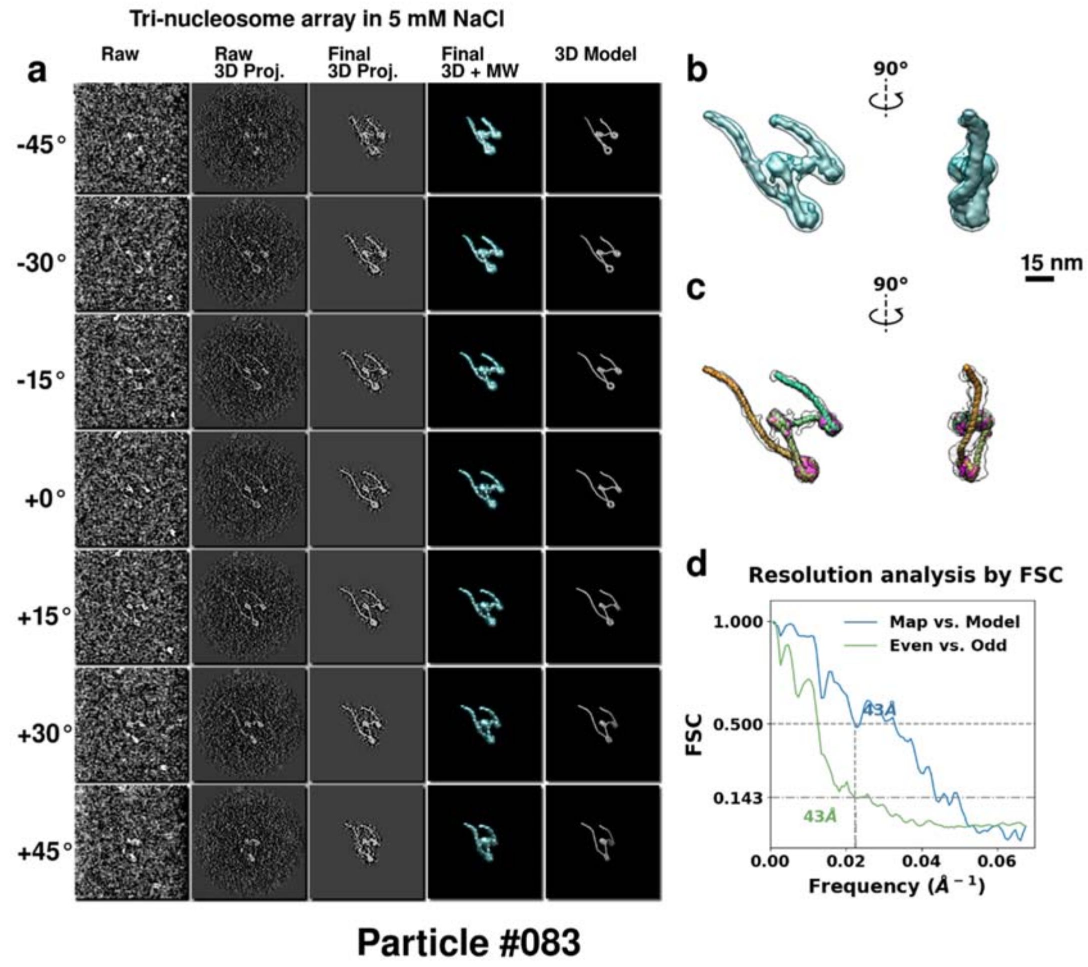

Supplementary Fig. 96. Cryo-ET 3D reconstruction of individual tri-nucleosome particle (index no. 83) in 5 mM NaCl. a, IPET 3D reconstruction of individual tri-nucleosome particles. The first column shows seven representative tilt images of an individual particle after CTF correction. Through alignment of the tilt images to a common center for 3D reconstruction via iterative refinement, the second and third columns display the 3D projections of the reconstruction before and after particle-shaped masking, respectively. The fourth column shows the final 3D reconstruction with missing wedge correction, and the fifth column presents the flexibly fitted model at the corresponding tilt angles. **b**, Zoomed-in view of the final 3D density map displayed in orthogonal views, shown at two contour levels. **c**, Superimposition of the high contour level map from (b) onto its flexibly fitted model. **d**, Resolution evaluation of the final 3D density map using two criteria: Fourier shell correlation (FSC) between two-half maps reconstructed from the even and odd index of the tilted series and FSC between the final 3D map and the fitted structure model. The resolution for the former and latter criteria is evaluated at frequencies of 0.5 and 0.143, respectively.

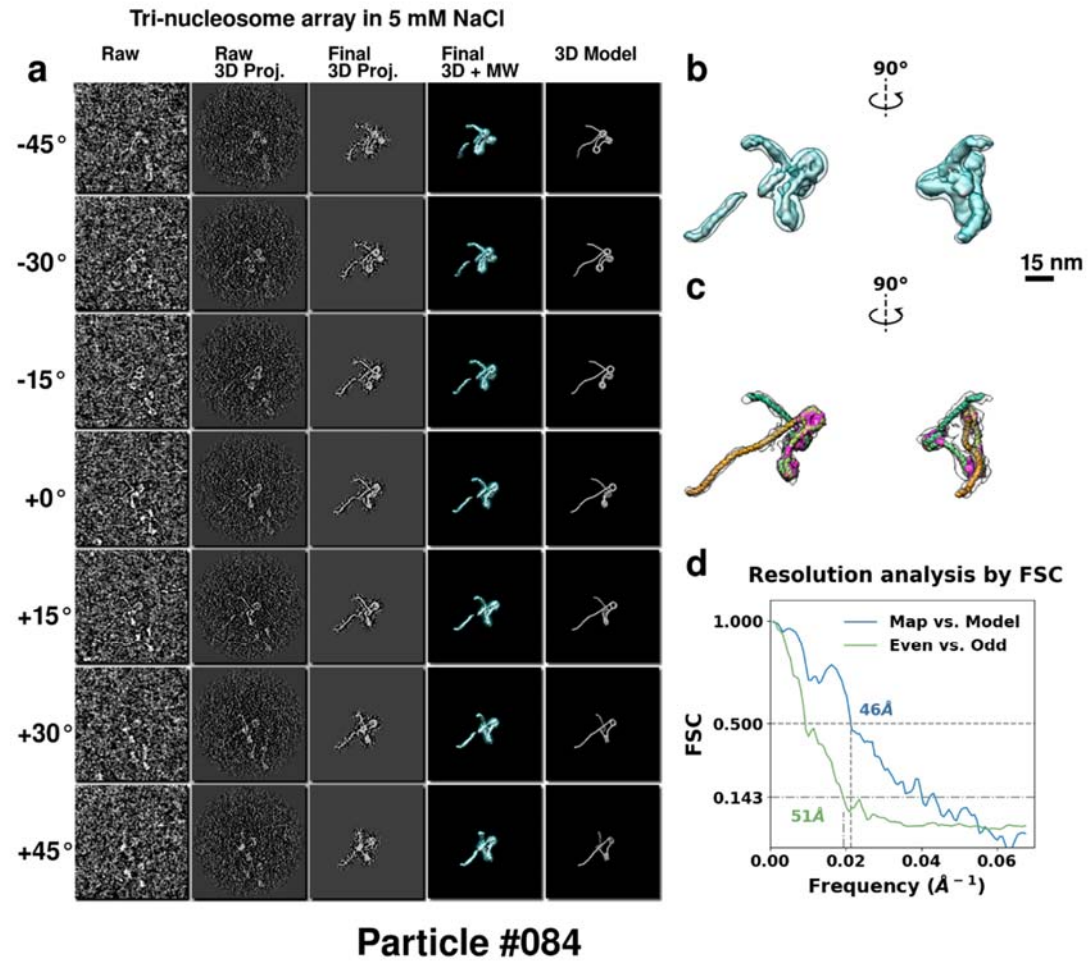

Supplementary Fig. 97. Cryo-ET 3D reconstruction of individual tri-nucleosome particle (index no. 84) in 5 mM NaCl. **a**, IPET 3D reconstruction of individual tri-nucleosome particles. The first column shows seven representative tilt images of an individual particle after CTF correction. Through alignment of the tilt images to a common center for 3D reconstruction via iterative refinement, the second and third columns display the 3D projections of the reconstruction before and after particle-shaped masking, respectively. The fourth column shows the final 3D reconstruction with missing wedge correction, and the fifth column presents the flexibly fitted model at the corresponding tilt angles. **b**, Zoomed-in view of the final 3D density map displayed in orthogonal views, shown at two contour levels. **c**, Superimposition of the high contour level map from (b) onto its flexibly fitted model. **d**, Resolution evaluation of the final 3D density map using two criteria: Fourier shell correlation (FSC) between two-half maps reconstructed from the even and odd index of the tilted series and FSC between the final 3D map and the fitted structure model. The resolution for the former and latter criteria is evaluated at frequencies of 0.5 and 0.143, respectively.

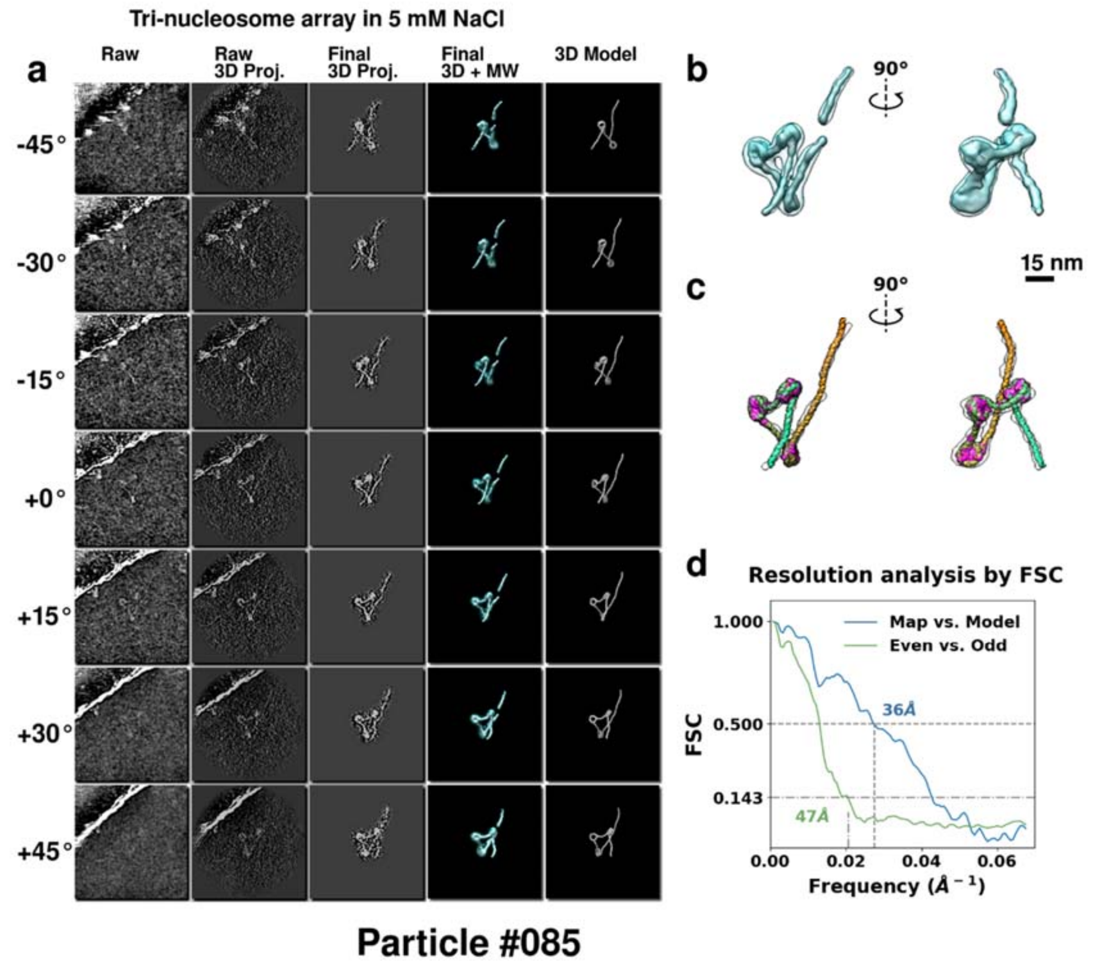

Supplementary Fig. 98. Cryo-ET 3D reconstruction of individual tri-nucleosome particle (index no. 85) in 5 mM NaCl. **a**, IPET 3D reconstruction of individual tri-nucleosome particles. The first column shows seven representative tilt images of an individual particle after CTF correction. Through alignment of the tilt images to a common center for 3D reconstruction via iterative refinement, the second and third columns display the 3D projections of the reconstruction before and after particle-shaped masking, respectively. The fourth column shows the final 3D reconstruction with missing wedge correction, and the fifth column presents the flexibly fitted model at the corresponding tilt angles. **b**, Zoomed-in view of the final 3D density map displayed in orthogonal views, shown at two contour levels. **c**, Superimposition of the high contour level map from (b) onto its flexibly fitted model. **d**, Resolution evaluation of the final 3D density map using two criteria: Fourier shell correlation (FSC) between two-half maps reconstructed from the even and odd index of the tilted series and FSC between the final 3D map and the fitted structure model. The resolution for the former and latter criteria is evaluated at frequencies of 0.5 and 0.143, respectively.

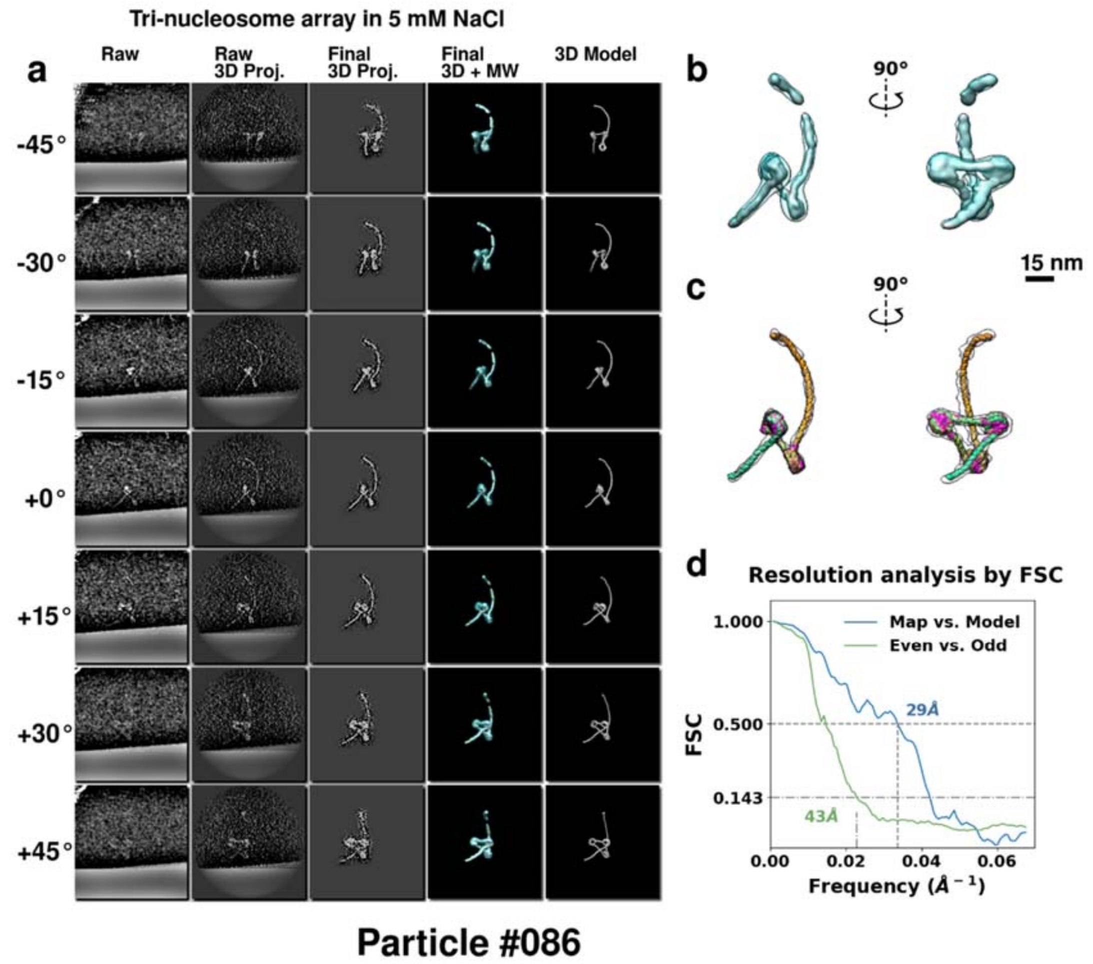

Supplementary Fig. 99. Cryo-ET 3D reconstruction of individual tri-nucleosome particle (index no. 86) in 5 mM NaCl. a, IPET 3D reconstruction of individual tri-nucleosome particles. The first column shows seven representative tilt images of an individual particle after CTF correction. Through alignment of the tilt images to a common center for 3D reconstruction via iterative refinement, the second and third columns display the 3D projections of the reconstruction before and after particle-shaped masking, respectively. The fourth column shows the final 3D reconstruction with missing wedge correction, and the fifth column presents the flexibly fitted model at the corresponding tilt angles. **b**, Zoomed-in view of the final 3D density map displayed in orthogonal views, shown at two contour levels. **c**, Superimposition of the high contour level map from (b) onto its flexibly fitted model. **d**, Resolution evaluation of the final 3D density map using two criteria: Fourier shell correlation (FSC) between two-half maps reconstructed from the even and odd index of the tilted series and FSC between the final 3D map and the fitted structure model. The resolution for the former and latter criteria is evaluated at frequencies of 0.5 and 0.143, respectively.

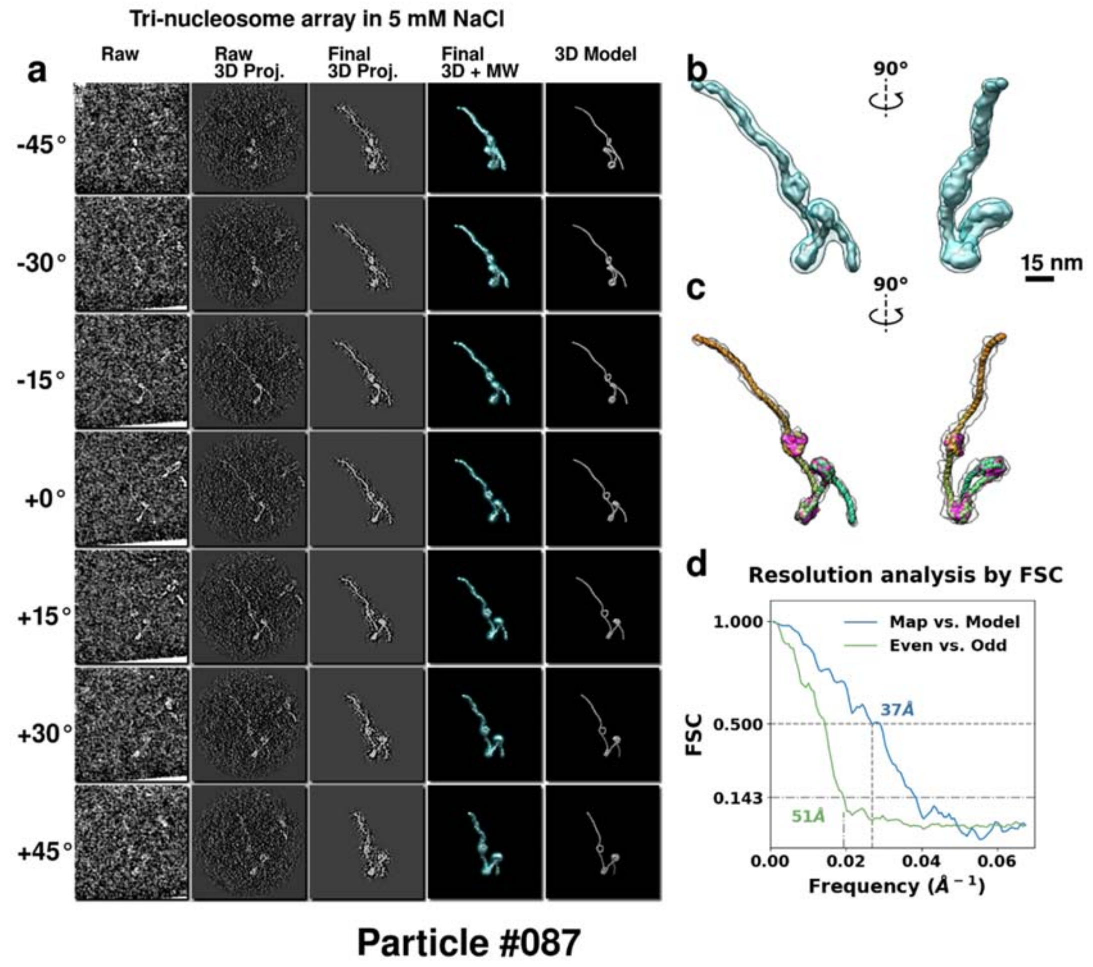

Supplementary Fig. 100. Cryo-ET 3D reconstruction of individual tri-nucleosome particle (index no. 87) in 5 mM NaCl. **a**, IPET 3D reconstruction of individual tri-nucleosome particles. The first column shows seven representative tilt images of an individual particle after CTF correction. Through alignment of the tilt images to a common center for 3D reconstruction via iterative refinement, the second and third columns display the 3D projections of the reconstruction before and after particle-shaped masking, respectively. The fourth column shows the final 3D reconstruction with missing wedge correction, and the fifth column presents the flexibly fitted model at the corresponding tilt angles. **b**, Zoomed-in view of the final 3D density map displayed in orthogonal views, shown at two contour levels. **c**, Superimposition of the high contour level map from (b) onto its flexibly fitted model. **d**, Resolution evaluation of the final 3D density map using two criteria: Fourier shell correlation (FSC) between two-half maps reconstructed from the even and odd index of the tilted series and FSC between the final 3D map and the fitted structure model. The resolution for the former and latter criteria is evaluated at frequencies of 0.5 and 0.143, respectively.

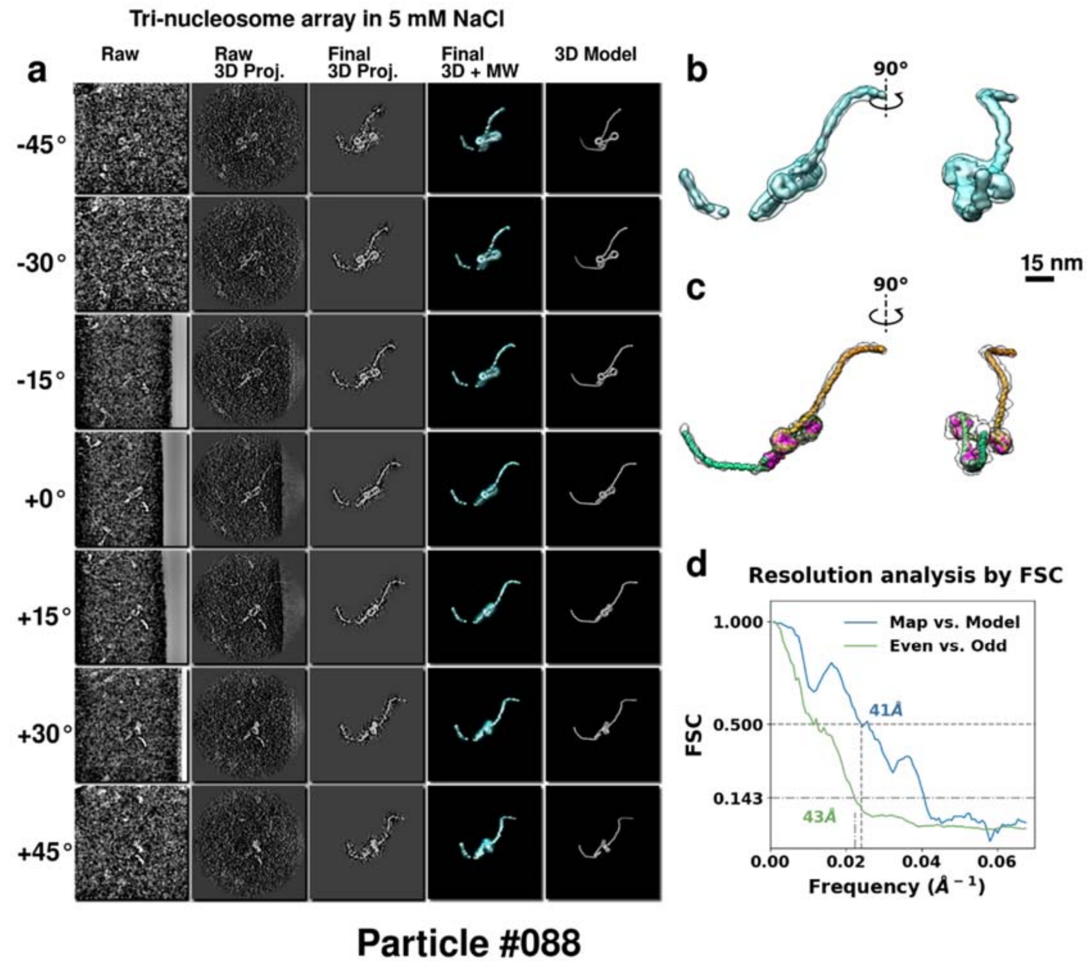

Supplementary Fig. 101. Cryo-ET 3D reconstruction of individual tri-nucleosome particle (index no. 88) in 5 mM NaCl. a, IPET 3D reconstruction of individual tri-nucleosome particles. The first column shows seven representative tilt images of an individual particle after CTF correction. Through alignment of the tilt images to a common center for 3D reconstruction via iterative refinement, the second and third columns display the 3D projections of the reconstruction before and after particle-shaped masking, respectively. The fourth column shows the final 3D reconstruction with missing wedge correction, and the fifth column presents the flexibly fitted model at the corresponding tilt angles. **b**, Zoomed-in view of the final 3D density map displayed in orthogonal views, shown at two contour levels. **c**, Superimposition of the high contour level map from (b) onto its flexibly fitted model. **d**, Resolution evaluation of the final 3D density map using two criteria: Fourier shell correlation (FSC) between two-half maps reconstructed from the even and odd index of the tilted series and FSC between the final 3D map and the fitted structure model. The resolution for the former and latter criteria is evaluated at frequencies of 0.5 and 0.143, respectively.

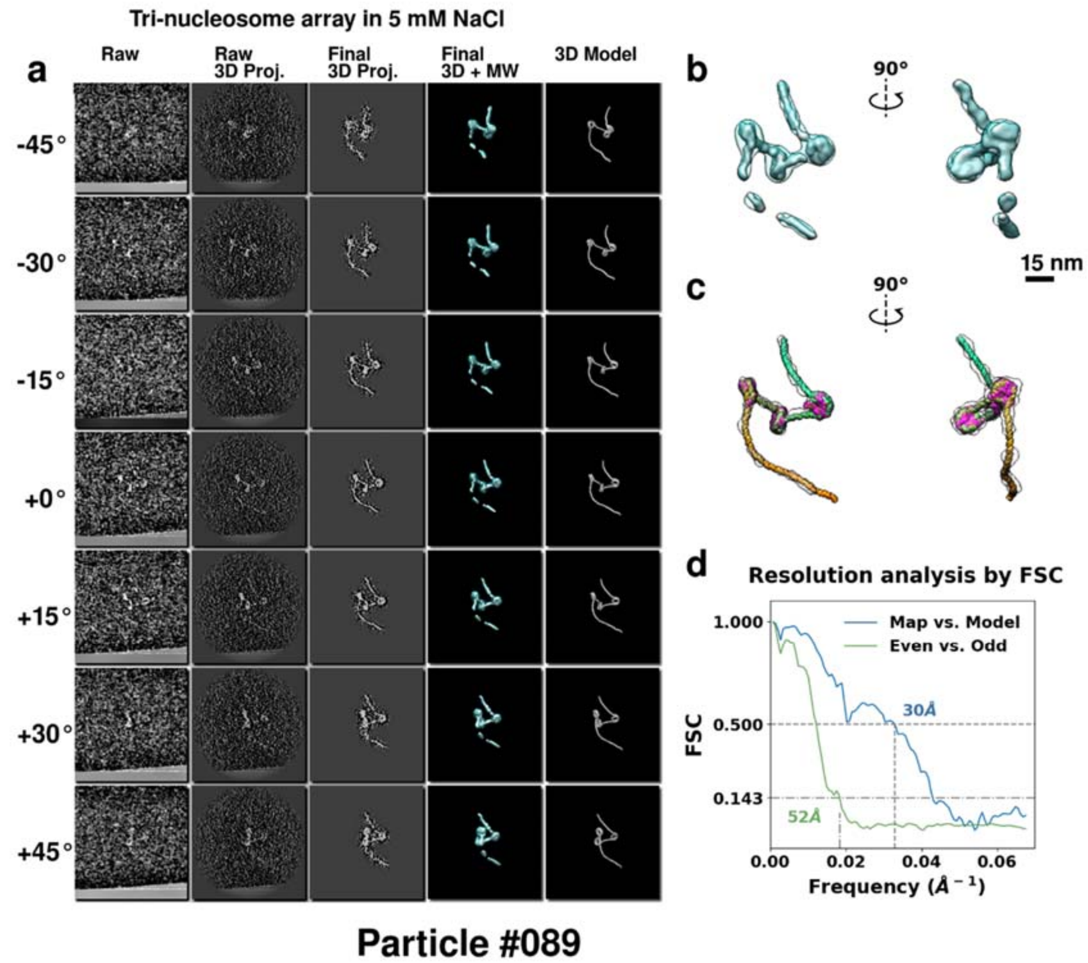

Supplementary Fig. 102. Cryo-ET 3D reconstruction of individual tri-nucleosome particle (index no. 89) in 5 mM NaCl. a, IPET 3D reconstruction of individual tri-nucleosome particles. The first column shows seven representative tilt images of an individual particle after CTF correction. Through alignment of the tilt images to a common center for 3D reconstruction via iterative refinement, the second and third columns display the 3D projections of the reconstruction before and after particle-shaped masking, respectively. The fourth column shows the final 3D reconstruction with missing wedge correction, and the fifth column presents the flexibly fitted model at the corresponding tilt angles. **b**, Zoomed-in view of the final 3D density map displayed in orthogonal views, shown at two contour levels. **c**, Superimposition of the high contour level map from (b) onto its flexibly fitted model. **d**, Resolution evaluation of the final 3D density map using two criteria: Fourier shell correlation (FSC) between two-half maps reconstructed from the even and odd index of the tilted series and FSC between the final 3D map and the fitted structure model. The resolution for the former and latter criteria is evaluated at frequencies of 0.5 and 0.143, respectively.

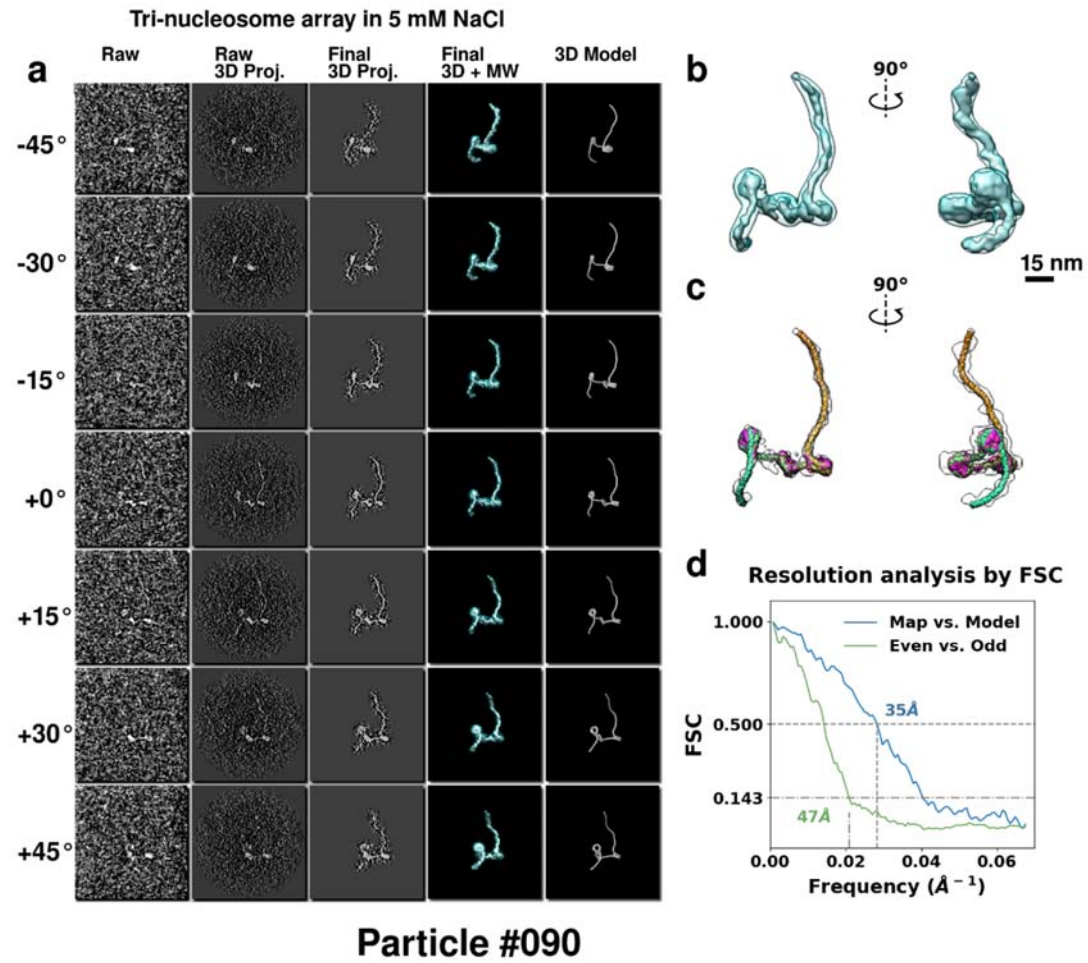

Supplementary Fig. 103. Cryo-ET 3D reconstruction of individual tri-nucleosome particle (index no. 90) in 5 mM NaCl. a, IPET 3D reconstruction of individual tri-nucleosome particles. The first column shows seven representative tilt images of an individual particle after CTF correction. Through alignment of the tilt images to a common center for 3D reconstruction via iterative refinement, the second and third columns display the 3D projections of the reconstruction before and after particle-shaped masking, respectively. The fourth column shows the final 3D reconstruction with missing wedge correction, and the fifth column presents the flexibly fitted model at the corresponding tilt angles. **b**, Zoomed-in view of the final 3D density map displayed in orthogonal views, shown at two contour levels. **c**, Superimposition of the high contour level map from (b) onto its flexibly fitted model. **d**, Resolution evaluation of the final 3D density map using two criteria: Fourier shell correlation (FSC) between two-half maps reconstructed from the even and odd index of the tilted series and FSC between the final 3D map and the fitted structure model. The resolution for the former and latter criteria is evaluated at frequencies of 0.5 and 0.143, respectively.

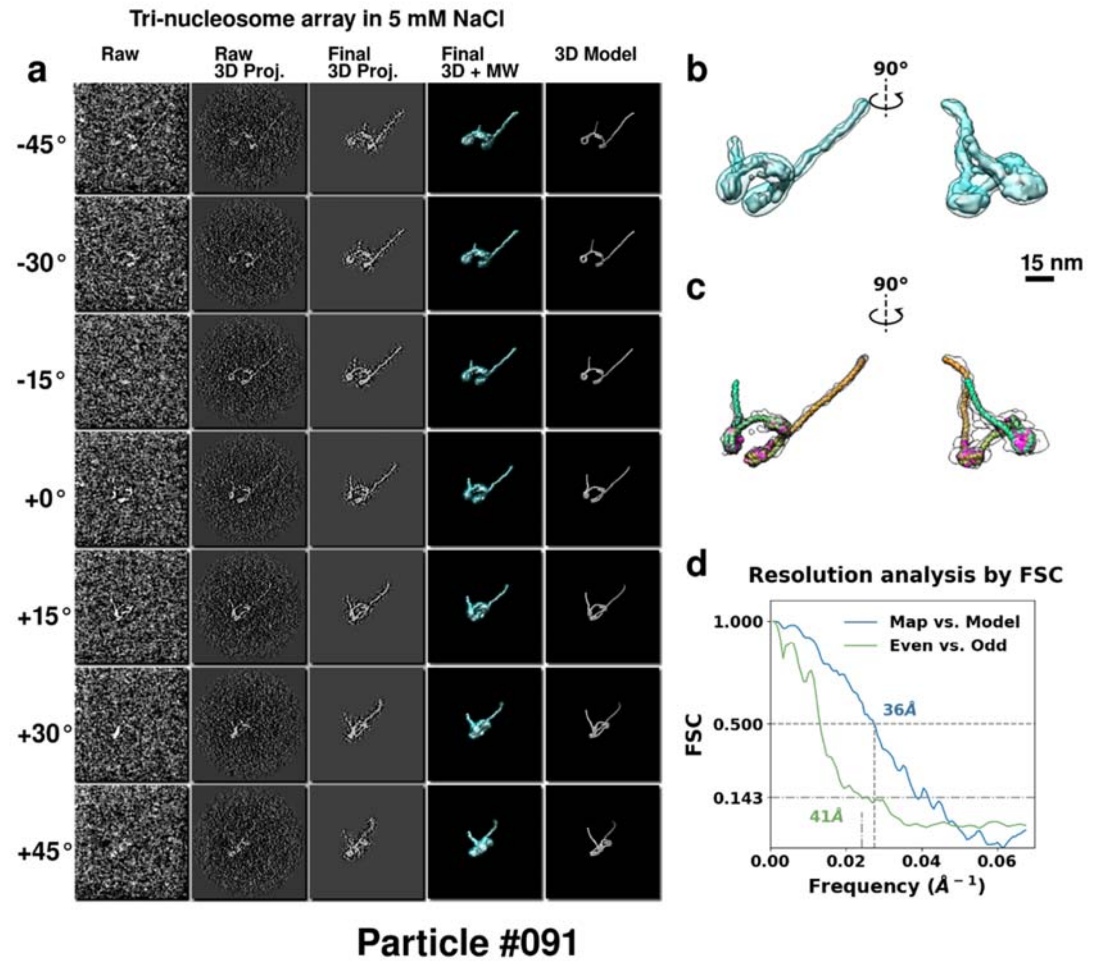

Supplementary Fig. 104. Cryo-ET 3D reconstruction of individual tri-nucleosome particle (index no. 91) in 5 mM NaCl. a, IPET 3D reconstruction of individual tri-nucleosome particles. The first column shows seven representative tilt images of an individual particle after CTF correction. Through alignment of the tilt images to a common center for 3D reconstruction via iterative refinement, the second and third columns display the 3D projections of the reconstruction before and after particle-shaped masking, respectively. The fourth column shows the final 3D reconstruction with missing wedge correction, and the fifth column presents the flexibly fitted model at the corresponding tilt angles. **b**, Zoomed-in view of the final 3D density map displayed in orthogonal views, shown at two contour levels. **c**, Superimposition of the high contour level map from (b) onto its flexibly fitted model. **d**, Resolution evaluation of the final 3D density map using two criteria: Fourier shell correlation (FSC) between two-half maps reconstructed from the even and odd index of the tilted series and FSC between the final 3D map and the fitted structure model. The resolution for the former and latter criteria is evaluated at frequencies of 0.5 and 0.143, respectively.

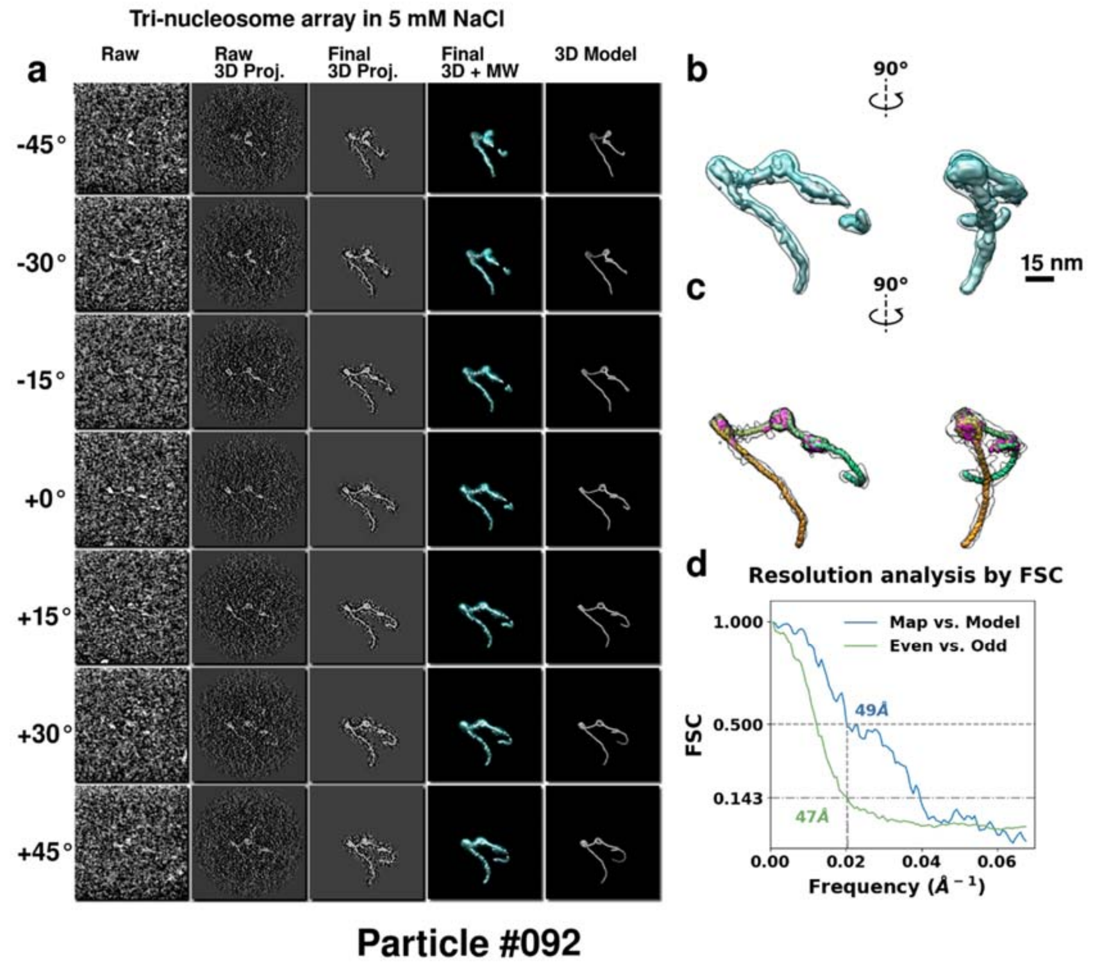

Supplementary Fig. 105. Cryo-ET 3D reconstruction of individual tri-nucleosome particle (index no. 92) in 5 mM NaCl. a, IPET 3D reconstruction of individual tri-nucleosome particles. The first column shows seven representative tilt images of an individual particle after CTF correction. Through alignment of the tilt images to a common center for 3D reconstruction via iterative refinement, the second and third columns display the 3D projections of the reconstruction before and after particle-shaped masking, respectively. The fourth column shows the final 3D reconstruction with missing wedge correction, and the fifth column presents the flexibly fitted model at the corresponding tilt angles. **b**, Zoomed-in view of the final 3D density map displayed in orthogonal views, shown at two contour levels. **c**, Superimposition of the high contour level map from (b) onto its flexibly fitted model. **d**, Resolution evaluation of the final 3D density map using two criteria: Fourier shell correlation (FSC) between two-half maps reconstructed from the even and odd index of the tilted series and FSC between the final 3D map and the fitted structure model. The resolution for the former and latter criteria is evaluated at frequencies of 0.5 and 0.143, respectively.

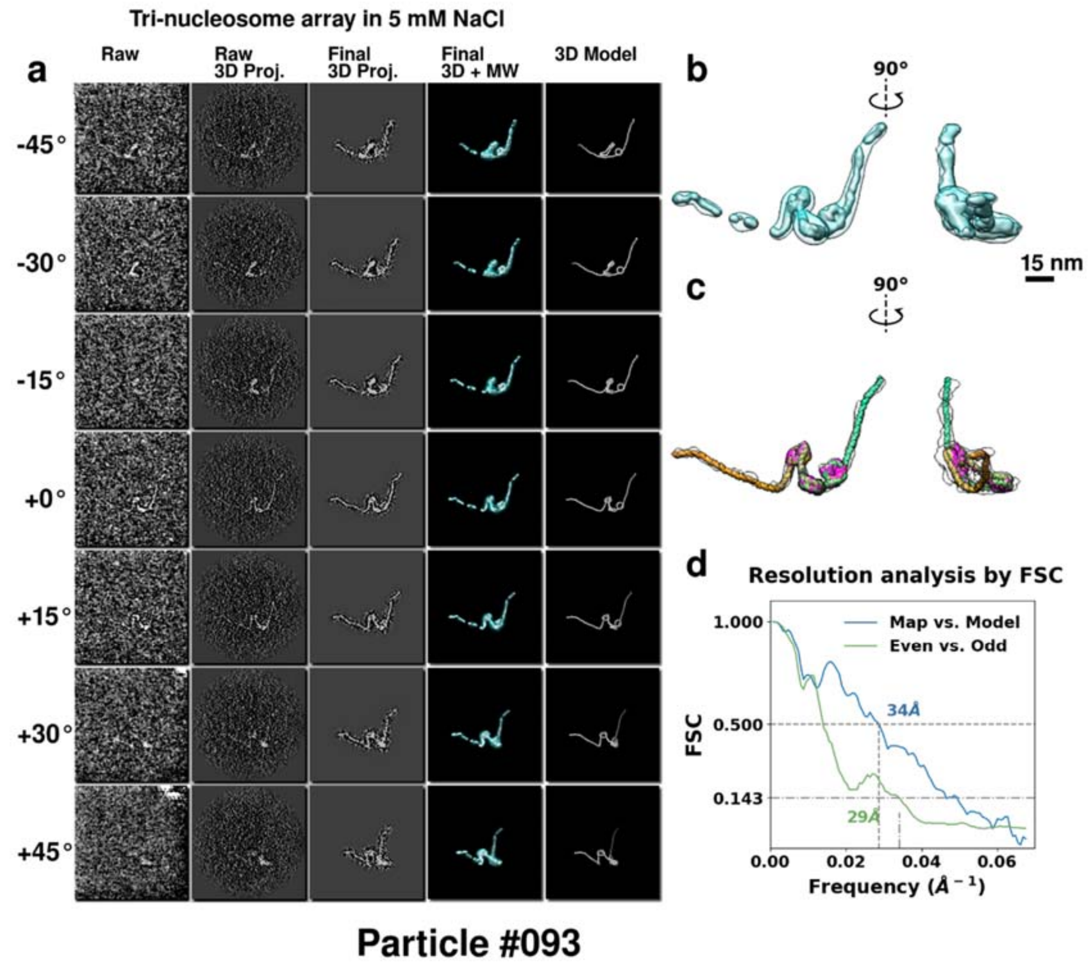

Supplementary Fig. 106. Cryo-ET 3D reconstruction of individual tri-nucleosome particle (index no. 93) in 5 mM NaCl. a, IPET 3D reconstruction of individual tri-nucleosome particles. The first column shows seven representative tilt images of an individual particle after CTF correction. Through alignment of the tilt images to a common center for 3D reconstruction via iterative refinement, the second and third columns display the 3D projections of the reconstruction before and after particle-shaped masking, respectively. The fourth column shows the final 3D reconstruction with missing wedge correction, and the fifth column presents the flexibly fitted model at the corresponding tilt angles. **b**, Zoomed-in view of the final 3D density map displayed in orthogonal views, shown at two contour levels. **c**, Superimposition of the high contour level map from (b) onto its flexibly fitted model. **d**, Resolution evaluation of the final 3D density map using two criteria: Fourier shell correlation (FSC) between two-half maps reconstructed from the even and odd index of the tilted series and FSC between the final 3D map and the fitted structure model. The resolution for the former and latter criteria is evaluated at frequencies of 0.5 and 0.143, respectively.

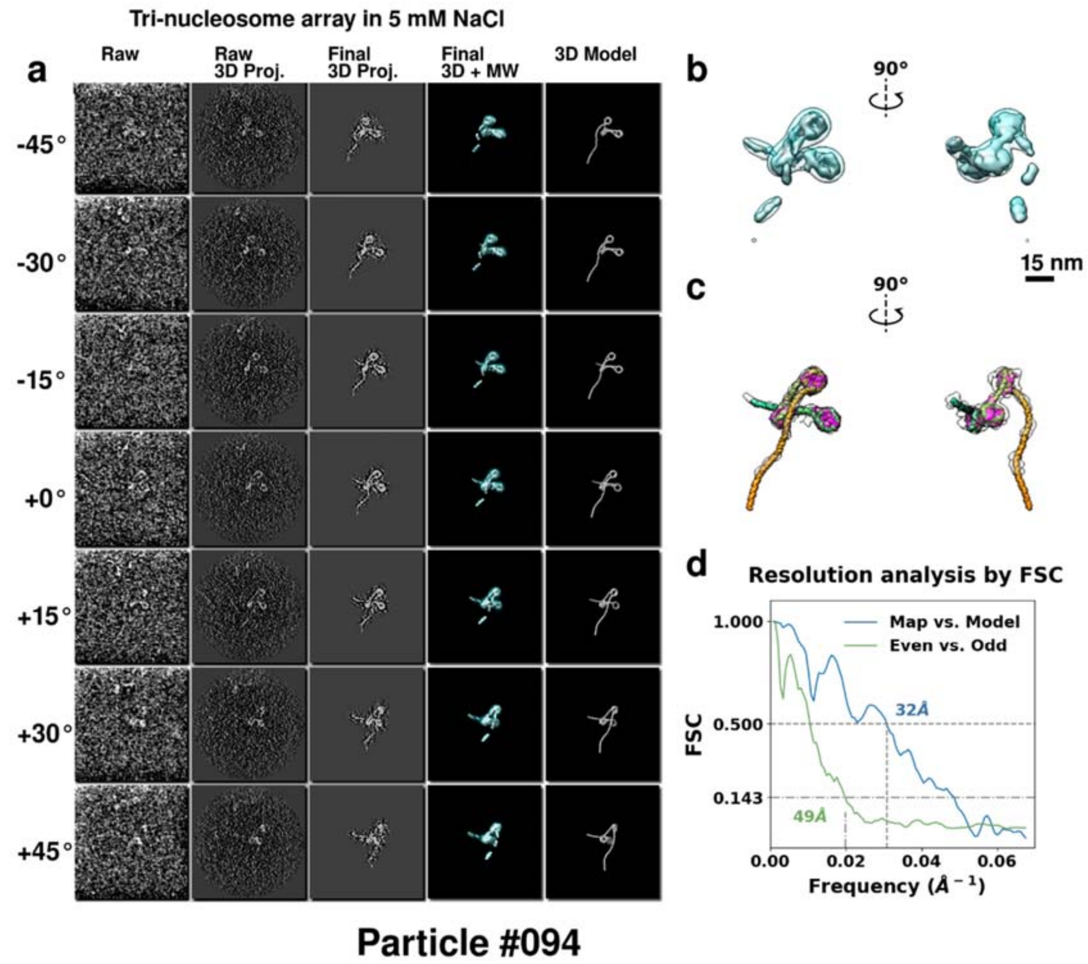

Supplementary Fig. 107. Cryo-ET 3D reconstruction of individual tri-nucleosome particle (index no. 94) in 5 mM NaCl. a, IPET 3D reconstruction of individual tri-nucleosome particles. The first column shows seven representative tilt images of an individual particle after CTF correction. Through alignment of the tilt images to a common center for 3D reconstruction via iterative refinement, the second and third columns display the 3D projections of the reconstruction before and after particle-shaped masking, respectively. The fourth column shows the final 3D reconstruction with missing wedge correction, and the fifth column presents the flexibly fitted model at the corresponding tilt angles. **b**, Zoomed-in view of the final 3D density map displayed in orthogonal views, shown at two contour levels. **c**, Superimposition of the high contour level map from (b) onto its flexibly fitted model. **d**, Resolution evaluation of the final 3D density map using two criteria: Fourier shell correlation (FSC) between two-half maps reconstructed from the even and odd index of the tilted series and FSC between the final 3D map and the fitted structure model. The resolution for the former and latter criteria is evaluated at frequencies of 0.5 and 0.143, respectively.

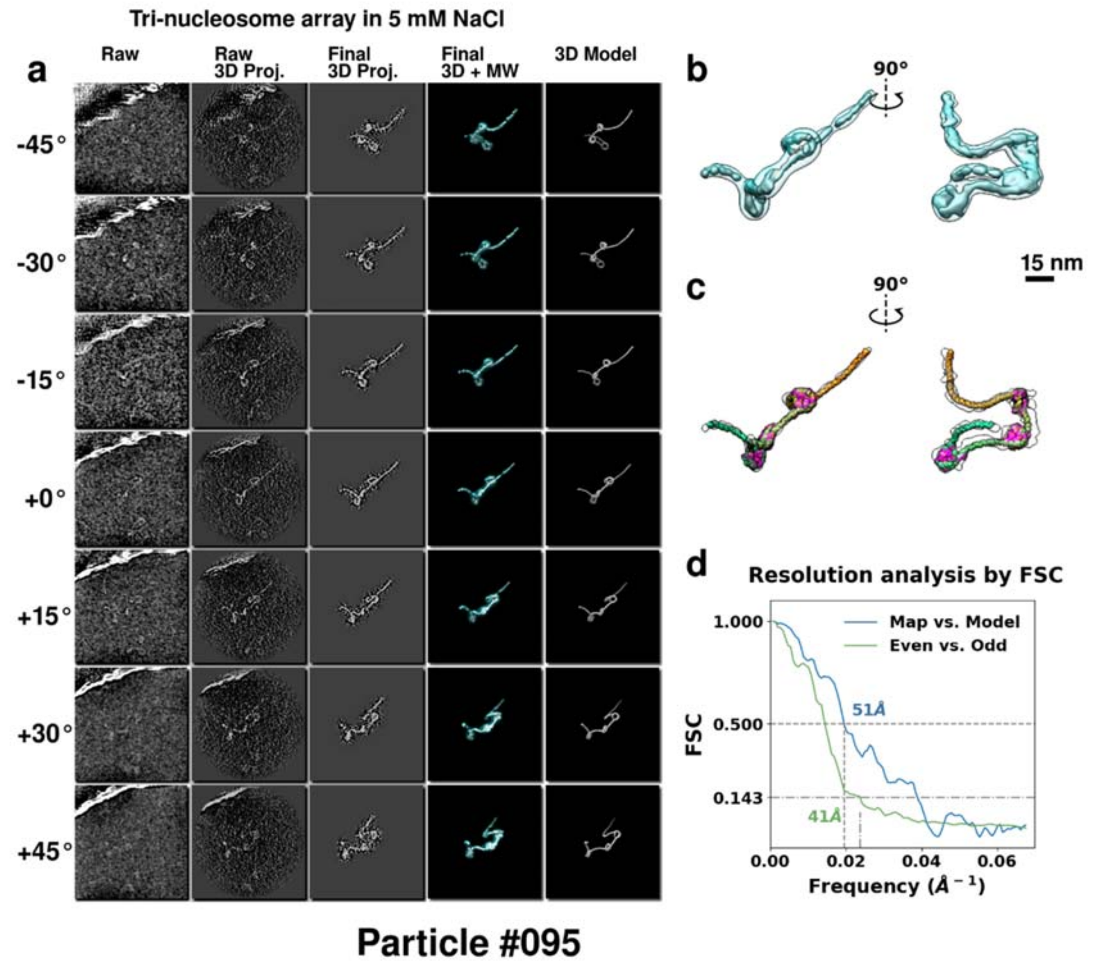

Supplementary Fig. 108. Cryo-ET 3D reconstruction of individual tri-nucleosome particle (index no. 95) in 5 mM NaCl. a, IPET 3D reconstruction of individual tri-nucleosome particles. The first column shows seven representative tilt images of an individual particle after CTF correction. Through alignment of the tilt images to a common center for 3D reconstruction via iterative refinement, the second and third columns display the 3D projections of the reconstruction before and after particle-shaped masking, respectively. The fourth column shows the final 3D reconstruction with missing wedge correction, and the fifth column presents the flexibly fitted model at the corresponding tilt angles. **b**, Zoomed-in view of the final 3D density map displayed in orthogonal views, shown at two contour levels. **c**, Superimposition of the high contour level map from (b) onto its flexibly fitted model. **d**, Resolution evaluation of the final 3D density map using two criteria: Fourier shell correlation (FSC) between two-half maps reconstructed from the even and odd index of the tilted series and FSC between the final 3D map and the fitted structure model. The resolution for the former and latter criteria is evaluated at frequencies of 0.5 and 0.143, respectively.

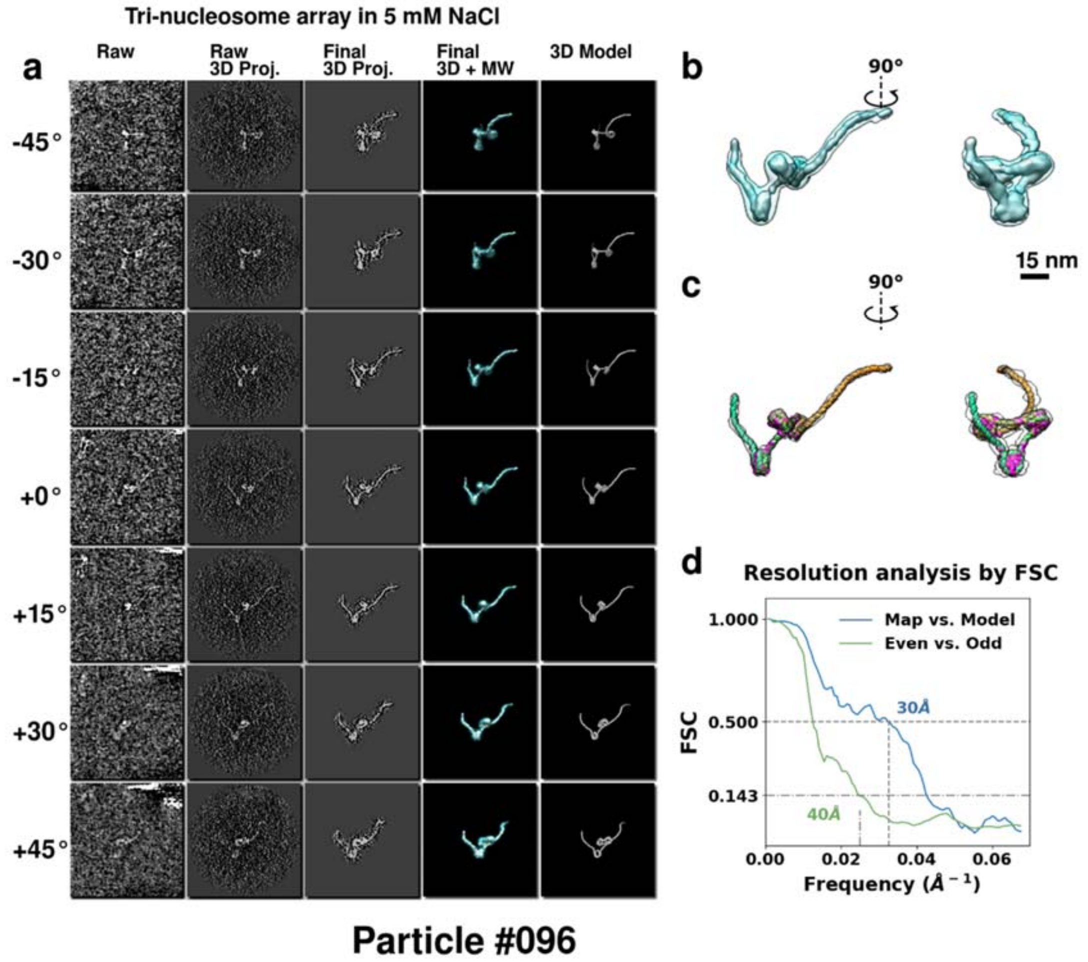

Supplementary Fig. 109. Cryo-ET 3D reconstruction of individual tri-nucleosome particle (index no. 96) in 5 mM NaCl. a, IPET 3D reconstruction of individual tri-nucleosome particles. The first column shows seven representative tilt images of an individual particle after CTF correction. Through alignment of the tilt images to a common center for 3D reconstruction via iterative refinement, the second and third columns display the 3D projections of the reconstruction before and after particle-shaped masking, respectively. The fourth column shows the final 3D reconstruction with missing wedge correction, and the fifth column presents the flexibly fitted model at the corresponding tilt angles. **b**, Zoomed-in view of the final 3D density map displayed in orthogonal views, shown at two contour levels. **c**, Superimposition of the high contour level map from (b) onto its flexibly fitted model. **d**, Resolution evaluation of the final 3D density map using two criteria: Fourier shell correlation (FSC) between two-half maps reconstructed from the even and odd index of the tilted series and FSC between the final 3D map and the fitted structure model. The resolution for the former and latter criteria is evaluated at frequencies of 0.5 and 0.143, respectively.

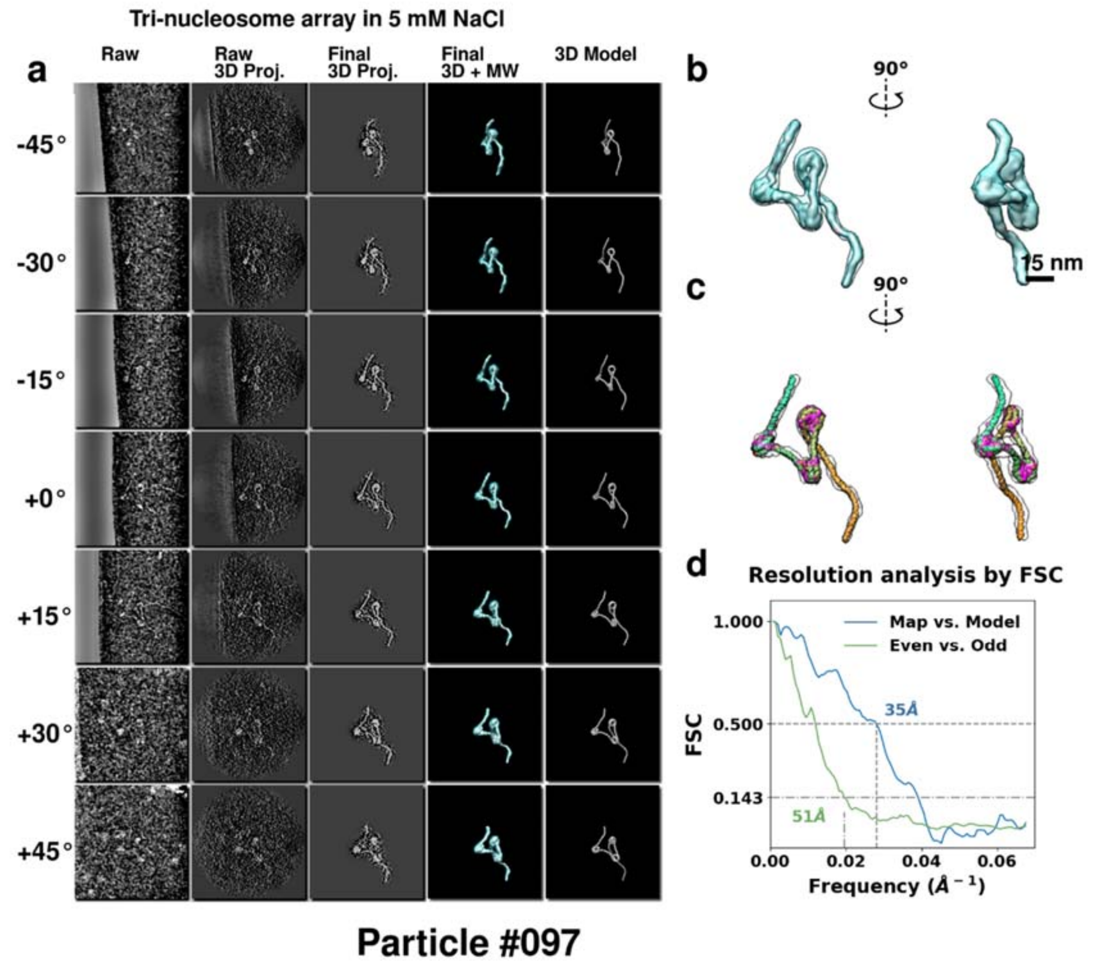

Supplementary Fig. 110. Cryo-ET 3D reconstruction of individual tri-nucleosome particle (index no. 97) in 5 mM NaCl. a, IPET 3D reconstruction of individual tri-nucleosome particles. The first column shows seven representative tilt images of an individual particle after CTF correction. Through alignment of the tilt images to a common center for 3D reconstruction via iterative refinement, the second and third columns display the 3D projections of the reconstruction before and after particle-shaped masking, respectively. The fourth column shows the final 3D reconstruction with missing wedge correction, and the fifth column presents the flexibly fitted model at the corresponding tilt angles. **b**, Zoomed-in view of the final 3D density map displayed in orthogonal views, shown at two contour levels. **c**, Superimposition of the high contour level map from (b) onto its flexibly fitted model. **d**, Resolution evaluation of the final 3D density map using two criteria: Fourier shell correlation (FSC) between two-half maps reconstructed from the even and odd index of the tilted series and FSC between the final 3D map and the fitted structure model. The resolution for the former and latter criteria is evaluated at frequencies of 0.5 and 0.143, respectively.

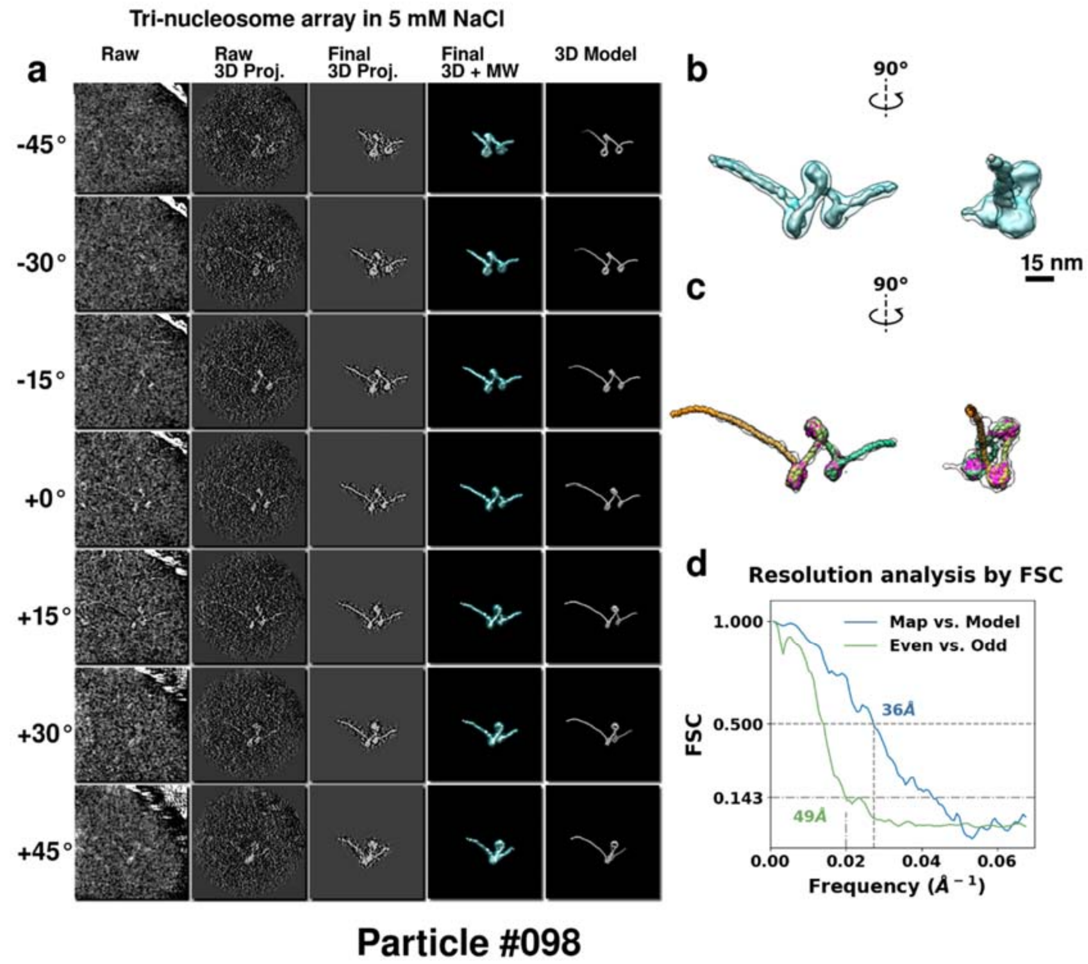

Supplementary Fig. 111. Cryo-ET 3D reconstruction of individual tri-nucleosome particle (index no. 98) in 5 mM NaCl. a, IPET 3D reconstruction of individual tri-nucleosome particles. The first column shows seven representative tilt images of an individual particle after CTF correction. Through alignment of the tilt images to a common center for 3D reconstruction via iterative refinement, the second and third columns display the 3D projections of the reconstruction before and after particle-shaped masking, respectively. The fourth column shows the final 3D reconstruction with missing wedge correction, and the fifth column presents the flexibly fitted model at the corresponding tilt angles. **b**, Zoomed-in view of the final 3D density map displayed in orthogonal views, shown at two contour levels. **c**, Superimposition of the high contour level map from (b) onto its flexibly fitted model. **d**, Resolution evaluation of the final 3D density map using two criteria: Fourier shell correlation (FSC) between two-half maps reconstructed from the even and odd index of the tilted series and FSC between the final 3D map and the fitted structure model. The resolution for the former and latter criteria is evaluated at frequencies of 0.5 and 0.143, respectively.

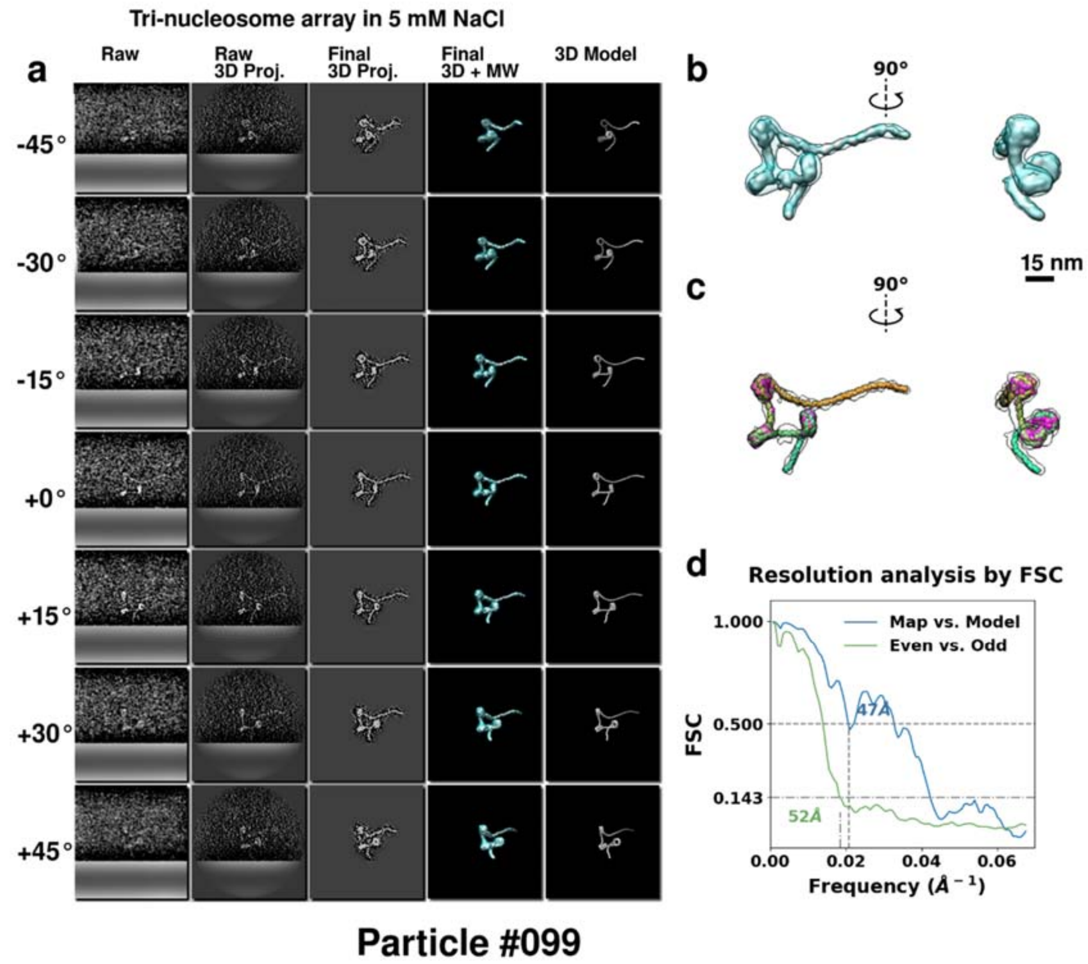

Supplementary Fig. 112. Cryo-ET 3D reconstruction of individual tri-nucleosome particle (index no. 99) in 5 mM NaCl. a, IPET 3D reconstruction of individual tri-nucleosome particles. The first column shows seven representative tilt images of an individual particle after CTF correction. Through alignment of the tilt images to a common center for 3D reconstruction via iterative refinement, the second and third columns display the 3D projections of the reconstruction before and after particle-shaped masking, respectively. The fourth column shows the final 3D reconstruction with missing wedge correction, and the fifth column presents the flexibly fitted model at the corresponding tilt angles. **b**, Zoomed-in view of the final 3D density map displayed in orthogonal views, shown at two contour levels. **c**, Superimposition of the high contour level map from (b) onto its flexibly fitted model. **d**, Resolution evaluation of the final 3D density map using two criteria: Fourier shell correlation (FSC) between two-half maps reconstructed from the even and odd index of the tilted series and FSC between the final 3D map and the fitted structure model. The resolution for the former and latter criteria is evaluated at frequencies of 0.5 and 0.143, respectively.

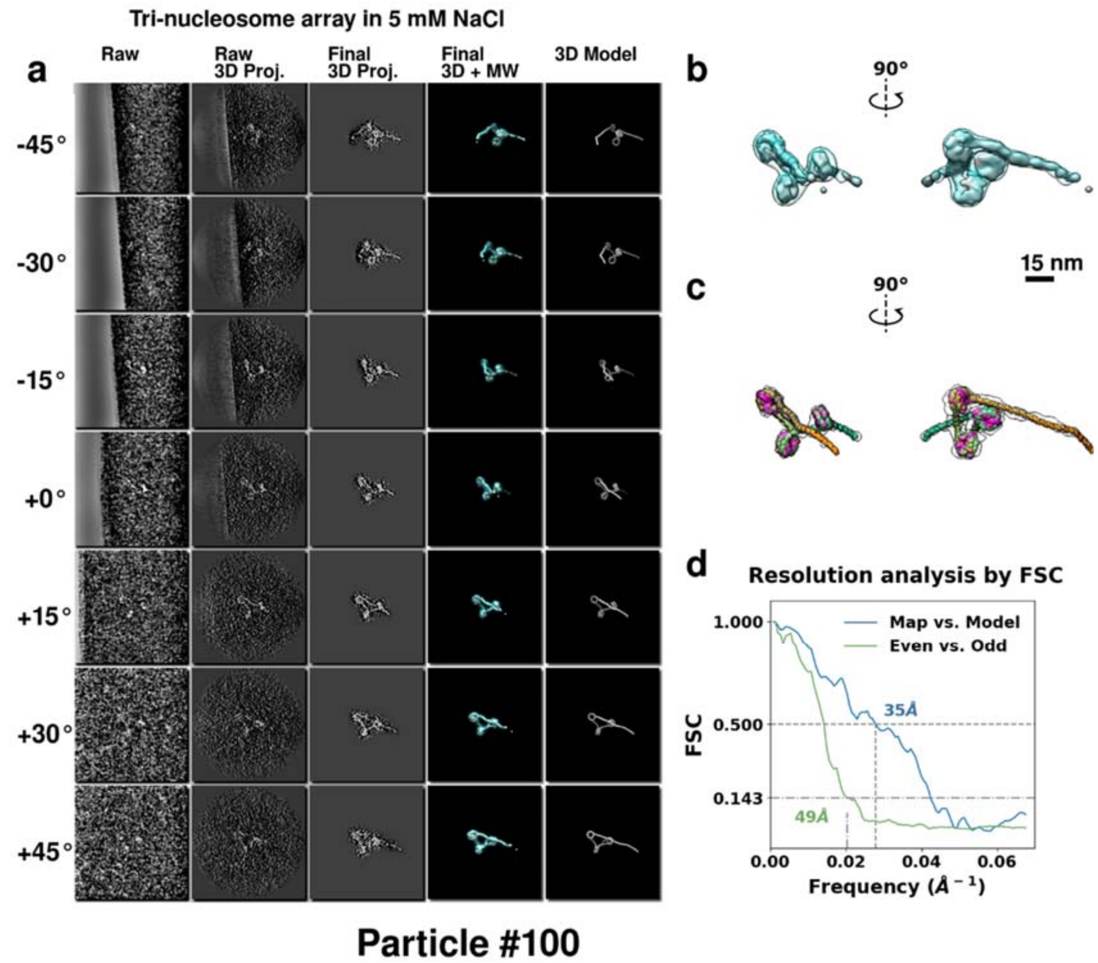

Supplementary Fig. 113. Cryo-ET 3D reconstruction of individual tri-nucleosome particle (index no. 100) in 5 mM NaCl. a, IPET 3D reconstruction of individual tri-nucleosome particles. The first column shows seven representative tilt images of an individual particle after CTF correction. Through alignment of the tilt images to a common center for 3D reconstruction via iterative refinement, the second and third columns display the 3D projections of the reconstruction before and after particle-shaped masking, respectively. The fourth column shows the final 3D reconstruction with missing wedge correction, and the fifth column presents the flexibly fitted model at the corresponding tilt angles. **b**, Zoomed-in view of the final 3D density map displayed in orthogonal views, shown at two contour levels. **c**, Superimposition of the high contour level map from (b) onto its flexibly fitted model. **d**, Resolution evaluation of the final 3D density map using two criteria: Fourier shell correlation (FSC) between two-half maps reconstructed from the even and odd index of the tilted series and FSC between the final 3D map and the fitted structure model. The resolution for the former and latter criteria is evaluated at frequencies of 0.5 and 0.143, respectively.

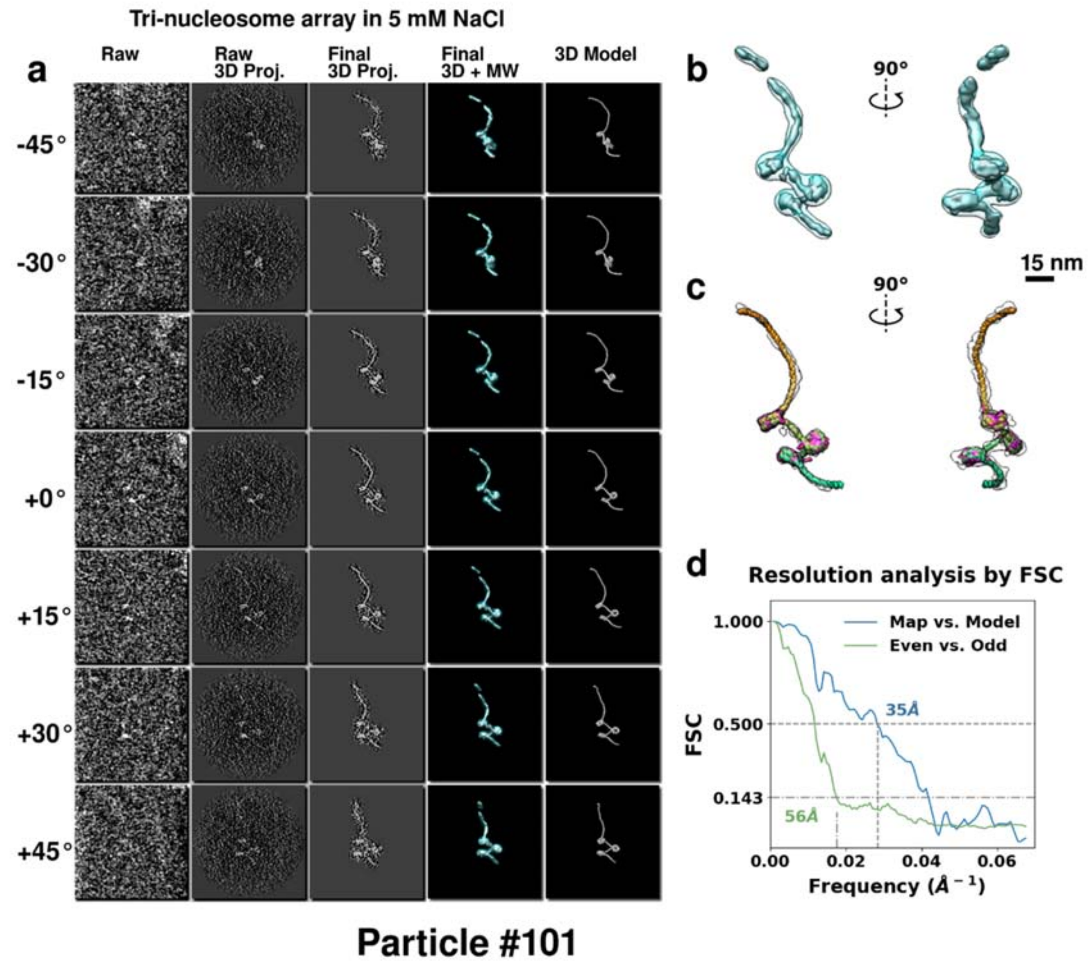

Supplementary Fig. 114. Cryo-ET 3D reconstruction of individual tri-nucleosome particle (index no. 101) in 5 mM NaCl. a, IPET 3D reconstruction of individual tri-nucleosome particles. The first column shows seven representative tilt images of an individual particle after CTF correction. Through alignment of the tilt images to a common center for 3D reconstruction via iterative refinement, the second and third columns display the 3D projections of the reconstruction before and after particle-shaped masking, respectively. The fourth column shows the final 3D reconstruction with missing wedge correction, and the fifth column presents the flexibly fitted model at the corresponding tilt angles. **b**, Zoomed-in view of the final 3D density map displayed in orthogonal views, shown at two contour levels. **c**, Superimposition of the high contour level map from (b) onto its flexibly fitted model. **d**, Resolution evaluation of the final 3D density map using two criteria: Fourier shell correlation (FSC) between two-half maps reconstructed from the even and odd index of the tilted series and FSC between the final 3D map and the fitted structure model. The resolution for the former and latter criteria is evaluated at frequencies of 0.5 and 0.143, respectively.

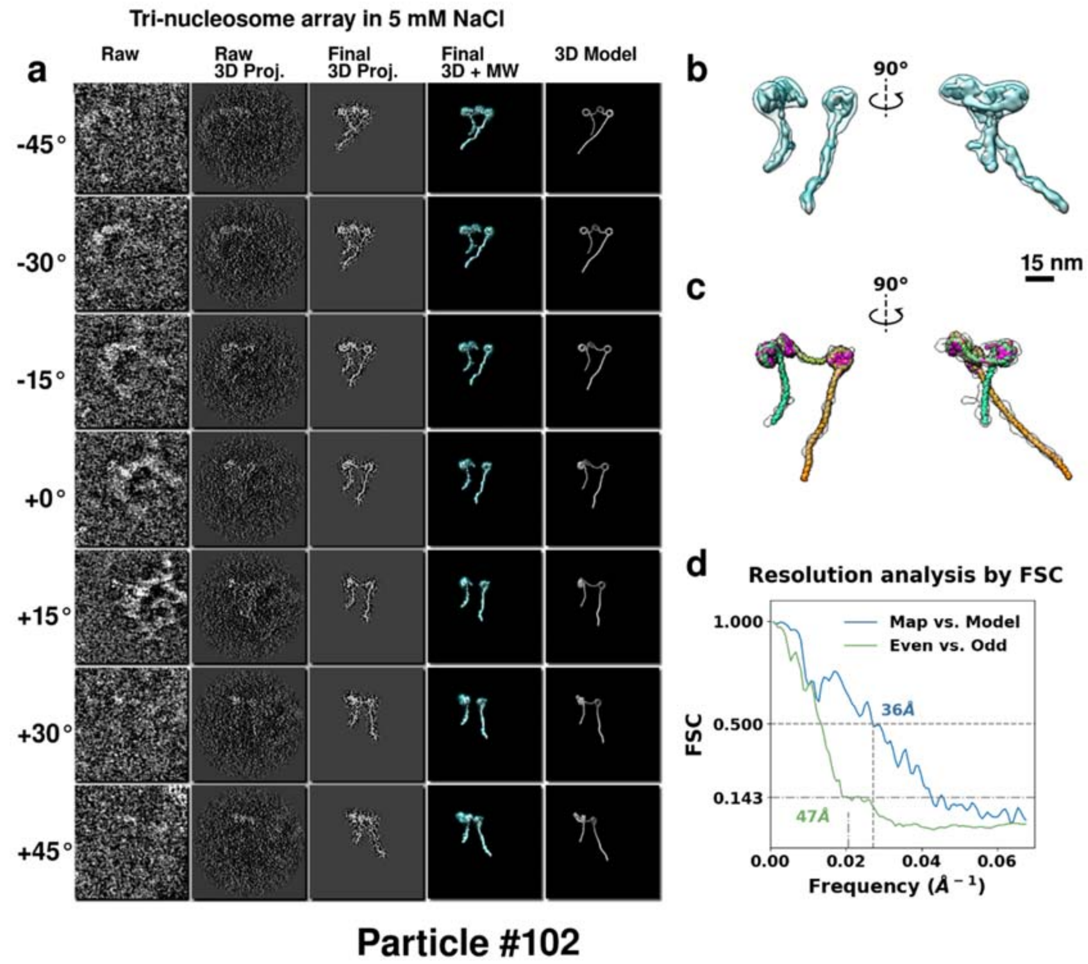

Supplementary Fig. 115. Cryo-ET 3D reconstruction of individual tri-nucleosome particle (index no. 102) in 5 mM NaCl. a, IPET 3D reconstruction of individual tri-nucleosome particles. The first column shows seven representative tilt images of an individual particle after CTF correction. Through alignment of the tilt images to a common center for 3D reconstruction via iterative refinement, the second and third columns display the 3D projections of the reconstruction before and after particle-shaped masking, respectively. The fourth column shows the final 3D reconstruction with missing wedge correction, and the fifth column presents the flexibly fitted model at the corresponding tilt angles. **b**, Zoomed-in view of the final 3D density map displayed in orthogonal views, shown at two contour levels. **c**, Superimposition of the high contour level map from (b) onto its flexibly fitted model. **d**, Resolution evaluation of the final 3D density map using two criteria: Fourier shell correlation (FSC) between two-half maps reconstructed from the even and odd index of the tilted series and FSC between the final 3D map and the fitted structure model. The resolution for the former and latter criteria is evaluated at frequencies of 0.5 and 0.143, respectively.

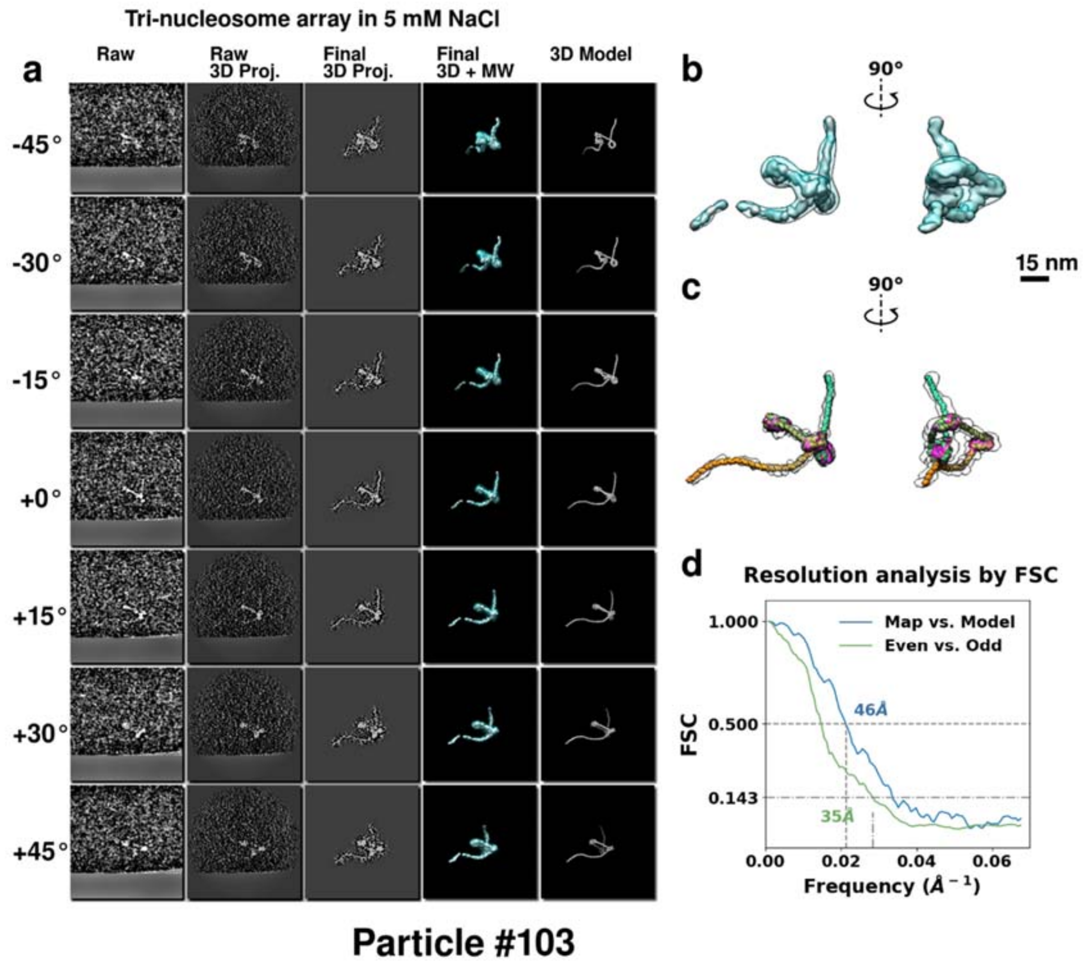

Supplementary Fig. 116. Cryo-ET 3D reconstruction of individual tri-nucleosome particle (index no. 103) in 5 mM NaCl. a, IPET 3D reconstruction of individual tri-nucleosome particles. The first column shows seven representative tilt images of an individual particle after CTF correction. Through alignment of the tilt images to a common center for 3D reconstruction via iterative refinement, the second and third columns display the 3D projections of the reconstruction before and after particle-shaped masking, respectively. The fourth column shows the final 3D reconstruction with missing wedge correction, and the fifth column presents the flexibly fitted model at the corresponding tilt angles. **b**, Zoomed-in view of the final 3D density map displayed in orthogonal views, shown at two contour levels. **c**, Superimposition of the high contour level map from (b) onto its flexibly fitted model. **d**, Resolution evaluation of the final 3D density map using two criteria: Fourier shell correlation (FSC) between two-half maps reconstructed from the even and odd index of the tilted series and FSC between the final 3D map and the fitted structure model. The resolution for the former and latter criteria is evaluated at frequencies of 0.5 and 0.143, respectively.

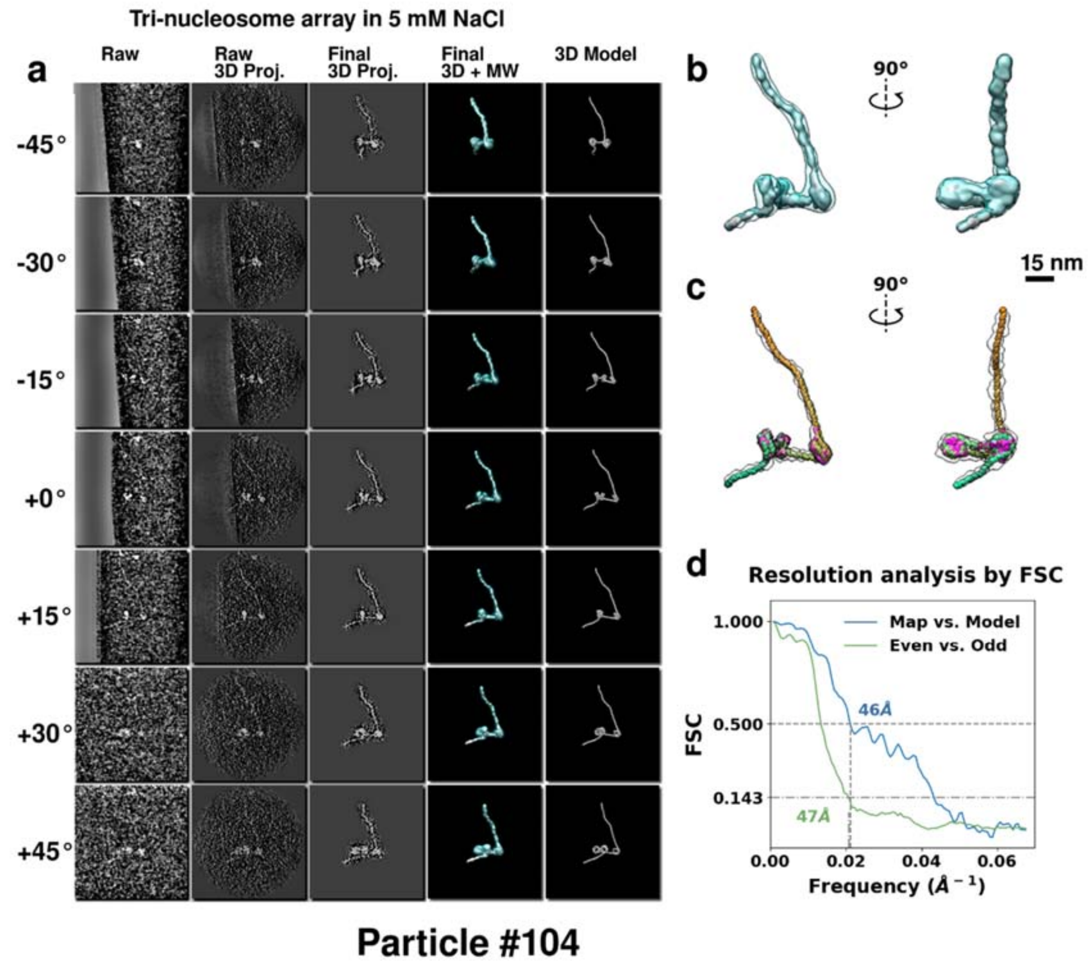

Supplementary Fig. 117. Cryo-ET 3D reconstruction of individual tri-nucleosome particle (index no. 104) in 5 mM NaCl. a, IPET 3D reconstruction of individual tri-nucleosome particles. The first column shows seven representative tilt images of an individual particle after CTF correction. Through alignment of the tilt images to a common center for 3D reconstruction via iterative refinement, the second and third columns display the 3D projections of the reconstruction before and after particle-shaped masking, respectively. The fourth column shows the final 3D reconstruction with missing wedge correction, and the fifth column presents the flexibly fitted model at the corresponding tilt angles. **b**, Zoomed-in view of the final 3D density map displayed in orthogonal views, shown at two contour levels. **c**, Superimposition of the high contour level map from (b) onto its flexibly fitted model. **d**, Resolution evaluation of the final 3D density map using two criteria: Fourier shell correlation (FSC) between two-half maps reconstructed from the even and odd index of the tilted series and FSC between the final 3D map and the fitted structure model. The resolution for the former and latter criteria is evaluated at frequencies of 0.5 and 0.143, respectively.

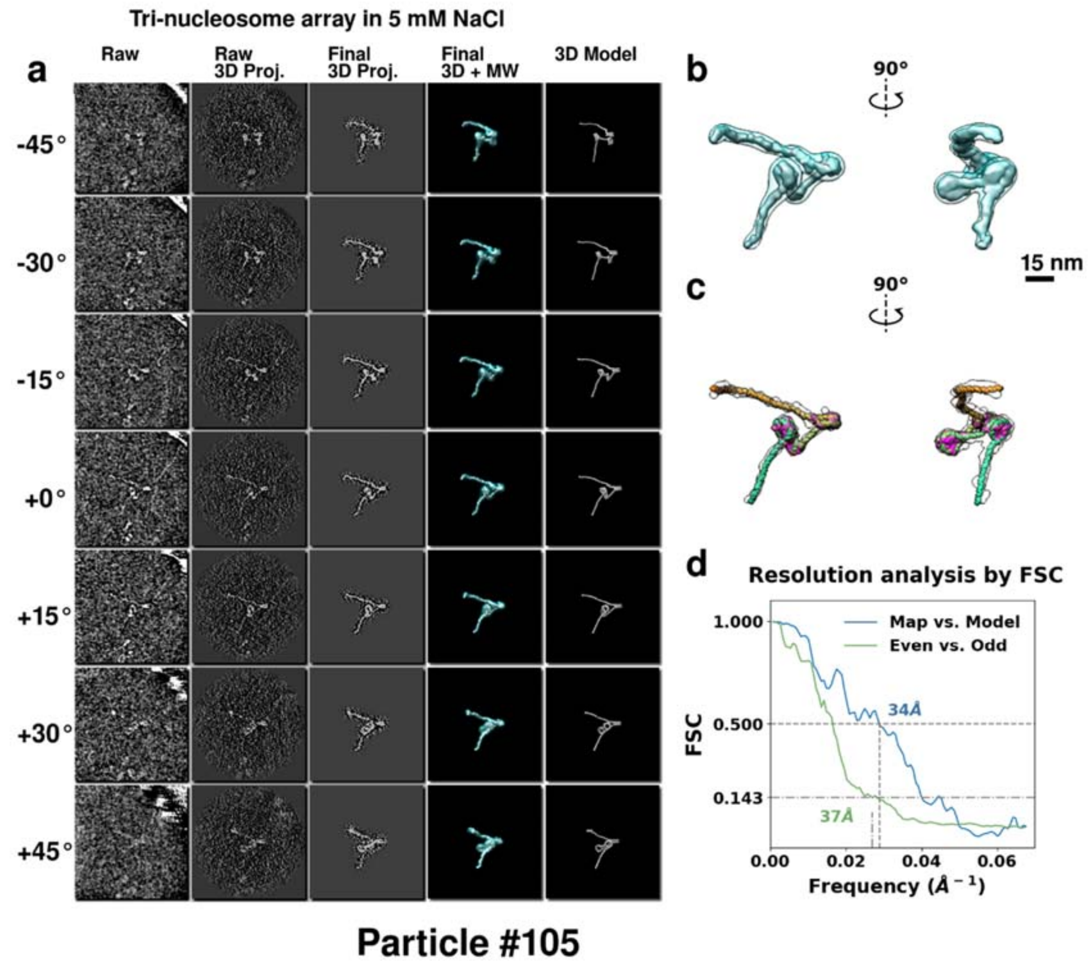

Supplementary Fig. 118. Cryo-ET 3D reconstruction of individual tri-nucleosome particle (index no. 105) in 5 mM NaCl. a, IPET 3D reconstruction of individual tri-nucleosome particles. The first column shows seven representative tilt images of an individual particle after CTF correction. Through alignment of the tilt images to a common center for 3D reconstruction via iterative refinement, the second and third columns display the 3D projections of the reconstruction before and after particle-shaped masking, respectively. The fourth column shows the final 3D reconstruction with missing wedge correction, and the fifth column presents the flexibly fitted model at the corresponding tilt angles. **b**, Zoomed-in view of the final 3D density map displayed in orthogonal views, shown at two contour levels. **c**, Superimposition of the high contour level map from (b) onto its flexibly fitted model. **d**, Resolution evaluation of the final 3D density map using two criteria: Fourier shell correlation (FSC) between two-half maps reconstructed from the even and odd index of the tilted series and FSC between the final 3D map and the fitted structure model. The resolution for the former and latter criteria is evaluated at frequencies of 0.5 and 0.143, respectively.

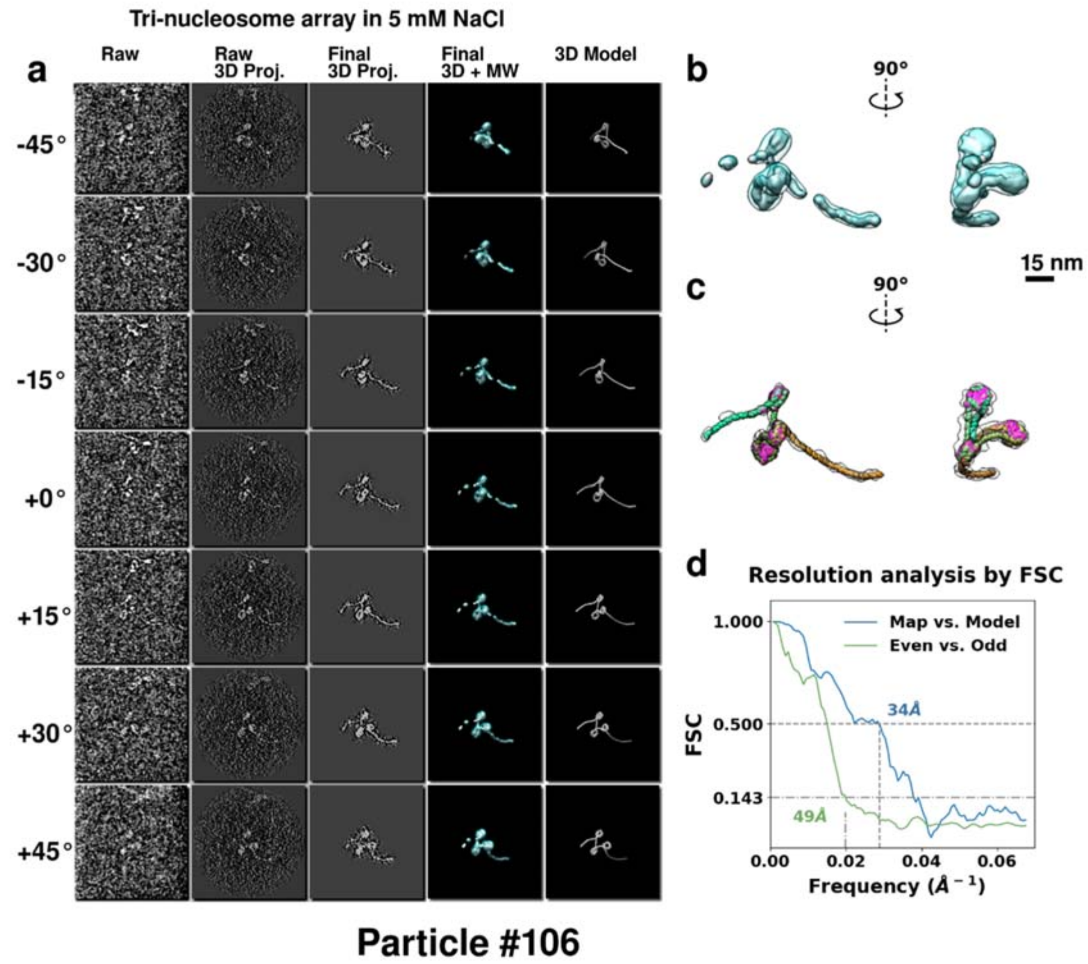

Supplementary Fig. 119. Cryo-ET 3D reconstruction of individual tri-nucleosome particle (index no. 106) in 5 mM NaCl. a, IPET 3D reconstruction of individual tri-nucleosome particles. The first column shows seven representative tilt images of an individual particle after CTF correction. Through alignment of the tilt images to a common center for 3D reconstruction via iterative refinement, the second and third columns display the 3D projections of the reconstruction before and after particle-shaped masking, respectively. The fourth column shows the final 3D reconstruction with missing wedge correction, and the fifth column presents the flexibly fitted model at the corresponding tilt angles. **b**, Zoomed-in view of the final 3D density map displayed in orthogonal views, shown at two contour levels. **c**, Superimposition of the high contour level map from (b) onto its flexibly fitted model. **d**, Resolution evaluation of the final 3D density map using two criteria: Fourier shell correlation (FSC) between two-half maps reconstructed from the even and odd index of the tilted series and FSC between the final 3D map and the fitted structure model. The resolution for the former and latter criteria is evaluated at frequencies of 0.5 and 0.143, respectively.

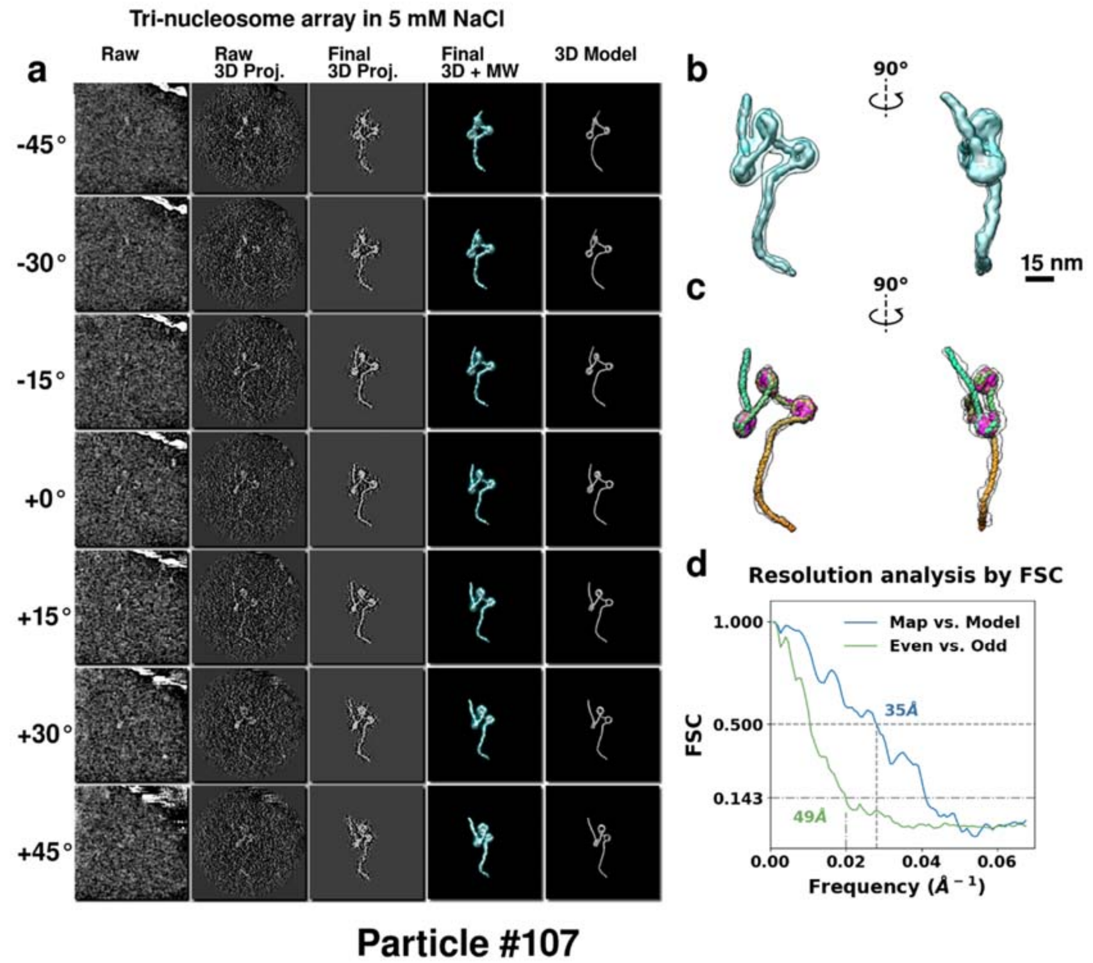

Supplementary Fig. 120. Cryo-ET 3D reconstruction of individual tri-nucleosome particle (index no. 107) in 5 mM NaCl. a, IPET 3D reconstruction of individual tri-nucleosome particles. The first column shows seven representative tilt images of an individual particle after CTF correction. Through alignment of the tilt images to a common center for 3D reconstruction via iterative refinement, the second and third columns display the 3D projections of the reconstruction before and after particle-shaped masking, respectively. The fourth column shows the final 3D reconstruction with missing wedge correction, and the fifth column presents the flexibly fitted model at the corresponding tilt angles. **b**, Zoomed-in view of the final 3D density map displayed in orthogonal views, shown at two contour levels. **c**, Superimposition of the high contour level map from (b) onto its flexibly fitted model. **d**, Resolution evaluation of the final 3D density map using two criteria: Fourier shell correlation (FSC) between two-half maps reconstructed from the even and odd index of the tilted series and FSC between the final 3D map and the fitted structure model. The resolution for the former and latter criteria is evaluated at frequencies of 0.5 and 0.143, respectively.

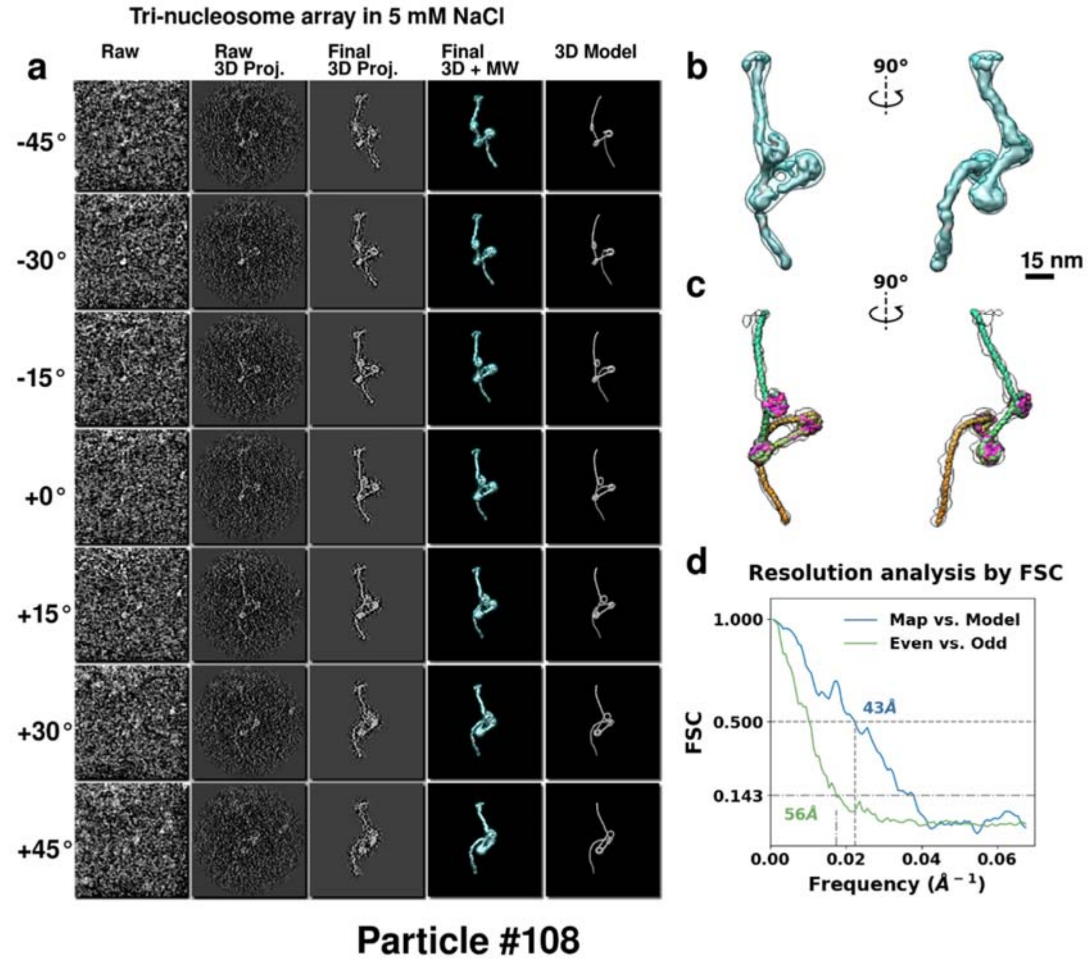

Supplementary Fig. 121. Cryo-ET 3D reconstruction of individual tri-nucleosome particle (index no. 108) in 5 mM NaCl. a, IPET 3D reconstruction of individual tri-nucleosome particles. The first column shows seven representative tilt images of an individual particle after CTF correction. Through alignment of the tilt images to a common center for 3D reconstruction via iterative refinement, the second and third columns display the 3D projections of the reconstruction before and after particle-shaped masking, respectively. The fourth column shows the final 3D reconstruction with missing wedge correction, and the fifth column presents the flexibly fitted model at the corresponding tilt angles. **b**, Zoomed-in view of the final 3D density map displayed in orthogonal views, shown at two contour levels. **c**, Superimposition of the high contour level map from (b) onto its flexibly fitted model. **d**, Resolution evaluation of the final 3D density map using two criteria: Fourier shell correlation (FSC) between two-half maps reconstructed from the even and odd index of the tilted series and FSC between the final 3D map and the fitted structure model. The resolution for the former and latter criteria is evaluated at frequencies of 0.5 and 0.143, respectively.

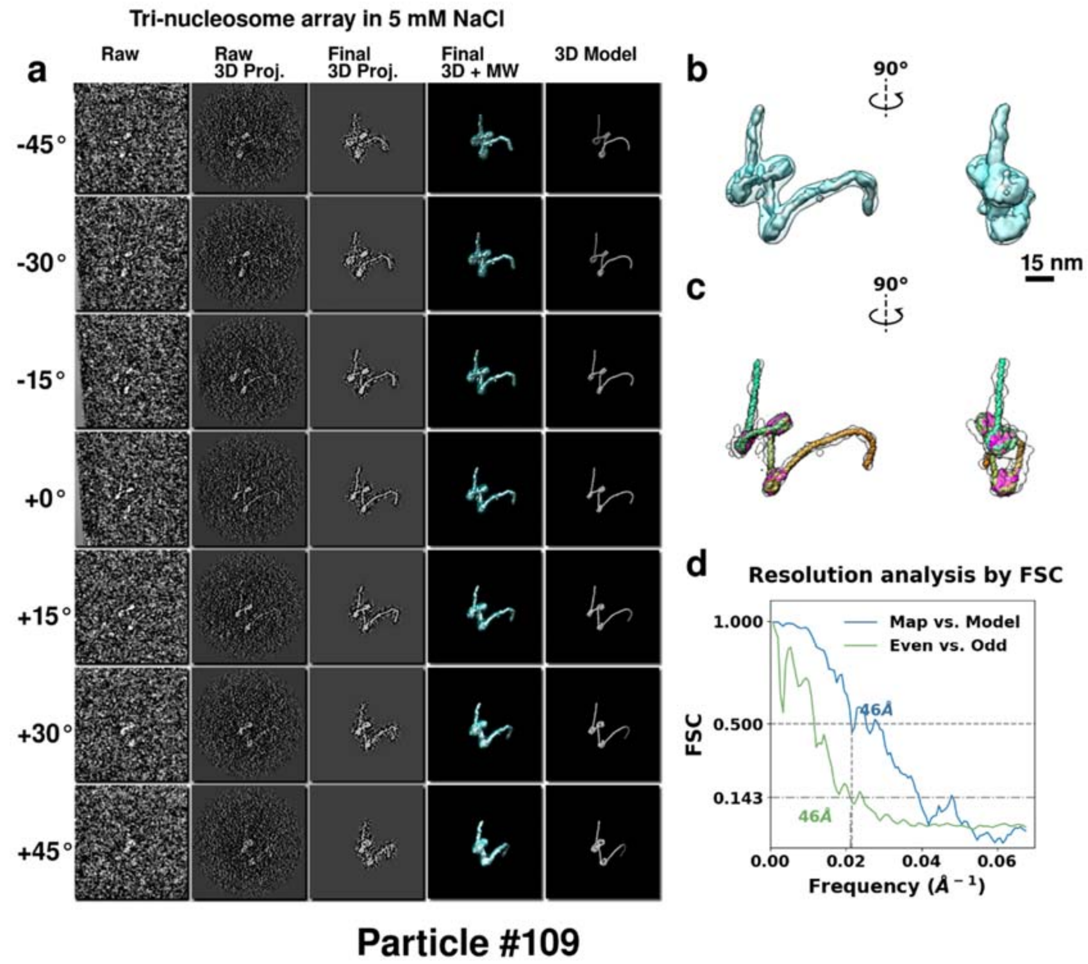

Supplementary Fig. 122. Cryo-ET 3D reconstruction of individual tri-nucleosome particle (index no. 109) in 5 mM NaCl. a, IPET 3D reconstruction of individual tri-nucleosome particles. The first column shows seven representative tilt images of an individual particle after CTF correction. Through alignment of the tilt images to a common center for 3D reconstruction via iterative refinement, the second and third columns display the 3D projections of the reconstruction before and after particle-shaped masking, respectively. The fourth column shows the final 3D reconstruction with missing wedge correction, and the fifth column presents the flexibly fitted model at the corresponding tilt angles. **b**, Zoomed-in view of the final 3D density map displayed in orthogonal views, shown at two contour levels. **c**, Superimposition of the high contour level map from (b) onto its flexibly fitted model. **d**, Resolution evaluation of the final 3D density map using two criteria: Fourier shell correlation (FSC) between two-half maps reconstructed from the even and odd index of the tilted series and FSC between the final 3D map and the fitted structure model. The resolution for the former and latter criteria is evaluated at frequencies of 0.5 and 0.143, respectively.

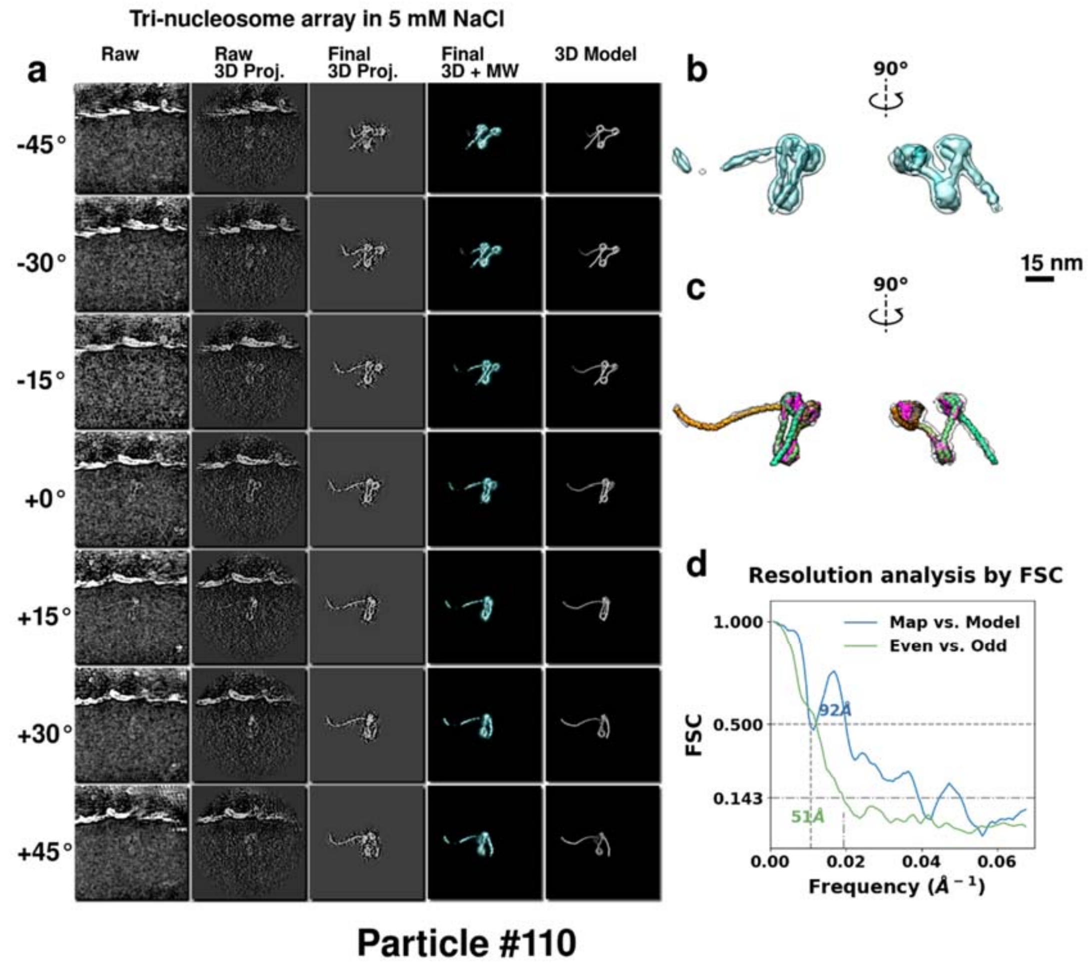

Supplementary Fig. 123. Cryo-ET 3D reconstruction of individual tri-nucleosome particle (index no. 110) in 5 mM NaCl. a, IPET 3D reconstruction of individual tri-nucleosome particles. The first column shows seven representative tilt images of an individual particle after CTF correction. Through alignment of the tilt images to a common center for 3D reconstruction via iterative refinement, the second and third columns display the 3D projections of the reconstruction before and after particle-shaped masking, respectively. The fourth column shows the final 3D reconstruction with missing wedge correction, and the fifth column presents the flexibly fitted model at the corresponding tilt angles. **b**, Zoomed-in view of the final 3D density map displayed in orthogonal views, shown at two contour levels. **c**, Superimposition of the high contour level map from (b) onto its flexibly fitted model. **d**, Resolution evaluation of the final 3D density map using two criteria: Fourier shell correlation (FSC) between two-half maps reconstructed from the even and odd index of the tilted series and FSC between the final 3D map and the fitted structure model. The resolution for the former and latter criteria is evaluated at frequencies of 0.5 and 0.143, respectively.

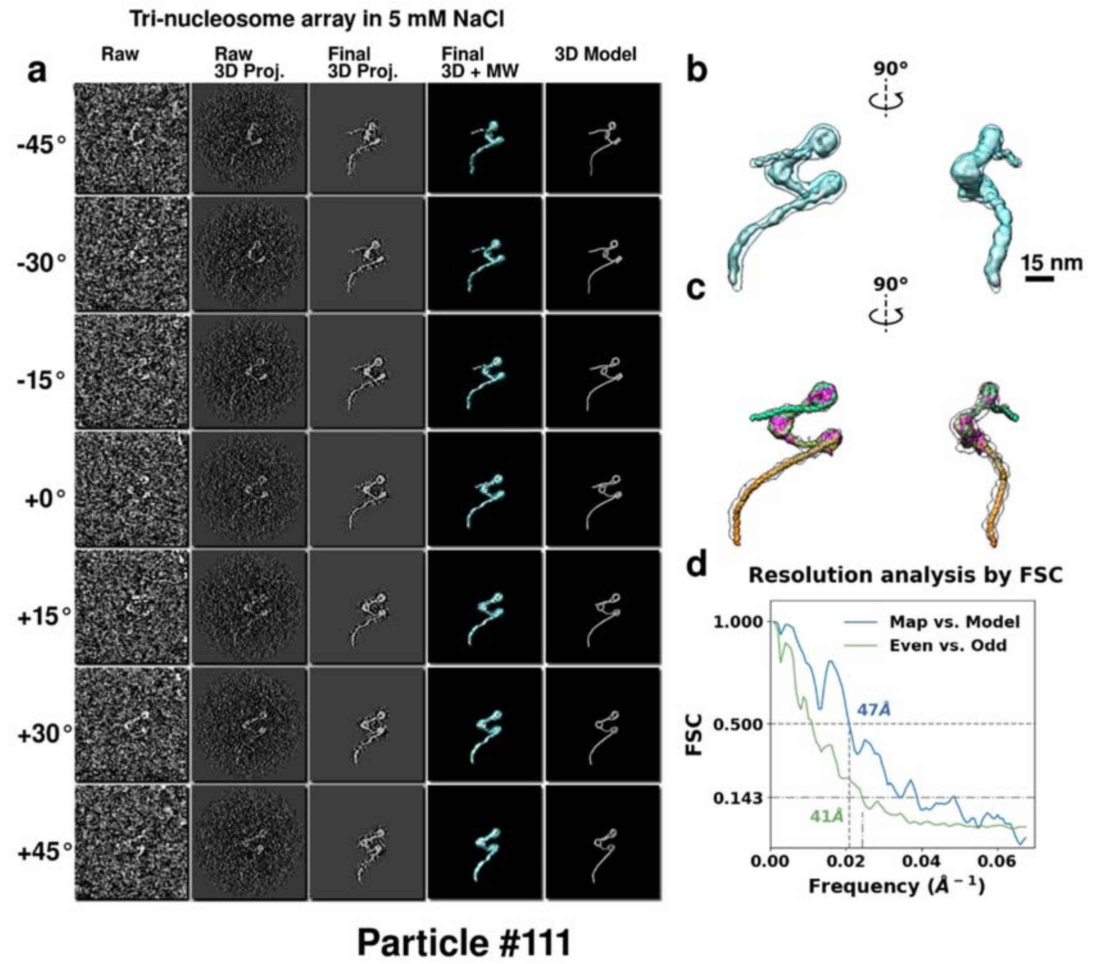

Supplementary Fig. 124. Cryo-ET 3D reconstruction of individual tri-nucleosome particle (index no. 111) in 5 mM NaCl. a, IPET 3D reconstruction of individual tri-nucleosome particles. The first column shows seven representative tilt images of an individual particle after CTF correction. Through alignment of the tilt images to a common center for 3D reconstruction via iterative refinement, the second and third columns display the 3D projections of the reconstruction before and after particle-shaped masking, respectively. The fourth column shows the final 3D reconstruction with missing wedge correction, and the fifth column presents the flexibly fitted model at the corresponding tilt angles. **b**, Zoomed-in view of the final 3D density map displayed in orthogonal views, shown at two contour levels. **c**, Superimposition of the high contour level map from (b) onto its flexibly fitted model. **d**, Resolution evaluation of the final 3D density map using two criteria: Fourier shell correlation (FSC) between two-half maps reconstructed from the even and odd index of the tilted series and FSC between the final 3D map and the fitted structure model. The resolution for the former and latter criteria is evaluated at frequencies of 0.5 and 0.143, respectively.

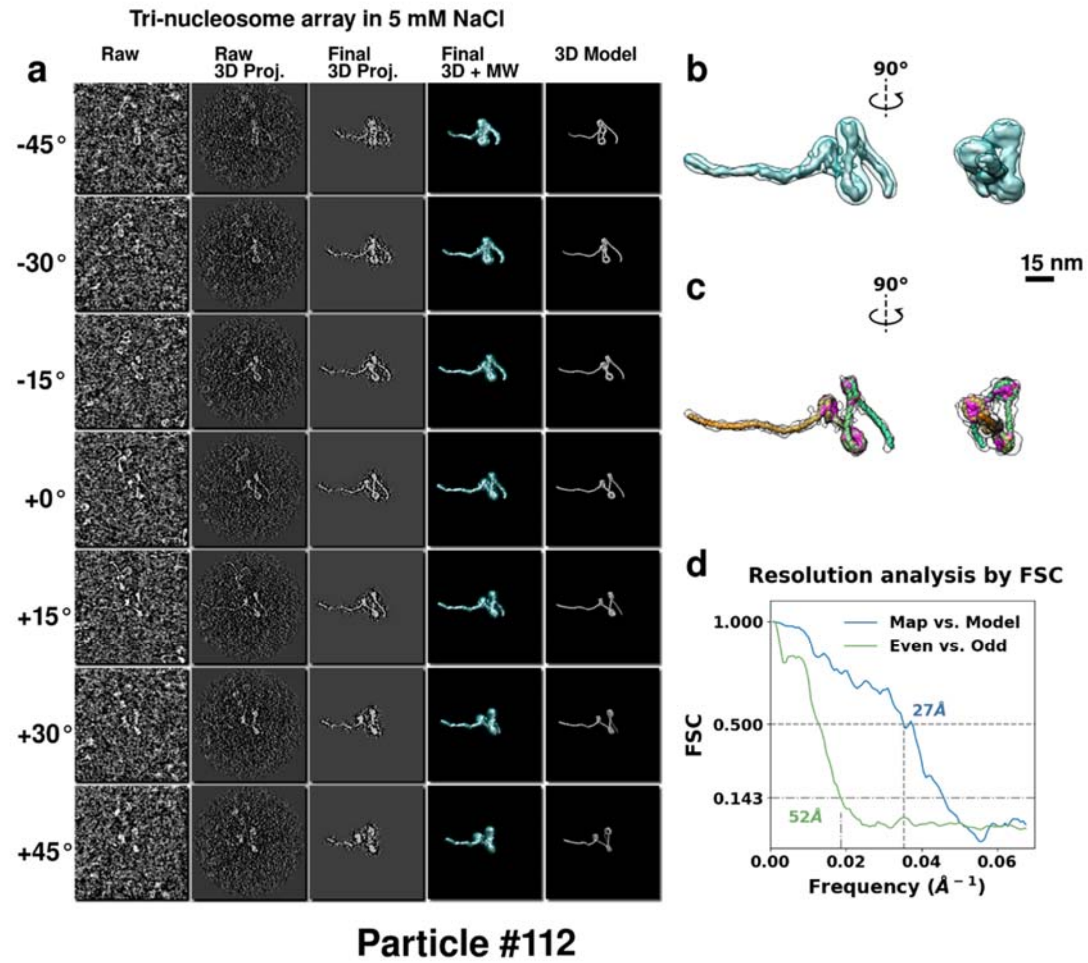

Supplementary Fig. 125. Cryo-ET 3D reconstruction of individual tri-nucleosome particle (index no. 112) in 5 mM NaCl. a, IPET 3D reconstruction of individual tri-nucleosome particles. The first column shows seven representative tilt images of an individual particle after CTF correction. Through alignment of the tilt images to a common center for 3D reconstruction via iterative refinement, the second and third columns display the 3D projections of the reconstruction before and after particle-shaped masking, respectively. The fourth column shows the final 3D reconstruction with missing wedge correction, and the fifth column presents the flexibly fitted model at the corresponding tilt angles. **b**, Zoomed-in view of the final 3D density map displayed in orthogonal views, shown at two contour levels. **c**, Superimposition of the high contour level map from (b) onto its flexibly fitted model. **d**, Resolution evaluation of the final 3D density map using two criteria: Fourier shell correlation (FSC) between two-half maps reconstructed from the even and odd index of the tilted series and FSC between the final 3D map and the fitted structure model. The resolution for the former and latter criteria is evaluated at frequencies of 0.5 and 0.143, respectively.

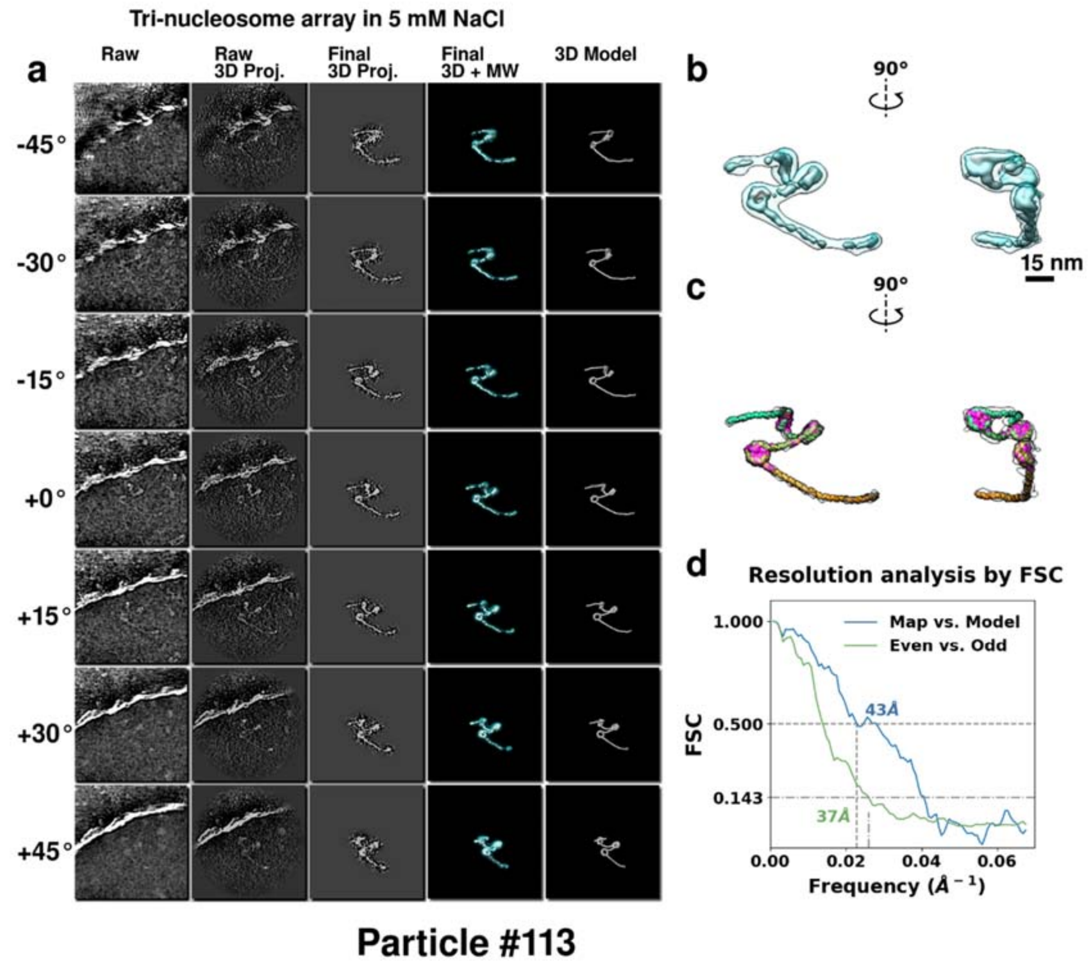

Supplementary Fig. 126. Cryo-ET 3D reconstruction of individual tri-nucleosome particle (index no. 113) in 5 mM NaCl. a, IPET 3D reconstruction of individual tri-nucleosome particles. The first column shows seven representative tilt images of an individual particle after CTF correction. Through alignment of the tilt images to a common center for 3D reconstruction via iterative refinement, the second and third columns display the 3D projections of the reconstruction before and after particle-shaped masking, respectively. The fourth column shows the final 3D reconstruction with missing wedge correction, and the fifth column presents the flexibly fitted model at the corresponding tilt angles. **b**, Zoomed-in view of the final 3D density map displayed in orthogonal views, shown at two contour levels. **c**, Superimposition of the high contour level map from (b) onto its flexibly fitted model. **d**, Resolution evaluation of the final 3D density map using two criteria: Fourier shell correlation (FSC) between two-half maps reconstructed from the even and odd index of the tilted series and FSC between the final 3D map and the fitted structure model. The resolution for the former and latter criteria is evaluated at frequencies of 0.5 and 0.143, respectively.

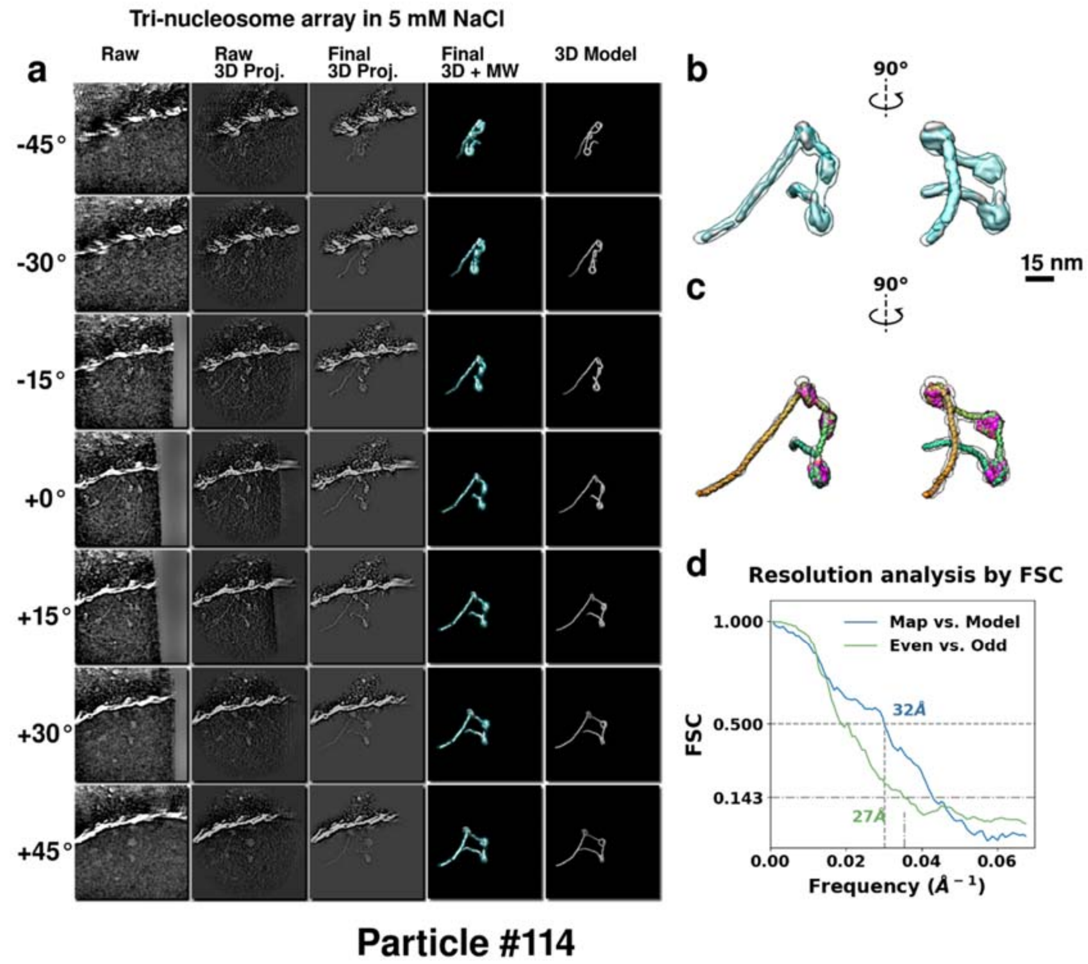

Supplementary Fig. 127. Cryo-ET 3D reconstruction of individual tri-nucleosome particle (index no. 114) in 5 mM NaCl. a, IPET 3D reconstruction of individual tri-nucleosome particles. The first column shows seven representative tilt images of an individual particle after CTF correction. Through alignment of the tilt images to a common center for 3D reconstruction via iterative refinement, the second and third columns display the 3D projections of the reconstruction before and after particle-shaped masking, respectively. The fourth column shows the final 3D reconstruction with missing wedge correction, and the fifth column presents the flexibly fitted model at the corresponding tilt angles. **b**, Zoomed-in view of the final 3D density map displayed in orthogonal views, shown at two contour levels. **c**, Superimposition of the high contour level map from (b) onto its flexibly fitted model. **d**, Resolution evaluation of the final 3D density map using two criteria: Fourier shell correlation (FSC) between two-half maps reconstructed from the even and odd index of the tilted series and FSC between the final 3D map and the fitted structure model. The resolution for the former and latter criteria is evaluated at frequencies of 0.5 and 0.143, respectively.

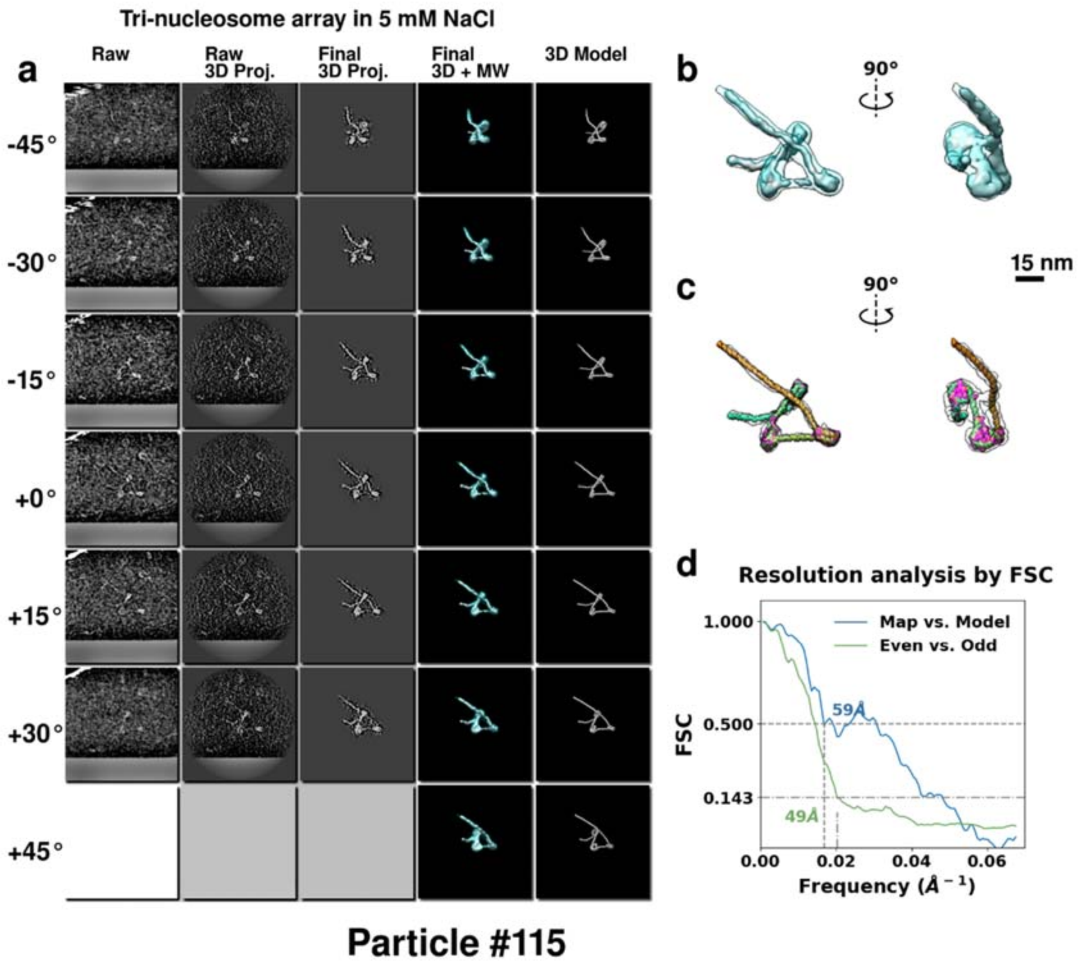

Supplementary Fig. 128. Cryo-ET 3D reconstruction of individual tri-nucleosome particle (index no. 115) in 5 mM NaCl. a, IPET 3D reconstruction of individual tri-nucleosome particles. The first column shows seven representative tilt images of an individual particle after CTF correction. Through alignment of the tilt images to a common center for 3D reconstruction via iterative refinement, the second and third columns display the 3D projections of the reconstruction before and after particle-shaped masking, respectively. The fourth column shows the final 3D reconstruction with missing wedge correction, and the fifth column presents the flexibly fitted model at the corresponding tilt angles. **b**, Zoomed-in view of the final 3D density map displayed in orthogonal views, shown at two contour levels. **c**, Superimposition of the high contour level map from (b) onto its flexibly fitted model. **d**, Resolution evaluation of the final 3D density map using two criteria: Fourier shell correlation (FSC) between two-half maps reconstructed from the even and odd index of the tilted series and FSC between the final 3D map and the fitted structure model. The resolution for the former and latter criteria is evaluated at frequencies of 0.5 and 0.143, respectively.

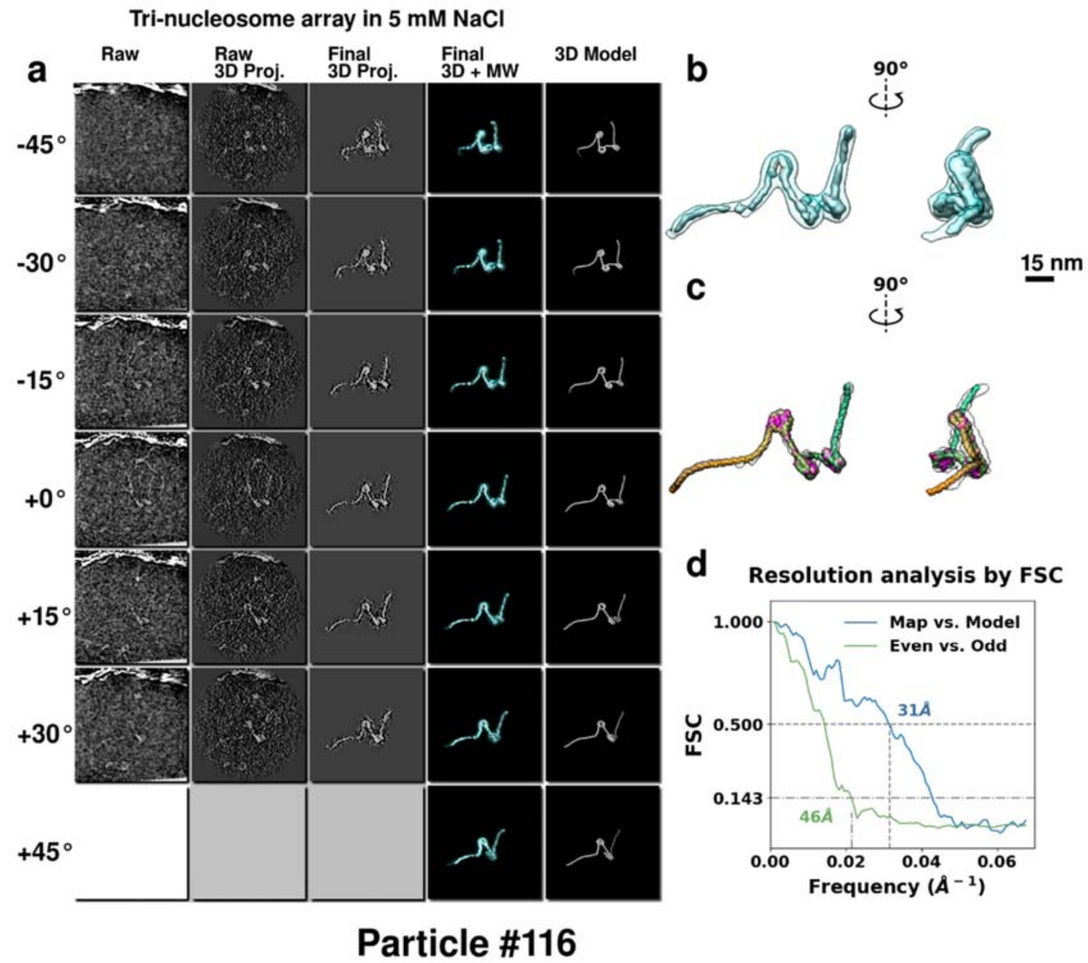

Supplementary Fig. 129. Cryo-ET 3D reconstruction of individual tri-nucleosome particle (index no. 116) in 5 mM NaCl. a, IPET 3D reconstruction of individual tri-nucleosome particles. The first column shows seven representative tilt images of an individual particle after CTF correction. Through alignment of the tilt images to a common center for 3D reconstruction via iterative refinement, the second and third columns display the 3D projections of the reconstruction before and after particle-shaped masking, respectively. The fourth column shows the final 3D reconstruction with missing wedge correction, and the fifth column presents the flexibly fitted model at the corresponding tilt angles. **b**, Zoomed-in view of the final 3D density map displayed in orthogonal views, shown at two contour levels. **c**, Superimposition of the high contour level map from (b) onto its flexibly fitted model. **d**, Resolution evaluation of the final 3D density map using two criteria: Fourier shell correlation (FSC) between two-half maps reconstructed from the even and odd index of the tilted series and FSC between the final 3D map and the fitted structure model. The resolution for the former and latter criteria is evaluated at frequencies of 0.5 and 0.143, respectively.

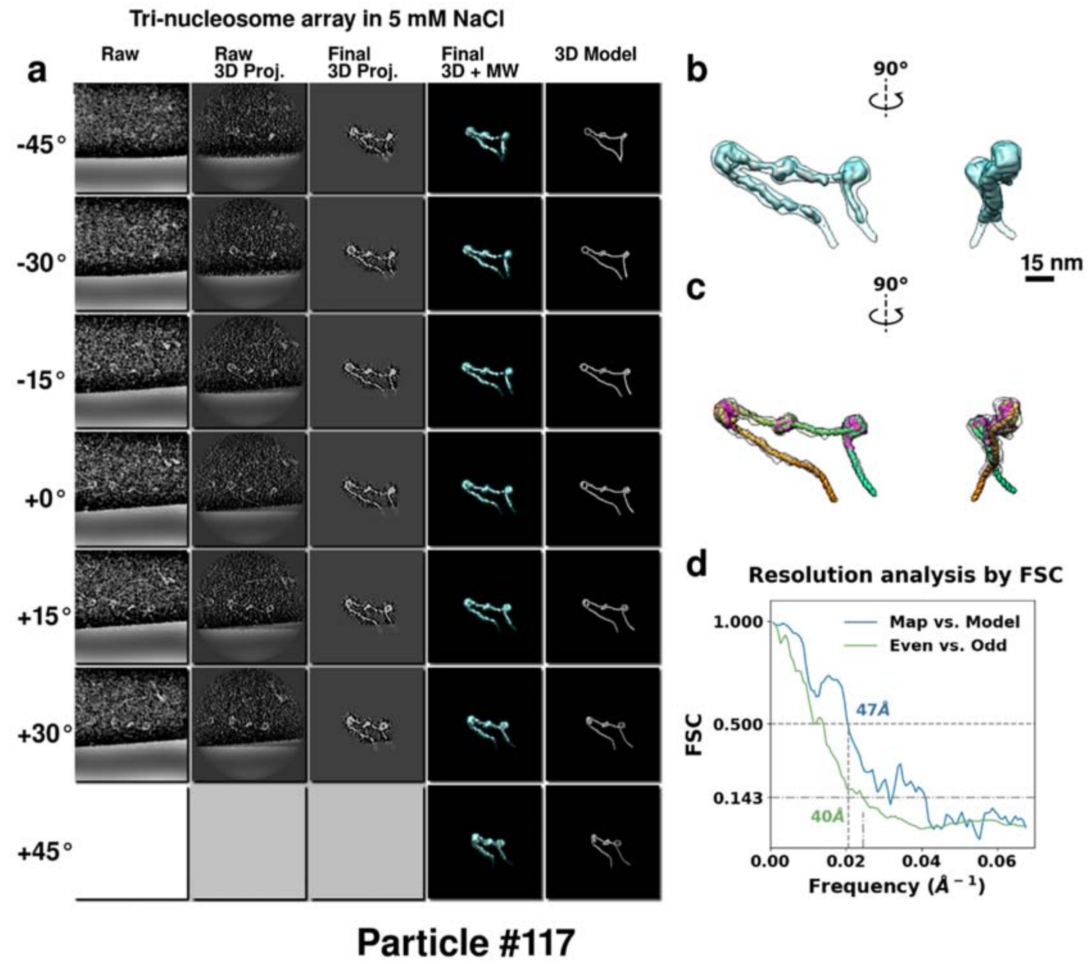

Supplementary Fig. 130. Cryo-ET 3D reconstruction of individual tri-nucleosome particle (index no. 117) in 5 mM NaCl. a, IPET 3D reconstruction of individual tri-nucleosome particles. The first column shows seven representative tilt images of an individual particle after CTF correction. Through alignment of the tilt images to a common center for 3D reconstruction via iterative refinement, the second and third columns display the 3D projections of the reconstruction before and after particle-shaped masking, respectively. The fourth column shows the final 3D reconstruction with missing wedge correction, and the fifth column presents the flexibly fitted model at the corresponding tilt angles. **b**, Zoomed-in view of the final 3D density map displayed in orthogonal views, shown at two contour levels. **c**, Superimposition of the high contour level map from (b) onto its flexibly fitted model. **d**, Resolution evaluation of the final 3D density map using two criteria: Fourier shell correlation (FSC) between two-half maps reconstructed from the even and odd index of the tilted series and FSC between the final 3D map and the fitted structure model. The resolution for the former and latter criteria is evaluated at frequencies of 0.5 and 0.143, respectively.

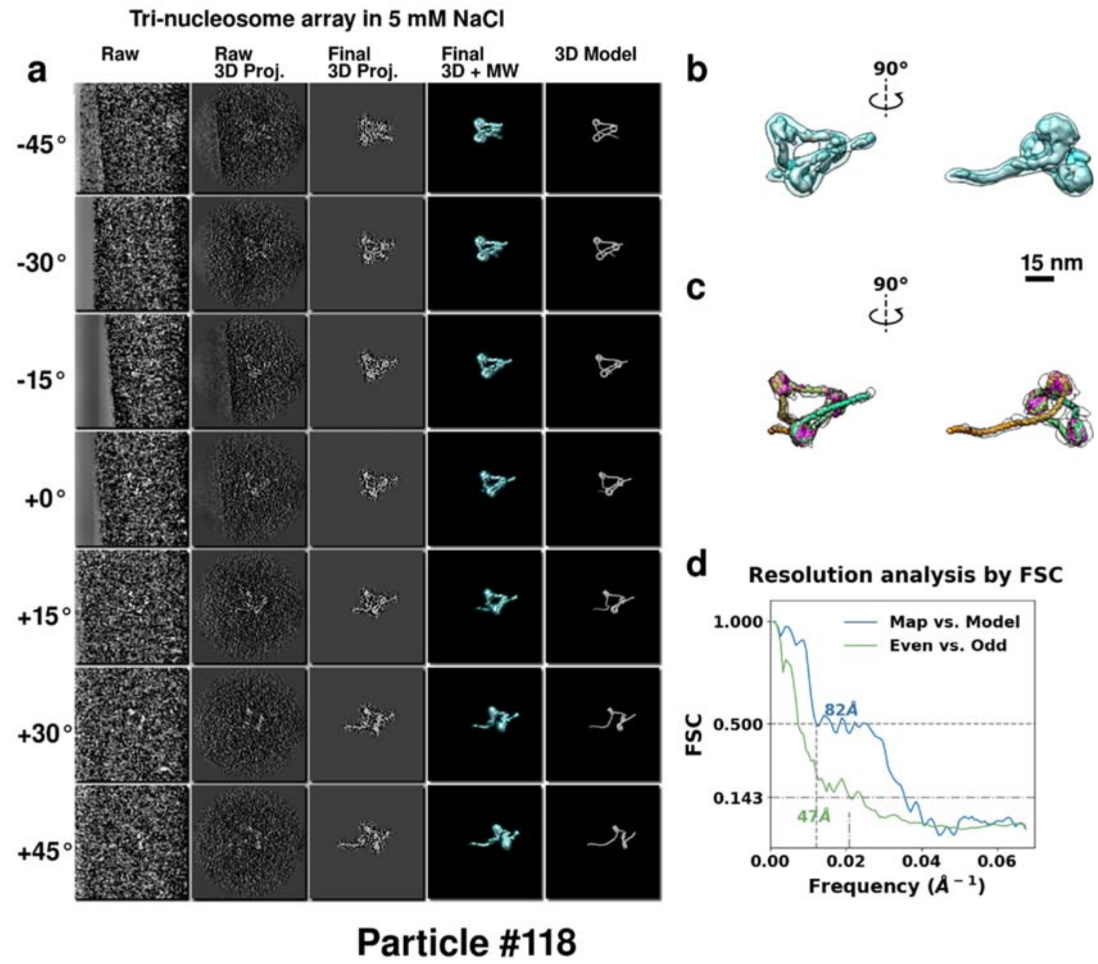

Supplementary Fig. 131. Cryo-ET 3D reconstruction of individual tri-nucleosome particle (index no. 118) in 5 mM NaCl. a, IPET 3D reconstruction of individual tri-nucleosome particles. The first column shows seven representative tilt images of an individual particle after CTF correction. Through alignment of the tilt images to a common center for 3D reconstruction via iterative refinement, the second and third columns display the 3D projections of the reconstruction before and after particle-shaped masking, respectively. The fourth column shows the final 3D reconstruction with missing wedge correction, and the fifth column presents the flexibly fitted model at the corresponding tilt angles. **b**, Zoomed-in view of the final 3D density map displayed in orthogonal views, shown at two contour levels. **c**, Superimposition of the high contour level map from (b) onto its flexibly fitted model. **d**, Resolution evaluation of the final 3D density map using two criteria: Fourier shell correlation (FSC) between two-half maps reconstructed from the even and odd index of the tilted series and FSC between the final 3D map and the fitted structure model. The resolution for the former and latter criteria is evaluated at frequencies of 0.5 and 0.143, respectively.

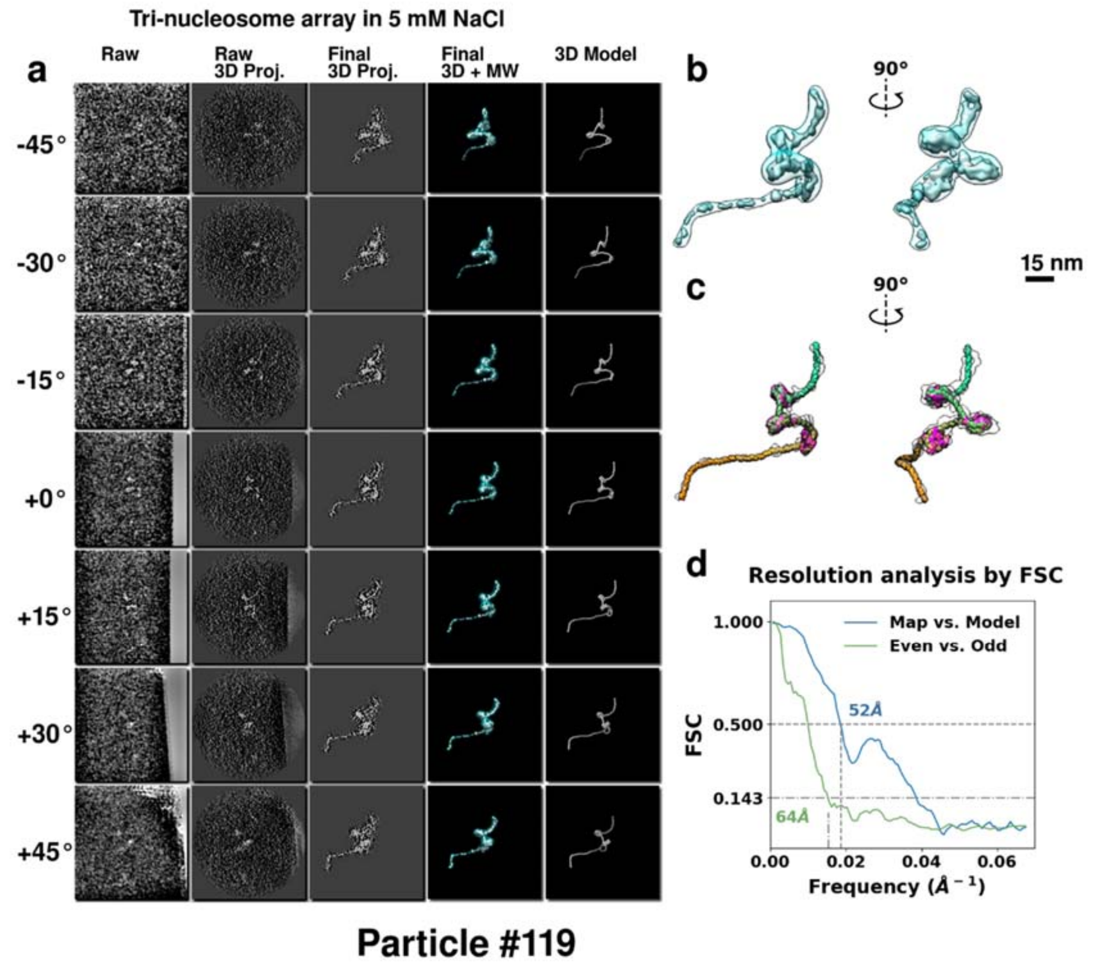

Supplementary Fig. 132. Cryo-ET 3D reconstruction of individual tri-nucleosome particle (index no. 119) in 5 mM NaCl. a, IPET 3D reconstruction of individual tri-nucleosome particles. The first column shows seven representative tilt images of an individual particle after CTF correction. Through alignment of the tilt images to a common center for 3D reconstruction via iterative refinement, the second and third columns display the 3D projections of the reconstruction before and after particle-shaped masking, respectively. The fourth column shows the final 3D reconstruction with missing wedge correction, and the fifth column presents the flexibly fitted model at the corresponding tilt angles. **b**, Zoomed-in view of the final 3D density map displayed in orthogonal views, shown at two contour levels. **c**, Superimposition of the high contour level map from (b) onto its flexibly fitted model. **d**, Resolution evaluation of the final 3D density map using two criteria: Fourier shell correlation (FSC) between two-half maps reconstructed from the even and odd index of the tilted series and FSC between the final 3D map and the fitted structure model. The resolution for the former and latter criteria is evaluated at frequencies of 0.5 and 0.143, respectively.

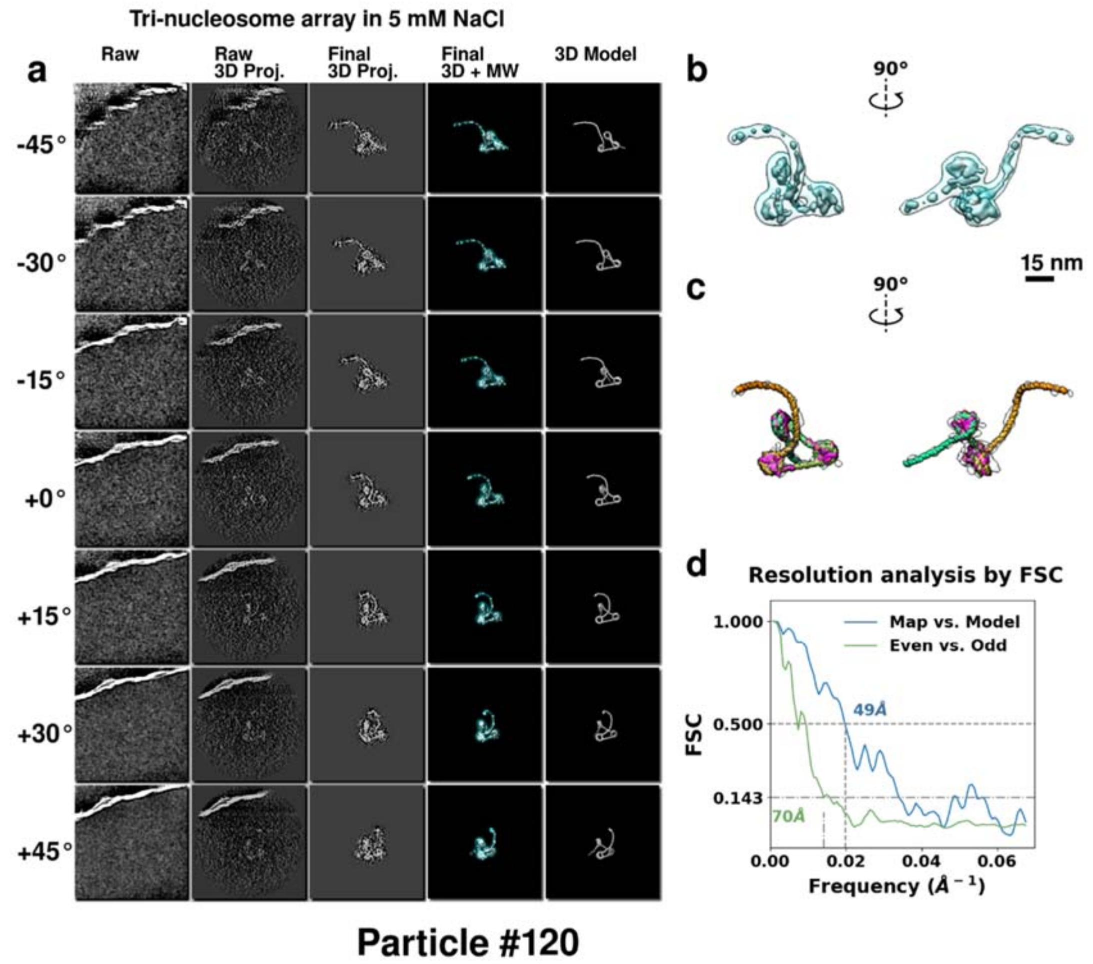

Supplementary Fig. 133. Cryo-ET 3D reconstruction of individual tri-nucleosome particle (index no. 120) in 5 mM NaCl. a, IPET 3D reconstruction of individual tri-nucleosome particles. The first column shows seven representative tilt images of an individual particle after CTF correction. Through alignment of the tilt images to a common center for 3D reconstruction via iterative refinement, the second and third columns display the 3D projections of the reconstruction before and after particle-shaped masking, respectively. The fourth column shows the final 3D reconstruction with missing wedge correction, and the fifth column presents the flexibly fitted model at the corresponding tilt angles. **b**, Zoomed-in view of the final 3D density map displayed in orthogonal views, shown at two contour levels. **c**, Superimposition of the high contour level map from (b) onto its flexibly fitted model. **d**, Resolution evaluation of the final 3D density map using two criteria: Fourier shell correlation (FSC) between two-half maps reconstructed from the even and odd index of the tilted series and FSC between the final 3D map and the fitted structure model. The resolution for the former and latter criteria is evaluated at frequencies of 0.5 and 0.143, respectively.

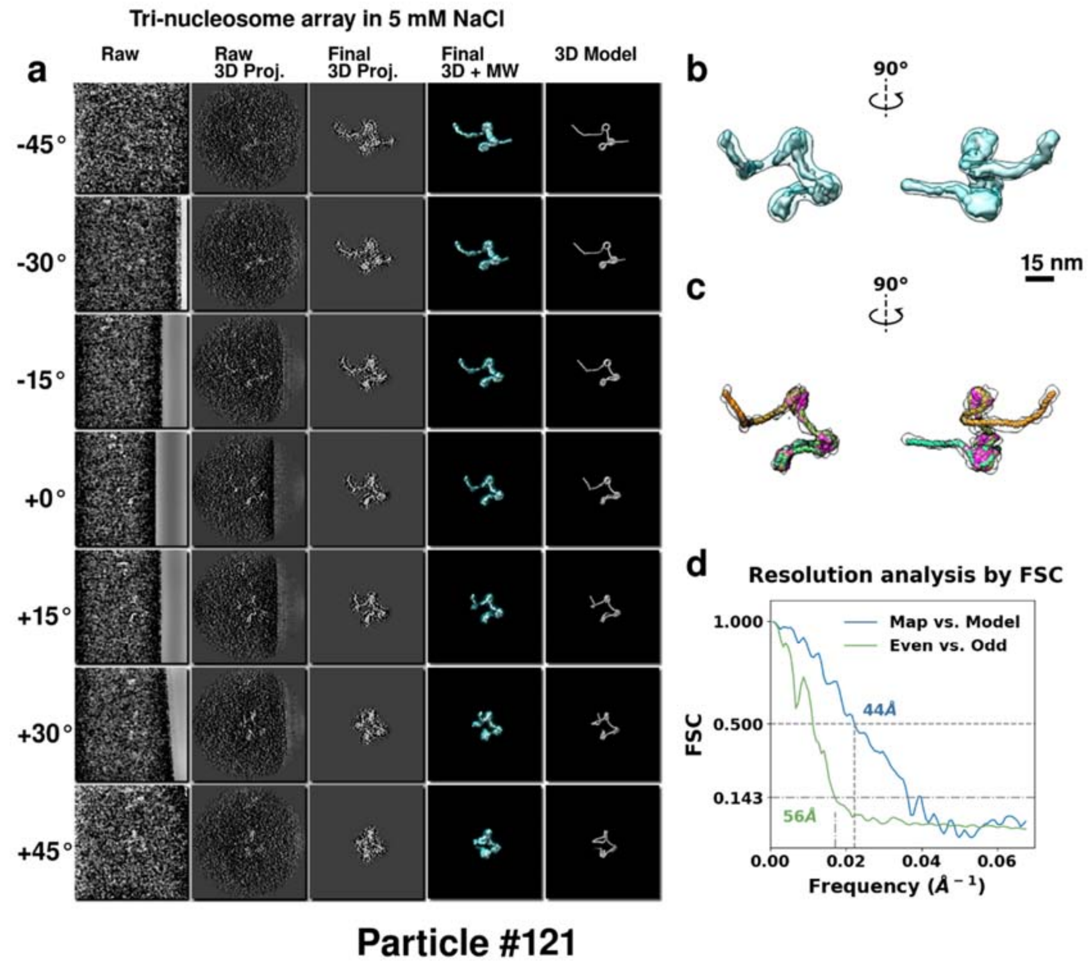

Supplementary Fig. 134. Cryo-ET 3D reconstruction of individual tri-nucleosome particle (index no. 121) in 5 mM NaCl. a, IPET 3D reconstruction of individual tri-nucleosome particles. The first column shows seven representative tilt images of an individual particle after CTF correction. Through alignment of the tilt images to a common center for 3D reconstruction via iterative refinement, the second and third columns display the 3D projections of the reconstruction before and after particle-shaped masking, respectively. The fourth column shows the final 3D reconstruction with missing wedge correction, and the fifth column presents the flexibly fitted model at the corresponding tilt angles. **b**, Zoomed-in view of the final 3D density map displayed in orthogonal views, shown at two contour levels. **c**, Superimposition of the high contour level map from (b) onto its flexibly fitted model. **d**, Resolution evaluation of the final 3D density map using two criteria: Fourier shell correlation (FSC) between two-half maps reconstructed from the even and odd index of the tilted series and FSC between the final 3D map and the fitted structure model. The resolution for the former and latter criteria is evaluated at frequencies of 0.5 and 0.143, respectively.

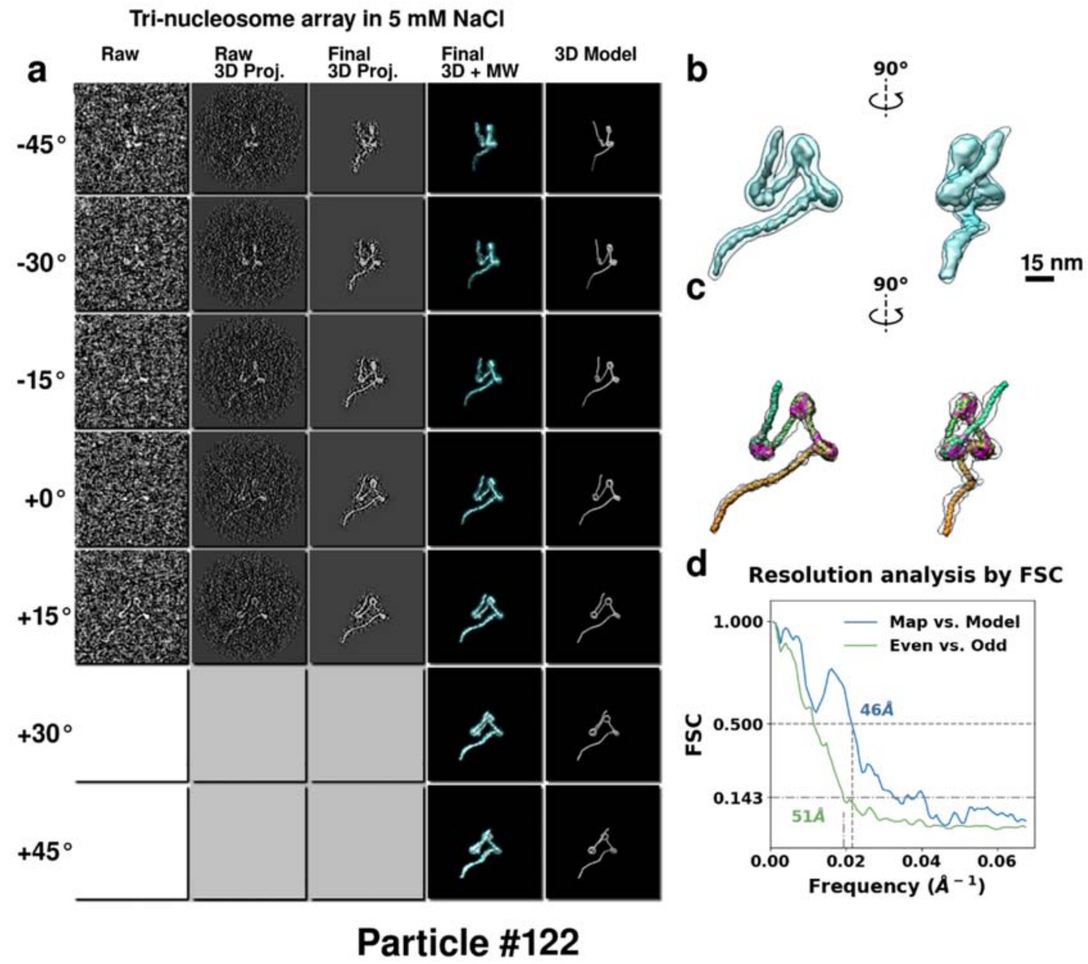

Supplementary Fig. 135. Cryo-ET 3D reconstruction of individual tri-nucleosome particle (index no. 122) in 5 mM NaCl. a, IPET 3D reconstruction of individual tri-nucleosome particles. The first column shows seven representative tilt images of an individual particle after CTF correction. Through alignment of the tilt images to a common center for 3D reconstruction via iterative refinement, the second and third columns display the 3D projections of the reconstruction before and after particle-shaped masking, respectively. The fourth column shows the final 3D reconstruction with missing wedge correction, and the fifth column presents the flexibly fitted model at the corresponding tilt angles. **b**, Zoomed-in view of the final 3D density map displayed in orthogonal views, shown at two contour levels. **c**, Superimposition of the high contour level map from (b) onto its flexibly fitted model. **d**, Resolution evaluation of the final 3D density map using two criteria: Fourier shell correlation (FSC) between two-half maps reconstructed from the even and odd index of the tilted series and FSC between the final 3D map and the fitted structure model. The resolution for the former and latter criteria is evaluated at frequencies of 0.5 and 0.143, respectively.

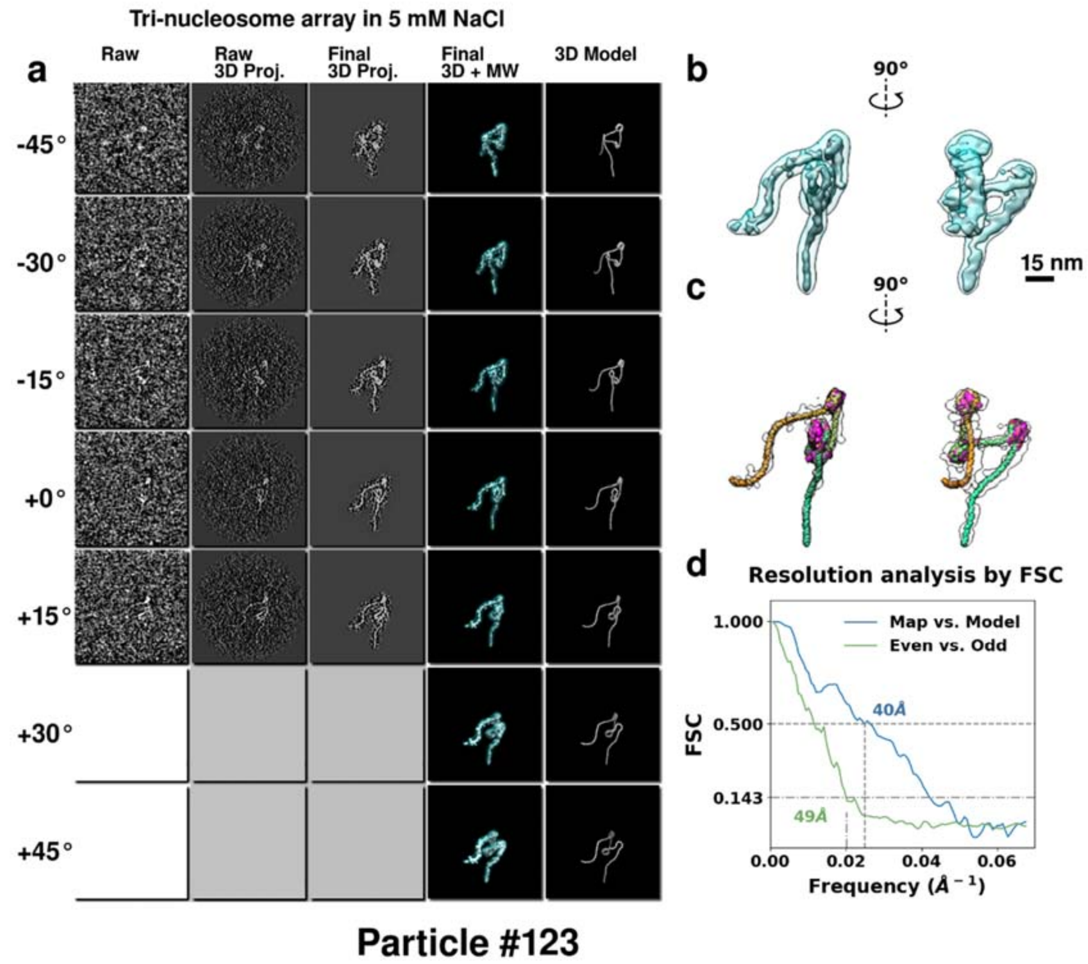

Supplementary Fig. 136. Cryo-ET 3D reconstruction of individual tri-nucleosome particle (index no. 123) in 5 mM NaCl. a, IPET 3D reconstruction of individual tri-nucleosome particles. The first column shows seven representative tilt images of an individual particle after CTF correction. Through alignment of the tilt images to a common center for 3D reconstruction via iterative refinement, the second and third columns display the 3D projections of the reconstruction before and after particle-shaped masking, respectively. The fourth column shows the final 3D reconstruction with missing wedge correction, and the fifth column presents the flexibly fitted model at the corresponding tilt angles. **b**, Zoomed-in view of the final 3D density map displayed in orthogonal views, shown at two contour levels. **c**, Superimposition of the high contour level map from (b) onto its flexibly fitted model. **d**, Resolution evaluation of the final 3D density map using two criteria: Fourier shell correlation (FSC) between two-half maps reconstructed from the even and odd index of the tilted series and FSC between the final 3D map and the fitted structure model. The resolution for the former and latter criteria is evaluated at frequencies of 0.5 and 0.143, respectively.

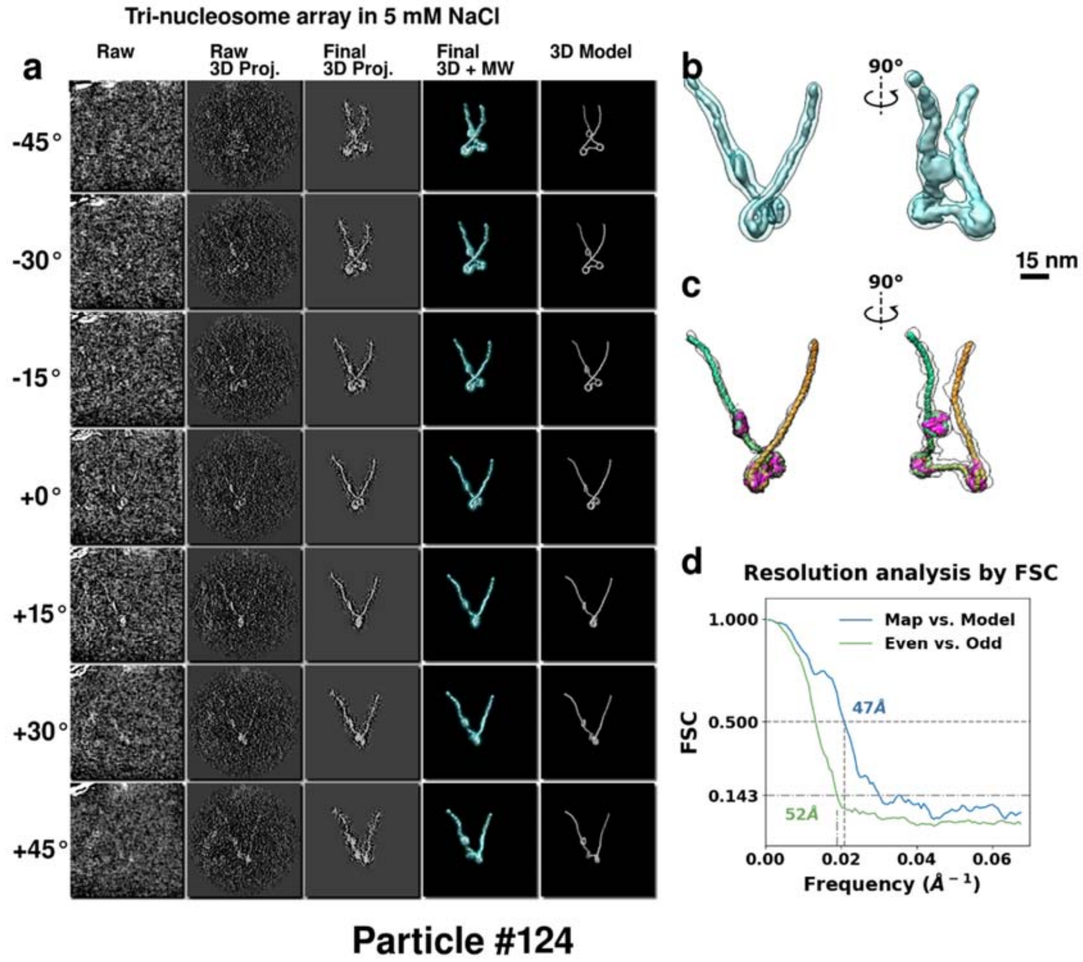

Supplementary Fig. 137. Cryo-ET 3D reconstruction of individual tri-nucleosome particle (index no. 124) in 5 mM NaCl. a, IPET 3D reconstruction of individual tri-nucleosome particles. The first column shows seven representative tilt images of an individual particle after CTF correction. Through alignment of the tilt images to a common center for 3D reconstruction via iterative refinement, the second and third columns display the 3D projections of the reconstruction before and after particle-shaped masking, respectively. The fourth column shows the final 3D reconstruction with missing wedge correction, and the fifth column presents the flexibly fitted model at the corresponding tilt angles. **b**, Zoomed-in view of the final 3D density map displayed in orthogonal views, shown at two contour levels. **c**, Superimposition of the high contour level map from (b) onto its flexibly fitted model. **d**, Resolution evaluation of the final 3D density map using two criteria: Fourier shell correlation (FSC) between two-half maps reconstructed from the even and odd index of the tilted series and FSC between the final 3D map and the fitted structure model. The resolution for the former and latter criteria is evaluated at frequencies of 0.5 and 0.143, respectively.

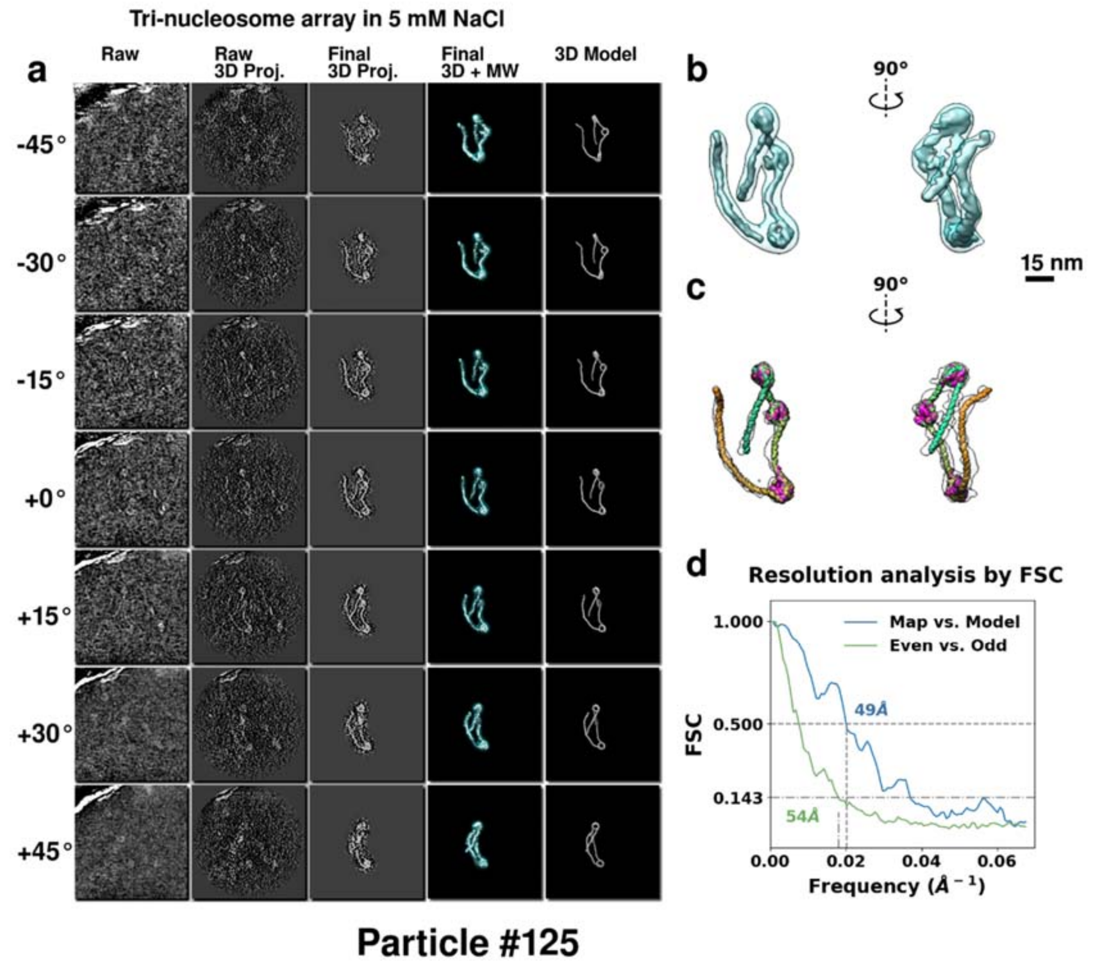

Supplementary Fig. 138. Cryo-ET 3D reconstruction of individual tri-nucleosome particle (index no. 125) in 5 mM NaCl. a, IPET 3D reconstruction of individual tri-nucleosome particles. The first column shows seven representative tilt images of an individual particle after CTF correction. Through alignment of the tilt images to a common center for 3D reconstruction via iterative refinement, the second and third columns display the 3D projections of the reconstruction before and after particle-shaped masking, respectively. The fourth column shows the final 3D reconstruction with missing wedge correction, and the fifth column presents the flexibly fitted model at the corresponding tilt angles. **b**, Zoomed-in view of the final 3D density map displayed in orthogonal views, shown at two contour levels. **c**, Superimposition of the high contour level map from (b) onto its flexibly fitted model. **d**, Resolution evaluation of the final 3D density map using two criteria: Fourier shell correlation (FSC) between two-half maps reconstructed from the even and odd index of the tilted series and FSC between the final 3D map and the fitted structure model. The resolution for the former and latter criteria is evaluated at frequencies of 0.5 and 0.143, respectively.

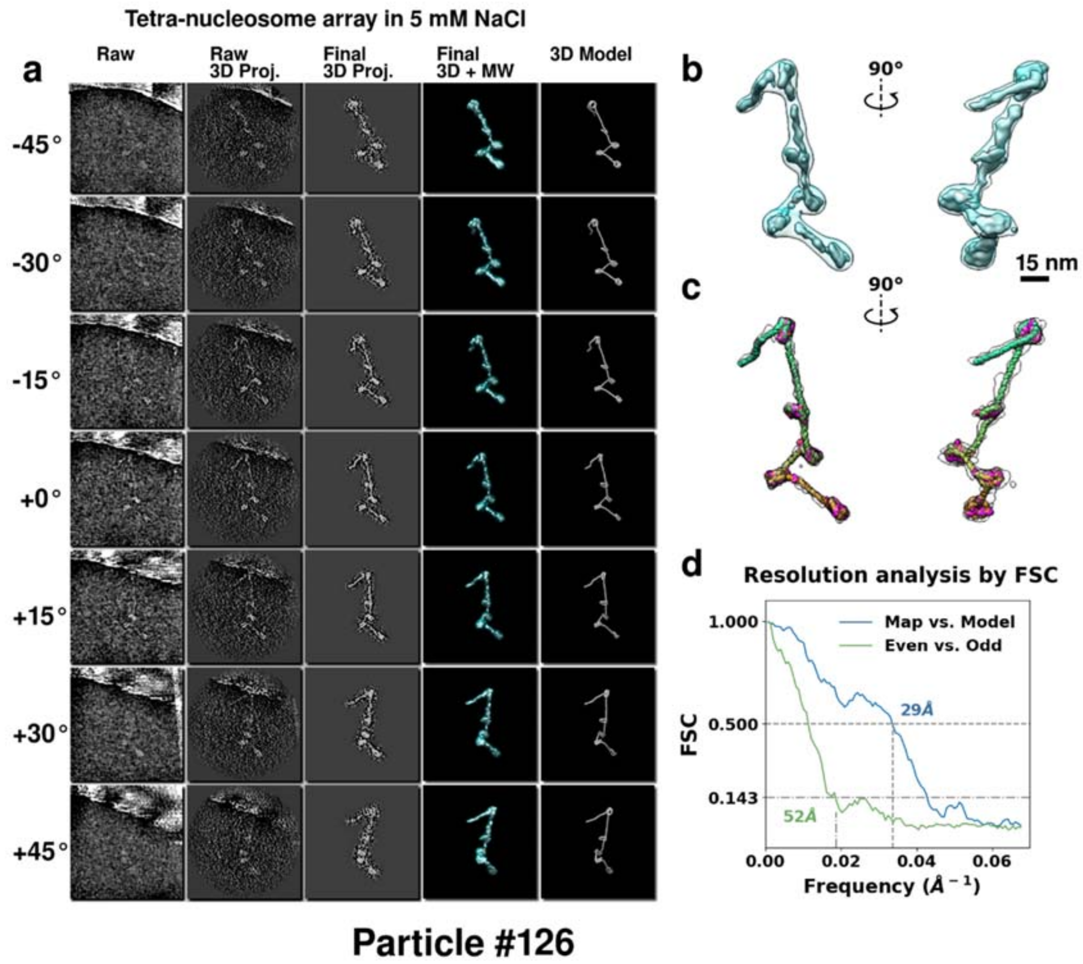

Supplementary Fig. 139. Cryo-ET 3D reconstruction of individual tetra-nucleosome particle (index no. 126) in 5 mM NaCl. a, IPET 3D reconstruction of individual tetra-nucleosome particles. The first column shows seven representative tilt images of an individual particle after CTF correction. Through alignment of the tilt images to a common center for 3D reconstruction via iterative refinement, the second and third columns display the 3D projections of the reconstruction before and after particle-shaped masking, respectively. The fourth column shows the final 3D reconstruction with missing wedge correction, and the fifth column presents the flexibly fitted model at the corresponding tilt angles. **b**, Zoomed-in view of the final 3D density map displayed in orthogonal views, shown at two contour levels. **c**, Superimposition of the high contour level map from (b) onto its flexibly fitted model. **d**, Resolution evaluation of the final 3D density map using two criteria: Fourier shell correlation (FSC) between two-half maps reconstructed from the even and odd index of the tilted series and FSC between the final 3D map and the fitted structure model. The resolution for the former and latter criteria is evaluated at frequencies of 0.5 and 0.143, respectively.

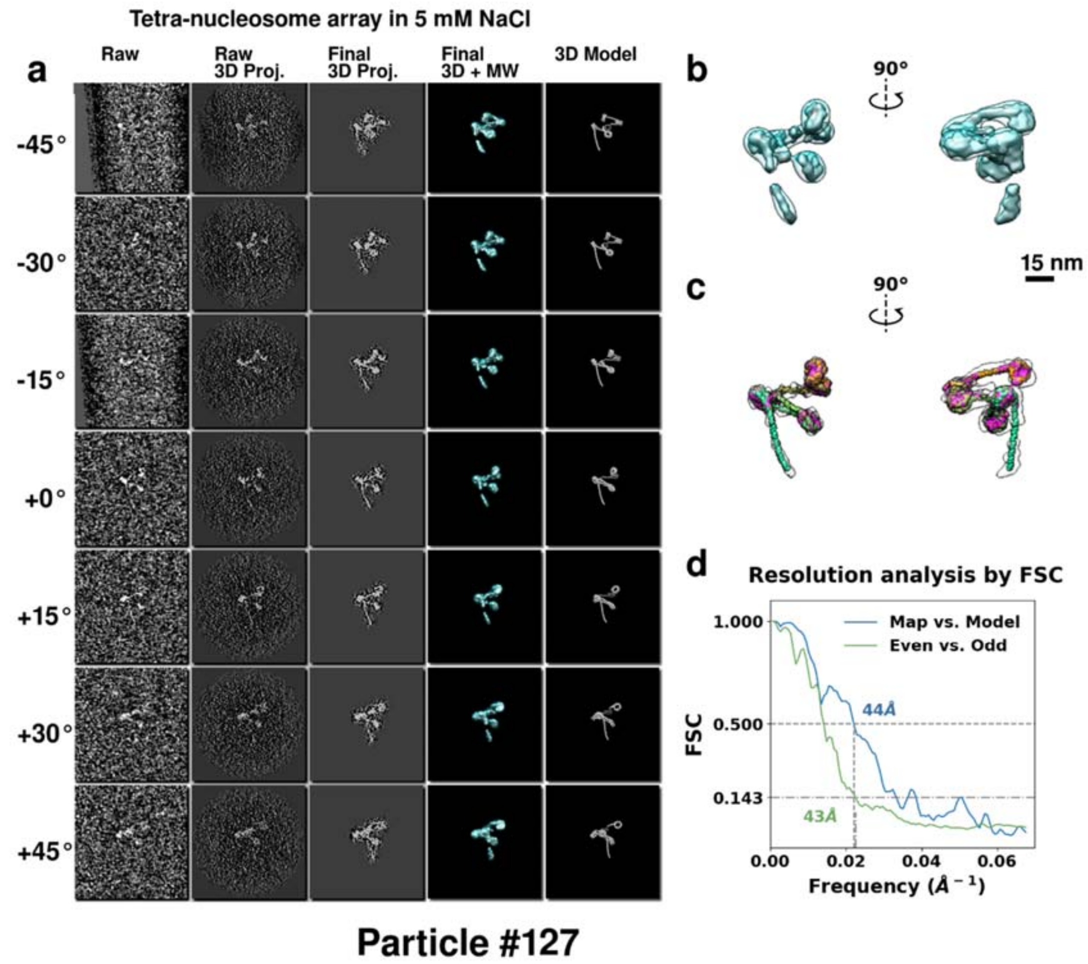

Supplementary Fig. 140. Cryo-ET 3D reconstruction of individual tetra-nucleosome particle (index no. 127) in 5 mM NaCl. **a**, IPET 3D reconstruction of individual tetra-nucleosome particles. The first column shows seven representative tilt images of an individual particle after CTF correction. Through alignment of the tilt images to a common center for 3D reconstruction via iterative refinement, the second and third columns display the 3D projections of the reconstruction before and after particle-shaped masking, respectively. The fourth column shows the final 3D reconstruction with missing wedge correction, and the fifth column presents the flexibly fitted model at the corresponding tilt angles. **b**, Zoomed-in view of the final 3D density map displayed in orthogonal views, shown at two contour levels. **c**, Superimposition of the high contour level map from (b) onto its flexibly fitted model. **d**, Resolution evaluation of the final 3D density map using two criteria: Fourier shell correlation (FSC) between two-half maps reconstructed from the even and odd index of the tilted series and FSC between the final 3D map and the fitted structure model. The resolution for the former and latter criteria is evaluated at frequencies of 0.5 and 0.143, respectively.

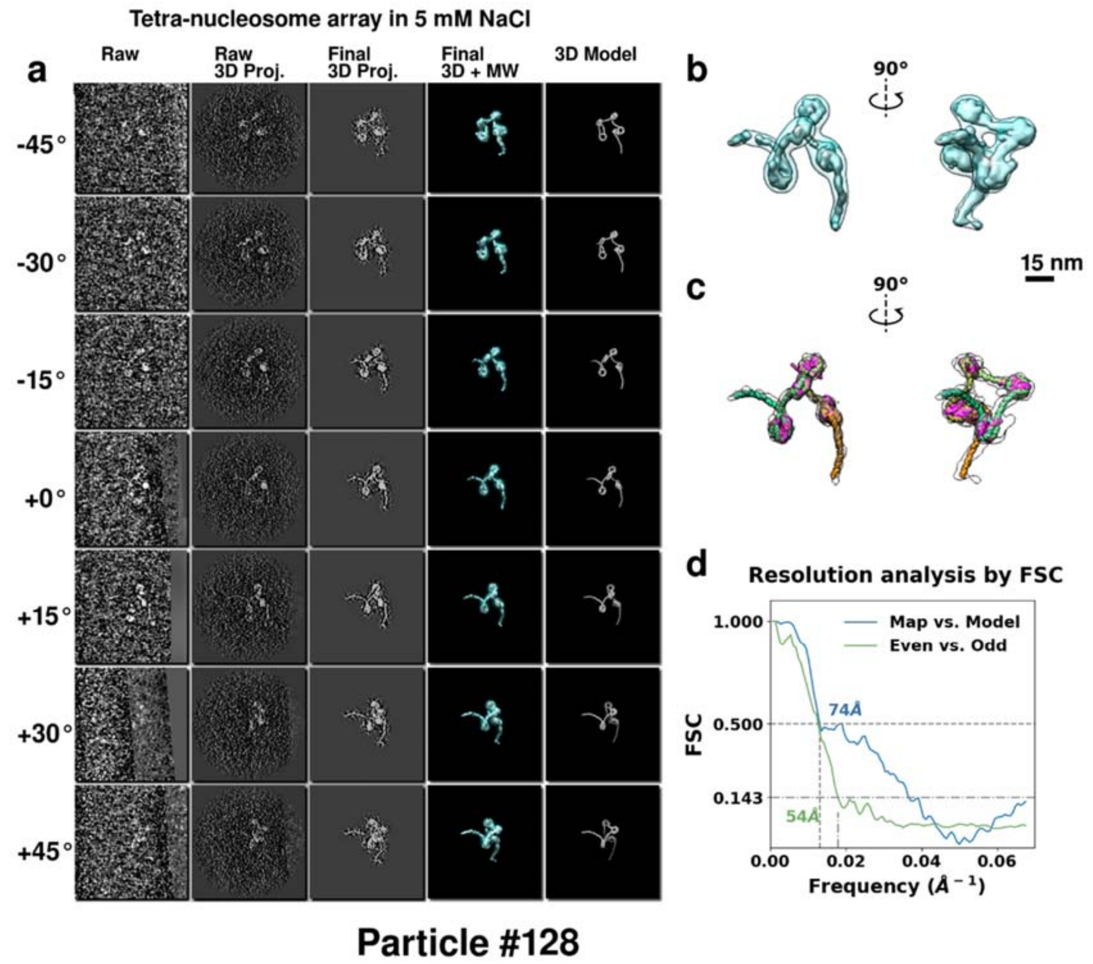

Supplementary Fig. 141. Cryo-ET 3D reconstruction of individual tetra-nucleosome particle (index no. 128) in 5 mM NaCl. a, IPET 3D reconstruction of individual tetra-nucleosome particles. The first column shows seven representative tilt images of an individual particle after CTF correction. Through alignment of the tilt images to a common center for 3D reconstruction via iterative refinement, the second and third columns display the 3D projections of the reconstruction before and after particle-shaped masking, respectively. The fourth column shows the final 3D reconstruction with missing wedge correction, and the fifth column presents the flexibly fitted model at the corresponding tilt angles. **b**, Zoomed-in view of the final 3D density map displayed in orthogonal views, shown at two contour levels. **c**, Superimposition of the high contour level map from (b) onto its flexibly fitted model. **d**, Resolution evaluation of the final 3D density map using two criteria: Fourier shell correlation (FSC) between two-half maps reconstructed from the even and odd index of the tilted series and FSC between the final 3D map and the fitted structure model. The resolution for the former and latter criteria is evaluated at frequencies of 0.5 and 0.143, respectively.

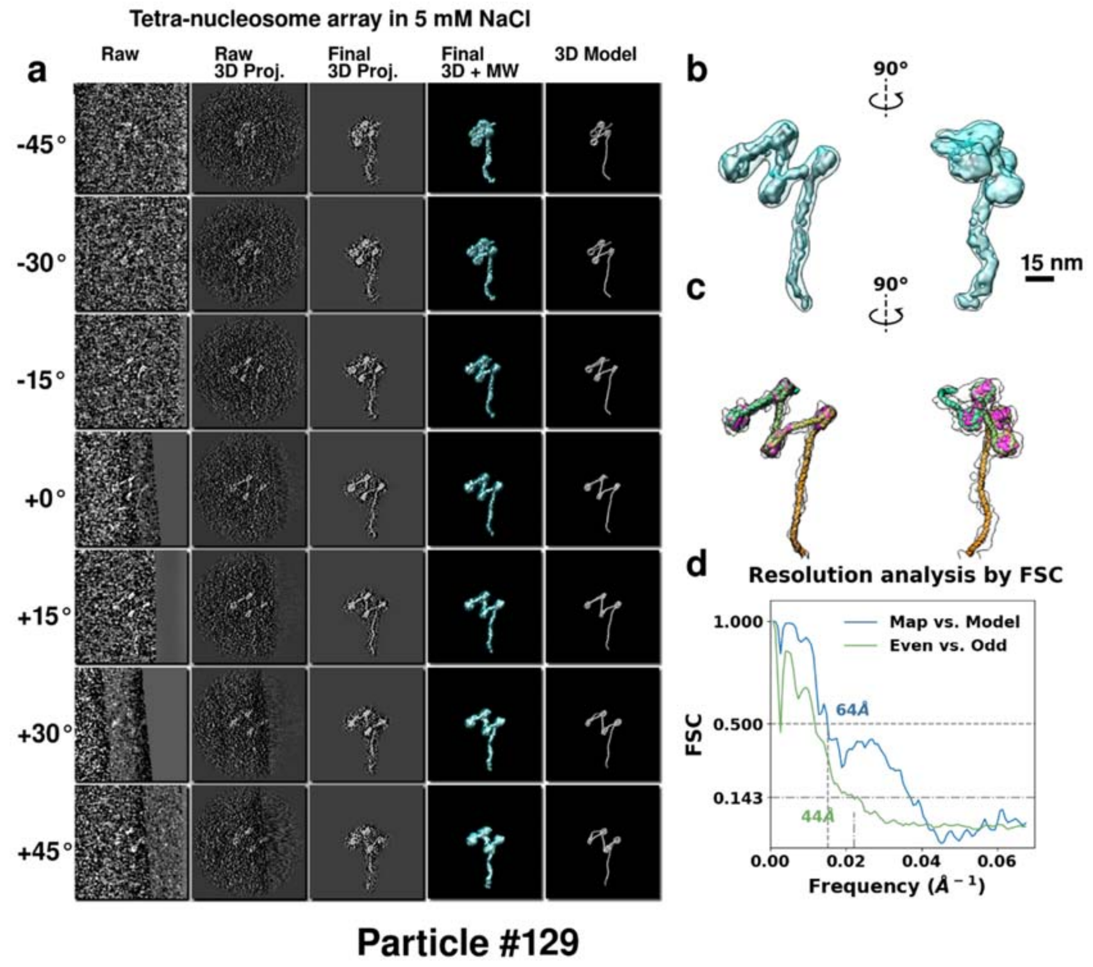

Supplementary Fig. 142. Cryo-ET 3D reconstruction of individual tetra-nucleosome particle (index no. 129) in 5 mM NaCl. a, IPET 3D reconstruction of individual tetra-nucleosome particles. The first column shows seven representative tilt images of an individual particle after CTF correction. Through alignment of the tilt images to a common center for 3D reconstruction via iterative refinement, the second and third columns display the 3D projections of the reconstruction before and after particle-shaped masking, respectively. The fourth column shows the final 3D reconstruction with missing wedge correction, and the fifth column presents the flexibly fitted model at the corresponding tilt angles. **b**, Zoomed-in view of the final 3D density map displayed in orthogonal views, shown at two contour levels. **c**, Superimposition of the high contour level map from (b) onto its flexibly fitted model. **d**, Resolution evaluation of the final 3D density map using two criteria: Fourier shell correlation (FSC) between two-half maps reconstructed from the even and odd index of the tilted series and FSC between the final 3D map and the fitted structure model. The resolution for the former and latter criteria is evaluated at frequencies of 0.5 and 0.143, respectively.

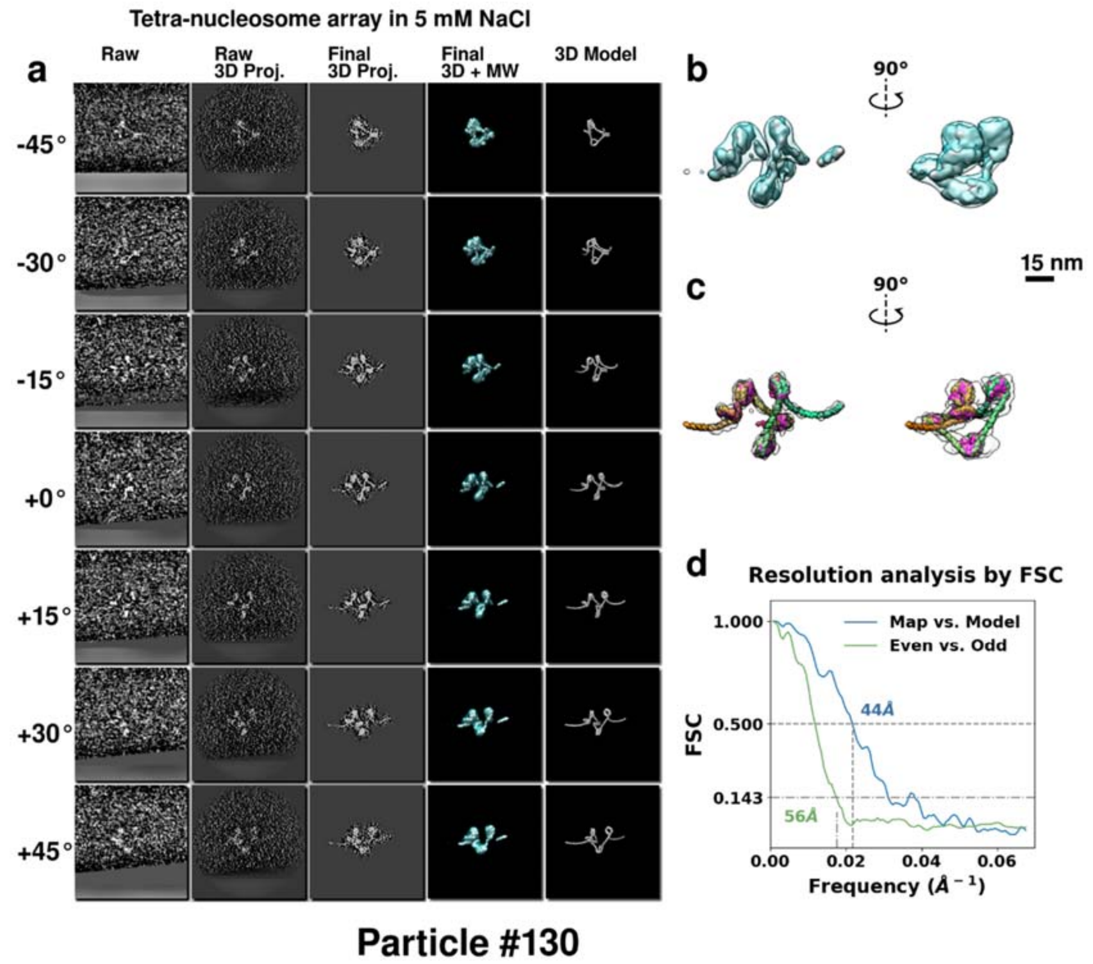

Supplementary Fig. 143. Cryo-ET 3D reconstruction of individual tetra-nucleosome particle (index no. 130) in 5 mM NaCl. a, IPET 3D reconstruction of individual tetra-nucleosome particles. The first column shows seven representative tilt images of an individual particle after CTF correction. Through alignment of the tilt images to a common center for 3D reconstruction via iterative refinement, the second and third columns display the 3D projections of the reconstruction before and after particle-shaped masking, respectively. The fourth column shows the final 3D reconstruction with missing wedge correction, and the fifth column presents the flexibly fitted model at the corresponding tilt angles. **b**, Zoomed-in view of the final 3D density map displayed in orthogonal views, shown at two contour levels. **c**, Superimposition of the high contour level map from (b) onto its flexibly fitted model. **d**, Resolution evaluation of the final 3D density map using two criteria: Fourier shell correlation (FSC) between two-half maps reconstructed from the even and odd index of the tilted series and FSC between the final 3D map and the fitted structure model. The resolution for the former and latter criteria is evaluated at frequencies of 0.5 and 0.143, respectively.

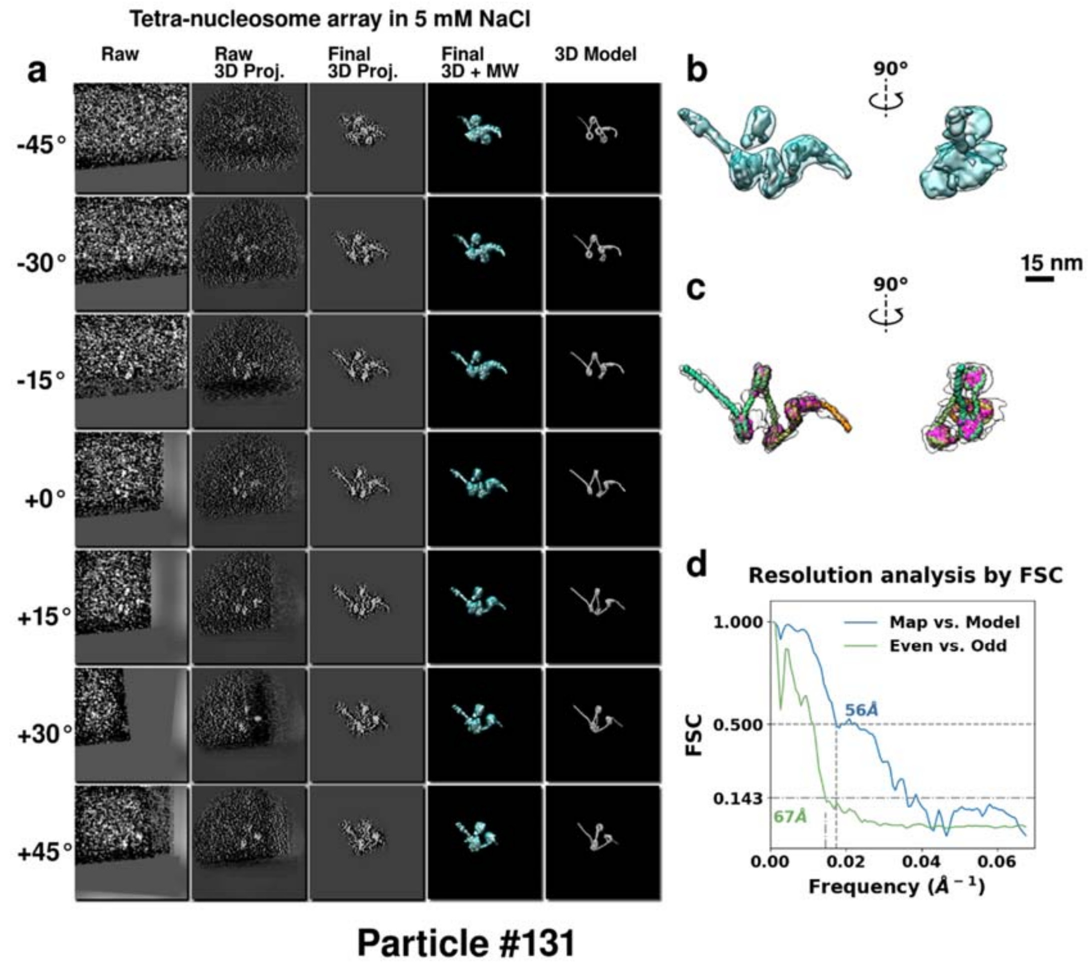

Supplementary Fig. 144. Cryo-ET 3D reconstruction of individual tetra-nucleosome particle (index no. 131) in 5 mM NaCl. a, IPET 3D reconstruction of individual tetra-nucleosome particles. The first column shows seven representative tilt images of an individual particle after CTF correction. Through alignment of the tilt images to a common center for 3D reconstruction via iterative refinement, the second and third columns display the 3D projections of the reconstruction before and after particle-shaped masking, respectively. The fourth column shows the final 3D reconstruction with missing wedge correction, and the fifth column presents the flexibly fitted model at the corresponding tilt angles. **b**, Zoomed-in view of the final 3D density map displayed in orthogonal views, shown at two contour levels. **c**, Superimposition of the high contour level map from (b) onto its flexibly fitted model. **d**, Resolution evaluation of the final 3D density map using two criteria: Fourier shell correlation (FSC) between two-half maps reconstructed from the even and odd index of the tilted series and FSC between the final 3D map and the fitted structure model. The resolution for the former and latter criteria is evaluated at frequencies of 0.5 and 0.143, respectively.

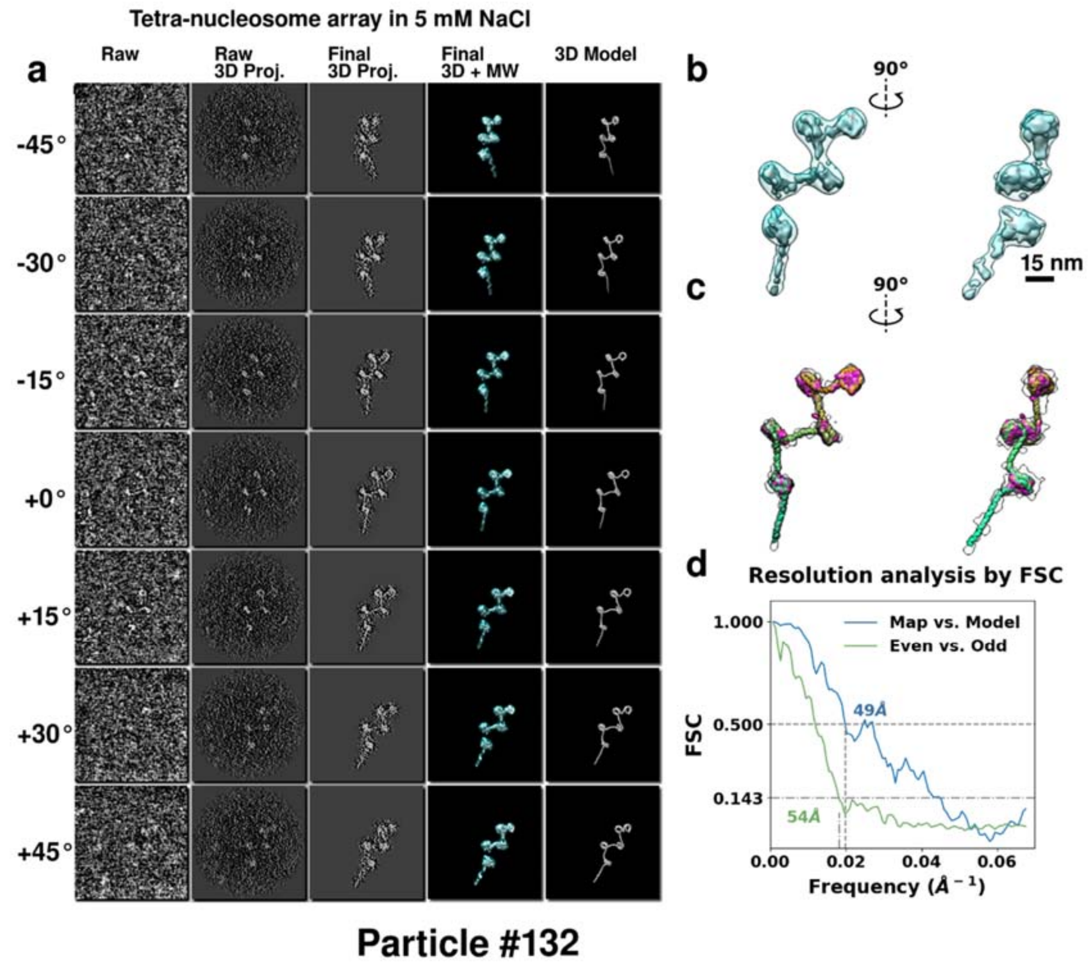

Supplementary Fig. 145. Cryo-ET 3D reconstruction of individual tetra-nucleosome particle (index no. 132) in 5 mM NaCl. a, IPET 3D reconstruction of individual tetra-nucleosome particles. The first column shows seven representative tilt images of an individual particle after CTF correction. Through alignment of the tilt images to a common center for 3D reconstruction via iterative refinement, the second and third columns display the 3D projections of the reconstruction before and after particle-shaped masking, respectively. The fourth column shows the final 3D reconstruction with missing wedge correction, and the fifth column presents the flexibly fitted model at the corresponding tilt angles. **b**, Zoomed-in view of the final 3D density map displayed in orthogonal views, shown at two contour levels. **c**, Superimposition of the high contour level map from (b) onto its flexibly fitted model. **d**, Resolution evaluation of the final 3D density map using two criteria: Fourier shell correlation (FSC) between two-half maps reconstructed from the even and odd index of the tilted series and FSC between the final 3D map and the fitted structure model. The resolution for the former and latter criteria is evaluated at frequencies of 0.5 and 0.143, respectively.

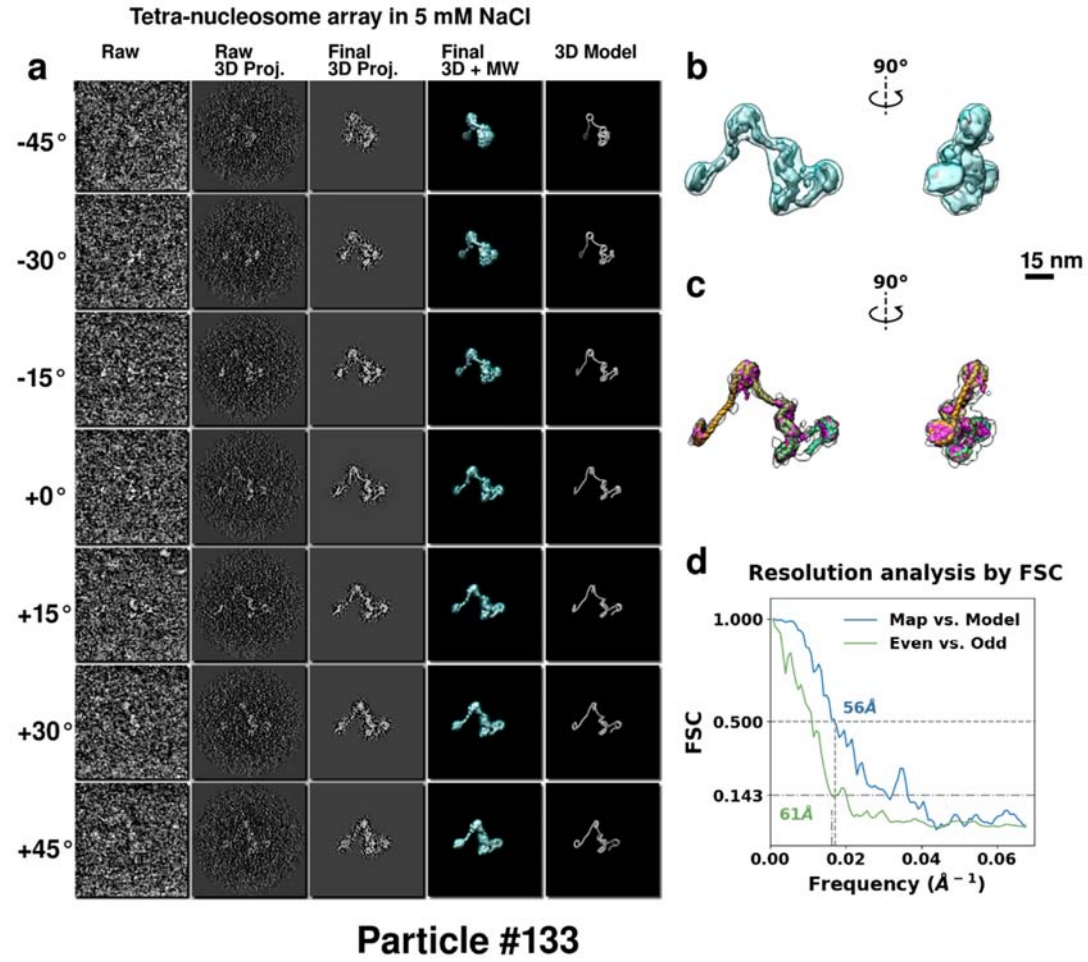

Supplementary Fig. 146. Cryo-ET 3D reconstruction of individual tetra-nucleosome particle (index no. 133) in 5 mM NaCl. a, IPET 3D reconstruction of individual tetra-nucleosome particles. The first column shows seven representative tilt images of an individual particle after CTF correction. Through alignment of the tilt images to a common center for 3D reconstruction via iterative refinement, the second and third columns display the 3D projections of the reconstruction before and after particle-shaped masking, respectively. The fourth column shows the final 3D reconstruction with missing wedge correction, and the fifth column presents the flexibly fitted model at the corresponding tilt angles. **b**, Zoomed-in view of the final 3D density map displayed in orthogonal views, shown at two contour levels. **c**, Superimposition of the high contour level map from (b) onto its flexibly fitted model. **d**, Resolution evaluation of the final 3D density map using two criteria: Fourier shell correlation (FSC) between two-half maps reconstructed from the even and odd index of the tilted series and FSC between the final 3D map and the fitted structure model. The resolution for the former and latter criteria is evaluated at frequencies of 0.5 and 0.143, respectively.

Supplementary Fig

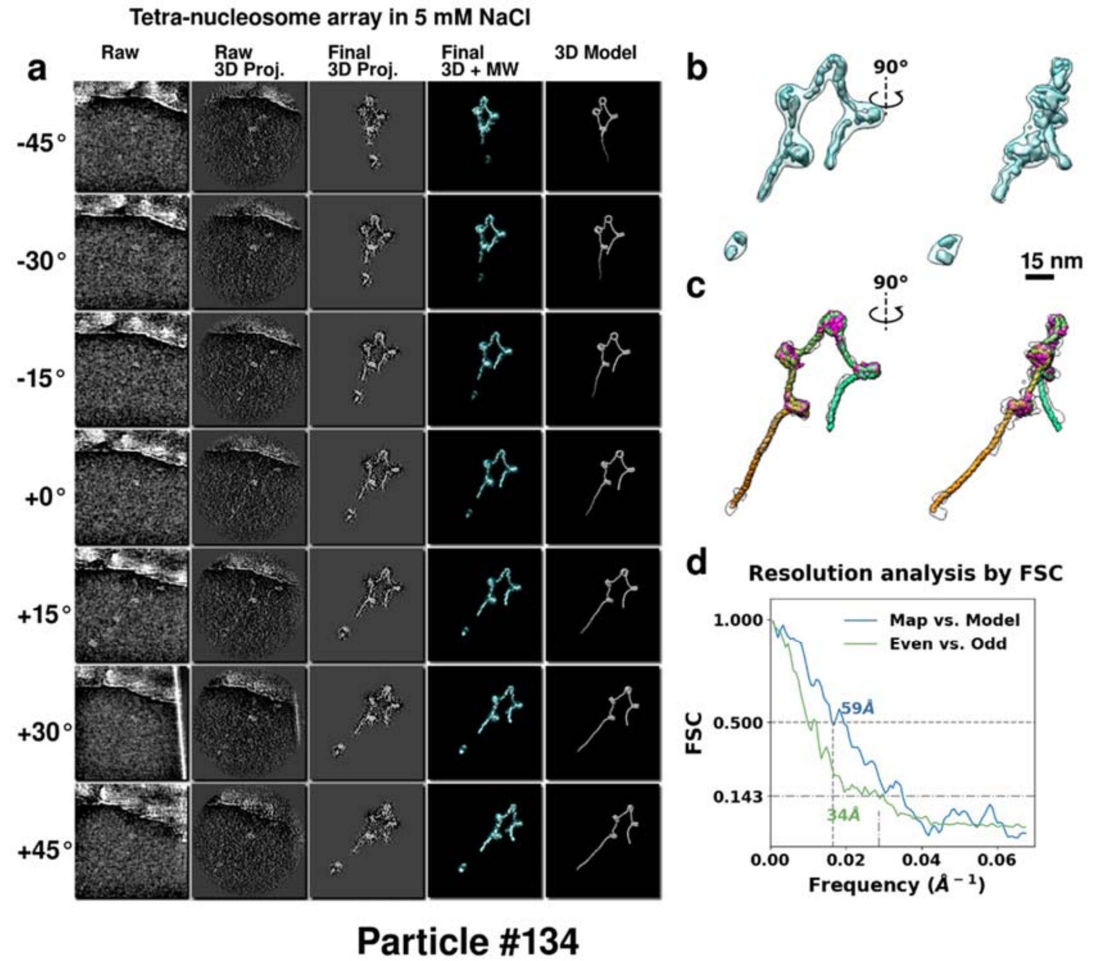

Supplementary Fig. 147. Cryo-ET 3D reconstruction of individual tetra-nucleosome particle (index no. 134) in 5 mM NaCl. a, IPET 3D reconstruction of individual tetra-nucleosome particles. The first column shows seven representative tilt images of an individual particle after CTF correction. Through alignment of the tilt images to a common center for 3D reconstruction via iterative refinement, the second and third columns display the 3D projections of the reconstruction before and after particle-shaped masking, respectively. The fourth column shows the final 3D reconstruction with missing wedge correction, and the fifth column presents the flexibly fitted model at the corresponding tilt angles. **b**, Zoomed-in view of the final 3D density map displayed in orthogonal views, shown at two contour levels. **c**, Superimposition of the high contour level map from (b) onto its flexibly fitted model. **d**, Resolution evaluation of the final 3D density map using two criteria: Fourier shell correlation (FSC) between two-half maps reconstructed from the even and odd index of the tilted series and FSC between the final 3D map and the fitted structure model. The resolution for the former and latter criteria is evaluated at frequencies of 0.5 and 0.143, respectively.

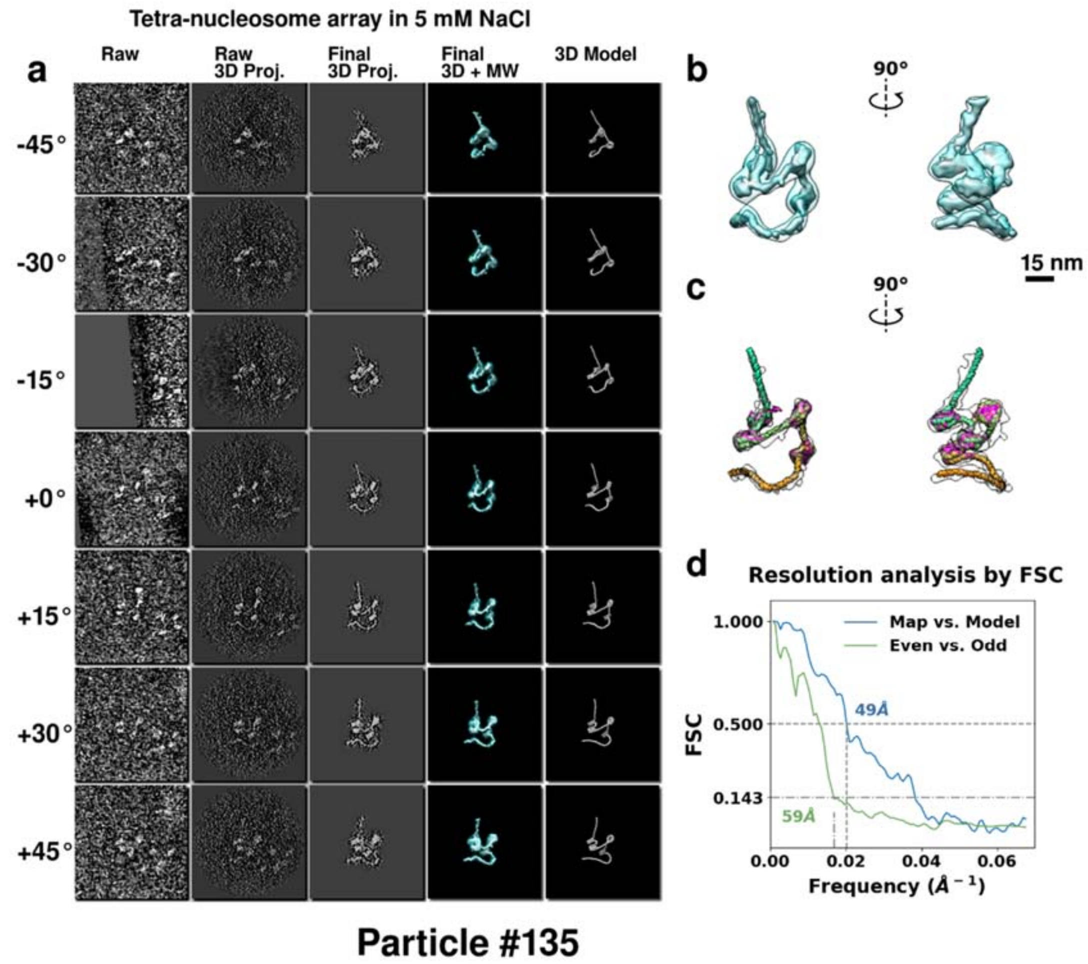

Supplementary Fig. 148. Cryo-ET 3D reconstruction of individual tetra-nucleosome particle (index no. 135) in 5 mM NaCl. a, IPET 3D reconstruction of individual tetra-nucleosome particles. The first column shows seven representative tilt images of an individual particle after CTF correction. Through alignment of the tilt images to a common center for 3D reconstruction via iterative refinement, the second and third columns display the 3D projections of the reconstruction before and after particle-shaped masking, respectively. The fourth column shows the final 3D reconstruction with missing wedge correction, and the fifth column presents the flexibly fitted model at the corresponding tilt angles. **b**, Zoomed-in view of the final 3D density map displayed in orthogonal views, shown at two contour levels. **c**, Superimposition of the high contour level map from (b) onto its flexibly fitted model. **d**, Resolution evaluation of the final 3D density map using two criteria: Fourier shell correlation (FSC) between two-half maps reconstructed from the even and odd index of the tilted series and FSC between the final 3D map and the fitted structure model. The resolution for the former and latter criteria is evaluated at frequencies of 0.5 and 0.143, respectively.

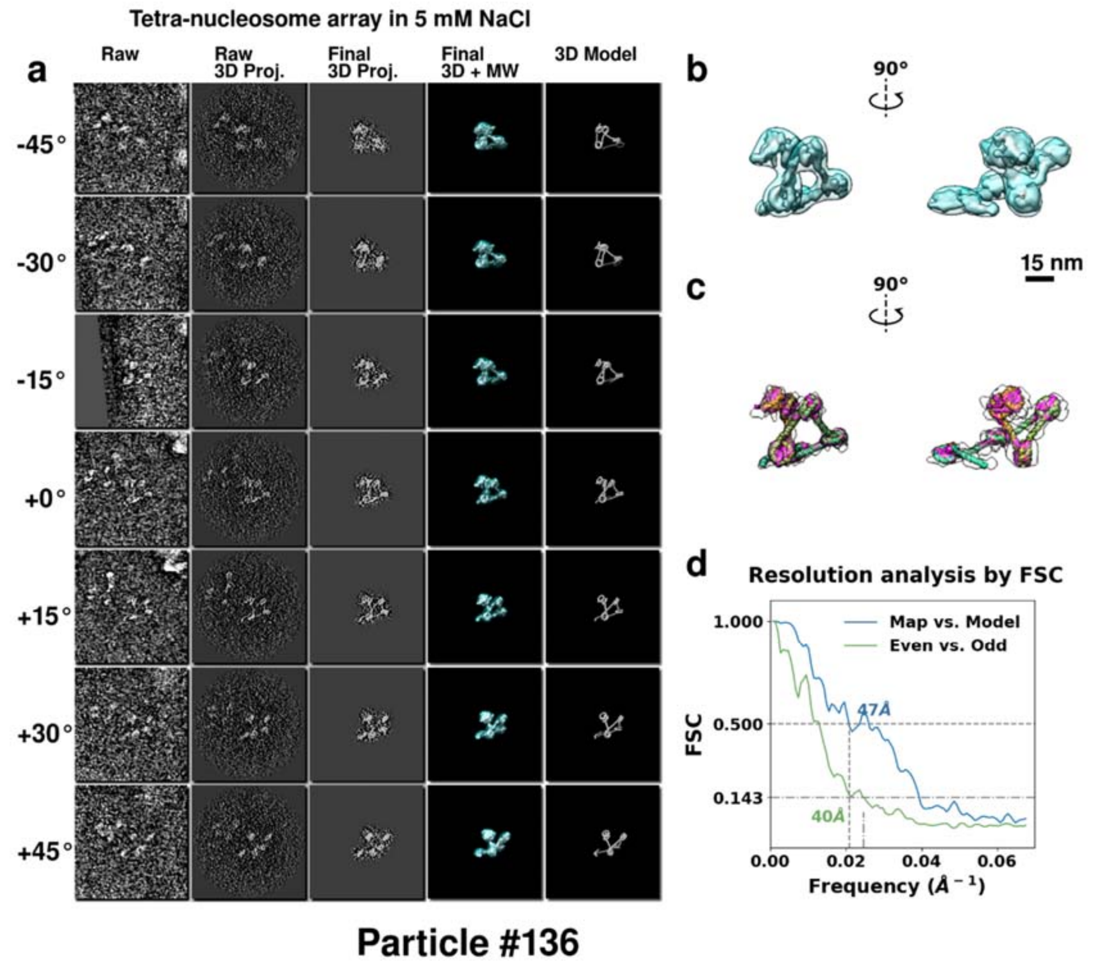

Supplementary Fig. 149. Cryo-ET 3D reconstruction of individual tetra-nucleosome particle (index no. 136) in 5 mM NaCl. a, IPET 3D reconstruction of individual tetra-nucleosome particles. The first column shows seven representative tilt images of an individual particle after CTF correction. Through alignment of the tilt images to a common center for 3D reconstruction via iterative refinement, the second and third columns display the 3D projections of the reconstruction before and after particle-shaped masking, respectively. The fourth column shows the final 3D reconstruction with missing wedge correction, and the fifth column presents the flexibly fitted model at the corresponding tilt angles. **b**, Zoomed-in view of the final 3D density map displayed in orthogonal views, shown at two contour levels. **c**, Superimposition of the high contour level map from (b) onto its flexibly fitted model. **d**, Resolution evaluation of the final 3D density map using two criteria: Fourier shell correlation (FSC) between two-half maps reconstructed from the even and odd index of the tilted series and FSC between the final 3D map and the fitted structure model. The resolution for the former and latter criteria is evaluated at frequencies of 0.5 and 0.143, respectively.

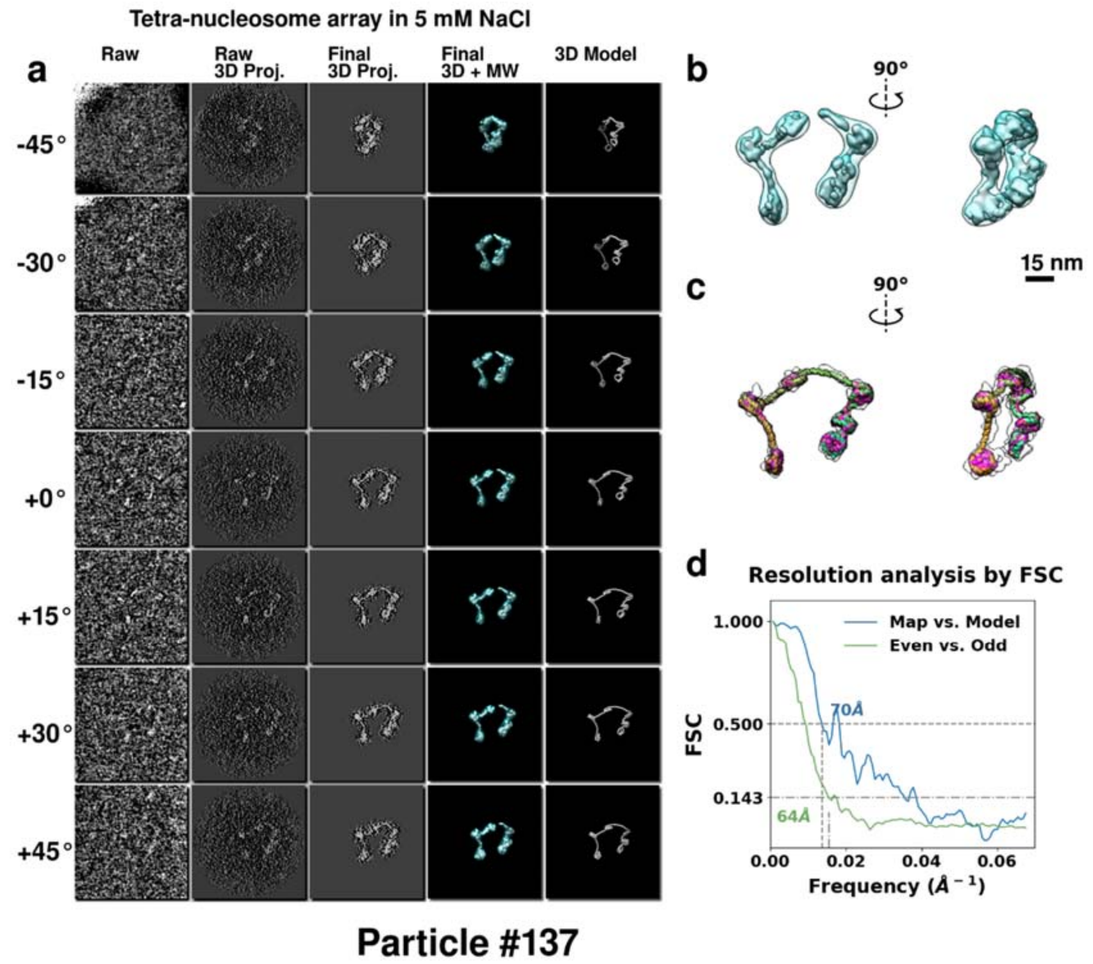

Supplementary Fig. 150. Cryo-ET 3D reconstruction of individual tetra-nucleosome particle (index no. 137) in 5 mM NaCl. a, IPET 3D reconstruction of individual tetra-nucleosome particles. The first column shows seven representative tilt images of an individual particle after CTF correction. Through alignment of the tilt images to a common center for 3D reconstruction via iterative refinement, the second and third columns display the 3D projections of the reconstruction before and after particle-shaped masking, respectively. The fourth column shows the final 3D reconstruction with missing wedge correction, and the fifth column presents the flexibly fitted model at the corresponding tilt angles. **b**, Zoomed-in view of the final 3D density map displayed in orthogonal views, shown at two contour levels. **c**, Superimposition of the high contour level map from (b) onto its flexibly fitted model. **d**, Resolution evaluation of the final 3D density map using two criteria: Fourier shell correlation (FSC) between two-half maps reconstructed from the even and odd index of the tilted series and FSC between the final 3D map and the fitted structure model. The resolution for the former and latter criteria is evaluated at frequencies of 0.5 and 0.143, respectively.

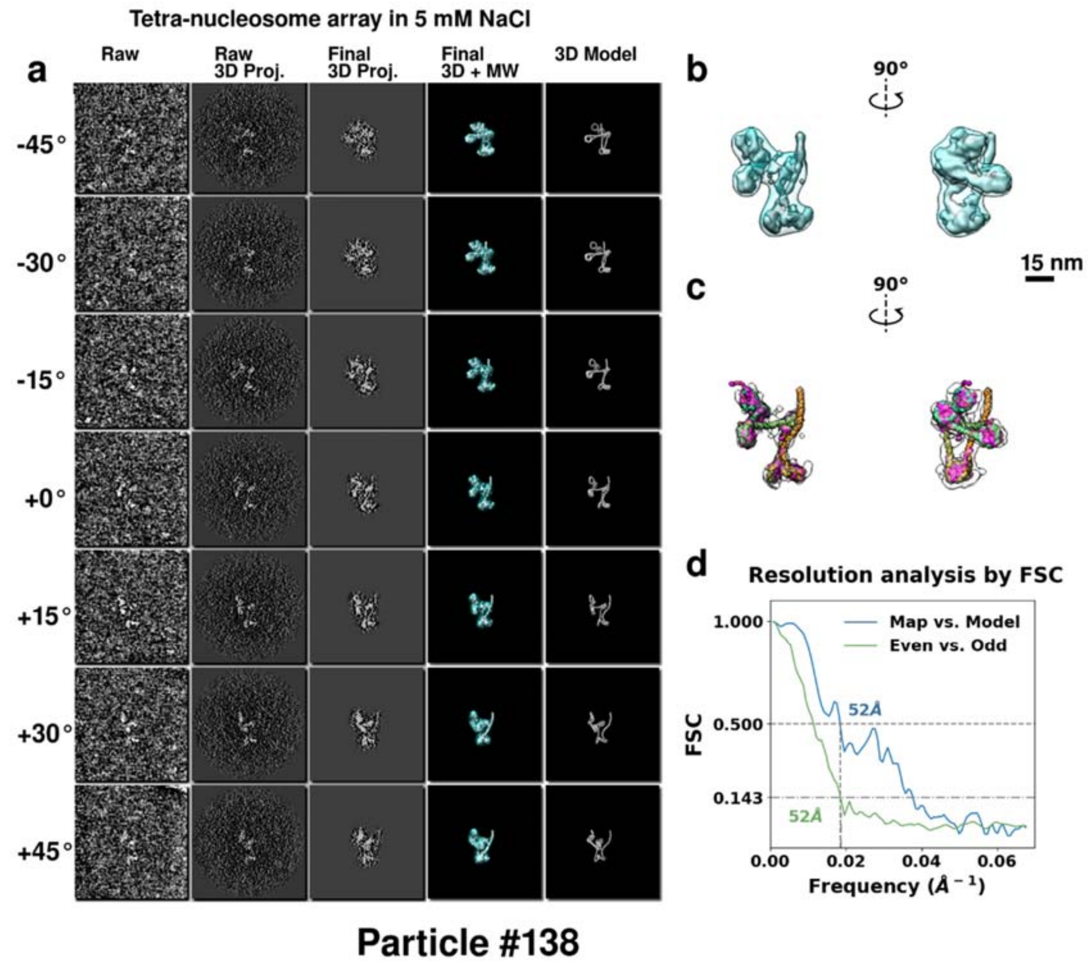

Supplementary Fig. 151. Cryo-ET 3D reconstruction of individual tetra-nucleosome particle (index no. 138) in 5 mM NaCl. a, IPET 3D reconstruction of individual tetra-nucleosome particles. The first column shows seven representative tilt images of an individual particle after CTF correction. Through alignment of the tilt images to a common center for 3D reconstruction via iterative refinement, the second and third columns display the 3D projections of the reconstruction before and after particle-shaped masking, respectively. The fourth column shows the final 3D reconstruction with missing wedge correction, and the fifth column presents the flexibly fitted model at the corresponding tilt angles. **b**, Zoomed-in view of the final 3D density map displayed in orthogonal views, shown at two contour levels. **c**, Superimposition of the high contour level map from (b) onto its flexibly fitted model. **d**, Resolution evaluation of the final 3D density map using two criteria: Fourier shell correlation (FSC) between two-half maps reconstructed from the even and odd index of the tilted series and FSC between the final 3D map and the fitted structure model. The resolution for the former and latter criteria is evaluated at frequencies of 0.5 and 0.143, respectively.

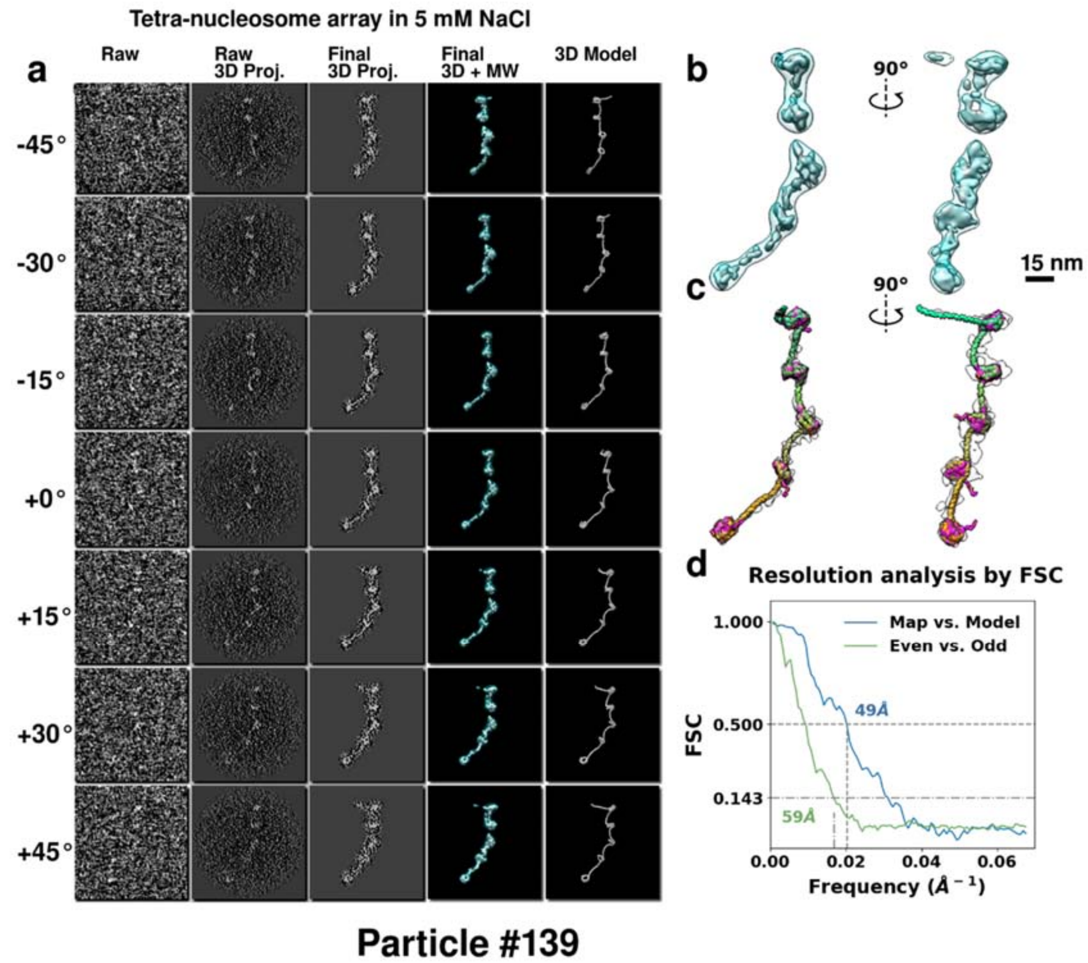

Supplementary Fig. 152. Cryo-ET 3D reconstruction of individual tetra-nucleosome particle (index no. 139) in 5 mM NaCl. a, IPET 3D reconstruction of individual tetra-nucleosome particles. The first column shows seven representative tilt images of an individual particle after CTF correction. Through alignment of the tilt images to a common center for 3D reconstruction via iterative refinement, the second and third columns display the 3D projections of the reconstruction before and after particle-shaped masking, respectively. The fourth column shows the final 3D reconstruction with missing wedge correction, and the fifth column presents the flexibly fitted model at the corresponding tilt angles. **b**, Zoomed-in view of the final 3D density map displayed in orthogonal views, shown at two contour levels. **c**, Superimposition of the high contour level map from (b) onto its flexibly fitted model. **d**, Resolution evaluation of the final 3D density map using two criteria: Fourier shell correlation (FSC) between two-half maps reconstructed from the even and odd index of the tilted series and FSC between the final 3D map and the fitted structure model. The resolution for the former and latter criteria is evaluated at frequencies of 0.5 and 0.143, respectively.

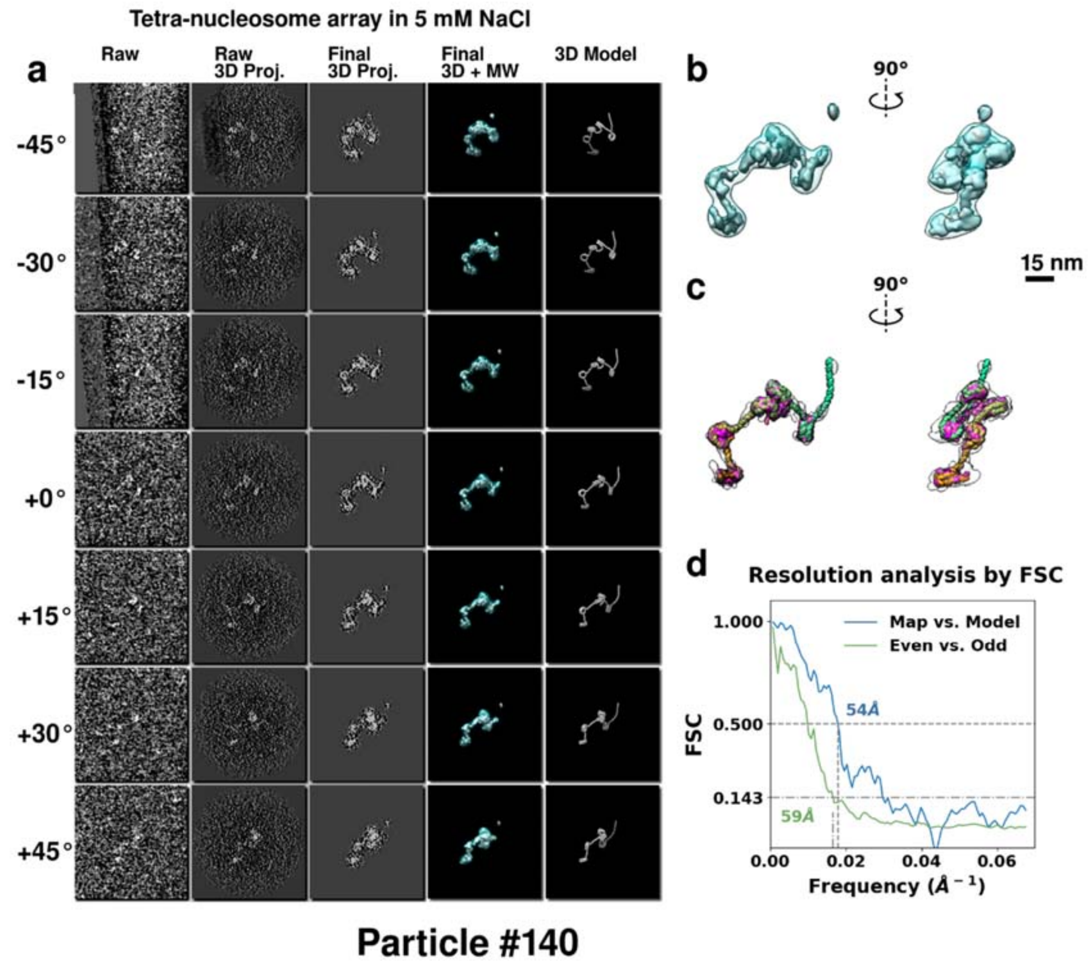

Supplementary Fig. 153. Cryo-ET 3D reconstruction of individual tetra-nucleosome particle (index no. 140) in 5 mM NaCl. **a**, IPET 3D reconstruction of individual tetra-nucleosome particles. The first column shows seven representative tilt images of an individual particle after CTF correction. Through alignment of the tilt images to a common center for 3D reconstruction via iterative refinement, the second and third columns display the 3D projections of the reconstruction before and after particle-shaped masking, respectively. The fourth column shows the final 3D reconstruction with missing wedge correction, and the fifth column presents the flexibly fitted model at the corresponding tilt angles. **b**, Zoomed-in view of the final 3D density map displayed in orthogonal views, shown at two contour levels. **c**, Superimposition of the high contour level map from (b) onto its flexibly fitted model. **d**, Resolution evaluation of the final 3D density map using two criteria: Fourier shell correlation (FSC) between two-half maps reconstructed from the even and odd index of the tilted series and FSC between the final 3D map and the fitted structure model. The resolution for the former and latter criteria is evaluated at frequencies of 0.5 and 0.143, respectively.

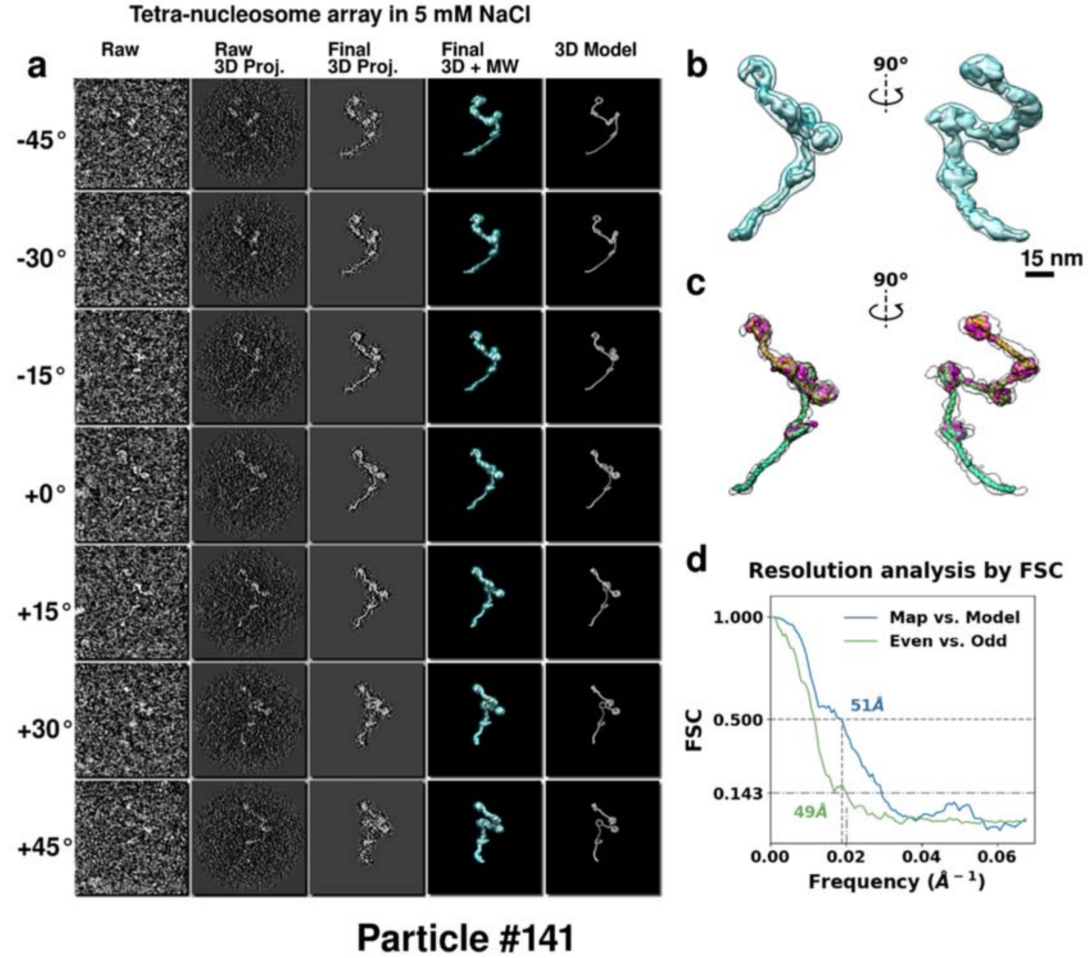

Supplementary Fig. 154. Cryo-ET 3D reconstruction of individual tetra-nucleosome particle (index no. 141) in 5 mM NaCl. a, IPET 3D reconstruction of individual tetra-nucleosome particles. The first column shows seven representative tilt images of an individual particle after CTF correction. Through alignment of the tilt images to a common center for 3D reconstruction via iterative refinement, the second and third columns display the 3D projections of the reconstruction before and after particle-shaped masking, respectively. The fourth column shows the final 3D reconstruction with missing wedge correction, and the fifth column presents the flexibly fitted model at the corresponding tilt angles. **b**, Zoomed-in view of the final 3D density map displayed in orthogonal views, shown at two contour levels. **c**, Superimposition of the high contour level map from (b) onto its flexibly fitted model. **d**, Resolution evaluation of the final 3D density map using two criteria: Fourier shell correlation (FSC) between two-half maps reconstructed from the even and odd index of the tilted series and FSC between the final 3D map and the fitted structure model. The resolution for the former and latter criteria is evaluated at frequencies of 0.5 and 0.143, respectively.

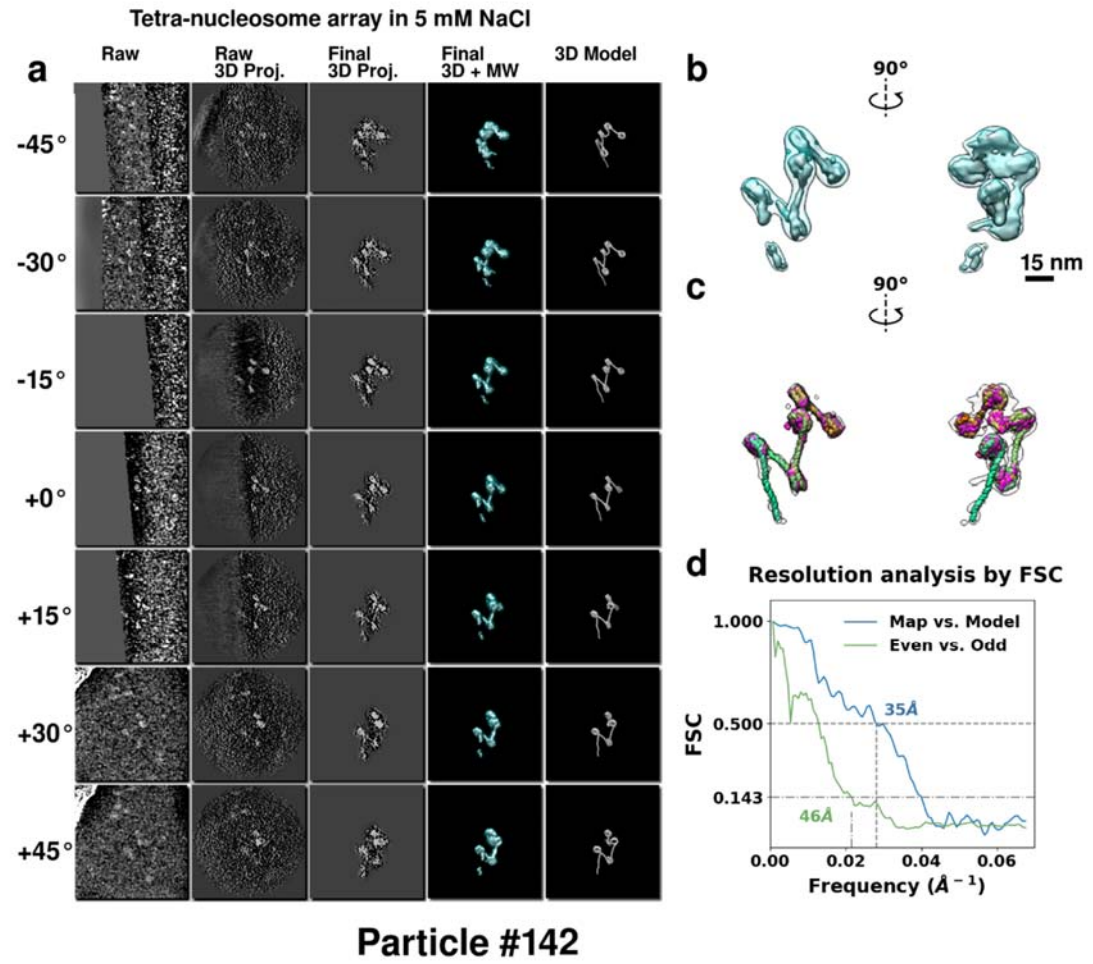

Supplementary Fig. 155. Cryo-ET 3D reconstruction of individual tetra-nucleosome particle (index no. 142) in 5 mM NaCl. a, IPET 3D reconstruction of individual tetra-nucleosome particles. The first column shows seven representative tilt images of an individual particle after CTF correction. Through alignment of the tilt images to a common center for 3D reconstruction via iterative refinement, the second and third columns display the 3D projections of the reconstruction before and after particle-shaped masking, respectively. The fourth column shows the final 3D reconstruction with missing wedge correction, and the fifth column presents the flexibly fitted model at the corresponding tilt angles. **b**, Zoomed-in view of the final 3D density map displayed in orthogonal views, shown at two contour levels. **c**, Superimposition of the high contour level map from (b) onto its flexibly fitted model. **d**, Resolution evaluation of the final 3D density map using two criteria: Fourier shell correlation (FSC) between two-half maps reconstructed from the even and odd index of the tilted series and FSC between the final 3D map and the fitted structure model. The resolution for the former and latter criteria is evaluated at frequencies of 0.5 and 0.143, respectively.

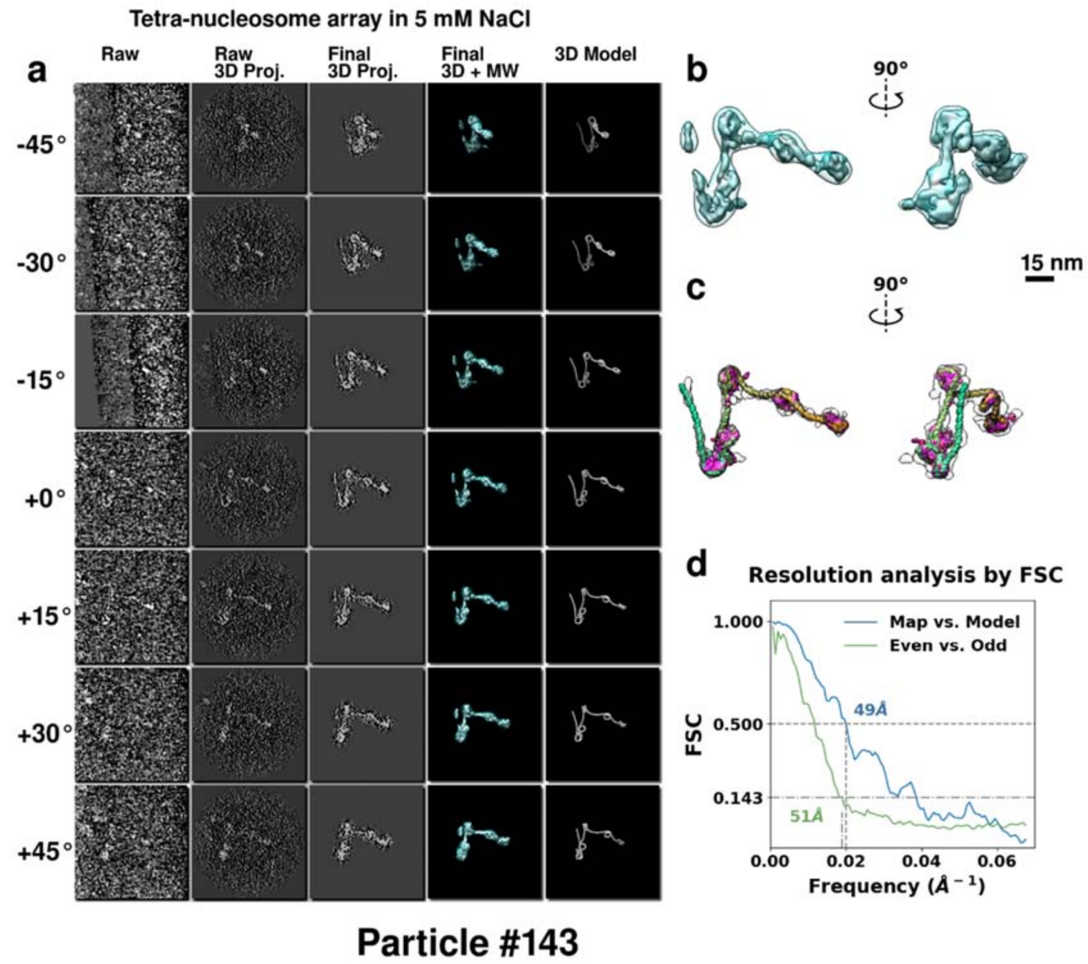

Supplementary Fig. 156. Cryo-ET 3D reconstruction of individual tetra-nucleosome particle (index no. 143) in 5 mM NaCl. a, IPET 3D reconstruction of individual tetra-nucleosome particles. The first column shows seven representative tilt images of an individual particle after CTF correction. Through alignment of the tilt images to a common center for 3D reconstruction via iterative refinement, the second and third columns display the 3D projections of the reconstruction before and after particle-shaped masking, respectively. The fourth column shows the final 3D reconstruction with missing wedge correction, and the fifth column presents the flexibly fitted model at the corresponding tilt angles. **b**, Zoomed-in view of the final 3D density map displayed in orthogonal views, shown at two contour levels. **c**, Superimposition of the high contour level map from (b) onto its flexibly fitted model. **d**, Resolution evaluation of the final 3D density map using two criteria: Fourier shell correlation (FSC) between two-half maps reconstructed from the even and odd index of the tilted series and FSC between the final 3D map and the fitted structure model. The resolution for the former and latter criteria is evaluated at frequencies of 0.5 and 0.143, respectively.

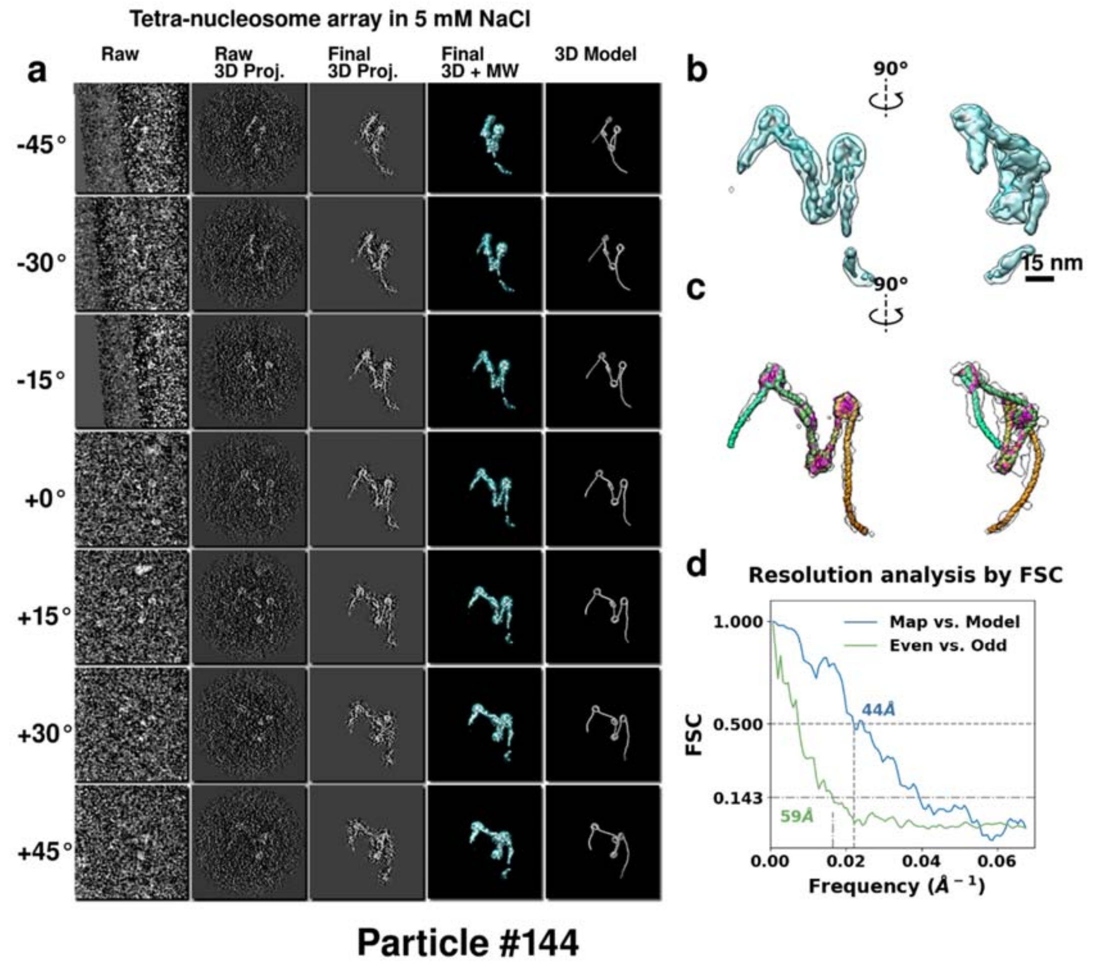

Supplementary Fig. 157. Cryo-ET 3D reconstruction of individual tetra-nucleosome particle (index no. 144) in 5 mM NaCl. a, IPET 3D reconstruction of individual tetra-nucleosome particles. The first column shows seven representative tilt images of an individual particle after CTF correction. Through alignment of the tilt images to a common center for 3D reconstruction via iterative refinement, the second and third columns display the 3D projections of the reconstruction before and after particle-shaped masking, respectively. The fourth column shows the final 3D reconstruction with missing wedge correction, and the fifth column presents the flexibly fitted model at the corresponding tilt angles. **b**, Zoomed-in view of the final 3D density map displayed in orthogonal views, shown at two contour levels. **c**, Superimposition of the high contour level map from (b) onto its flexibly fitted model. **d**, Resolution evaluation of the final 3D density map using two criteria: Fourier shell correlation (FSC) between two-half maps reconstructed from the even and odd index of the tilted series and FSC between the final 3D map and the fitted structure model. The resolution for the former and latter criteria is evaluated at frequencies of 0.5 and 0.143, respectively.

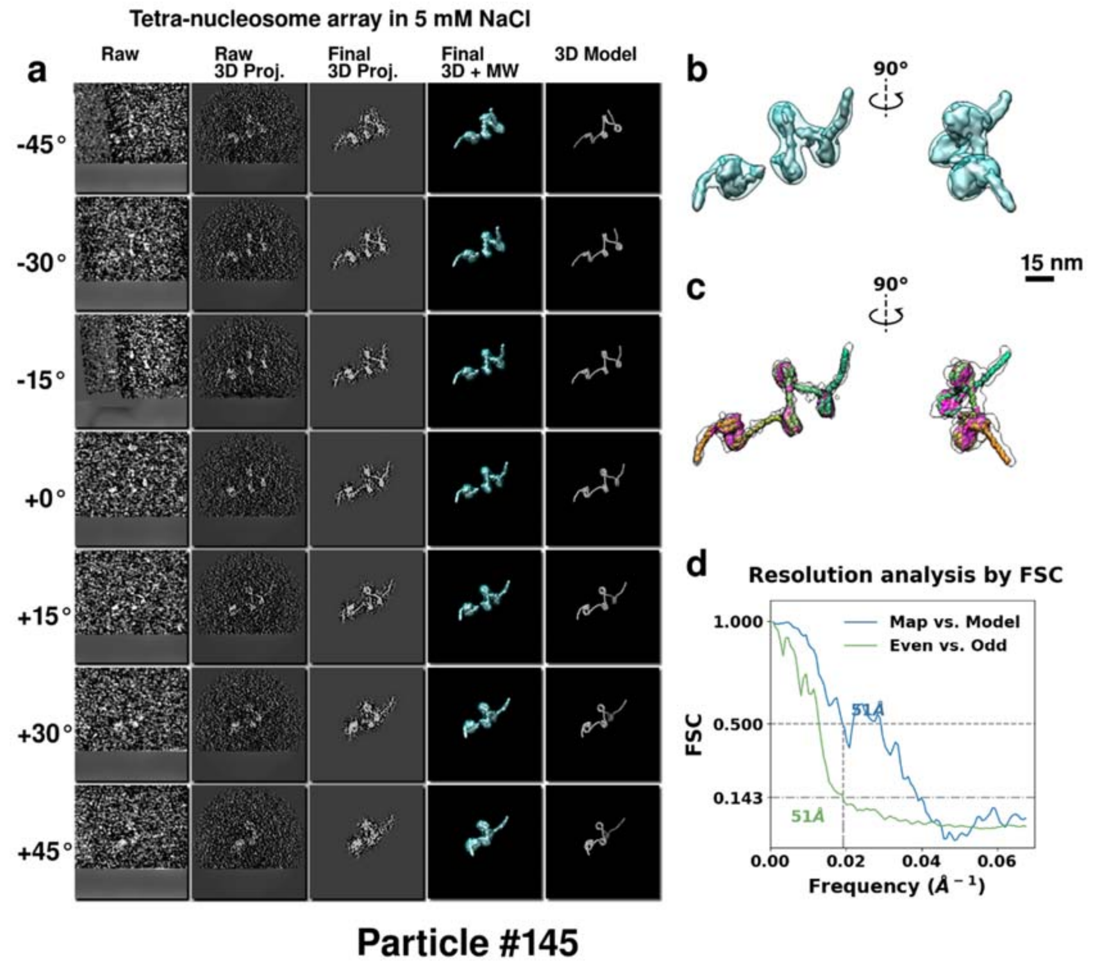

Supplementary Fig. 158. Cryo-ET 3D reconstruction of individual tetra-nucleosome particle (index no. 145) in 5 mM NaCl. a, IPET 3D reconstruction of individual tetra-nucleosome particles. The first column shows seven representative tilt images of an individual particle after CTF correction. Through alignment of the tilt images to a common center for 3D reconstruction via iterative refinement, the second and third columns display the 3D projections of the reconstruction before and after particle-shaped masking, respectively. The fourth column shows the final 3D reconstruction with missing wedge correction, and the fifth column presents the flexibly fitted model at the corresponding tilt angles. **b**, Zoomed-in view of the final 3D density map displayed in orthogonal views, shown at two contour levels. **c**, Superimposition of the high contour level map from (b) onto its flexibly fitted model. **d**, Resolution evaluation of the final 3D density map using two criteria: Fourier shell correlation (FSC) between two-half maps reconstructed from the even and odd index of the tilted series and FSC between the final 3D map and the fitted structure model. The resolution for the former and latter criteria is evaluated at frequencies of 0.5 and 0.143, respectively.

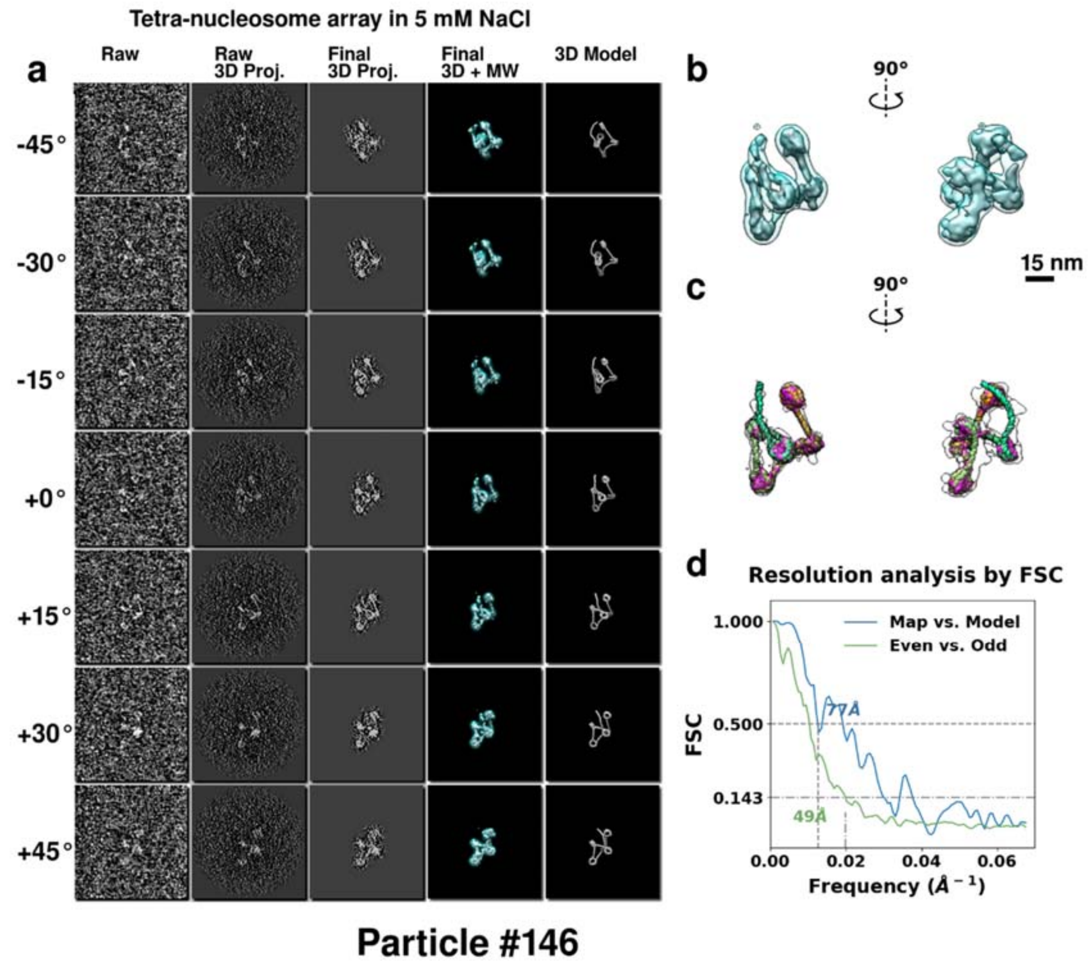

Supplementary Fig. 159. Cryo-ET 3D reconstruction of individual tetra-nucleosome particle (index no. 146) in 5 mM NaCl. a, IPET 3D reconstruction of individual tetra-nucleosome particles. The first column shows seven representative tilt images of an individual particle after CTF correction. Through alignment of the tilt images to a common center for 3D reconstruction via iterative refinement, the second and third columns display the 3D projections of the reconstruction before and after particle-shaped masking, respectively. The fourth column shows the final 3D reconstruction with missing wedge correction, and the fifth column presents the flexibly fitted model at the corresponding tilt angles. **b**, Zoomed-in view of the final 3D density map displayed in orthogonal views, shown at two contour levels. **c**, Superimposition of the high contour level map from (b) onto its flexibly fitted model. **d**, Resolution evaluation of the final 3D density map using two criteria: Fourier shell correlation (FSC) between two-half maps reconstructed from the even and odd index of the tilted series and FSC between the final 3D map and the fitted structure model. The resolution for the former and latter criteria is evaluated at frequencies of 0.5 and 0.143, respectively.

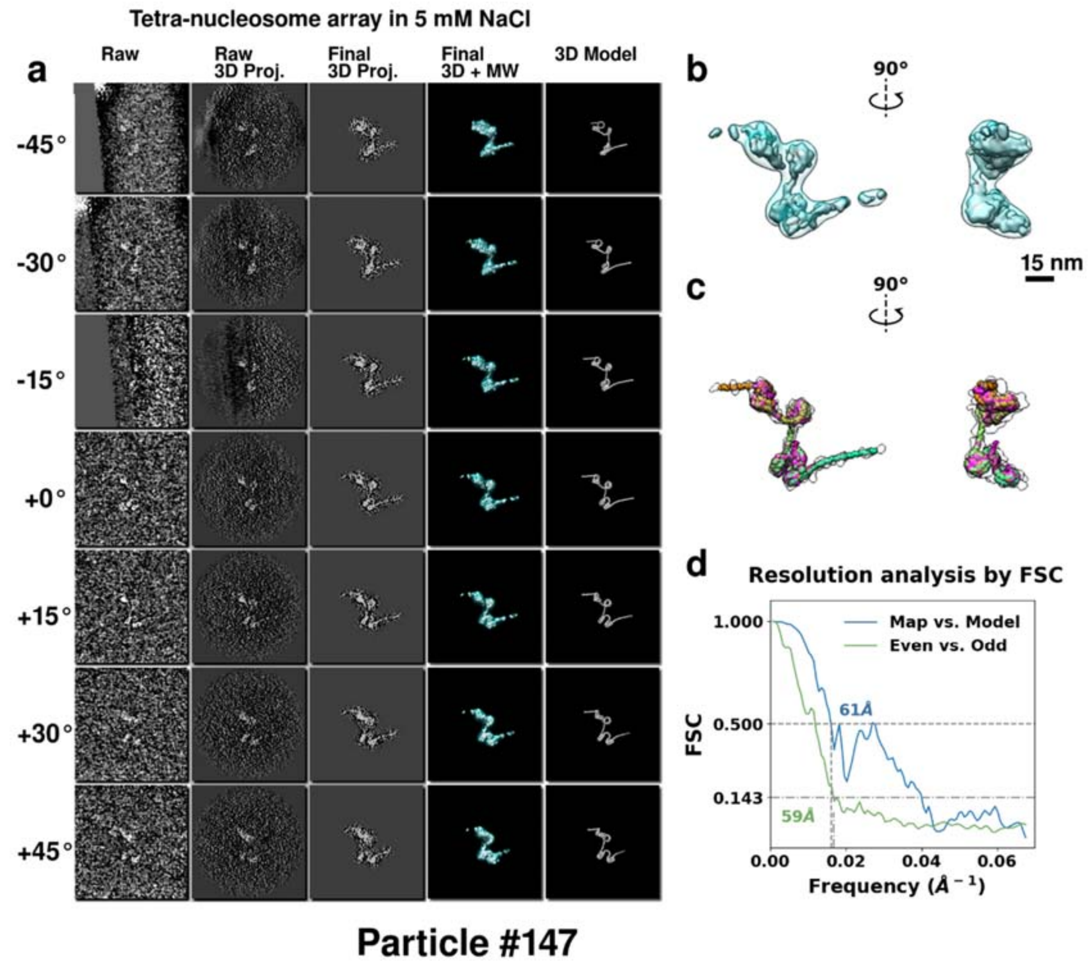

Supplementary Fig. 160. Cryo-ET 3D reconstruction of individual tetra-nucleosome particle (index no. 147) in 5 mM NaCl. a, IPET 3D reconstruction of individual tetra-nucleosome particles. The first column shows seven representative tilt images of an individual particle after CTF correction. Through alignment of the tilt images to a common center for 3D reconstruction via iterative refinement, the second and third columns display the 3D projections of the reconstruction before and after particle-shaped masking, respectively. The fourth column shows the final 3D reconstruction with missing wedge correction, and the fifth column presents the flexibly fitted model at the corresponding tilt angles. **b**, Zoomed-in view of the final 3D density map displayed in orthogonal views, shown at two contour levels. **c**, Superimposition of the high contour level map from (b) onto its flexibly fitted model. **d**, Resolution evaluation of the final 3D density map using two criteria: Fourier shell correlation (FSC) between two-half maps reconstructed from the even and odd index of the tilted series and FSC between the final 3D map and the fitted structure model. The resolution for the former and latter criteria is evaluated at frequencies of 0.5 and 0.143, respectively.

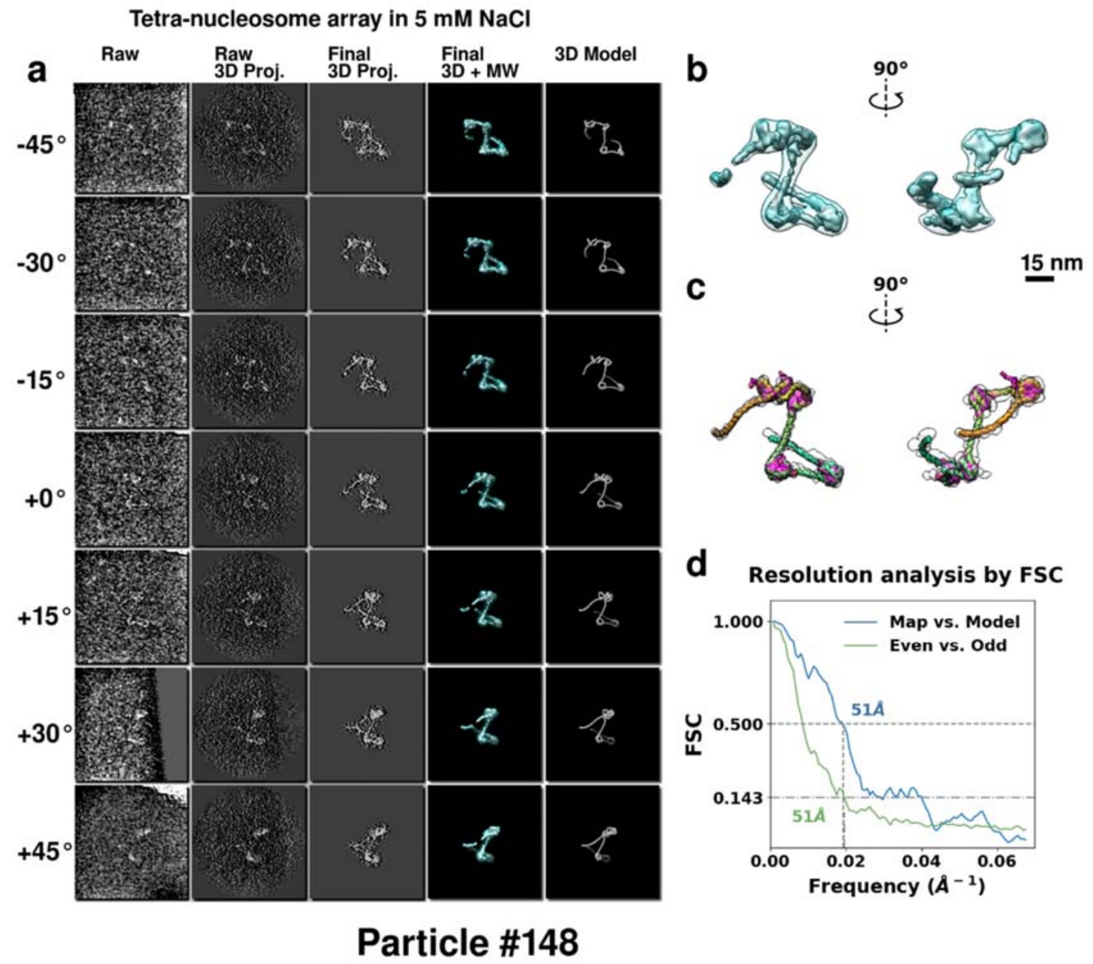

Supplementary Fig. 161. Cryo-ET 3D reconstruction of individual tetra-nucleosome particle (index no. 148) in 5 mM NaCl. a, IPET 3D reconstruction of individual tetra-nucleosome particles. The first column shows seven representative tilt images of an individual particle after CTF correction. Through alignment of the tilt images to a common center for 3D reconstruction via iterative refinement, the second and third columns display the 3D projections of the reconstruction before and after particle-shaped masking, respectively. The fourth column shows the final 3D reconstruction with missing wedge correction, and the fifth column presents the flexibly fitted model at the corresponding tilt angles. **b**, Zoomed-in view of the final 3D density map displayed in orthogonal views, shown at two contour levels. **c**, Superimposition of the high contour level map from (b) onto its flexibly fitted model. **d**, Resolution evaluation of the final 3D density map using two criteria: Fourier shell correlation (FSC) between two-half maps reconstructed from the even and odd index of the tilted series and FSC between the final 3D map and the fitted structure model. The resolution for the former and latter criteria is evaluated at frequencies of 0.5 and 0.143, respectively.

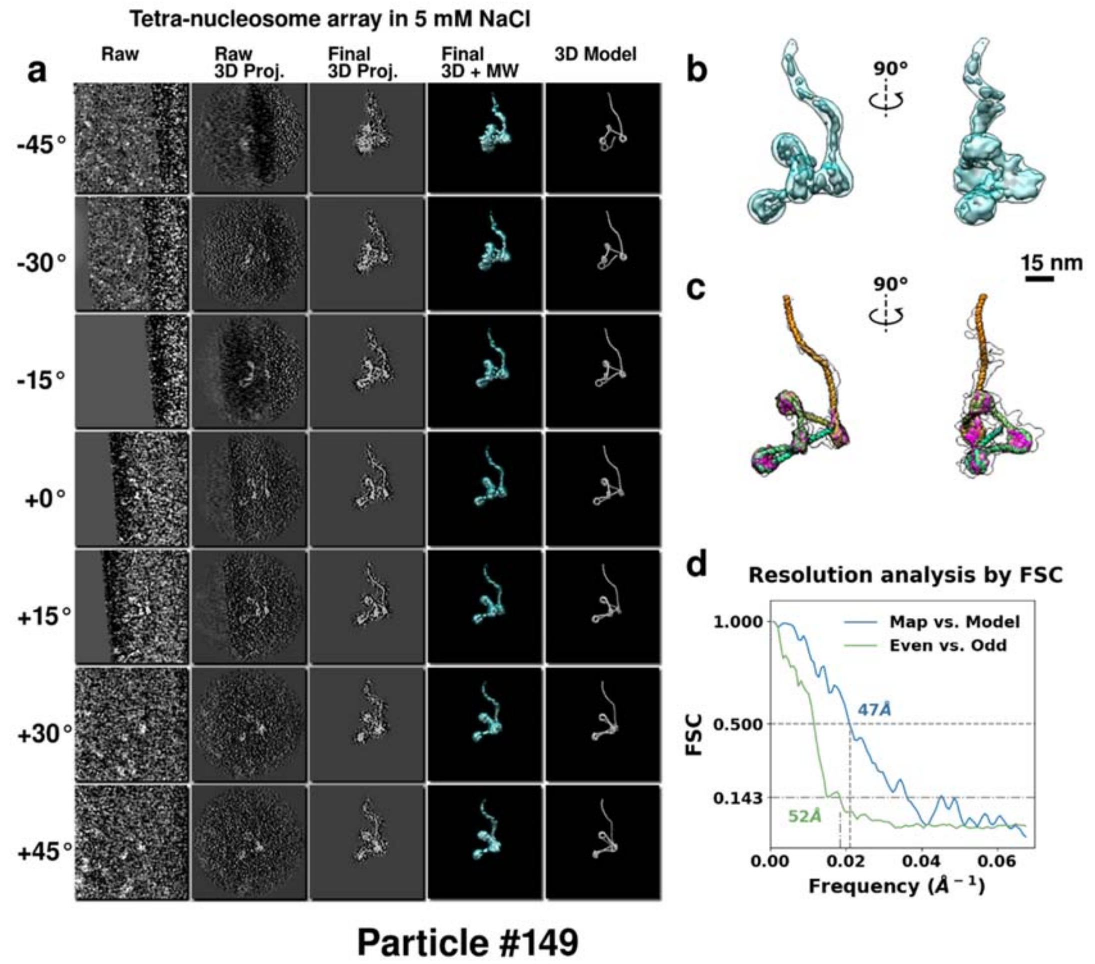

Supplementary Fig. 162. Cryo-ET 3D reconstruction of individual tetra-nucleosome particle (index no. 149) in 5 mM NaCl. a, IPET 3D reconstruction of individual tetra-nucleosome particles. The first column shows seven representative tilt images of an individual particle after CTF correction. Through alignment of the tilt images to a common center for 3D reconstruction via iterative refinement, the second and third columns display the 3D projections of the reconstruction before and after particle-shaped masking, respectively. The fourth column shows the final 3D reconstruction with missing wedge correction, and the fifth column presents the flexibly fitted model at the corresponding tilt angles. **b**, Zoomed-in view of the final 3D density map displayed in orthogonal views, shown at two contour levels. **c**, Superimposition of the high contour level map from (b) onto its flexibly fitted model. **d**, Resolution evaluation of the final 3D density map using two criteria: Fourier shell correlation (FSC) between two-half maps reconstructed from the even and odd index of the tilted series and FSC between the final 3D map and the fitted structure model. The resolution for the former and latter criteria is evaluated at frequencies of 0.5 and 0.143, respectively.

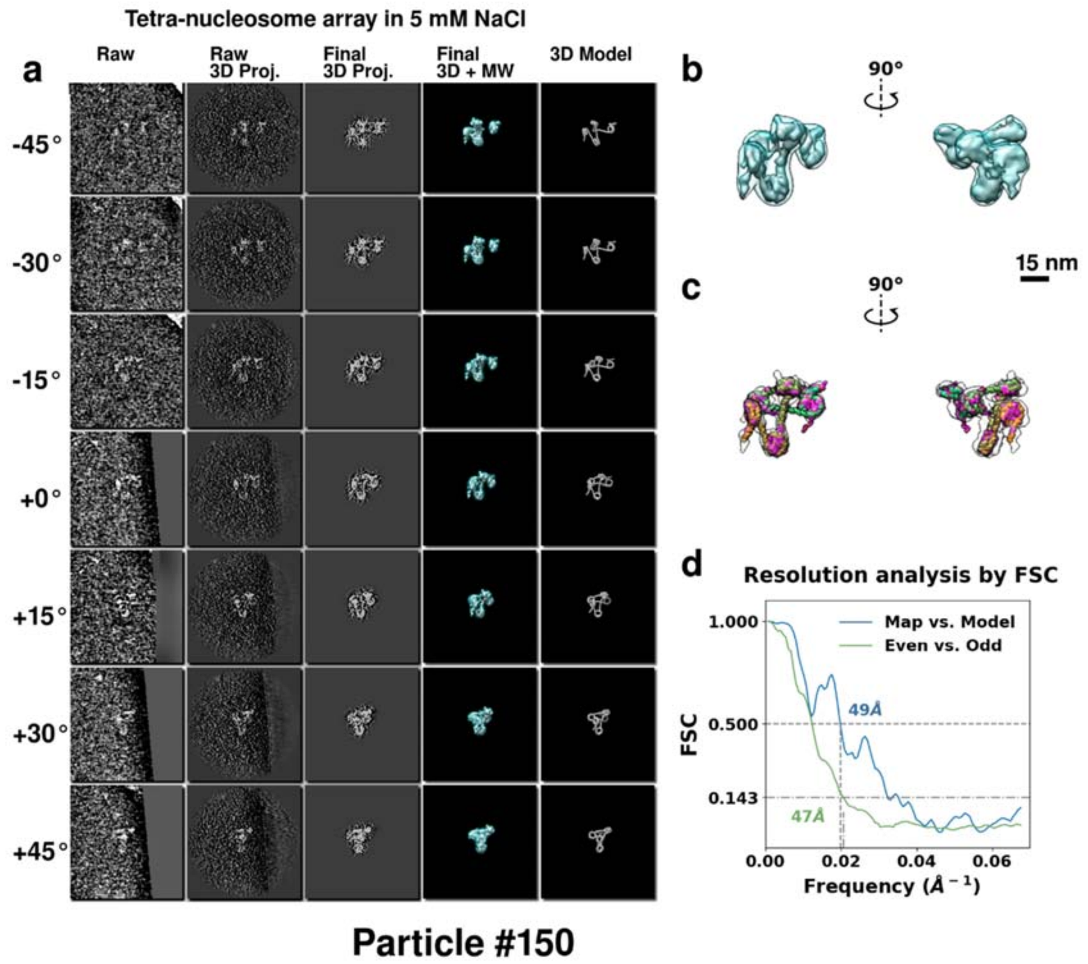

Supplementary Fig. 163. Cryo-ET 3D reconstruction of individual tetra-nucleosome particle (index no. 150) in 5 mM NaCl. a, IPET 3D reconstruction of individual tetra-nucleosome particles. The first column shows seven representative tilt images of an individual particle after CTF correction. Through alignment of the tilt images to a common center for 3D reconstruction via iterative refinement, the second and third columns display the 3D projections of the reconstruction before and after particle-shaped masking, respectively. The fourth column shows the final 3D reconstruction with missing wedge correction, and the fifth column presents the flexibly fitted model at the corresponding tilt angles. **b**, Zoomed-in view of the final 3D density map displayed in orthogonal views, shown at two contour levels. **c**, Superimposition of the high contour level map from (b) onto its flexibly fitted model. **d**, Resolution evaluation of the final 3D density map using two criteria: Fourier shell correlation (FSC) between two-half maps reconstructed from the even and odd index of the tilted series and FSC between the final 3D map and the fitted structure model. The resolution for the former and latter criteria is evaluated at frequencies of 0.5 and 0.143, respectively.

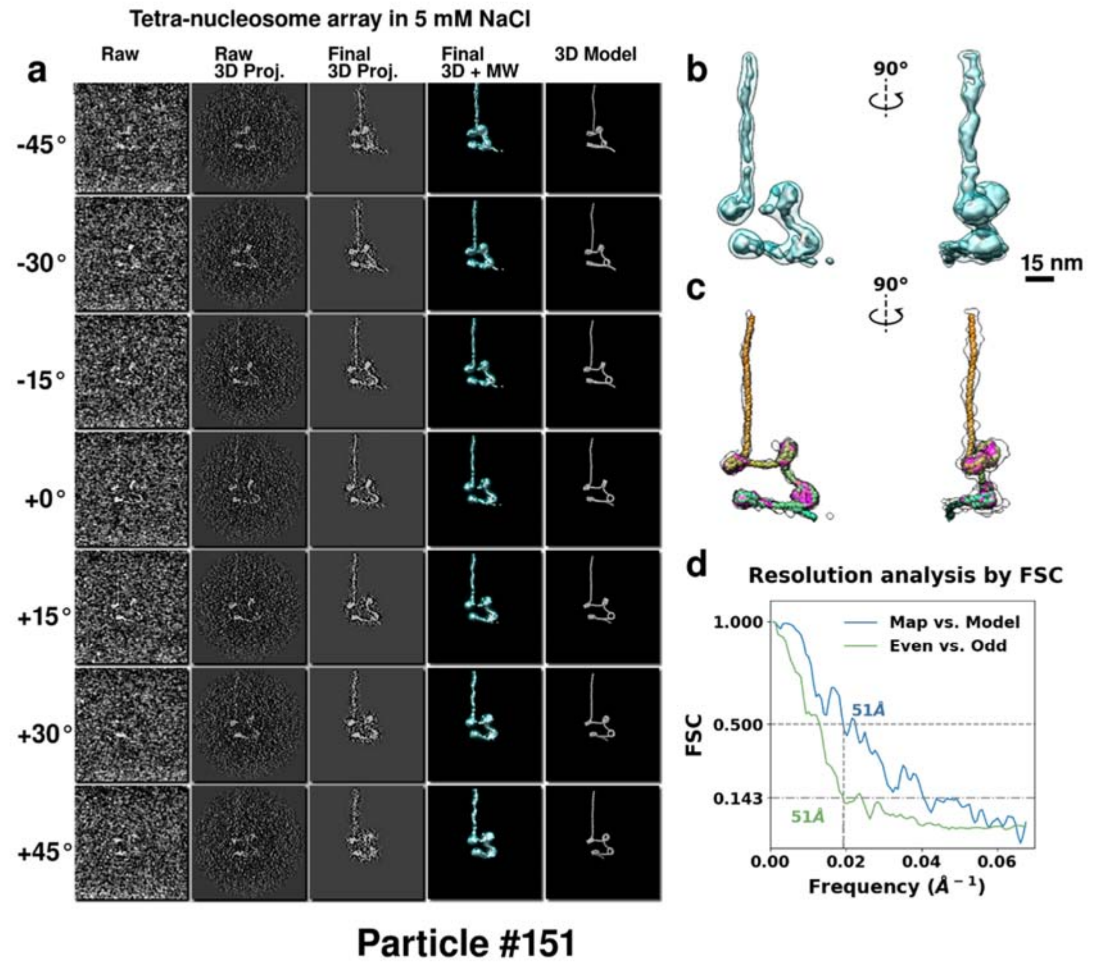

Supplementary Fig. 164. Cryo-ET 3D reconstruction of individual tetra-nucleosome particle (index no. 151) in 5 mM NaCl. a, IPET 3D reconstruction of individual tetra-nucleosome particles. The first column shows seven representative tilt images of an individual particle after CTF correction. Through alignment of the tilt images to a common center for 3D reconstruction via iterative refinement, the second and third columns display the 3D projections of the reconstruction before and after particle-shaped masking, respectively. The fourth column shows the final 3D reconstruction with missing wedge correction, and the fifth column presents the flexibly fitted model at the corresponding tilt angles. **b**, Zoomed-in view of the final 3D density map displayed in orthogonal views, shown at two contour levels. **c**, Superimposition of the high contour level map from (b) onto its flexibly fitted model. **d**, Resolution evaluation of the final 3D density map using two criteria: Fourier shell correlation (FSC) between two-half maps reconstructed from the even and odd index of the tilted series and FSC between the final 3D map and the fitted structure model. The resolution for the former and latter criteria is evaluated at frequencies of 0.5 and 0.143, respectively.

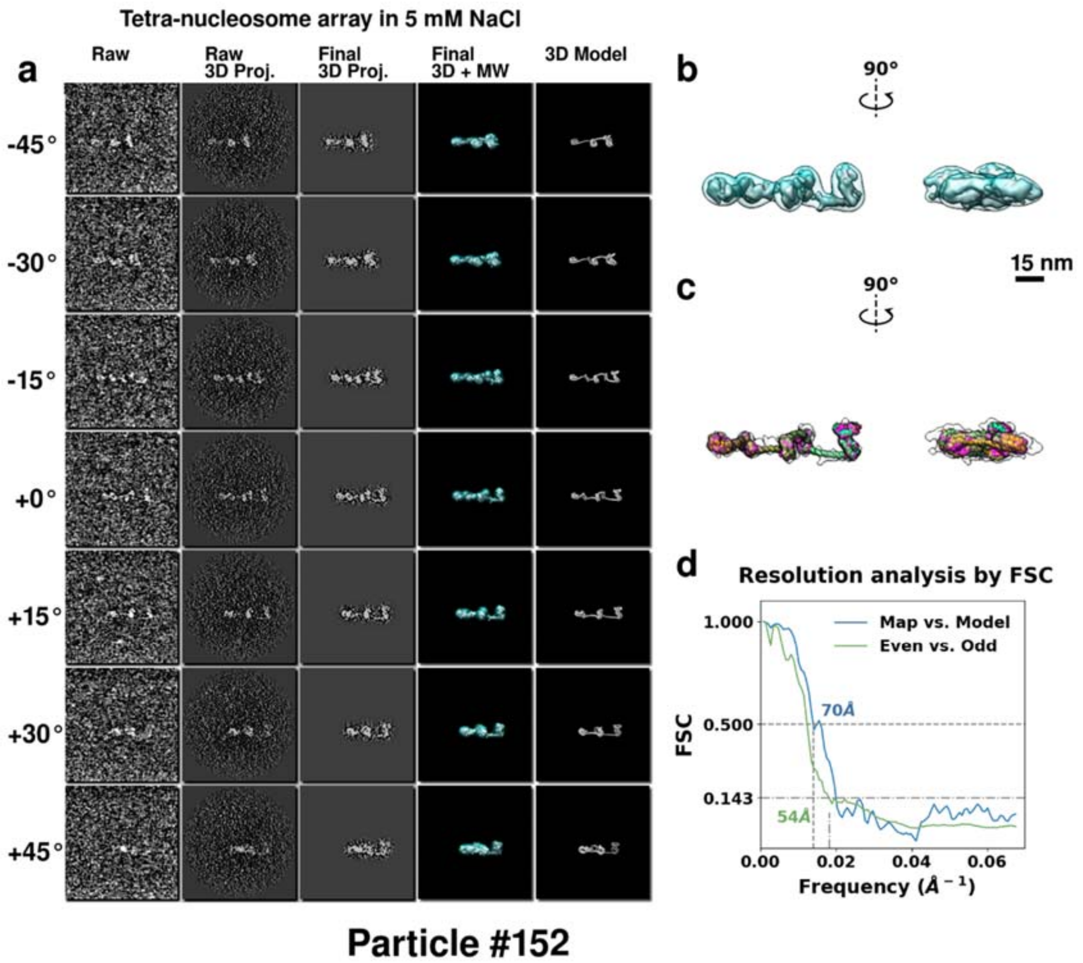

Supplementary Fig. 165. Cryo-ET 3D reconstruction of individual tetra-nucleosome particle (index no. 152) in 5 mM NaCl. a, IPET 3D reconstruction of individual tetra-nucleosome particles. The first column shows seven representative tilt images of an individual particle after CTF correction. Through alignment of the tilt images to a common center for 3D reconstruction via iterative refinement, the second and third columns display the 3D projections of the reconstruction before and after particle-shaped masking, respectively. The fourth column shows the final 3D reconstruction with missing wedge correction, and the fifth column presents the flexibly fitted model at the corresponding tilt angles. **b**, Zoomed-in view of the final 3D density map displayed in orthogonal views, shown at two contour levels. **c**, Superimposition of the high contour level map from (b) onto its flexibly fitted model. **d**, Resolution evaluation of the final 3D density map using two criteria: Fourier shell correlation (FSC) between two-half maps reconstructed from the even and odd index of the tilted series and FSC between the final 3D map and the fitted structure model. The resolution for the former and latter criteria is evaluated at frequencies of 0.5 and 0.143, respectively.

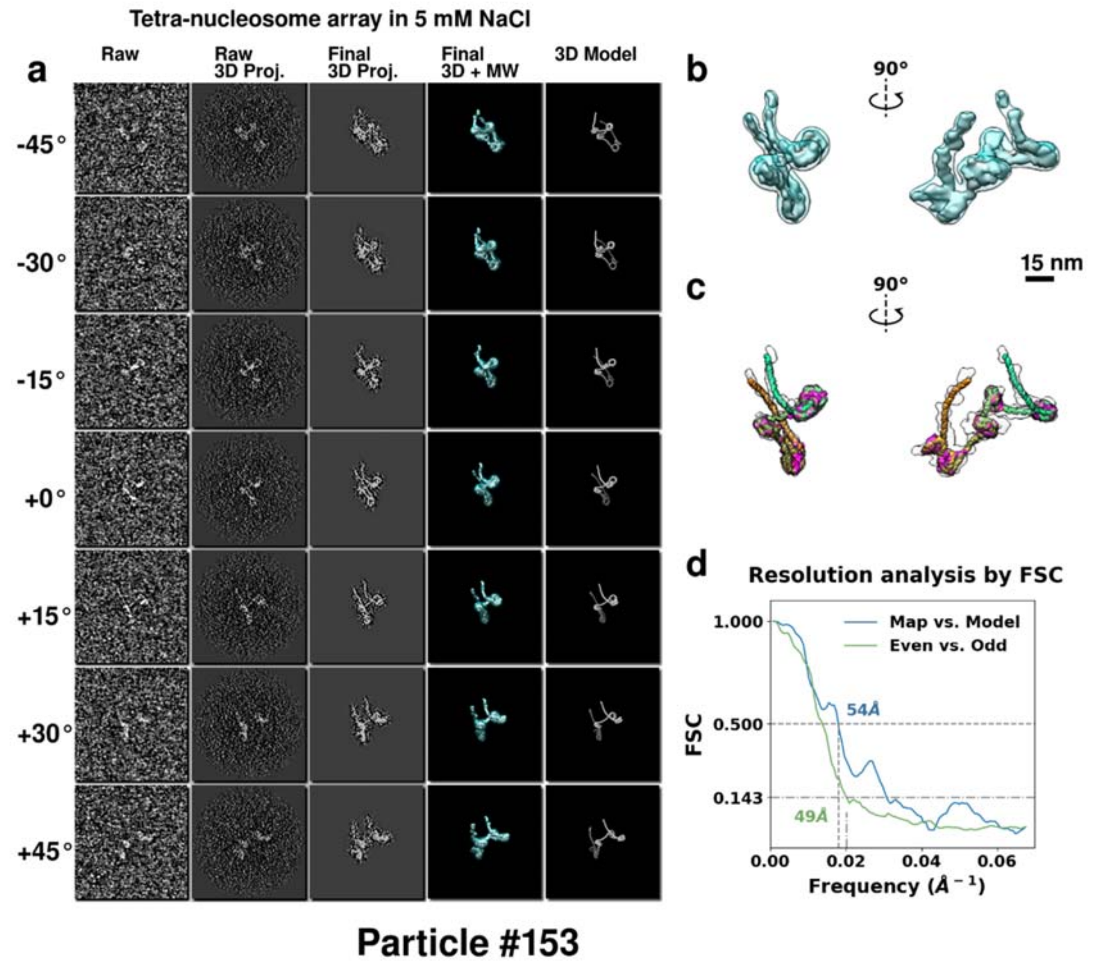

Supplementary Fig. 166. Cryo-ET 3D reconstruction of individual tetra-nucleosome particle (index no. 153) in 5 mM NaCl. a, IPET 3D reconstruction of individual tetra-nucleosome particles. The first column shows seven representative tilt images of an individual particle after CTF correction. Through alignment of the tilt images to a common center for 3D reconstruction via iterative refinement, the second and third columns display the 3D projections of the reconstruction before and after particle-shaped masking, respectively. The fourth column shows the final 3D reconstruction with missing wedge correction, and the fifth column presents the flexibly fitted model at the corresponding tilt angles. **b**, Zoomed-in view of the final 3D density map displayed in orthogonal views, shown at two contour levels. **c**, Superimposition of the high contour level map from (b) onto its flexibly fitted model. **d**, Resolution evaluation of the final 3D density map using two criteria: Fourier shell correlation (FSC) between two-half maps reconstructed from the even and odd index of the tilted series and FSC between the final 3D map and the fitted structure model. The resolution for the former and latter criteria is evaluated at frequencies of 0.5 and 0.143, respectively.

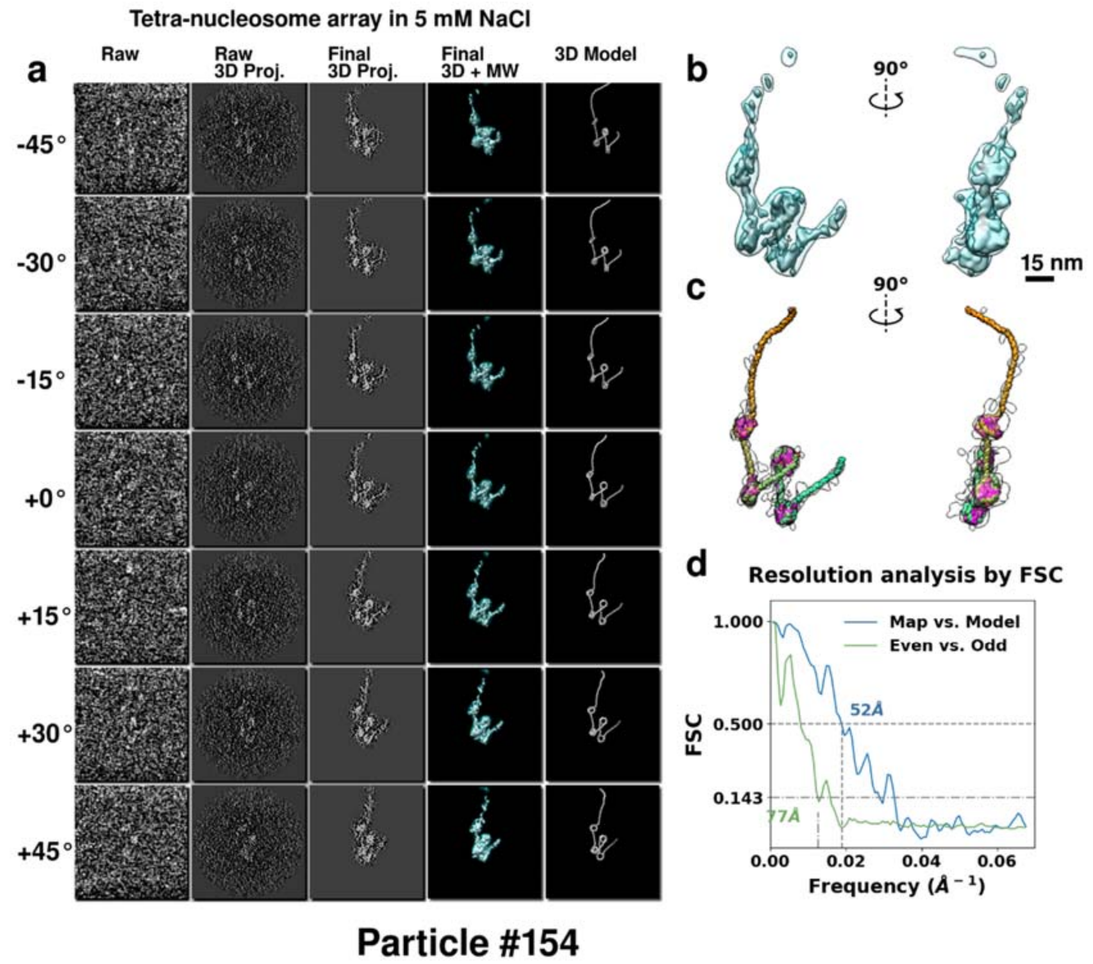

Supplementary Fig. 167. Cryo-ET 3D reconstruction of individual tetra-nucleosome particle (index no. 154) in 5 mM NaCl. a, IPET 3D reconstruction of individual tetra-nucleosome particles. The first column shows seven representative tilt images of an individual particle after CTF correction. Through alignment of the tilt images to a common center for 3D reconstruction via iterative refinement, the second and third columns display the 3D projections of the reconstruction before and after particle-shaped masking, respectively. The fourth column shows the final 3D reconstruction with missing wedge correction, and the fifth column presents the flexibly fitted model at the corresponding tilt angles. **b**, Zoomed-in view of the final 3D density map displayed in orthogonal views, shown at two contour levels. **c**, Superimposition of the high contour level map from (b) onto its flexibly fitted model. **d**, Resolution evaluation of the final 3D density map using two criteria: Fourier shell correlation (FSC) between two-half maps reconstructed from the even and odd index of the tilted series and FSC between the final 3D map and the fitted structure model. The resolution for the former and latter criteria is evaluated at frequencies of 0.5 and 0.143, respectively.

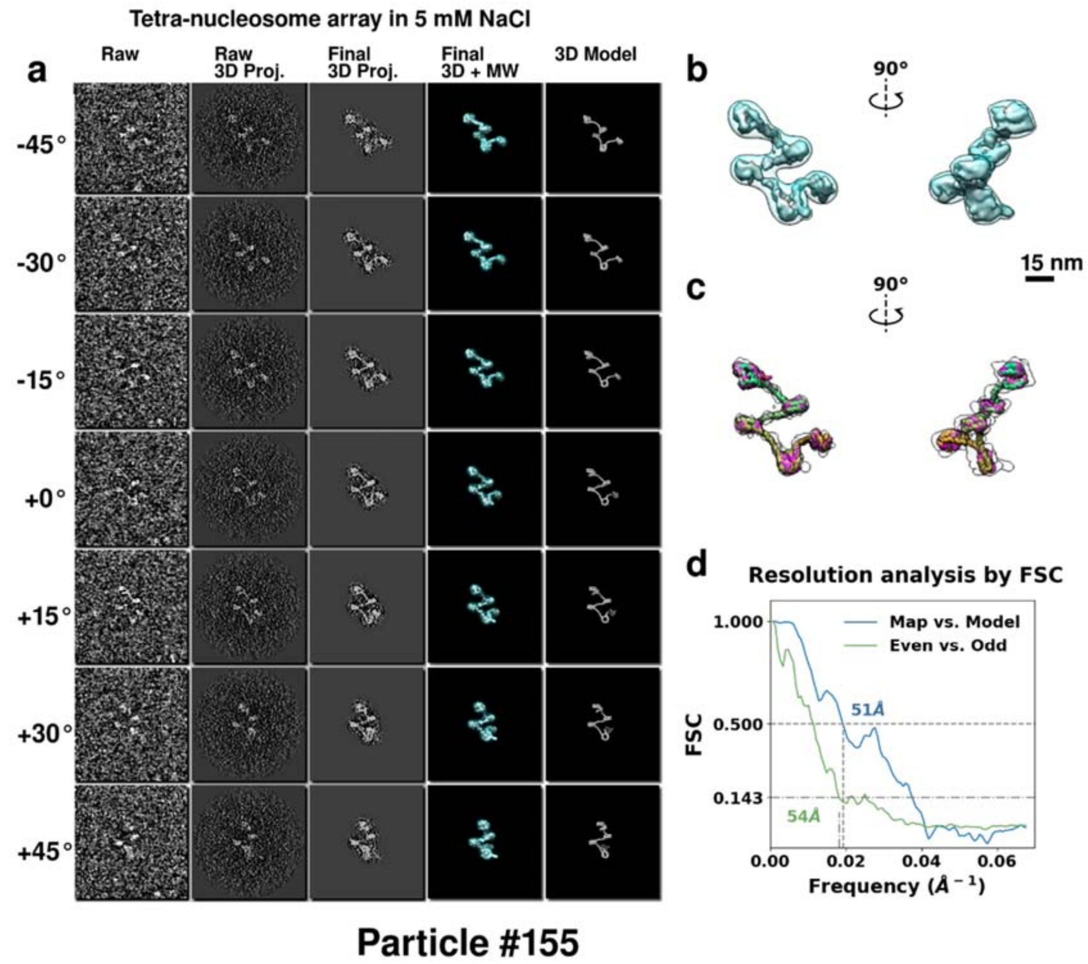

Supplementary Fig. 168. Cryo-ET 3D reconstruction of individual tetra-nucleosome particle (index no. 155) in 5 mM NaCl. a, IPET 3D reconstruction of individual tetra-nucleosome particles. The first column shows seven representative tilt images of an individual particle after CTF correction. Through alignment of the tilt images to a common center for 3D reconstruction via iterative refinement, the second and third columns display the 3D projections of the reconstruction before and after particle-shaped masking, respectively. The fourth column shows the final 3D reconstruction with missing wedge correction, and the fifth column presents the flexibly fitted model at the corresponding tilt angles. **b**, Zoomed-in view of the final 3D density map displayed in orthogonal views, shown at two contour levels. **c**, Superimposition of the high contour level map from (b) onto its flexibly fitted model. **d**, Resolution evaluation of the final 3D density map using two criteria: Fourier shell correlation (FSC) between two-half maps reconstructed from the even and odd index of the tilted series and FSC between the final 3D map and the fitted structure model. The resolution for the former and latter criteria is evaluated at frequencies of 0.5 and 0.143, respectively.

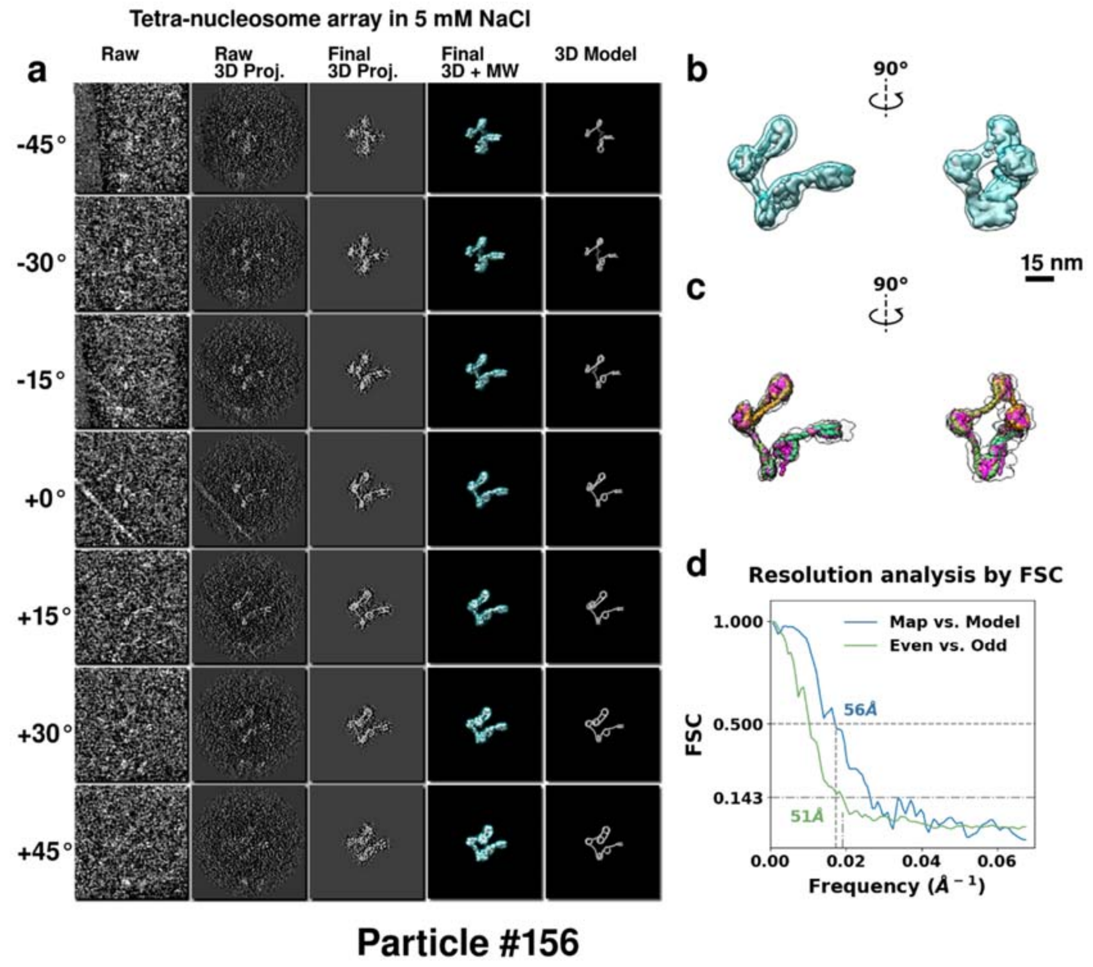

Supplementary Fig. 169. Cryo-ET 3D reconstruction of individual tetra-nucleosome particle (index no. 156) in 5 mM NaCl. a, IPET 3D reconstruction of individual tetra-nucleosome particles. The first column shows seven representative tilt images of an individual particle after CTF correction. Through alignment of the tilt images to a common center for 3D reconstruction via iterative refinement, the second and third columns display the 3D projections of the reconstruction before and after particle-shaped masking, respectively. The fourth column shows the final 3D reconstruction with missing wedge correction, and the fifth column presents the flexibly fitted model at the corresponding tilt angles. **b**, Zoomed-in view of the final 3D density map displayed in orthogonal views, shown at two contour levels. **c**, Superimposition of the high contour level map from (b) onto its flexibly fitted model. **d**, Resolution evaluation of the final 3D density map using two criteria: Fourier shell correlation (FSC) between two-half maps reconstructed from the even and odd index of the tilted series and FSC between the final 3D map and the fitted structure model. The resolution for the former and latter criteria is evaluated at frequencies of 0.5 and 0.143, respectively.

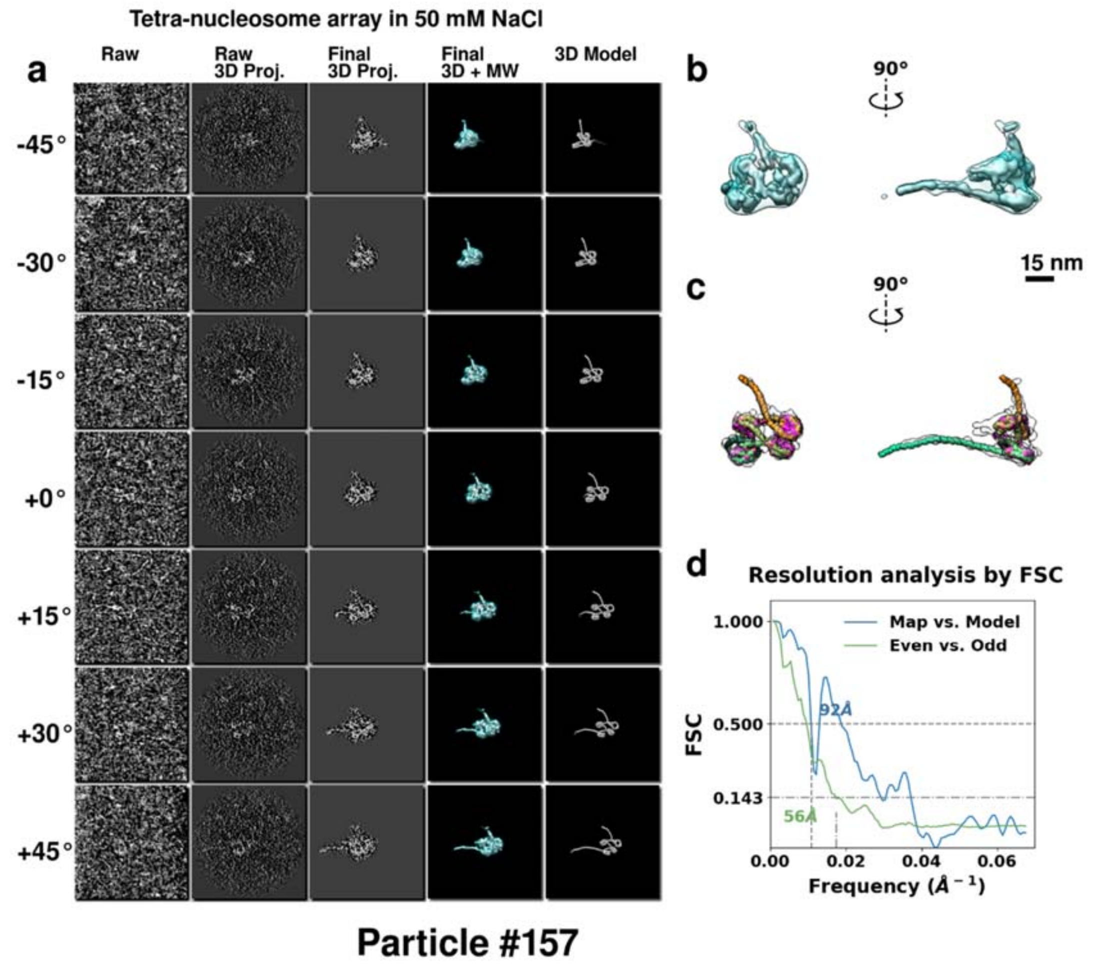

Supplementary Fig. 170. Cryo-ET 3D reconstruction of individual tetra-nucleosome particle (index no. 157 in 50 mM NaCl. a, IPET 3D reconstruction of individual tetra-nucleosome particles. The first column shows seven representative tilt images of an individual particle after CTF correction. Through alignment of the tilt images to a common center for 3D reconstruction via iterative refinement, the second and third columns display the 3D projections of the reconstruction before and after particle-shaped masking, respectively. The fourth column shows the final 3D reconstruction with missing wedge correction, and the fifth column presents the flexibly fitted model at the corresponding tilt angles. **b**, Zoomed-in view of the final 3D density map displayed in orthogonal views, shown at two contour levels. **c**, Superimposition of the high contour level map from (b) onto its flexibly fitted model. **d**, Resolution evaluation of the final 3D density map using two criteria: Fourier shell correlation (FSC) between two-half maps reconstructed from the even and odd index of the tilted series and FSC between the final 3D map and the fitted structure model. The resolution for the former and latter criteria is evaluated at frequencies of 0.5 and 0.143, respectively.

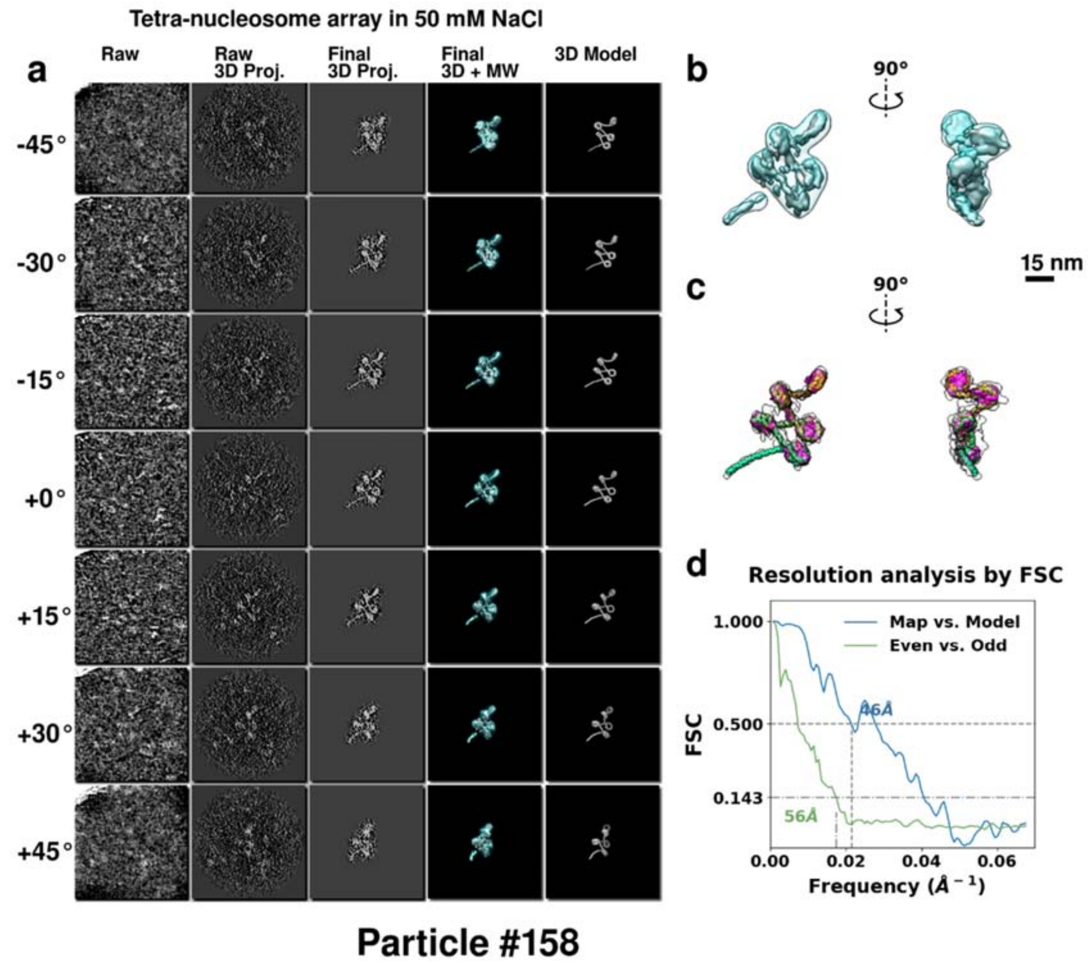

Supplementary Fig. 171. Cryo-ET 3D reconstruction of individual tetra-nucleosome particle (index no. 158 in 50 mM NaCl. a, IPET 3D reconstruction of individual tetra-nucleosome particles. The first column shows seven representative tilt images of an individual particle after CTF correction. Through alignment of the tilt images to a common center for 3D reconstruction via iterative refinement, the second and third columns display the 3D projections of the reconstruction before and after particle-shaped masking, respectively. The fourth column shows the final 3D reconstruction with missing wedge correction, and the fifth column presents the flexibly fitted model at the corresponding tilt angles. **b**, Zoomed-in view of the final 3D density map displayed in orthogonal views, shown at two contour levels. **c**, Superimposition of the high contour level map from (b) onto its flexibly fitted model. **d**, Resolution evaluation of the final 3D density map using two criteria: Fourier shell correlation (FSC) between two-half maps reconstructed from the even and odd index of the tilted series and FSC between the final 3D map and the fitted structure model. The resolution for the former and latter criteria is evaluated at frequencies of 0.5 and 0.143, respectively.

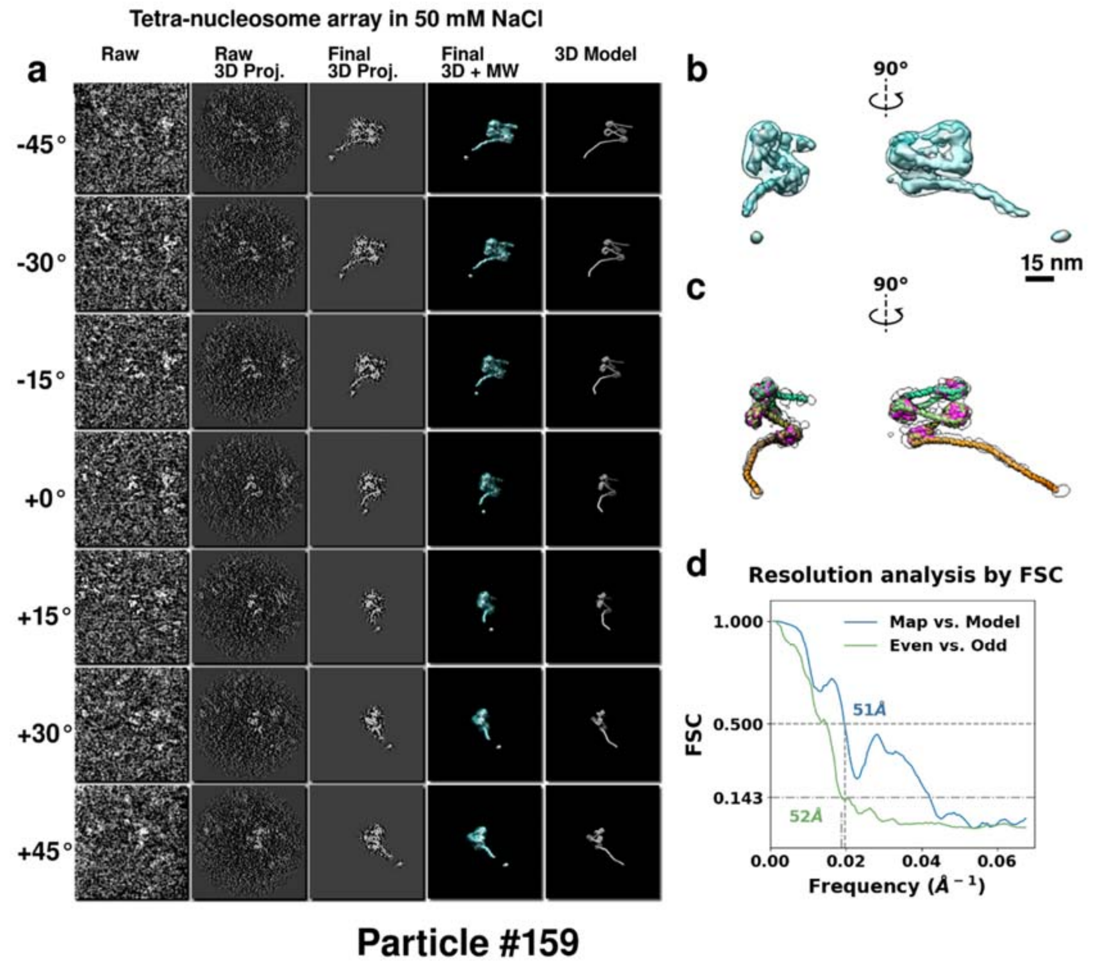

Supplementary Fig. 172. Cryo-ET 3D reconstruction of individual tetra-nucleosome particle (index no. 159 in 50 mM NaCl. a, IPET 3D reconstruction of individual tetra-nucleosome particles. The first column shows seven representative tilt images of an individual particle after CTF correction. Through alignment of the tilt images to a common center for 3D reconstruction via iterative refinement, the second and third columns display the 3D projections of the reconstruction before and after particle-shaped masking, respectively. The fourth column shows the final 3D reconstruction with missing wedge correction, and the fifth column presents the flexibly fitted model at the corresponding tilt angles. **b**, Zoomed-in view of the final 3D density map displayed in orthogonal views, shown at two contour levels. **c**, Superimposition of the high contour level map from (b) onto its flexibly fitted model. **d**, Resolution evaluation of the final 3D density map using two criteria: Fourier shell correlation (FSC) between two-half maps reconstructed from the even and odd index of the tilted series and FSC between the final 3D map and the fitted structure model. The resolution for the former and latter criteria is evaluated at frequencies of 0.5 and 0.143, respectively.

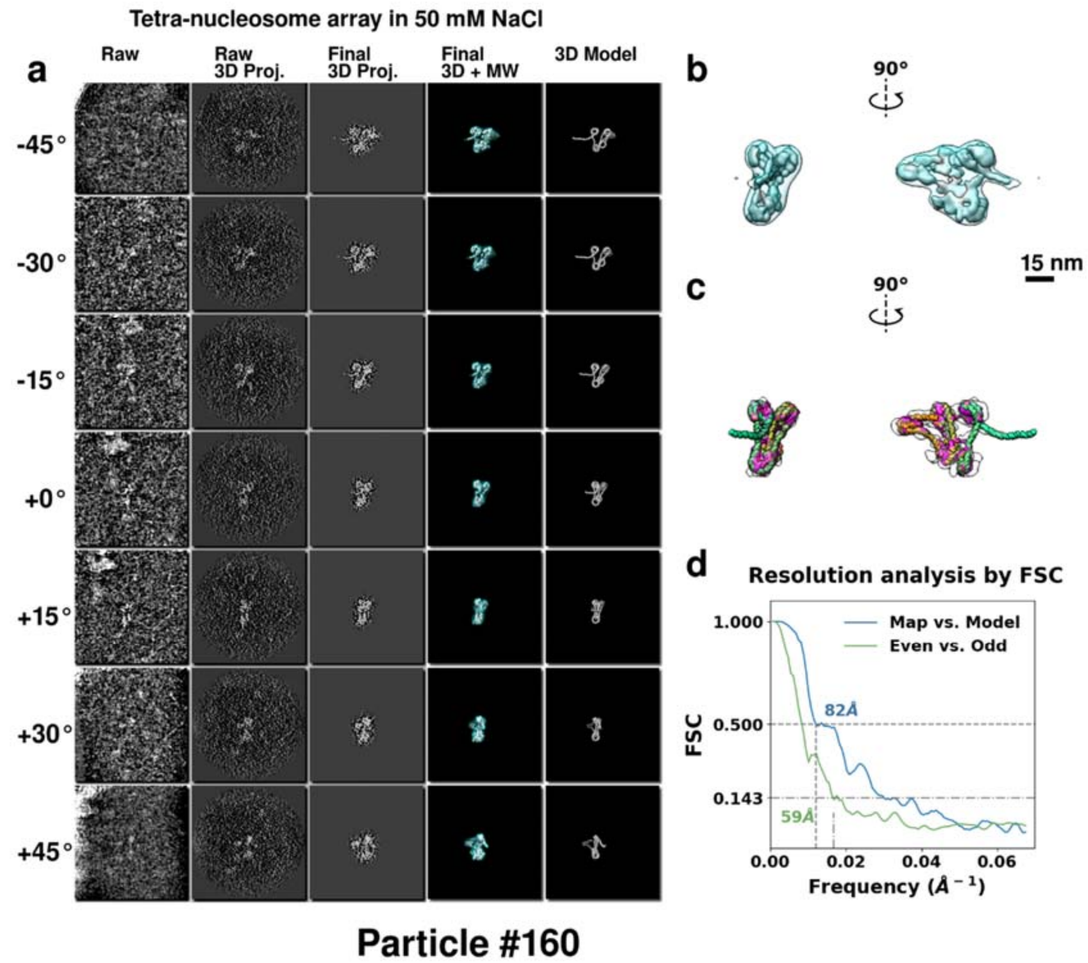

Supplementary Fig. 173. Cryo-ET 3D reconstruction of individual tetra-nucleosome particle (index no. 160 in 50 mM NaCl. a, IPET 3D reconstruction of individual tetra-nucleosome particles. The first column shows seven representative tilt images of an individual particle after CTF correction. Through alignment of the tilt images to a common center for 3D reconstruction via iterative refinement, the second and third columns display the 3D projections of the reconstruction before and after particle-shaped masking, respectively. The fourth column shows the final 3D reconstruction with missing wedge correction, and the fifth column presents the flexibly fitted model at the corresponding tilt angles. **b**, Zoomed-in view of the final 3D density map displayed in orthogonal views, shown at two contour levels. **c**, Superimposition of the high contour level map from (b) onto its flexibly fitted model. **d**, Resolution evaluation of the final 3D density map using two criteria: Fourier shell correlation (FSC) between two-half maps reconstructed from the even and odd index of the tilted series and FSC between the final 3D map and the fitted structure model. The resolution for the former and latter criteria is evaluated at frequencies of 0.5 and 0.143, respectively.

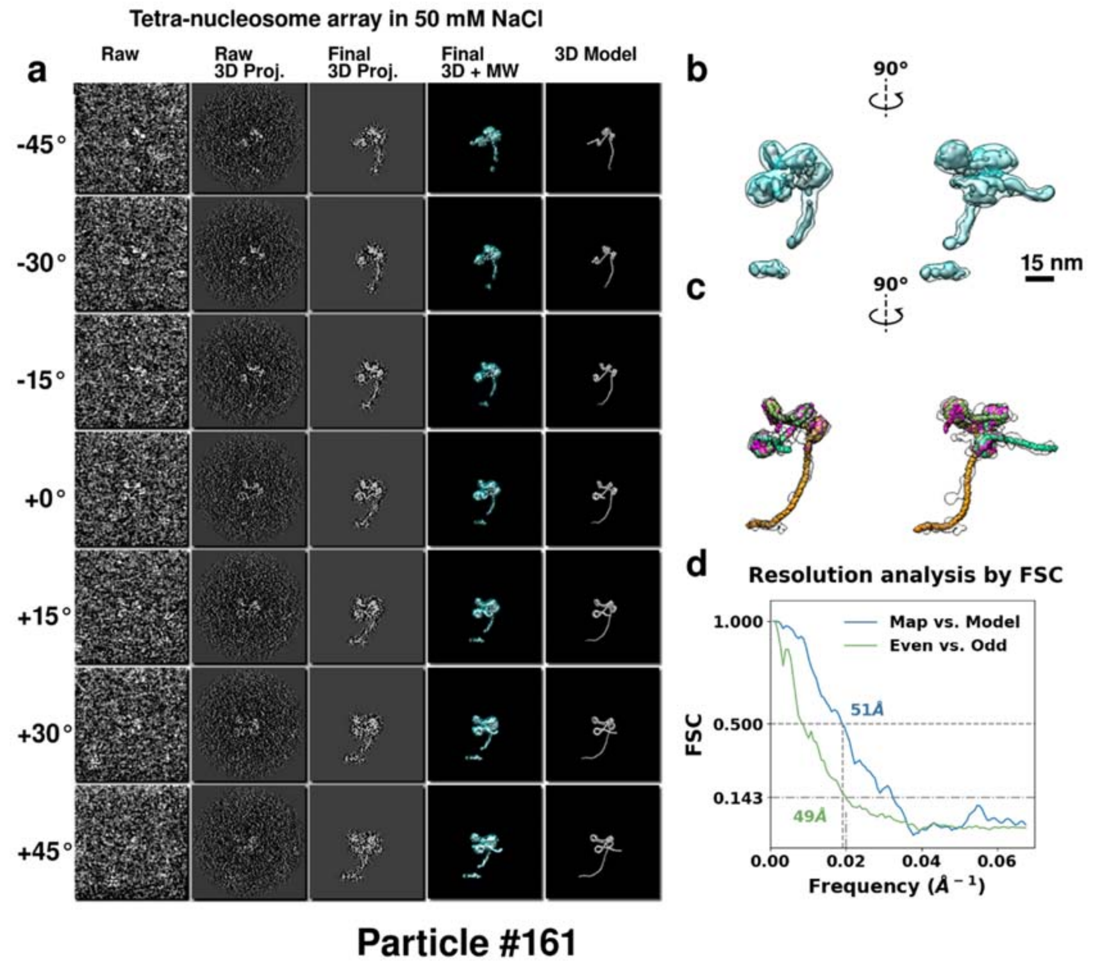

Supplementary Fig. 174. Cryo-ET 3D reconstruction of individual tetra-nucleosome particle (index no. 161 in 50 mM NaCl. a, IPET 3D reconstruction of individual tetra-nucleosome particles. The first column shows seven representative tilt images of an individual particle after CTF correction. Through alignment of the tilt images to a common center for 3D reconstruction via iterative refinement, the second and third columns display the 3D projections of the reconstruction before and after particle-shaped masking, respectively. The fourth column shows the final 3D reconstruction with missing wedge correction, and the fifth column presents the flexibly fitted model at the corresponding tilt angles. **b**, Zoomed-in view of the final 3D density map displayed in orthogonal views, shown at two contour levels. **c**, Superimposition of the high contour level map from (b) onto its flexibly fitted model. **d**, Resolution evaluation of the final 3D density map using two criteria: Fourier shell correlation (FSC) between two-half maps reconstructed from the even and odd index of the tilted series and FSC between the final 3D map and the fitted structure model. The resolution for the former and latter criteria is evaluated at frequencies of 0.5 and 0.143, respectively.

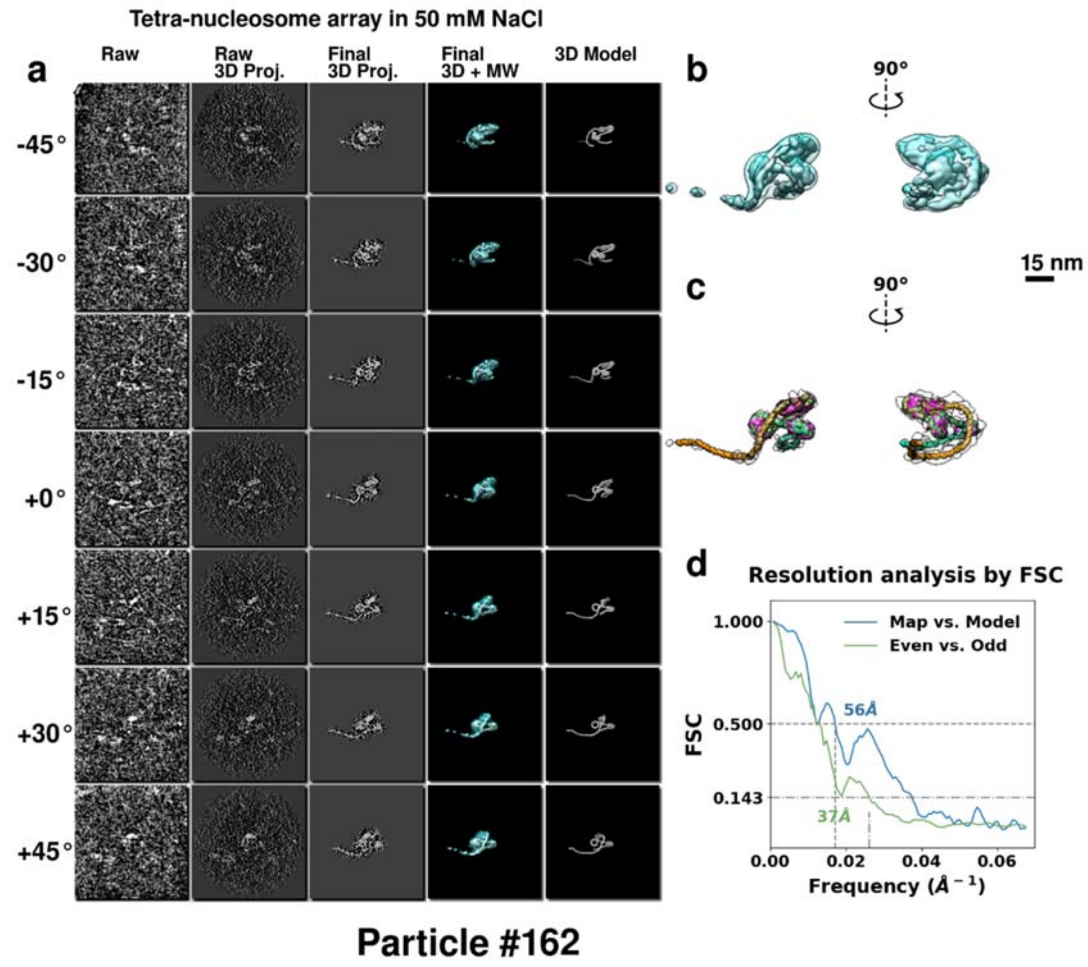

Supplementary Fig. 175. Cryo-ET 3D reconstruction of individual tetra-nucleosome particle (index no. 162 in 50 mM NaCl. a, IPET 3D reconstruction of individual tetra-nucleosome particles. The first column shows seven representative tilt images of an individual particle after CTF correction. Through alignment of the tilt images to a common center for 3D reconstruction via iterative refinement, the second and third columns display the 3D projections of the reconstruction before and after particle-shaped masking, respectively. The fourth column shows the final 3D reconstruction with missing wedge correction, and the fifth column presents the flexibly fitted model at the corresponding tilt angles. **b**, Zoomed-in view of the final 3D density map displayed in orthogonal views, shown at two contour levels. **c**, Superimposition of the high contour level map from (b) onto its flexibly fitted model. **d**, Resolution evaluation of the final 3D density map using two criteria: Fourier shell correlation (FSC) between two-half maps reconstructed from the even and odd index of the tilted series and FSC between the final 3D map and the fitted structure model. The resolution for the former and latter criteria is evaluated at frequencies of 0.5 and 0.143, respectively.

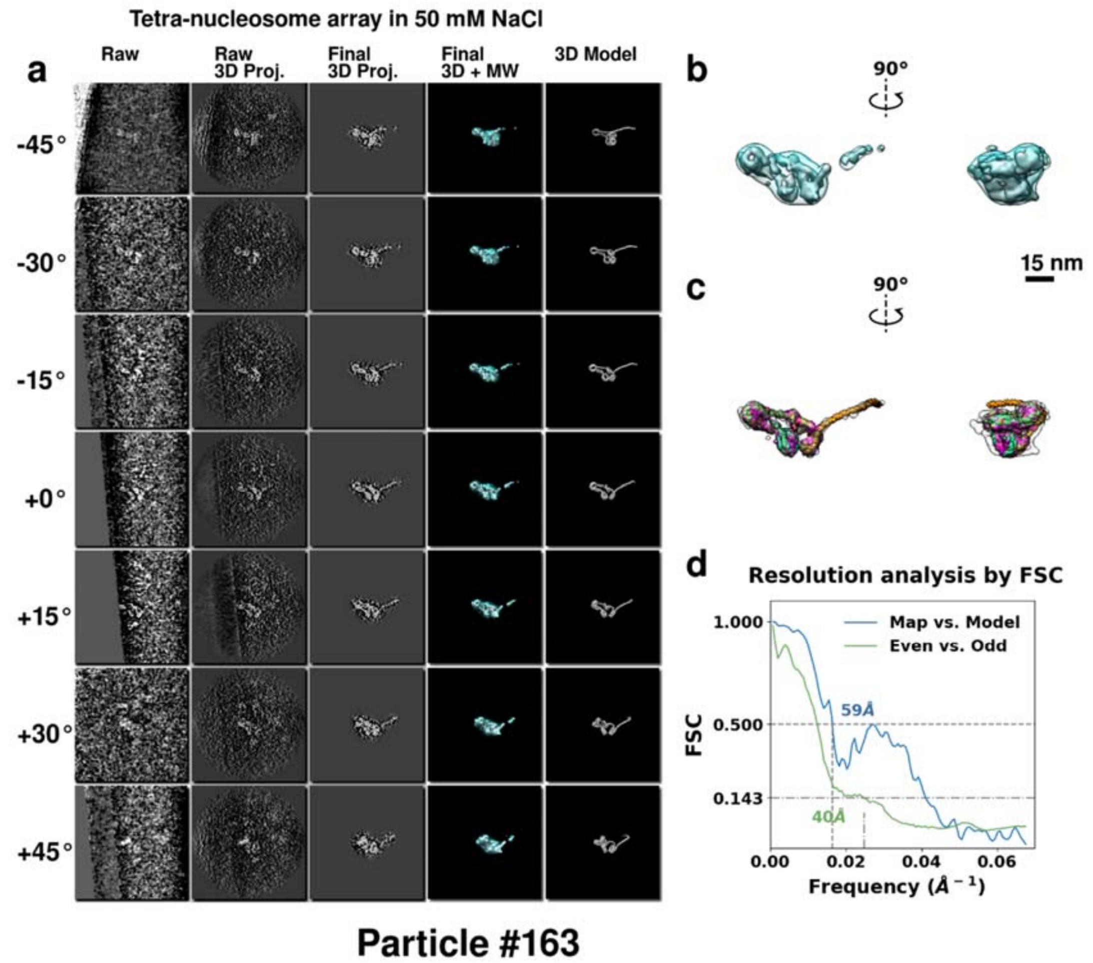

Supplementary Fig. 176. Cryo-ET 3D reconstruction of individual tetra-nucleosome particle (index no. 163 in 50 mM NaCl. a, IPET 3D reconstruction of individual tetra-nucleosome particles. The first column shows seven representative tilt images of an individual particle after CTF correction. Through alignment of the tilt images to a common center for 3D reconstruction via iterative refinement, the second and third columns display the 3D projections of the reconstruction before and after particle-shaped masking, respectively. The fourth column shows the final 3D reconstruction with missing wedge correction, and the fifth column presents the flexibly fitted model at the corresponding tilt angles. **b**, Zoomed-in view of the final 3D density map displayed in orthogonal views, shown at two contour levels. **c**, Superimposition of the high contour level map from (b) onto its flexibly fitted model. **d**, Resolution evaluation of the final 3D density map using two criteria: Fourier shell correlation (FSC) between two-half maps reconstructed from the even and odd index of the tilted series and FSC between the final 3D map and the fitted structure model. The resolution for the former and latter criteria is evaluated at frequencies of 0.5 and 0.143, respectively.

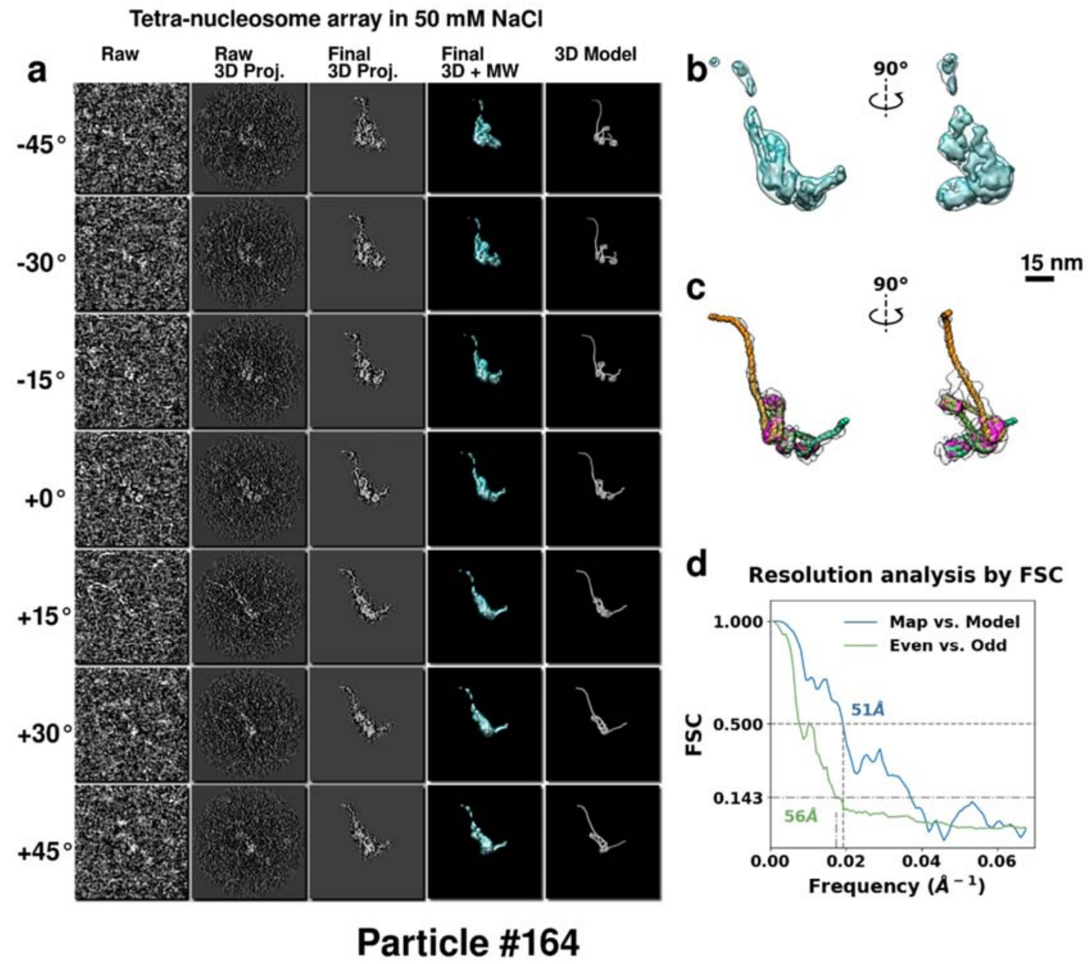

Supplementary Fig. 177. Cryo-ET 3D reconstruction of individual tetra-nucleosome particle (index no. 164 in 50 mM NaCl. a, IPET 3D reconstruction of individual tetra-nucleosome particles. The first column shows seven representative tilt images of an individual particle after CTF correction. Through alignment of the tilt images to a common center for 3D reconstruction via iterative refinement, the second and third columns display the 3D projections of the reconstruction before and after particle-shaped masking, respectively. The fourth column shows the final 3D reconstruction with missing wedge correction, and the fifth column presents the flexibly fitted model at the corresponding tilt angles. **b**, Zoomed-in view of the final 3D density map displayed in orthogonal views, shown at two contour levels. **c**, Superimposition of the high contour level map from (b) onto its flexibly fitted model. **d**, Resolution evaluation of the final 3D density map using two criteria: Fourier shell correlation (FSC) between two-half maps reconstructed from the even and odd index of the tilted series and FSC between the final 3D map and the fitted structure model. The resolution for the former and latter criteria is evaluated at frequencies of 0.5 and 0.143, respectively.

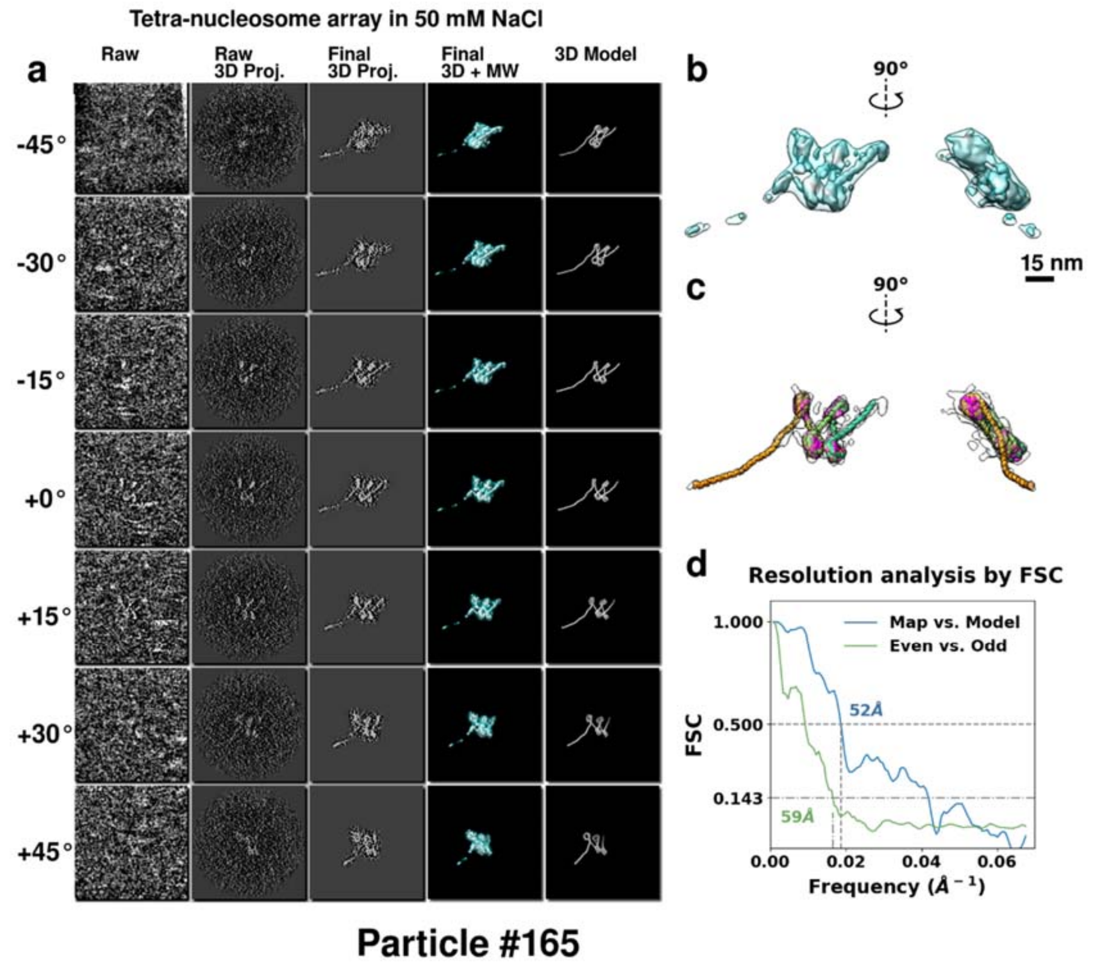

Supplementary Fig. 178. Cryo-ET 3D reconstruction of individual tetra-nucleosome particle (index no. 165 in 50 mM NaCl. a, IPET 3D reconstruction of individual tetra-nucleosome particles. The first column shows seven representative tilt images of an individual particle after CTF correction. Through alignment of the tilt images to a common center for 3D reconstruction via iterative refinement, the second and third columns display the 3D projections of the reconstruction before and after particle-shaped masking, respectively. The fourth column shows the final 3D reconstruction with missing wedge correction, and the fifth column presents the flexibly fitted model at the corresponding tilt angles. **b**, Zoomed-in view of the final 3D density map displayed in orthogonal views, shown at two contour levels. **c**, Superimposition of the high contour level map from (b) onto its flexibly fitted model. **d**, Resolution evaluation of the final 3D density map using two criteria: Fourier shell correlation (FSC) between two-half maps reconstructed from the even and odd index of the tilted series and FSC between the final 3D map and the fitted structure model. The resolution for the former and latter criteria is evaluated at frequencies of 0.5 and 0.143, respectively.

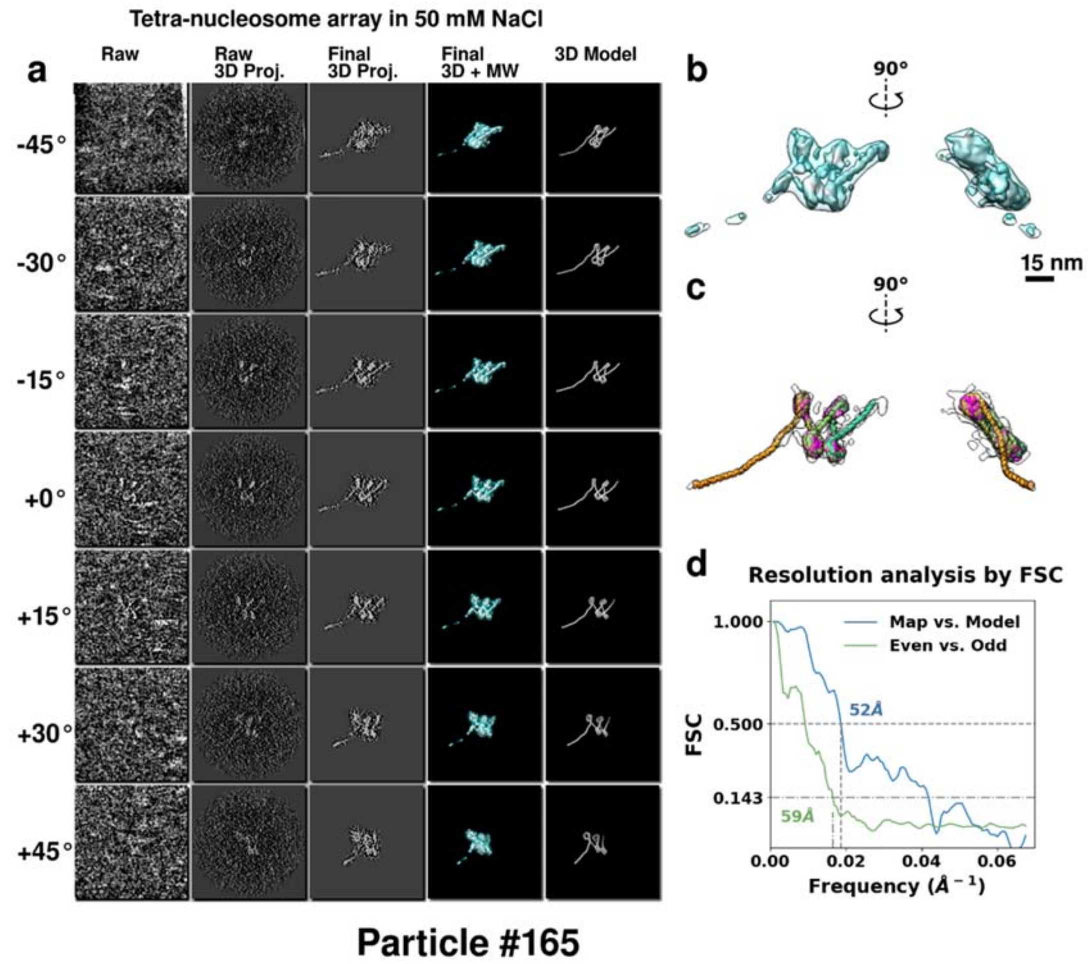

Supplementary Fig. 179. Cryo-ET 3D reconstruction of individual tetra-nucleosome particle (index no. 166 in 50 mM NaCl. a, IPET 3D reconstruction of individual tetra-nucleosome particles. The first column shows seven representative tilt images of an individual particle after CTF correction. Through alignment of the tilt images to a common center for 3D reconstruction via iterative refinement, the second and third columns display the 3D projections of the reconstruction before and after particle-shaped masking, respectively. The fourth column shows the final 3D reconstruction with missing wedge correction, and the fifth column presents the flexibly fitted model at the corresponding tilt angles. **b**, Zoomed-in view of the final 3D density map displayed in orthogonal views, shown at two contour levels. **c**, Superimposition of the high contour level map from (b) onto its flexibly fitted model. **d**, Resolution evaluation of the final 3D density map using two criteria: Fourier shell correlation (FSC) between two-half maps reconstructed from the even and odd index of the tilted series and FSC between the final 3D map and the fitted structure model. The resolution for the former and latter criteria is evaluated at frequencies of 0.5 and 0.143, respectively.

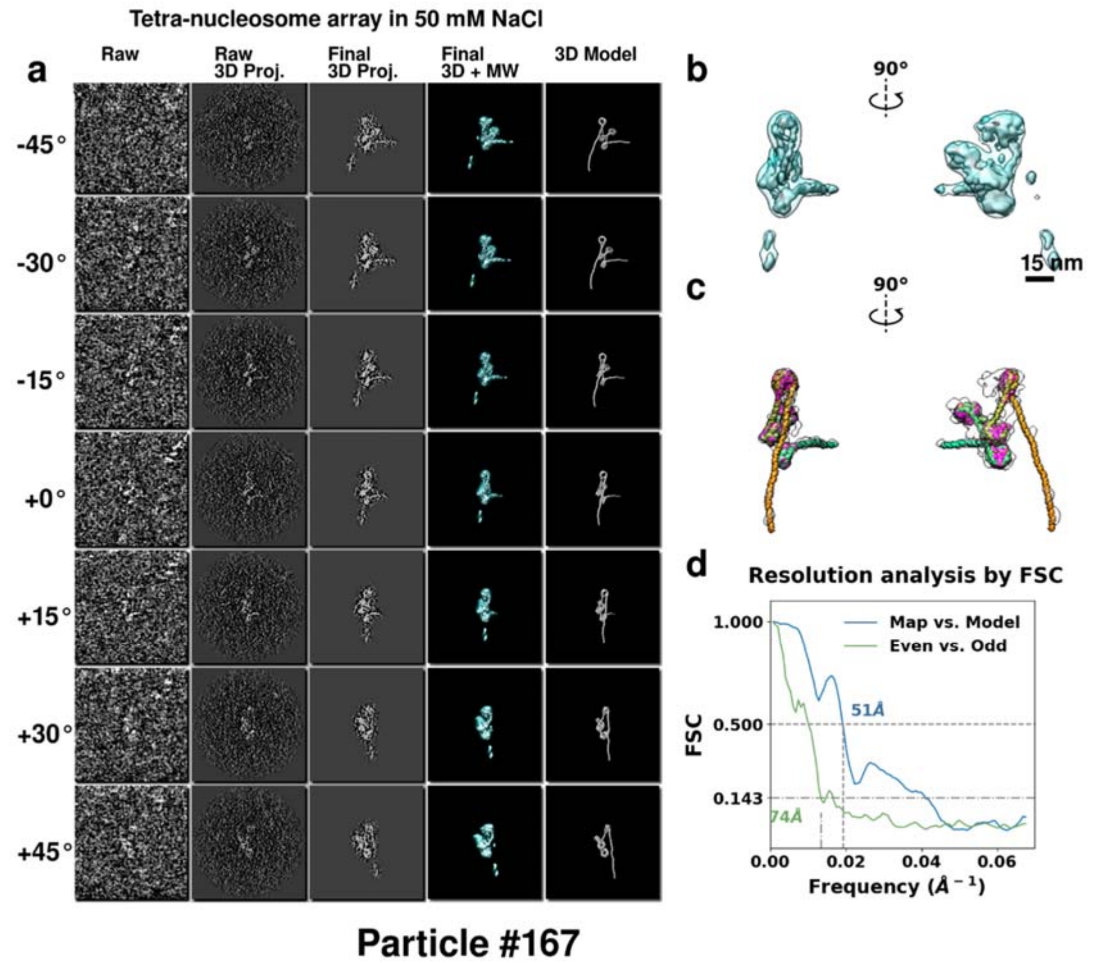

Supplementary Fig. 180. Cryo-ET 3D reconstruction of individual tetra-nucleosome particle (index no. 167 in 50 mM NaCl. a, IPET 3D reconstruction of individual tetra-nucleosome particles. The first column shows seven representative tilt images of an individual particle after CTF correction. Through alignment of the tilt images to a common center for 3D reconstruction via iterative refinement, the second and third columns display the 3D projections of the reconstruction before and after particle-shaped masking, respectively. The fourth column shows the final 3D reconstruction with missing wedge correction, and the fifth column presents the flexibly fitted model at the corresponding tilt angles. **b**, Zoomed-in view of the final 3D density map displayed in orthogonal views, shown at two contour levels. **c**, Superimposition of the high contour level map from (b) onto its flexibly fitted model. **d**, Resolution evaluation of the final 3D density map using two criteria: Fourier shell correlation (FSC) between two-half maps reconstructed from the even and odd index of the tilted series and FSC between the final 3D map and the fitted structure model. The resolution for the former and latter criteria is evaluated at frequencies of 0.5 and 0.143, respectively.

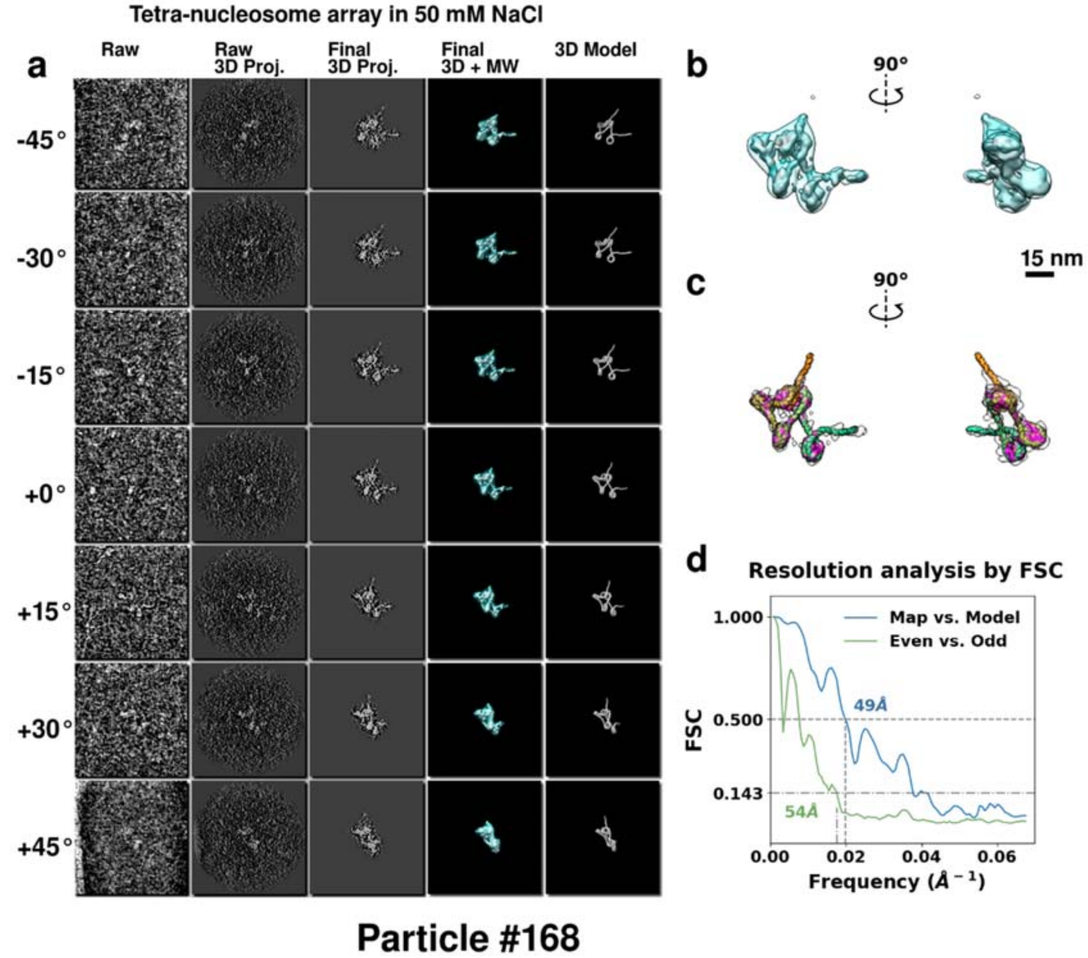

Supplementary Fig. 181. Cryo-ET 3D reconstruction of individual tetra-nucleosome particle (index no. 168 in 50 mM NaCl. a, IPET 3D reconstruction of individual tetra-nucleosome particles. The first column shows seven representative tilt images of an individual particle after CTF correction. Through alignment of the tilt images to a common center for 3D reconstruction via iterative refinement, the second and third columns display the 3D projections of the reconstruction before and after particle-shaped masking, respectively. The fourth column shows the final 3D reconstruction with missing wedge correction, and the fifth column presents the flexibly fitted model at the corresponding tilt angles. **b**, Zoomed-in view of the final 3D density map displayed in orthogonal views, shown at two contour levels. **c**, Superimposition of the high contour level map from (b) onto its flexibly fitted model. **d**, Resolution evaluation of the final 3D density map using two criteria: Fourier shell correlation (FSC) between two-half maps reconstructed from the even and odd index of the tilted series and FSC between the final 3D map and the fitted structure model. The resolution for the former and latter criteria is evaluated at frequencies of 0.5 and 0.143, respectively.

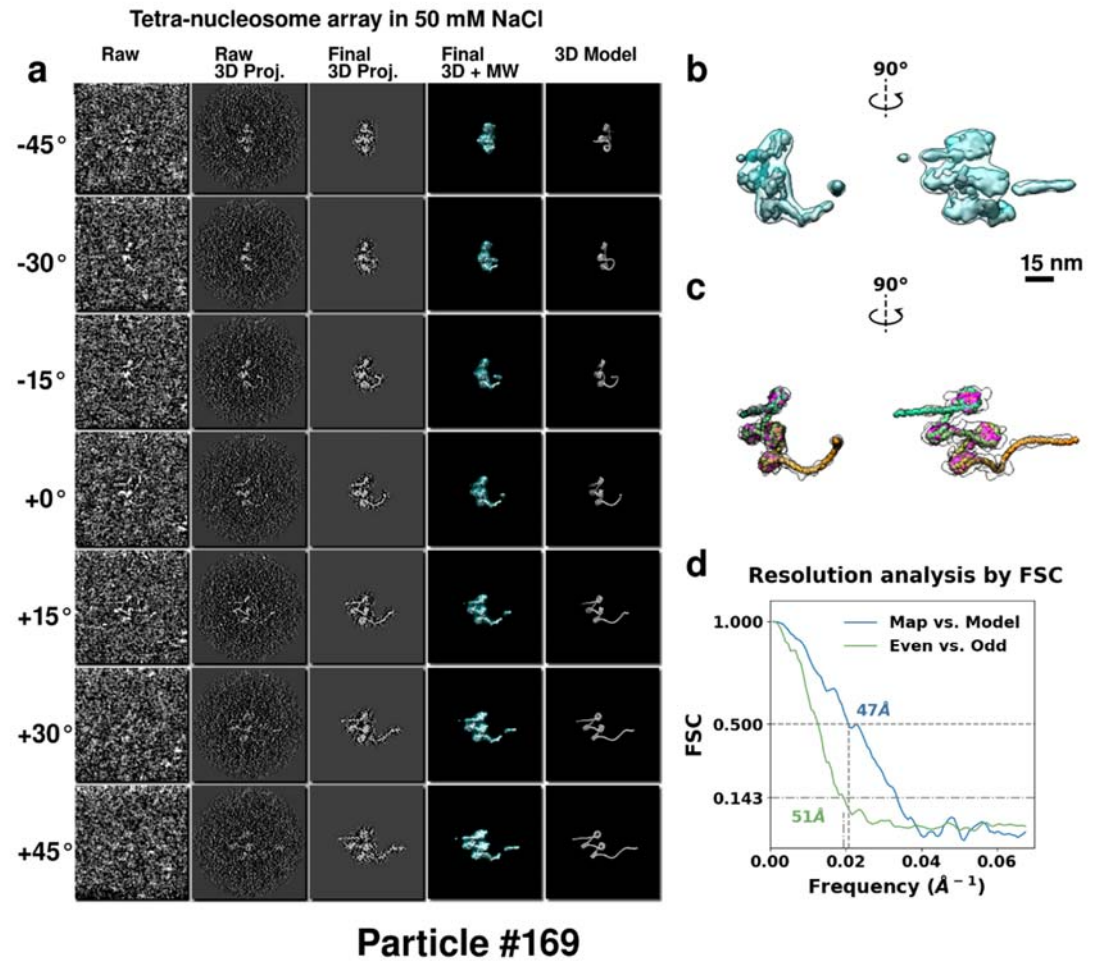

Supplementary Fig. 182. Cryo-ET 3D reconstruction of individual tetra-nucleosome particle (index no. 169 in 50 mM NaCl. a, IPET 3D reconstruction of individual tetra-nucleosome particles. The first column shows seven representative tilt images of an individual particle after CTF correction. Through alignment of the tilt images to a common center for 3D reconstruction via iterative refinement, the second and third columns display the 3D projections of the reconstruction before and after particle-shaped masking, respectively. The fourth column shows the final 3D reconstruction with missing wedge correction, and the fifth column presents the flexibly fitted model at the corresponding tilt angles. **b**, Zoomed-in view of the final 3D density map displayed in orthogonal views, shown at two contour levels. **c**, Superimposition of the high contour level map from (b) onto its flexibly fitted model. **d**, Resolution evaluation of the final 3D density map using two criteria: Fourier shell correlation (FSC) between two-half maps reconstructed from the even and odd index of the tilted series and FSC between the final 3D map and the fitted structure model. The resolution for the former and latter criteria is evaluated at frequencies of 0.5 and 0.143, respectively.

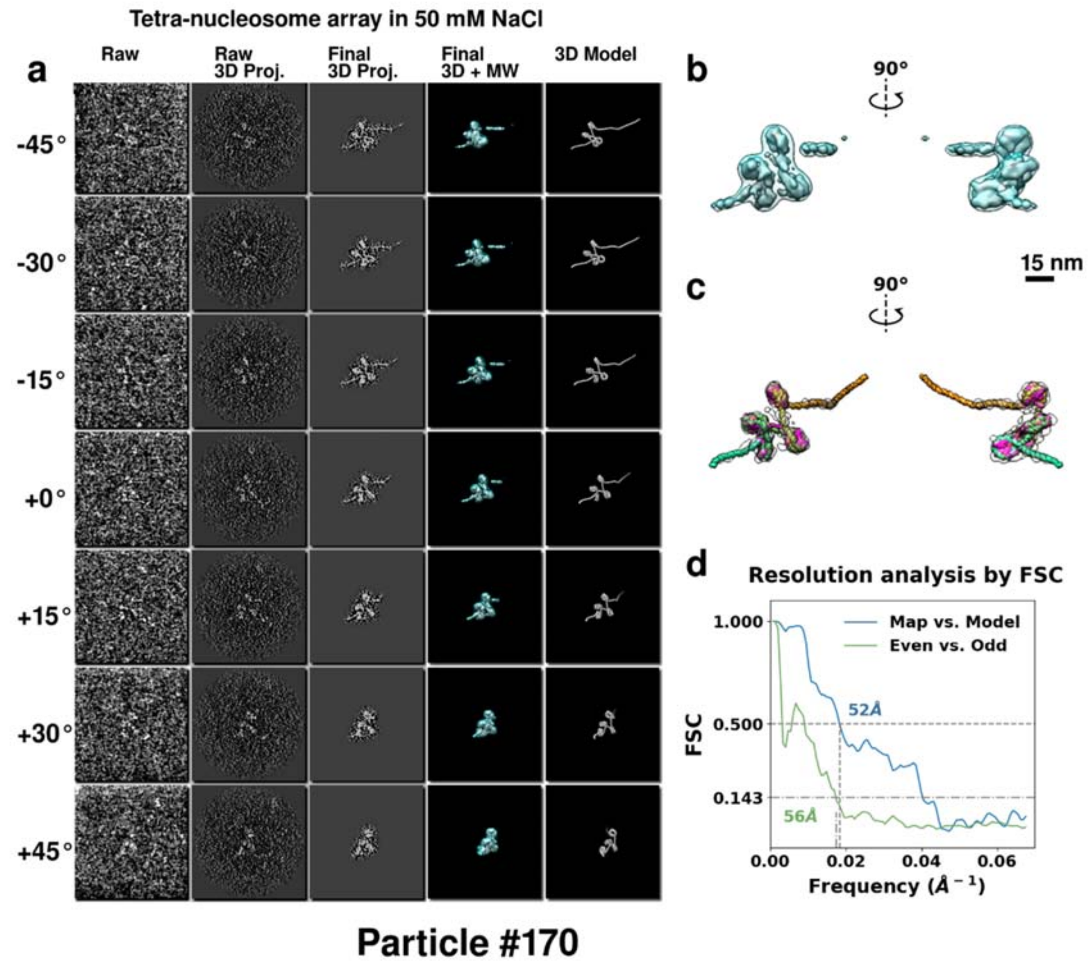

Supplementary Fig. 183. Cryo-ET 3D reconstruction of individual tetra-nucleosome particle (index no. 170 in 50 mM NaCl. a, IPET 3D reconstruction of individual tetra-nucleosome particles. The first column shows seven representative tilt images of an individual particle after CTF correction. Through alignment of the tilt images to a common center for 3D reconstruction via iterative refinement, the second and third columns display the 3D projections of the reconstruction before and after particle-shaped masking, respectively. The fourth column shows the final 3D reconstruction with missing wedge correction, and the fifth column presents the flexibly fitted model at the corresponding tilt angles. **b**, Zoomed-in view of the final 3D density map displayed in orthogonal views, shown at two contour levels. **c**, Superimposition of the high contour level map from (b) onto its flexibly fitted model. **d**, Resolution evaluation of the final 3D density map using two criteria: Fourier shell correlation (FSC) between two-half maps reconstructed from the even and odd index of the tilted series and FSC between the final 3D map and the fitted structure model. The resolution for the former and latter criteria is evaluated at frequencies of 0.5 and 0.143, respectively.

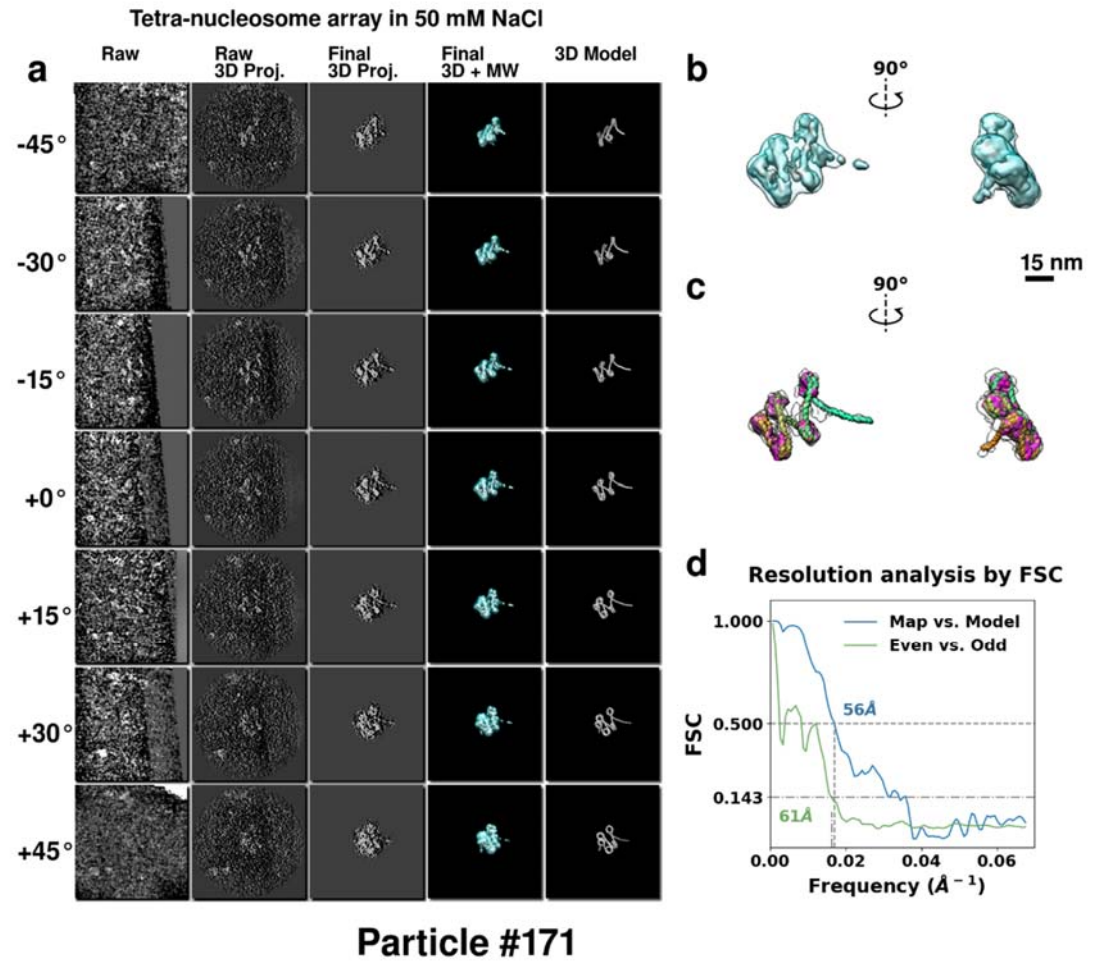

Supplementary Fig. 184. Cryo-ET 3D reconstruction of individual tetra-nucleosome particle (index no. 171 in 50 mM NaCl. a, IPET 3D reconstruction of individual tetra-nucleosome particles. The first column shows seven representative tilt images of an individual particle after CTF correction. Through alignment of the tilt images to a common center for 3D reconstruction via iterative refinement, the second and third columns display the 3D projections of the reconstruction before and after particle-shaped masking, respectively. The fourth column shows the final 3D reconstruction with missing wedge correction, and the fifth column presents the flexibly fitted model at the corresponding tilt angles. **b**, Zoomed-in view of the final 3D density map displayed in orthogonal views, shown at two contour levels. **c**, Superimposition of the high contour level map from (b) onto its flexibly fitted model. **d**, Resolution evaluation of the final 3D density map using two criteria: Fourier shell correlation (FSC) between two-half maps reconstructed from the even and odd index of the tilted series and FSC between the final 3D map and the fitted structure model. The resolution for the former and latter criteria is evaluated at frequencies of 0.5 and 0.143, respectively.

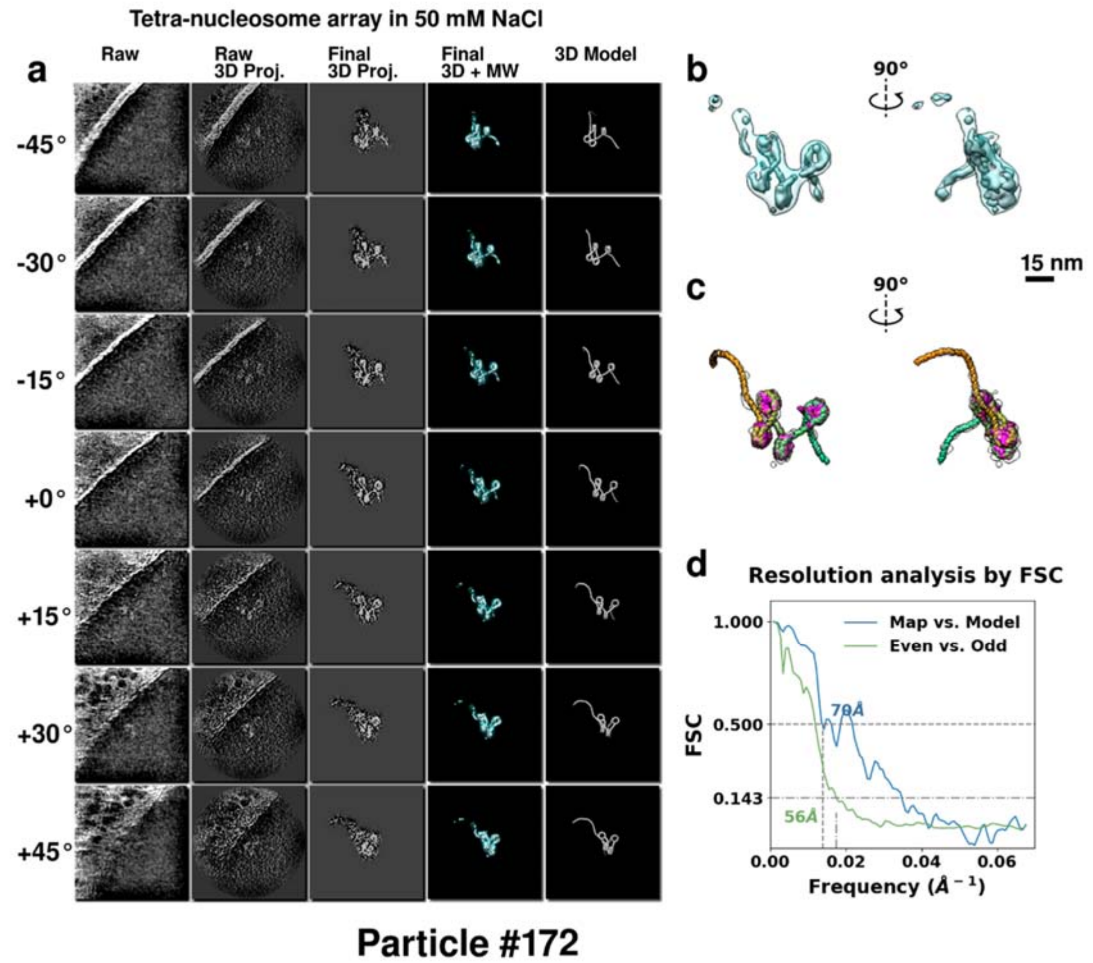

Supplementary Fig. 185. Cryo-ET 3D reconstruction of individual tetra-nucleosome particle (index no. 172 in 50 mM NaCl. a, IPET 3D reconstruction of individual tetra-nucleosome particles. The first column shows seven representative tilt images of an individual particle after CTF correction. Through alignment of the tilt images to a common center for 3D reconstruction via iterative refinement, the second and third columns display the 3D projections of the reconstruction before and after particle-shaped masking, respectively. The fourth column shows the final 3D reconstruction with missing wedge correction, and the fifth column presents the flexibly fitted model at the corresponding tilt angles. **b**, Zoomed-in view of the final 3D density map displayed in orthogonal views, shown at two contour levels. **c**, Superimposition of the high contour level map from (b) onto its flexibly fitted model. **d**, Resolution evaluation of the final 3D density map using two criteria: Fourier shell correlation (FSC) between two-half maps reconstructed from the even and odd index of the tilted series and FSC between the final 3D map and the fitted structure model. The resolution for the former and latter criteria is evaluated at frequencies of 0.5 and 0.143, respectively.

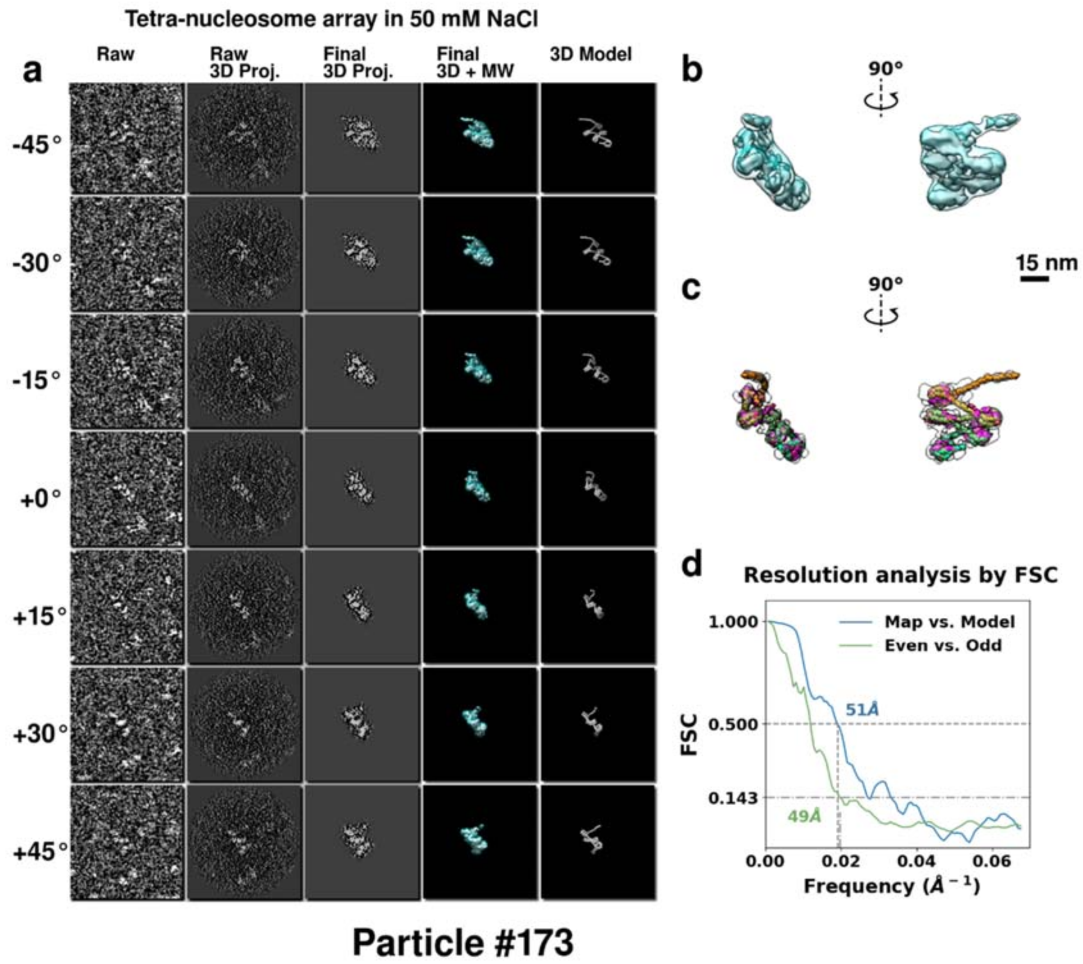

Supplementary Fig. 186. Cryo-ET 3D reconstruction of individual tetra-nucleosome particle (index no. 173 in 50 mM NaCl. a, IPET 3D reconstruction of individual tetra-nucleosome particles. The first column shows seven representative tilt images of an individual particle after CTF correction. Through alignment of the tilt images to a common center for 3D reconstruction via iterative refinement, the second and third columns display the 3D projections of the reconstruction before and after particle-shaped masking, respectively. The fourth column shows the final 3D reconstruction with missing wedge correction, and the fifth column presents the flexibly fitted model at the corresponding tilt angles. **b**, Zoomed-in view of the final 3D density map displayed in orthogonal views, shown at two contour levels. **c**, Superimposition of the high contour level map from (b) onto its flexibly fitted model. **d**, Resolution evaluation of the final 3D density map using two criteria: Fourier shell correlation (FSC) between two-half maps reconstructed from the even and odd index of the tilted series and FSC between the final 3D map and the fitted structure model. The resolution for the former and latter criteria is evaluated at frequencies of 0.5 and 0.143, respectively.

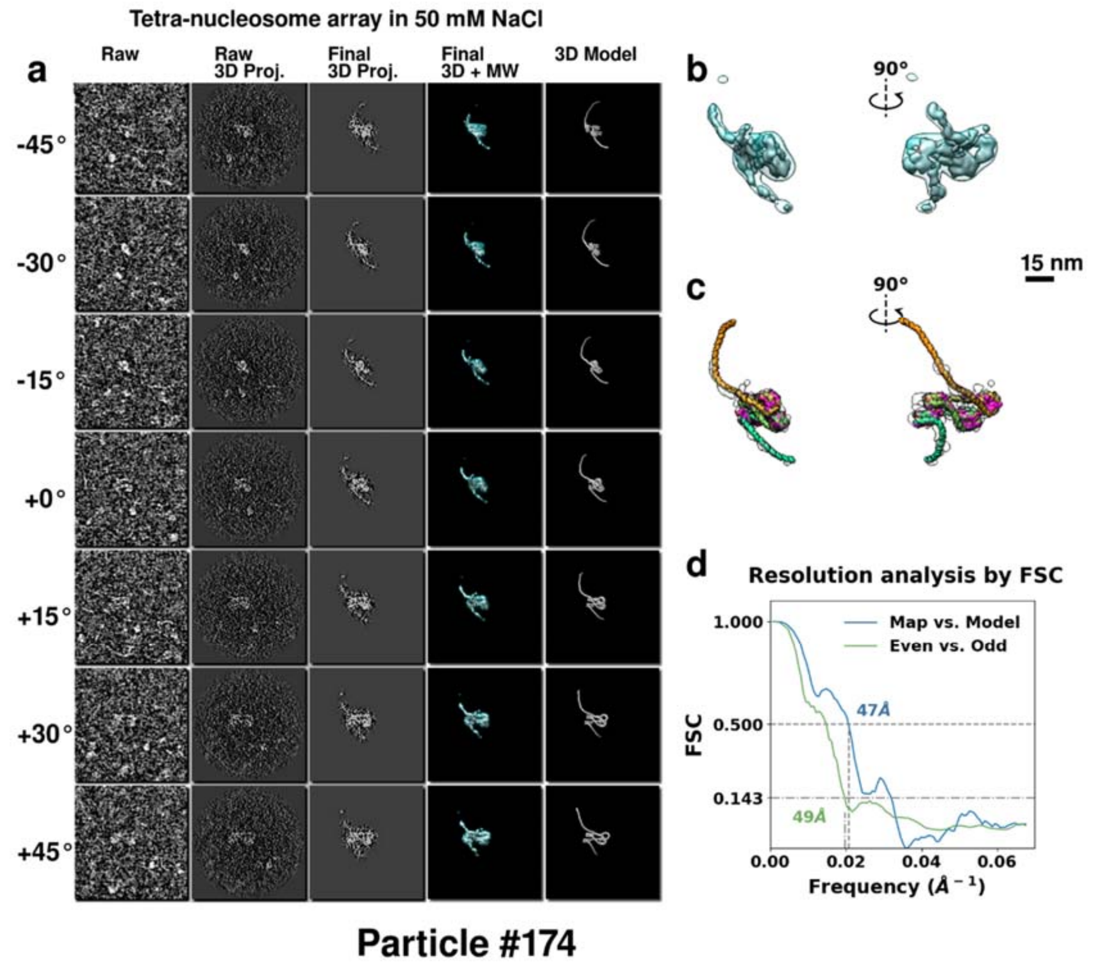

Supplementary Fig. 187. Cryo-ET 3D reconstruction of individual tetra-nucleosome particle (index no. 174 in 50 mM NaCl. a, IPET 3D reconstruction of individual tetra-nucleosome particles. The first column shows seven representative tilt images of an individual particle after CTF correction. Through alignment of the tilt images to a common center for 3D reconstruction via iterative refinement, the second and third columns display the 3D projections of the reconstruction before and after particle-shaped masking, respectively. The fourth column shows the final 3D reconstruction with missing wedge correction, and the fifth column presents the flexibly fitted model at the corresponding tilt angles. **b**, Zoomed-in view of the final 3D density map displayed in orthogonal views, shown at two contour levels. **c**, Superimposition of the high contour level map from (b) onto its flexibly fitted model. **d**, Resolution evaluation of the final 3D density map using two criteria: Fourier shell correlation (FSC) between two-half maps reconstructed from the even and odd index of the tilted series and FSC between the final 3D map and the fitted structure model. The resolution for the former and latter criteria is evaluated at frequencies of 0.5 and 0.143, respectively.

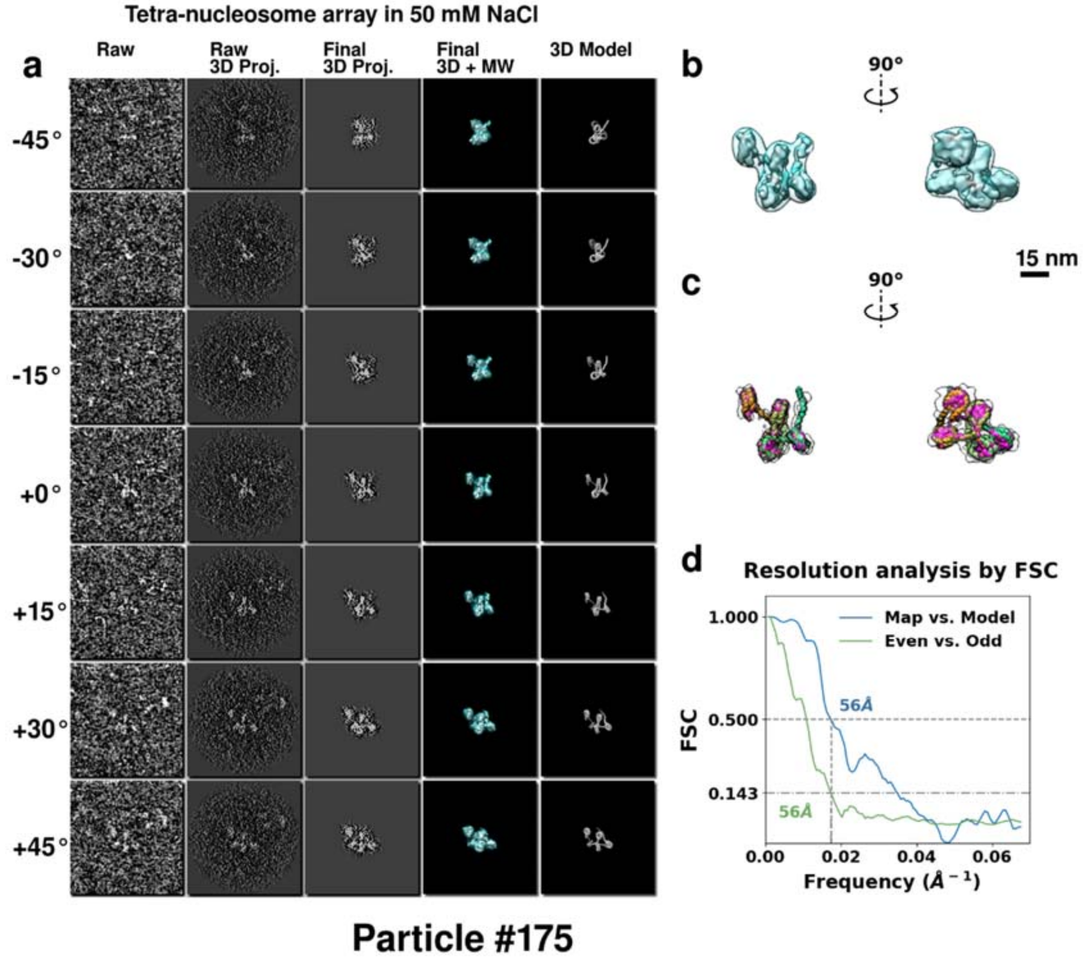

Supplementary Fig. 188. Cryo-ET 3D reconstruction of individual tetra-nucleosome particle (index no. 175 in 50 mM NaCl. a, IPET 3D reconstruction of individual tetra-nucleosome particles. The first column shows seven representative tilt images of an individual particle after CTF correction. Through alignment of the tilt images to a common center for 3D reconstruction via iterative refinement, the second and third columns display the 3D projections of the reconstruction before and after particle-shaped masking, respectively. The fourth column shows the final 3D reconstruction with missing wedge correction, and the fifth column presents the flexibly fitted model at the corresponding tilt angles. **b**, Zoomed-in view of the final 3D density map displayed in orthogonal views, shown at two contour levels. **c**, Superimposition of the high contour level map from (b) onto its flexibly fitted model. **d**, Resolution evaluation of the final 3D density map using two criteria: Fourier shell correlation (FSC) between two-half maps reconstructed from the even and odd index of the tilted series and FSC between the final 3D map and the fitted structure model. The resolution for the former and latter criteria is evaluated at frequencies of 0.5 and 0.143, respectively.

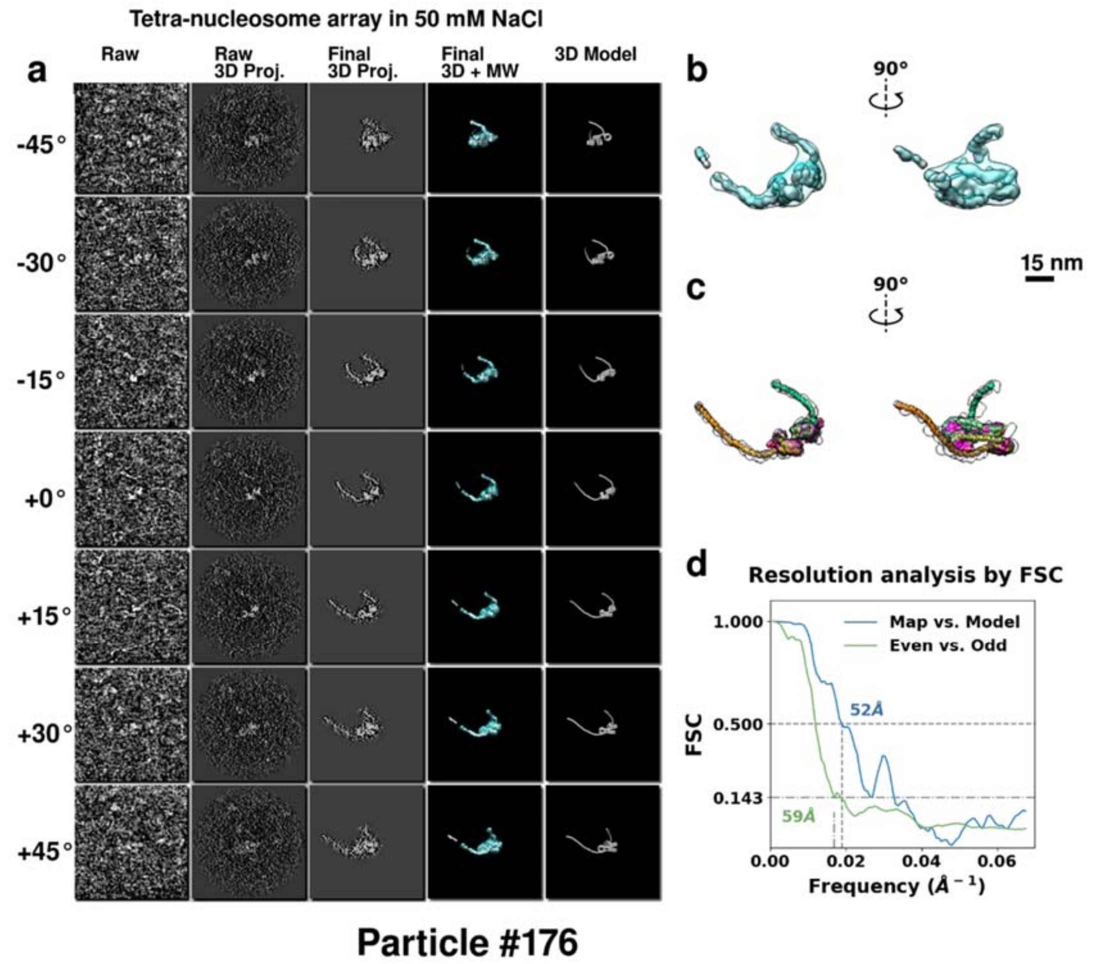

Supplementary Fig. 189. Cryo-ET 3D reconstruction of individual tetra-nucleosome particle (index no. 176 in 50 mM NaCl. a, IPET 3D reconstruction of individual tetra-nucleosome particles. The first column shows seven representative tilt images of an individual particle after CTF correction. Through alignment of the tilt images to a common center for 3D reconstruction via iterative refinement, the second and third columns display the 3D projections of the reconstruction before and after particle-shaped masking, respectively. The fourth column shows the final 3D reconstruction with missing wedge correction, and the fifth column presents the flexibly fitted model at the corresponding tilt angles. **b**, Zoomed-in view of the final 3D density map displayed in orthogonal views, shown at two contour levels. **c**, Superimposition of the high contour level map from (b) onto its flexibly fitted model. **d**, Resolution evaluation of the final 3D density map using two criteria: Fourier shell correlation (FSC) between two-half maps reconstructed from the even and odd index of the tilted series and FSC between the final 3D map and the fitted structure model. The resolution for the former and latter criteria is evaluated at frequencies of 0.5 and 0.143, respectively.

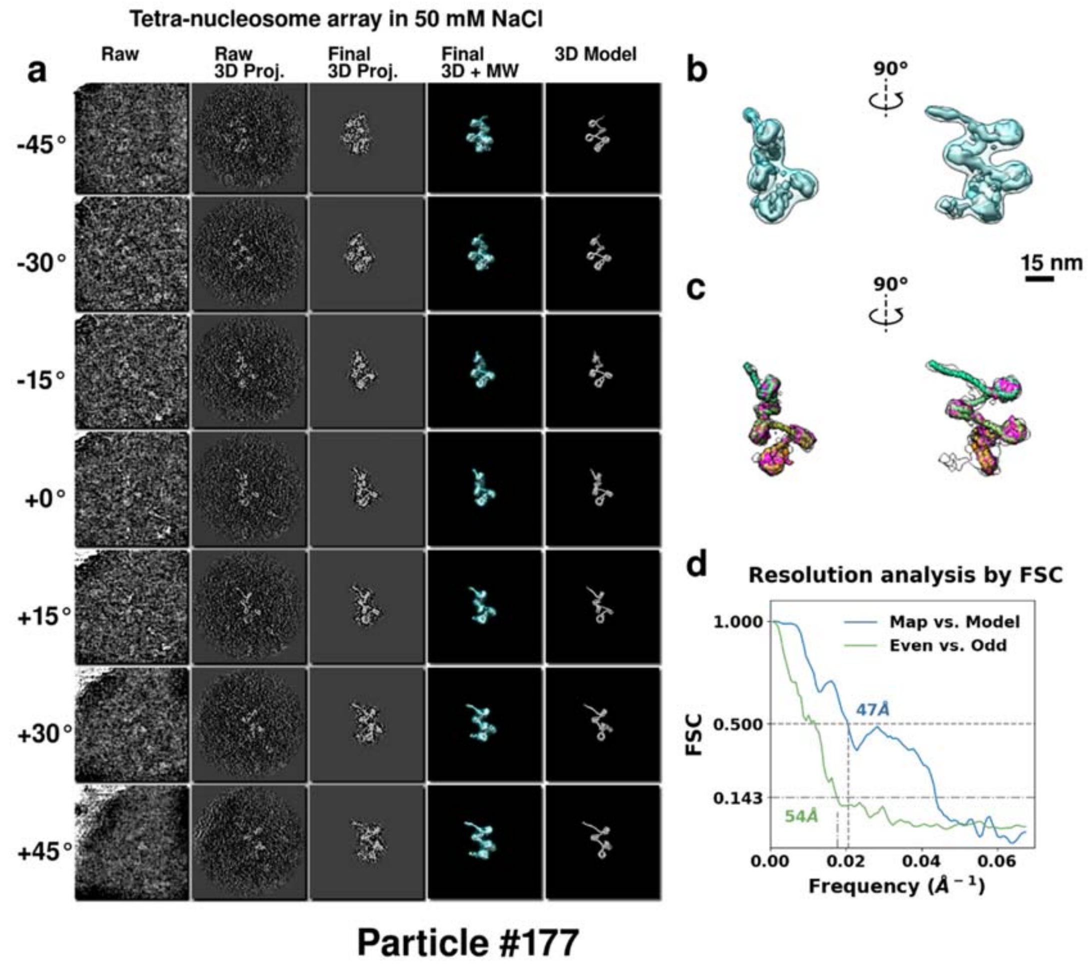

Supplementary Fig. 190. Cryo-ET 3D reconstruction of individual tetra-nucleosome particle (index no. 177 in 50 mM NaCl. a, IPET 3D reconstruction of individual tetra-nucleosome particles. The first column shows seven representative tilt images of an individual particle after CTF correction. Through alignment of the tilt images to a common center for 3D reconstruction via iterative refinement, the second and third columns display the 3D projections of the reconstruction before and after particle-shaped masking, respectively. The fourth column shows the final 3D reconstruction with missing wedge correction, and the fifth column presents the flexibly fitted model at the corresponding tilt angles. **b**, Zoomed-in view of the final 3D density map displayed in orthogonal views, shown at two contour levels. **c**, Superimposition of the high contour level map from (b) onto its flexibly fitted model. **d**, Resolution evaluation of the final 3D density map using two criteria: Fourier shell correlation (FSC) between two-half maps reconstructed from the even and odd index of the tilted series and FSC between the final 3D map and the fitted structure model. The resolution for the former and latter criteria is evaluated at frequencies of 0.5 and 0.143, respectively.

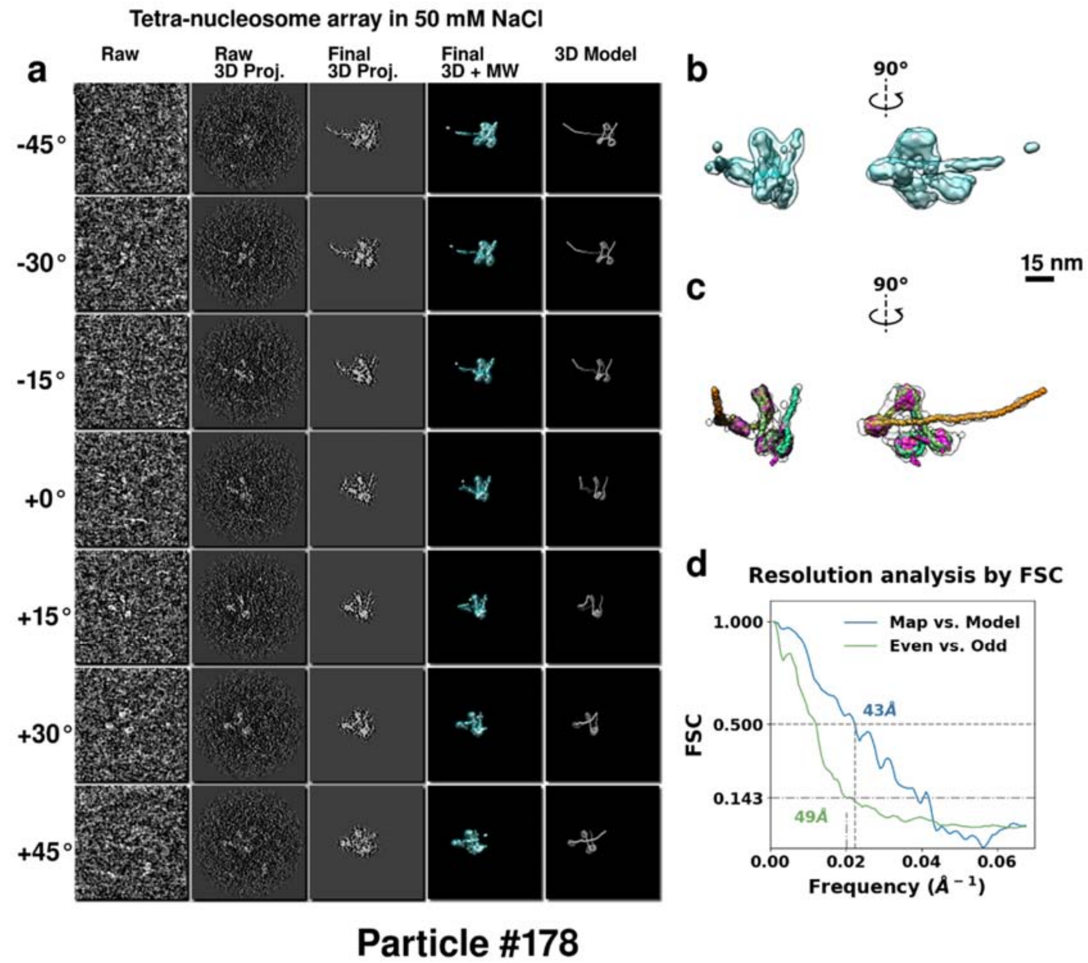

Supplementary Fig. 191. Cryo-ET 3D reconstruction of individual tetra-nucleosome particle (index no. 178 in 50 mM NaCl. a, IPET 3D reconstruction of individual tetra-nucleosome particles. The first column shows seven representative tilt images of an individual particle after CTF correction. Through alignment of the tilt images to a common center for 3D reconstruction via iterative refinement, the second and third columns display the 3D projections of the reconstruction before and after particle-shaped masking, respectively. The fourth column shows the final 3D reconstruction with missing wedge correction, and the fifth column presents the flexibly fitted model at the corresponding tilt angles. **b**, Zoomed-in view of the final 3D density map displayed in orthogonal views, shown at two contour levels. **c**, Superimposition of the high contour level map from (b) onto its flexibly fitted model. **d**, Resolution evaluation of the final 3D density map using two criteria: Fourier shell correlation (FSC) between two-half maps reconstructed from the even and odd index of the tilted series and FSC between the final 3D map and the fitted structure model. The resolution for the former and latter criteria is evaluated at frequencies of 0.5 and 0.143, respectively.

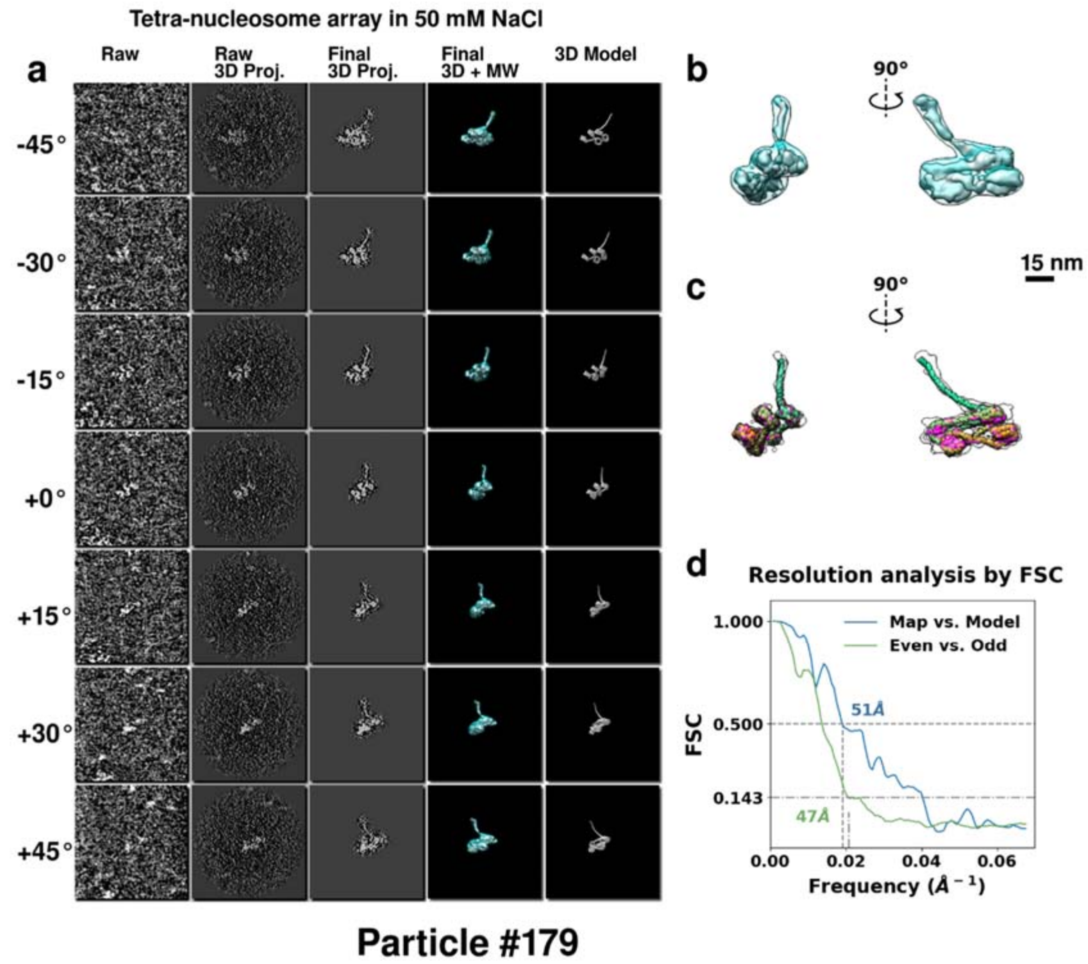

Supplementary Fig. 192. Cryo-ET 3D reconstruction of individual tetra-nucleosome particle (index no. 179 in 50 mM NaCl. a, IPET 3D reconstruction of individual tetra-nucleosome particles. The first column shows seven representative tilt images of an individual particle after CTF correction. Through alignment of the tilt images to a common center for 3D reconstruction via iterative refinement, the second and third columns display the 3D projections of the reconstruction before and after particle-shaped masking, respectively. The fourth column shows the final 3D reconstruction with missing wedge correction, and the fifth column presents the flexibly fitted model at the corresponding tilt angles. **b**, Zoomed-in view of the final 3D density map displayed in orthogonal views, shown at two contour levels. **c**, Superimposition of the high contour level map from (b) onto its flexibly fitted model. **d**, Resolution evaluation of the final 3D density map using two criteria: Fourier shell correlation (FSC) between two-half maps reconstructed from the even and odd index of the tilted series and FSC between the final 3D map and the fitted structure model. The resolution for the former and latter criteria is evaluated at frequencies of 0.5 and 0.143, respectively.

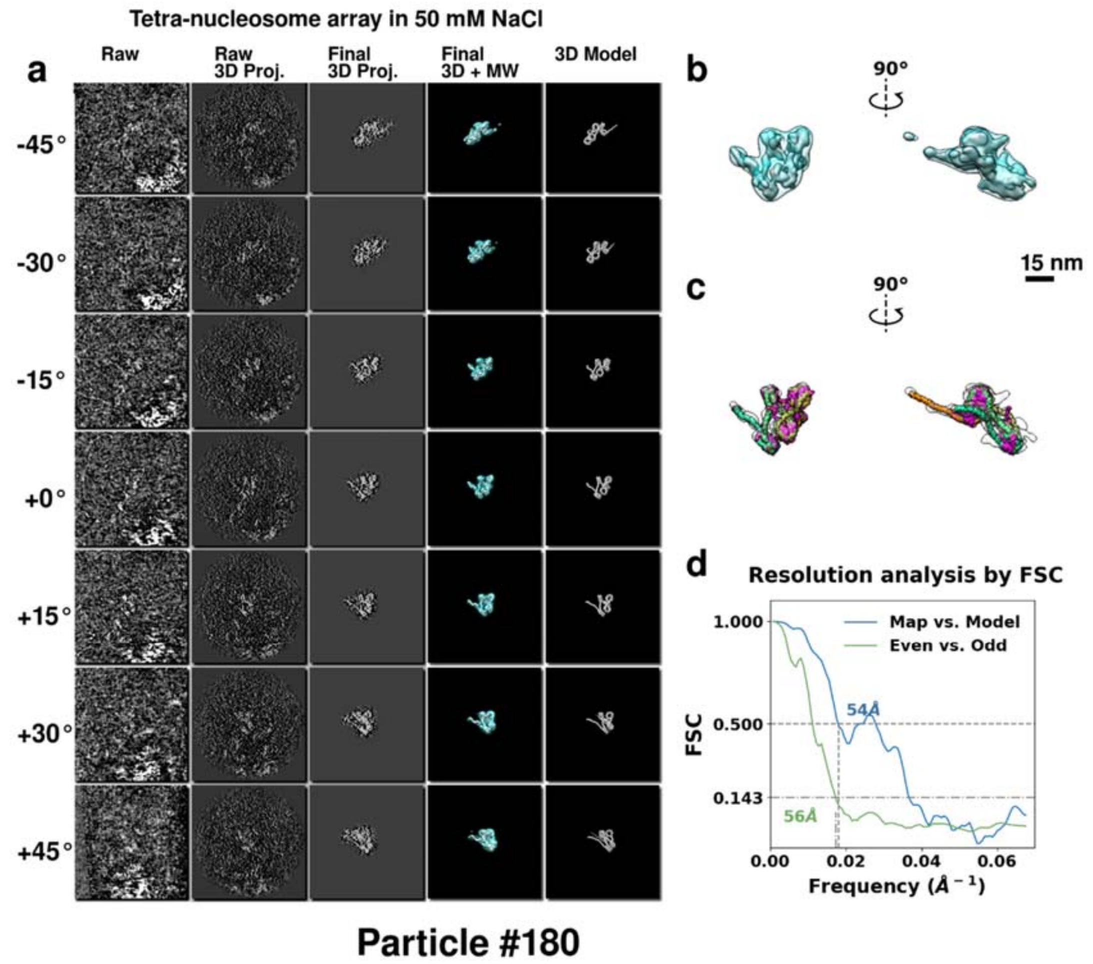

Supplementary Fig. 193. Cryo-ET 3D reconstruction of individual tetra-nucleosome particle (index no. 180 in 50 mM NaCl. a, IPET 3D reconstruction of individual tetra-nucleosome particles. The first column shows seven representative tilt images of an individual particle after CTF correction. Through alignment of the tilt images to a common center for 3D reconstruction via iterative refinement, the second and third columns display the 3D projections of the reconstruction before and after particle-shaped masking, respectively. The fourth column shows the final 3D reconstruction with missing wedge correction, and the fifth column presents the flexibly fitted model at the corresponding tilt angles. **b**, Zoomed-in view of the final 3D density map displayed in orthogonal views, shown at two contour levels. **c**, Superimposition of the high contour level map from (b) onto its flexibly fitted model. **d**, Resolution evaluation of the final 3D density map using two criteria: Fourier shell correlation (FSC) between two-half maps reconstructed from the even and odd index of the tilted series and FSC between the final 3D map and the fitted structure model. The resolution for the former and latter criteria is evaluated at frequencies of 0.5 and 0.143, respectively.

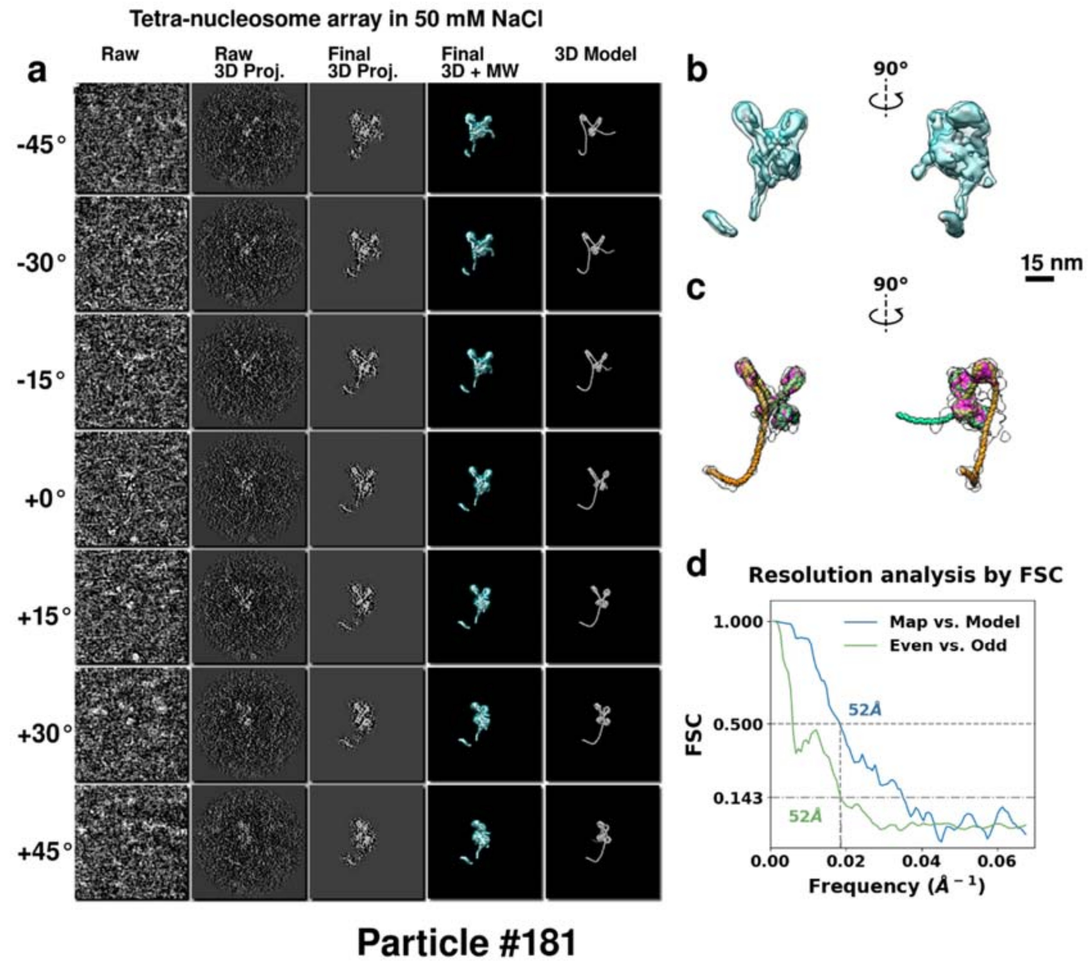

Supplementary Fig. 194. Cryo-ET 3D reconstruction of individual tetra-nucleosome particle (index no. 181 in 50 mM NaCl. a, IPET 3D reconstruction of individual tetra-nucleosome particles. The first column shows seven representative tilt images of an individual particle after CTF correction. Through alignment of the tilt images to a common center for 3D reconstruction via iterative refinement, the second and third columns display the 3D projections of the reconstruction before and after particle-shaped masking, respectively. The fourth column shows the final 3D reconstruction with missing wedge correction, and the fifth column presents the flexibly fitted model at the corresponding tilt angles. **b**, Zoomed-in view of the final 3D density map displayed in orthogonal views, shown at two contour levels. **c**, Superimposition of the high contour level map from (b) onto its flexibly fitted model. **d**, Resolution evaluation of the final 3D density map using two criteria: Fourier shell correlation (FSC) between two-half maps reconstructed from the even and odd index of the tilted series and FSC between the final 3D map and the fitted structure model. The resolution for the former and latter criteria is evaluated at frequencies of 0.5 and 0.143, respectively.

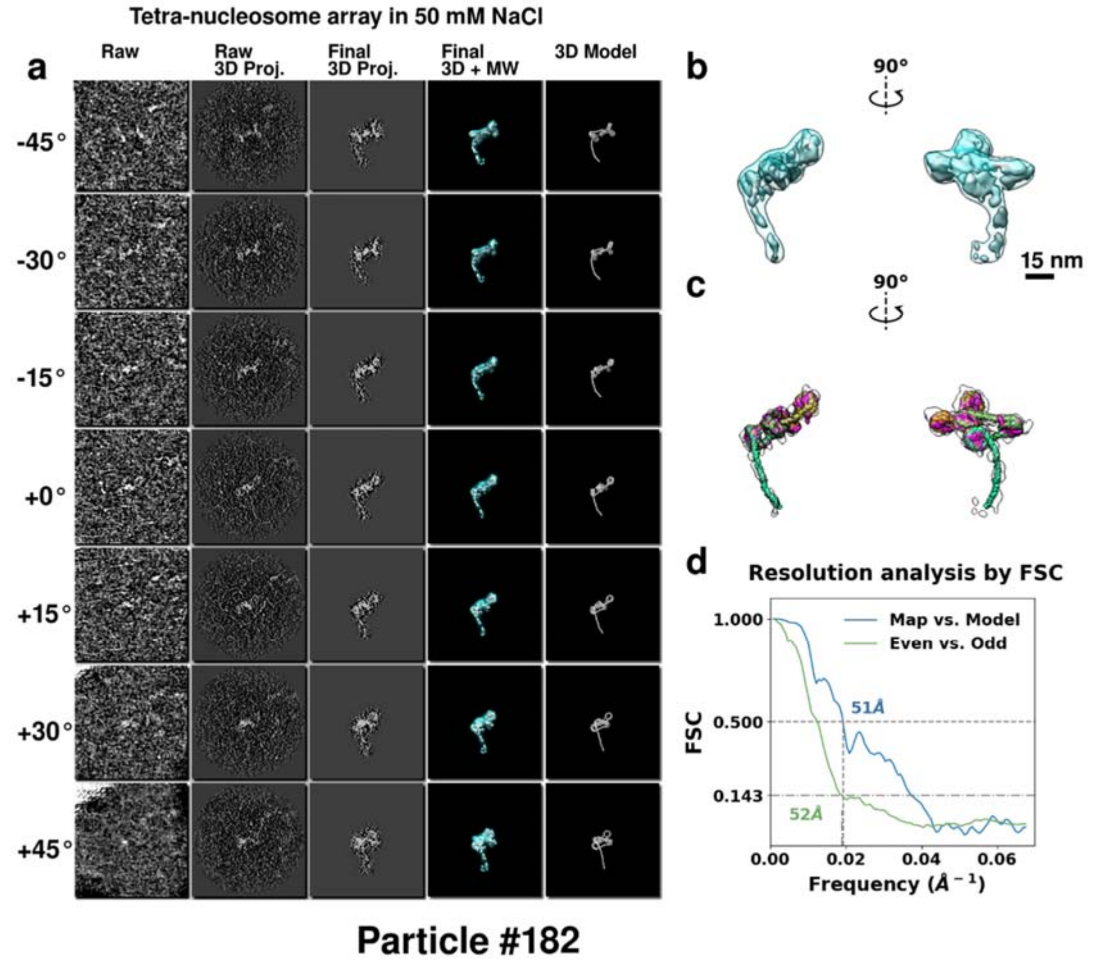

Supplementary Fig. 195. Cryo-ET 3D reconstruction of individual tetra-nucleosome particle (index no. 182 in 50 mM NaCl. a, IPET 3D reconstruction of individual tetra-nucleosome particles. The first column shows seven representative tilt images of an individual particle after CTF correction. Through alignment of the tilt images to a common center for 3D reconstruction via iterative refinement, the second and third columns display the 3D projections of the reconstruction before and after particle-shaped masking, respectively. The fourth column shows the final 3D reconstruction with missing wedge correction, and the fifth column presents the flexibly fitted model at the corresponding tilt angles. **b**, Zoomed-in view of the final 3D density map displayed in orthogonal views, shown at two contour levels. **c**, Superimposition of the high contour level map from (b) onto its flexibly fitted model. **d**, Resolution evaluation of the final 3D density map using two criteria: Fourier shell correlation (FSC) between two-half maps reconstructed from the even and odd index of the tilted series and FSC between the final 3D map and the fitted structure model. The resolution for the former and latter criteria is evaluated at frequencies of 0.5 and 0.143, respectively.

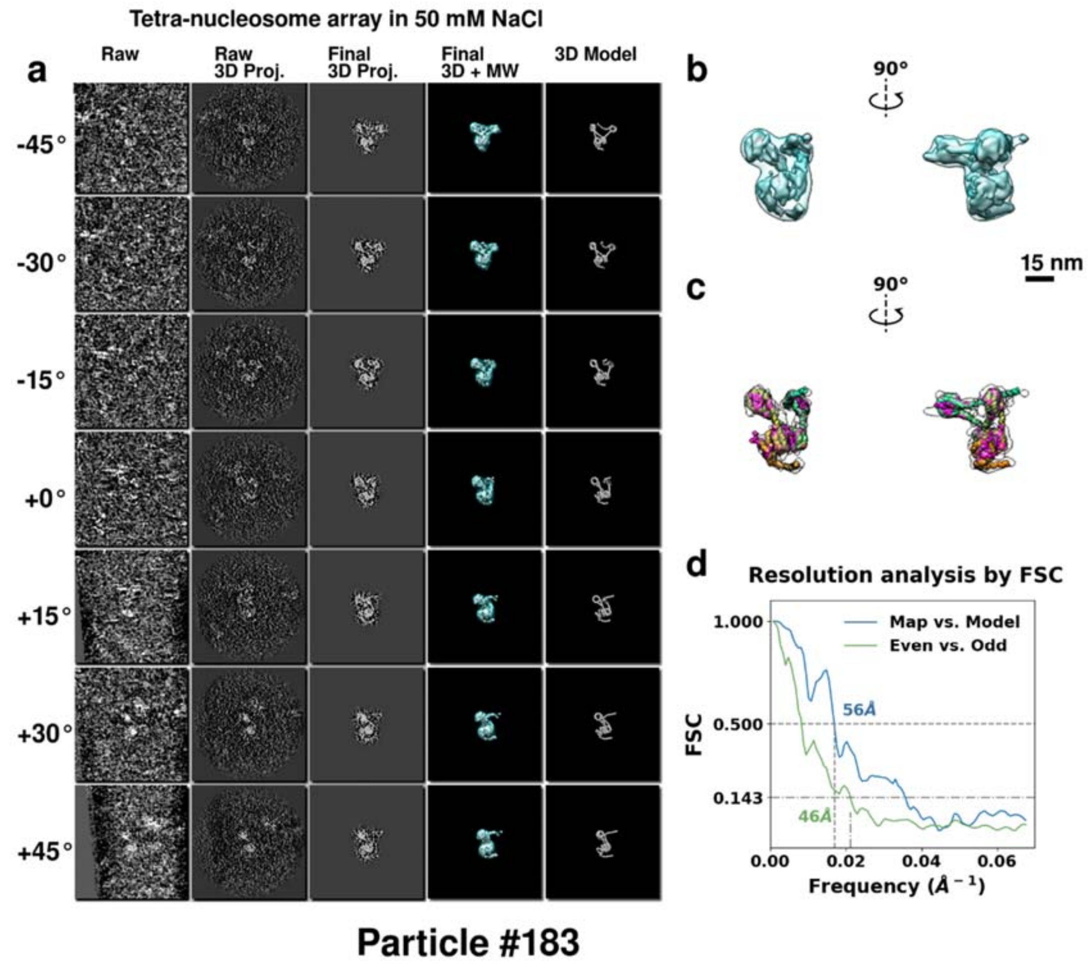

Supplementary Fig. 196. Cryo-ET 3D reconstruction of individual tetra-nucleosome particle (index no. 183 in 50 mM NaCl. a, IPET 3D reconstruction of individual tetra-nucleosome particles. The first column shows seven representative tilt images of an individual particle after CTF correction. Through alignment of the tilt images to a common center for 3D reconstruction via iterative refinement, the second and third columns display the 3D projections of the reconstruction before and after particle-shaped masking, respectively. The fourth column shows the final 3D reconstruction with missing wedge correction, and the fifth column presents the flexibly fitted model at the corresponding tilt angles. **b**, Zoomed-in view of the final 3D density map displayed in orthogonal views, shown at two contour levels. **c**, Superimposition of the high contour level map from (b) onto its flexibly fitted model. **d**, Resolution evaluation of the final 3D density map using two criteria: Fourier shell correlation (FSC) between two-half maps reconstructed from the even and odd index of the tilted series and FSC between the final 3D map and the fitted structure model. The resolution for the former and latter criteria is evaluated at frequencies of 0.5 and 0.143, respectively.

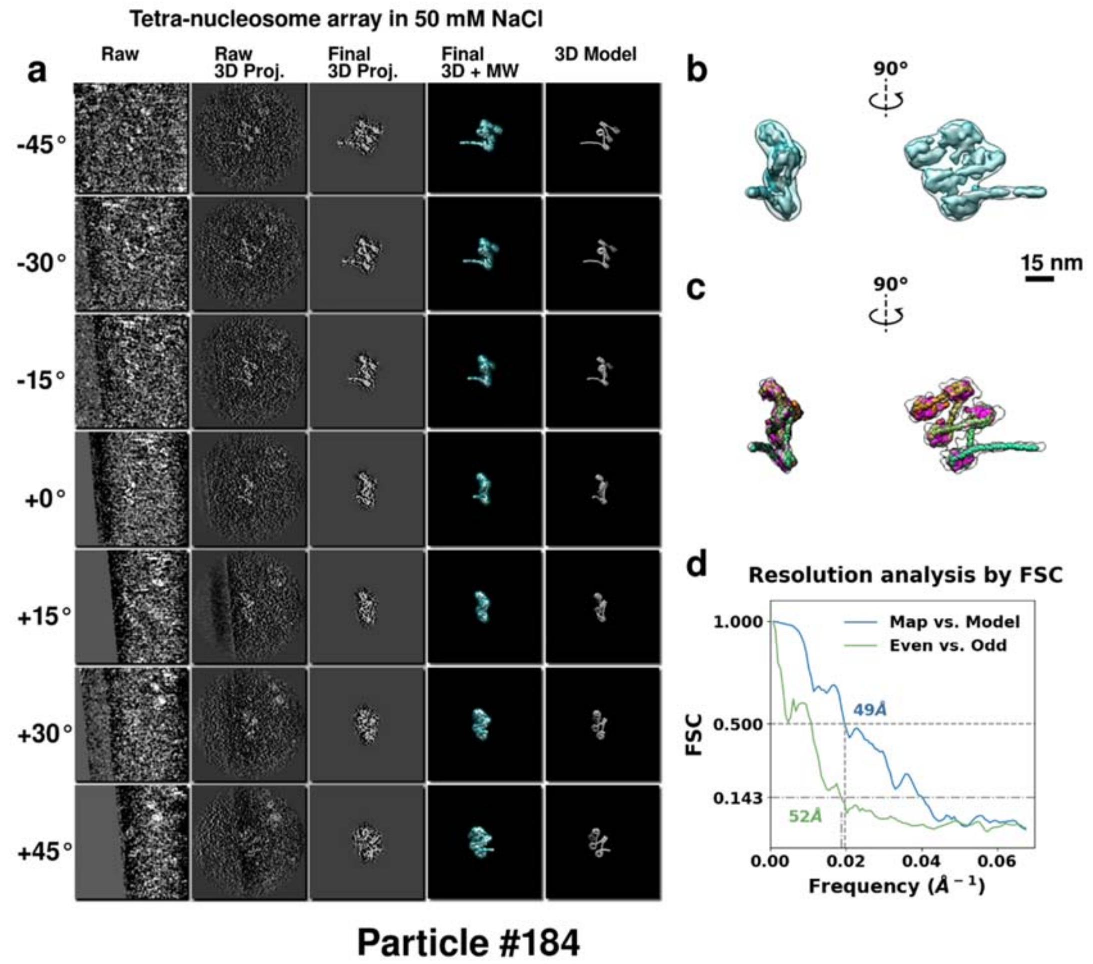

Supplementary Fig. 197. Cryo-ET 3D reconstruction of individual tetra-nucleosome particle (index no. 184 in 50 mM NaCl. a, IPET 3D reconstruction of individual tetra-nucleosome particles. The first column shows seven representative tilt images of an individual particle after CTF correction. Through alignment of the tilt images to a common center for 3D reconstruction via iterative refinement, the second and third columns display the 3D projections of the reconstruction before and after particle-shaped masking, respectively. The fourth column shows the final 3D reconstruction with missing wedge correction, and the fifth column presents the flexibly fitted model at the corresponding tilt angles. **b**, Zoomed-in view of the final 3D density map displayed in orthogonal views, shown at two contour levels. **c**, Superimposition of the high contour level map from (b) onto its flexibly fitted model. **d**, Resolution evaluation of the final 3D density map using two criteria: Fourier shell correlation (FSC) between two-half maps reconstructed from the even and odd index of the tilted series and FSC between the final 3D map and the fitted structure model. The resolution for the former and latter criteria is evaluated at frequencies of 0.5 and 0.143, respectively.

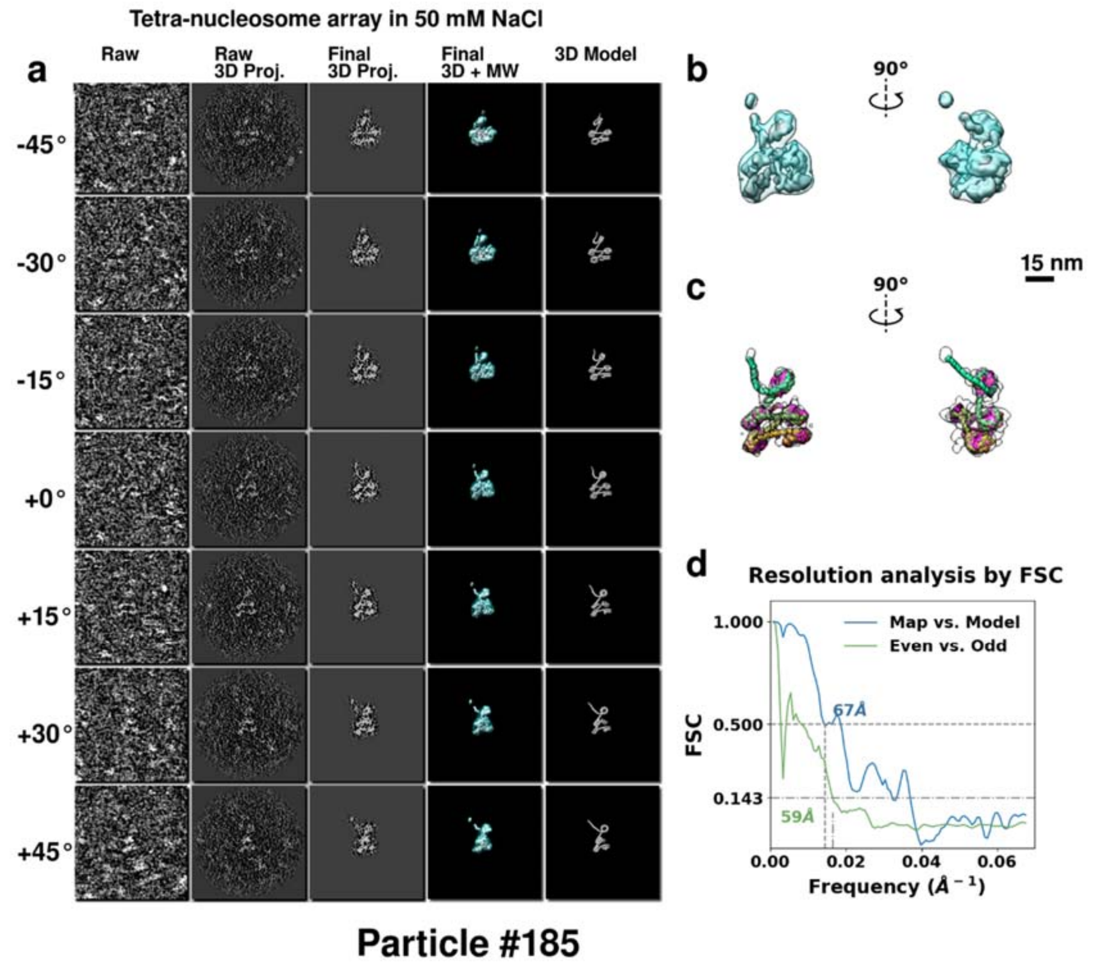

Supplementary Fig. 198. Cryo-ET 3D reconstruction of individual tetra-nucleosome particle (index no. 185 in 50 mM NaCl. a, IPET 3D reconstruction of individual tetra-nucleosome particles. The first column shows seven representative tilt images of an individual particle after CTF correction. Through alignment of the tilt images to a common center for 3D reconstruction via iterative refinement, the second and third columns display the 3D projections of the reconstruction before and after particle-shaped masking, respectively. The fourth column shows the final 3D reconstruction with missing wedge correction, and the fifth column presents the flexibly fitted model at the corresponding tilt angles. **b**, Zoomed-in view of the final 3D density map displayed in orthogonal views, shown at two contour levels. **c**, Superimposition of the high contour level map from (b) onto its flexibly fitted model. **d**, Resolution evaluation of the final 3D density map using two criteria: Fourier shell correlation (FSC) between two-half maps reconstructed from the even and odd index of the tilted series and FSC between the final 3D map and the fitted structure model. The resolution for the former and latter criteria is evaluated at frequencies of 0.5 and 0.143, respectively.

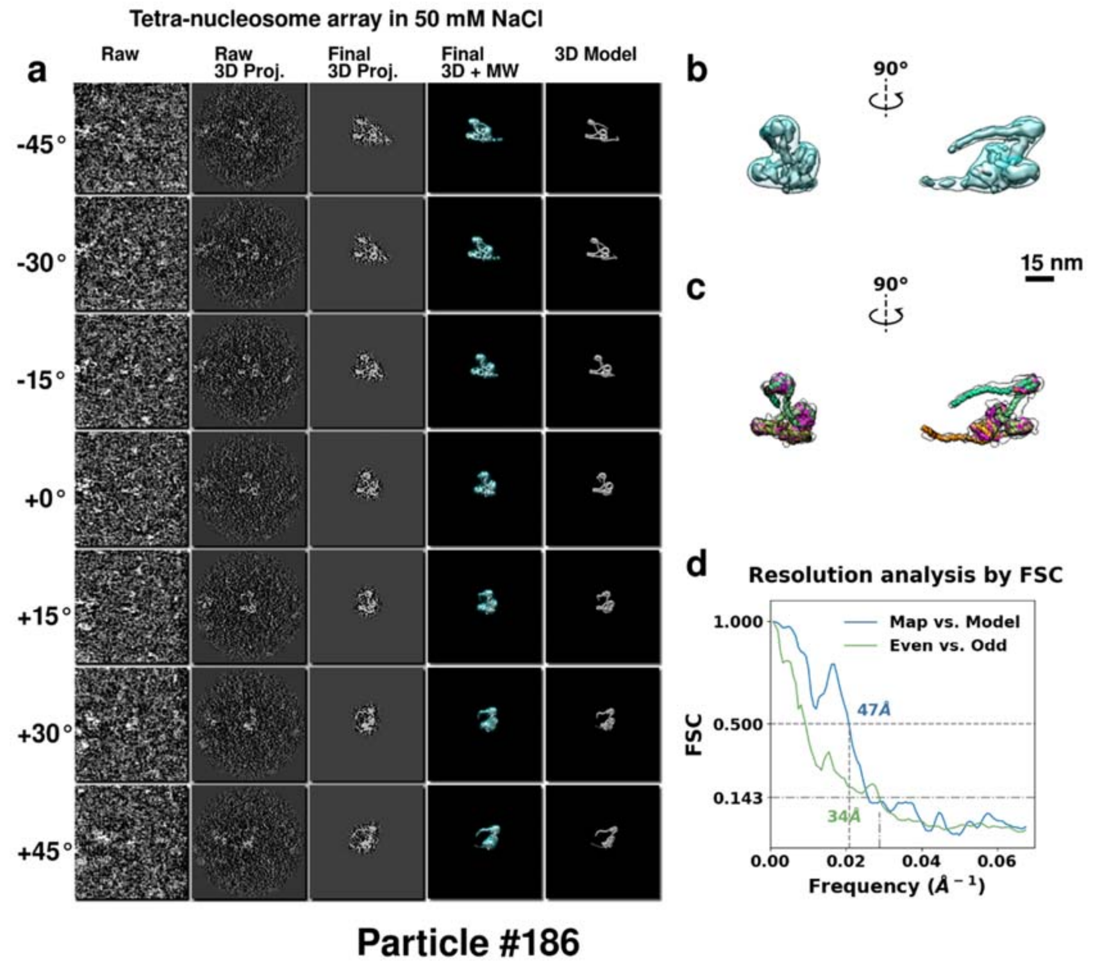

Supplementary Fig. 199. Cryo-ET 3D reconstruction of individual tetra-nucleosome particle (index no. 186 in 50 mM NaCl. a, IPET 3D reconstruction of individual tetra-nucleosome particles. The first column shows seven representative tilt images of an individual particle after CTF correction. Through alignment of the tilt images to a common center for 3D reconstruction via iterative refinement, the second and third columns display the 3D projections of the reconstruction before and after particle-shaped masking, respectively. The fourth column shows the final 3D reconstruction with missing wedge correction, and the fifth column presents the flexibly fitted model at the corresponding tilt angles. **b**, Zoomed-in view of the final 3D density map displayed in orthogonal views, shown at two contour levels. **c**, Superimposition of the high contour level map from (b) onto its flexibly fitted model. **d**, Resolution evaluation of the final 3D density map using two criteria: Fourier shell correlation (FSC) between two-half maps reconstructed from the even and odd index of the tilted series and FSC between the final 3D map and the fitted structure model. The resolution for the former and latter criteria is evaluated at frequencies of 0.5 and 0.143, respectively.

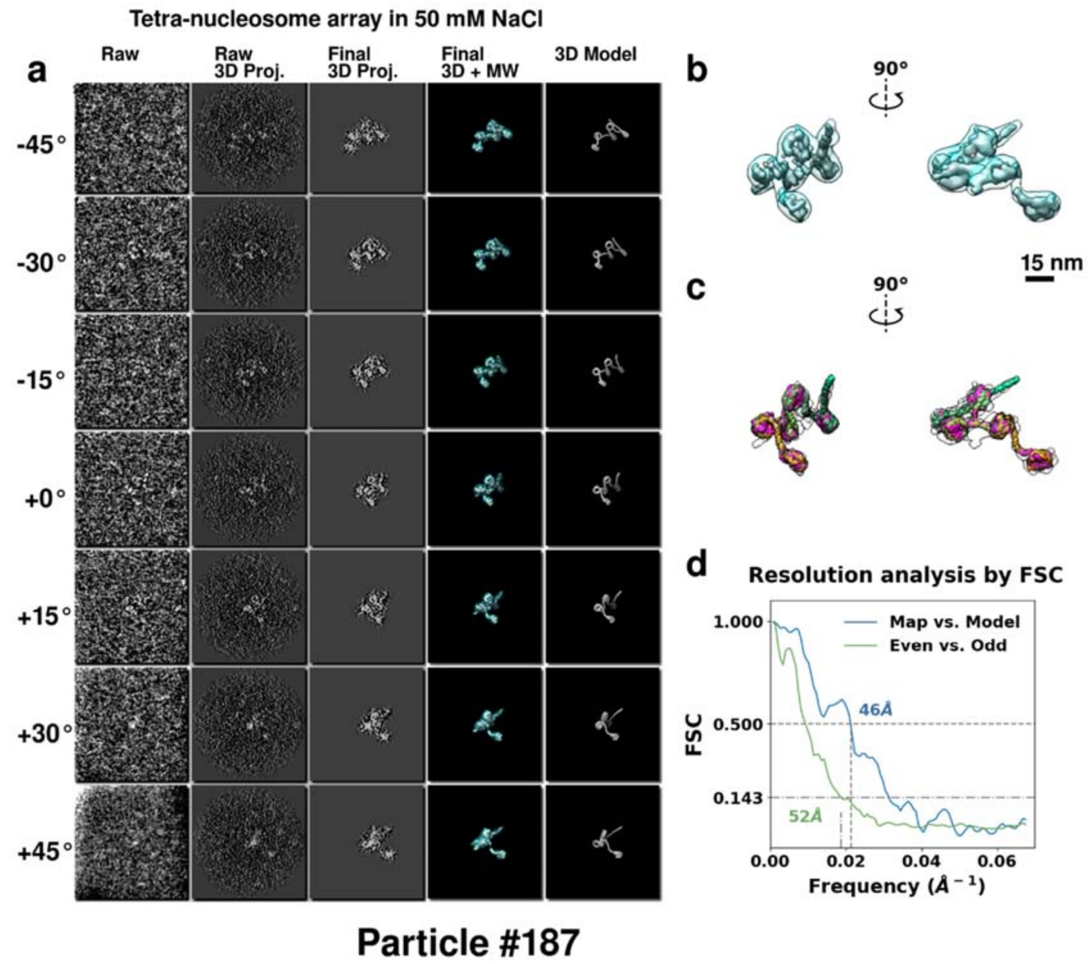

Supplementary Fig. 200. Cryo-ET 3D reconstruction of individual tetra-nucleosome particle (index no. 187 in 50 mM NaCl. a, IPET 3D reconstruction of individual tetra-nucleosome particles. The first column shows seven representative tilt images of an individual particle after CTF correction. Through alignment of the tilt images to a common center for 3D reconstruction via iterative refinement, the second and third columns display the 3D projections of the reconstruction before and after particle-shaped masking, respectively. The fourth column shows the final 3D reconstruction with missing wedge correction, and the fifth column presents the flexibly fitted model at the corresponding tilt angles. **b**, Zoomed-in view of the final 3D density map displayed in orthogonal views, shown at two contour levels. **c**, Superimposition of the high contour level map from (b) onto its flexibly fitted model. **d**, Resolution evaluation of the final 3D density map using two criteria: Fourier shell correlation (FSC) between two-half maps reconstructed from the even and odd index of the tilted series and FSC between the final 3D map and the fitted structure model. The resolution for the former and latter criteria is evaluated at frequencies of 0.5 and 0.143, respectively.

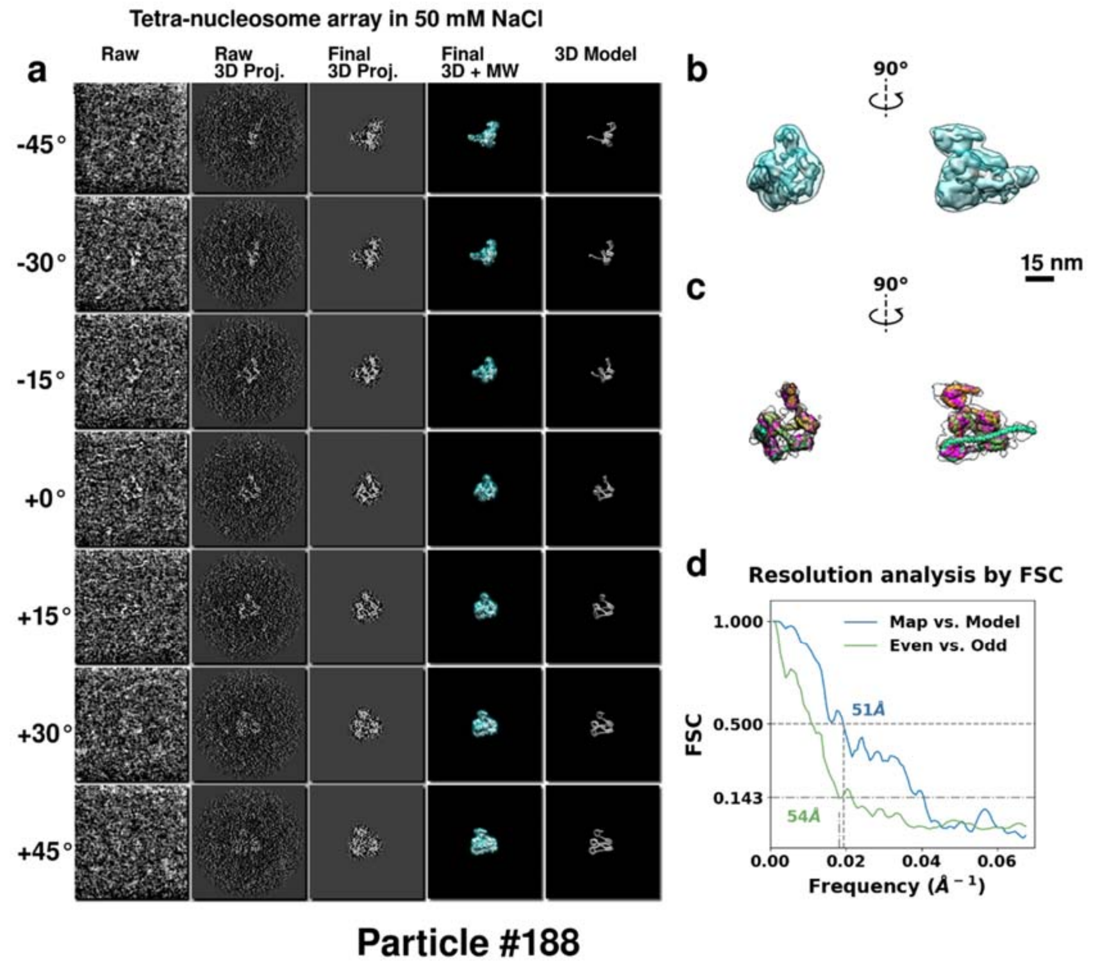

Supplementary Fig. 201. Cryo-ET 3D reconstruction of individual tetra-nucleosome particle (index no. 188 in 50 mM NaCl. a, IPET 3D reconstruction of individual tetra-nucleosome particles. The first column shows seven representative tilt images of an individual particle after CTF correction. Through alignment of the tilt images to a common center for 3D reconstruction via iterative refinement, the second and third columns display the 3D projections of the reconstruction before and after particle-shaped masking, respectively. The fourth column shows the final 3D reconstruction with missing wedge correction, and the fifth column presents the flexibly fitted model at the corresponding tilt angles. **b**, Zoomed-in view of the final 3D density map displayed in orthogonal views, shown at two contour levels. **c**, Superimposition of the high contour level map from (b) onto its flexibly fitted model. **d**, Resolution evaluation of the final 3D density map using two criteria: Fourier shell correlation (FSC) between two-half maps reconstructed from the even and odd index of the tilted series and FSC between the final 3D map and the fitted structure model. The resolution for the former and latter criteria is evaluated at frequencies of 0.5 and 0.143, respectively.

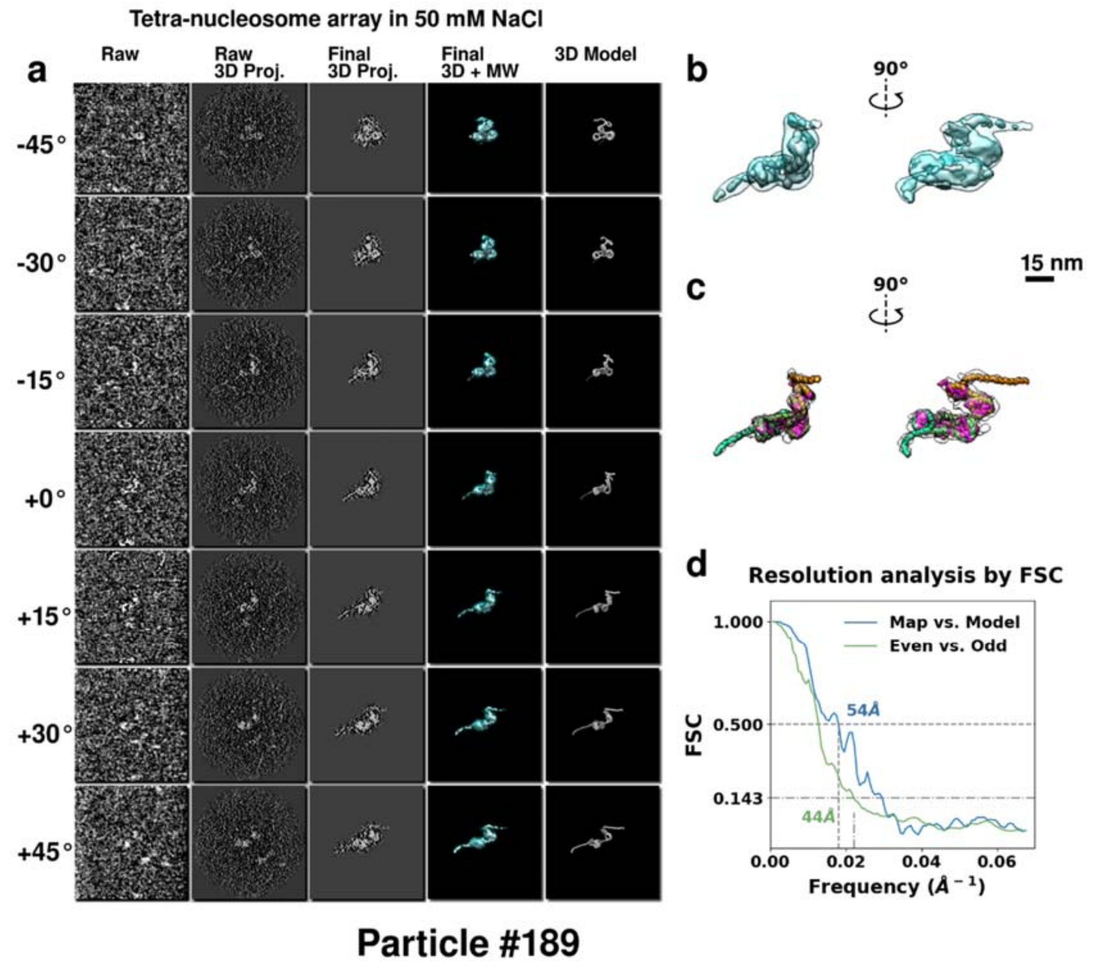

Supplementary Fig. 202. Cryo-ET 3D reconstruction of individual tetra-nucleosome particle (index no. 189 in 50 mM NaCl. a, IPET 3D reconstruction of individual tetra-nucleosome particles. The first column shows seven representative tilt images of an individual particle after CTF correction. Through alignment of the tilt images to a common center for 3D reconstruction via iterative refinement, the second and third columns display the 3D projections of the reconstruction before and after particle-shaped masking, respectively. The fourth column shows the final 3D reconstruction with missing wedge correction, and the fifth column presents the flexibly fitted model at the corresponding tilt angles. **b**, Zoomed-in view of the final 3D density map displayed in orthogonal views, shown at two contour levels. **c**, Superimposition of the high contour level map from (b) onto its flexibly fitted model. **d**, Resolution evaluation of the final 3D density map using two criteria: Fourier shell correlation (FSC) between two-half maps reconstructed from the even and odd index of the tilted series and FSC between the final 3D map and the fitted structure model. The resolution for the former and latter criteria is evaluated at frequencies of 0.5 and 0.143, respectively.

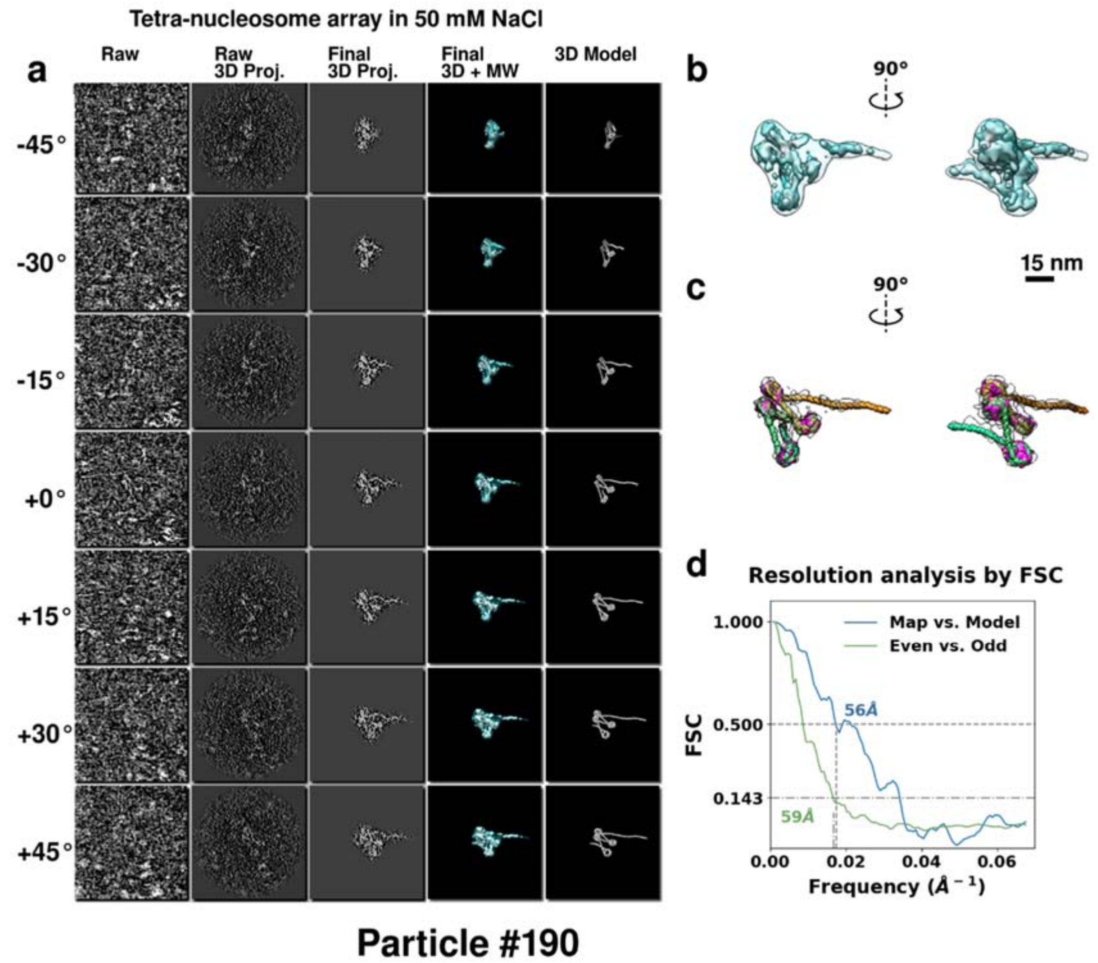

Supplementary Fig. 203. Cryo-ET 3D reconstruction of individual tetra-nucleosome particle (index no. 190 in 50 mM NaCl. a, IPET 3D reconstruction of individual tetra-nucleosome particles. The first column shows seven representative tilt images of an individual particle after CTF correction. Through alignment of the tilt images to a common center for 3D reconstruction via iterative refinement, the second and third columns display the 3D projections of the reconstruction before and after particle-shaped masking, respectively. The fourth column shows the final 3D reconstruction with missing wedge correction, and the fifth column presents the flexibly fitted model at the corresponding tilt angles. **b**, Zoomed-in view of the final 3D density map displayed in orthogonal views, shown at two contour levels. **c**, Superimposition of the high contour level map from (b) onto its flexibly fitted model. **d**, Resolution evaluation of the final 3D density map using two criteria: Fourier shell correlation (FSC) between two-half maps reconstructed from the even and odd index of the tilted series and FSC between the final 3D map and the fitted structure model. The resolution for the former and latter criteria is evaluated at frequencies of 0.5 and 0.143, respectively.

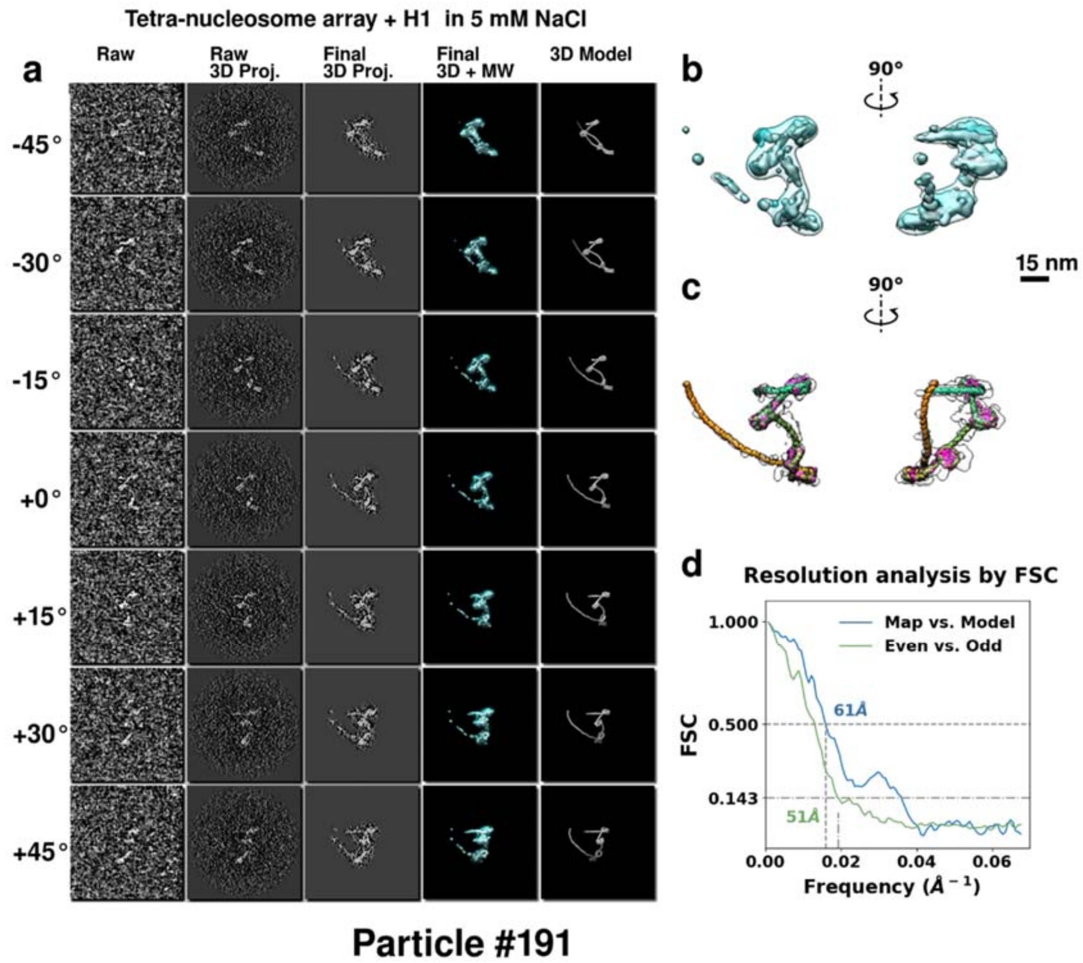

Supplementary Fig. 204. Cryo-ET 3D reconstruction of individual tetra-nucleosome particle (index no. 191 in 50 mM NaCl. a, IPET 3D reconstruction of individual tetra-nucleosome particles. The first column shows seven representative tilt images of an individual particle after CTF correction. Through alignment of the tilt images to a common center for 3D reconstruction via iterative refinement, the second and third columns display the 3D projections of the reconstruction before and after particle-shaped masking, respectively. The fourth column shows the final 3D reconstruction with missing wedge correction, and the fifth column presents the flexibly fitted model at the corresponding tilt angles. **b**, Zoomed-in view of the final 3D density map displayed in orthogonal views, shown at two contour levels. **c**, Superimposition of the high contour level map from (b) onto its flexibly fitted model. **d**, Resolution evaluation of the final 3D density map using two criteria: Fourier shell correlation (FSC) between two-half maps reconstructed from the even and odd index of the tilted series and FSC between the final 3D map and the fitted structure model. The resolution for the former and latter criteria is evaluated at frequencies of 0.5 and 0.143, respectively.

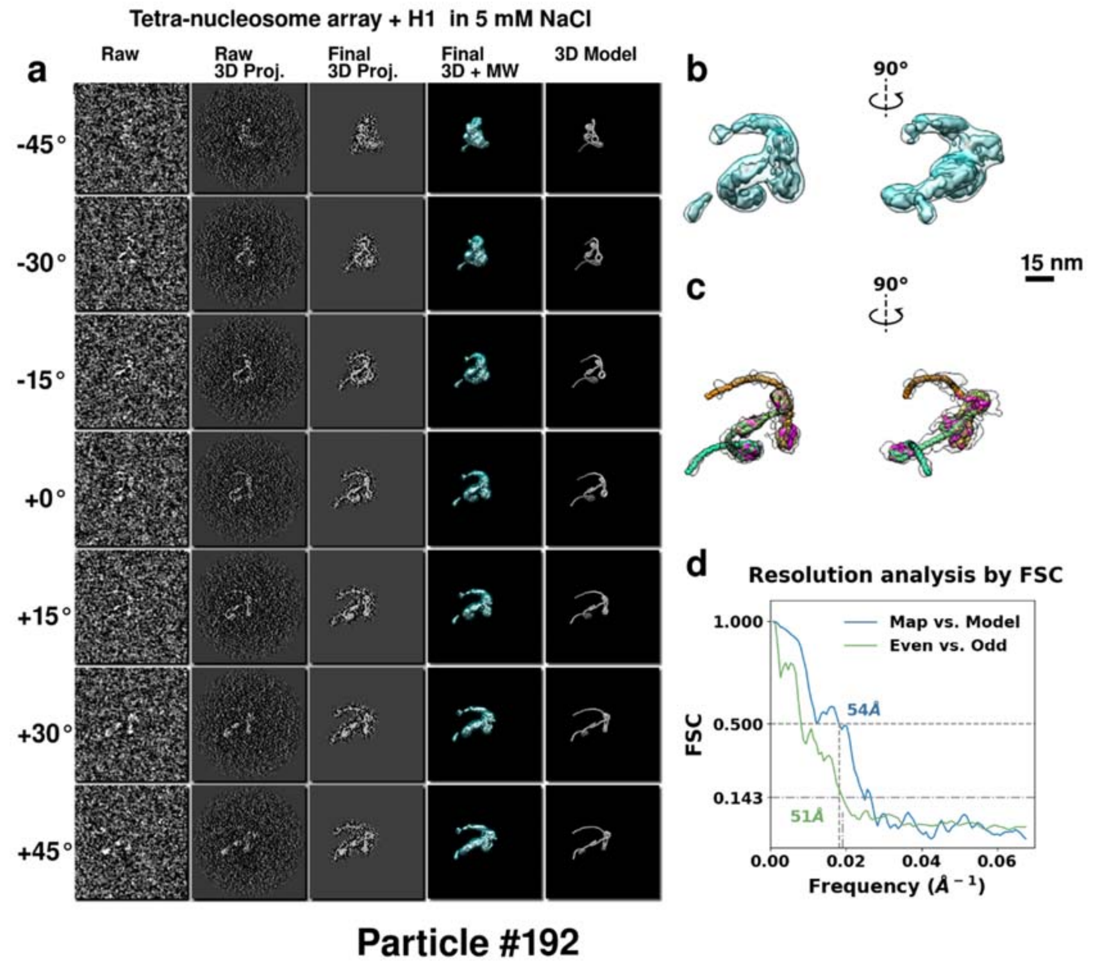

Supplementary Fig. 205. Cryo-ET 3D reconstruction of individual tetra-nucleosome particle (index no. 192) in 5 mM NaCl and presence of H1. a, IPET 3D reconstruction of individual tetra-nucleosome particles. The first column shows seven representative tilt images of an individual particle after CTF correction. Through alignment of the tilt images to a common center for 3D reconstruction via iterative refinement, the second and third columns display the 3D projections of the reconstruction before and after particle-shaped masking, respectively. The fourth column shows the final 3D reconstruction with missing wedge correction, and the fifth column presents the flexibly fitted model at the corresponding tilt angles. **b**, Zoomed-in view of the final 3D density map displayed in orthogonal views, shown at two contour levels. **c**, Superimposition of the high contour level map from (b) onto its flexibly fitted model. **d**, Resolution evaluation of the final 3D density map using two criteria: Fourier shell correlation (FSC) between two-half maps reconstructed from the even and odd index of the tilted series and FSC between the final 3D map and the fitted structure model. The resolution for the former and latter criteria is evaluated at frequencies of 0.5 and 0.143, respectively.

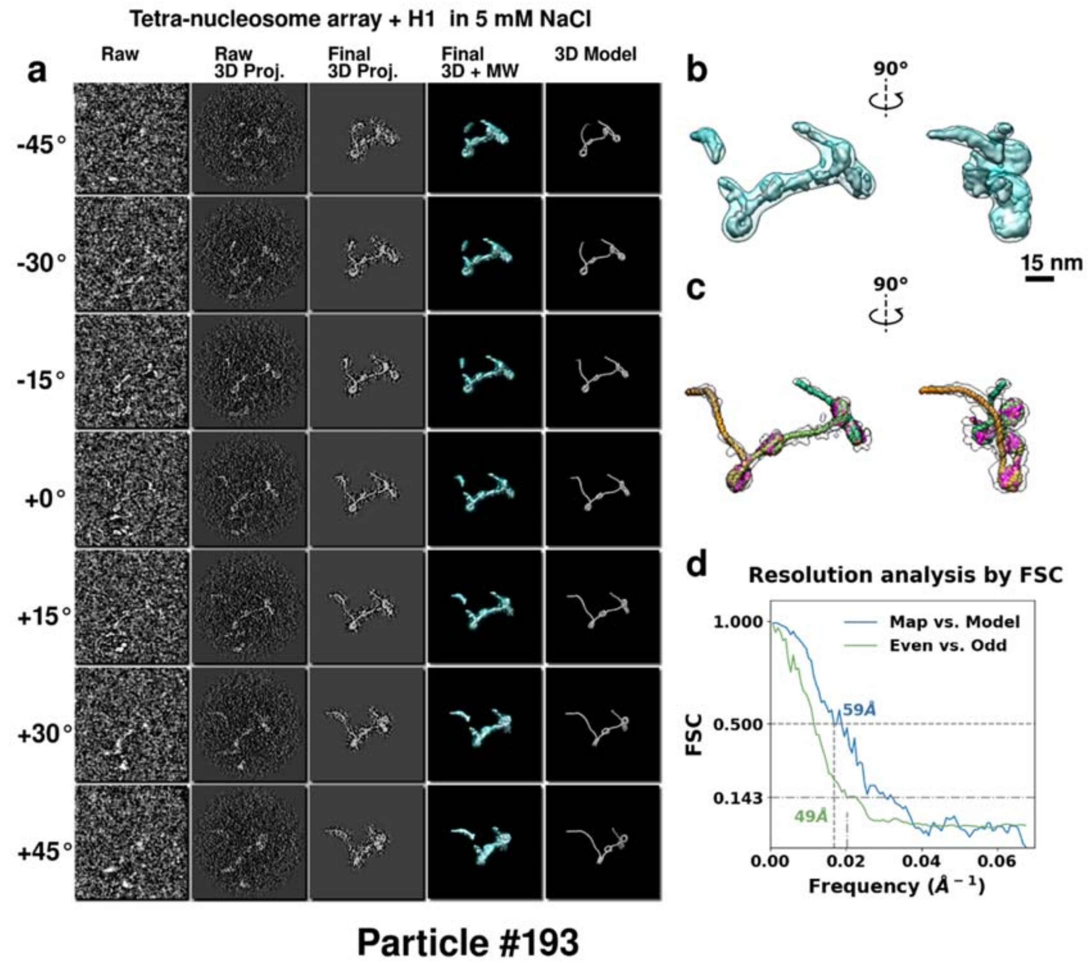

Supplementary Fig. 206. Cryo-ET 3D reconstruction of individual tetra-nucleosome particle (index no. 193) in 5 mM NaCl and presence of H1. a, IPET 3D reconstruction of individual tetra-nucleosome particles. The first column shows seven representative tilt images of an individual particle after CTF correction. Through alignment of the tilt images to a common center for 3D reconstruction via iterative refinement, the second and third columns display the 3D projections of the reconstruction before and after particle-shaped masking, respectively. The fourth column shows the final 3D reconstruction with missing wedge correction, and the fifth column presents the flexibly fitted model at the corresponding tilt angles. **b**, Zoomed-in view of the final 3D density map displayed in orthogonal views, shown at two contour levels. **c**, Superimposition of the high contour level map from (b) onto its flexibly fitted model. **d**, Resolution evaluation of the final 3D density map using two criteria: Fourier shell correlation (FSC) between two-half maps reconstructed from the even and odd index of the tilted series and FSC between the final 3D map and the fitted structure model. The resolution for the former and latter criteria is evaluated at frequencies of 0.5 and 0.143, respectively.

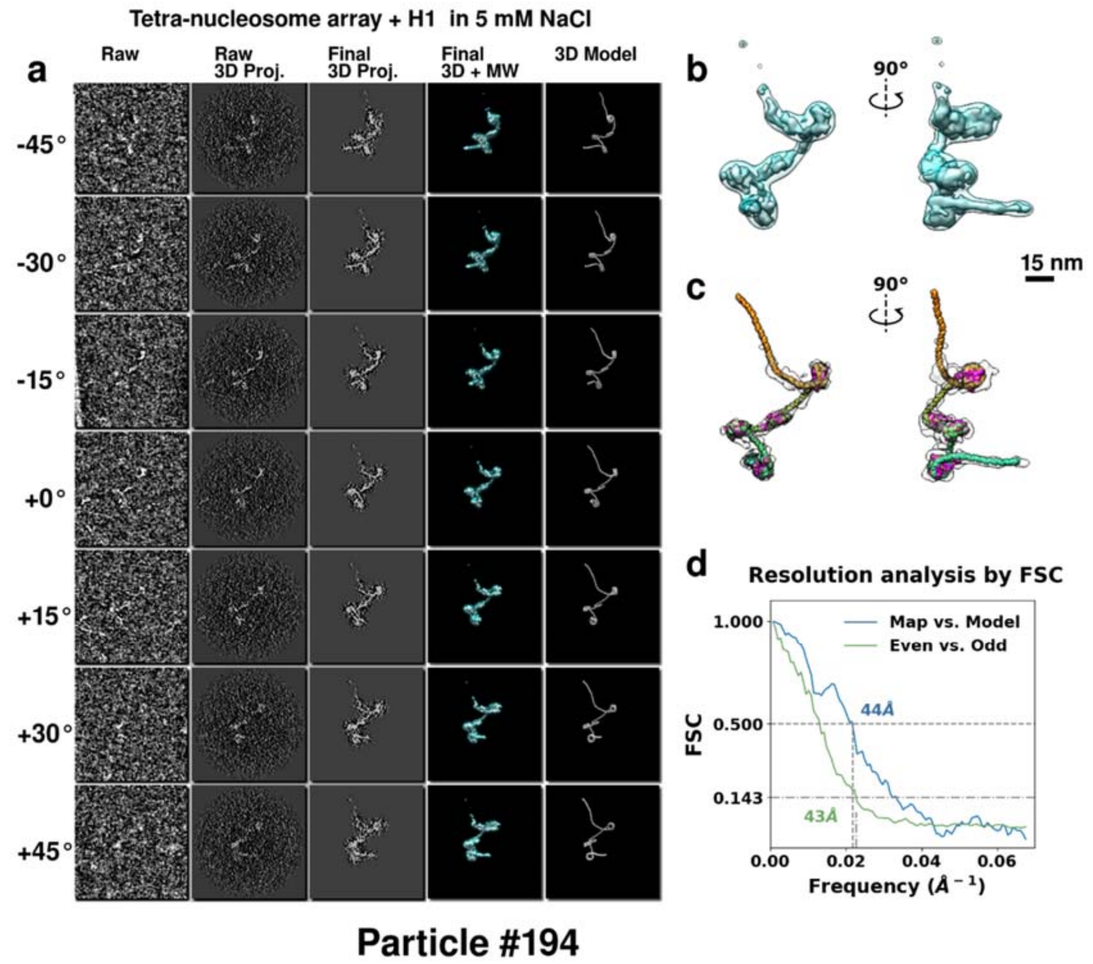

Supplementary Fig. 207. Cryo-ET 3D reconstruction of individual tetra-nucleosome particle (index no. 194) in 5 mM NaCl and presence of H1. a, IPET 3D reconstruction of individual tetra-nucleosome particles. The first column shows seven representative tilt images of an individual particle after CTF correction. Through alignment of the tilt images to a common center for 3D reconstruction via iterative refinement, the second and third columns display the 3D projections of the reconstruction before and after particle-shaped masking, respectively. The fourth column shows the final 3D reconstruction with missing wedge correction, and the fifth column presents the flexibly fitted model at the corresponding tilt angles. **b**, Zoomed-in view of the final 3D density map displayed in orthogonal views, shown at two contour levels. **c**, Superimposition of the high contour level map from (b) onto its flexibly fitted model. **d**, Resolution evaluation of the final 3D density map using two criteria: Fourier shell correlation (FSC) between two-half maps reconstructed from the even and odd index of the tilted series and FSC between the final 3D map and the fitted structure model. The resolution for the former and latter criteria is evaluated at frequencies of 0.5 and 0.143, respectively.

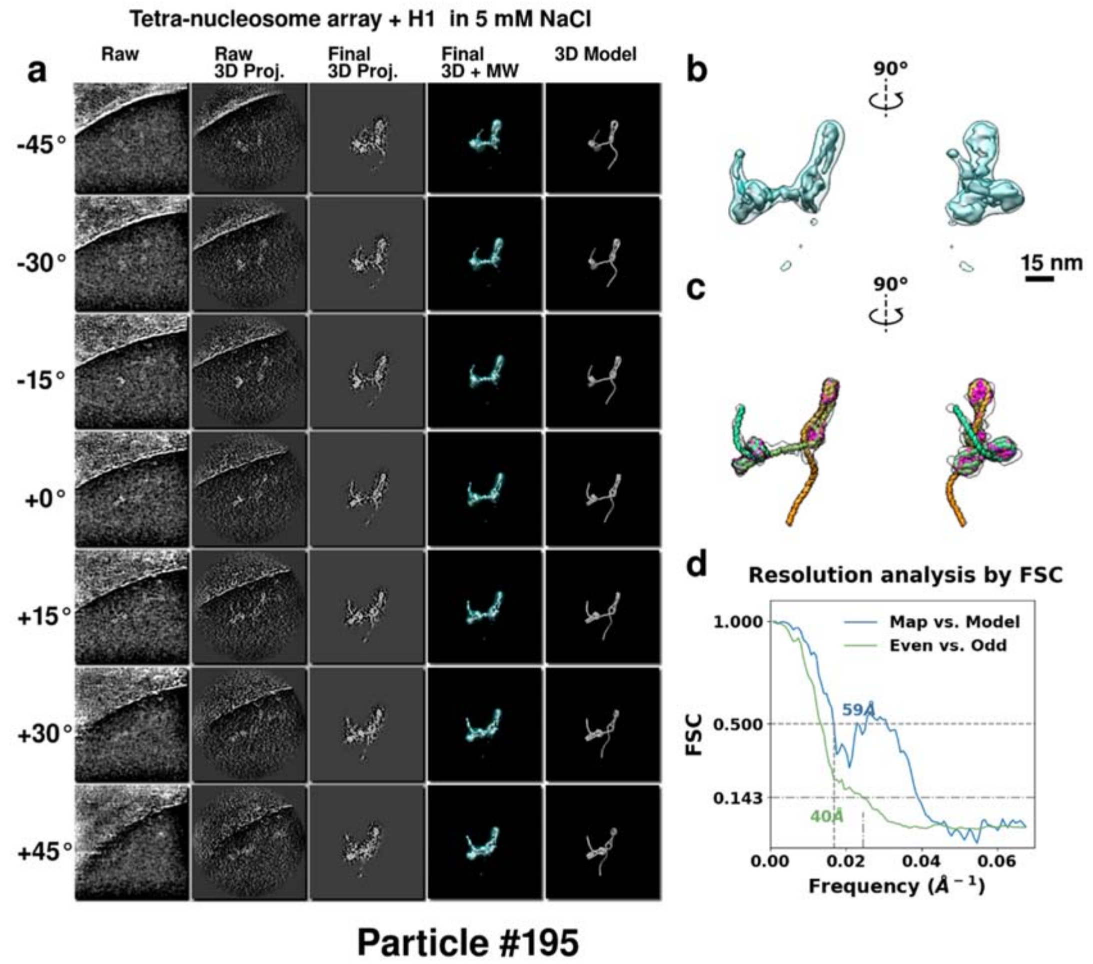

Supplementary Fig. 208. Cryo-ET 3D reconstruction of individual tetra-nucleosome particle (index no. 195) in 5 mM NaCl and presence of H1. a, IPET 3D reconstruction of individual tetra-nucleosome particles. The first column shows seven representative tilt images of an individual particle after CTF correction. Through alignment of the tilt images to a common center for 3D reconstruction via iterative refinement, the second and third columns display the 3D projections of the reconstruction before and after particle-shaped masking, respectively. The fourth column shows the final 3D reconstruction with missing wedge correction, and the fifth column presents the flexibly fitted model at the corresponding tilt angles. **b**, Zoomed-in view of the final 3D density map displayed in orthogonal views, shown at two contour levels. **c**, Superimposition of the high contour level map from (b) onto its flexibly fitted model. **d**, Resolution evaluation of the final 3D density map using two criteria: Fourier shell correlation (FSC) between two-half maps reconstructed from the even and odd index of the tilted series and FSC between the final 3D map and the fitted structure model. The resolution for the former and latter criteria is evaluated at frequencies of 0.5 and 0.143, respectively.

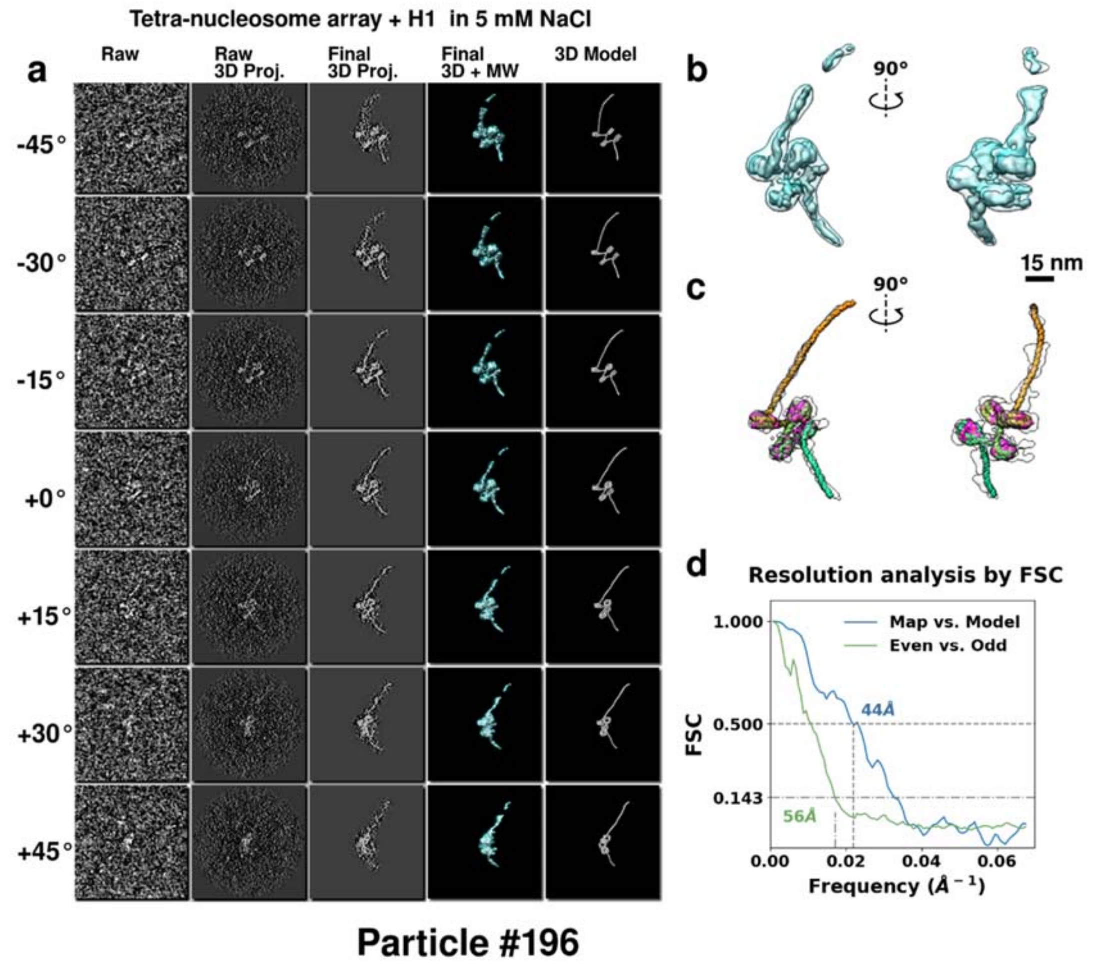

Supplementary Fig. 209. Cryo-ET 3D reconstruction of individual tetra-nucleosome particle (index no. 196) in 5 mM NaCl and presence of H1. a, IPET 3D reconstruction of individual tetra-nucleosome particles. The first column shows seven representative tilt images of an individual particle after CTF correction. Through alignment of the tilt images to a common center for 3D reconstruction via iterative refinement, the second and third columns display the 3D projections of the reconstruction before and after particle-shaped masking, respectively. The fourth column shows the final 3D reconstruction with missing wedge correction, and the fifth column presents the flexibly fitted model at the corresponding tilt angles. **b**, Zoomed-in view of the final 3D density map displayed in orthogonal views, shown at two contour levels. **c**, Superimposition of the high contour level map from (b) onto its flexibly fitted model. **d**, Resolution evaluation of the final 3D density map using two criteria: Fourier shell correlation (FSC) between two-half maps reconstructed from the even and odd index of the tilted series and FSC between the final 3D map and the fitted structure model. The resolution for the former and latter criteria is evaluated at frequencies of 0.5 and 0.143, respectively.

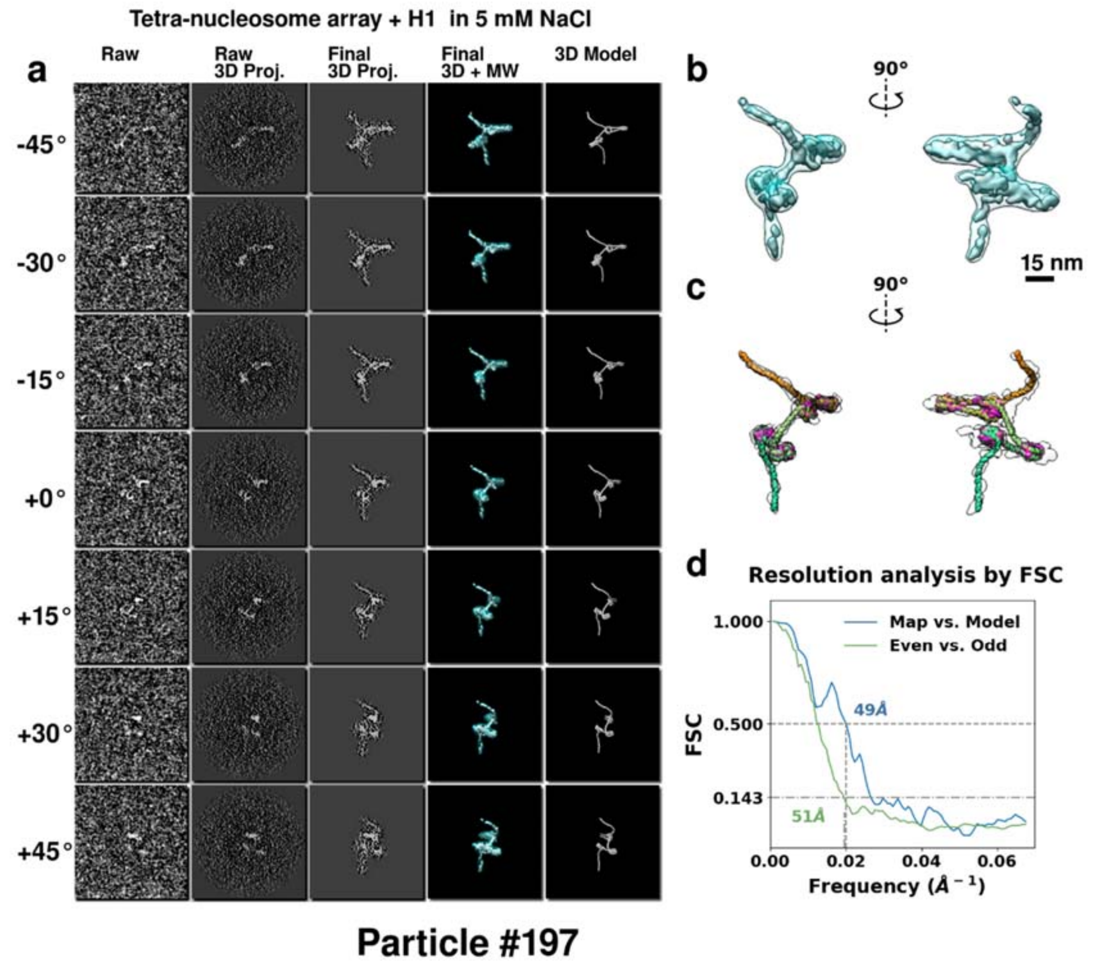

Supplementary Fig. 210. Cryo-ET 3D reconstruction of individual tetra-nucleosome particle (index no. 197) in 5 mM NaCl and presence of H1. a, IPET 3D reconstruction of individual tetra-nucleosome particles. The first column shows seven representative tilt images of an individual particle after CTF correction. Through alignment of the tilt images to a common center for 3D reconstruction via iterative refinement, the second and third columns display the 3D projections of the reconstruction before and after particle-shaped masking, respectively. The fourth column shows the final 3D reconstruction with missing wedge correction, and the fifth column presents the flexibly fitted model at the corresponding tilt angles. **b**, Zoomed-in view of the final 3D density map displayed in orthogonal views, shown at two contour levels. **c**, Superimposition of the high contour level map from (b) onto its flexibly fitted model. **d**, Resolution evaluation of the final 3D density map using two criteria: Fourier shell correlation (FSC) between two-half maps reconstructed from the even and odd index of the tilted series and FSC between the final 3D map and the fitted structure model. The resolution for the former and latter criteria is evaluated at frequencies of 0.5 and 0.143, respectively.

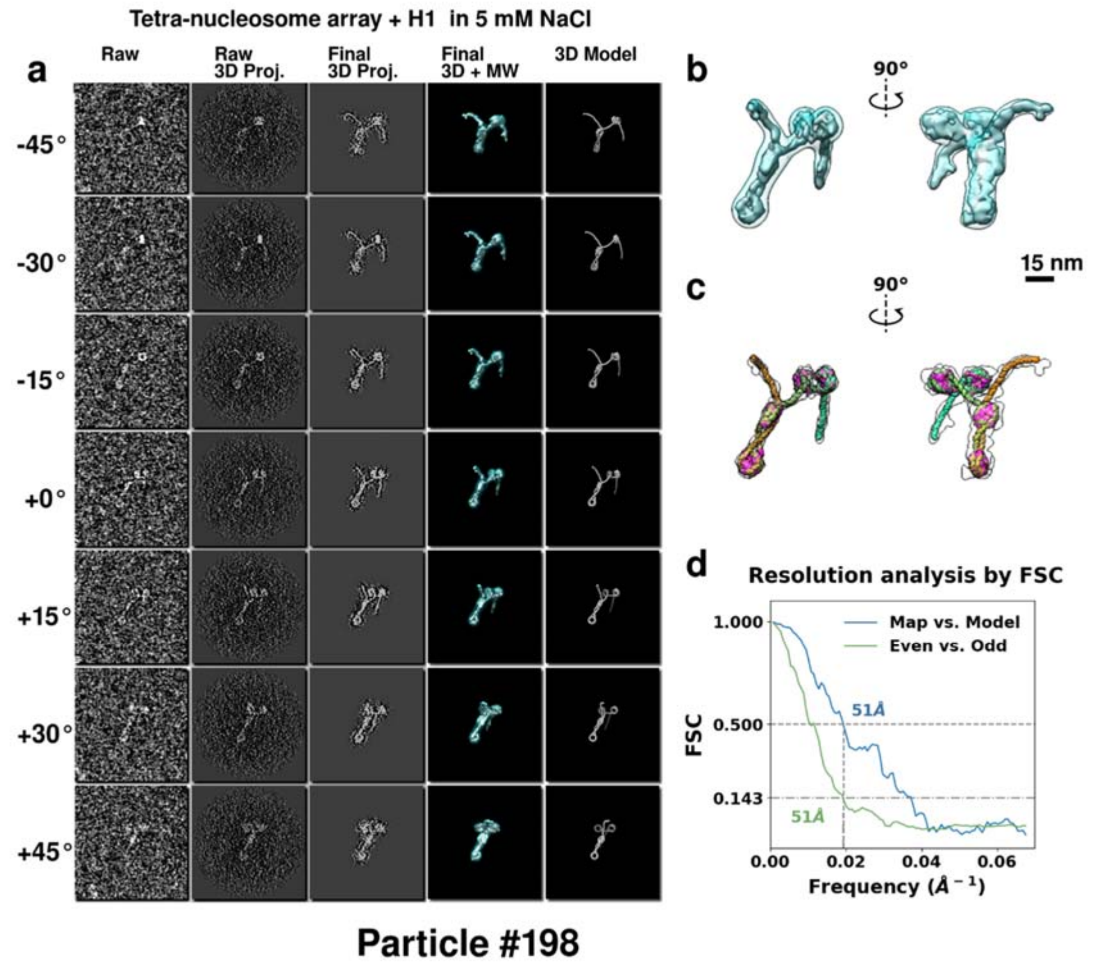

Supplementary Fig. 211. Cryo-ET 3D reconstruction of individual tetra-nucleosome particle (index no. 198) in 5 mM NaCl and presence of H1. a, IPET 3D reconstruction of individual tetra-nucleosome particles. The first column shows seven representative tilt images of an individual particle after CTF correction. Through alignment of the tilt images to a common center for 3D reconstruction via iterative refinement, the second and third columns display the 3D projections of the reconstruction before and after particle-shaped masking, respectively. The fourth column shows the final 3D reconstruction with missing wedge correction, and the fifth column presents the flexibly fitted model at the corresponding tilt angles. **b**, Zoomed-in view of the final 3D density map displayed in orthogonal views, shown at two contour levels. **c**, Superimposition of the high contour level map from (b) onto its flexibly fitted model. **d**, Resolution evaluation of the final 3D density map using two criteria: Fourier shell correlation (FSC) between two-half maps reconstructed from the even and odd index of the tilted series and FSC between the final 3D map and the fitted structure model. The resolution for the former and latter criteria is evaluated at frequencies of 0.5 and 0.143, respectively.

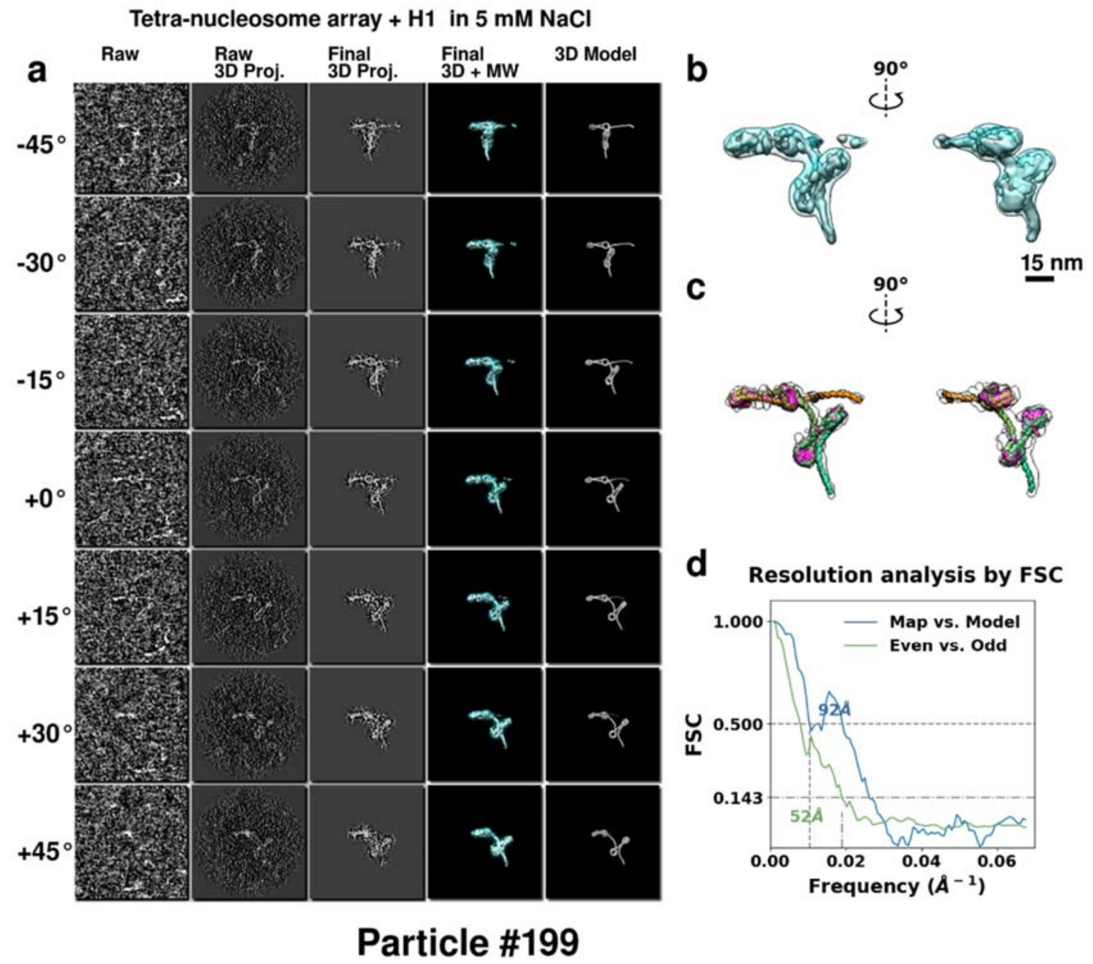

Supplementary Fig. 212. Cryo-ET 3D reconstruction of individual tetra-nucleosome particle (index no. 199) in 5 mM NaCl and presence of H1. a, IPET 3D reconstruction of individual tetra-nucleosome particles. The first column shows seven representative tilt images of an individual particle after CTF correction. Through alignment of the tilt images to a common center for 3D reconstruction via iterative refinement, the second and third columns display the 3D projections of the reconstruction before and after particle-shaped masking, respectively. The fourth column shows the final 3D reconstruction with missing wedge correction, and the fifth column presents the flexibly fitted model at the corresponding tilt angles. **b**, Zoomed-in view of the final 3D density map displayed in orthogonal views, shown at two contour levels. **c**, Superimposition of the high contour level map from (b) onto its flexibly fitted model. **d**, Resolution evaluation of the final 3D density map using two criteria: Fourier shell correlation (FSC) between two-half maps reconstructed from the even and odd index of the tilted series and FSC between the final 3D map and the fitted structure model. The resolution for the former and latter criteria is evaluated at frequencies of 0.5 and 0.143, respectively.

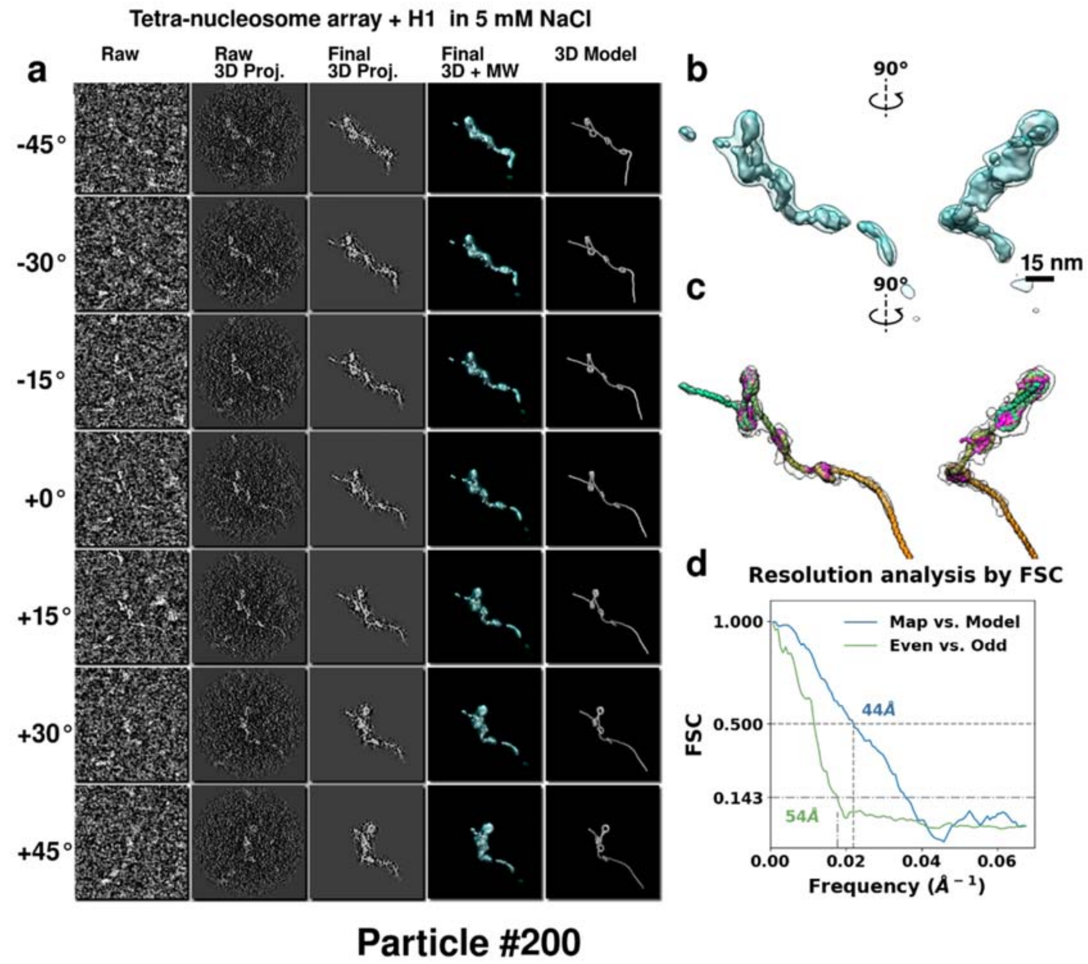

Supplementary Fig. 213. Cryo-ET 3D reconstruction of individual tetra-nucleosome particle (index no. 200) in 5 mM NaCl and presence of H1. a, IPET 3D reconstruction of individual tetra-nucleosome particles. The first column shows seven representative tilt images of an individual particle after CTF correction. Through alignment of the tilt images to a common center for 3D reconstruction via iterative refinement, the second and third columns display the 3D projections of the reconstruction before and after particle-shaped masking, respectively. The fourth column shows the final 3D reconstruction with missing wedge correction, and the fifth column presents the flexibly fitted model at the corresponding tilt angles. **b**, Zoomed-in view of the final 3D density map displayed in orthogonal views, shown at two contour levels. **c**, Superimposition of the high contour level map from (b) onto its flexibly fitted model. **d**, Resolution evaluation of the final 3D density map using two criteria: Fourier shell correlation (FSC) between two-half maps reconstructed from the even and odd index of the tilted series and FSC between the final 3D map and the fitted structure model. The resolution for the former and latter criteria is evaluated at frequencies of 0.5 and 0.143, respectively.

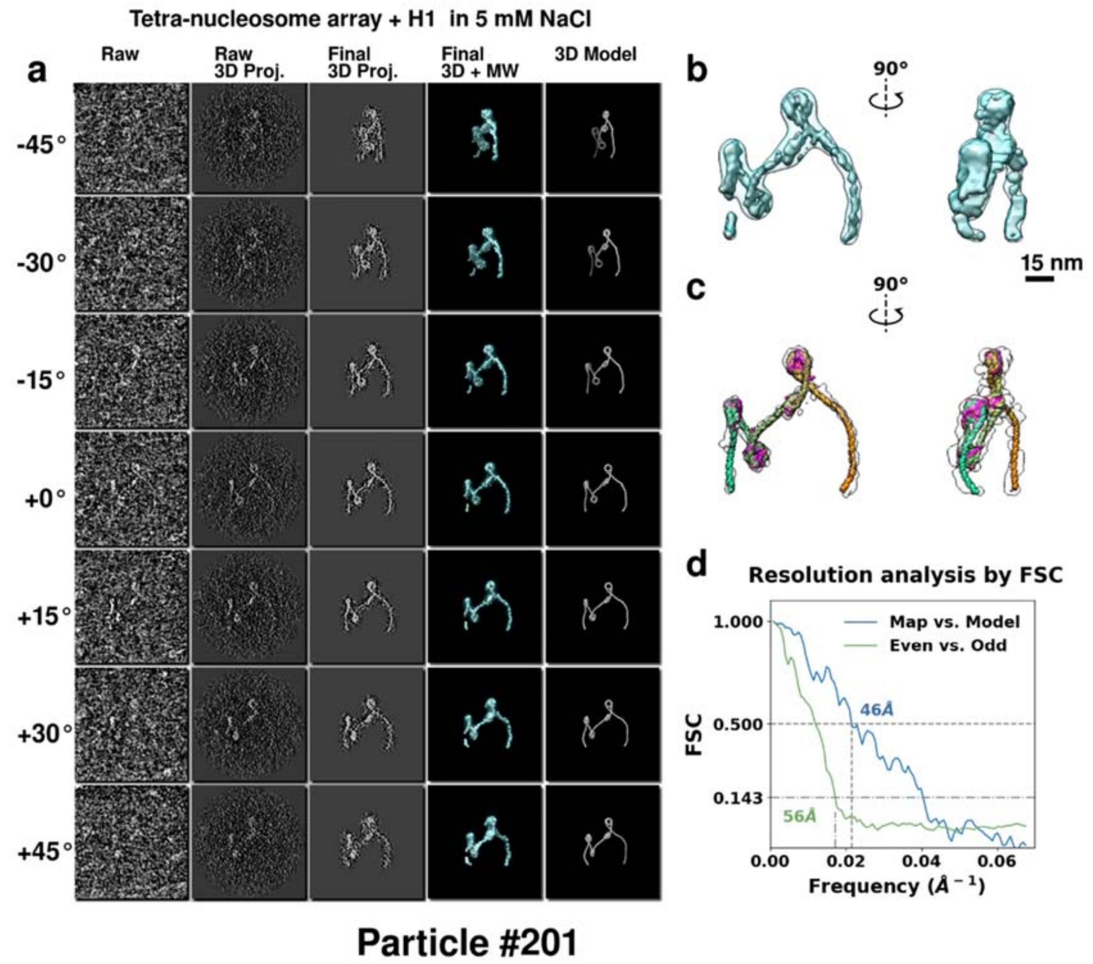

Supplementary Fig. 214. Cryo-ET 3D reconstruction of individual tetra-nucleosome particle (index no. 201) in 5 mM NaCl and presence of H1. a, IPET 3D reconstruction of individual tetra-nucleosome particles. The first column shows seven representative tilt images of an individual particle after CTF correction. Through alignment of the tilt images to a common center for 3D reconstruction via iterative refinement, the second and third columns display the 3D projections of the reconstruction before and after particle-shaped masking, respectively. The fourth column shows the final 3D reconstruction with missing wedge correction, and the fifth column presents the flexibly fitted model at the corresponding tilt angles. **b**, Zoomed-in view of the final 3D density map displayed in orthogonal views, shown at two contour levels. **c**, Superimposition of the high contour level map from (b) onto its flexibly fitted model. **d**, Resolution evaluation of the final 3D density map using two criteria: Fourier shell correlation (FSC) between two-half maps reconstructed from the even and odd index of the tilted series and FSC between the final 3D map and the fitted structure model. The resolution for the former and latter criteria is evaluated at frequencies of 0.5 and 0.143, respectively.

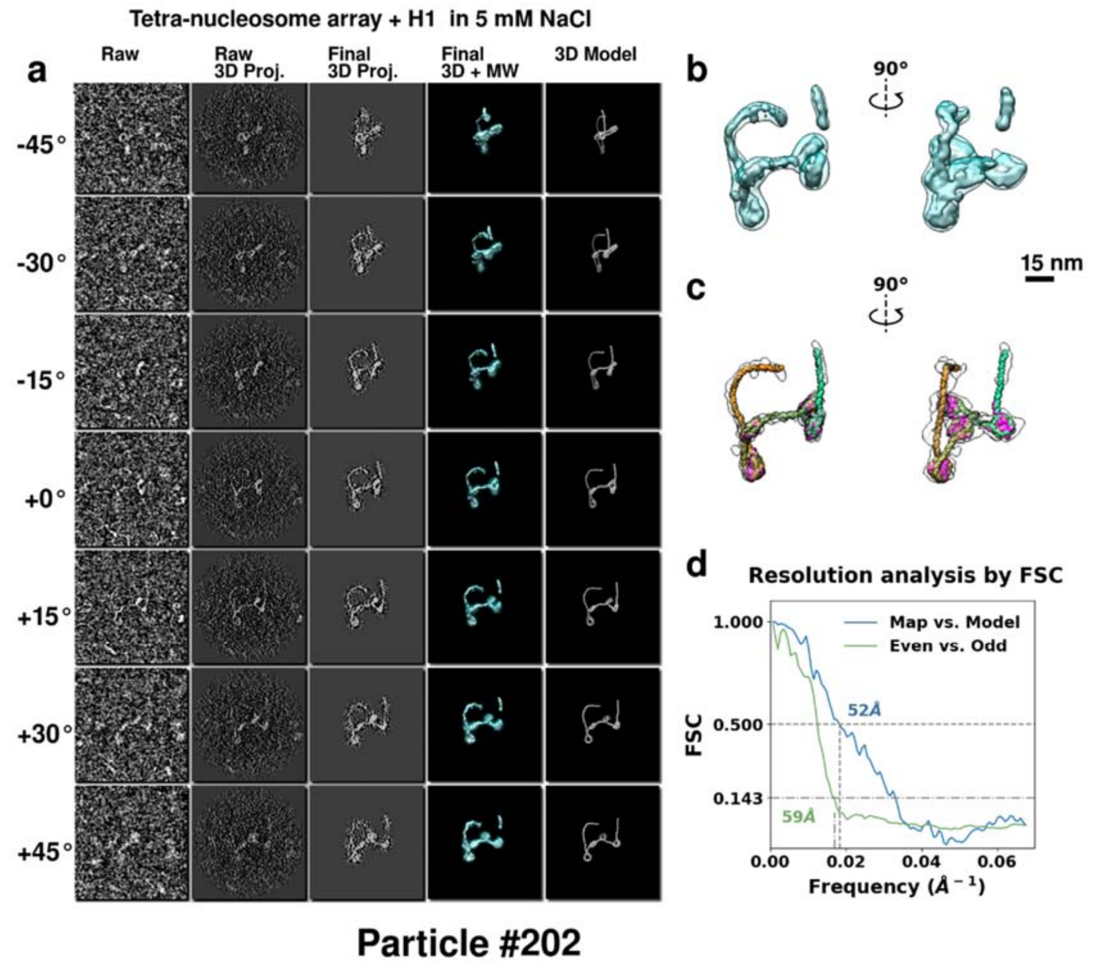

Supplementary Fig. 215. Cryo-ET 3D reconstruction of individual tetra-nucleosome particle (index no. 202) in 5 mM NaCl and presence of H1. a, IPET 3D reconstruction of individual tetra-nucleosome particles. The first column shows seven representative tilt images of an individual particle after CTF correction. Through alignment of the tilt images to a common center for 3D reconstruction via iterative refinement, the second and third columns display the 3D projections of the reconstruction before and after particle-shaped masking, respectively. The fourth column shows the final 3D reconstruction with missing wedge correction, and the fifth column presents the flexibly fitted model at the corresponding tilt angles. **b**, Zoomed-in view of the final 3D density map displayed in orthogonal views, shown at two contour levels. **c**, Superimposition of the high contour level map from (b) onto its flexibly fitted model. **d**, Resolution evaluation of the final 3D density map using two criteria: Fourier shell correlation (FSC) between two-half maps reconstructed from the even and odd index of the tilted series and FSC between the final 3D map and the fitted structure model. The resolution for the former and latter criteria is evaluated at frequencies of 0.5 and 0.143, respectively.

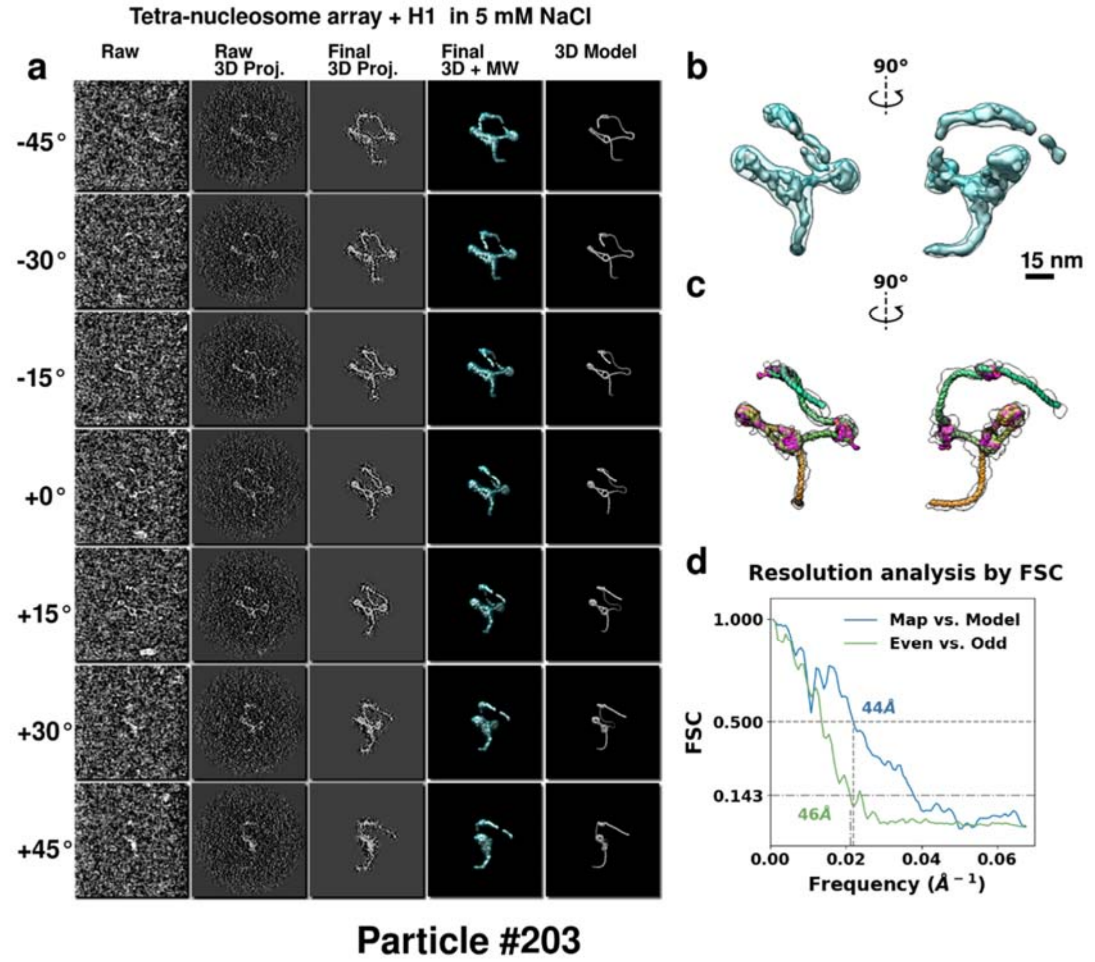

Supplementary Fig. 216. Cryo-ET 3D reconstruction of individual tetra-nucleosome particle (index no. 203) in 5 mM NaCl and presence of H1. a, IPET 3D reconstruction of individual tetra-nucleosome particles. The first column shows seven representative tilt images of an individual particle after CTF correction. Through alignment of the tilt images to a common center for 3D reconstruction via iterative refinement, the second and third columns display the 3D projections of the reconstruction before and after particle-shaped masking, respectively. The fourth column shows the final 3D reconstruction with missing wedge correction, and the fifth column presents the flexibly fitted model at the corresponding tilt angles. **b**, Zoomed-in view of the final 3D density map displayed in orthogonal views, shown at two contour levels. **c**, Superimposition of the high contour level map from (b) onto its flexibly fitted model. **d**, Resolution evaluation of the final 3D density map using two criteria: Fourier shell correlation (FSC) between two-half maps reconstructed from the even and odd index of the tilted series and FSC between the final 3D map and the fitted structure model. The resolution for the former and latter criteria is evaluated at frequencies of 0.5 and 0.143, respectively.

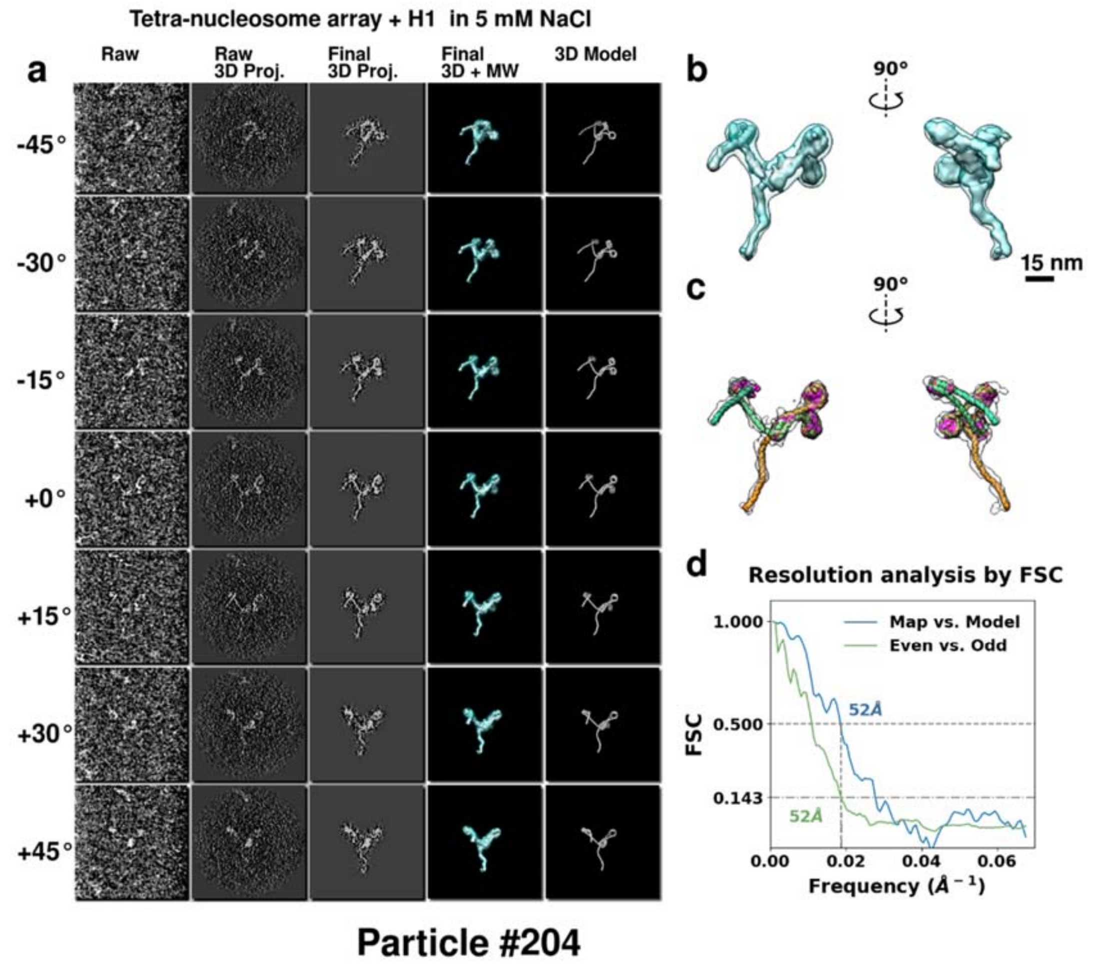

Supplementary Fig. 217. Cryo-ET 3D reconstruction of individual tetra-nucleosome particle (index no. 204) in 5 mM NaCl and presence of H1. a, IPET 3D reconstruction of individual tetra-nucleosome particles. The first column shows seven representative tilt images of an individual particle after CTF correction. Through alignment of the tilt images to a common center for 3D reconstruction via iterative refinement, the second and third columns display the 3D projections of the reconstruction before and after particle-shaped masking, respectively. The fourth column shows the final 3D reconstruction with missing wedge correction, and the fifth column presents the flexibly fitted model at the corresponding tilt angles. **b**, Zoomed-in view of the final 3D density map displayed in orthogonal views, shown at two contour levels. **c**, Superimposition of the high contour level map from (b) onto its flexibly fitted model. **d**, Resolution evaluation of the final 3D density map using two criteria: Fourier shell correlation (FSC) between two-half maps reconstructed from the even and odd index of the tilted series and FSC between the final 3D map and the fitted structure model. The resolution for the former and latter criteria is evaluated at frequencies of 0.5 and 0.143, respectively.

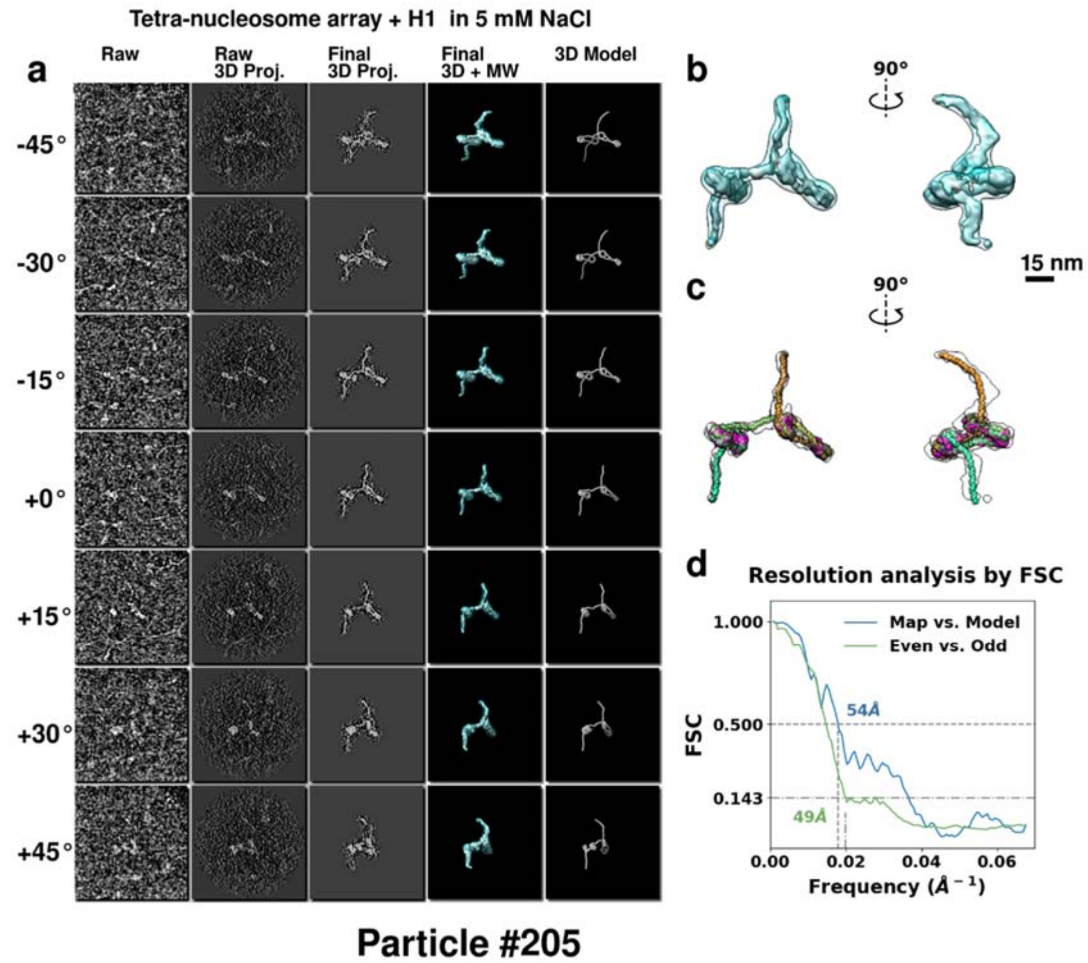

Supplementary Fig. 218. Cryo-ET 3D reconstruction of individual tetra-nucleosome particle (index no. 205) in 5 mM NaCl and presence of H1. a, IPET 3D reconstruction of individual tetra-nucleosome particles. The first column shows seven representative tilt images of an individual particle after CTF correction. Through alignment of the tilt images to a common center for 3D reconstruction via iterative refinement, the second and third columns display the 3D projections of the reconstruction before and after particle-shaped masking, respectively. The fourth column shows the final 3D reconstruction with missing wedge correction, and the fifth column presents the flexibly fitted model at the corresponding tilt angles. **b**, Zoomed-in view of the final 3D density map displayed in orthogonal views, shown at two contour levels. **c**, Superimposition of the high contour level map from (b) onto its flexibly fitted model. **d**, Resolution evaluation of the final 3D density map using two criteria: Fourier shell correlation (FSC) between two-half maps reconstructed from the even and odd index of the tilted series and FSC between the final 3D map and the fitted structure model. The resolution for the former and latter criteria is evaluated at frequencies of 0.5 and 0.143, respectively.

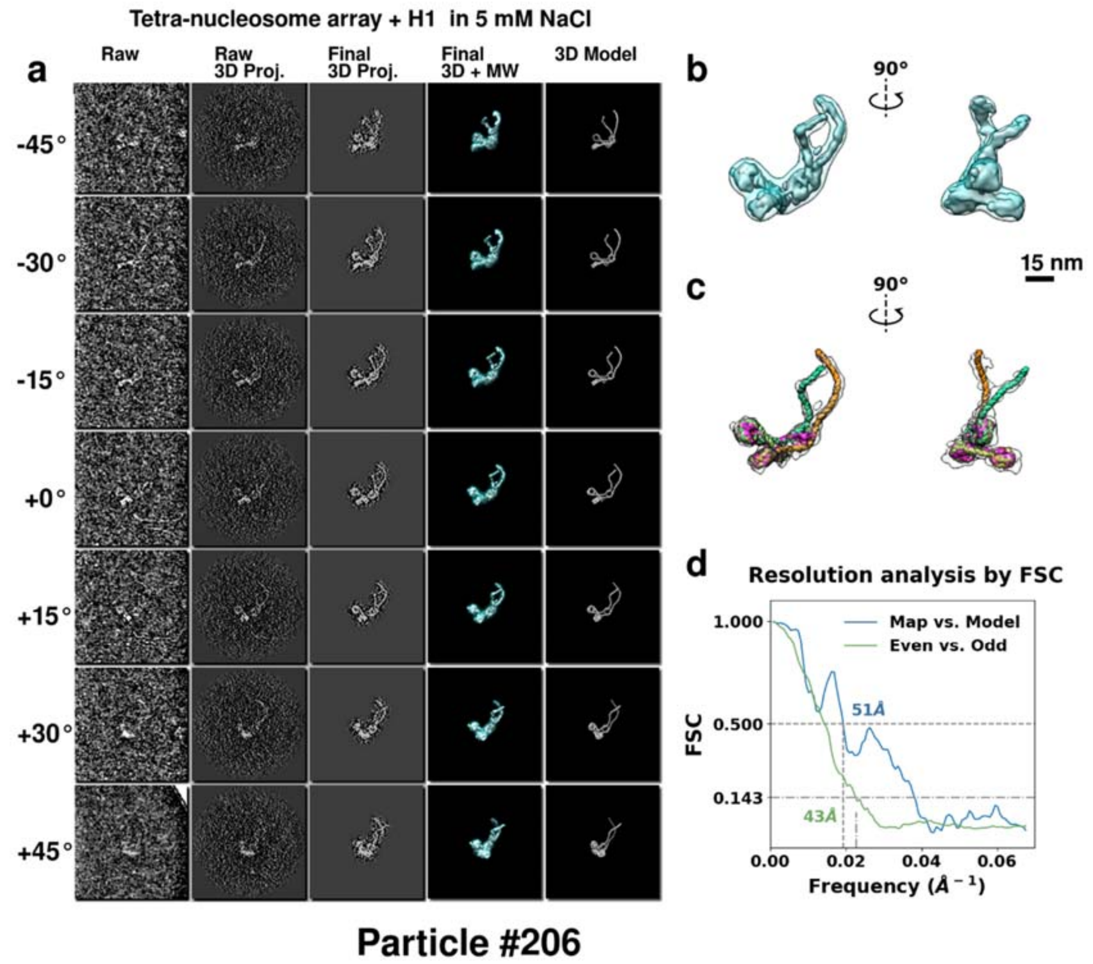

Supplementary Fig. 229. Cryo-ET 3D reconstruction of individual tetra-nucleosome particle (index no. 206) in 5 mM NaCl and presence of H1. a, IPET 3D reconstruction of individual tetra-nucleosome particles. The first column shows seven representative tilt images of an individual particle after CTF correction. Through alignment of the tilt images to a common center for 3D reconstruction via iterative refinement, the second and third columns display the 3D projections of the reconstruction before and after particle-shaped masking, respectively. The fourth column shows the final 3D reconstruction with missing wedge correction, and the fifth column presents the flexibly fitted model at the corresponding tilt angles. **b**, Zoomed-in view of the final 3D density map displayed in orthogonal views, shown at two contour levels. **c**, Superimposition of the high contour level map from (b) onto its flexibly fitted model. **d**, Resolution evaluation of the final 3D density map using two criteria: Fourier shell correlation (FSC) between two-half maps reconstructed from the even and odd index of the tilted series and FSC between the final 3D map and the fitted structure model. The resolution for the former and latter criteria is evaluated at frequencies of 0.5 and 0.143, respectively.

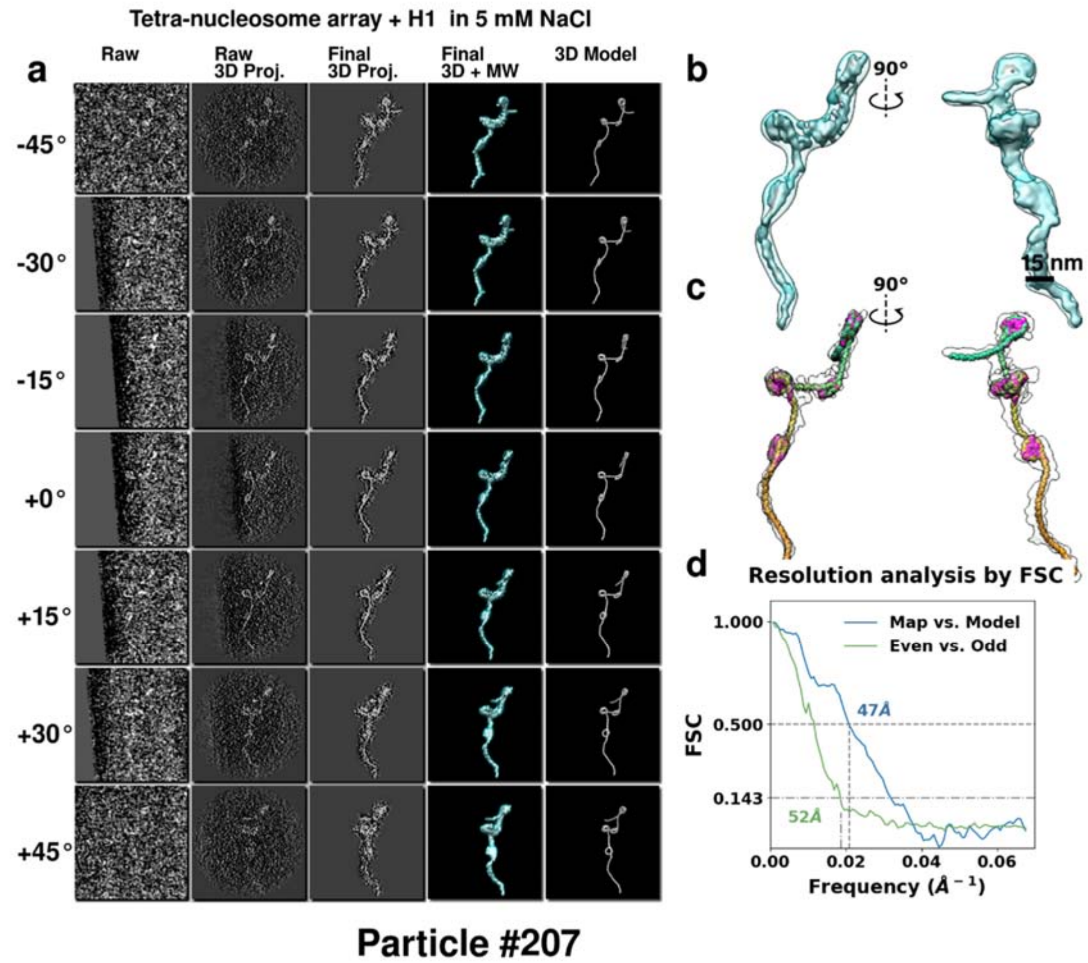

Supplementary Fig. 230. Cryo-ET 3D reconstruction of individual tetra-nucleosome particle (index no. 207) in 5 mM NaCl and presence of H1. a, IPET 3D reconstruction of individual tetra-nucleosome particles. The first column shows seven representative tilt images of an individual particle after CTF correction. Through alignment of the tilt images to a common center for 3D reconstruction via iterative refinement, the second and third columns display the 3D projections of the reconstruction before and after particle-shaped masking, respectively. The fourth column shows the final 3D reconstruction with missing wedge correction, and the fifth column presents the flexibly fitted model at the corresponding tilt angles. **b**, Zoomed-in view of the final 3D density map displayed in orthogonal views, shown at two contour levels. **c**, Superimposition of the high contour level map from (b) onto its flexibly fitted model. **d**, Resolution evaluation of the final 3D density map using two criteria: Fourier shell correlation (FSC) between two-half maps reconstructed from the even and odd index of the tilted series and FSC between the final 3D map and the fitted structure model. The resolution for the former and latter criteria is evaluated at frequencies of 0.5 and 0.143, respectively.

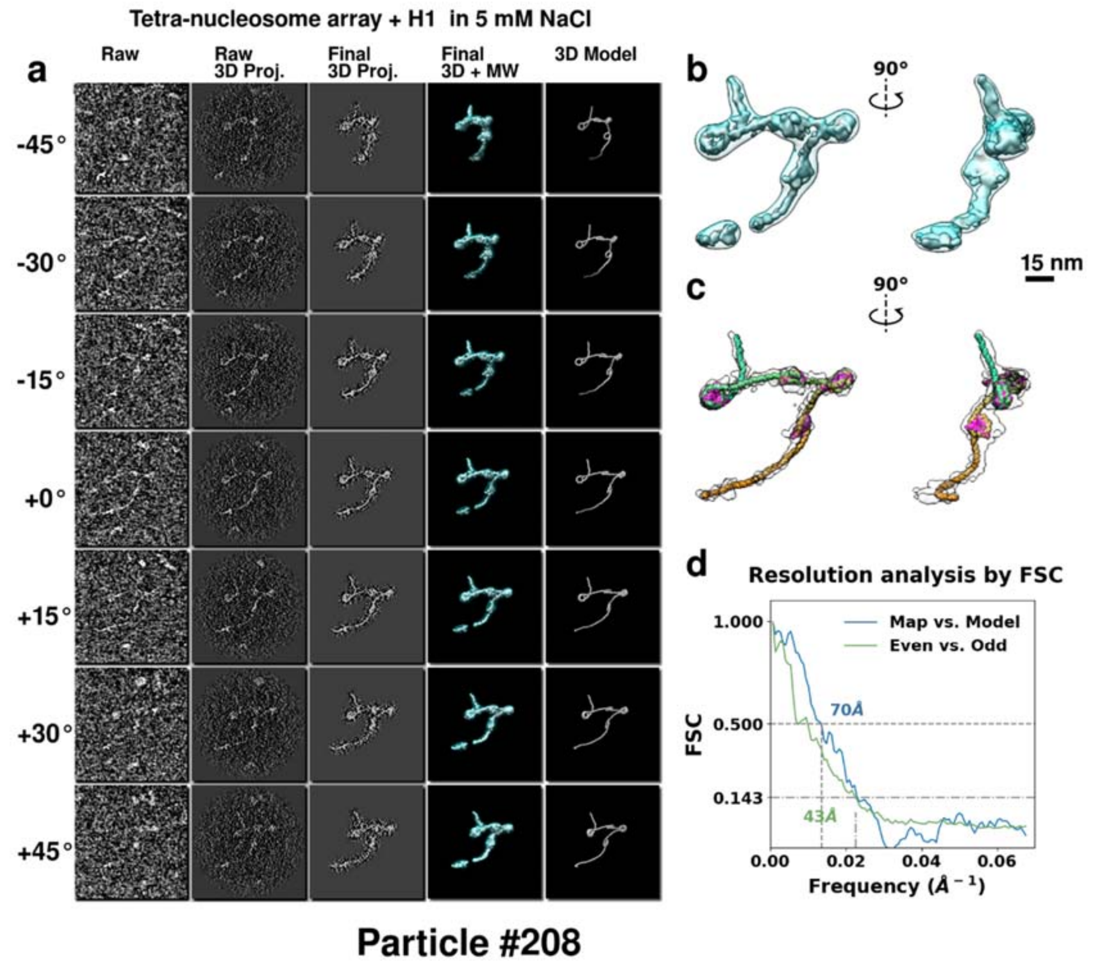

Supplementary Fig. 231. Cryo-ET 3D reconstruction of individual tetra-nucleosome particle (index no. 208) in 5 mM NaCl and presence of H1. a, IPET 3D reconstruction of individual tetra-nucleosome particles. The first column shows seven representative tilt images of an individual particle after CTF correction. Through alignment of the tilt images to a common center for 3D reconstruction via iterative refinement, the second and third columns display the 3D projections of the reconstruction before and after particle-shaped masking, respectively. The fourth column shows the final 3D reconstruction with missing wedge correction, and the fifth column presents the flexibly fitted model at the corresponding tilt angles. **b**, Zoomed-in view of the final 3D density map displayed in orthogonal views, shown at two contour levels. **c**, Superimposition of the high contour level map from (b) onto its flexibly fitted model. **d**, Resolution evaluation of the final 3D density map using two criteria: Fourier shell correlation (FSC) between two-half maps reconstructed from the even and odd index of the tilted series and FSC between the final 3D map and the fitted structure model. The resolution for the former and latter criteria is evaluated at frequencies of 0.5 and 0.143, respectively.

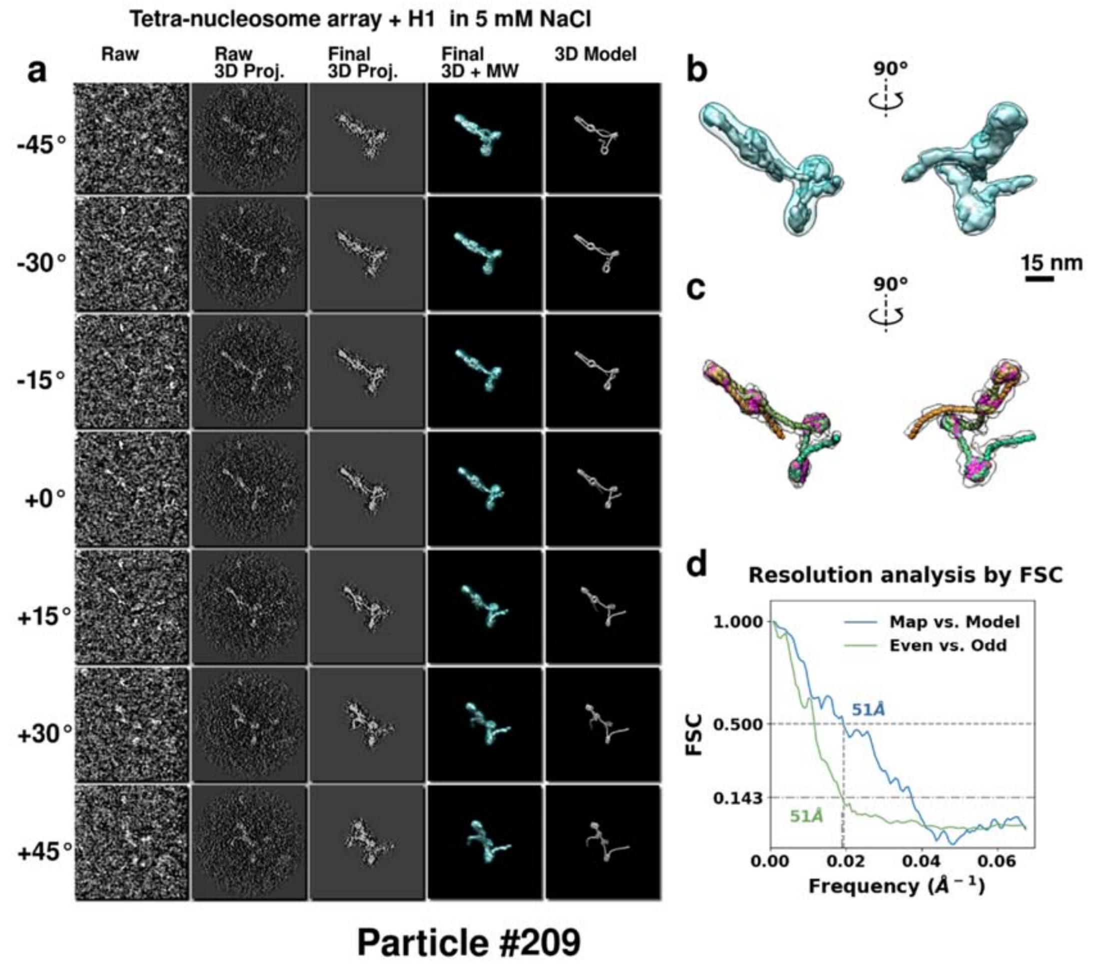

Supplementary Fig. 232. Cryo-ET 3D reconstruction of individual tetra-nucleosome particle (index no. 209) in 5 mM NaCl and presence of H1. a, IPET 3D reconstruction of individual tetra-nucleosome particles. The first column shows seven representative tilt images of an individual particle after CTF correction. Through alignment of the tilt images to a common center for 3D reconstruction via iterative refinement, the second and third columns display the 3D projections of the reconstruction before and after particle-shaped masking, respectively. The fourth column shows the final 3D reconstruction with missing wedge correction, and the fifth column presents the flexibly fitted model at the corresponding tilt angles. **b**, Zoomed-in view of the final 3D density map displayed in orthogonal views, shown at two contour levels. **c**, Superimposition of the high contour level map from (b) onto its flexibly fitted model. **d**, Resolution evaluation of the final 3D density map using two criteria: Fourier shell correlation (FSC) between two-half maps reconstructed from the even and odd index of the tilted series and FSC between the final 3D map and the fitted structure model. The resolution for the former and latter criteria is evaluated at frequencies of 0.5 and 0.143, respectively.

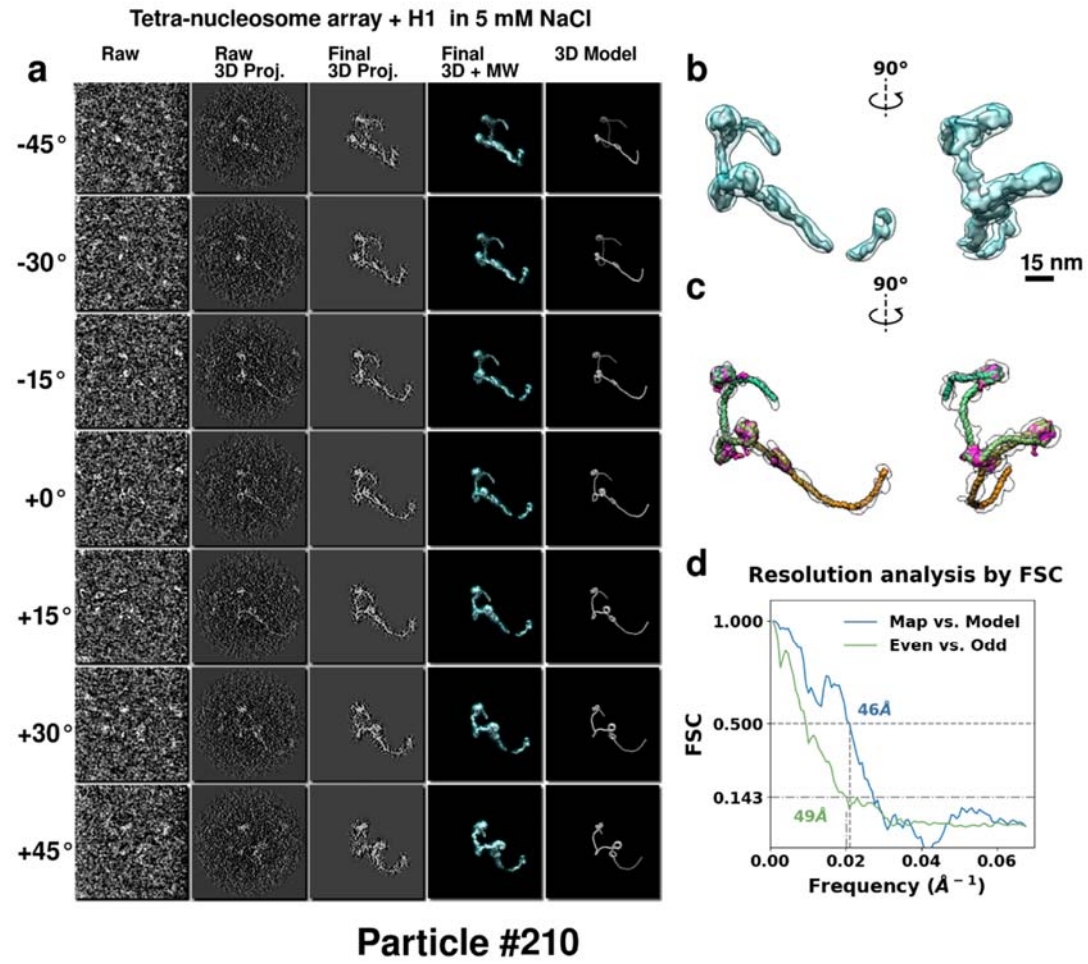

Supplementary Fig. 233. Cryo-ET 3D reconstruction of individual tetra-nucleosome particle (index no. 210) in 5 mM NaCl and presence of H1. a, IPET 3D reconstruction of individual tetra-nucleosome particles. The first column shows seven representative tilt images of an individual particle after CTF correction. Through alignment of the tilt images to a common center for 3D reconstruction via iterative refinement, the second and third columns display the 3D projections of the reconstruction before and after particle-shaped masking, respectively. The fourth column shows the final 3D reconstruction with missing wedge correction, and the fifth column presents the flexibly fitted model at the corresponding tilt angles. **b**, Zoomed-in view of the final 3D density map displayed in orthogonal views, shown at two contour levels. **c**, Superimposition of the high contour level map from (b) onto its flexibly fitted model. **d**, Resolution evaluation of the final 3D density map using two criteria: Fourier shell correlation (FSC) between two-half maps reconstructed from the even and odd index of the tilted series and FSC between the final 3D map and the fitted structure model. The resolution for the former and latter criteria is evaluated at frequencies of 0.5 and 0.143, respectively.

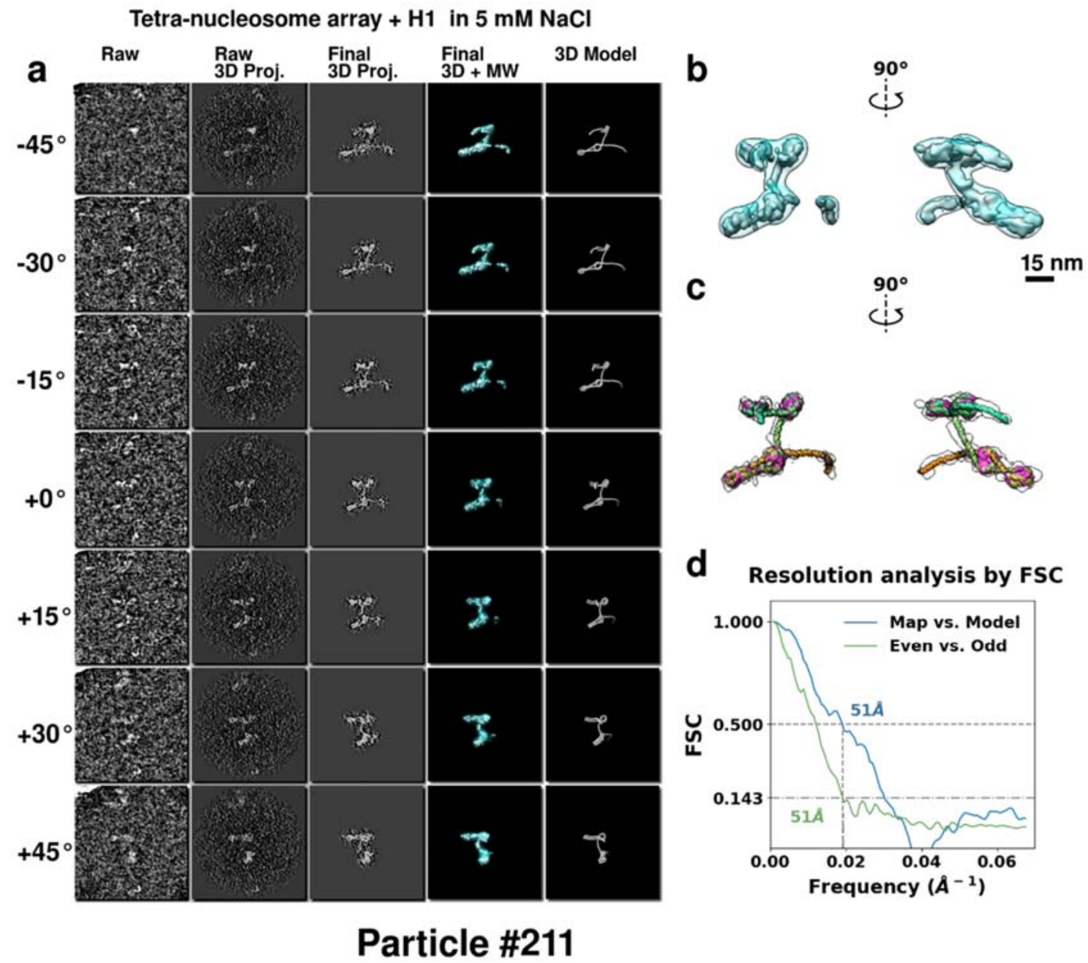

Supplementary Fig. 234. Cryo-ET 3D reconstruction of individual tetra-nucleosome particle (index no. 211) in 5 mM NaCl and presence of H1. a, IPET 3D reconstruction of individual tetra-nucleosome particles. The first column shows seven representative tilt images of an individual particle after CTF correction. Through alignment of the tilt images to a common center for 3D reconstruction via iterative refinement, the second and third columns display the 3D projections of the reconstruction before and after particle-shaped masking, respectively. The fourth column shows the final 3D reconstruction with missing wedge correction, and the fifth column presents the flexibly fitted model at the corresponding tilt angles. **b**, Zoomed-in view of the final 3D density map displayed in orthogonal views, shown at two contour levels. **c**, Superimposition of the high contour level map from (b) onto its flexibly fitted model. **d**, Resolution evaluation of the final 3D density map using two criteria: Fourier shell correlation (FSC) between two-half maps reconstructed from the even and odd index of the tilted series and FSC between the final 3D map and the fitted structure model. The resolution for the former and latter criteria is evaluated at frequencies of 0.5 and 0.143, respectively.

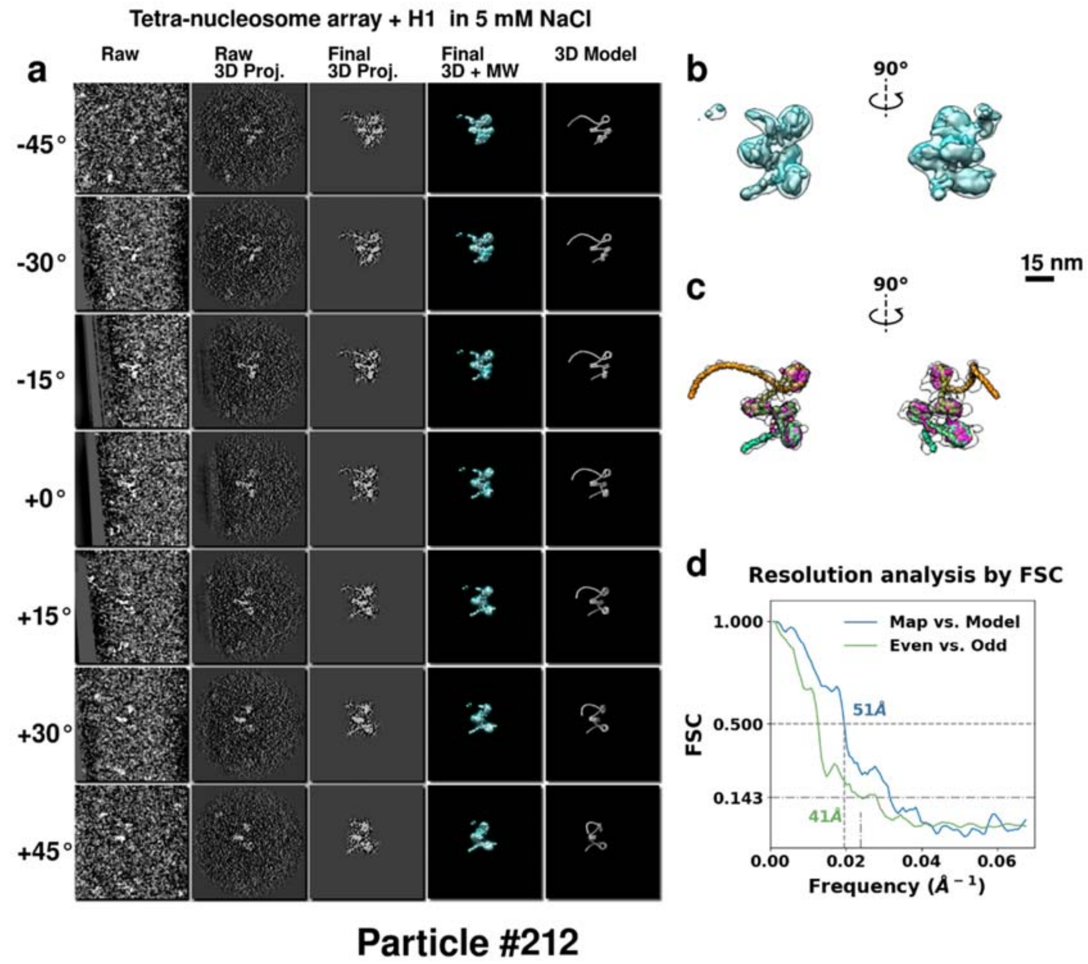

Supplementary Fig. 235. Cryo-ET 3D reconstruction of individual tetra-nucleosome particle (index no. 212) in 5 mM NaCl and presence of H1. a, IPET 3D reconstruction of individual tetra-nucleosome particles. The first column shows seven representative tilt images of an individual particle after CTF correction. Through alignment of the tilt images to a common center for 3D reconstruction via iterative refinement, the second and third columns display the 3D projections of the reconstruction before and after particle-shaped masking, respectively. The fourth column shows the final 3D reconstruction with missing wedge correction, and the fifth column presents the flexibly fitted model at the corresponding tilt angles. **b**, Zoomed-in view of the final 3D density map displayed in orthogonal views, shown at two contour levels. **c**, Superimposition of the high contour level map from (b) onto its flexibly fitted model. **d**, Resolution evaluation of the final 3D density map using two criteria: Fourier shell correlation (FSC) between two-half maps reconstructed from the even and odd index of the tilted series and FSC between the final 3D map and the fitted structure model. The resolution for the former and latter criteria is evaluated at frequencies of 0.5 and 0.143, respectively.

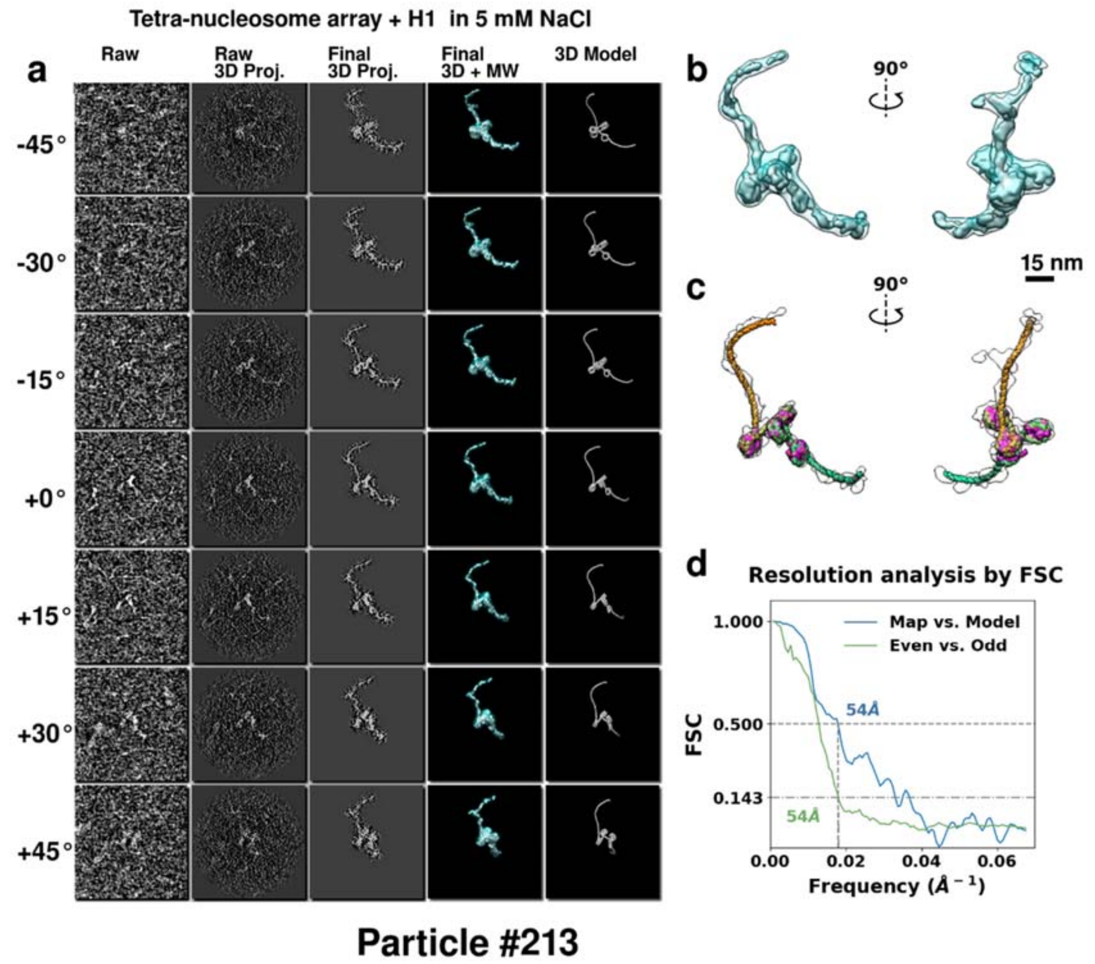

Supplementary Fig. 236. Cryo-ET 3D reconstruction of individual tetra-nucleosome particle (index no. 213) in 5 mM NaCl and presence of H1. a, IPET 3D reconstruction of individual tetra-nucleosome particles. The first column shows seven representative tilt images of an individual particle after CTF correction. Through alignment of the tilt images to a common center for 3D reconstruction via iterative refinement, the second and third columns display the 3D projections of the reconstruction before and after particle-shaped masking, respectively. The fourth column shows the final 3D reconstruction with missing wedge correction, and the fifth column presents the flexibly fitted model at the corresponding tilt angles. **b**, Zoomed-in view of the final 3D density map displayed in orthogonal views, shown at two contour levels. **c**, Superimposition of the high contour level map from (b) onto its flexibly fitted model. **d**, Resolution evaluation of the final 3D density map using two criteria: Fourier shell correlation (FSC) between two-half maps reconstructed from the even and odd index of the tilted series and FSC between the final 3D map and the fitted structure model. The resolution for the former and latter criteria is evaluated at frequencies of 0.5 and 0.143, respectively.

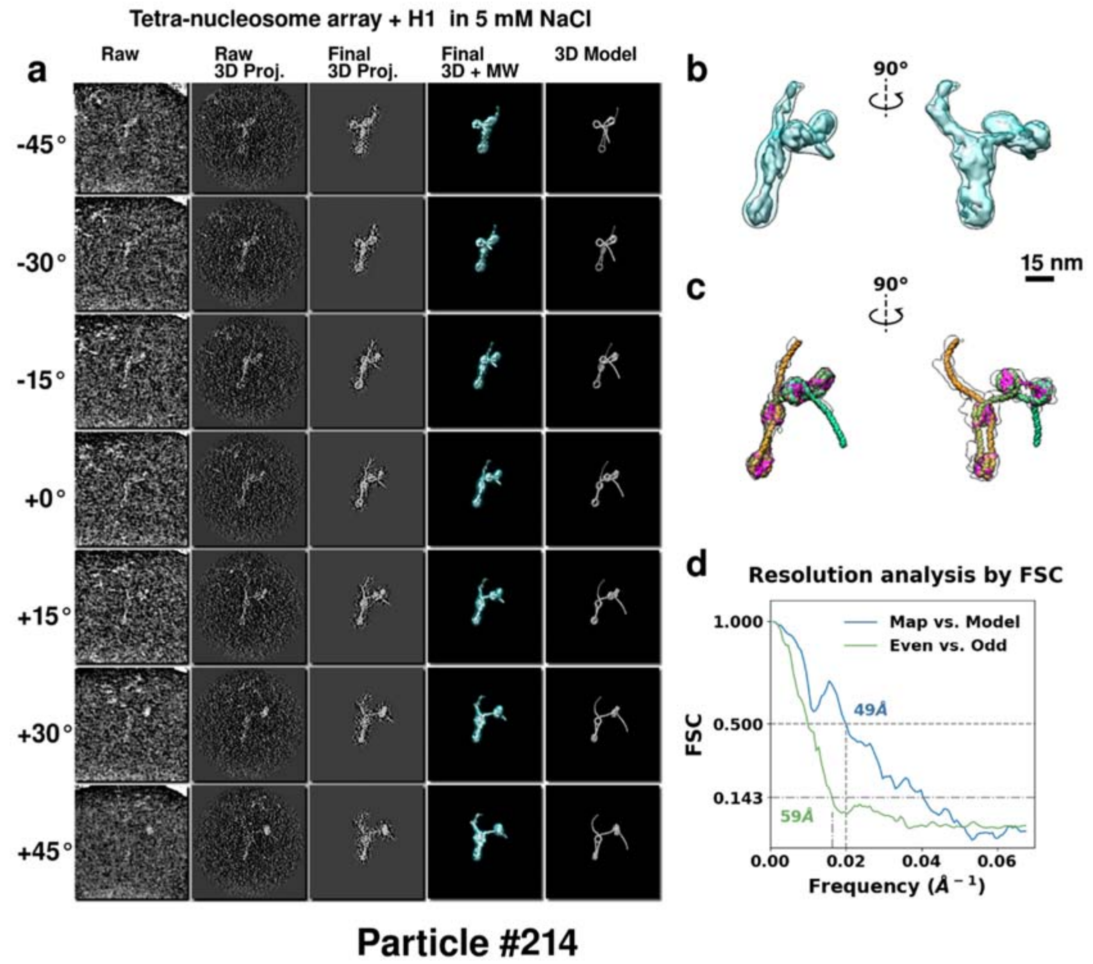

Supplementary Fig. 237. Cryo-ET 3D reconstruction of individual tetra-nucleosome particle (index no. 214) in 5 mM NaCl and presence of H1. a, IPET 3D reconstruction of individual tetra-nucleosome particles. The first column shows seven representative tilt images of an individual particle after CTF correction. Through alignment of the tilt images to a common center for 3D reconstruction via iterative refinement, the second and third columns display the 3D projections of the reconstruction before and after particle-shaped masking, respectively. The fourth column shows the final 3D reconstruction with missing wedge correction, and the fifth column presents the flexibly fitted model at the corresponding tilt angles. **b**, Zoomed-in view of the final 3D density map displayed in orthogonal views, shown at two contour levels. **c**, Superimposition of the high contour level map from (b) onto its flexibly fitted model. **d**, Resolution evaluation of the final 3D density map using two criteria: Fourier shell correlation (FSC) between two-half maps reconstructed from the even and odd index of the tilted series and FSC between the final 3D map and the fitted structure model. The resolution for the former and latter criteria is evaluated at frequencies of 0.5 and 0.143, respectively.

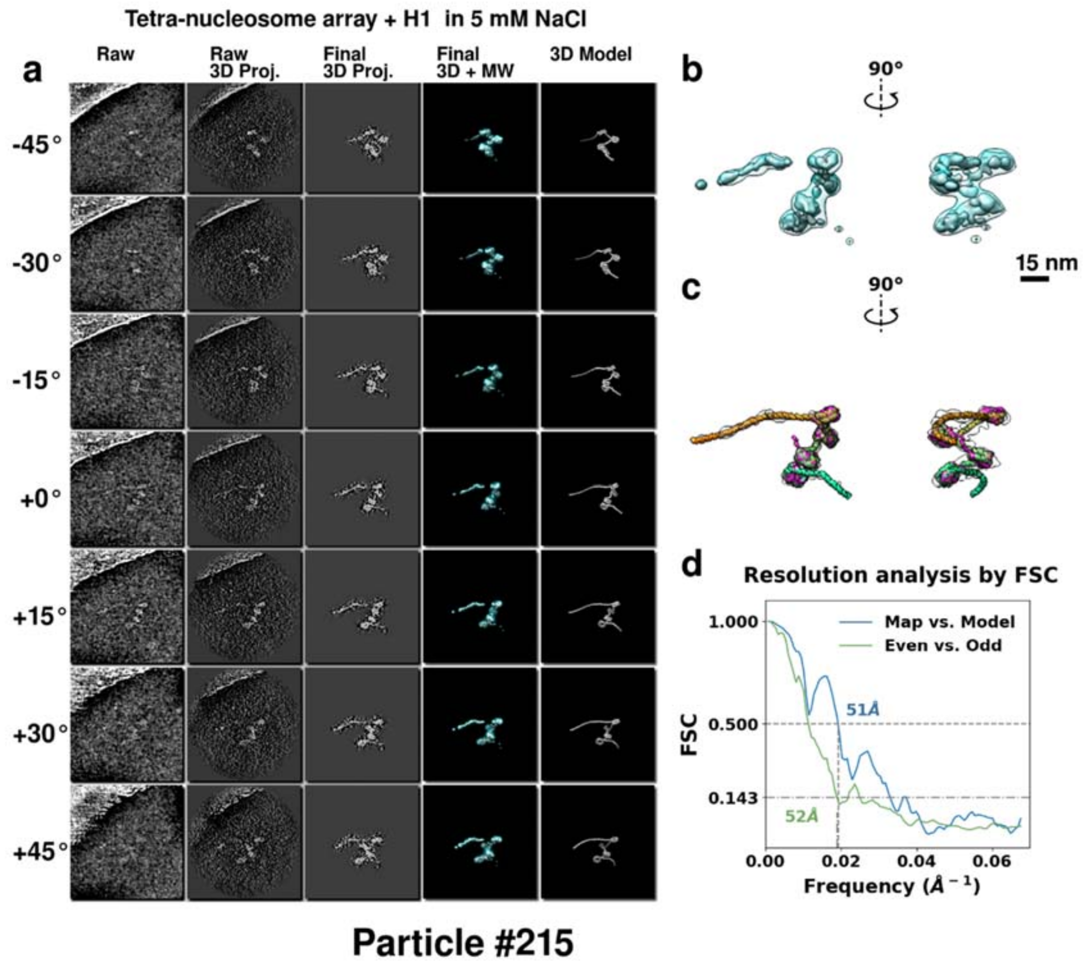

Supplementary Fig. 238. Cryo-ET 3D reconstruction of individual tetra-nucleosome particle (index no. 215) in 5 mM NaCl and presence of H1. a, IPET 3D reconstruction of individual tetra-nucleosome particles. The first column shows seven representative tilt images of an individual particle after CTF correction. Through alignment of the tilt images to a common center for 3D reconstruction via iterative refinement, the second and third columns display the 3D projections of the reconstruction before and after particle-shaped masking, respectively. The fourth column shows the final 3D reconstruction with missing wedge correction, and the fifth column presents the flexibly fitted model at the corresponding tilt angles. **b**, Zoomed-in view of the final 3D density map displayed in orthogonal views, shown at two contour levels. **c**, Superimposition of the high contour level map from (b) onto its flexibly fitted model. **d**, Resolution evaluation of the final 3D density map using two criteria: Fourier shell correlation (FSC) between two-half maps reconstructed from the even and odd index of the tilted series and FSC between the final 3D map and the fitted structure model. The resolution for the former and latter criteria is evaluated at frequencies of 0.5 and 0.143, respectively.

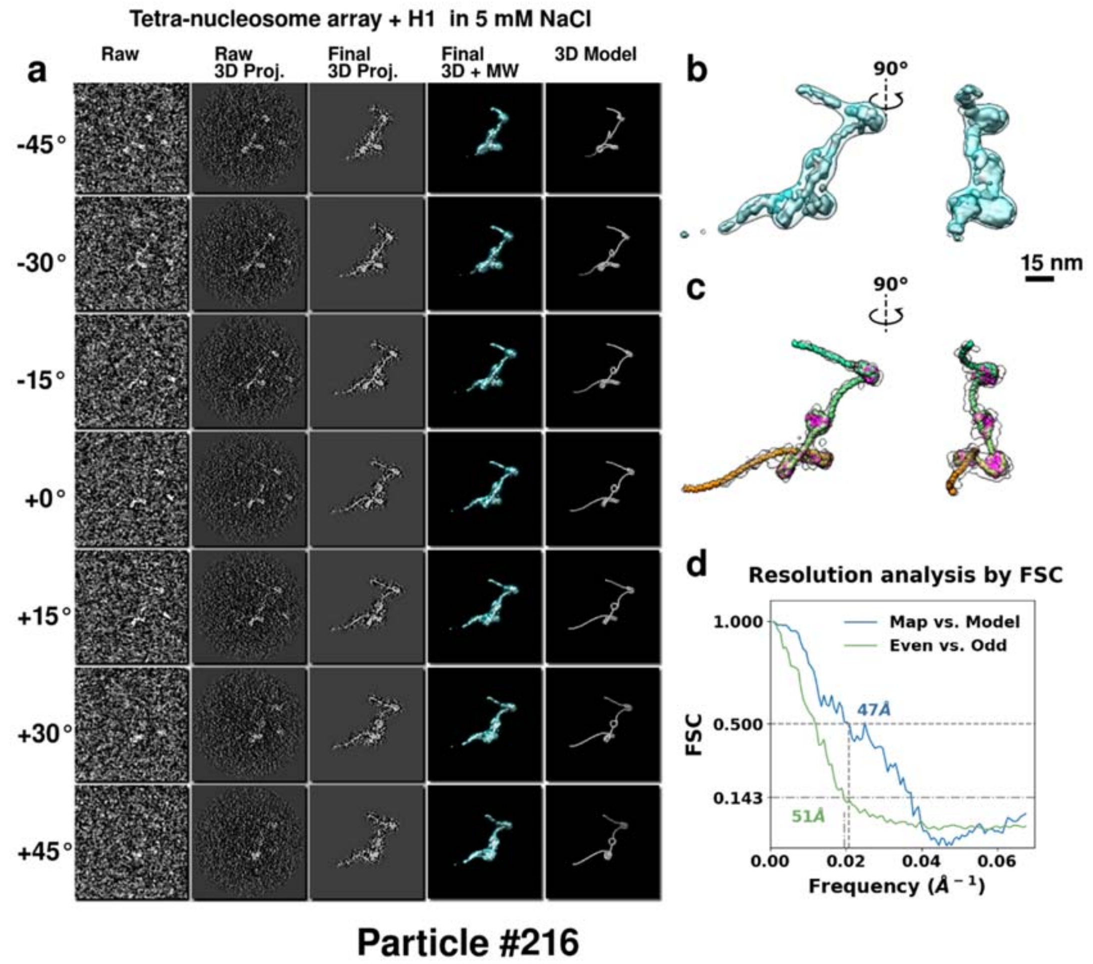

Supplementary Fig. 239. Cryo-ET 3D reconstruction of individual tetra-nucleosome particle (index no. 216) in 5 mM NaCl and presence of H1. a, IPET 3D reconstruction of individual tetra-nucleosome particles. The first column shows seven representative tilt images of an individual particle after CTF correction. Through alignment of the tilt images to a common center for 3D reconstruction via iterative refinement, the second and third columns display the 3D projections of the reconstruction before and after particle-shaped masking, respectively. The fourth column shows the final 3D reconstruction with missing wedge correction, and the fifth column presents the flexibly fitted model at the corresponding tilt angles. **b**, Zoomed-in view of the final 3D density map displayed in orthogonal views, shown at two contour levels. **c**, Superimposition of the high contour level map from (b) onto its flexibly fitted model. **d**, Resolution evaluation of the final 3D density map using two criteria: Fourier shell correlation (FSC) between two-half maps reconstructed from the even and odd index of the tilted series and FSC between the final 3D map and the fitted structure model. The resolution for the former and latter criteria is evaluated at frequencies of 0.5 and 0.143, respectively.

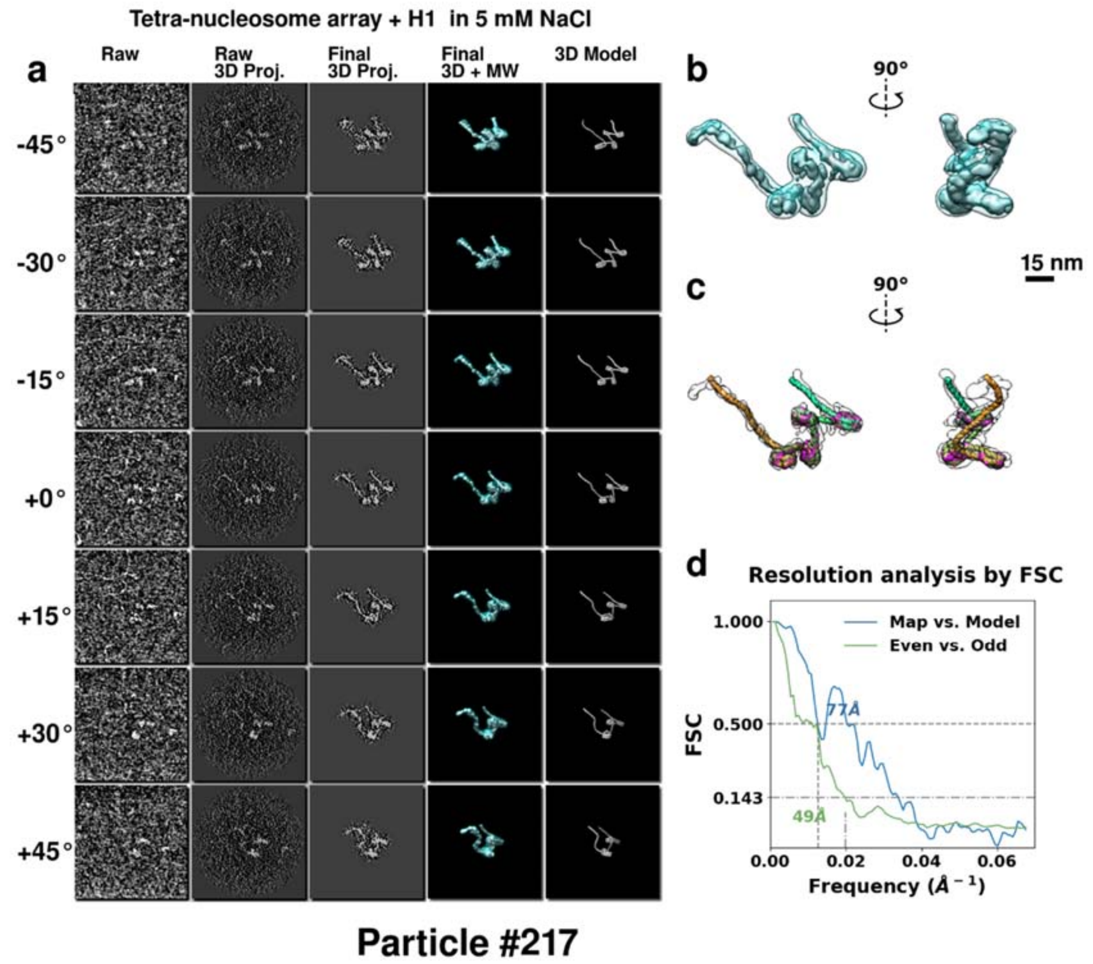

Supplementary Fig. 240. Cryo-ET 3D reconstruction of individual tetra-nucleosome particle (index no. 217) in 5 mM NaCl and presence of H1. a, IPET 3D reconstruction of individual tetra-nucleosome particles. The first column shows seven representative tilt images of an individual particle after CTF correction. Through alignment of the tilt images to a common center for 3D reconstruction via iterative refinement, the second and third columns display the 3D projections of the reconstruction before and after particle-shaped masking, respectively. The fourth column shows the final 3D reconstruction with missing wedge correction, and the fifth column presents the flexibly fitted model at the corresponding tilt angles. **b**, Zoomed-in view of the final 3D density map displayed in orthogonal views, shown at two contour levels. **c**, Superimposition of the high contour level map from (b) onto its flexibly fitted model. **d**, Resolution evaluation of the final 3D density map using two criteria: Fourier shell correlation (FSC) between two-half maps reconstructed from the even and odd index of the tilted series and FSC between the final 3D map and the fitted structure model. The resolution for the former and latter criteria is evaluated at frequencies of 0.5 and 0.143, respectively.

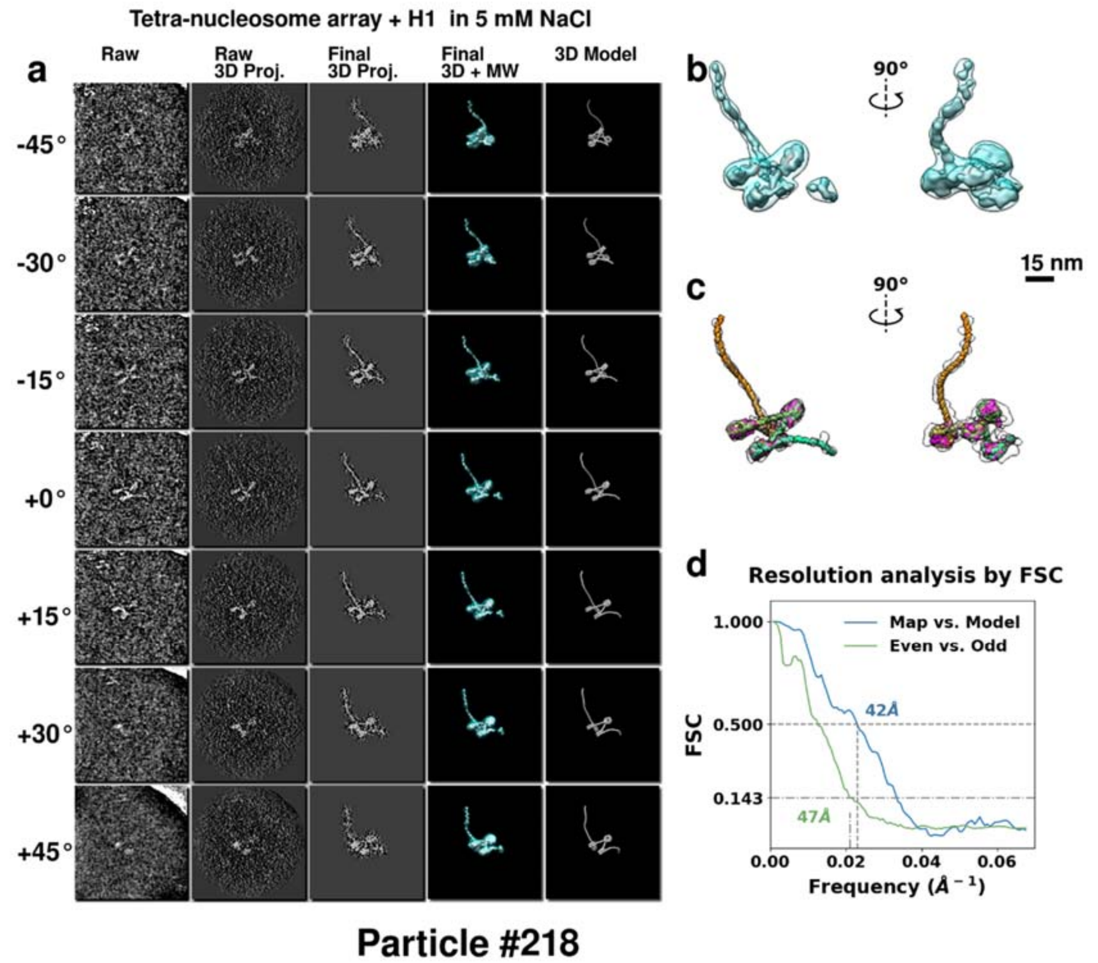

Supplementary Fig. 241. Cryo-ET 3D reconstruction of individual tetra-nucleosome particle (index no. 218) in 5 mM NaCl and presence of H1. a, IPET 3D reconstruction of individual tetra-nucleosome particles. The first column shows seven representative tilt images of an individual particle after CTF correction. Through alignment of the tilt images to a common center for 3D reconstruction via iterative refinement, the second and third columns display the 3D projections of the reconstruction before and after particle-shaped masking, respectively. The fourth column shows the final 3D reconstruction with missing wedge correction, and the fifth column presents the flexibly fitted model at the corresponding tilt angles. **b**, Zoomed-in view of the final 3D density map displayed in orthogonal views, shown at two contour levels. **c**, Superimposition of the high contour level map from (b) onto its flexibly fitted model. **d**, Resolution evaluation of the final 3D density map using two criteria: Fourier shell correlation (FSC) between two-half maps reconstructed from the even and odd index of the tilted series and FSC between the final 3D map and the fitted structure model. The resolution for the former and latter criteria is evaluated at frequencies of 0.5 and 0.143, respectively.

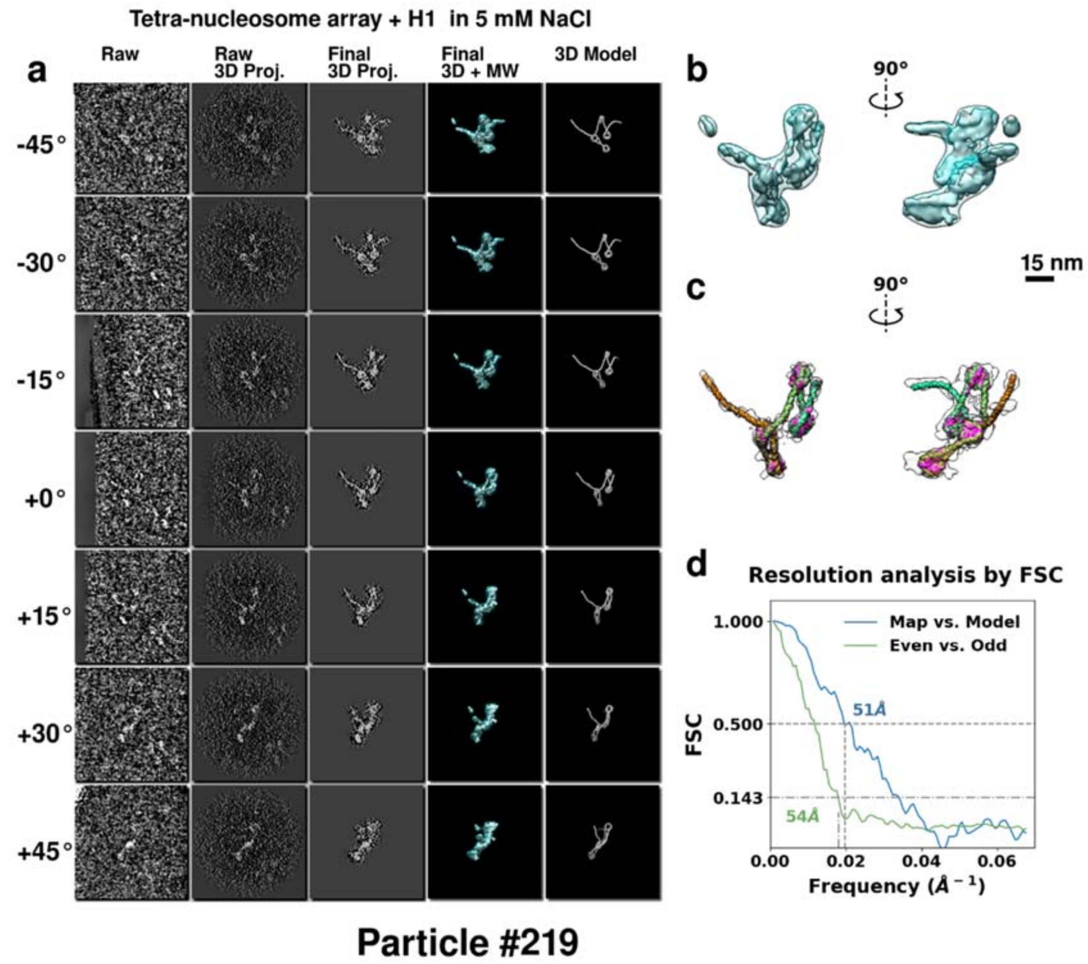

Supplementary Fig. 242. Cryo-ET 3D reconstruction of individual tetra-nucleosome particle (index no. 219) in 5 mM NaCl and presence of H1. a, IPET 3D reconstruction of individual tetra-nucleosome particles. The first column shows seven representative tilt images of an individual particle after CTF correction. Through alignment of the tilt images to a common center for 3D reconstruction via iterative refinement, the second and third columns display the 3D projections of the reconstruction before and after particle-shaped masking, respectively. The fourth column shows the final 3D reconstruction with missing wedge correction, and the fifth column presents the flexibly fitted model at the corresponding tilt angles. **b**, Zoomed-in view of the final 3D density map displayed in orthogonal views, shown at two contour levels. **c**, Superimposition of the high contour level map from (b) onto its flexibly fitted model. **d**, Resolution evaluation of the final 3D density map using two criteria: Fourier shell correlation (FSC) between two-half maps reconstructed from the even and odd index of the tilted series and FSC between the final 3D map and the fitted structure model. The resolution for the former and latter criteria is evaluated at frequencies of 0.5 and 0.143, respectively.

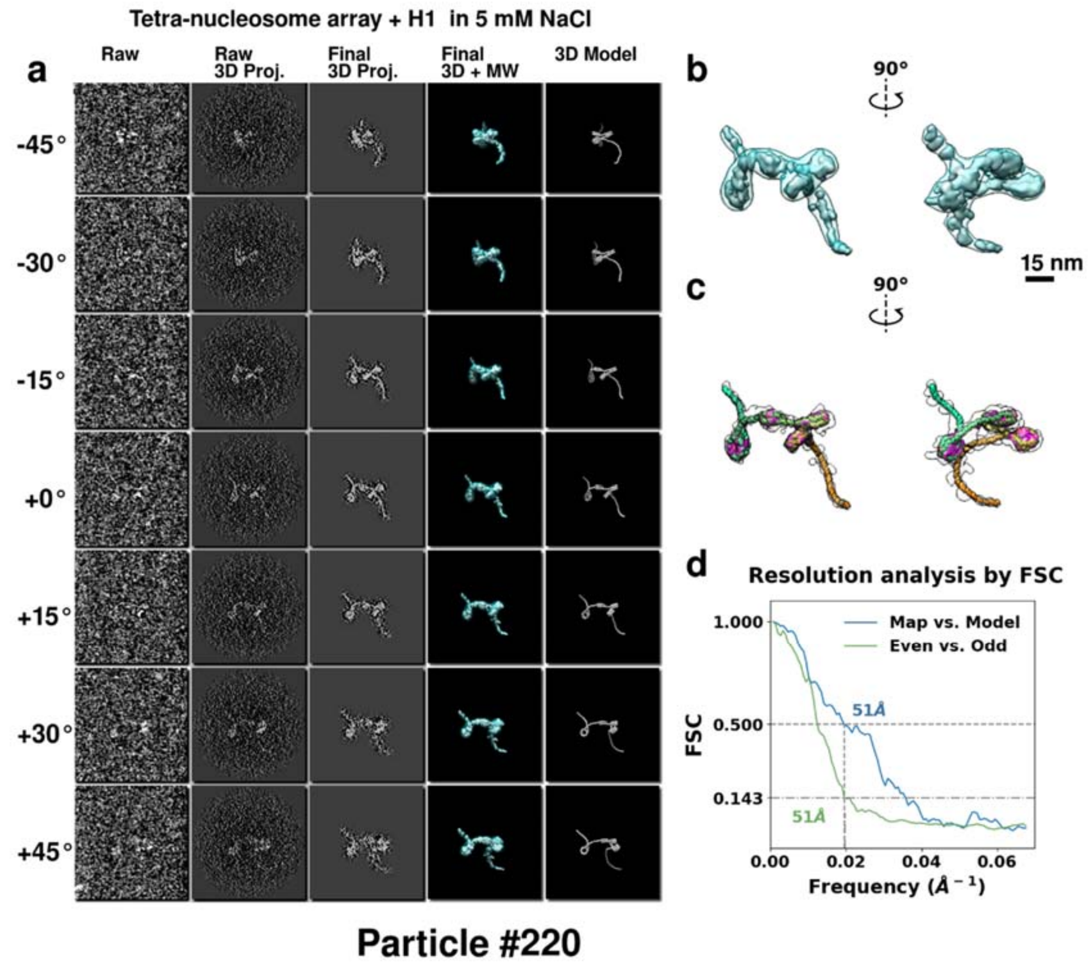

Supplementary Fig. 243. Cryo-ET 3D reconstruction of individual tetra-nucleosome particle (index no. 220) in 5 mM NaCl and presence of H1. a, IPET 3D reconstruction of individual tetra-nucleosome particles. The first column shows seven representative tilt images of an individual particle after CTF correction. Through alignment of the tilt images to a common center for 3D reconstruction via iterative refinement, the second and third columns display the 3D projections of the reconstruction before and after particle-shaped masking, respectively. The fourth column shows the final 3D reconstruction with missing wedge correction, and the fifth column presents the flexibly fitted model at the corresponding tilt angles. **b**, Zoomed-in view of the final 3D density map displayed in orthogonal views, shown at two contour levels. **c**, Superimposition of the high contour level map from (b) onto its flexibly fitted model. **d**, Resolution evaluation of the final 3D density map using two criteria: Fourier shell correlation (FSC) between two-half maps reconstructed from the even and odd index of the tilted series and FSC between the final 3D map and the fitted structure model. The resolution for the former and latter criteria is evaluated at frequencies of 0.5 and 0.143, respectively.

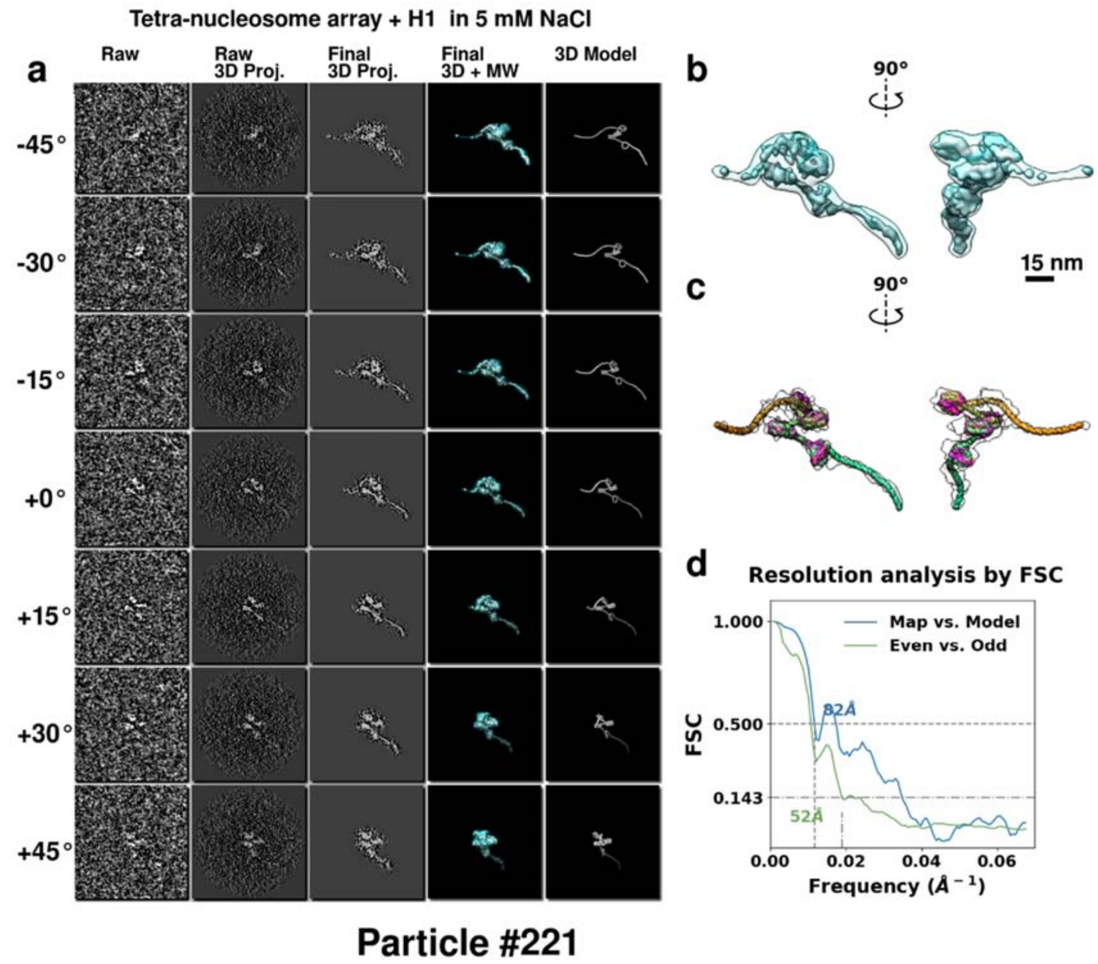

Supplementary Fig. 244. Cryo-ET 3D reconstruction of individual tetra-nucleosome particle (index no. 221) in 5 mM NaCl and presence of H1. a, IPET 3D reconstruction of individual tetra-nucleosome particles. The first column shows seven representative tilt images of an individual particle after CTF correction. Through alignment of the tilt images to a common center for 3D reconstruction via iterative refinement, the second and third columns display the 3D projections of the reconstruction before and after particle-shaped masking, respectively. The fourth column shows the final 3D reconstruction with missing wedge correction, and the fifth column presents the flexibly fitted model at the corresponding tilt angles. **b**, Zoomed-in view of the final 3D density map displayed in orthogonal views, shown at two contour levels. **c**, Superimposition of the high contour level map from (b) onto its flexibly fitted model. **d**, Resolution evaluation of the final 3D density map using two criteria: Fourier shell correlation (FSC) between two-half maps reconstructed from the even and odd index of the tilted series and FSC between the final 3D map and the fitted structure model. The resolution for the former and latter criteria is evaluated at frequencies of 0.5 and 0.143, respectively.

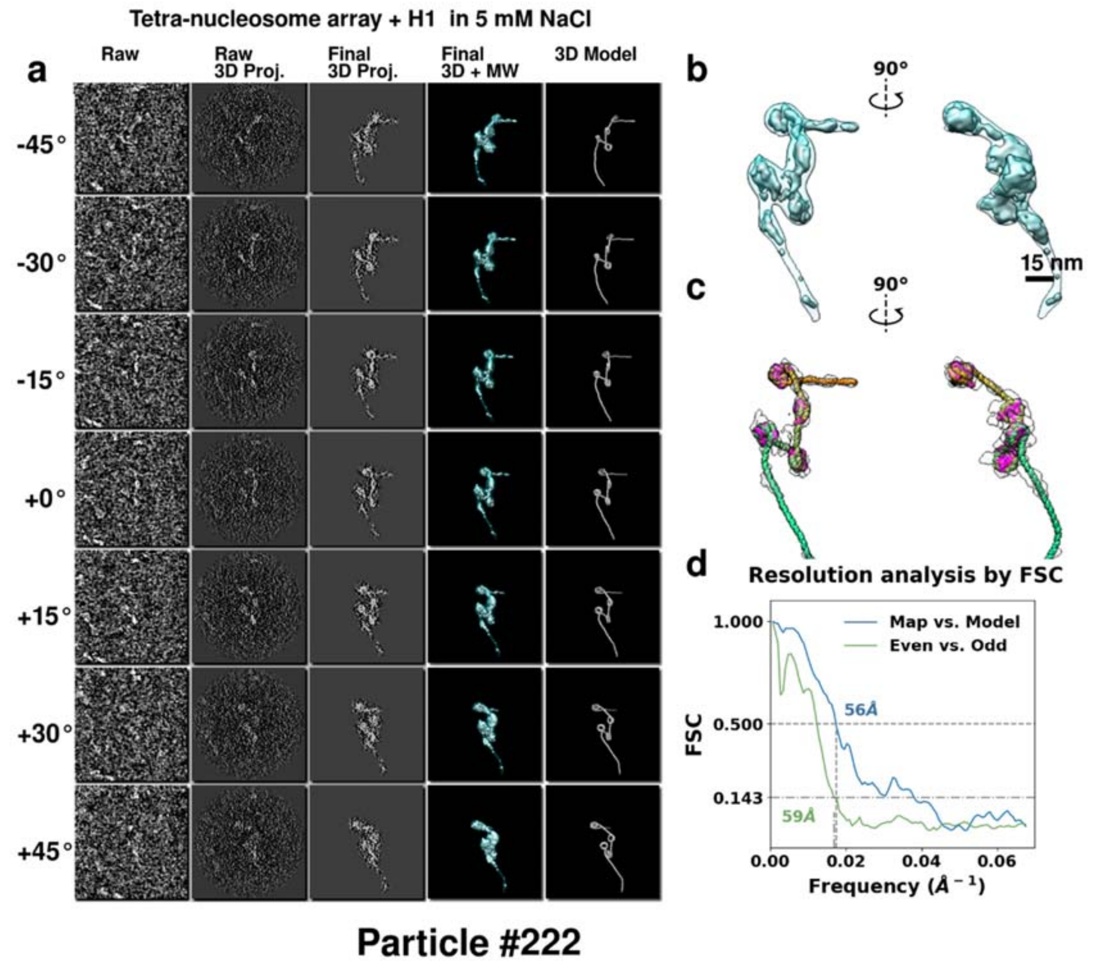

Supplementary Fig. 245. Cryo-ET 3D reconstruction of individual tetra-nucleosome particle (index no. 222) in 5 mM NaCl and presence of H1. a, IPET 3D reconstruction of individual tetra-nucleosome particles. The first column shows seven representative tilt images of an individual particle after CTF correction. Through alignment of the tilt images to a common center for 3D reconstruction via iterative refinement, the second and third columns display the 3D projections of the reconstruction before and after particle-shaped masking, respectively. The fourth column shows the final 3D reconstruction with missing wedge correction, and the fifth column presents the flexibly fitted model at the corresponding tilt angles. **b**, Zoomed-in view of the final 3D density map displayed in orthogonal views, shown at two contour levels. **c**, Superimposition of the high contour level map from (b) onto its flexibly fitted model. **d**, Resolution evaluation of the final 3D density map using two criteria: Fourier shell correlation (FSC) between two-half maps reconstructed from the even and odd index of the tilted series and FSC between the final 3D map and the fitted structure model. The resolution for the former and latter criteria is evaluated at frequencies of 0.5 and 0.143, respectively.

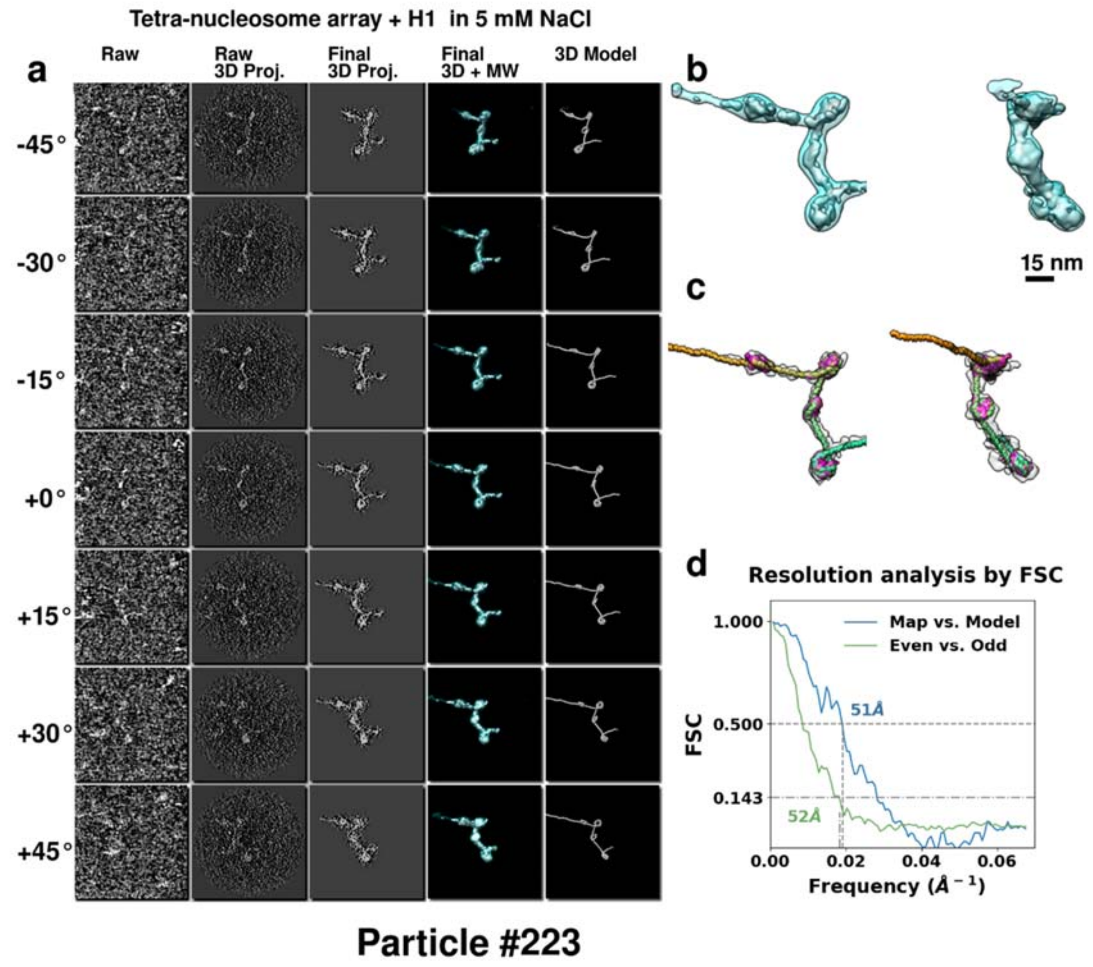

Supplementary Fig. 246. Cryo-ET 3D reconstruction of individual tetra-nucleosome particle (index no. 223) in 5 mM NaCl and presence of H1. a, IPET 3D reconstruction of individual tetra-nucleosome particles. The first column shows seven representative tilt images of an individual particle after CTF correction. Through alignment of the tilt images to a common center for 3D reconstruction via iterative refinement, the second and third columns display the 3D projections of the reconstruction before and after particle-shaped masking, respectively. The fourth column shows the final 3D reconstruction with missing wedge correction, and the fifth column presents the flexibly fitted model at the corresponding tilt angles. **b**, Zoomed-in view of the final 3D density map displayed in orthogonal views, shown at two contour levels. **c**, Superimposition of the high contour level map from (b) onto its flexibly fitted model. **d**, Resolution evaluation of the final 3D density map using two criteria: Fourier shell correlation (FSC) between two-half maps reconstructed from the even and odd index of the tilted series and FSC between the final 3D map and the fitted structure model. The resolution for the former and latter criteria is evaluated at frequencies of 0.5 and 0.143, respectively.
