# Supplementary material for: Angle between DNA linker and nucleosome core particle regulates array compaction revealed by individual-particle cryo-electron tomography
Source: Nat Commun. 2024 May 23;15:4395. doi: 10.1038/s41467-024-48305-1 (PMC11116431; doi:10.1038/s41467-024-48305-1)
Supplement: Supplementary file 2 — Description of Additional Supplementary Files [file 41467_2024_48305_MOESM2_ESM.pdf]

Supplementary Data 1:
Detailed information on IPET 3D reconstructions of all 223 particles. All data were acquired at 300 kV high-tension under a defocus within the range of 2.5-3.5 µm. For 3D reconstruction, no symmetry was imposed; no initial model was human involved. Each 3D reconstruction was achieved by refinement of the images acquired from one particle without any averaging.

Supplementary Data 2:
Statistics of Structural Variety. This includes the structural variety measured from experiments, along with the parameters used to generate the simulated hectanucleosome array fibers and mini-chromosomes. "*TwoGaussian.Fitting*" refers to the distribution fitted by two Gaussian functions using the *sklearn.mixture.GaussianMixture* package.

Supplementary Movie 1:
3D structural diversity of mono-nucleosome in 5 mM Na^+. Individual-particle 3D structures reveal the structural diversity of mono-nucleosome in 5 mM Na^+.

Supplementary Movie 2:
3D structural diversities of di-, tri-, and tetra-nucleosome arrays in 5 mM Na^+. Individual-particle 3D structures reveal the structural diversity of di-nucleosome, tri-nucleosome, and tetra-nucleosome arrays at 5 mM Na^+.

Supplementary Movie 3:
Conformational changes in tetra-nucleosome induced by 50 mM Na^+ and H1. Individual-particle 3D structures reveal conformational changes in tetra-nucleosome arrays induced by increased Na^+ concentration and in the presence of H1.
